# Supplementary material for: Photochemical C3-amination of pyridines via Zincke imine intermediates
Source: Nat Commun. 2025 May 31;16:5072. doi: 10.1038/s41467-025-59809-9 (PMC12126555; doi:10.1038/s41467-025-59809-9)
Supplement: Supplementary file 1 — Supplementary Information [file 41467_2025_59809_MOESM1_ESM.pdf]

Supplementary Information

Photochemical C3-Amination of Pyridines via Zincke Imine Intermediates

Kitti Franciska Szabó,+[a] Piotr Banachowicz,+[a] Antoni Powała,[a,b] Danijela Lunic,[c] Ignacio Funes Ardoiz,*[c] and Dorota Gryko*[a]

[a]*Institute of Organic Chemistry Polish Academy of Sciences; Kasprzaka 44/52, 01-224 Warsaw, Poland; correspondence: dorota.gryko@icho.edu.pl*

[b]*Department of Chemistry Warsaw University of Technology, Noakowskiego 3, 00-664 Warsaw, Poland*

[c]*Complejo Científico-Tecnológico Universidad de La Rioja, Madre de Dios, 53, 26004 Logroño, La Rioja, Spain; correspondence:ignacio.funesa@unirioja.es*

correspondence:

dorota.gryko@icho.edu.pl

ignacio.funesa@unirioja.es

Table of Contents

1. General information **11**

1.1. Reaction set-up **12**

1.2. Preparation of the model reaction system **13**

1.3. Model reaction **16**

1.4. Preliminary results **18**

1.4.1 Photofunctionalization of the Zincke imine 18

1.4.2 Control experiments 18

1.4.3. Initial conditions 19

1.4.3. Optimisation studies 19

2. Mechanistic investigations **24**

2.1. UV-VIS spectroscopy of the starting material **24**

2.2. Kinetic studies **25**

2.3. Regioselectivity of the model reaction **26**

2.4. Characterization and reactivity of functionalized Zincke imine 2a **27**

3. Preparation of substrates **29**

3.1. Preparation of substituted pyridines **29**

3.2. Preparation of Zincke imines **35**

3.3. Preparation of pyridinium salts **45**

3.3.1. Preparation of *N*,4-dimethylbenzenesulfonohydrazide (from *N*-methylhydrazine) 45

3.3.2. Preparation of *N*,4-dimethylbenzenesulfonohydrazide (from *N*-methylhydrazine sulphate) 45

3.3.3. Preparation of *N*-Aminopirydinium salt (Py-salt)[2] 45

3.4 Experiments with DMPO and TEMPO radical traps **46**

3.4.1 DMPO radical trap 46

3.4.2. TEMPO radical trap 47

4. Photocatalysed functionalization of Zincke imines **48**

4.1. Isolation and characterization of reaction intermediate 2a/2b **48**

4.2. General Protocol for photoamination and closure of the Zincke imine (General Procedure 3) 49

4.3. General procedure for the functionalization of 2-alkyl Zincke imines (General procedure 4) 57

4.4. Scale-up protocol **59**

Reaction set-up 59

Reaction protocol 59

4.5. One-pot procedure **60**

4.6 Other functionalizations **61**

General protocol for deprotection of the NTs (according to Rodríguez et al. protocol)[38] 61

Preparation of the amide 38. 61

Preparation of *N*-oxide 39 62
Preparation of the methylpyridinium iodide 40 62
4.7 Meta-meta (amination-bromination) difunctionalisation of Zincke imine 63
"One-pot" protocol 63
4.8 Unsuccessful functionalisation of Zincke imines 64
4.9 *N*-aminopyridinium salts scope 65
5. DFT Calculations 66
5.1. Computational Details 66
5.2. Comparison of free energy profiles for the regioselective addition of the N-centred radical.. 67
5.3. Ring closing mechanism 68
5.4. Alkyl vs Aryl selectivity 69
6. ^{1}H and ^{13}C NMR spectra 70
6.1. Pyridines 70
^{1}H NMR spectrum of pyridine S2a ($CDCl_3$, 298 K) 70
^{1}H NMR spectrum of pyridine S3a ($CDCl_3$, 298 K) 71
^{1}H NMR spectrum of pyridine S4a ($CDCl_3$, 298 K) 71
^{1}H NMR spectrum of pyridine S5a ($CDCl_3$, 298 K) 72
^{1}H NMR spectrum of pyridine S6a ($CDCl_3$, 298 K) 72
^{1}H NMR spectrum of pyridine S7a ($CDCl_3$, 298 K) 73
^{1}H NMR spectrum of pyridine S8a ($CDCl_3$, 298 K) 73
^{1}H NMR spectrum of pyridine S9a ($CDCl_3$, 298 K) 74
^{1}H NMR spectrum of pyridine S10a ($CDCl_3$, 298 K) 74
^{1}H NMR spectrum of pyridine S11a ($CDCl_3$, 298 K) 75
^{1}H NMR spectrum of pyridine S12a ($CDCl_3$, 298 K) 75
^{1}H NMR spectrum of pyridine S13a ($CDCl_3$, 298 K) 76
^{1}H NMR spectrum of pyridine S14a ($CDCl_3$, 298 K) 76
^{1}H NMR spectrum of pyridine S15a ($CDCl_3$, 298 K) 77
^{1}H NMR spectrum of pyridine S16a ($CDCl_3$, 298 K) 77
^{1}H NMR spectrum of pyridine S17a ($CDCl_3$, 298 K) 78
^{1}H NMR spectrum of pyridine S18a ($CDCl_3$, 298 K) 78
^{1}H NMR spectrum of pyridine S19a ($CDCl_3$, 298 K) 79
^{1}H NMR spectrum of pyridine S20a ($CDCl_3$, 298 K) 79
^{1}H NMR spectrum of pyridine S21a ($CDCl_3$, 298 K) 80
^{1}H NMR spectrum of pyridine S22a ($CDCl_3$, 298 K) 80
^{1}H NMR spectrum of pyridine S23a ($CDCl_3$, 298 K) 81
^{1}H NMR spectrum of pyridine S24a ($CDCl_3$, 298 K) 81
^{1}H NMR spectrum of pyridine S25a ($CDCl_3$, 298 K) 82

^{1}H NMR spectrum of pyridine S26a ($CDCl_3$, 298 K)....... 83
^{1}H NMR spectrum of pyridine S27a ($CDCl_3$, 298 K)....... 83
^{1}H NMR spectrum of pyridine S28a ($CDCl_3$, 298 K)....... 84
^{1}H NMR spectrum of pyridine S29a ($CDCl_3$, 298 K)....... 84
^{1}H NMR spectrum of pyridine S30a ($CDCl_3$, 298 K)....... 85
^{1}H NMR spectrum of pyridine S31a ($CDCl_3$, 298 K)....... 85
^{1}H NMR spectrum of pyridine S32a ($CDCl_3$, 298 K)....... 86
^{1}H NMR spectrum of pyridine S33a ($CDCl_3$, 298 K)....... 86
^{1}H NMR spectrum of pyridine S34a ($CDCl_3$, 298 K)....... 87
^{1}H NMR spectrum of pyridine S35a ($CDCl_3$, 298 K)....... 88
6.2. Zincke imines....... 89
^{1}H NMR spectrum of Zincke imine 2 ($CDCl_3$, 298 K)....... 89
^{1}H NMR spectrum of Zincke imine S2b ($CDCl_3$, 298 K) 90
^{1}H NMR spectrum of Zincke imine S3b ($CDCl_3$, 298 K) 91
^{13}C NMR spectrum of Zincke imine S3b ($CDCl_3$, 298 K) 91
^{1}H NMR spectrum of Zincke imine S4b ($CDCl_3$, 298 K) 92
^{13}C NMR spectrum of Zincke imine S4b ($CDCl_3$, 298 K) 92
^{1}H NMR spectrum of Zincke imine S5b ($CDCl_3$, 298 K) 93
^{13}C NMR spectrum of Zincke imine S5b ($CDCl_3$, 298 K) 93
^{1}H NMR spectrum of Zincke imine S6b ($CDCl_3$, 298 K) 94
^{13}C NMR spectrum of Zincke imine S6b ($CDCl_3$, 298 K) 94
^{1}H NMR spectrum of Zincke imine S7b ($CDCl_3$, 298 K) 95
^{13}C NMR spectrum of Zincke imine S7b ($CDCl_3$, 298 K) 95
^{1}H NMR spectrum of Zincke imine S8b ($CDCl_3$, 298 K) 96
^{13}C NMR spectrum of Zincke imine S8b ($CDCl_3$, 298 K) 96
^{1}H NMR spectrum of Zincke imine S9b ($CDCl_3$, 298 K) 97
^{13}C NMR spectrum of Zincke imine S9b ($CDCl_3$, 298 K) 97
^{1}H NMR spectrum of Zincke imine S10b ($CDCl_3$, 298 K) 98
^{13}C NMR spectrum of Zincke imine S10b ($CDCl_3$, 298 K) 98
^{1}H NMR spectrum of Zincke imine S11b ($CDCl_3$, 298 K)....... 99
^{13}C NMR spectrum of Zincke imine S11b ($CDCl_3$, 298 K)....... 99
^{1}H NMR spectrum of Zincke imie S12b ($CDCl_3$, 298 K) 100
^{13}C NMR spectrum of Zincke imine S12b ($CDCl_3$, 298 K) 100
^{1}H NMR spectrum of Zincke imine S13b ($CDCl_3$, 298 K) 101
^{13}C NMR spectrum of Zincke imine S13b ($CDCl_3$, 298 K) 101
^{1}H NMR spectrum of Zincke imine S14b ($CDCl_3$, 298 K) 102
^{13}C NMR spectrum of Zincke imine S14b ($CDCl_3$, 298 K) 102

^{1}H NMR spectrum of Zincke imine S15b ($CDCl_3$, 298 K) 103
^{13}C NMR spectrum of Zincke imine S15b ($CDCl_3$, 298 K) 103
^{1}H NMR spectrum of Zincke imine S16b ($CDCl_3$, 298 K) 104
^{13}C NMR spectrum of Zincke imine S16b ($CDCl_3$, 298 K) 104
^{1}H NMR spectrum of Zincke imine S17b ($CDCl_3$, 298 K) 105
^{13}C NMR spectrum of Zincke imine S17b ($CDCl_3$, 298 K) 105
^{1}H NMR spectrum of Zincke imine S18b ($CDCl_3$, 298 K) 106
^{13}C NMR spectrum of Zincke imine S18b ($CDCl_3$, 298 K) 106
^{1}H NMR spectrum of Zincke imine S19b ($CDCl_3$, 298 K) 107
^{13}C NMR spectrum of Zincke imine S19b ($CDCl_3$, 298 K) 107
^{1}H NMR spectrum of Zincke imine S20b ($CDCl_3$, 298 K) 108
^{13}C NMR spectrum of Zincke imine S20b ($CDCl_3$, 298 K) 108
^{1}H NMR spectrum of Zincke imine S21b ($CDCl_3$, 298 K) 109
^{13}C NMR spectrum of Zincke imine S21b ($CDCl_3$, 298 K) 109
^{1}H NMR spectrum of Zincke imine S22b ($CDCl_3$, 298 K) 110
^{13}C NMR spectrum of Zincke imine S22b ($CDCl_3$, 298 K) 110
^{1}H NMR spectrum of Zincke imine S23b ($CDCl_3$, 298 K) 111
^{13}C NMR spectrum of Zincke imine S23b ($CDCl_3$, 298 K) 111
^{1}H NMR spectrum of Zincke imine S24b ($CDCl_3$, 298 K) 112
^{13}C NMR spectrum of Zincke imine S24b ($CDCl_3$, 298 K) 112
^{1}H NMR spectrum of Zincke imine S25b ($CDCl_3$, 298 K) 113
^{13}C NMR spectrum of Zincke imine S25b ($CDCl_3$, 298 K) 113
^{1}H NMR spectrum of Zincke imine S26b ($CDCl_3$, 298 K) 114
^{13}C NMR spectrum of Zincke imine S26b ($CDCl_3$, 298 K) 114
^{1}H NMR spectrum of Zincke imine S27b ($CDCl_3$, 298 K) 115
^{13}C NMR spectrum of Zincke imine S27b ($CDCl_3$, 298 K) 115
^{1}H NMR spectrum of Zincke imine S28b ($CDCl_3$, 298 K) 116
^{13}C NMR spectrum of Zincke imine S28b ($CDCl_3$, 298 K) 116
^{1}H NMR spectrum of Zincke imine S29b ($CDCl_3$, 298 K) 117
^{13}C NMR spectrum of Zincke imine S29b ($CDCl_3$, 298 K) 117
^{1}H NMR spectrum of Zincke imine S30b ($CDCl_3$, 298 K) 118
^{13}C NMR spectrum of Zincke imine S30b ($CDCl_3$, 298 K) 118
^{1}H NMR spectrum of Zincke imine S31b ($CDCl_3$, 298 K) 119
^{13}C NMR spectrum of Zincke imine S31b ($CDCl_3$, 298 K) 119
^{1}H NMR spectrum of Zincke imine S32b ($CDCl_3$, 298 K) 120
^{13}C NMR spectrum of Zincke imine S32b ($CDCl_3$, 298 K) 120
^{1}H NMR spectrum of Zincke imine S33b ($CDCl_3$, 298 K) 121

^{13}C NMR spectrum of Zincke imine S33b ($CDCl_3$, 298 K) ... 121
^{1}H NMR spectrum of Zincke imine S34b ($CDCl_3$, 298 K) ... 122
^{13}C NMR spectrum of Zincke imine S34b ($CDCl_3$, 298 K) ... 122
^{1}H NMR spectrum of Zincke imine S35b ($CDCl_3$, 298 K) ... 123
^{13}C NMR spectrum of Zincke imine S35b ($CDCl_3$, 298 K) ... 123
^{1}H NMR spectrum of Zincke imine S36b ($CDCl_3$, 298 K) ... 124
^{13}C NMR spectrum of Zincke imine S36b ($CDCl_3$, 298 K) ... 124
^{1}H NMR spectrum of Zincke imine S37b ($CDCl_3$, 298 K) ... 125
^{13}C NMR spectrum of Zincke imine S37b ($CDCl_3$, 298 K) ... 125
^{1}H NMR spectrum of Zincke imine S38b ($CDCl_3$, 298 K) ... 126
^{1}H NMR spectrum of Zincke imine S39b ($CDCl_3$, 298 K) ... 126
^{1}H NMR spectrum of Zincke imine S40b ($CDCl_3$, 298 K) ... 127
^{13}C NMR spectrum of Zincke imine S40b ($CDCl_3$, 298 K) ... 127
^{1}H NMR spectrum of Zincke imine S41b ($CDCl_3$, 298 K) ... 128
^{13}C NMR spectrum of Zincke imine S41b ($CDCl_3$, 298 K) ... 128
^{1}H NMR spectrum of Zincke imine S42b ($CDCl_3$, 298 K) ... 129
^{13}C NMR spectrum of Zincke imine S42b ($CDCl_3$, 298 K) ... 129
^{1}H NMR spectrum of Zincke imine S43b ($CDCl_3$, 298 K) ... 130
^{13}C NMR spectrum of Zincke imine S43b ($CDCl_3$, 298 K) ... 130
^{1}H NMR spectrum of Zincke imine S44b ($CDCl_3$, 298 K) ... 131
^{13}C NMR spectrum of Zincke imine S44b ($CDCl_3$, 298 K) ... 131
^{1}H NMR spectrum of Zincke imine S45b ($CDCl_3$, 298 K) ... 132
^{13}C NMR spectrum of Zincke imine S45b ($CDCl_3$, 298 K) ... 132
^{1}H NMR spectrum of Zincke imine S46b ($CDCl_3$, 298 K) ... 133
^{1}H NMR spectrum of Zincke imine S47b ($CDCl_3$, 298 K) ... 134
^{13}C NMR spectrum of Zincke imine S47b ($CDCl_3$, 298 K) ... 134
^{1}H NMR spectrum of Zincke imine S48b ($CDCl_3$, 298 K) ... 135
^{13}C NMR spectrum of Zincke imine S48b ($CDCl_3$, 298 K) ... 136
^{1}H NMR spectrum of compound S49b ($CDCl_3$, 298 K) ... 136
^{1}H NMR spectrum of compound S50b ($CDCl_3$, 298 K) ... 137
6.3. Functionalised pyridines ... 137
^{1}H NMR spectrum of compound 3a ($CDCl_3$, 298 K) ... 137
^{1}H-^{1}H COSY spectrum of compound 3a ($CDCl_3$, 298 K) ... 138
^{13}C NMR spectrum of compound 3a ($CDCl_3$, 298 K) ... 138
^{1}H NMR spectrum of compound 3b ($CDCl_3$, 298 K) ... 139
^{1}H-^{1}H COSY spectrum of compound 3b ($CDCl_3$, 298 K) ... 140
^{13}C NMR spectrum of compound 3b ($CDCl_3$, 298 K) ... 140

^{1}H NMR spectrum of compound 3c ($CDCl_3$, 298 K) 141
^{1}H-^{1}H COSY spectrum of compound 3c ($CDCl_3$, 298 K) 141
^{13}C NMR spectrum of compound 3c ($CDCl_3$, 298 K) 142
^{1}H NMR spectrum of compound 2a ($CDCl_3$, 298 K) 142
^{13}C NMR spectrum of compound 2a ($CDCl_3$, 298 K) 143
^{1}H-^{1}H COSY spectrum of compound 2a ($CDCl_3$, 298 K) 143
^{1}H NMR spectrum of compound 4a ($CDCl_3$, 298 K) 144
^{13}C NMR spectrum of compound 4a ($CDCl_3$, 298 K) 144
^{1}H NMR spectrum of compound 4b ($CDCl_3$, 298 K) 145
^{13}C NMR spectrum of compound 4b ($CDCl_3$, 298 K) 145
^{1}H NMR spectrum of compound 5a ($CDCl_3$, 298 K) 146
^{13}C NMR spectrum of compound 5a ($CDCl_3$, 298 K) 146
^{1}H NMR spectrum of compound 5b ($CDCl_3$, 298 K) 147
^{13}C NMR spectrum of compound 5b ($CDCl_3$, 298 K) 147
^{1}H NMR spectrum of compound 6a ($CDCl_3$, 298 K) 148
^{13}C NMR spectrum of compound 6a ($CDCl_3$, 298 K) 148
^{1}H NMR spectrum of compound 6b ($CDCl_3$, 298 K) 149
^{13}C NMR spectrum of compound 6b ($CDCl_3$, 298 K) 149
^{1}H NMR spectrum of compound 7a ($CDCl_3$, 298 K) 150
^{13}C NMR spectrum of compound 7a ($CDCl_3$, 298 K) 150
^{1}H NMR spectrum of compound 8a ($CDCl_3$, 298 K) 151
^{13}C NMR spectrum of compound 8a ($CDCl_3$, 298 K) 151
^{1}H NMR spectrum of compound 9a ($CDCl_3$, 298 K) 152
^{13}C NMR spectrum of compound 9a ($CDCl_3$, 298 K) 152
^{1}H NMR spectrum of compound 9b ($CDCl_3$, 298 K) 153
^{13}C NMR spectrum of compound 9b ($CDCl_3$, 298 K) 153
^{1}H NMR spectrum of compound 10a ($CDCl_3$, 298 K) 154
^{13}C NMR spectrum of compound 10a ($CDCl_3$, 298 K) 154
^{1}H NMR spectrum of compound 11a ($CDCl_3$, 298 K) 155
^{13}C NMR spectrum of compound 11a ($CDCl_3$, 298 K) 155
^{1}H NMR spectrum of compound 12a ($CDCl_3$, 298 K) 156
^{13}C NMR spectrum of compound 12a ($CDCl_3$, 298 K) 156
^{1}H NMR spectrum of compound 12b ($CDCl_3$, 298 K) 157
^{13}C NMR spectrum of compound 12b ($CDCl_3$, 298 K) 157
^{1}H NMR spectrum of compound 13a ($CDCl_3$, 298 K) 158
^{13}C NMR spectrum of compound 13a ($CDCl_3$, 298 K) 158
^{1}H NMR spectrum of compound 14a ($CDCl_3$, 298 K) 159

^{13}C NMR spectrum of compound 14a ($CDCl_3$, 298 K) 159
^{1}H NMR spectrum of compound 14b ($CDCl_3$, 298 K) 160
^{13}C NMR spectrum of compound 14b ($CDCl_3$, 298 K) 160
^{1}H NMR spectrum of compound 15a ($CDCl_3$, 298 K) 161
^{13}C NMR spectrum of compound 15a ($CDCl_3$, 298 K) 161
^{1}H NMR spectrum of compound 15b ($CDCl_3$, 298 K) 162
^{13}C NMR spectrum of compound 15b ($CDCl_3$, 298 K) 162
^{1}H NMR spectrum of compound 16a ($CDCl_3$, 298 K) 163
^{13}C NMR spectrum of compound 16a ($CDCl_3$, 298 K) 163
^{1}H NMR spectrum of compound 17a ($CDCl_3$, 298 K) 164
^{13}C NMR spectrum of compound 17a ($CDCl_3$, 298 K) 164
^{13}C NMR spectrum of compound 18a ($CDCl_3$, 298 K) 165
^{1}H NMR spectrum of compound 19a ($CDCl_3$, 298 K) 166
^{13}C NMR spectrum of compound 19a ($CDCl_3$, 298 K) 166
^{1}H NMR spectrum of compound 20a ($CDCl_3$, 298 K) 167
^{13}C NMR spectrum of compound 20a ($CDCl_3$, 298 K) 167
^{1}H NMR spectrum of compound 21a ($CDCl_3$, 298 K) 168
^{13}C NMR spectrum of compound 21a ($CDCl_3$, 298 K) 168
^{1}H NMR spectrum of compound 22a ($CDCl_3$, 298 K) 169
^{13}C NMR spectrum of compound 22a ($CDCl_3$, 298 K) 169
^{1}H NMR spectrum of compound 23a ($CDCl_3$, 298 K) 170
^{13}C NMR spectrum of compound 23a ($CDCl_3$, 298 K) 170
^{1}H NMR spectrum of compound 24a ($CDCl_3$, 298 K) 171
^{13}C NMR spectrum of compound 24a ($CDCl_3$, 298 K) 171
^{1}H NMR spectrum of compound 24b ($CDCl_3$, 298 K) 172
^{13}C NMR spectrum of compound 24b ($CDCl_3$, 298 K) 172
^{1}H NMR spectrum of compound 25a ($CDCl_3$, 298 K) 173
^{13}C NMR spectrum of compound 25a ($CDCl_3$, 298 K) 173
^{1}H NMR spectrum of compound 26a ($CDCl_3$, 298 K) 174
^{13}C NMR spectrum of compound 26a ($CDCl_3$, 298 K) 174
^{1}H NMR spectrum of compound 27a ($CDCl_3$, 298 K) 175
^{13}C NMR spectrum of compound 27a ($CDCl_3$, 298 K) 175
^{1}H NMR spectrum of compound 28a ($CDCl_3$, 298 K) 176
^{13}C NMR spectrum of compound 28a ($CDCl_3$, 298 K) 176
^{1}H NMR spectrum of compound 29a ($CDCl_3$, 298 K) 177
^{13}C NMR spectrum of compound 29a ($CDCl_3$, 298 K) 177
^{1}H NMR spectrum of compound 30b ($CDCl_3$, 298 K) 178

^{13}C NMR spectrum of compound 30b ($CDCl_3$, 298 K) ... 178
^{1}H NMR spectrum of compound 31b ($CDCl_3$, 298 K) ... 179
^{13}C NMR spectrum of compound 31b ($CDCl_3$, 298 K) ... 179
^{1}H NMR spectrum of compound 32b ($CDCl_3$, 298 K) ... 180
^{13}C NMR spectrum of compound 32b ($CDCl_3$, 298 K) ... 180
^{1}H NMR spectrum of compound 33a ($CDCl_3$, 298 K) ... 181
^{13}C NMR spectrum of compound 33a ($CDCl_3$, 298 K) ... 181
^{1}H NMR spectrum of compound 33b ($CDCl_3$, 298 K) ... 182
^{13}C NMR spectrum of compound 33b ($CDCl_3$, 298 K) ... 182
^{1}H NMR spectrum of compound 34b ($CDCl_3$, 298 K) ... 183
^{13}C NMR spectrum of compound 34b ($CDCl_3$, 298 K) ... 183
^{1}H NMR spectrum of compound 35a ($CDCl_3$, 298 K) ... 184
^{13}C NMR spectrum of compound 35a ($CDCl_3$, 298 K) ... 184
^{1}H NMR spectrum of compound 35b ($CDCl_3$, 298 K) ... 185
^{13}C NMR spectrum of compound 35b ($CDCl_3$, 298 K) ... 185
^{1}H NMR spectrum of compound 35b (CD_3OD, 298 K) ... 186
^{13}C NMR spectrum of compound 35b (CD_3OD, 298 K) ... 186
^{1}H NMR spectrum of compound 35b (DMSO-d_6, 298 K) ... 187
^{1}H NMR spectrum of compound 35b (DMSO-d_6, 353 K) ... 187
^{13}C NMR spectrum of compound 35b (DMSO-d_6, 353 K) ... 188
LR-MS chromatogram of compound 35b ... 189
GC-FID chromatogram of compound 35b ... 189
^{1}H NMR spectrum of salt Py-salt (DMSO-d_6, 298 K) ... 190
^{1}H NMR spectrum of compound 36 ($CDCl_3$, 298 K) ... 191
^{13}C NMR spectrum of compound 36 ($CDCl_3$, 298 K) ... 191
^{1}H NMR spectrum of compound 37 ($CDCl_3$, 298 K) ... 192
^{13}C NMR spectrum of compound 37 ($CDCl_3$, 298 K) ... 192
^{1}H NMR spectrum of compound 38 ($CDCl_3$, 298 K) ... 193
^{13}C NMR spectrum of compound 38 ($CDCl_3$, 298 K) ... 193
^{19}F NMR spectrum of compound 38 ($CDCl_3$, 298 K) ... 194
^{1}H NMR spectrum of compound 39 ($CDCl_3$, 298 K) ... 195
^{13}C NMR spectrum of compound 39 ($CDCl_3$, 298 K) ... 195
^{1}H NMR spectrum of compound 40 (DMSO-d_6, 298 K) ... 196
^{13}C NMR spectrum of compound 40 (DMSO-d_6, 298 K) ... 196
^{1}H NMR spectrum of compound 41a ($CDCl_3$, 298 K) ... 197
^{13}C NMR spectrum of compound 41a ($CDCl_3$, 298 K) ... 197
^{1}H NMR spectrum of compound 41b ($CDCl_3$, 298 K) ... 198

^{13}C NMR spectrum of compound 41b ($CDCl_3$, 298 K) 198

7. Crystallographic data for intermediate 2a 199

8. Supplementary References 201

1. General information

All solvents and commercially available reagents were purchased as reagent grade and used without further purification, unless otherwise noted. Reactions were monitored by thin layer chromatography (TLC), using 0.20 mm Merck silica plates (60F-254) and visualized using UV-light or potassium permanganate stain with heat as a developing agent. GC yields were calibrated with dodecane as the internal standard.

NMR spectra were recorded on Bruker 400 MHz or Varian 600 MHz and calibrated using solvent residual peaks ($CHCl_3$ – 7.26 ppm ^{1}H NMR, 77.16 ppm ^{13}C NMR, D2O - 4.64 ppm ^{1}H NMR) or TMS as an internal reference. Chemical shifts are reported relatively in δ-scale as parts per million (ppm) referenced to the residual solvent peak. Coupling constants *J* are given in Hertz (Hz), and the following abbreviations were used to indicate signal multiplicity: ^{1}H NMR: s = singlet, d = doublet, t = triplet, q = quartet, m = multiplet, and the respective combinations.

Low-resolution mass spectra (LRMS) were recorded on an Applied Biosystems API 365 **mass spectrometer using an electrospray ionization (ESI)** technique. High resolution mass spectra (HRMS) were recorded on a Waters AutoSpec Premier instrument using electron ionization (EI) or a Waters SYNAPT G2-S HRMS instrument using electrospray ionization (ESI) or atmospheric-pressure chemical ionization (APCI) with a time-of-flight detector (TOF).

GC-FID analyses were performed using Shimadzu GCMS-QP2010 SE with helium as carrier gas chromatograph with FID detector and Zebron ZB 5MSi column (length: 30.0 m; thickness: 0.25 um, diameter: 0.25 mm).

GC programme: time: 19.39 min; pressure: 121.8 kPa; total flow: 30.3 mL/min; column flow: 1.30 mL/min; linear velocity: 33.1 cm/s; purge flow: 3.0 mL/min; split ratio: 20.0.

Flash column chromatography was performed on the CombiFlash NextGen 300 flash chromatography system. Colum chromatography was performed using Merck silica gel 60 (230-400 mesh).

Preparative HPLC separations were performed using a Knauer HPLC chromatograph with PDA detector and a Preparative column chromatography Knauer EII 100-10 Si column (250 x 20 mm), flow rate: 15 ml / min. Model reaction

1.1. Reaction set-up

Experiments (optimisation studies and scope) were carried out in the UOSlab Miniphoto photoreactor (**Supplementary Figure 1**). Violet light irradiation (emission maximum at 405 nm) was applied to each reaction vial with the use of 7 LUMINUS LED units (of overall 24 W intensity when 100% power applied). The ambient temperature of the LED block was maintained by cooling with Huber MiniChiller 600 ($T_{reaction}$ ~0-5 °C).

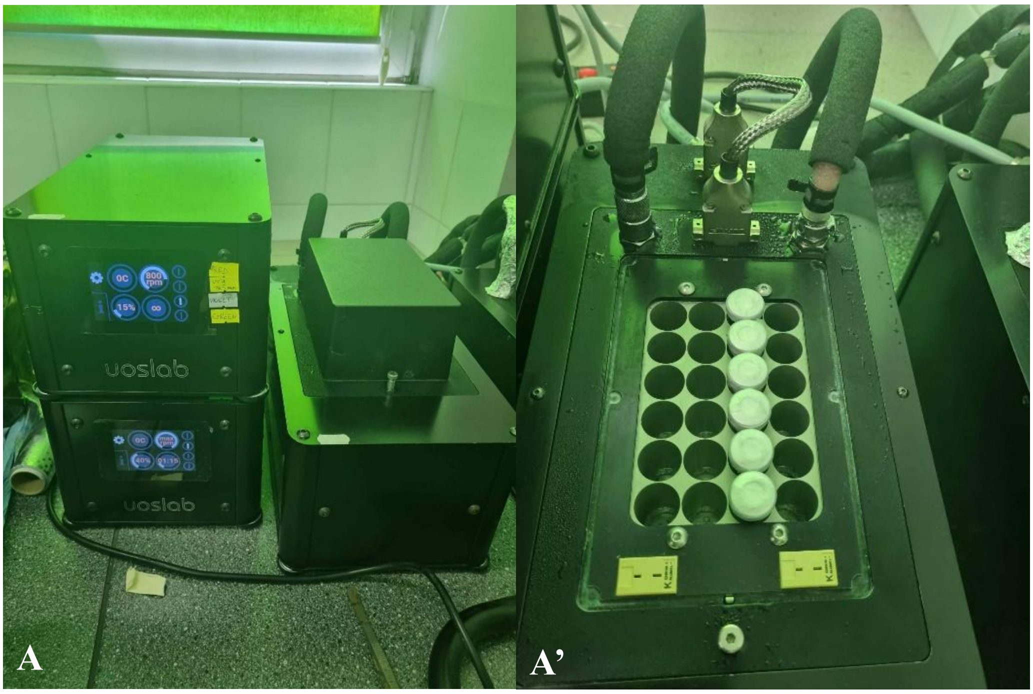

Supplementary Figure 1. *Photochemical reaction set-up: UOSlab Miniphoto photoreactor (A and A').*

1.2. Preparation of the model reaction system

Zincke imine as a starting material for the optimisation studies was prepared according to the modified McNally's protocol.[1]

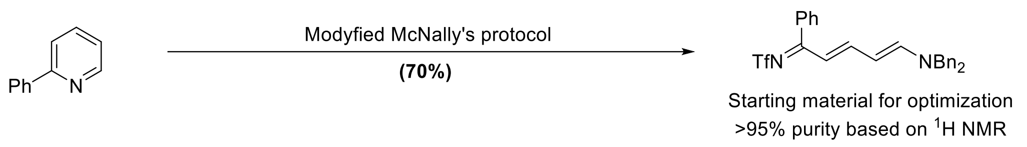

Supplementary Figure 2. *General scheme for the preparation of the starting material for optimisation studies.*

Zincke imines are rather stable compounds (if they are stored in pure form). In particular, 2-aryl Zincke imines can be stored in the presence of air and moisture at room temperature for a couple of days without significant decomposition.

Pyridinium salt (**Py-salt**) was prepared according to the Goliszewska's protocol.[2]

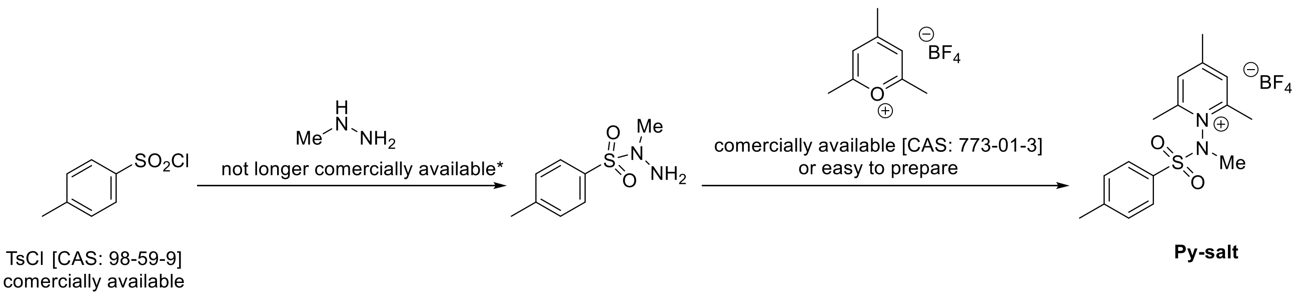

Supplementary Figure 3. *General scheme for the preparation of pyridinium salt* ***Py-salt****. (for details, see page 44); *could be replaced with methylhydrazine sulphate [CAS: 302-15-8] which is readily available and not expensive.*

The amount of 2-phenylpyridine in the reaction mixtures was monitored by the GC-FID calibration curve with dodecane as internal standard. The curve was validated using 5.4 mg of 2-phenylpyridine (**1**) in 10 ml of AcOEt solution. The average error of 3 measurements was lower than 2% (**Supplementary Figure 4**).

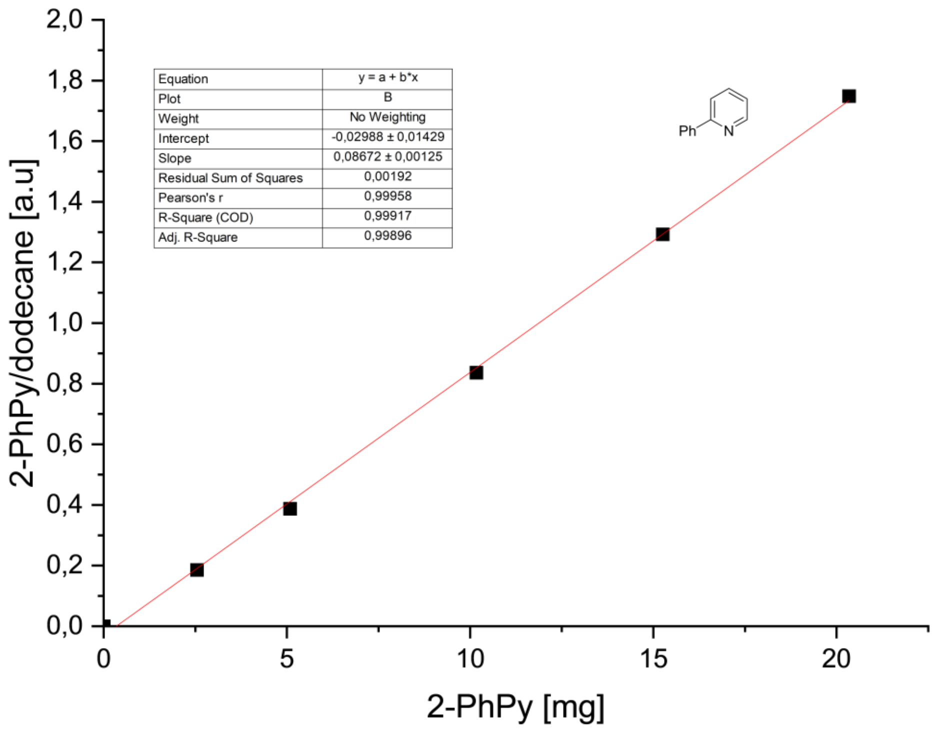

Supplementary Figure 4. GC-FID calibration curve for 2-phenylpyridine (**1**) with dodecane as internal standard.

Zincke imines were cyclized by heating with an excess of NH_4OAc. For simplification, saturated NH_4OAc solution was prepared in anhydrous ethanol (~24% w/w). The cyclization process is quantitative on GC-FID analysis of the reaction mixture.

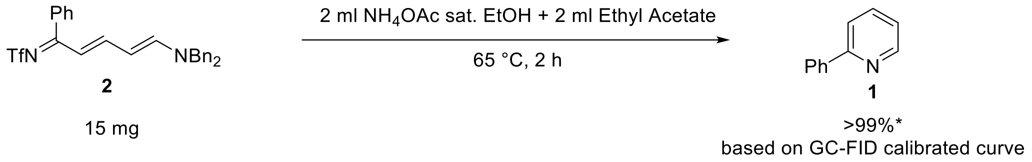

Supplementary Figure 5. *Recyclization process with the saturated* NH_4OAc *in the ethanol solution.*

*The reaction could be also performed in the DMSO/MeCN/EtOH or MeCN/EtOH mixture *In a DCM/EtOH mixture the reaction mixture was heated up to 45 °C typically for 4 h.

Procedure: Zincke imine **2** (0.031 mmol, 15 mg) was dissolved in AcOEt (2 ml) and a saturated solution of NH_4OAc in anhydrous EtOH (2 ml) was added. The reaction mixture was heated to 65 °C for 2 h. After the indicated time, dodecane as an internal standard was added (15 µl) and the mixture was diluted to 25 ml with AcOEt. The crude reaction mixture was then analysed with the GC-FID calibrated method indicating quantitative conversion of **2** to **1**.

1.3. Model reaction

The model reaction involves Zincke imine (**2**) prepared from 2-phenylpyridine (**1**) and *N*-methylpyridinium salt (**Py-salt**). Intermediate **2a** was not isolated in that process.

A general protocol for optimisation studies:

Zincke imine **2** (24 mg, 0.05 mmol), pyridinium salt **Py-salt** (x mmol), **cat.** (x mg, x μmol, <5 mol%) were placed in the closed cup vial and a solvent (x ml) and a co-solvent [if required] (x ml) were added through the septum. The reaction mixture was placed in ultrasound bath and degassed by bubbling argon through the solution for 15 min. The vial was then transferred to the photoreactor and irradiated with LED [wavelength] light (x W) for x h maintaining a temperature between 0 °C to 5 °C with the dedicated cooling system. The solution was then diluted with AcOEt (~15 ml) and dodecane was added as an internal standard (15 μl). The sample (2 ml) was filtered through cotton and subjected to GC-FID measurements.

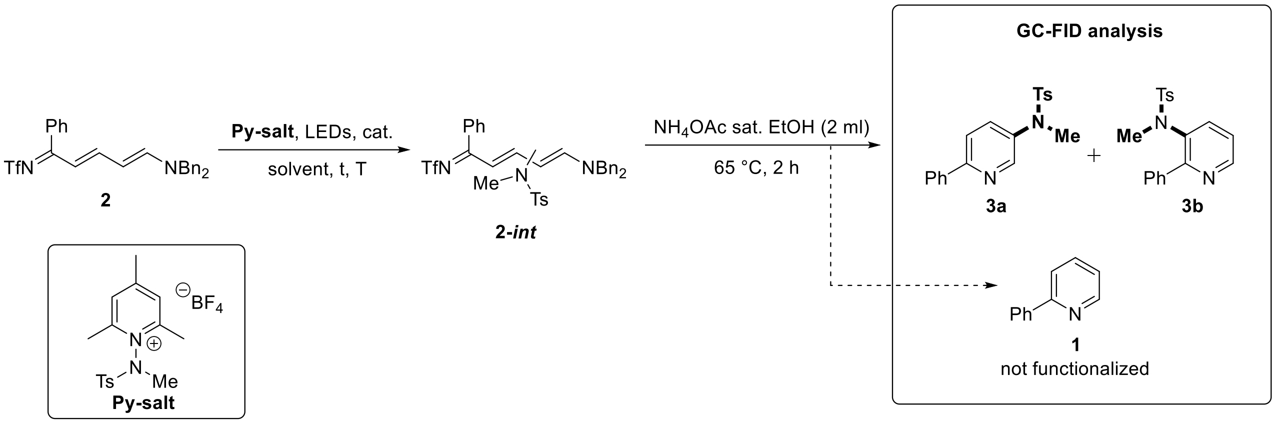

Supplementary Figure 6. *Model reaction system.*

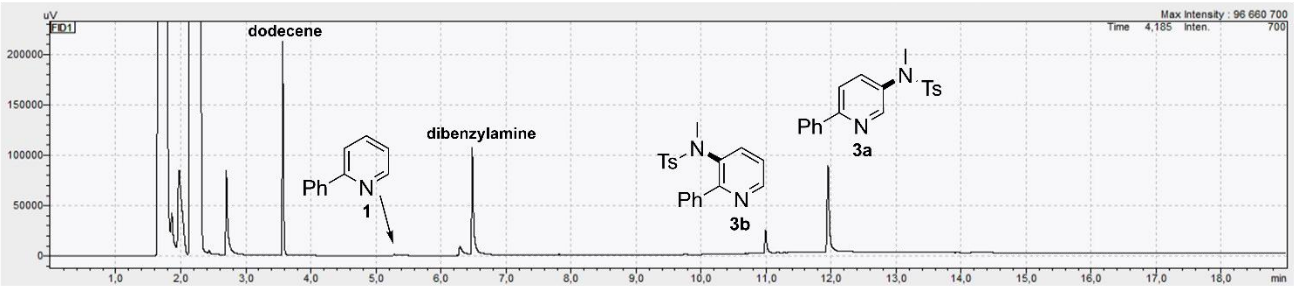

Supplementary Figure 7. *Typical GC-FID chromatogram of the crude reaction mixture (under optimised condition, >99% yield and >99 % conversion of the starting material)*

Based on the analytically pure sample of isolated product **3a** (50 mg), the GC-FID calibration curve with dodecane as an internal standard was prepared. The method curve was validated using **3a** (5.4 mg) in AcOEt (10 ml). The average error of 3 measurements was lower than 3%. Yields were directly calculated from the calibrated and validated curve (in optimal conditions confirmed also with ^{1}H NMR with CH_2Br_2 as internal standard).

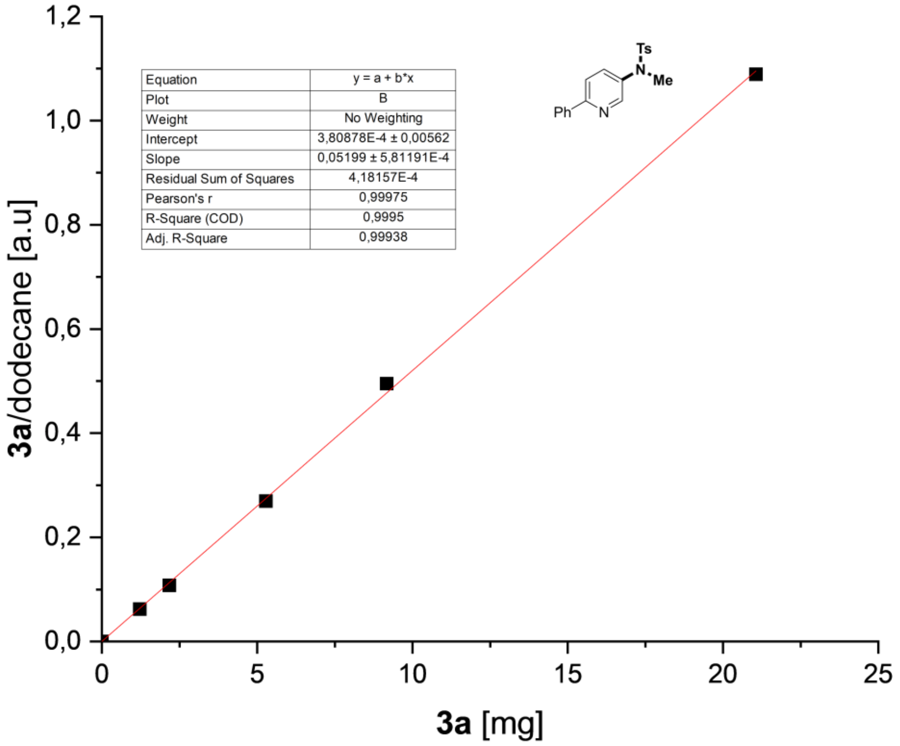

Supplementary Figure 8. *GC-FID calibration curve for product **3a** with dodecane as internal standard.*

1.4. Preliminary results

1.4.1 Photofunctionalization of the Zincke imine

Preliminary studies were initiated by testing our hypothesis that Zincke imine can undergo photofunctionalization with *N*-aminopyridinium salts. As a first, we have tested typical solvents and light sources for the pyridinium salt activation along with *fac*-Ir(ppy)₃, which has been already proven to act as effective photoredox catalyst compatible with different pyridinium salts.[2,3] Photochemical reactions were typically set up using a Zincke imine **2** 50 mg while maintaining the temperature between 0 and 5 °C with a dedicated cooling system.

Supplementary Table 1. Initial LED wavelength and solvent screening.

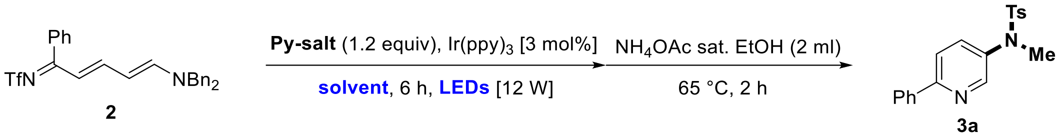

entry	solvent	light source	yield 3a [%][a]
1	MeCN	UV (365 nm)	<5%
2	MeCN	violet (405 nm)	20%
3	MeCN	blue (465 nm)	<5%
4	DCM	UV (365 nm)	<5%
5	DCM	violet (405 nm)	<5%
6	DCM	blue (465 nm)	<5%

[a]*isolated yield*

1.4.2 Control experiments

For the determination of photochemical nature of the reaction, control experiments were performed (no light, no catalyst etc.) and we also checked stability of starting material when exposed to catalyst or light.

Supplementary Table 2. Control experiments.

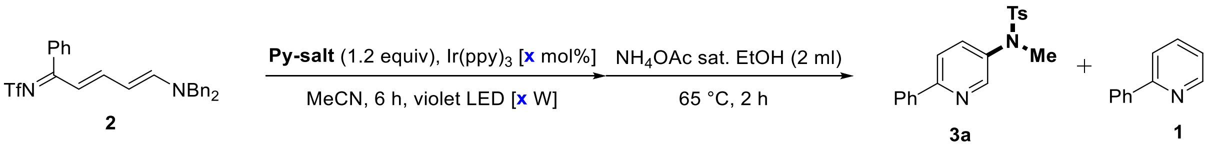

entry	modification	light source	yield 3a [%][a]	yield 1 [%][a]
1	none	violet (405 nm)	20	70
2	no light	no light	<1	87
3	no catalyst	violet (405 nm)	<5	76
4	no catalyst, no light	no light	0	>99

[a]*GC-FID yield*

Exposure of Zincke imine to light itself or the catalyst is responsible for unwanted reactivity (**entry 2** and **3**) only a combination of both the catalyst and light leads to the desired product (**entry 1**).

1.4.3. Initial conditions

Looking for a starting point for optimisation studies, several conditions were tested.

Supplementary Table 3. Initial conditions for the reaction set-up.

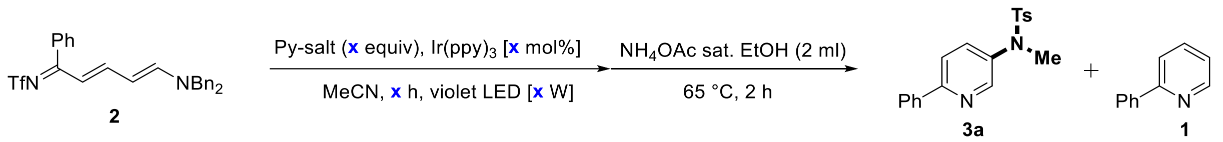

entry	concentration [mmol/ml]	irradiation power [W]	catalyst loading [mol%]	Py-salt [equiv.]	irradiation time [h]	yield 3a [%][a]	yield 1 [%][a]
1	0.05	24	1	2	8	41	14
2	0.025	12	3	3	12	36	20
3	0.0125	18	3	1.3	6	27	67
4	0.05	24	1	2	8	41	14
**5**	**0.05**	**24**	**3**	**2**	**8**	**46**	**9**
6	0.05	24	5	2	8	45	9
**7**	**0.05**	**24**	**3**	**2**	**2**	**43**	**17**
8	0.05	24	3	2	24	36	5

[a]GC-FID yield

The conditions presented in entry 7 were chosen as a promising starting point for our optimisation studies (*reaction time is the shortest and the yield is comparable to entry* 5).

1.4.3. Optimisation studies

Catalyst loading

Systematic studies were focused on optimisation of parameters such as the amount of catalyst and pyridinium salt **Py-salt**, the irradiation power, and the reaction time.

Supplementary Table 4. Optimisation of the catalyst loading.

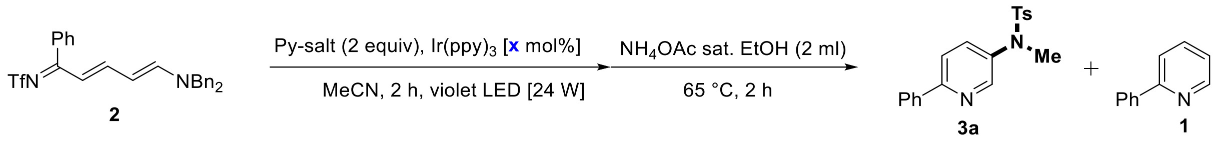

entry	catalyst loading [mol%]	yield 3a [%][a]	yield 1 [%][a]
1	0	5	66
2	0.2	22	44
3	0.5	35	27
**4**	**1.5**	**44**	**17**
5	2.5	44	14
8	4.5	40	13

[a]GC-FID yield

The conditions presented in **entry 4** were chosen because of the lowest catalyst loading giving satisfactory results.

Amount of pyridinium salt and its concentration

A detailed analysis of the reaction mixture showed that apart from pyridine **3a**, second regioisomer **3b** was also formed. Additional optimisation including concentration and an amount of the salt allows to increase efficacy outcome of the reaction.

Supplementary Table 5. Optimisation of the amount of the pyridinium salt and concentration.

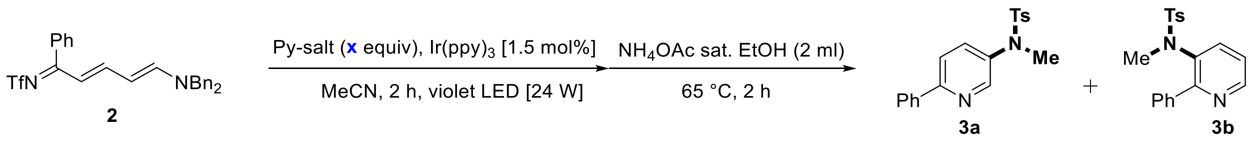

entry	Py-salt [equiv.]	concentration [mmol/ml]	yield 3a [%][a]	3a : 3b ratio	yield 1 [%][a]	total yield 3a & 3b [%][a]
0	2.0	0.05	44	2.1 : 1	17	65
1	1.1	0.05	38	2.1 : 1	35	55
2	1.5	0.05	39	2.0 : 1	22	59
3	5.0	0.05	31	2.6 : 1	10	43
4	2.0	0.1	34	nd	28	nd
**5**	**2.0**	**0.0125**	**48**	**2.6 : 1**	**8**	**66**

[a]GC-FID yield

Equivalents of pyridinium salt and concentration (under lower irradiation power [2.4 W])

Subsequent modifications, including decreasing the irradiation power (from 24 W to 2.4 W) improved selectivity and vastly eliminated side products (yield of **1** + **3a** + **3b** ~100%, **entry 5**).

Supplementary Table 6. Optimisation of the amount of the pyridinium salt and concentration under lower irradiation power [2.4W]

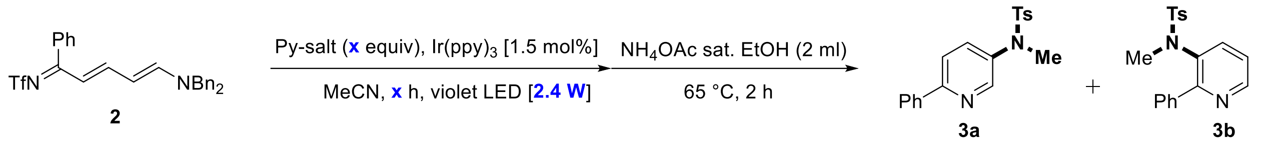

entry	Py-salt [equiv.]	concentration [mmol/ml]	t [h]	3a : 3b ratio	total yield 3a & 3b [%][a]	yield 1 [%][a]
1	1.3	0.025	12	4.0 : 1	21	59
2	1.3	0.025	2	2.0 : 1	6	80
3	1.5	0.025	12	3.6 : 1	29	51
4	2.0	0.025	12	3.6 : 1	36	40
**5**	**2.0**	**0.0125**	**12**	**3.1 : 1**	**66**	**33**

[a]GC-FID yield

The addition of 3 equivalents of salts gave better selectivity (**3a**:**3b** 4.8:1) (due to the faster consumption of isomer **3b** to form a disubstituted derivative (characterized by ^{1}H NMR, ^{13}C NMR, ^{1}H-^{1}H COSY and HRMS; for details, see pages 164-165).

Reaction time

Decreasing the concentration of along with the elongation of the irradiation time increased the yield (**entry 2**).

Supplementary Table 7. Fine tuning of the concentration of the reaction mixture.

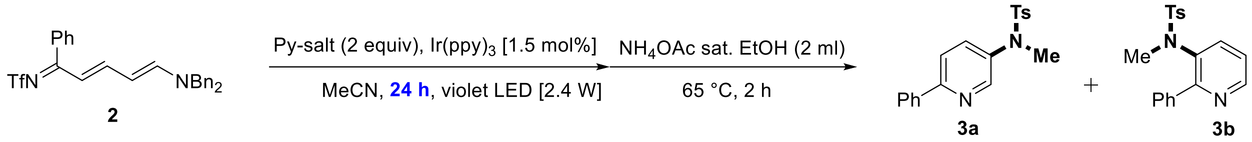

entry	concentration [mmol/ml]	3a : 3b ratio	total yield 3a & 3b [%][a]	yield 1 [%][a]
1	0.0125	3.1 : 1	76	17
**2**	**0.00625**	**3.1 : 0**	**83**	**13**

[a]GC-FID yield

Fine-tuning of the solvent (addition of the second solvent)

The addition of a co-solvent led to a further increase in the yield and regioselectivity of the reaction.

Supplementary Table 8. Fine tuning of the solvent.

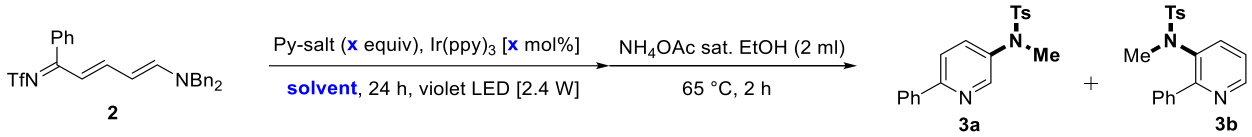

entry	Py-salt [equiv.]	solvent	t [h]	3a : 3b ratio	total yield 3a & 3b [%]	yield 1 [%][a]
1	2.0	MeCN/DMSO (v/v 1:1)	24	6.3 : 1	88	<5
2	1.8	MeCN/DMSO (v/v 1:1)	24	5.2 : 1	98	<5
**3**	**1.5**	MeCN/DMSO (v/v 1:1)	**24**	**5.5 : 1**	**98**	<5
**4***	**1.5**	MeCN/DMSO (v/v 1:1)	**24**	**5.5 : 1**	**98**	<5
**5***	**1.5**	AcOEt/DMSO (v/v 1:1)	**24**	**7.3 : 1**	**88**	<5
**6***	**1.5**	DCM/DMSO (v/v 1:1)	**24**	**5.6 : 1**	**87**	<5

[a]GC-FID yield, * 2 mol% of Ir(ppy)$_3$

*Reactions were set-up on a 25 mg scale of **2** (~6.25 mM).*

Increasing the amount of the catalyst to 2 mol% did not affect the yield, though it helped with the reproducibility of the reaction.

Reproducibility and robustness

To confirm reproducibility and robustness of the developed method the model reaction was set up independently by three authors.

Supplementary Table 9. Reproducibility and robustness of the optimised reaction.

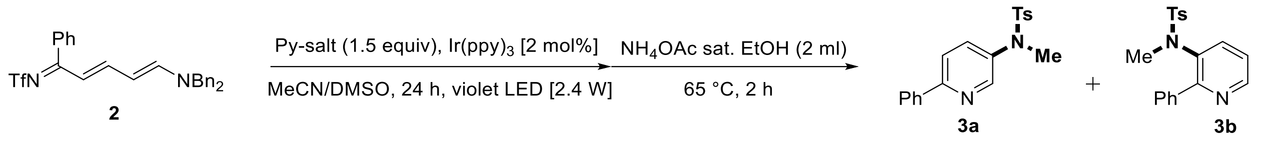

entry	3a : 3b ratio	total yield 3a & 3b [%][a]
1*	4.8 : 1	>99
2**	5.0 : 1	>99
3***	4.8 : 1	>99

*Piotr Banachowicz
**Kitti Szabó
***Antoni Powala
[a]GC-FID yield

*In addition, the reaction mixture from **entry 1** was worked out with the standard protocol (for details, see page **42**) leading to 95% isolated yield. The reaction mixture from **entry 2** was worked up only with the extraction step, and then ^{1}H NMR with CH_2Br_2 as an internal standard was measured gives >95% yield and ~5:1 regioisomers mixture.*

Other Zincke derivatives

Supplementary Table 10. Testing other Zincke Imines derivatives.

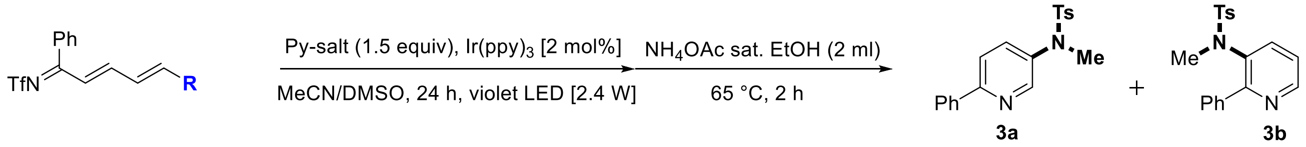

entry	Symbol of Zincke imine	R	3a : 3b ratio	total yield 3a & 3b [%][a]
1	**2**	NBn_2	4.8 : 1	>99
2	**S49b**	morpholine	NA	nd
3	**S50b**	NPhBn	26	1.9:1

NA – not assigned; nd – not detected

2. Mechanistic investigations

2.1. UV-VIS spectroscopy of the starting material

In general, Zincke imines are colourful derivatives that range from yellow to red and absorb light in the blue region. To explain why blue light irradiation does not give satisfactory results for the model reaction of Zincke imine **2** with Py-salt, UV-Vis spectra of both the photocatalyst and the Zincke imine were measured.

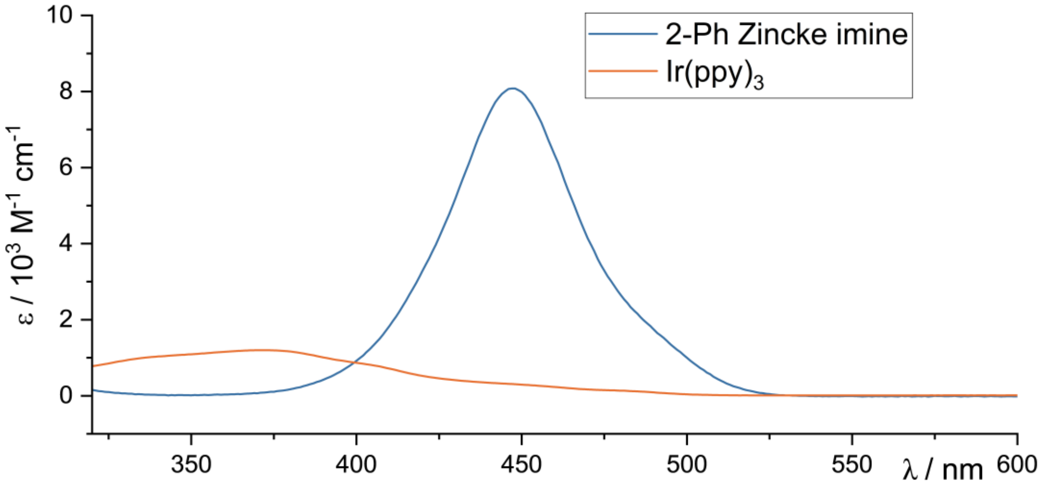

Supplementary Figure 9. *UV-VIS spectrum of 2-phenyl Zincke Imine (**2**) [solution in MeCN] and Ir(ppy)$_3$ [solution in MeCN].*

<u>*Conclusion:*</u> UV-VIS data clearly show that in the blue region Zincke imine **2** is a much stronger absorber than the catalyst. As a result, the Zincke imine is excited instead of the catalyst, thus, violet light (405 nm) was used to initiate the model reaction.

2.2. Kinetic studies

For kinetic studies, the model reaction under optimal conditions was set-up in one 10 ml closed-cup vial and in an indicated period, 100 µl aliquots were taken and treated with the saturated solution of NH_4OAc to convert intermediates **2** to pyridines (**3a**, **3b,** and **1**). After heating for the indicated time, the reaction mixture was diluted, and dodecane was added as an internal standard. The crude reaction mixtures were analysed using the calibrated GC-FID method.

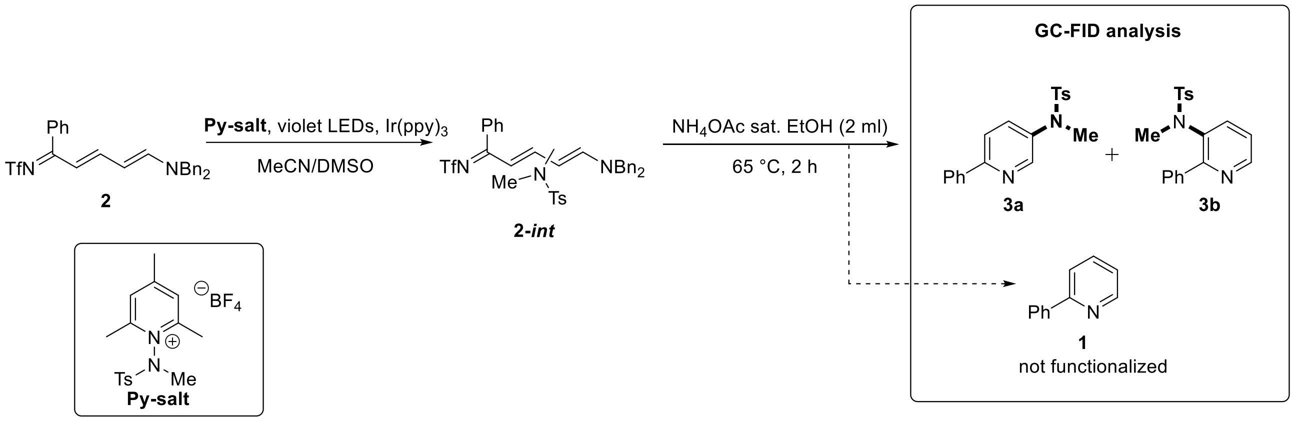

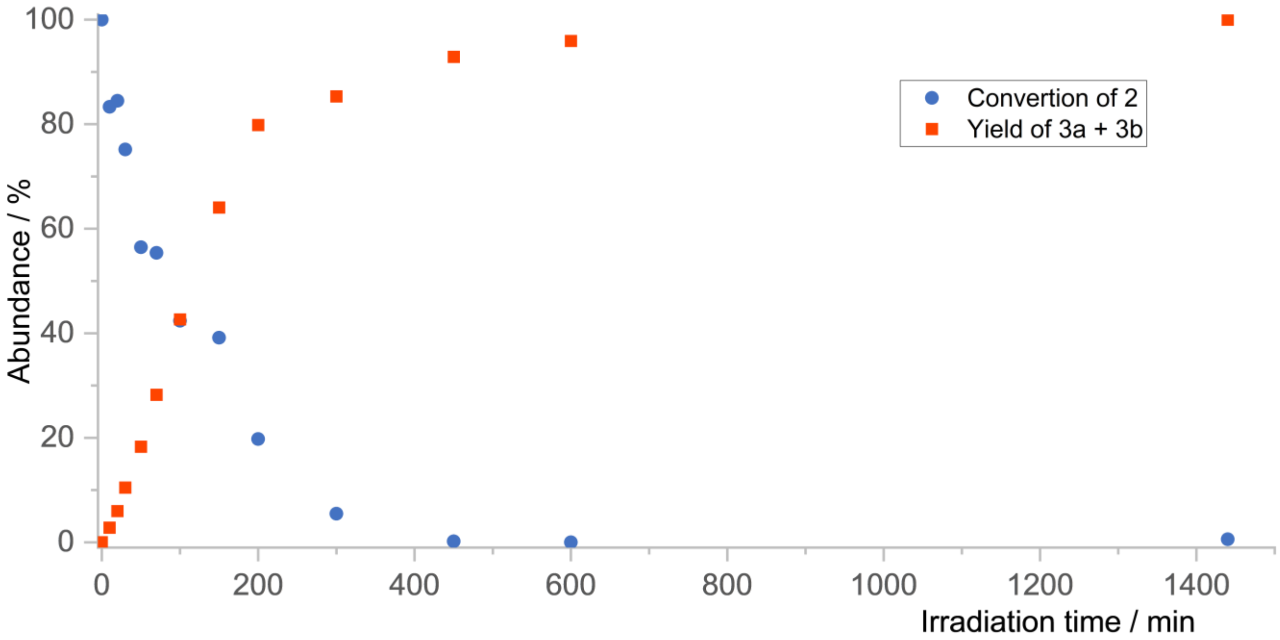

Supplementary Figure 10. *Total yield (**3a** + **3b**) and conversion of starting material **2**.*

The reaction profile indicates that the reaction is almost finished after 12 h (conversion of **2** >95%). For the scope and limitation studies reaction time were always extended to 24 h to assure full conversion of starting materials for a larger group of substrates.

2.3. Regioselectivity of the model reaction

According to the proposed reaction mechanism, two possible regioisomers can be formed. Based on a detailed analysis of ^{1}H, ^{1}H-^{1}H COSY spectra (for details, see pages 160), and the X-ray structure of main functionalized Zincke imine **2a** (**Supplementary Figure 12**), the regioselectivity of the photoamidation reaction was demonstrated univocally. In addition, the strongest shielded proton (which could be assigned as *CH* in 2 position of the pyridine moiety) has a very characteristic resonance and, in most cases, characteristic multiplicity.

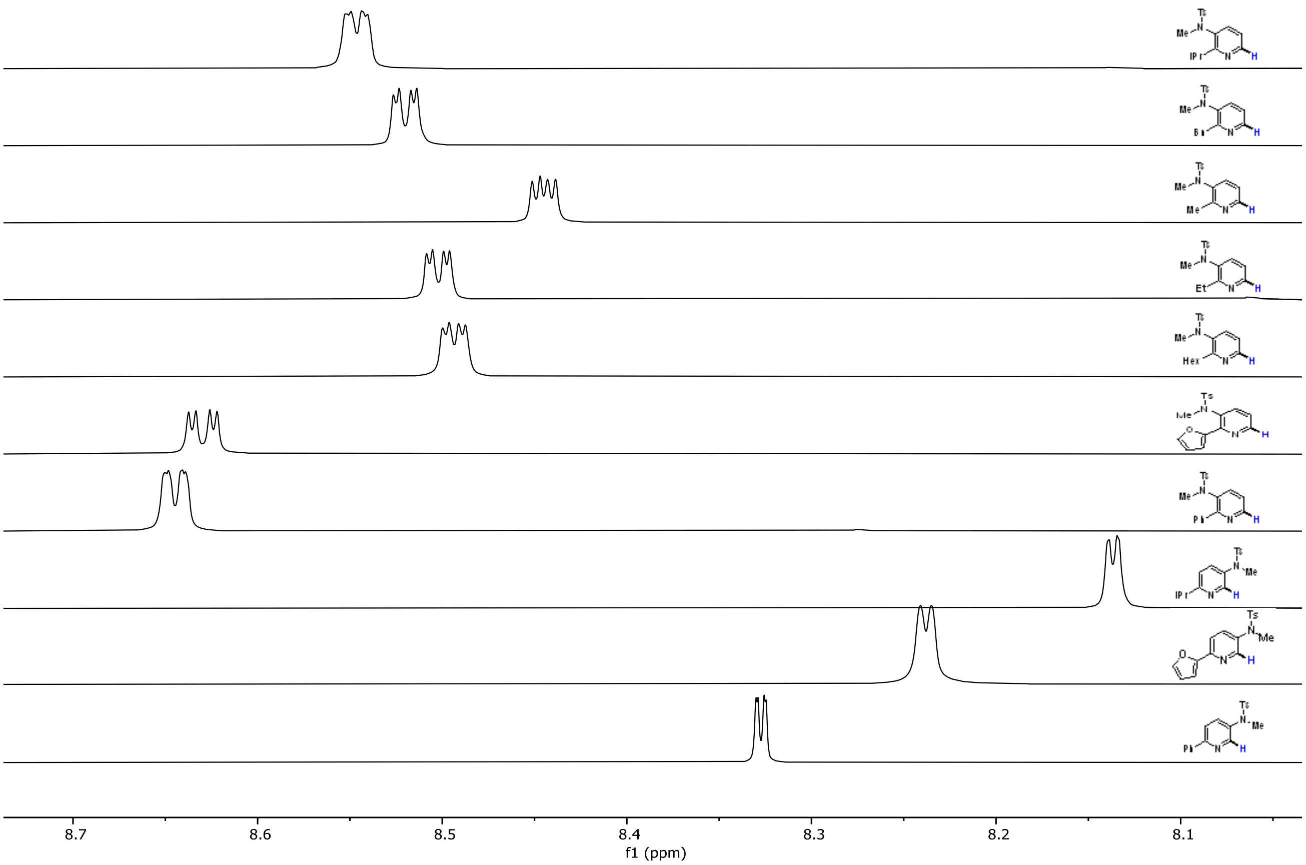

Supplementary Figure 11. *Examples of ^{1}H NMR spectra of products with assigned structure (zoom of the diagnostic region).*

2.4. Characterization and reactivity of functionalized Zincke imine 2a

To isolate functionalized derivative **2a**, photochemical reactions were set-up (12 x 25 mg of **2**) without a subsequent ring closure step.

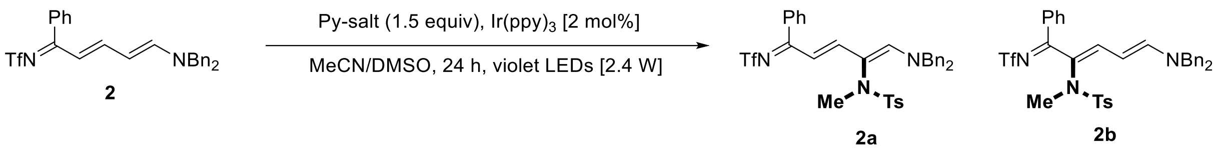

The reaction mixture after work-up was subjected to two consecutive columns chromatography in the hexanes/DCM/AcOEt (80:10:10) eluent system. It allowed to obtain ~ 100 mg of pure intermediate **2a** (most of compounds were disposed as mixed fractions). **^{1}H NMR** (500 MHz, $CDCl_3$) δ 7.77 (d, J = 7.9 Hz, 2H), 7.54 – 6.85 (m, 18H), 5.69 (s, 1H), 5.44 (s, 1H), 4.48 (d, J = 15.4 Hz, 4H), 2.86 (s, 3H), 2.35 (s, 3H); **^{13}C NMR** (126 MHz, $CDCl_3$) δ 178.0, 156.5, 156.1, 144.9, 137.5, 135.7, 134.9, 134.1, 131.5, 130.0, 129.3, 129.2, 128.6, 128.3, 127.5, 127.3, 126.9, 120.7, 118.1, 113.4, 111.3, 62.3, 51.7, 36.7, 21.5; **HRMS** (ESI) calcd. for $C_{34}H_{33}N_3O_4S_2$ [M+H] 668.1865; found 668.1863; Single crystal was obtained by diffuse *n*-hexane to the solution of **2a** in HPLC grade DCM.

^{1}H NMR, ^{1}H-^{1}H COSY spectra analysis along with X-ray analysis of a single crystal (obtained by diffusion of hexanes to DCM solution of intermediate **2a**) unambiguously confirmed the constitution of intermediate **2a**.

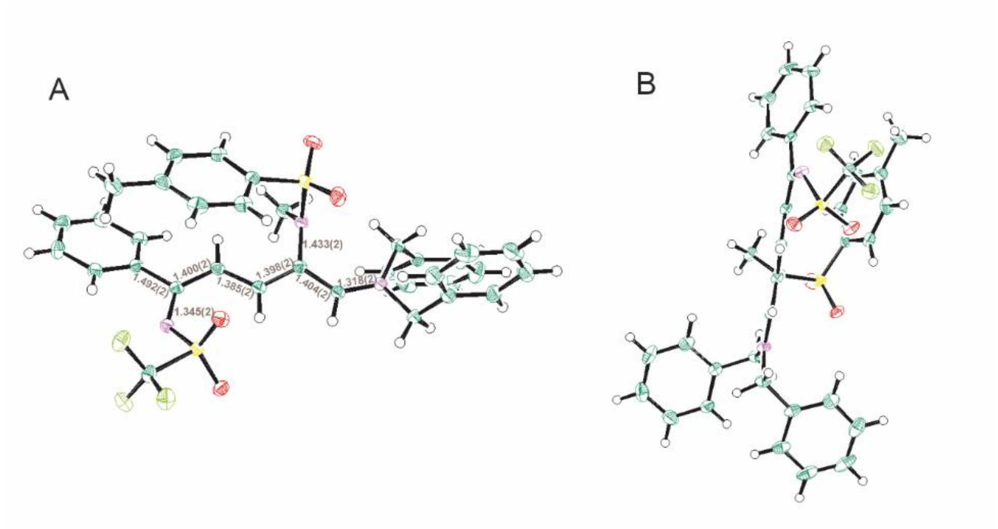

Supplementary Figure 12. *Crystal structure of compound* ***2a*** *(50% probability) A) top view, B) side view (solvent was omitted for clarity)*

In addition, the isolated pure sample of intermediate **2a** was subjected to the ring closure reaction under standard conditions. The crude reaction mixture was monitored using the calibrated GC-FID method, the analysis clearly indicated the formation of only one product (expected compound **3a**).

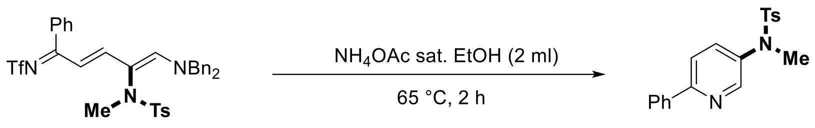

2a 3a

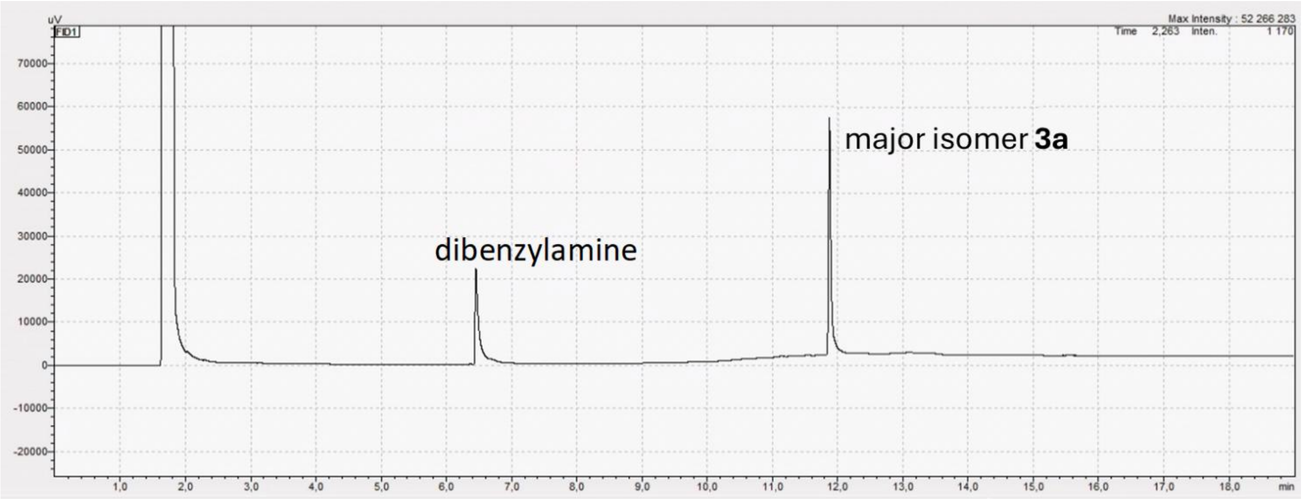

Supplementary Figure 13. *GC FID chromatogram from ring closing of pure isomer **2a**.*

3. Preparation of substrates

3.1. Preparation of substituted pyridines

Arylpyridines were prepared according to the **general procedure 1**.[4]

General Procedure 1. To a closed-cup vial 2-bromopyridine (250 mg, 1.59 mmol), arylboronic acid (2.23 mmol), Na_2CO_3 (1.35 g, 12.74 mmol), and $Pd(PPh_3)_4$ (5 mol%, 92.0 mg, 0.08 mmol) were added and the vial was closed under argon. Through the septum, toluene (3 ml) and H_2O: EtOH mixture (4 ml, 1:1 v/v) was added. The reaction mixture was heated at 100 °C for 18 h +/- 1h, while intensively stirred. After being cooled to room temperature, the vial was opened, and an upper layer was collected with Pasteur's pipette followed by the addition of 5 ml of AcOEt. The combined organic phases were dried over anhydrous sodium sulphate and then evaporated with silica gel (dry load for the preparation of samples for flash chromatography). Pure aryl pyridines were isolated by flash chromatography in the hexanes/AcOEt gradient.

Flash program: time: 25 min; column: silica 12 g; flow rate: 30 mL/min; automatic peak hold: on.

entry	time [min]	hexanes [%]	AcOEt [%]
1	0	100	0
2	25	85	15

2-(4-methoxyphenyl)pyridine (S2a)[5]

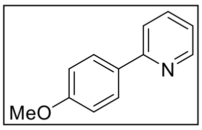

White solid (1.19 mmol, 221 mg, 75%); **^{1}H NMR** (400 MHz, $CDCl_3$) δ 8.68 – 8.62 (m, 1H), 7.98 – 7.93 (m, 2H), 7.74 – 7.65 (m, 2H), 7.20 – 7.14 (m, 1H), 7.03 – 6.96 (m, 2H), 3.87 (s, 3H).

2-(3-methoxyphenyl)pyridine (S3a)[6]

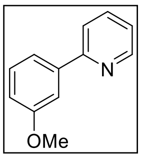

Colorless oil (1.35 mmol, 250 mg, 85%); **^{1}H NMR** (400 MHz, $CDCl_3$) δ 8.74 – 8.66 (m, 1H), 7.79 – 7.71 (m, 2H), 7.62 – 7.53 (m, 2H), 7.39 (t, *J* = 7.9 Hz, 1H), 7.27 – 7.22 (m, 1H), 6.98 (ddd, *J* = 8.2, 2.7, 1.0 Hz, 1H), 3.91 (s, 3H).

2-(3,5-dimethoxyphenyl)pyridine (S4a)[7]

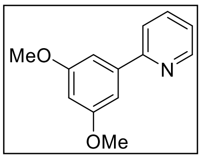

Colorless oil (1.51 mmol, 325 mg, 95%); **^{1}H NMR** (400 MHz, $CDCl_3$) δ 8.69 (ddd, *J* = 4.9, 1.8, 1.0 Hz, 1H), 7.77 – 7.72 (m, 1H), 7.70 (d, *J* = 7.9 Hz, 1H), 7.26 – 7.21 (m, 1H), 7.16 (d, *J* = 2.3 Hz, 2H), 6.54 (t, *J* = 2.3 Hz, 1H), 3.88 (s, 6H).

2-(2,6-dimethoxyphenyl)pyridine (S5a)[8]

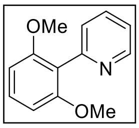

Colorless oil (0.97 mmol, 207 mg, 61%); **^{1}H NMR** (400 MHz, $CDCl_3$) δ 8.74 (ddd, *J* = 4.9, 1.9, 1.0 Hz, 1H), 7.76 – 7.69 (m, 1H), 7.36 – 7.29 (m, 2H), 7.23 (ddd, *J* = 7.6, 4.9, 1.2 Hz, 1H), 6.67 (s, 1H), 6.65 (s, 1H), 3.74 (s, 6H).

2-(2-(methylthio)phenyl)pyridine (S6a)[9]

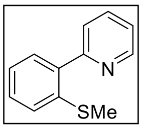

Colorless oil (0.92 mmol, 185 mg, 58%); **^{1}H NMR** (400 MHz, $CDCl_3$) δ 8.74 – 8.69 (m, 1H), 7.75 (td, *J* = 7.7, 1.8 Hz, 1H), 7.56 (dt, *J* = 7.9, 1.1 Hz, 1H), 7.46 – 7.41 (m, 1H), 7.40 – 7.33 (m, 2H), 7.29 – 7.21 (m, 2H), 2.39 (s, 3H).

2-(2-tolyl)pyridine (S7a)[6]

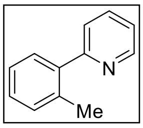

Colorless oil (1.03 mmol, 175 mg); **^{1}H NMR** (400 MHz, $CDCl_3$) δ 8.70 (d, *J* = 4.9 Hz, 1H), 7.74 (td, *J* = 7.7, 1.8 Hz, 1H), 7.40 (d, *J* = 7.8 Hz, 2H), 7.32 – 7.21 (m, 4H), 2.37 (s, 3H).

2-(2,4-dimethylphenyl)pyridine (S8a)[10]

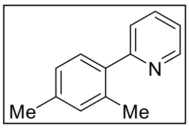

Colorless oil (1.40 mmol, 256 mg, 88%); **^{1}H NMR** (400 MHz, $CDCl_3$) δ 8.72 – 8.66 (m, 1H), 7.73 (td, *J* = 7.7, 1.8 Hz, 1H), 7.39 (dt, *J* = 7.8, 1.1 Hz, 1H), 7.32 (d, *J* = 7.6 Hz, 1H), 7.23 (ddd, *J* = 7.5, 4.9, 1.2 Hz, 1H), 7.10 (d, *J* = 8.3 Hz, 2H), 2.38 (s, 6H).

2-(3,5-di-*tert*-butylphenyl)pyridine (S9a)[11]

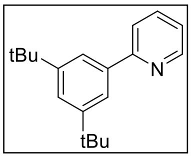

White solid (1.49 mmol, 397 mg, 93%); **^{1}H NMR** (400 MHz, $CDCl_3$) δ 8.70 (ddd, *J* = 4.9, 1.8, 1.1 Hz, 1H), 7.80 (d, *J* = 1.8 Hz, 2H), 7.76 – 7.68 (m, 2H), 7.50 (t, *J* = 1.8 Hz, 1H), 7.21 (ddd, *J* = 6.7, 4.8, 1.8 Hz, 1H), 1.39 (s, 18H).

2-(pyren-1-yl)pyridine (S10a)[5]

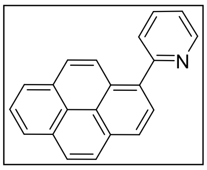

Off-White solid (1.28 mmol, 360 mg, 81%); **^{1}H NMR** (400 MHz, $CDCl_3$) δ 8.89 (ddd, *J* = 4.9, 1.9, 1.0 Hz, 1H), 8.40 (d, *J* = 9.3 Hz, 1H), 8.27 (d, *J* = 7.9 Hz, 1H), 8.24 – 8.16 (m, 3H), 8.12 (s, 2H), 8.09 (d, *J* = 9.3 Hz, 1H), 8.03 (t, *J* = 7.6 Hz, 1H), 7.90 (td, *J* = 7.7, 1.9 Hz, 1H), 7.75 (dt, *J*=7.8, 1.1, 1H), 7.39 (ddd, *J* = 7.6, 4.9, 1.2 Hz, 1H).

2-(phenanthren-9-yl)pyridine (S11a)[5]

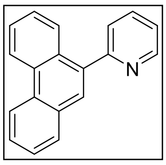

White solid (1.28 mmol, 309 mg, 80%) **^{1}H NMR** (400 MHz, $CDCl_3$) δ 8.83 (ddd, *J* = 4.9, 1.8, 1.0 Hz, 1H), 8.80 – 8.77 (m, 1H), 8.73 (dd, *J* = 8.2, 0.6 Hz, 1H), 8.08 (dd, *J* = 8.6, 1.3 Hz, 1H), 7.93 (dd, *J* = 7.9, 1.5 Hz, 1H), 7.89 – 7.83 (m, 2H), 7.72 – 7.55 (m, 5H), 7.40 – 7.35 (m, 1H).

9-(4-(pyridin-2-yl)phenyl)-9H-carbazole (S12a)[12]

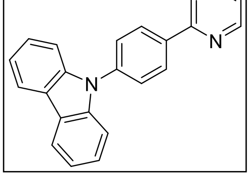

White solid (1.29 mmol, 413 mg, 81%); **^{1}H NMR** (400 MHz, $CDCl_3$) δ 8.77 (dt, *J* = 4.7, 1.4 Hz, 1H), 8.27 – 8.22 (m, 2H), 8.17 (d, *J* = 7.8 Hz, 2H), 7.85 – 7.79 (m, 2H), 7.73 – 7.68 (m, 2H), 7.50 (d, *J* = 9.2 Hz, 2H), 7.48 – 7.41 (m, 2H), 7.35 – 7.28 (m, 3H).

2-(naphthalen-2-yl)pyridine (S13a)[13]

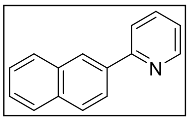

White solid (1.28 mmol, 261 mg, 80%); **^{1}H NMR** (400 MHz, $CDCl_3$) δ 8.77 (ddd, *J* = 4.8, 1.9, 0.9 Hz, 1H), 8.50 (d, *J* = 1.9 Hz, 1H), 8.16 (dd, *J* = 8.6, 1.8 Hz, 1H), 8.01 – 7.85 (m, 4H), 7.81 (td, *J* = 7.7, 1.9 Hz, 1H), 7.57 – 7.47 (m, 2H), 7.33 – 7.24 (m, 1 H).

2-([1,1'-biphenyl]-2-yl)pyridine (S14a)[14]

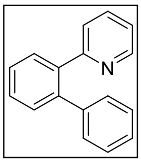

Colorless oil (1.27 mmol, 293 mg, 80%); **^{1}H NMR** (400 MHz, $CDCl_3$) δ 8.63 (ddd, *J* = 4.9, 1.9, 1.0 Hz, 1H), 7.73 – 7.67 (m, 1H), 7.50 – 7.42 (m, 3H), 7.41 – 7.35 (m, 1H), 7.25 – 7.21 (m, 3H), 7.18 – 7.14 (m, 2H), 7.12 – 7.07 (m, 1H), 6.88 (dt, *J* = 7.9, 1.1 Hz, 1H).

2-([1,1'-biphenyl]-3-yl)pyridine (S15a)[14]

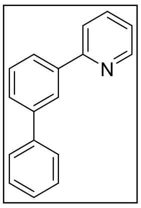

Colorless oil (1.56 mmol, 366 mg, 98%); **^{1}H NMR** (400 MHz, $CDCl_3$) δ 8.74 (dt, *J* = 4.7, 1.3 Hz, 1H), 8.25 (t, *J* = 1.8 Hz, 1H), 8.04 – 7.94 (m, 1H), 7.84 – 7.74 (m, 2H), 7.68 (ddt, *J* = 13.1, 7.5, 1.5 Hz, 2H), 7.56 (t, *J* = 7.7 Hz, 1H), 7.51 – 7.44 (m, 2H), 7.41 – 7.36 (m, 1H), 7.33 – 7.20 (m, 2H).

2-([1,1'-biphenyl]-4-yl)pyridine (S16a)[14]

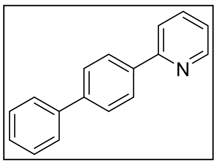

White solid (1.28 mmol, 298 mg, 81%); **^{1}H NMR** (400 MHz, $CDCl_3$) δ 8.72 (dt, *J* = 4.8, 1.4 Hz, 1H), 8.12 – 8.05 (m, 2H), 7.78 (dd, *J* = 6.3, 1.6 Hz, 2H), 7.74 – 7.70 (m, 2H), 7.67 (dd, *J* = 8.3, 1.3 Hz, 2H), 7.47 (t, *J* = 7.5 Hz, 2H), 7.40 – 7.34 (m, 1H), 7.26 – 7.22 (m, 1H).

4-(pyridin-2-yl)benzonitrile (S17a)[15]

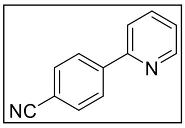

White solid (1.18 mmol, 212 mg, 74%); **^{1}H NMR** (400 MHz, $CDCl_3$) δ 8.74 (ddd, *J* = 4.8, 1.8, 1.0 Hz, 1H), 8.15 – 8.10 (m, 2H), 7.84 – 7.79 (m, 1H), 7.79 – 7.75 (m, 3H), 7.32 (ddd, *J* = 7.2, 4.8, 1.4 Hz, 1H).

2-(4-nitrophenyl)pyridine (S18a)[5]

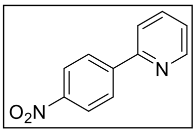

White solid (1.00 mmol, 200 mg, 63%); **^{1}H NMR** (400 MHz, $CDCl_3$) δ 8.77 (dt, *J* = 4.8, 1.4 Hz, 1H), 8.38 – 8.30 (m, 2H), 8.23 – 8.15 (m, 2H), 7.89 – 7.78 (m, 2H), 7.35 (ddd, *J* = 6.2, 4.8, 2.4 Hz, 1H).

2-(3-nitrophenyl)pyridine (S19a)[16]

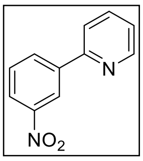

White solid (0.94 mmol, 187 mg, 59%); **^{1}H NMR** (400 MHz, $CDCl_3$) δ 8.88 (t, *J* = 2.0 Hz, 1H), 8.76 (dt, *J* = 4.9, 1.4 Hz, 1H), 8.39 (dt, *J* = 7.8, 1.4 Hz, 1H), 8.28 (ddd, *J* = 8.2, 2.3, 1.1 Hz, 1H), 7.89 – 7.79 (m, 2H), 7.67 (t, *J* = 8.0 Hz, 1H), 7.34 (ddd, *J* = 5.7, 4.8, 2.7 Hz, 1H).

2-(4-(trifluoromethyl)phenyl)pyridine (S20a)[17]

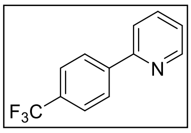

White solid (1.32 mmol, 294 mg, 83%); **^{1}H NMR** (400 MHz, $CDCl_3$) δ 8.73 (ddd, *J* = 4.8, 1.8, 1.1 Hz, 1H), 8.15 – 8.08 (m, 2H), 7.84 – 7.78 (m, 1H), 7.77 (dd, *J* = 1.6, 1.0 Hz, 1H), 7.76 – 7.69 (m, 2H), 7.29 (ddd, *J* = 6.7, 4.8, 1.7 Hz, 1H).

2-(3,5-difluorophenyl)pyridine (S21a)[7]

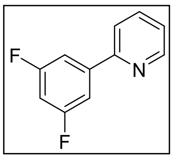

White solid (0.67 mmol, 128 mg, 42%); **^{1}H NMR** (400 MHz, $CDCl_3$) δ 8.70 (ddd, *J* = 4.9, 1.8, 1.0 Hz, 1H), 7.78 (td, *J* = 7.7, 1.8 Hz, 1H), 7.69 (d, *J* = 8.0 Hz, 1H), 7.59 – 7.51 (m, 2H), 7.31 – 7.27 (m, 1H), 6.85 (tt, *J* = 8.7, 2.4 Hz, 1H).

2-(furan-2-yl)pyridine (S22a)[17]

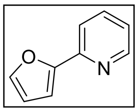

Colorless oil (0.74 mmol, 108 mg, 47%); **^{1}H NMR** (400 MHz, $CDCl_3$) δ 8.62 – 8.56 (m, 1H), 7.74 – 7.66 (m, 2H), 7.55 – 7.51 (m, 1H), 7.15 (ddd, *J* = 6.7, 4.9, 2.1 Hz, 1H), 7.05 (dd, *J* = 3.4, 0.8 Hz, 1H), 6.53 (dd, *J* = 3.4, 1.8 Hz, 1H).

2-(thiophen-2-yl)pyridine (S23a)[5]

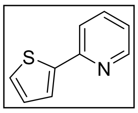

Off-white solid (1.13 mmol, 182 mg, 71%); **^{1}H NMR** (400 MHz, $CDCl_3$) δ 8.64 – 8.46 (m, 1H), 7.71 – 7.61 (m, 2H), 7.58 (td, *J* = 3.6, 1.2 Hz, 1H), 7.39 (ddt, *J* = 6.1, 4.1, 2.0 Hz, 1H), 7.12 (ddt, *J* = 12.0, 5.0, 3.4 Hz, 2H).

2-(benzo[b]thiophen-2-yl)pyridine (S24a)[17]

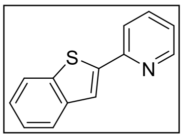

Tan solid (0.72 mmol, 151 mg, 45%); **^{1}H NMR** (400 MHz, $CDCl_3$) δ 8.79 – 8.72 (m, 1H), 8.47 (dd, *J* = 8.3, 1.1 Hz, 1H), 7.94 – 7.87 (m, 1H), 7.82 – 7.76 (m, 2H), 7.70 (dt, *J* = 7.9, 1.1 Hz, 1H), 7.47 – 7.43 (m, 1H), 7.40 (ddd, *J* = 8.2, 7.1, 1.4 Hz, 1H), 7.30 – 7.26 (m, 1H).

2-(benzo[b]thiophen-3-yl)pyridine (S25a)[17]

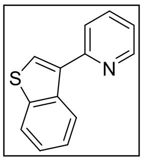

Pink oil (0.52 mmol, 111 mg, 33%); **^{1}H NMR** (400 MHz, $CDCl_3$) δ 8.76 (ddd, *J* = 4.9, 1.8, 1.0 Hz, 1H), 8.50 – 8.44 (m, 1H), 7.95 – 7.88 (m, 1H), 7.83 – 7.76 (m, 2H), 7.73 – 7.67 (m, 1H), 7.49 – 7.36 (m, 2H), 7.30 – 7.26 (m, 1H).

***tert*-butyl 2-(pyridin-2-yl)-1*H*-pyrrole-1-carboxylate (S26a)**[17]

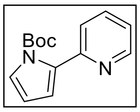

Colorless oil (0.57 mmol, 140 mg, 36%); **^{1}H NMR** (400 MHz, $CDCl_3$) δ 8.61 (ddd, *J* = 4.9, 1.8, 1.0 Hz, 1H), 7.71 – 7.64 (m, 1H), 7.39 (dt, *J* = 7.9, 1.1 Hz, 1H), 7.36 (dd, *J* = 3.3, 1.7 Hz, 1H), 7.19 (ddd, *J* = 7.5, 4.9, 1.2 Hz, 1H), 6.41 (dd, *J* = 3.3, 1.7 Hz, 1H), 6.24 (t, *J* = 3.3 Hz, 1H), 1.36 (s, 9H).

***tert*-butyl 2-(pyridin-2-yl)-1*H*-indole-1-carboxylate (S27a)**[17]

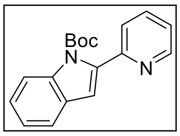

Colorless oil (1.05 mmol, 309 mg, 66%); **^{1}H NMR** (400 MHz, $CDCl_3$) δ 8.67 (ddd, *J* = 4.9, 1.9, 1.0 Hz, 1H), 8.19 (d, *J* = 8.3 Hz, 1H), 7.74 (td, *J* = 7.7, 1.8 Hz, 1H), 7.58 (d, *J* = 7.7 Hz, 1H), 7.51 (d, *J* = 7.9 Hz, 1H), 7.36 (ddd, *J* = 8.4, 7.2, 1.3 Hz, 1H), 7.29 – 7.22 (m, 2H), 6.77 (s, 1H), 1.34 (s, 9H).

2,5-diphenylpyridine (S28a)[18]

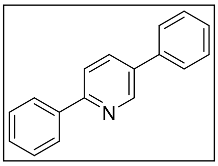

3 Equivalents of phenylboronic acid and 2,5-dibromopyridine (1.59 mmol) were used; white solid (0.97 mmol, 224 mg, 61%); **^{1}H NMR** (400 MHz, $CDCl_3$) δ 8.94 (dd, *J* = 2.4, 0.9 Hz, 1H), 8.09 – 8.01 (m, 2H), 7.96 (dd, *J* = 8.3, 2.4 Hz, 1H), 7.81 (dd, *J* = 8.3, 0.9 Hz, 1H), 7.68 – 7.60 (m, 2H), 7.54 – 7.49 (m, 3H), 7.49 – 7.37 (m, 3H).

***N*,*N*-dimethyl-4-(pyridin-2-yl)aniline (S29a)**[17]

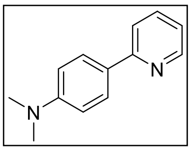

Off-white solid (0.91 mmol, 180 mg, 57%); **^{1}H NMR** (400 MHz, $CDCl_3$) δ 8.66 – 8.60 (m, 1H), 7.97 – 7.89 (m, 2H), 7.72 – 7.61 (m, 2H), 7.11 (ddd, *J* = 6.7, 4.9, 2.1 Hz, 1H), 6.85 – 6.77 (m, 2H), 3.03 (s, 6H).

2-(2-(trifluoromethyl)phenyl)pyridine (S30a)[14]

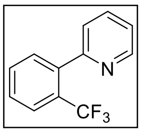

Colorless oil (0.97 mmol, 216 mg, 61%); **^{1}H NMR** (400 MHz, $CDCl_3$) δ 8.70 (dt, *J* = 4.9, 1.3 Hz, 1H), 7.81 – 7.70 (m, 2H), 7.63 (td, *J* = 7.6, 1.3 Hz, 1H), 7.58 – 7.48 (m, 2H), 7.44 (d, *J* = 7.8 Hz, 1H), 7.32 (ddd, *J* = 7.6, 4.9, 1.2 Hz, 1H).

Other pyridines

2-benzylpyridine (S31a); prepared according to the Seto's procedure.[19]

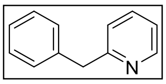

2 Steps; pale yellow oil (3.18 mmol, 538 mg, 43%), ~ 80% purity based on the ^{1}H NMR, the compound was used in this form for the preparation of the Zincke imine); ^{1}H NMR (400 MHz, $CDCl_3$) δ 8.57 – 8.53 (m, 1H), 7.57 (td, *J* = 7.7, 1.9 Hz, 1H), 7.34 – 7.18 (m, 5H), 7.13 – 7.07 (m, 2H), 4.16 (s, 2H).

2-(methoxy(phenyl)methyl)pyridine (S32a); prepared according to the Wong's procedure.[20]

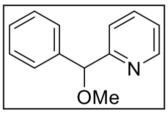

2 Steps; pale yellow oil (2.15 mmol, 428 mg, 84%); **^{1}H NMR** (400 MHz, $CDCl_3$) δ 8.58 – 8.52 (m, 1H), 7.68 (td, *J* = 7.7, 1.8 Hz, 1H), 7.50 (d, *J* = 7.8 Hz, 1H), 7.46 – 7.41 (m, 2H), 7.34 (dd, *J* = 8.4, 6.7 Hz, 2H), 7.30 – 7.22 (m, 1H), 7.16 (ddd, J = 7.5, 4.9, 1.2 Hz, 1H), 5.39 (s, 1H), 3.45 (s, 3H).

phenyl(pyridin-2-yl)methanol (S33a); prepared according to the Seto's procedure.[19]

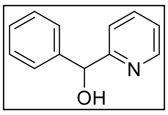

Pale yellow oil (7.95 mmol, 1.47 g, 97%); **^{1}H NMR** (400 MHz, $CDCl_3$) δ 8.57 (ddd, *J* = 4.9, 1.8, 1.0 Hz, 1H), 7.61 (td, J = 7.7, 1.7 Hz, 1H), 7.41 – 7.33 (m, 3H), 7.33 – 7.28 (m, 1H), 7.28 – 7.20 (m, 1H), 7.20 – 7.11 (m, 2H), 5.75 (d, *J* = 4.0 Hz, 1H), 5.23 (d, *J* = 4.3 Hz, 1H).

***N*,*N*-diphenylpyridin-2-amine (S34a) prepared according to the Verkade's procedure.**[21]

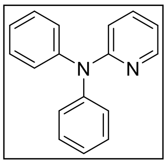

Off-white solid (1.27 mmol, 313 mg, 71%); ^{1}H NMR (400 MHz, $CDCl_3$) δ 8.23 (dd, *J* = 5.0, 1.2 Hz, 1H), 7.48 – 7.40 (m, 1H), 7.36 – 7.27 (m, 4H), 7.18 (d, *J* = 8.4 Hz, 4H), 7.16 – 7.09 (m, 2H), 6.81 – 6.72 (m, 2H).

2,5-diphenylpyridine (S35a) prepared according to the Wierks procedure.[22]

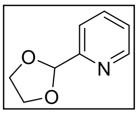

Pale yellow oil (15.9 mmol, 2.40 g, 80%); **^{1}H NMR** (400 MHz, $CDCl_3$) δ 8.62 (ddd, *J* = 4.8, 1.8, 0.9 Hz, 1H), 7.73 (td, *J* = 7.7, 1.8 Hz, 1H), 7.53 (dt, *J* = 7.8, 1.1 Hz, 1H), 7.31 – 7.24 (m, 1H), 5.86 (s, 1H), 4.23 – 4.02 (m, 4H).

3.2.Preparation of Zincke imines

Zincke imines were prepared according to **general procedure 2 (modified McNally's procedure)**. [1]

General Procedure 2. In a closed-cup vial, pyridine (0.1 mmol) was dissolved in anhydrous DCM (10 ml) and cooled to -78 °C (acetone/dry ice bath) followed by the addition of trifluoromethanesulfonic anhydride (1 mmol, 1.0 equiv.). The reaction mixture was stirred for 1 h at -78 ° C. After an indicated time, the solution of dibenzylamine (1.2 mmol, 1.2 equiv.) in anhydrous DCM (1 ml) was added dropwise and stirring was continued for another 30 min at -78 °C. The reaction mixture was removed from the cryogenic bath and allowed to reach room temperature. The crude reaction mixture was evaporated with silica gel (dry load for preparation of the sample for flash chromatography) and the pure product was isolated by flash chromatography in hexanes/DCM (85:15)/ AcOEt gradient.

Flash program: time: 25 min; column: silica 12 g; flow rate: 30 mL/min; automatic peak hold: on.

entry	time [min]	hexanes/DCM (85:10 v/v) [%]	AcOEt [%]
1	0	100	0
2	25	80	20

***N*-((1*E*,2*E*,4*E*)-5-(dibenzylamino)-1-phenylpenta-2,4-dien-1-ylidene)-1,1,1-trifluoromethanesulfonamide (2)**[1]

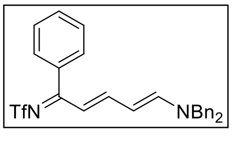

Orange solidified foam (0.7 mmol, 338 mg, 70%); **^{1}H NMR** (400 MHz, $CDCl_3$) δ 7.60 (dd, *J* = 6.9, 1.6 Hz, 2H), 7.54 – 7.48 (m, 1H), 7.47 – 7.36 (m, 8H), 7.34 – 7.25 (m, 2H), 7.16 (dd, *J* = 7.7, 1.8 Hz, 4H), 6.72 (d, *J* = 13.7 Hz, 1H), 5.84 (t, *J* = 12.2 Hz, 1H), 4.44 (s, 4H).

***N*-((1*Z*,2*E*,4*E*)-5-(dibenzylamino)-1-(4-methoxyphenyl)penta-2,4-dien-1-ylidene)-1,1,1-trifluoromethanesulfonamide (S2b)**

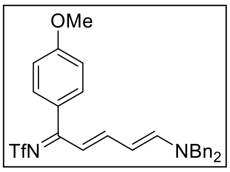

Yellow solid (0.48 mmol, 246 mg, 48%); **^{1}H NMR** (600 MHz, $CDCl_3$) δ 7.64 – 7.61 (m, 2H), 7.39 (tt, J = 8.8, 6.0 Hz, 7H), 7.29 (d, *J* = 12.4 Hz, 1H), 7.19 – 7.12 (m, 4H), 6.95 – 6.91 (m, 2H), 6.66 (d, *J* = 13.8 Hz, 1H), 5.81 (t, *J* = 12.2 Hz, 1H), 4.42 (s, 4H), 3.86 (s, 3H); **^{13}C NMR** (126 MHz, $CDCl_3$) δ 177.3, 162.6, 159.1, 156.9, 131.9, 130.3, 129.2, 128.7, 128.3, 127.7, 127.2, 120.8, 118.3, 114.3, 113.6, 102.3, 59.6, 55.4, 51.3; **HRMS** (ESI) calcd. for $C_{27}H_{26}F_3N_2O_3S$ [M+H] 515.1611; found 515.1616.

***N*-((1*Z*,2*E*,4*E*)-5-(dibenzylamino)-1-(3-methoxyphenyl)penta-2,4-dien-1-ylidene)-1,1,1-trifluoromethanesulfonamide (S3b)**

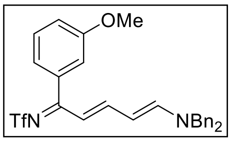

Orange solid (0.22 mmol, 113 mg, 22%); **^{1}H NMR** (500 MHz, $CDCl_3$) δ 7.38 (d, *J* = 7.2 Hz, 6H), 7.38 – 7.31 (m, 2H), 7.33 – 7.26 (m, 1H), 7.14 (ddd, *J* = 7.8, 6.1, 2.0 Hz, 6H), 7.03 (ddd, J = 8.3, 2.7, 1.0 Hz, 1H), 6.68 (d, *J* = 13.7 Hz, 1H), 5.83 (t, *J* = 12.1 Hz, 1H), 4.44 (s, *2*H), 4.42 (s, 2H), 3.83 (s, 3H); **^{13}C NMR** (126 MHz, $CDCl_3$) δ 177.9, 159.9, 159.3, 157.4, 139.3, 134.2, 133.7, 129.2, 129.1, 128.8, 128.4, 127.7, 127.2, 122.1, 118.2, 117.0, 114.7, 114.5, 102.6, 59.7, 55.4, 51.4, 30.9; **HRMS** (ESI) calcd. for $C_{27}H_{26}F_3N_2O_3S$ [M+H] 515.1611; found 515.1619.

_N_-((1_E_,2_E_,4_E_)-5-(dibenzylamino)-1-(3,5-dimethoxyphenyl)penta-2,4-dien-1-ylidene)-1,1,1-trifluoromethanesulfonamide (S4b)

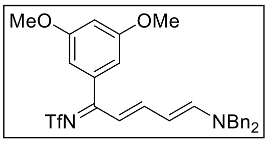

Yellow solid (0.81 mmol, 441 mg, 81%); **^{1}H NMR** (400 MHz, $CDCl_3$) δ 7.45 – 7.28 (m, 8H), 7.15 (dd, *J* = 7.6, 1.9 Hz, 4H), 6.73 (d, *J* = 2.3 Hz, 2H), 6.66 (d, *J* = 13.6 Hz, 1H), 6.57 (t, *J* = 2.3 Hz, 1H), 5.82 (t, *J* = 12.2 Hz, 1H), 4.43 (d, *J* = 7.1 Hz, 4H), 3.81 (s, 6H); **^{13}C NMR** (101 MHz, $CDCl_3$) δ 177.8, 160.4, 160.0, 157.6, 139.9, 134.2, 129.2, 128.8, 128.4, 127.8, 127.2, 121.1, 117.9, 114.4, 107.6, 103.0, 102.6, 59.8, 55.5, 51.9; **HRMS** (ESI) calcd. for $C_{28}H_{28}F_3N_2O_4S$ [M+H] 545.1722; found 545.1735.

_N_-((1_Z_,2_E_,4_E_)-5-(dibenzylamino)-1-(2,6-dimethoxyphenyl)penta-2,4-dien-1-ylidene)-1,1,1-trifluoromethanesulfonamide (S5b)

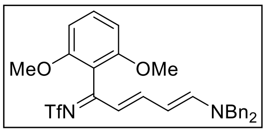

Yellow solid (0.48 mmol, 262 mg, 48%); **^{1}H NMR** (400 MHz, $CDCl_3$) δ 7.44 – 7.28 (m, 7H), 7.24 – 7.01 (m, 6H), 6.60 (s, 2H), 6.47 (s, 1H), 5.73 (t, *J* = 12.2 Hz, 1H), 4.37 (s, 4H), 3.81 (s, 6H); **^{13}C NMR** (101 MHz, $CDCl_3$) δ 157.2, 156.1, 130.9, 129.1, 128.3, 127.6, 118.4, 104.3, 101.9, 56.41; **HRMS** (ESI) calcd. for $C_{28}H_{28}F_3N_2O_4S$ [M+H] 545.1722; found 545.1727.

_N_-((1_Z_,2_E_,4_E_)-5-(dibenzylamino)-1-(2-(methylthio)phenyl)penta-2,4-dien-1-ylidene)-1,1,1-trifluoromethanesulfonamide (S6b)

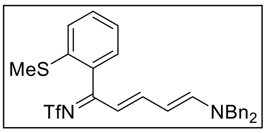

Yellow solid (0.32 mmol, 170 mg, 32%); **^{1}H NMR** (500 MHz, $CDCl_3$) δ 7.37 (d, *J* = 7.4 Hz, 8H), 7.25 – 7.17 (m, 3H), 7.13 (d, *J* = 7.6 Hz, 4H), 6.99 (d, *J* = 12.7 Hz, 1H), 6.67 (d, *J* = 13.5 Hz, 1H), 5.81 (t, *J* = 12.2 Hz, 1H), 4.39 (s, 4H), 2.46 (s, 3H); **^{13}C NMR** (126 MHz, $CDCl_3$) δ 180.9, 162.3, 160.1, 140.2, 139.8, 132.4, 131.9, 131.6, 131.5, 131.1, 130.4, 130.2, 129.9, 127.6, 118.6, 105.2, 62.3, 53.9, 19.7; **HRMS** (ESI) calcd. for $C_{38}H_{31}F_3N_3O_2S$ [M+H] 531.1388; found 531.1392.

_N_-((1_Z_,2_E_,4_E_)-5-(dibenzylamino)-1-(o-tolyl)penta-2,4-dien-1-ylidene)-1,1,1-trifluoromethanesulfonamide (S7b)

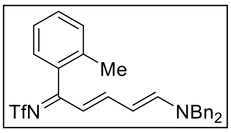

Yellow solid (0.32 mmol, 170 mg, 32%); **^{1}H NMR** (400 MHz, $CDCl_3$) δ 7.38 (s, 1H), 7.37 (d, *J* = 3.7 Hz, 1H), 7.36 – 7.27 (m, 5H), 7.27 – 7.08 (m, 8H), 6.93 (t, *J* = 12.8 Hz, 1H), 6.68 (d, *J* = 13.7 Hz, 1H), 5.78 (t, *J* = 12.2 Hz, 1H), 4.39 (s, 4H), 2.32 (s, 3H); **^{13}C NMR** (101 MHz, $CDCl_3$) δ 180.2, 159.5, 157.1, 137.2, 135.8, 130.4, 129.2, 129.2, 128.4, 127.7, 127.2, 125.2, 116.3, 102.1, 19.3; **HRMS** (ESI) calcd. for $C_{27}H_{26}F_3N_2O_2S$ [M+H] 499.1667; found 499.1665.

_N_-((1_Z_,2_E_,4_E_)-5-(dibenzylamino)-1-(2,4-dimethylphenyl)penta-2,4-dien-1-ylidene)-1,1,1-trifluoromethanesulfonamide (S8b)

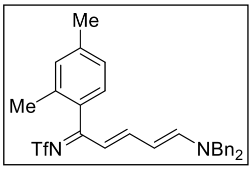

Yellow solid (0.79 mmol 405 mg, 79%); **^{1}H NMR** (500 MHz, $CDCl_3$) δ 7.37 (d, *J* = 7.3 Hz, 6H), 7.13 (d, *J* = 7.9 Hz, 6H), 7.08 – 7.01 (m, 2H), 6.96 (t, *J* = 12.8 Hz, 1H), 6.65 (d, *J* = 13.6 Hz, 1H), 5.77 (t, *J* = 12.2 Hz, 1H), 4.38 (s, 4H), 2.35 (s, 3H), 2.29 (s, 3H); **^{13}C NMR** (126 MHz, $CDCl_3$) δ 183.2, 162.1, 159.6, 141.9, 138.5, 137.0, 136.5, 133.9, 131.8, 131.4, 131.2, 131.0, 130.3, 129.9, 128.5, 123.3, 119.3, 104.6, 23.9, 22.0; **HRMS** (ESI) calcd. for $C_{28}H_{28}F_3N_2O_2S$ [M+H] 513.1824; found 513.1830.

N-((1Z,2E,4E)-1-(3,5-di-tert-butylphenyl)-5-(dibenzylamino)penta-2,4-dien-1-ylidene)-1,1,1-trifluoromethanesulfonamide (S9b)

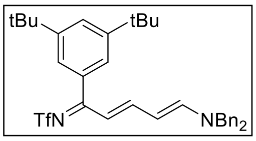

Yellow solid (0.56 mmol, 334,15 mg, 56%); **^{1}H NMR** (600 MHz, $CDCl_3$) δ 7.55 (t, *J* = 1.8 Hz, 1H), 7.44 (d, *J* = 1.8 Hz, 2H), 7.42 – 7.34 (m, 7H), 7.21 (d, *J* = 12.4 Hz, 1H), 7.19 – 7.12 (m, 4H), 6.70 (d, *J* = 13.7 Hz, 1H), 5.85 (t, *J* = 12.2 Hz, 1H), 4.43 (s, 4H), 1.34 (s, 18H); **^{13}C NMR** (151 MHz, $CDCl_3$) δ 179.2, 159.7, 156.7, 150.5, 137.2, 129.2, 127.8, 127.3, 125.4, 124.3, 114.7, 102.4, 34.9, 31.3; **HRMS** (ESI) calcd. for $C_{34}H_{39}F_3N_2O_2S$ [M+H] 597.2757; found 597.2757.

N-((1Z,2E,4E)-5-(dibenzylamino)-1-(pyren-4-yl)penta-2,4-dien-1-ylidene)-1,1,1-trifluoromethanesulfonamide (S10b)

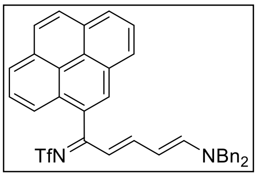

Red solid (0.59 mmol, 359 mg, 59%); **^{1}H NMR** (400 MHz, $CDCl_3$) δ 8.27 – 8.17 (m, 4H), 8.16 – 7.95 (m, 5H), 7.42 –7.27 (m, 6H), 7.16 – 6.77 (m, 7H), 5.83 (t, *J* = 12.0 Hz, 1H), 4.35 (s, 2H) 4.25 (s, 2H); **^{13}C NMR** (126 MHz, $CDCl_3$) δ 179.5, 157.8, 134.2, 133.8, 132.3, 131.4, 131.0, 129.4, 129.4, 129.2, 129.0, 128.7, 128.6, 127.9, 127.4, 127.4, 126.6, 126.5, 125.9, 125.8, 125.0, 124.8, 124.7, 124.3, 117.6, 102.8, 51.5; **HRMS** (ESI) calcd. for $C_{36}H_{28}F_3N_2O_2S$ [M+H] 609.1824; found 609.1821.

N-((1Z,2E,4E)-5-(dibenzylamino)-1-(phenanthren-9-yl)penta-2,4-dien-1-ylidene)-1,1,1-trifluoromethanesulfonamide (S11b)

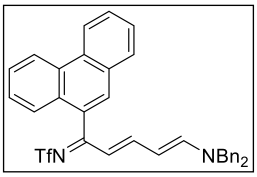

Yellow solid (0.49 mmol, 286 mg, 49%); **^{1}H NMR** (400 MHz, $CDCl_3$) δ 8.72 (t, *J* = 7.5 Hz, 2H), 8.04 (d, *J* = 8.1 Hz, 1H), 7.93 (d, *J* = 8.1 Hz, 1H), 7.77 – 7.59 (m, 5H), 7.36 (s, 6H), 7.17 – 6.85 (m, 7H), 5.82 (t, *J* = 12.1 Hz, 1H), 4.36 (s, 2H), 4.30 (s, 2H); **^{13}C NMR** (101 MHz, $CDCl_3$) δ 178.9, 160.2, 157.8, 134.0, 133.6, 130.7, 130.5, 130.3, 130.0, 129.2, 129.2, 128.8, 128.5, 127.8, 127.6, 127.4, 127.2, 127.2, 127.0, 126.7, 122.7, 122.6, 121.0, 117.8, 116.8, 102.4, 98.5, 77.2, 59.7, 51.3; **HRMS** (ESI) calcd. for $C_{34}H_{28}F_3N_2O_2S$ [M+H] 585.1824; found 585.1821.

N-((1Z,2E,4E)-1-(4-(9H-carbazol-9-yl)phenyl)-5-(dibenzylamino)penta-2,4-dien-1-ylidene)-1,1,1-trifluoromethanesulfonamide (S12b)

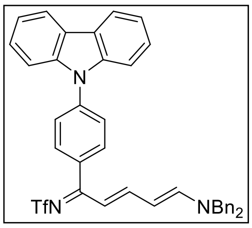

Red solid (0.29 mmol, 188 mg, 29%); **^{1}H NMR** (400 MHz, $CDCl_3$) δ 7.74 – 7.63 (m, 4H), 7.44 – 7.27 (m, 8H), 7.15 (d, *J* = 5.8 Hz, 4H) 6.67 (d, *J* = 13.6 Hz, 1H), 5.90 (t, *J* = 12.1 Hz, 1H), 4.47 (d, *J* = 11.1 Hz, 4H); **^{13}C NMR** (126 MHz, $CDCl_3$) δ 176.7, 160.1, 158.1, 140.8, 140.5, 136.9, 133.9, 131.6, 129.5, 129.1, 128.7, 128.0, 127.5, 126.5, 126.3, 123.9, 120.7, 120.6, 114.5, 110.0, 103.2, 60.1, 51.7; **HRMS** (ESI) calcd. for $C_{38}H_{31}F_3N_3O_2S$ [M+H] 650.2089; found 650.2084.

N-((1Z,2E,4E)-5-(dibenzylamino)-1-(naphthalen-1-yl)penta-2,4-dien-1-ylidene)-1,1,1-trifluoromethanesulfonamide (S13b)

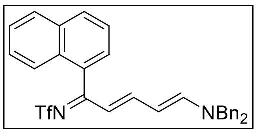

Red solid (0.51 mmol, 272 mg, 51%); **^{1}H NMR** (500 MHz, $CDCl_3$) δ 8.08 (s, 1H), 7.92 (d, *J* = 6.9 Hz, 1H), 7.88 (d, *J* = 8.7 Hz, 2H), 7.69 (dd, *J* = 8.5, 1.8 Hz, 1H), 7.59 – 7.52 (m, 2H), 7.44 – 7.32 (m, 7H), 7.24 (s, 1H), 7.15 (s, 4H), 6.81 (d, *J* = 13.7 Hz, 1H), 5.87 (t, *J* = 12.1 Hz, 1H), 4.42 (s, 4H); **^{13}C NMR** (126 MHz, $CDCl_3$) δ 178.1, 160.0, 157.4, 135.4, 134.5, 134.2, 133.7, 132.4, 130.1, 129.2, 128.9, 128.8,

128.4, 128.0, 127.8, 127.7, 127.6, 127.2, 126.6, 126.4, 120.8, 118.3, 114.8, 102.7, 59.7, 51.4; **HRMS** (ESI) calcd. for $C_{30}H_{26}F_3N_2O_2S$ [M+H] 535.1667; found 535.1663.

***N*-((1*Z*,2*E*,4*E*)-1-([1,1'-biphenyl]-2-yl)-5-(dibenzylamino)penta-2,4-dien-1-ylidene)-1,1,1-trifluoromethanesulfonamide (S14b)**

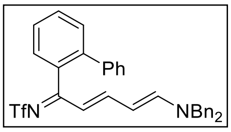

Yellow solid (0.31 mmol, 174 mg, 31%); **^{1}H NMR** (600 MHz, $CDCl_3$) δ 7.54 – 7.45 (m, 2H), 7.45 – 7.28 (m, 14H), 7.19 – 7.01 (m, 5H), 7.01-6.85 6.49-6.33 (s, 1H) 5.57 (t, *J* = 12.2 Hz, 1H), 4.39 (s, 2H), 4.32 (s, 2H); **^{13}C NMR** (151 MHz, $CDCl_3$) δ 179.6, 156.7, 140.5, 130.0, 129.8, 129.1, 128.6, 128.2, 127.6, 127.2, 127.0, 102.2, 51.3. **HRMS** (ESI) calcd. for $C_{32}H_{28}F_3N_2O_2S$ [M+H] 561.1824; found 561.1830. ^{1}H NMR (600 MHz, Chloroform-*d*) δ 7.54 – 7.45 (m, 2H), 7.45 – 7.28 (m, 13H), 7.19 – 7.01 (m, 5H), 5.57 (t, *J* = 12.2 Hz, 1H), 4.35 (d, *J* = 42.9 Hz, 4H).

***N*-((1*Z*,2*E*,4*E*)-1-([1,1'-biphenyl]-3-yl)-5-(dibenzylamino)penta-2,4-dien-1-ylidene)-1,1,1-trifluoromethanesulfonamide (S15b)**

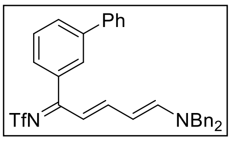

Yellow solid (0.48 mmol, 269 mg, 48%); **^{1}H NMR** (400 MHz, $CDCl_3$) δ 7.80 (s, 1H), 7.74 – 7.68 (m, 1H), 7.64 – 7.43 (m, 6H), 7.43 – 7.32 (m, 8H), 7.29 (d, *J* = 12.3 Hz, 1H), 7.15 (d, *J* = 7.4 Hz, 4H), 6.74 (d, *J* = 13.7 Hz, 1H), 5.84 (t, *J* = 12.2 Hz, 1H), 4.43 (s, 2H), 4.41 (s, 2H); **^{13}C NMR** (101 MHz, $CDCl_3$) δ 178.1, 160.0, 157.6, 141.2, 140.3, 138.6, 134.2, 133.7, 131.1, 129.7, 129.7, 129.2, 128.9, 128.8, 128.7, 128.5, 128.4, 128.2, 128.2, 127.8, 127.7, 127.2, 121.1, 117.9, 114.6, 102.7, 59.7, 51.4; **HRMS** (ESI) calcd. for $C_{32}H_{28}F_3N_2O_2S$ [M+H] 561.1824; found 561.1824.

***N*-((1*Z*,2*E*,4*E*)-1-([1,1'-biphenyl]-4-yl)-5-(dibenzylamino)penta-2,4-dien-1-ylidene)-1,1,1-trifluoromethanesulfonamide (S16b)**

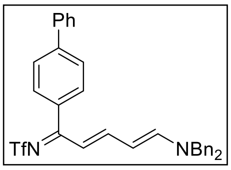

Yellow solid (0.40 mmol, 224 mg, 40%); **^{1}H NMR** (400 MHz, $CDCl_3$) δ 7.74 – 7.60 (m, 4H), 7.49 (d, *J* = 7.1 Hz, 1H), 7.50 – 7.41 (m, 1H), 7.45 – 7.33 (m, 4H), 7.32 (d, *J* = 12.3 Hz, 1H), 7.20 – 7.14 (m, 3H), 6.75 (d, *J* = 13.7 Hz, 1H), 5.87 (t, *J* = 12.1 Hz, 1H), 4.45 (s, 3H); **^{13}C NMR** (101 MHz, $CDCl_3$) δ 177.7, 159.6, 157.3, 144.1, 140.1, 136.8, 130.3, 129.2, 128.9, 128.0, 127.8, 127.2, 126.9, 114.6, 102.6, 51.4. **HRMS** (ESI) calcd. for $C_{32}H_{28}F_3N_2O_2S$ [M+H] 561.1824; found 561.1832.

***N*-((1*Z*,2*E*,4*E*)-1-(4-cyanophenyl)-5-(dibenzylamino)penta-2,4-dien-1-ylidene)-1,1,1-trifluoromethanesulfonamide (S17b)**

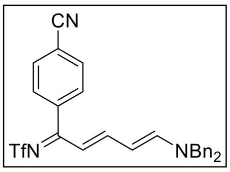

Yellow solid (0.75 mmol, 382 mg, 75%); **^{1}H NMR** (400 MHz, $CDCl_3$) δ 7.74 – 7.63 (m, 4H), 7.44 – 7.27 (m, 8H), 7.15 (d, *J* = 5.8 Hz, 4H), 6.67 (d, *J* = 13.6 Hz, 1H), 5.90 (t, *J* = 12.1 Hz, 1H), 4.48 (s, 2H), 4.45 (s, 2H);**^{13}C NMR** (126 MHz, $CDCl_3$) δ 175.2, 160.4, 158.9, 142.7, 133.9, 133.5, 132.2, 130.2, 129.6, 129.6, 129.3, 128.9, 128.1, 127.4, 121.0, 118.4, 114.4, 114.1, 103.8, 60.3, 51.9; **HRMS** (ESI) calcd. for $C_{27}H_{23}F_3N_3O_2S$ [M+H] 510.1457; found 510.1455.

***N*-((1*Z*,2*E*,4*E*)-5-(dibenzylamino)-1-(4-nitrophenyl)penta-2,4-dien-1-ylidene)-1,1,1-trifluoromethanesulfonamide (S18b)**

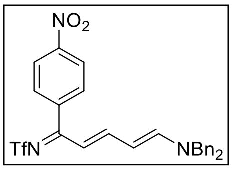

Red solid (0.33 mmol, 175 mg, 33%); **^{1}H NMR** (400 MHz, $CDCl_3$) δ 8.26 (d, *J* = 8.8 Hz, 2H), 7.72 (d, *J* = 8.7 Hz, 2H), 7.39 (d, *J* = 7.2 Hz, 7H), 7.31 (d, *J* = 12.9 Hz, 1H), 7.15 (dd, *J* = 7.2, 2.2 Hz, 4H), 6.69 (d, *J* = 13.6 Hz, 1H), 5.92 (t, *J* = 12.1 Hz, 1H), 4.49 (s, 2H), 4.46 (s, 2H); **^{13}C NMR** (101 MHz, $CDCl_3$) δ 174.5, 160.3, 158.9, 148.9, 144.3, 133.6, 133.2, 130.2, 129.4, 129.1, 128.7, 127.9, 127.2, 123.4, 121.1, 117.9, 114.0, 103.7, 60.1, 51.7; **HRMS** (ESI) calcd. for $C_{26}H_{23}F_3N_3O_4S$ [M+H] 530.1361; found 530.1366.

***N*-((1*Z*,2*E*,4*E*)-5-(dibenzylamino)-1-(3-nitrophenyl)penta-2,4-dien-1-ylidene)-1,1,1-trifluoromethanesulfonamide (S19b)**

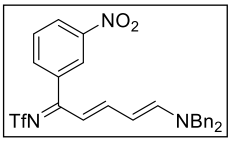

Red solid (0.27 mmol, 143 mg, 27%); **^{1}H NMR** (500 MHz, $CDCl_3$) δ 8.39 (s, 1H), 8.32 (d, *J* = 8.2 Hz, 1H), 7.93 (d, *J* = 7.8 Hz, 1H), 7.62 (t, *J* = 7.9 Hz, 1H), 7.39 (d, *J* = 5.8 Hz, 8H), 7.16 (d, *J* = 7.2 Hz, 4H), 6.70 (d, *J* = 13.5 Hz, 1H), 5.94 (t, *J* = 12.1 Hz, 1H), 4.49 (s, 2H), 4.46 (s, 2H); **^{13}C NMR** (126 MHz, $CDCl_3$) δ 160.3, 159.0, 147.8, 139.8, 135.3, 133.6, 133.2, 129.5, 129.3, 129.1, 128.6, 127.9, 127.2, 125.2, 124.1, 120.7, 118.2, 113.6, 103.8, 60.1, 51.6, 30.9; **HRMS** (ESI) calcd. for $C_{26}H_{23}F_3N_3O_4S$ [M+H] 530.1361; found 530.1366.

***N*-((1*Z*,2*E*,4*E*)-5-(dibenzylamino)-1-(4-(trifluoromethyl)phenyl)penta-2,4-dien-1-ylidene)-1,1,1-trifluoromethanesulfonamide (S20b)**

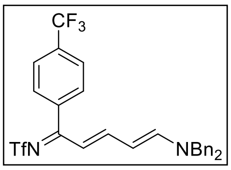

Red solid (0.14 mmol, 77 mg, 14%); **^{1}H NMR** (500 MHz, $CDCl_3$) δ 7.67 (d, *J* = 8.9 Hz, 4H), 7.38 (s, 8H), 7.15 (d, *J* = 6.0 Hz, 4H), 6.69 (d, J = 13.4 Hz, 1H), 5.88 (t, *J* = 12.4 Hz, 1H), 4.47 (s, 2H), 4.44 (s, 2H); **^{13}C NMR** (126 MHz, $CDCl_3$) δ 175.9, 160.2, 158.4, 141.5, 133.9, 133.4, 132.2, 129.9, 129.8, 129.7, 129.4, 129.3, 129.0, 128.6, 127.8, 127.2, 125.2, 125.2, 120.7, 114.2, 103.3, 59.9, 51.5, 50.0; **HRMS** (ESI) calcd. for $C_{27}H_{23}F_6N_2O_2S$ [M+H] 553.1384; found 553.1380.

***N*-((1*Z*,2*E*,4*E*)-5-(dibenzylamino)-1-(3,5-difluorophenyl)penta-2,4-dien-1-ylidene)-1,1,1-trifluoromethanesulfonamide (S21b)**

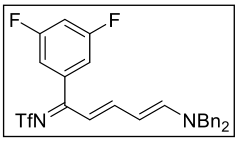

Red solid (0.44 mmol, 229 mg, 44%); **^{1}H NMR** (400 MHz, $CDCl_3$) δ 7.39 (q, *J* = 9.8, 8.2 Hz, 8H), 7.16 (s, 4H), 7.13 – 7.07 (m, 2H), 6.92 (tt, *J* = 8.7, 2.4 Hz, 1H), 6.64 (d, *J* = 13.6 Hz, 1H), 5.88 (t, *J* = 12.1 Hz, 1H), 4.49 (s, 2H), 4.45 (s, 2H); **^{13}C NMR** (101 MHz, $CDCl_3$) δ 174.3, 163.8, 163.7, 161.3, 161.2, 160.2, 158.7, 141.2, 141.1, 133.8, 133.3, 129.3, 129.0, 128.6, 127.9, 127.2, 124.2, 121.1, 117.9, 114.7, 113.4, 112.6, 112.6, 112.5, 112.4, 106.3, 106.0, 105.8, 103.4, 77.3, 77.0, 76.7, 60.0, 51.6; **HRMS** (ESI) calcd. for $C_{26}H_{22}F_5N_2O_2S$ [M+H] 521.1322; found 521.1326.

***N*-((1*E*,2*E*,4*E*)-5-(dibenzylamino)-1-(furan-2-yl)penta-2,4-dien-1-ylidene)-1,1,1-trifluoromethanesulfonamide (S22b)**[23]

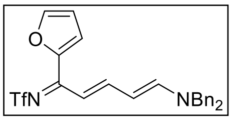

Yellow solid (0.68 mmol, 323 mg, 68%); **^{1}H NMR** (400 MHz, $CDCl_3$) δ 8.12 (t, *J* = 12.9 Hz, 1H), 7.67 – 7.56 (m, 2H), 7.46 – 7.30 (m, 7H), 7.19 (s, 4H), 6.65 (d, *J* = 13.6 Hz, 1H), 6.57 (dd, *J* = 3.7, 1.7 Hz, 1H), 5.95 (t, *J* = 12.1 Hz, 1H), 4.53 (s, 2H), 4.48 (s, 2H); **^{13}C NMR** (126 MHz, $CDCl_3$) δ

160.5, 158.9, 152.6, 146.7, 134.5, 133.9, 133.6, 133.5, 129.8, 128.9, 128.5, 127.9, 127.2, 121.2, 119.0, 118.3, 113.6, 111.8, 104.2, 60.0, 51.4.

***N*-((1*Z*,2*E*,4*E*)-5-(dibenzylamino)-1-(thiophen-2-yl)penta-2,4-dien-1-ylidene)-1,1,1-trifluoromethanesulfonamide (S23b)**

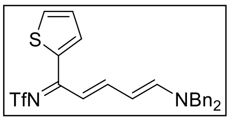

Orange solid (0.40 mmol, 196 mg, 40%); **^{1}H NMR** (400 MHz, $CDCl_3$) δ 7.94 – 7.77 (m, 1H), 7.71 – 7.46 (m, 3H), 7.39 (m, 6H), 7.14 (m, 5H), 6.66 (d, *J* = 13.6 Hz, 1H), 5.88 (t, J = 12.1 Hz, 1H), 4.49 (s, 2H), 4.45 (s, 2H); **^{13}C NMR** (101 MHz, $CDCl_3$) δ 167.3, 159.3, 158.6, 143.2, 134.1, 133.6, 132.8, 132.5, 129.2, 128.9, 128.4, 128.1, 128.0, 127.2, 124.6, 121.4, 118.2, 112.4, 103.5, 59.9, 51.5; **HRMS** (ESI) calcd. for $C_{24}H_{22}F_3N_2O_2S_2$ [M+H] 491.1075; found 491.1076.

***N*-((1*Z*,2*E*,4*E*)-1-(benzo[b]thiophen-2-yl)-5-(dibenzylamino)penta-2,4-dien-1-ylidene)-1,1,1-trifluoromethanesulfonamide (S24b)**

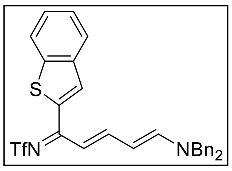

Orange solid (0.10 mmol, 54 mg, 10%); **^{1}H NMR** (600 MHz, $CDCl_3$) δ 7.94 – 7.89 (m, 1H), 7.88 (s, 1H), 7.83 (dd, J = 7.1, 4.9 Hz, 2H), 7.54 (d, J = 12.2 Hz, 1H), 7.44 – 7.35 (m, 8H), 7.18 (d, J = 7.1 Hz, 4H), 6.73 (d, J = 13.5 Hz, 1H), 5.95 (t, J = 12.1 Hz, 1H), 4.50 (s, 2H), 4.46 (s, 2H); **^{13}C NMR** (101 MHz, $CDCl_3$) δ 167.7, 159.3, 158.8, 143.1, 142.3, 139.3, 129.3, 129.2, 129.0, 128.5, 127.9, 127.3, 126.7, 125.2, 124.8, 122.5, 112.7, 103.8, 60.0, 51.5; **HRMS** (ESI) calcd. for $C_{28}H_{24}F_3N_2O_2S_2$ [M+H] 541.1231; found 541.1238.

***N*-((1*Z*,2*E*,4*E*)-1-(benzo[b]thiophen-3-yl)-5-(dibenzylamino)penta-2,4-dien-1-ylidene)-1,1,1-trifluoromethanesulfonamide (S25b)**

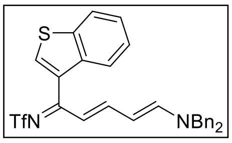

Orange solid (0.61 mmol, 330 mg, 61%); **^{1}H NMR** (500 MHz, $CDCl_3$) δ 8.13 (dd, *J* = 7.3, 1.4 Hz, 1H), 7.86 (d, *J* = 6.9 Hz, 1H), 7.76 (s, 1H), 7.45 – 7.33 (m, 9H), 7.24 (d, *J* = 12.3 Hz, 1H), 7.14 (s, 4H), 6.82 (d, *J* = 13.5 Hz, 1H), 5.83 (t, *J* = 12.2 Hz, 1H), 4.42 (s, 4H); **^{13}C NMR** (126 MHz, $CDCl_3$) δ 172.1, 159.0, 157.4, 140.0, 137.4, 134.2, 133.9, 133.7, 132.1, 129.2, 128.8, 128.4, 127.8, 127.2, 125.2, 125.0, 124.1, 122.4, 118.1, 115.8, 102.3, 59.7, 51.4; **HRMS** (ESI) calcd. for $C_{28}H_{24}F_3N_2O_2S_2$ [M+H] 541.1231; found 541.1238.

***tert*-butyl 2-((1*Z*,2*E*,4*E*)-5-(dibenzylamino)-1-(((trifluoromethyl)sulfonyl)imino)penta-2,4-dien-1-yl)-1H-pyrrole-1-carboxylate (S26b)**

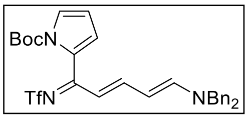

Orange solid (0.47 mmol, 270 mg, 47%); **^{1}H NMR** (500 MHz, $CDCl_3$) δ 7.43 – 7.31 (m, 8H), 7.28 – 7.23 (m, 1H), 7.15 (d, *J* = 7.0 Hz, 4H), 6.56 (d, *J* = 13.6 Hz, 1H), 6.49 (dd, *J* = 3.4, 1.7 Hz, 1H), 6.23 (t, *J* = 3.3 Hz, 1H), 5.75 (t, *J* = 12.2 Hz, 1H), 4.42 (s, 4H), 1.54 (s, 9H); **^{13}C NMR** (126 MHz, $CDCl_3$) δ 170.8, 157.8, 156.6, 148.2, 129.3, 129.2, 127.7, 127.2, 125.0, 120.6, 119.6, 117.1, 110.5, 101.9, 85.2, 30.9, 27.5; **HRMS** (ESI) calcd. for $C_{29}H_{30}F_3N_3O_4S$ [M+H] 574.1987; found 574.1985.

***tert*-butyl 2-((1*Z*,2*E*,4*E*)-5-(dibenzylamino)-1-(((trifluoromethyl)sulfonyl)imino)penta-2,4-dien-1-yl)-1H-indole-1-carboxylate (S27b)**

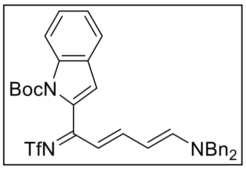

Orange solid (0.55 mmol, 343 mg, 55%); **^{1}H NMR** (400 MHz, $CDCl_3$) δ 8.19 (d, *J* = 7.4 Hz, 1H), 7.59 (d, *J* = 7.7 Hz, 1H), 7.42 – 7.31 (m, 8H), 7.30 – 7.21 (m, 2H), 7.14 (dd, *J* = 7.5, 2.0 Hz, 4H), 6.82 (s, 1H), 6.63 (d, *J* = 13.6 Hz, 1H), 5.78 (t, *J* = 12.2 Hz, 1H), 4.41 (s, 4H), 1.59 (s, 9H); **^{13}C NMR** (101 MHz, $CDCl_3$) δ 171.3, 157.0, 149.1, 137.3, 134.5, 129.2, 128.8, 128.2, 127.8, 127.2, 125.9, 123.2, 121.5, 116.8, 115.2, 113.8, 102.1, 85.1, 27.7; **HRMS** (ESI) calcd. for $C_{33}H_{33}F_3N_3O_4S$ [M+H] 624.2144; found 624.2142.

***N*-((2*E*,3*E*,5*E*)-6-(dibenzylamino)hexa-3,5-dien-2-ylidene)-1,1,1-trifluoromethanesulfonamide (S28b)**

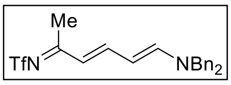

Yellow solid (0.32 mmol, 136 mg, 32%); **^{1}H NMR** (500 MHz, $CDCl_3$) δ 7.64 (s, 1H), 7.38 (d, *J* = 15.9 Hz, 7H), 7.17 (s, 4H), 6.10 (s, 1H), 5.67 (t, *J* = 12.2 Hz, 1H), 4.44 (s, 4H), 2.42 (s, 3H); **^{13}C NMR** (126 MHz, Chloroform-*d*) δ = 180.5, 156.8, 155.3, 129.2, 128.7, 128.4, 127.9, 127.2, 116.4, 101.3, 59.6, 51.4; **HRMS** (ESI) calcd. for $C_{21}H_{22}F_3N_2O_2S$ [M+H] 423.1354; found 423.1353.

***N*-((3*E*,4*E*,6*E*)-7-(dibenzylamino)hepta-4,6-dien-3-ylidene)-1,1,1-trifluoromethanesulfonamide (S29b)**

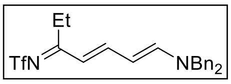

Yellow solid (0.60 mmol, 261 mg, 60%); **^{1}H NMR** (500 MHz, $CDCl_3$) δ 7.68 – 7.53 (m, 1H), 7.36 (d, *J* = 18.8 Hz, 7H), 7.17 (d, *J* = 7.9 Hz, 4H), 6.25 (d, *J* = 13.8 Hz, 1H), 5.68 (t, *J* = 12.1 Hz, 1H), 4.43 (s, 4H), 2.71 (q, *J* = 7.5 Hz, 2H), 1.23 (t, *J* = 7.5 Hz, 3H); **^{13}C NMR** (126 MHz, $CDCl_3$) δ 185.4, 156.9, 155.2, 129.7, 128.8, 128.3, 127.7, 121.1, 118.6, 115.0, 101.8, 60.0, 30.2, 13.1; **HRMS** (ESI) calcd. for $C_{22}H_{24}F_3N_2O_2S$ [M+H] 437.1511; found 437.1501.

***N*-((1*E*,3*E*,5*Z*)-1-(dibenzylamino)-6-oxoundeca-1,3-dien-5-ylidene)-1,1,1-trifluoromethanesulfonamide (S30b)**

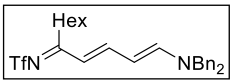

Yellow solid (0.58 mmol, 293 mg, 58%); **^{1}H NMR** (500 MHz, $CDCl_3$) δ 7.60 (t, *J* = 12.8 Hz, 1H), 7.36 (d, *J* = 12.4 Hz, 7H), 7.18 (s, 4H), 6.26 (d, *J* = 13.6 Hz, 1H), 5.67 (t, *J* = 12.1 Hz, 1H), 4.46 (s, 2H), 4.42 (s, 2H), 2.70 – 2.61 (m, 2H), 1.66 (p, *J* = 7.5 Hz, 2H), 1.36 (dt, *J* = 12.6, 7.0 Hz, 2H), 1.30 (d, *J* = 7.1 Hz, 2H), 0.88 (d, J = 13.8 Hz, 3H); **^{13}C NMR** (126 MHz, $CDCl_3$) δ 184.6, 157.0, 155.2, 129.7, 129.2, 128.8, 128.3, 127.7, 115.6, 101.9, 60.0, 51.8, 37.1, 32.0, 29.6, 29.1, 23.0, 14.5; **HRMS** (ESI) calcd. for $C_{26}H_{30}F_3N_2O_2S$ [M+H] 491.1982; found 491.1980.

***N*-((3*E*,4*E*,6*E*)-7-(dibenzylamino)-2-methylhepta-4,6-dien-3-ylidene)-1,1,1-trifluoromethanesulfonamide (S31b)**

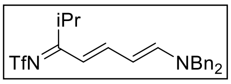

Yellow solid (0.47 mmol, 211 mg, 47%); **^{1}H NMR** (500 MHz, $CDCl_3$) δ 7.65 – 7.53 (m, 1H), 7.47 – 7.29 (m, 7H), 7.16 (s, 4H), 6.36 (d, *J* = 14.0 Hz, 1H), 5.70 (t, *J* = 12.1 Hz, 1H), 4.42 (s, 4H), 3.27 (p, *J* = 6.7 Hz, 1H), 1.18 (d, *J* = 6.6 Hz, 6H); **^{13}C NMR** (126 MHz, $CDCl_3$) δ 188.3, 156.4, 154.2, 134.9, 134.3, 129.4, 128.9, 128.6, 128.0, 127.4, 120.8, 118.3, 113.4, 101.7, 59.7, 51.6, 33.7, 21.6; **HRMS** (ESI) calcd. for $C_{23}H_{26}F_3N_2O_2S$ [M+H] 574.1987; found 574.1985.

***N*-((2*E*,3*E*,5*E*)-6-(dibenzylamino)-1-phenylhexa-3,5-dien-2-ylidene)-1,1,1-trifluoromethanesulfonamide (S32b)**

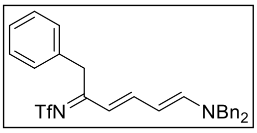

Yellow solid (0.42 mmol, 209 mg, 42%); **^{1}H NMR** (400 MHz, Chloroform-*d*) δ = 7.76 (d, *J*=12.6, 1H), 7.33 (d, *J*=23.0, 12H), 7.11 (s, 4H), 6.16 – 6.14 (m, 1H), 5.66 (t, *J*=12.2, 1H), 4.46 (s, 2H), 4.39 (s, 2H), 4.04 (s, 2H); **^{13}C NMR** (101 MHz, $CDCl_3$) δ 180.6, 157.5, 156.0, 136.7, 129.2, 129.0, 128.8, 128.6, 127.8, 127.2, 126.8, 121.0, 117.9, 114.7, 102.0, 67.0, 59.6, 51.3, 42.9; **HRMS** (ESI) calcd. for $C_{27}H_{26}F_3N_2O_2S$ [M+H] 499.1667; found 499.1670.

***N*-((2*Z*,3*E*,5*E*)-6-(dibenzylamino)-1-methoxy-1-phenylhexa-3,5-dien-2-ylidene)-1,1,1-trifluoromethanesulfonamide (S33b)**

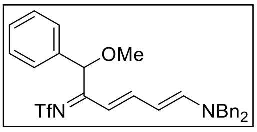

Yellow solid (0.23 mmol, 122 mg, 23%); **^{1}H NMR** (500 MHz, $CDCl_3$) δ 8.05 (dd, *J* = 13.7, 12.2 Hz, 1H), 7.53 – 7.48 (m, 2H), 7.42 (d, *J* = 12.3 Hz, 1H), 7.37 (d, *J* = 13.9 Hz, 6H), 7.33 (t, *J* = 7.5 Hz, 2H), 7.31 – 7.26 (m, 1H), 7.18 – 7.10 (m, 4H), 6.31 (d, *J* = 13.7 Hz, 1H), 5.75 (t, *J* = 12.2 Hz, 1H), 5.37 (s, 1H), 4.46 (s, 2H), 4.39 (s, 2H), 3.46 (s, 3H); **^{13}C NMR** (126 MHz, $CDCl_3$) δ 180.1, 158.5, 157.7, 138.5, 134.0, 133.5, 129.2, 128.9, 128.4, 128.0, 127.8, 127.2, 126.5, 111.0, 103.3, 85.4, 59.8, 57.5, 51.3; **HRMS** (ESI) calcd. for $C_{28}H_{228}F_3N_2O_3S$ [M+H] 529.1773; found 529.1782.

***N*-((1*Z*,2*E*,4*E*)-5-(dibenzylamino)-1-(2-(trifluoromethyl)phenyl)penta-2,4-dien-1-ylidene)-1,1,1-trifluoromethanesulfonamide (S34b)**

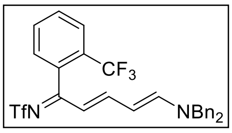

Yellow solid (0.53 mmol, 293 mg, 53%); **^{1}H NMR** (500 MHz, $CDCl_3$) δ 7.73 (d, *J* = 7.7 Hz, 1H), 7.57 (dt, *J* = 25.2, 7.2 Hz, 2H), 7.43 – 7.32 (m, 7H), 7.17 (s, 1H), 7.13 (d, *J* = 7.6 Hz, 4H), 6.75 (s, 2H), 5.81 (t, *J* = 11.8 Hz, 1H), 4.41 (s, 4H); **^{13}C NMR** (126 MHz, $CDCl_3$) δ 179.5, 159.1, 136.7, 136.1, 134.0, 132.6, 131.9, 131.5, 131.2, 130.4, 129.9, 129.2, 123.3, 120.7, 118.5, 105.2, 62.4, 54.0; **HRMS** (ESI) calcd. for $C_{27}H_{23}F_6N_2O_2S$ [M+H] 553.1384; found 553.1386.

***N*-((1*Z*,2*E*,4*E*)-5-(dibenzylamino)-1-(4-(dimethylamino)phenyl)penta-2,4-dien-1-ylidene)-1,1,1-trifluoromethanesulfonamide (S35b)**

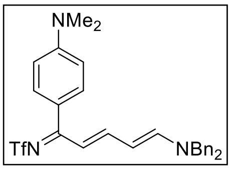

Red solid (0.20 mmol, 105 mg, 20%); **^{1}H NMR** (500 MHz, $CDCl_3$) δ 7.68 (d, *J* = 9.1 Hz, 2H), 7.50 (dd, *J* = 13.8, 11.8 Hz, 1H), 7.43 – 7.31 (m, 6H), 7.28 (d, *J* = 12.4 Hz, 1H), 7.16 (d, *J* = 6.5 Hz, 4H), 6.68 – 6.61 (m, 3H), 5.77 (t, *J* = 12.1 Hz, 1H), 4.41 (s, 4H), 3.06 (s, 6H); **^{13}C NMR** (126 MHz, $CDCl_3$) δ 179.3, 160.0, 158.5, 156.0, 135.1, 131.8, 131.0, 127.6, 123.8, 117.0, 113.5, 104.5, 42.7; **HRMS** (ESI) calcd. for $C_{27}H_{23}F_3N_3O_2S$ [M+H] 528.1933; found 528.1929.

(1*Z*,2*E*,4*E*)-5-(dibenzylamino)-*N*,*N*-diphenyl-*N'*-((trifluoromethyl)sulfonyl)penta-2,4-dienimidamide (S36b)

Yellow solid (0.11 mmol, 63 mg, 11%); **^{1}H NMR** (500 MHz, $CDCl_3$) δ 7.80 (dd, *J* = 14.1, 11.6 Hz, 1H), 7.38 – 7.27 (m, 12H), 7.25 – 7.20 (m, 5H), 7.13 (d, *J* = 7.2 Hz, 4H), 5.50 (d, *J* = 14.1 Hz, 1H), 5.38 (t, *J* = 12.1 Hz, 1H), 4.31 (s, 4H); **^{13}C NMR** (126 MHz, $CDCl_3$) δ 165.8, 154.6, 143.4, 129.3, 128.9, 127.5, 127.4, 107.8, 99.4; **HRMS** (ESI) calcd. for $C_{38}H_{31}F_3N_3O_2S$ [M+H] 576.1933; found 576.1926.

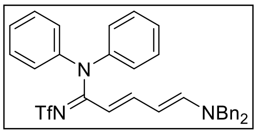

N-((1E,2Z,4E)-2-bromo-5-(dibenzylamino)-1-phenylpenta-2,4-dien-1-ylidene)-1,1,1-trifluoromethanesulfonamide (S37b)[23]

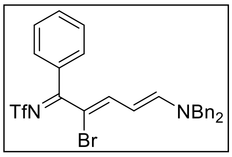

Yellow solid (0.30 mmol, 169 mg, 30%); **^{1}H NMR** (500 MHz, $CDCl_3$) δ 7.54 – 7.33 (m, 10H), 7.26 (s, 1H), 7.22 – 7.16 (m, 3H), 7.17 (s, 1H), 7.15 – 7.10 (m, 2H), 6.15 (t, *J* = 12.0 Hz, 1H), 4.46 (s, 2H), 4.42 (s, 2H); **^{13}C NMR** (126 MHz, $CDCl_3$) δ 176.4, 157.7, 156.7, 136.3, 133.9, 133.5, 130.5, 129.3, 129.2, 128.9, 128.6, 128.5, 128.2, 127.7, 127.6, 110.5, 103.2, 60.3, 59.7, 51.4, 21.0.

N-((1E,2Z,4E)-5-(dibenzylamino)-3-phenylpenta-2,4-dien-1-ylidene)-1,1,1-trifluoromethanesulfonamide (S38b)[24]

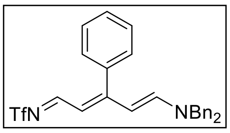

Yellow solid (0.10 mmol 48 mg, 10%); **^{1}H NMR** (400 MHz, $CDCl_3$) δ 8.00 (d, *J* = 11.2 Hz, 1H), 7.51 – 7.34 (m, 8H), 7.25 (s, 2H), 7.21 – 7.16 (m, 3H), 7.05 (s, 3H), 6.33 (d, *J* = 11.3 Hz, 1H), 5.96 (d, *J* = 12.6 Hz, 1H), 4.51 (s, 2H), 4.34 (s, 2H).

N-((1E,2E,4E)-5-(dibenzylamino)-2-phenylpenta-2,4-dien-1-ylidene)-1,1,1-trifluoromethanesulfonamide (S39b)[1]

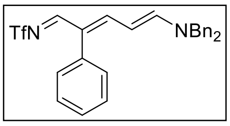

Yellow solid (0.80 mmol 387 mg, 80%); **^{1}H NMR** (400 MHz, $CDCl_3$) δ 8.39 (s, 1H), 7.52 (d, *J* = 12.2 Hz, 1H), 7.46 – 7.26 (m, 13H), 7.25 – 7.12 (m, 6H), 7.05 (s, 3H), 5.80 (t, *J* = 12.3 Hz, 1H), 4.53 (s, 3H), 4.30 (s, 3H).

N-((1E,2E,4E)-5-(dibenzylamino)penta-2,4-dien-1-ylidene)-1,1,1-trifluoromethanesulfonamide (S40b)[24]

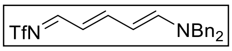

Yellow solid (0.50 mmol, 204 mg, 50%); **^{1}H NMR** (500 MHz, $CDCl_3$) δ 8.31 (d, *J* = 10.9 Hz, 1H), 7.54 (d, *J* = 12.2 Hz, 1H), 7.48 (d, *J* = 12.9 Hz, 1H), 7.46 – 7.34 (m, 6H), 7.18 (dd, *J* = 18.2, 7.2 Hz, 4H), 6.19 (dd, *J* = 13.4, 11.0 Hz, 1H), 5.87 (t, *J* = 12.2 Hz, 1H), 4.53 (s, 2H), 4.48 (s, 2H); **^{13}C NMR** (126 MHz, $CDCl_3$) δ 174.9, 165.8, 159.0, 133.7, 133.3, 129.3, 129.3, 129.0, 128.5, 128.1, 127.2, 121.1, 118.5, 115.8, 103.0, 59.9, 51.6.

N-((1E,2E,4E)-5-(dibenzylamino)-2-methylpenta-2,4-dien-1-ylidene)-1,1,1-trifluoromethanesulfonamide (S41b)

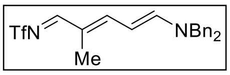

Yellow solid (0.60 mmol 253 mg, 60%); **^{1}H NMR** (500 MHz, $CDCl_3$) δ = 8.22 (s, 1H), 7.48 (d, *J* = 12.2, 1H), 7.39 (d, *J* = 11.3, 6H), 7.28 (d, *J* = 12.3, 1H), 7.19 (t, *J* = 8.4, 4H), 5.81 (t, *J* = 12.3, 1H), 4.53 (s, 2H), 4.49 (s, 2H), 1.86 (s, 3H); **^{13}C NMR** (126 MHz, $CDCl_3$) δ 175.4, 164.4, 157.8, 134.0, 133.6, 129.3, 129.2, 129.0, 128.5, 128.0, 127.3, 122.8, 121.1, 118.6, 100.1, 59.9, 51.7, 10.5; **HRMS** (ESI) calcd. for $C_{21}H_{22}F_3N_2O_3S$ [M+H] 423.1354; found 423.1356. **^{1}H NMR** (500 MHz, Chloroform-*d*) δ = 8.22 (s, 1H), 7.48 (d, *J*=12.2, 1H), 7.39 (d, *J*=11.3, 6H), 7.28 (d, *J*=12.3, 1H), 7.19 (t, *J*=8.4, 4H), 5.81 (t, *J*=12.3, 1H), 4.51 (d, *J*=17.9, 4H), 1.86 (s, 3H).

N-((1Z,2E,4Z)-5-(dibenzylamino)-1,4-diphenylpenta-2,4-dien-1-ylidene)-1,1,1-trifluoromethanesulfonamide (S42b)

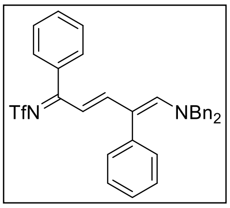

Yellow solid (0.18 mmol, 101 mg, 18%); **^{1}H NMR** (400 MHz, $CDCl_3$) δ 7.68 (d, *J* = 6.9 Hz, 2H), 7.61 (d, *J* = 11.3 Hz, 1H), 7.49 (t, *J* = 7.3 Hz, 4H), 7.33 (d, *J* = 17.5 Hz, 8H), 7.22 (s, 6H), 7.08 (m, 6H), 6.83 (d, *J* = 6.3 Hz, 1H), 6.48 (d, *J* = 6.1 Hz, 1H), 6.14 (s, 1H), 4.20 (s, 2H), 3.96 (s, 2H); **^{13}C NMR** (101 MHz, $CDCl_3$) δ 136.7, 136.5, 135.5, 134.7, 131.2, 130.3, 129.4, 129.4, 129.0, 128.8, 128.6, 128.4, 128.4, 128.3, 128.2, 128.1, 127.3, 127.1, 126.4, 121.3, 119.1, 113.5, 51.1, 30.8. **HRMS** (ESI) calcd. for $C_{32}H_{28}F_3N_2O_3S$ [M+H] 561.1824; found 561.1820.

methyl (1*Z*,2*E*,4*E*)-5-(dibenzylamino)-N-((trifluoromethyl)sulfonyl)penta-2,4-dienimidate (S43b)[25]

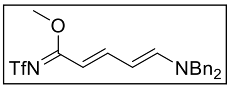

Yellow solid (0.29 mmol, 127 mg, 29%); **^{1}H NMR** (500 MHz, $CDCl_3$) δ 7.57 (dd, *J* = 14.0, 11.9 Hz, 1H), 7.42 – 7.30 (m, 6H), 7.17 (d, *J* = 3.1 Hz, 5H), 6.10 (d, *J* = 14.0 Hz, 1H), 5.57 (t, *J* = 12.2 Hz, 1H), 4.37 (s, 4H), 3.86 (s, 3H); **^{13}C NMR** (126 MHz, $CDCl_3$) δ 171.2, 154.5, 152.7, 129.1, 128.3, 127.4, 103.4, 99.4, 55.4; **HRMS** (ESI) calcd. for $C_{21}H_{21}F_3N_2O_3S$ [M+H] 461.1123; found 461.1122.

***N*-((1*Z*,2*Z*)-2-((*E*)-3-(dibenzylamino)allylidene)cyclohexylidene)-1,1,1-trifluoromethanesulfonamide (S44b)**[1]

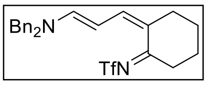

Yellow solid (0.48 mmol, 222 mg, 48%); **^{1}H NMR** (500 MHz, $CDCl_3$) δ 8.21 (d, *J* = 12.7 Hz, 1H), 7.52 (d, *J* = 12.1 Hz, 1H), 7.41 (s, 6H), 7.18 (s, 4H), 5.60 (d, *J* = 12.4 Hz, 1H), 4.50 (s, 2H), 4.45 (s, 2H), 3.01 (t, *J* = 6.2 Hz, 2H), 2.29 (d, *J* = 6.6 Hz, 2H), 1.78 – 1.59 (m, 4H); **^{13}C NMR** (126 MHz, $CDCl_3$) δ 182.1, 158.6, 152.5, 129.2, 128.8, 128.4, 127.9, 127.2, 120.2, 99.2, 59.9, 51.3, 34.2, 25.1, 22.0, 21.9.

***N*-((1*Z*,2*E*,4*E*)-5-(dibenzylamino)-1-(trimethylsilyl)penta-2,4-dien-1-ylidene)-1,1,1-trifluoromethanesulfonamide (S45b)**

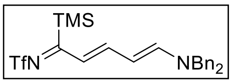

Yellow solid (0.56 mmol, 269 mg, 56%); **^{1}H NMR** (400 MHz, $CDCl_3$) δ 7.57 – 7.46 (m, 1H), 7.39 (s, 6H), 7.32 (d, *J* = 12.4 Hz, 1H), 7.18 (d, *J* = 7.4 Hz, 4H), 6.73 (d, *J* = 14.0 Hz, 1H), 5.73 (t, *J* = 12.1 Hz, 1H), 4.47 (s, 4H), 0.34 (s, 9H); **^{13}C NMR** (126 MHz, $CDCl_3$) δ 197.1, 156.8, 156.4, 129.2, 128.7, 128.3, 127.7, 127.2, 121.7, 120.0, 102.2, 60.2 51.2, 0.0; **HRMS** (ESI) calcd. for $C_{23}H_{28}F_3N_2O_2SSi$ [M+H] 481.1593; found 481.1594.

***N*-((1*Z*,2*E*,4*E*)-5-(dibenzylamino)-1-(1,3-dioxolan-2-yl)penta-2,4-dien-1-ylidene)-1,1,1-trifluoromethanesulfonamide (S46b)**[25]

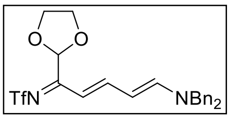

Yellow solid (0.14 mmol, 67 mg, 14%); **^{1}H NMR** (400 MHz, $CDCl_3$) δ 7.89 (t, *J* = 12.9 Hz, 1H), 7.55 – 7.34 (m, 7H), 7.14 (s, 4H), 6.35 (d, *J* = 13.8 Hz, 1H), 5.93 (s, 1H), 5.82 (t, *J* = 12.1 Hz, 1H), 4.51 (s, 2H), 4.46 (s, 2H), 4.20 – 3.96 (m, 4H).

***N*-((1*Z*,2*Z*)-2-((*E*)-3-(dibenzylamino)allylidene)cyclopentylidene)-1,1,1-trifluoromethanesulfonamide (S47b)**

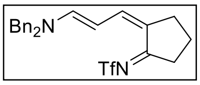

Yellow solid (0.40 mmol, 179 mg, 40%); **^{1}H NMR** (500 MHz, $CDCl_3$) δ 7.87 (d, *J* = 12.6 Hz, 1H), 7.47 (d, *J* = 12.2 Hz, 1H), 7.39 (s, 6H), 7.18 (d, *J* = 6.4 Hz, 4H), 5.51 (t, *J* = 12.4 Hz, 1H), 4.46 (s, 4H), 3.03 (t, *J* = 7.7 Hz, 2H), 2.48 – 2.37 (m, 2H), 1.94 (p, *J* = 7.6 Hz, 2H); **^{13}C NMR** (126 MHz, $CDCl_3$) δ 189.5, 157.8, 147.3, 134.3, 133.9, 129.2, 128.9, 128.3, 128.3, 127.9, 127.2, 125.7, 121.2, 118.6, 100.1, 59.7, 51.5, 37.1, 27.4, 21.7; **HRMS** (ESI) calcd. for $C_{23}H_{24}F_3N_2O_2S$ [M+H] 449.1511; found 449.1516.

***N*-((1*E*,2*Z*,4*E*)-2-bromo-5-(dibenzylamino)penta-2,4-dien-1-ylidene)-1,1,1-trifluoromethanesulfonamide (S48b)**[25]

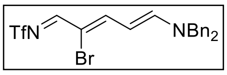

Red solid (0.45 mmol, 219 mg, 45%); **^{1}H NMR** (500 MHz, $CDCl_3$) δ 8.30 (s, 1H), 7.74 (s, 1H), 7.57 (d, *J* = 12.0 Hz, 1H), 7.48 – 7.35 (m, 6H), 7.22 (d, *J* = 7.1 Hz, 4H), 6.24 (t, *J* = 12.0 Hz, 1H), 4.59 (s, 2H), 4.57 (s, 2H); **^{13}C NMR** (126 MHz, $CDCl_3$) δ 169.6, 161.8, 160.3, 133.0, 132.8, 129.4, 129.4, 129.3, 128.9, 128.3, 127.6, 106.1, 103.8, 60.3, 52.2.

1,1,1-trifluoro-*N*-((1Z,2E,4E)-5-morpholino-1-phenylpenta-2,4-dien-1-ylidene)methanesulfonamide (S49b)[1]

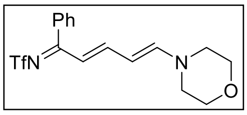

Red solid (0.30 mmol, 30%) **^{1}H NMR** (600 MHz, $CDCl_3$) δ 7.59 – 7.54 (m, 2H), 7.51 – 7.46 (m, 1H), 7.44 – 7.39 (m, 2H), 7.29 (dd, *J* = 13.7, 12.0 Hz, 1H), 6.96 (d, *J* = 12.2 Hz, 1H), 6.69 (d, *J* = 13.6 Hz, 1H), 5.69 (t, *J* = 12.2 Hz, 1H), 3.80 – 3.68 (m, 4H), 3.42 (t, J = 4.9 Hz, 4H).

***N*-((1Z,2E,4E)-5-(benzyl(phenyl)amino)-1-phenylpenta-2,4-dien-1-ylidene)-1,1,1-trifluoromethanesulfonamide (S50b)**[1]

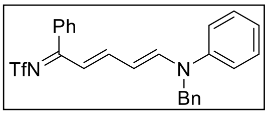

Red solid (0.70 mmol, 70%) **^{1}H NMR** (400 MHz, $CDCl_3$) δ 7.61 – 7.55 (m, 2H), 7.54 – 7.48 (m, 1H), 7.44 (dt, *J* = 7.9, 1.3 Hz, 2H), 7.37 (dddd, *J* = 13.6, 8.4, 6.5, 4.8 Hz, 6H), 7.27 – 7.23 (m, 3H), 7.22 – 7.18 (m, 2H), 7.18 – 7.12 (m, 2H), 6.70 (d, *J* = 13.9 Hz, 1H), 5.85 – 5.65 (m, 1H), 4.97 (d, *J* = 2.3 Hz, 2H).

3.3. Preparation of pyridinium salts

3.3.1. Preparation of *N*,4-dimethylbenzenesulfonohydrazide (from *N*-methylhydrazine)

To an ice-cold stirred solution of tosyl chloride (0.04 mol, 7.62 g) in THF (50 ml) *N*-methylhydrazine (4.4 ml, 0.08 mol) was added dropwise while the temperature was maintained between 0 and 5 ° C. After that, the mixture was stirred an additional 4 h. The solvent was evaporated under reduced pressure and 100 ml of cold water was added. The white solid was filtered, washed with water, and dried under high vacuum, producing the product as a white solid (26 mmol, 5.28 g, 66%).

3.3.2. Preparation of *N*,4-dimethylbenzenesulfonohydrazide (from *N*-methylhydrazine sulphate)

To an ice-cold stirred solution of tosyl chloride (0.04 mol, 7.62 g,) in THF/H_2O (50 ml + 50 ml) *N*- methylhydrazine sulphate (11.5 g, 0.08 mol) together with TEA (22.3 ml, 0.016 mol) were added while the temperature was maintained between 0 and 5 °C. After that, the mixture was stirred an additional 4 h. The solvent was evaporated under reduced pressure and 100 ml of cold water was added. The white solid was filtered, washed with water and dried under high vacuum, giving the product as a white solid (24.8 mmol, 4.96 g, 62%).

3.3.3. Preparation of *N*-Aminopirydinium salt (Py-salt)[2]

2,4,6-Trimethylpyrylium tetrafluoroborate (4.0 mmol, 1.0 equiv) was suspended in absolute EtOH (10 ml). Subsequently, *N*,4-dimethylbenzenesulfonohydrazide (4.8 mmol, 1.2 equiv) was added and the reaction mixture was stirred at room temperature for 16 h. After this time, Et_2O (20 ml) was added to the mixture. The resulting precipitate was filtered off, washed with Et_2O, and dried under vacuum. The crude salt was recrystallised from EtOH/Et_2O mixture to produce an off white solid (2.8 mmol, 1.08 g, 69% yield). **^{1}H NMR** (400 MHz, DMSO-d_6) δ 7.99 – 7.82 (m, 4H), 7.57 (d, J = 8.0 Hz, 2H), 3.57 (s, 3H), 2.47 – 2.58 (m, 12H).

3.4 Experiments with DMPO and TEMPO radical traps

3.4.1 DMPO radical trap

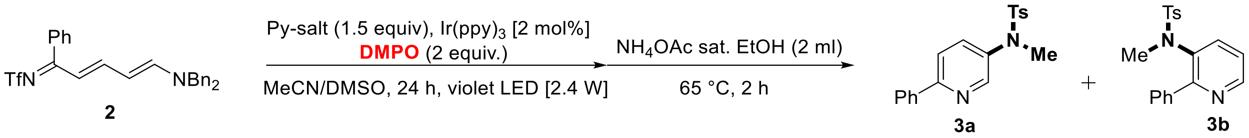

The reaction was set up following the general model reaction procedure (section 5.1) on a 0.05 mmol scale with the addition of DMPO (0.1 mmol, 2.0 equiv.) before irradiation. The reaction was irradiated for 24 hours and after that time, the crude reaction mixture was analysed by ESI MS.

The MS analysis of the crude reaction mixture revealed the presence of a peak at 297.13 m/z, corresponding to the DMPO amidyl radical adduct:

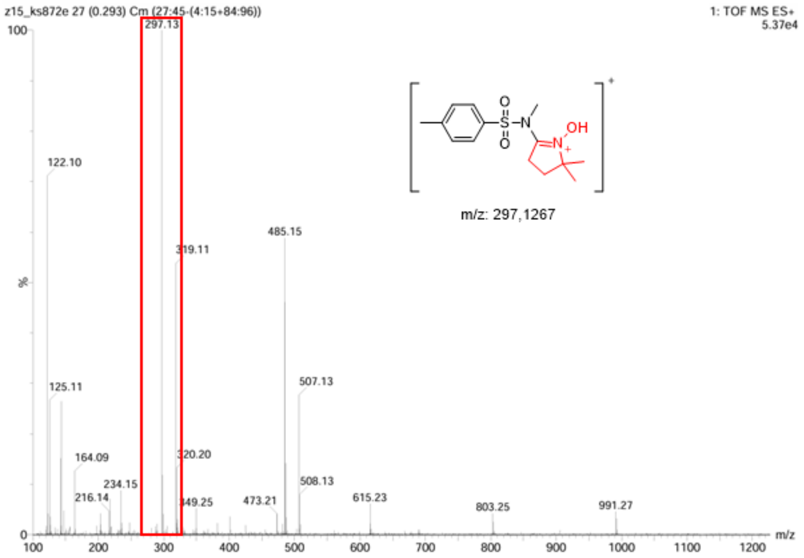

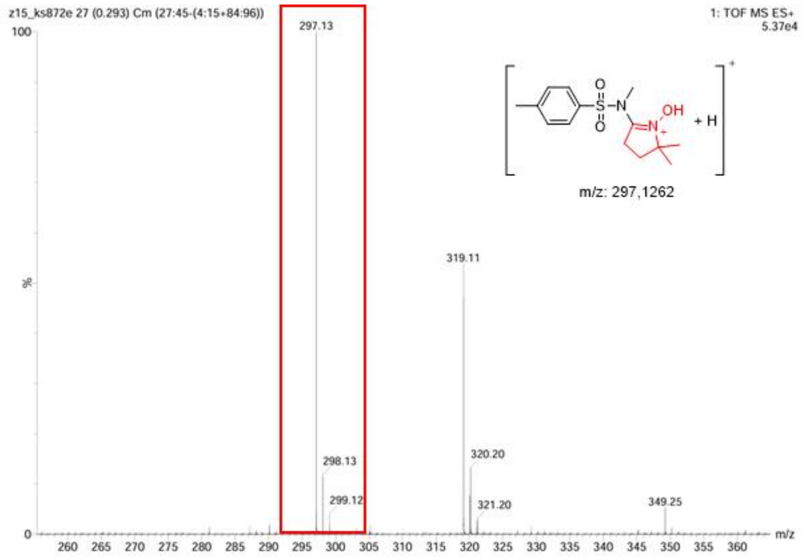

Conclusions No product formation was observed when DMPO was added prior irradiation. The result proves that the reaction is radical in nature. The presence of a peak in ESI MS spectra that corresponds to the adduct corroborates the formation of an amidyl radical.

3.4.2. TEMPO radical trap

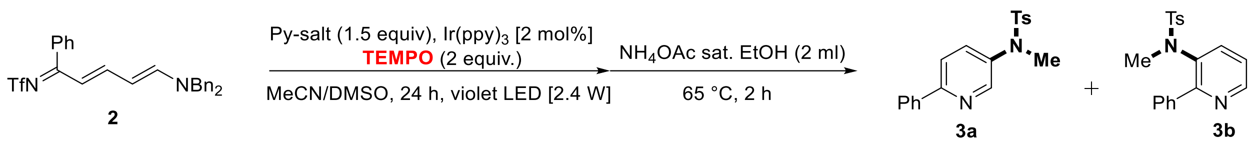

The reaction was set up following the general model reaction procedure (section 5.1) on a 0.05 mmol scale with the addition of TEMPO (0.1 mmol, 2.0 equiv.) before just irradiation or after 2 hours of irradiation of the reaction mixture. The irradiation in both experiments was maintained for 24 hours and after that time, crude reaction mixtures were analysed by ESI MS, TLC and ^{1}H NMR.

Conclusion: No product formation was observed when TEMPO was added prior to irradiation. TLC shows no conversion of the substrates and proves that the reaction is radical in nature. Furthermore, the crude ^{1}H NMR shows no conversion of the substrate.

4. Photocatalysed functionalization of Zincke imines

4.1. Isolation and characterization of reaction intermediate 2a/2b

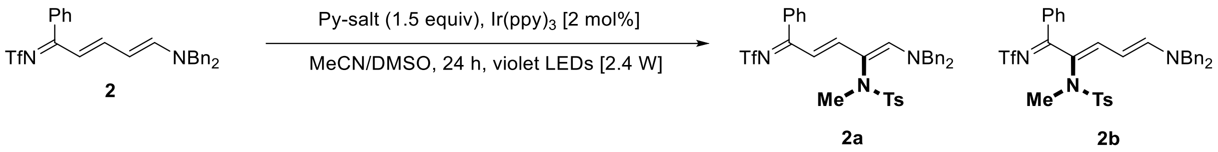

***N*-((1Z,3E,5Z)-1-(dibenzylamino)-5-phenyl-5-(((trifluoromethyl)sulfonyl)imino)penta-1,3-dien-2-yl)-*N*,4-dimethylbenzenesulfonamide (2c-*int*)**

(6 Reactions were set-up, each in 10 ml closed-cup vials) Zincke imine **2** (0.05 mmol), **Py-salt** (0.075 mmol), Ir(ppy)$_3$ (0.67 mg, ~10 μmol, 2 mol%) were placed in the closed-cup vial and MeCN (4 ml) and DMSO (4 ml) were added through the septum. The reaction mixture was placed in ultrasound bath and degassed by bubbling argon through the solution for 15 min. The vial was then placed in the photoreactor and irradiated with violet light (2.4 W) for 24 h, maintaining a temperature between 0 ° C and 5 °C. DMSO and excess of NH_4OAc were removed by extraction (AcOEt/ H_2O). The reaction mixtures were combined, and the organic phase was dried over anhydrous sodium sulphate and concentrated under reduced pressure. **2a** was isolated in a series of 2 chromatography in a mixture (80:10:10) as an orange-yellow amorphous solid; **^{1}H NMR** (500 MHz, $CDCl_3$) δ 7.77 (d, J = 7.9 Hz, 2H), 7.54 – 6.85 (m, 18H), 5.69 (s, 1H), 5.44 (s, 1H), 4.48 (d, J = 15.4 Hz, 4H), 2.86 (s, 3H), 2.35 (s, 3H); **^{13}C NMR** (126 MHz, $CDCl_3$) δ 178.0, 156.5, 156.1, 144.9, 137.5, 135.7, 134.9, 134.1, 131.5, 130.0, 129.3, 129.2, 128.6, 128.3, 127.5, 127.3, 126.9, 120.7, 118.1, 113.4, 111.3, 62.3, 51.7, 36.7, 30.9, 21.5; **HRMS** (ESI) calcd. for $C_{34}H_{33}N_3O_4S_2$ [M+H] 668.1865; found 668.1863. Single crystal was obtained by diffusing *n*-hexane to the solution of **2a** in HPLC grade DCM. The analytical sample (15 mg) of the isolated functionalized Zincke imine was subjected to a ring closure procedure with saturated NH_4OAc (1 ml) and heated up to 65 °C for 2 h yielding quantitatively (based on GC-FID) only a single isomer of closed, functionalized pyridines (without traces of parent pyridine (**1** or minor isomer **3b**).

4.2. General Protocol for photoamination and closure of the Zincke imine (General Procedure 3)

General Procedure 3. Zincke imine (0.05 mmol), **Py-salt** (0.075 mmol), $Ir(ppy)_3$ (0.67 mg, ~10 µmol, 2 mol%) were placed in the closed-cup vial and MeCN (4 ml) and DMSO (4 ml) were added through the septum. The reaction mixture was placed in ultrasound bath and degassed by bubbling argon through the solution for 15 min. The vial was then moved to the photoreactor and irradiated with violet light (2.4 W) for 24 h maintaining temperature between 0 °C to 5 °C with a dedicated cooling system. After the indicated time, a saturated NH_4OAc solution was added in anhydrous ethanol (2 ml) and reaction was heated up to 65 ° C for 2 h. DMSO and an excess of NH_4OAc were removed by extraction (AcOEt/ H_2O). The organic phase was dried over anhydrous sodium sulphate and evaporated with silica gel (dry load for the preparation of the sample for flash chromatography). The pure products were isolated by flash chromatography in the hexanes/AcOEt gradient.

Flash program: time: 25 min; column: silica 4 g; flow rate: 13 mL/min; automatic peak hold: on.

entry	time [min]	hexanes [%]	AcOEt [%]
1	0	95	5
2	25	75	25

Note:

- *Several products, especially minor regioisomers, were initially isolated by column or flash chromatography in a hexanes/AcOEt gradient, and due to some impurities from tailing N-methyltosylamide, further purification by semipreparative normal phase HPLC was necessary to obtain pure compounds.*

_N_,4-dimethyl-_N_-(6-phenylpyridin-3-yl)benzenesulfonamide (3a)

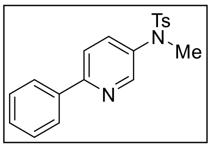

White solid (0.079 mmol, 26.6 mg, 79% yield); **^{1}H NMR** (600 MHz, $CDCl_3$) δ 8.33 (dd, *J* = 2.6, 0.8 Hz, 1H), 8.00 – 7.95 (m, 2H), 7.70 (dd, *J* = 8.5, 0.8 Hz, 1H), 7.62 (dd, *J* = 8.5, 2.6 Hz, 1H), 7.47 (t, *J* = 7.9 Hz, 4H), 7.44 – 7.40 (m, 1H), 7.27 (s, 2H), 3.23 (s, 3H), 2.42 (s, 3H); **^{13}C NMR** (151 MHz, $CDCl_3$) δ 155.9, 146.8, 144.2, 138.4, 136.9, 135.1, 133.2, 129.8, 129.3, 128.9, 127.9, 127.0, 120.2, 37.9, 21.6; **HRMS** (ESI) calcd. for $C_{19}H_{19}N_2O_2S$ [M+H] 339.1167; found 339.1174.

_N_,4-dimethyl-_N_-(2-phenylpyridin-3-yl)benzenesulfonamide (3b)

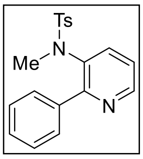

White solid (0.016 mmol, 5.5 mg, 16% yield); **^{1}H NMR** (500 MHz, $CDCl_3$) δ 8.64 (dd, *J* = 4.7, 1.6 Hz, 1H), 7.65 – 7.57 (m, 2H), 7.48 – 7.43 (m, 3H), 7.42 – 7.38 (m, 3H), 7.23 (d, *J* = 8.2 Hz, 3H), 3.04 (s, 3H), 2.43 (s, 3H); **^{13}C NMR** (126 MHz, $CDCl_3$) δ 158.9, 149.1, 144.0, 138.5, 136.2, 136.2, 135.2, 129.7, 128.9, 128.6, 128.3, 128.1, 122.7, 38.9, 21.6; **HRMS** (ESI) calcd. for $C_{19}H_{19}N_2O_2S$ [M+H] 339.1167; found 339.1162.

_N_,_N_'-(2-phenylpyridine-3,5-diyl)bis(N,4-dimethylbenzenesulfonamide) (3c)

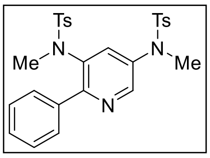

Off-white solid (side product – isolated by combining optimisation reaction mixtures); **^{1}H NMR** (600 MHz, $CDCl_3$) δ 8.27 (d, *J* = 2.4 Hz, 1H), 7.67 (dd, *J* = 6.8, 2.9 Hz, 2H), 7.49 (t, *J* = 8.1 Hz, 4H), 7.44 – 7.41 (m, 3H), 7.36 (d, *J* = 2.4 Hz, 1H), 7.30 (s, 2H), 7.28 – 7.24 (m, 2H), 3.17 (s, 3H), 2.98 (s, 3H), 2.44 (s, 3H), 2.43 (s, 3H); **^{13}C NMR** (151 MHz, $CDCl_3$) δ 157.0, 145.7, 144.5,

144.2, 137.7, 137.2, 136.0, 135.2, 134.2, 132.9, 129.9, 129.9, 129.0, 128.9, 128.4, 128.1, 127.9, 38.6, 37.8, 21.7; **HRMS** (ESI) calcd. for $C_{27}H_{28}N_3O_4S_2$ [M+H] 522.1521; found 522.1520.

***N*-(6-(4-methoxyphenyl)pyridin-3-yl)-*N*,4-dimethylbenzenesulfonamide (4a)**

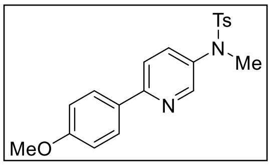

Purified by semi-preparative HPLC in hexanes/AcOEt gradient; white solid (0.071 mmol, 24.0 mg, 71% yield); **^{1}H NMR** (600 MHz, $CDCl_3$) δ 8.27 (d, *J* = 2.5 Hz, 1H), 7.96 – 7.91 (m, 2H), 7.64 (d, *J* = 8.5 Hz, 1H), 7.58 (dd, *J* = 8.5, 2.6 Hz, 1H), 7.47 (d, *J* = 8.2 Hz, 2H), 7.27 (d, *J* = 7.3 Hz, 2H), 7.02 – 6.97 (m, 2H), 3.87 (s, 3H), 3.22 (s, 3H), 2.42 (s, 3H); **^{13}C NMR** (151 MHz, $CDCl_3$) δ 160.6, 155.5, 146.6, 143.9, 136.1, 135.0, 133.1, 130.9, 129.6, 128.2, 127.7, 119.3, 114.1, 55.3, 37.8, 21.5; **HRMS** (ESI) calcd. for $C_{20}H_{21}N_2O_3S$ [M+H] 369.1273; found 369.1278.

***N*-(2-(4-methoxyphenyl)pyridin-3-yl)-N,4-dimethylbenzenesulfonamide (4b)**

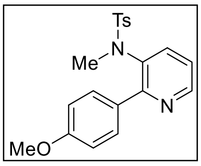

Purified by semi-preparative HPLC in hexanes/ AcOEt gradient; white solid (0.019 mmol, 6.5 mg, 19% yield); **^{1}H NMR** (500 MHz, $CDCl_3$) δ 8.62 (dd, *J* = 4.7, 1.6 Hz, 1H), 7.63 (d, *J* = 8.8 Hz, 2H), 7.51 (d, *J* = 8.3 Hz, 2H), 7.37 (dd, *J* = 8.0, 1.6 Hz, 1H), 7.25 (2H), 7.20 – 7.15 (m, 1H), 6.93 (d, *J* = 8.8 Hz, 2H), 3.86 (s, 3H), 3.06 (s, 3H), 2.44 (s, 3H); **^{13}C NMR** (126 MHz, $CDCl_3$) δ 159.9, 158.2, 148.9, 143.8, 136.0, 135.8, 135.3, 130.7, 130.2, 129.5, 127.9, 122.0, 113.6, 55.2, 38.7, 21.5.

***N*-(6-(3-methoxyphenyl)pyridin-3-yl)-*N*,4-dimethylbenzenesulfonamide (5a)**

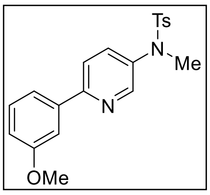

Purified by semi-preparative HPLC in hexanes/AcOEt gradient; white solid (0.060 mmol, 20.5 mg, 60% yield, ~5% MeNHTs); **^{1}H NMR** (500 MHz, $CDCl_3$) δ 8.33 (d, *J* = 1.9 Hz, 1H), 7.69 (d, *J* = 7.7 Hz, 1H), 7.61 (dd, *J* = 8.5, 2.6 Hz, 1H), 7.56 (d, *J* = 2.6 Hz, 1H), 7.52 (d, *J* = 8.2 Hz, 1H), 7.46 (d, *J* = 8.4 Hz, 2H), 7.38 (t, *J* = 7.9 Hz, 1H), 7.27 (d, *J* = 7.5 Hz, 2H), 6.97 (dd, *J* = 8.2, 2.6 Hz, 1H), 3.89 (s, 3H), 3.23 (s, 3H), 2.42 (s, 3H); **^{13}C NMR** (126 MHz, $CDCl_3$) δ 160.1, 155.5, 146.6, 144.0, 139.8, 136.9, 134.9, 133.0, 129.8, 129.7, 129.6, 127.7, 127.2, 120.3, 119.2, 115.3, 112.0, 55.3, 37.7, 21.5; **HRMS** (ESI) calcd. for $C_{20}H_{21}N_2O_3S$ [M+H] 369.1273; found 369.1271.

***N*-(2-(3-methoxyphenyl)pyridin-3-yl)-*N*,4-dimethylbenzenesulfonamide (5b)**

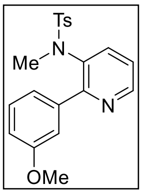

Purified by semi-preparative HPLC in hexanes/AcOEt gradient; white solid (0.021 mmol 7.1 mg, 21% yield); **^{1}H NMR** (500 MHz, $CDCl_3$) δ 8.64 (dd, *J* = 4.7, 1.6 Hz, 1H), 7.50 (d, *J* = 8.3 Hz, 2H), 7.46 – 7.42 (m, 1H), 7.30 (t, *J* = 7.9 Hz, 1H), 7.27 – 7.21 (m, 4H), 7.18 (d, *J* = 7.6 Hz, 1H), 6.96 (dd, *J* = 7.2, 2.7 Hz, 1H), 3.85 (s, 3H), 3.04 (s, 3H), 2.44 (s, 3H); **^{13}C NMR** (126 MHz, $CDCl_3$) δ 159.4, 158.5, 148.9, 143.8, 139.5, 136.1, 136.1, 135.4, 129.6, 129.1, 127.8, 122.6, 121.2, 114.9, 113.9, 55.3, 38.7, 21.5.

_N_-(6-(3,5-dimethoxyphenyl)pyridin-3-yl)-_N_,4-dimethylbenzenesulfonamide (6a)

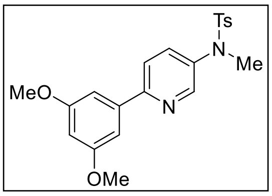

Purified by semi-preparative HPLC in hexanes/AcOEt gradient; off-white oil (0.069 mmol, 23.4 mg, 69% yield); **^{1}H NMR** (500 MHz, $CDCl_3$) δ 8.33 (d, *J* = 2.6 Hz, 1H), 7.67 (d, *J* = 8.6 Hz, 1H), 7.60 (dd, *J* = 8.5, 2.6 Hz, 1H), 7.46 (d, *J* = 8.3 Hz, 2H), 7.30 – 7.23 (m, 2H), 7.13 (d, *J* = 2.3 Hz, 2H), 6.54 (t, *J* = 2.3 Hz, 1H), 3.86 (s, 6H), 3.22 (s, 3H), 2.42 (s, 3H); **^{13}C NMR** (126 MHz, $CDCl_3$) δ 161.1, 155.4, 146.5, 144.0, 140.4, 137.0, 134.8, 133.0, 129.6, 127.7, 120.3, 104.9, 101.5, 55.5, 37.7, 21.0; **HRMS** (ESI) calcd. for $C_{21}H_{23}N_2O_4S$ [M+H]: 399.1379; found 399.1376.

_N_-(2-(3,5-dimethoxyphenyl)pyridin-3-yl)-_N_,4-dimethylbenzenesulfonamide (6b)

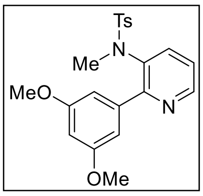

Purified by semi-preparative HPLC in hexanes/AcOEt gradient; off-white oil (0.021 mmol, 7.3 mg, 21% yield); **^{1}H NMR** (500 MHz, $CDCl_3$) δ 8.63 (dd, *J* = 4.7, 1.6 Hz, 1H), 7.54 (d, *J* = 8.3 Hz, 2H), 7.45 – 7.40 (m, 1H), 7.29 – 7.21 (m, 3H), 6.85 (d, *J* = 2.3 Hz, 2H), 6.52 (t, *J* = 2.3 Hz, 1H), 3.83 (s, 6H), 3.03 (s, 3H), 2.44 (s, 3H); **^{13}C NMR** (126 MHz, $CDCl_3$) δ 160.5, 158.4, 148.8, 143.8, 140.0, 136.1, 135.7, 129.6, 127.8, 122.7, 106.9, 101.4, 55.4, 38.8, 21.5.

_N_-(6-(2,6-dimethoxyphenyl)pyridin-3-yl)-_N_,4-dimethylbenzenesulfonamide (7a)

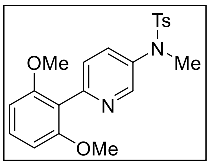

The ring closure reaction was performed in the microwave reactor (100 °C). Off-white solid (0.026 mmol, 8.9 mg, 26% yield); **^{1}H NMR** (400 MHz, $CDCl_3$) δ 8.29 (d, *J* = 1.9 Hz, 1H), 7.61 (dd, *J* = 8.4, 2.7 Hz, 1H), 7.49 (d, *J* = 8.3 Hz, 2H), 7.35 – 7.23 (m, 4H), 6.64 (d, *J* = 8.4 Hz, 2H), 3.73 (s, 6H), 3.23 (s, 3H), 2.41 (s, 3H); **^{13}C NMR** (126 MHz, $CDCl_3$) δ 158.0, 153.1, 146.1, 143.9, 136.2, 134.3, 133.2, 129.9, 129.5, 127.8, 126.2, 118.1, 104.1, 55.9, 37.8, 21.5; **HRMS** (ESI) calcd. for $C_{21}H_{23}N_2O_4S$ [M+H]: 399.1379; found 399.1381.

_N_,4-dimethyl-_N_-(6-(2-(methylthio)phenyl)pyridin-3-yl)benzenesulfonamide (8a)

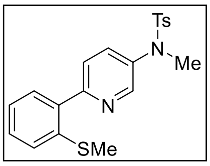

White solid (0.088 mmol, 30.2 mg, 88% yield); **^{1}H NMR** (600 MHz, $CDCl_3$) δ 8.30 (d, *J* = 2.0 Hz, 1H), 7.64 (dd, *J* = 8.4, 2.6 Hz, 1H), 7.55 (d, *J* = 8.4 Hz, 1H), 7.49 – 7.42 (m, 3H), 7.40 – 7.35 (m, 1H), 7.35 – 7.31 (m, 1H), 7.28 – 7.21 (m, 3H), 3.23 (s, 3H), 2.41 (s, 3H), 2.39 (s, 3H); **^{13}C NMR** (151 MHz, $CDCl_3$) δ 156.7, 145.9, 144.0, 138.5, 137.4, 136.7, 134.5, 133.0, 129.9, 129.6, 129.1, 127.7, 126.0, 124.9, 123.9, 37.8, 21.5, 16.4; **HRMS** (ESI) calcd. for $C_{20}H_{21}N_2O_2S_2$ [M+H]: 385.1044; found 385.1048.

_N_-(6-(4-(9H-carbazol-9-yl)phenyl)pyridin-3-yl)-_N_,4-dimethylbenzenesulfonamide (9a)

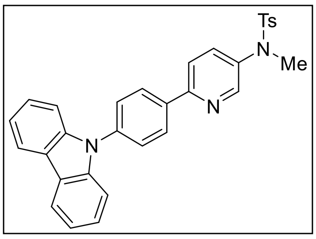

White solid (0.065 mmol, 32.7 mg, 65% yield); **^{1}H NMR** (400 MHz, $CDCl_3$) δ 8.40 (d, *J* = 2.6 Hz, 1H), 8.23 (d, *J* = 8.5 Hz, 2H), 8.16 (d, *J* = 7.8 Hz, 2H), 7.81 (d, *J* = 8.6 Hz, 1H), 7.70 (d, *J* = 8.5 Hz, 3H), 7.53 – 7.46 (m, 4H), 7.46 – 7.41 (m, 2H), 7.35 – 7.27 (m, 4H), 3.27 (s, 3H), 2.44 (s, 3H); **^{13}C NMR** (101 MHz, $CDCl_3$) δ 154.8, 146.9, 144.1, 140.7, 138.7, 137.3, 137.1, 135.0, 133.2, 129.7, 128.4, 127.8, 127.2, 126.0, 123.5, 120.3, 120.1, 120.1, 109.8, 37.8, 21.5; **HRMS** (ESI) calcd. for $C_{31}H_{26}N_3O_2S$ [M+H]: 504.1746; found 504.1749.

N-(2-(4-(9H-carbazol-9-yl)phenyl)pyridin-3-yl)-N,4-dimethylbenzenesulfonamide (9b)

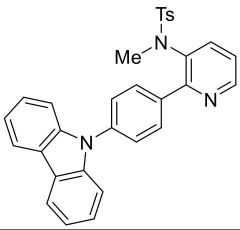

White solid (0.015 mmol, 7.6 mg, 15% yield); **^{1}H NMR** (400 MHz, $CDCl_3$) δ 8.71 (dd, J = 4.7, 1.6 Hz, 1H), 8.17 (d, J = 7.7 Hz, 2H), 7.90 (d, J = 8.4 Hz, 2H), 7.64 (d, J = 8.4 Hz, 2H), 7.59 – 7.49 (m, 5H), 7.48 – 7.41 (m, 2H), 7.37 – 7.27 (m, 5H), 3.17 (s, 3H), 2.41 (s, 3H); **^{13}C NMR** (101 MHz, $CDCl_3$) δ 149.1, 144.1, 140.7, 138.0, 137.4, 136.3, 130.5, 129.7, 128.1, 126.4, 126.0, 123.5, 122.9, 120.3, 120.1, 109.9, 38.9, 21.5; **HRMS** (ESI) calcd. for $C_{31}H_{26}N_3O_2S$ [M+H]: 504.1746; found 504.1750.

N,4-dimethyl-N-(6-(o-tolyl)pyridin-3-yl)benzenesulfonamide (10a)

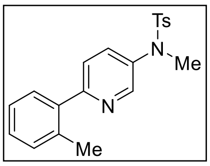

White solid (0.036 mmol, 12.8 mg, 36% yield); **^{1}H NMR** (500 MHz, CDCl3) δ 8.30 (d, J = 2.6 Hz, 1H), 7.63 (dd, J = 8.4, 2.7 Hz, 1H), 7.48 (d, J = 8.3 Hz, 2H), 7.42 – 7.37 (m, 2H), 7.34 – 7.23 (m, 5H), 3.24 (s, 3H), 2.42 (s, 3H), 2.37 (s, 3H); **^{13}C NMR** (126 MHz, $CDCl_3$) δ 158.5, 146.2, 144.1, 139.3, 136.4, 135.7, 134.5, 133.1, 130.8, 129.6, 129.6, 128.5, 127.7, 125.9, 123.8, 37.8, 21.5, 20.3; **HRMS** (ESI) calcd. for $C_{20}H_{21}N_2O_2S$ [M+H] 353.1324; found 353.1321.

N-(6-(2,4-dimethylphenyl)pyridin-3-yl)-N,4-dimethylbenzenesulfonamide (11a)

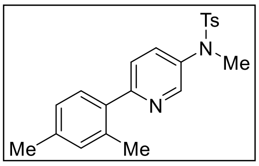

White solid (0.060 mmol, 22.0 mg, 60% yield); **^{1}H NMR** (500 MHz, $CDCl_3$) δ 8.29 (d, J = 2.6 Hz, 1H), 7.62 (dd, J = 8.4, 2.7 Hz, 1H), 7.48 (d, J = 8.4 Hz, 2H), 7.37 (d, J = 8.4 Hz, 1H), 7.33 – 7.25 (m, 3H), 7.09 (d, J = 8.8 Hz, 2H), 3.24 (s, 3H), 2.42 (s, 3H), 2.37 (s, 3H), 2.34 (s, 3H); **^{13}C NMR** (126 MHz, $CDCl_3$) δ 158.5, 146.2, 144.0, 138.3, 136.5, 136.1, 135.6, 134.5, 133.1, 131.6, 129.6, 127.7, 126.6, 123.8, 37.8, 21.5, 21.1, 20.3; **HRMS** (ESI) calcd. for $C_{21}H_{23}N_2O_2S$ [M+H] 367.1480; found 367.1483.

N-(6-(3,5-di-tert-butylphenyl)pyridin-3-yl)-N,4-dimethylbenzenesulfonamide (12a)

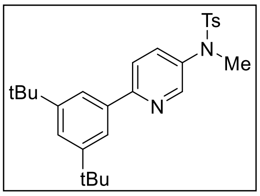

Purified by semi-preparative HPLC in hexanes/AcOEt gradient; pale yellow oil (0.054 mmol, 24.4 mg, 54% yield); **^{1}H NMR** (500 MHz, $CDCl_3$) δ 8.31 (d, J = 1.9 Hz, 1H), 7.79 (d, J = 1.8 Hz, 2H), 7.70 (d, J = 8.6 Hz, 1H), 7.63 (dd, J = 8.5, 2.6 Hz, 1H), 7.51 (t, J = 1.8 Hz, 1H), 7.47 (d, J = 8.3 Hz, 2H), 7.27 (d, J = 6.5 Hz, 2H), 3.23 (s, 3H), 2.42 (s, 3H), 1.39 (s, 18H); **^{13}C NMR** (126 MHz, $CDCl_3$) δ 157.0, 151.2, 146.5, 143.9, 137.8, 136.5, 135.0, 133.1, 129.6, 127.8, 123.5, 121.3, 120.5, 37.8, 35.0, 31.4, 21.5. **HRMS** (ESI) calcd. for $C_{27}H_{35}N_2O_2S$ [M+H] 451.2419; found 451.2415.

N-(2-(3,5-di-tert-butylphenyl)pyridin-3-yl)-N,4-dimethylbenzenesulfonamide (12b)

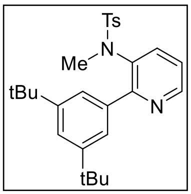

Purified by semi-preparative HPLC in hexanes/ AcOEt gradient; pale yellow oil (0.017 mmol, 7.6 mg, 17% yield); **^{1}H NMR** (500 MHz, $CDCl_3$) δ 8.66 (dd, J = 4.7, 1.6 Hz, 1H), 7.52 – 7.46 (m, 4H), 7.34 (d, J = 8.4 Hz, 2H), 7.23 (dd, J = 8.0, 4.7 Hz, 1H), 7.17 (d, J = 8.0 Hz, 2H), 3.03 (s, 3H), 2.40 (s, 3H), 1.34 (s, 18H); **^{13}C NMR** (126 MHz, $CDCl_3$) δ 160.3, 150.4, 148.9, 143.4, 137.5, 136.1, 136.0, 135.8, 129.5, 127.8, 123.1, 122.5, 122.3, 38.6, 34.9, 31.4, 21.5.

***N*-(6-([1,1'-biphenyl]-2-yl)pyridin-3-yl)-*N*,4-dimethylbenzenesulfonamide (13a)**

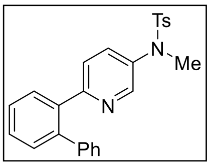

Purified by semi-preparative HPLC in hexanes/AcOEt gradient; white oil (0.040 mmol, 16.6 mg, 40% yield +~25% MeNHTs); ^{1}H NMR (500 MHz, $CDCl_3$) δ 8.27 (d, J = 2.7 Hz, 1H), 7.70 – 7.64 (m, 1H), 7.49 – 7.40 (m, 3H), 7.36 (d, *J* = 8.3 Hz, 2H), 7.27 – 7.21 (m, 5H), 7.18 (dd, *J* = 8.5, 2.6 Hz, 1H), 7.16 – 7.11 (m, 2H), 6.84 (d, *J* = 8.4 Hz, 1H), 3.14 (s, 3H), 2.40 (s, 3H); **^{13}C NMR** (126 MHz, $CDCl_3$) δ 157.8, 146.7, 144.0, 141.2, 140.6, 138.5, 136.0, 133.1, 132.8, 130.5, 130.3, 129.6, 129.6, 129.5, 128.7, 128.0, 127.7, 127.6, 127.2, 126.8, 125.0, 37.7, 21.5; **HRMS** (ESI) calcd. for $C_{25}H_{23}N_2O_2S$ [M+H]: 415.1480; found 415.1484.

***N*-(6-([1,1'-biphenyl]-3-yl)pyridin-3-yl)-*N*,4-dimethylbenzenesulfonamide (14a)**

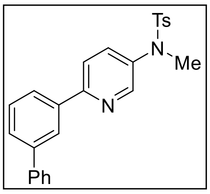

Purified by semi-preparative HPLC in hexanes/ AcOEt gradient; white solid (0.065 mmol, 26.9 mg, 65% yield); **^{1}H NMR** (500 MHz, $CDCl_3$) δ 8.34 (d, *J* = 2.6 Hz, 1H), 8.22 (s, 1H), 7.94 (d, *J* = 7.6 Hz, 1H), 7.77 (d, *J* = 8.6 Hz, 1H), 7.66 (t, *J* = 6.4 Hz, 4H), 7.55 (t, *J* = 7.7 Hz, 1H), 7.50 – 7.44 (m, 4H), 7.37 (t, *J* = 7.3 Hz, 1H), 7.27 (d, *J* = 8.0 Hz, 2H), 3.24 (s, 3H), 2.42 (s, 3H); **^{13}C NMR** (126 MHz, $CDCl_3$) δ 155.6, 146.7, 144.0, 141.8, 140.9, 138.8, 136.9, 135.0, 133.0, 129.6, 129.2, 128.8, 128.7, 128.0, 127.7, 127.4, 127.2, 125.8, 125.7, 120.2, 37.8, 21.5. **HRMS** (ESI) calcd. for $C_{25}H_{23}N_2O_2S$ [M+H]: 415.1480; found 415.1479.

***N*-(2-([1,1'-biphenyl]-3-yl)pyridin-3-yl)-*N*,4-dimethylbenzenesulfonamide (14b)**

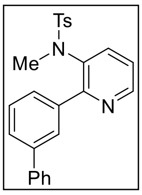

Purified by semi-preparative HPLC in hexanes/AcOEt gradient; white solid (0.018 mmol, 7.5 mg, 18% yield); **^{1}H NMR** (500 MHz, $CDCl_3$) δ 8.69 – 8.65 (m, 1H), 7.85 (s, 1H), 7.67 – 7.58 (m, 4H), 7.56 – 7.51 (m, 1H), 7.51 – 7.40 (m, 5H), 7.34 (t, *J* = 7.2 Hz, 1H), 7.27 (d, *J* = 13.6 Hz, 1H), 7.15 (d, *J* = 8.0 Hz, 2H), 3.09 (s, 3H), 2.34 (s, 3H); **^{13}C NMR** (126 MHz, $CDCl_3$) δ 158.7, 149.0, 143.8, 141.0, 140.9, 138.7, 136.3, 136.1, 135.0, 129.5, 128.7, 128.6, 127.9, 127.8, 127.7, 127.2, 127.2, 122.7, 38.8, 21.4.

***N*-(6-([1,1'-biphenyl]-4-yl)pyridin-3-yl)-*N*,4-dimethylbenzenesulfonamide (15a)**

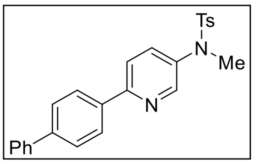

Purified by semi-preparative HPLC in hexanes/AcOEt gradient; white solid (0.061 mmol, 25.3 mg, 61% yield); **^{1}H NMR** (500 MHz, $CDCl_3$) δ 8.34 (d, *J* = 2.7 Hz, 1H), 8.06 (d, *J* = 8.4 Hz, 2H), 7.75 (d, *J* = 8.5 Hz, 1H), 7.72 (d, *J* = 8.5 Hz, 2H), 7.68 – 7.63 (m, 3H), 7.47 (t, *J* = 8.6 Hz, 4H), 7.38 (d, *J* = 8.7 Hz, 1H), 7.28 (d, *J* = 8.0 Hz, 2H), 3.22 (s, 3H), 2.42 (s, 3H); **^{13}C NMR** (126 MHz, $CDCl_3$) δ 155.3, 146.7, 144.0, 142.0, 140.4, 137.2, 136.8, 135.0, 133.0, 129.6, 128.8, 127.7, 127.6, 127.5, 127.2, 127.0, 120.0, 37.8, 30.9; **HRMS** (ESI) calcd. for $C_{25}H_{23}N_2O_2S$ [M+H]: 415.1480; found 415.1482.

***N*-(2-([1,1'-biphenyl]-4-yl)pyridin-3-yl)-*N*,4-dimethylbenzenesulfonamide (15b)**

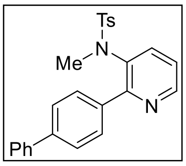

Purified by semi-preparative HPLC in hexanes/AcOEt gradient; white solid (0.019 mmol, 7.9 mg, 19% yield); **^{1}H NMR** (500 MHz, $CDCl_3$) δ 8.67 (dd, *J* = 4.7, 1.6 Hz, 1H), 7.72 (d, *J* = 8.4 Hz, 2H), 7.65 (dd, *J* = 12.8, 7.7 Hz, 4H), 7.51 – 7.44 (m, 5H), 7.41 – 7.31 (m, 1H), 7.25 – 7.20 (m, 3H), 3.11 (s, 3H), 2.41 (s, 3H); **^{13}C NMR** (126 MHz, $CDCl_3$) δ 158.3, 149.0, 143.8, 141.2, 140.7, 137.3, 136.2, 136.1, 135.1, 129.5, 129.3, 128.8, 127.9, 127.4, 127.1, 126.8, 122.6, 38.8, 21.5; **HRMS** (ESI) calcd. for $C_{25}H_{23}N_2O_2S$ [M+H]: 415.1480; found 415.1484.

N,4-dimethyl-N-(6-(naphthalen-2-yl)pyridin-3-yl)benzenesulfonamide (16a)

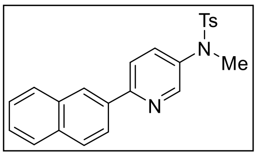

Off-white solid (0.047 mmol, 18.1 mg, 47% yield); **^{1}H NMR** (500 MHz, $CDCl_3$) δ 8.45 (s, 1H), 8.37 (d, J = 2.7 Hz, 1H), 8.10 (dd, J = 8.6, 1.8 Hz, 1H), 7.94 (d, J = 8.8 Hz, 2H), 7.90 – 7.84 (m, 2H), 7.70 – 7.66 (m, 1H), 7.52 (dt, J = 6.3, 3.4 Hz, 2H), 7.49 (d, J = 8.3 Hz, 2H), 7.28 (d, J = 9.3 Hz, 2H), 3.25 (s, 3H), 2.42 (s, 3H); **^{13}C NMR** (126 MHz, $CDCl_3$) δ 155.9, 147.0, 144.3, 137.1, 135.8, 135.4, 133.9, 133.7, 133.3, 129.9, 128.9, 128.8, 128.0, 127.9, 127.0, 126.7, 126.7, 124.6, 120.7, 38.0, 21.8; **HRMS** (ESI) calcd. for $C_{23}H_{21}N_2O_2S$ [M+H]: 389.1324; found 389.1327.

N,4-dimethyl-N-(6-(phenanthren-9-yl)pyridin-3-yl)benzenesulfonamide (17a)

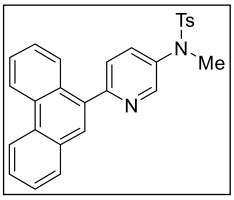

White solid (0.070 mmol, 30.7 mg, 70% yield); **^{1}H NMR** (400 MHz, $CDCl_3$) δ 8.76 (dd, J = 22.3, 7.9 Hz, 2H), 8.44 (d, J = 2.6 Hz, 1H), 8.08 (d, J = 7.8 Hz, 1H), 7.94 (d, J = 9.4 Hz, 1H), 7.87 (s, 1H), 7.78 – 7.67 (m, 3H), 7.64 (d, J = 8.3 Hz, 2H), 7.60 (d, J = 8.3 Hz, 1H), 7.56 (d, J = 8.3 Hz, 2H), 7.32 (d, J = 8.4 Hz, 2H), 3.31 (s, 3H), 2.44 (s, 3H); **^{13}C NMR** (101 MHz, $CDCl_3$) δ 157.8, 146.6, 144.2, 137.0, 136.1, 134.9, 133.3, 131.2, 130.8, 130.5, 130.0, 129.7, 129.7, 129.0, 128.8, 127.8, 127.2, 126.9, 126.7, 126.7, 126.3, 124.9, 123.0, 122.6, 37.9, 21.5; **HRMS** (ESI) calcd. for $C_{27}H_{23}N_2O_2S$ [M+H]: 439.1480; found 439.1484.

N,4-dimethyl-N-(6-(pyren-4-yl)pyridin-3-yl)benzenesulfonamide (18a)

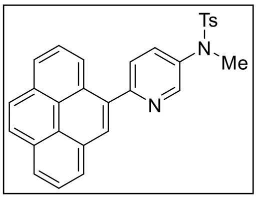

White solid (0.065 mmol, 30.0 mg, 65% yield); **^{1}H NMR** (400 MHz, $CDCl_3$) δ 8.52 (d, J = 2.3 Hz, 1H), 8.41 (d, J = 9.3 Hz, 1H), 8.30 – 8.15 (m, 4H), 8.15 – 8.09 (m, 3H), 8.05 (t, J = 7.6 Hz, 1H), 7.83 – 7.72 (m, 2H), 7.59 (d, J = 8.1 Hz, 2H), 7.34 (d, J = 7.9 Hz, 2H), 3.34 (s, 3H), 2.46 (s, 3H); **^{13}C NMR** (101 MHz, $CDCl_3$) δ 157.9, 146.8, 144.2, 136.7, 134.8, 134.5, 133.3, 131.6, 131.4, 130.8, 129.7, 129.7, 128.6, 128.2, 128.1, 127.8, 127.6, 127.3, 127.2, 126.1, 125.5, 125.4, 125.2, 125.1, 124.8, 124.5, 37.9, 21.5; **HRMS** (ESI) calcd. for $C_{29}H_{23}N_2O_2S$ [M+H]: 463.1480; found 463.1483.

N-(6-(4-cyanophenyl)pyridin-3-yl)-N,4-dimethylbenzenesulfonamide (19a)

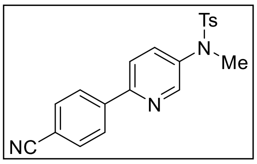

White solid (0.051 mmol, 18.5 mg, 51% yield); **^{1}H NMR** (500 MHz, $CDCl_3$) δ 8.40 (d, J = 1.9 Hz, 1H), 8.10 (d, J = 8.5 Hz, 2H), 7.75 (t, J = 8.8 Hz, 3H), 7.68 (dd, J = 8.5, 2.6 Hz, 1H), 7.47 (d, J = 8.3 Hz, 2H), 7.28 (d, J = 7.9 Hz, 2H), 3.24 (s, 3H), 2.43 (s, 3H); **^{13}C NMR** (126 MHz, $CDCl_3$) δ 153.7, 147.4, 144.7, 142.9, 138.5, 135.3, 133.5, 133.1, 130.2, 128.2, 127.9, 121.1, 119.2, 113.2, 38.1, 22.0; **HRMS** (ESI) calcd. for $C_{20}H_{18}N_3O_2S$ [M+H] 364.1120; found 522.1522.

N,4-dimethyl-N-(6-(4-nitrophenyl)pyridin-3-yl)benzenesulfonamide (20a)

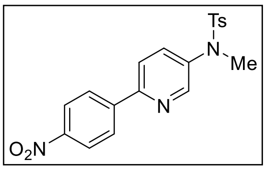

White solid (0.098 mmol, 37.6 mg, 98% yield); **^{1}H NMR** (500 MHz, $CDCl_3$) δ 8.43 (d, J = 2.6 Hz, 1H), 8.33 (d, J = 8.9 Hz, 2H), 8.17 (d, J = 8.9 Hz, 2H), 7.79 (d, J = 7.9 Hz, 1H), 7.70 (dd, J = 8.5, 2.6 Hz, 1H), 7.47 (d, J = 8.3 Hz, 2H), 7.28 (d, J = 8.0 Hz, 2H), 3.25 (s, 3H), 2.43 (s, 3H); **^{13}C NMR** (126 MHz, $CDCl_3$) δ 152.7, 148.2, 146.9, 144.3, 144.1, 138.1, 134.7, 133.0, 129.7, 127.7, 127.6, 124.0, 120.8, 37.6, 21.5; **HRMS** (ESI) calcd. for $C_{19}H_{18}N_3O_4S$ [M+H]: 384.1018; found 384.1021.

***N*,4-dimethyl-*N*-(6-(3-nitrophenyl)pyridin-3-yl)benzenesulfonamide (21a)**

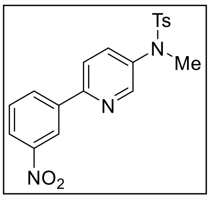

White solid (0.061 mmol, 21.0 mg, 61% yield); **^{1}H NMR** (400 MHz, $CDCl_3$) δ 8.75 (t, *J* = 2.0 Hz, 1H), 8.32 (dd, *J* = 2.7, 0.8 Hz, 1H), 8.25 (ddd, *J* = 7.8, 1.8, 1.0 Hz, 1H), 8.17 (ddd, *J* = 8.2, 2.3, 1.1 Hz, 1H), 7.74 – 7.67 (m, 1H), 7.61 – 7.53 (m, 2H), 7.40 – 7.35 (m, 2H), 7.23 – 7.16 (m, 2H), 3.15 (s, 3H), 2.33 (s, 3H). **^{13}C NMR** (101 MHz, $CDCl_3$) δ 152.8, 147.0, 144.3, 140.0, 138.0, 134.9, 133.0, 132.6, 129.8, 129.7, 129.7, 128.3, 127.7, 126.4, 123.7, 121.7, 120.2, 37.6, 21.5; **HRMS** (ESI) calcd. for $C_{19}H_{18}N_3O_4S$ [M+H]: 384.1018; found 384.1020.

***N*,4-dimethyl-*N*-(6-(4-(trifluoromethyl)phenyl)pyridin-3-yl)benzenesulfonamide (22a)**

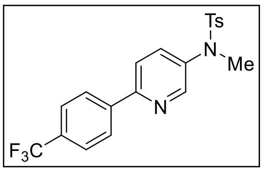

White solid (0.042 mmol, 17.2 mg, 42% yield); **^{1}H NMR** (500 MHz, $CDCl_3$) δ 8.38 (d, *J* = 1.9 Hz, 1H), 8.10 (d, *J* = 8.0 Hz, 2H), 7.74 (t, *J* = 8.3 Hz, 3H), 7.67 (dd, *J* = 8.5, 2.6 Hz, 1H), 7.47 (d, *J* = 8.3 Hz, 2H), 7.28 (d, *J* = 8.0 Hz, 2H), 3.24 (s, 3H), 2.40 (s, 3H); **^{13}C NMR** (126 MHz, $CDCl_3$) δ 153.9, 146.9, 144.2, 141.6, 137.6, 134.9, 133.0, 131.1, 130.9, 129.7, 127.7, 127.1, 125.8, 125.7, 125.7, 125.7, 125.1, 123.0, 120.4, 37.7, 21.5; **HRMS** (ESI) calcd. for $C_{20}H_{18}F_3N_2O_2S$ [M+H]: 407.1041; found 407.1043.

***N*-(6-(3,5-difluorophenyl)pyridin-3-yl)-*N*,4-dimethylbenzenesulfonamide (23a)**

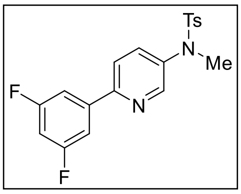

White solid (0.072 mmol, 26.9 mg, 72% yield); **^{1}H NMR** (400 MHz, $CDCl_3$) δ 8.35 (t, *J* = 1.7 Hz, 1H), 7.66 (d, *J* = 1.7 Hz, 2H), 7.56 – 7.49 (m, 2H), 7.46 (d, *J* = 8.3 Hz, 2H), 7.28 (d, *J* = 7.8 Hz, 2H), 6.90 – 6.82 (m, 1H), 3.23 (s, 3H), 2.43 (s, 3H); **^{13}C NMR** (101 MHz, $CDCl_3$) δ 164.7, 164.6, 162.2, 162.1, 146.7, 144.2, 137.8, 134.8, 133.1, 129.7, 127.7, 120.1, 109.8, 109.7, 109.6, 109.5, 104.6, 104.4, 104.1, 37.6, 21.5; **HRMS** (ESI) calcd. for $C_{19}H_{17}F_2N_2O_2S$ [M+H]: 375.0975; found 375.0983.

***N*-(6-(furan-2-yl)pyridin-3-yl)-*N*,4-dimethylbenzenesulfonamide (24a)**

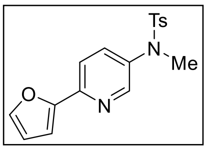

Purified by semi-preparative HPLC in the hexanes/AcOEt gradient (*N*-methyltosylamide is inseparable from the final product); white solid (0.014 mmol, 4.6 mg, 14% yield + TsNHMe); **^{1}H NMR** (400 MHz, $CDCl_3$) δ 8.24 (d, *J* = 2.6 Hz, 1H), 7.65 (d, *J* = 8.6 Hz, 1H), 7.58 (dd, *J* = 8.6, 2.6 Hz, 1H), 7.55 (s, 1H), 7.46 (d, *J* = 8.3 Hz, 2H), 7.30 – 7.25 (m, 2H), 7.04 (d, *J* = 3.4 Hz, 1H), 6.55 (s, 1H), 3.22 (s, 3H), 2.43 (s, 3H); **^{13}C NMR** (both for product and *N*-methyltosylamide) (101 MHz, $CDCl_3$) δ 147.7, 146.9, 144.0, 143.6, 143.5, 136.4, 134.9, 133.1, 129.7, 129.6, 127.7, 127.2, 118.3, 112.2, 109.2, 37.8, 29.3, 21.5, 21.5; **HRMS** (ESI) calcd. for $C_{17}H_{17}N_2O_3S$ [M+H]: 329.0960; found 329.0963.

***N*-(2-(furan-2-yl)pyridin-3-yl)-*N*,4-dimethylbenzenesulfonamide (24b)**

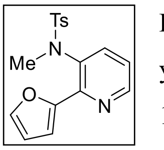

Purified by semi-preparative HPLC in hexanes/AcOEt gradient; white solid (0.021 mmol, 6.9 mg, 21% yield); **^{1}H NMR** (400 MHz, $CDCl_3$) δ 8.63 (dd, *J* = 4.6, 1.6 Hz, 1H), 7.64 (d, *J* = 8.3 Hz, 2H), 7.51 (dd, *J* = 1.8, 0.8 Hz, 1H), 7.34 – 7.29 (m, 3H), 7.24 (dd, *J* = 8.0, 1.6 Hz, 1H), 7.12 (dd, *J* = 8.0, 4.6 Hz, 1H), 6.53 (dd, *J* = 3.5, 1.8 Hz, 1H), 3.21 (s, 3H), 2.46 (s, 3H); **^{13}C NMR** (101 MHz, $CDCl_3$) δ 149.2, 143.9, 143.8, 136.6, 135.3, 133.7, 129.6, 128.0, 121.8, 113.5, 112.0, 38.4, 21.5; **HRMS** (ESI) calcd. for $C_{17}H_{17}N_2O_3S$ [M+H]: 329.0960; found 329.0962.

***N*,4-dimethyl-*N*-(6-(thiophen-2-yl)pyridin-3-yl)benzenesulfonamide (25a)**

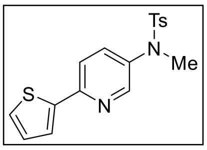

Off-white solid (0.049 mmol, 17.0 mg, 49% yield); **^{1}H NMR** (500 MHz, $CDCl_3$) δ 8.18 (dd, *J* = 2.5, 0.8 Hz, 1H), 7.62 (dd, *J* = 8.6, 0.9 Hz, 1H), 7.60 – 7.54 (m, 2H), 7.49 – 7.43 (m, 2H), 7.41 (dd, *J* = 5.0, 1.1 Hz, 1H), 7.27 (d, *J* = 8.2 Hz, 2H), 7.11 (dd, *J* = 5.0, 3.7 Hz, 1H), 3.20 (s, 3H), 2.42 (s, 3H); **^{13}C NMR** (126 MHz, $CDCl_3$) δ 151.3, 146.7, 144.3, 144.0, 136.7, 135.3, 133.2, 129.9, 128.4, 128.3, 128.0, 125.3, 118.7, 38.0, 21.8; **HRMS** (ESI) calcd. for $C_{17}H_{17}N_2O_2S_2$ [M+H]: 345.0731; found 345.0730.

***N*-(6-(benzo[b]thiophen-2-yl)pyridin-3-yl)-*N*,4-dimethylbenzenesulfonamide (26a)**

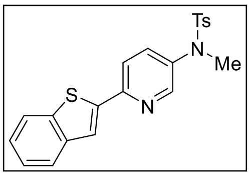

White solid (0.056 mmol, 22.2 mg, 56% yield); **^{1}H NMR** (400 MHz, $CDCl_3$) δ 8.25 (dd, *J* = 2.6, 0.7 Hz, 1H), 7.91 – 7.73 (m, 4H), 7.65 (dd, *J* = 8.6, 2.6 Hz, 1H), 7.52 – 7.45 (m, 2H), 7.42 – 7.33 (m, 2H), 7.28 (d, *J* = 7.9 Hz, 2H), 3.23 (s, 3H), 2.43 (s, 3H); **^{13}C NMR** (101 MHz, CDCl3) δ 150.9, 146.4, 144.1, 143.7, 140.8, 140.4, 137.1, 134.9, 133.0, 129.7, 127.7, 125.2, 124.6, 124.2, 122.5, 121.6, 119.3, 37.7, 21.5; **HRMS** (ESI) calcd. for $C_{21}H_{19}N_2O_2S_2$ [M+H]: 395.0888; found 395.0883.

***N*-(6-(benzo[b]thiophen-3-yl)pyridin-3-yl)-*N*,4-dimethylbenzenesulfonamide (27a)**

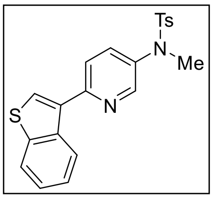

White solid (0.079 mmol, 31.3 mg, 79% yield); **^{1}H NMR** (500 MHz, $CDCl_3$) δ 8.50 – 8.45 (m, 1H), 8.37 (dt, *J* = 1.9, 0.9 Hz, 1H), 7.90 (dt, *J* = 7.9, 1.0 Hz, 1H), 7.81 (s, 1H), 7.70 – 7.62 (m, 2H), 7.51 – 7.30 (m, 5H), 7.30 – 7.23 (m, 1H), 3.24 (d, *J* = 0.7 Hz, 3H), 2.41 (s, 3H); **^{13}C NMR** (126 MHz, $CDCl_3$) δ 153.0, 146.6, 144.1, 140.8, 136.9, 136.5, 135.5, 135.0, 133.1, 129.7, 128.3, 127.8, 126.9, 124.7, 124.7, 124.1, 122.7, 122.1, 37.8, 21.5; **HRMS** (ESI) calcd. for $C_{21}H_{19}N_2O_2S_2$ [M+H]: 395.0888; found 395.0883.

***tert-butyl* 2-(5-((*N*,4-dimethylphenyl)sulfonamido)pyridin-2-yl)-1*H*-pyrrole-1-carboxylate (28a)**

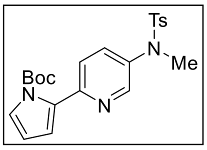

White solid (0.026 mmol, 11.1 mg, 26% yield); **^{1}H NMR** (400 MHz, $CDCl_3$) δ 8.29 – 8.23 (m, 1H), 7.58 – 7.46 (m, 3H), 7.41 – 7.34 (m, 2H), 7.29 (s, 2H), 6.46 (dt, *J* = 3.2, 1.5 Hz, 1H), 6.25 (td, *J* = 3.3, 1.3 Hz, 1H), 3.22 (d, *J* = 1.3 Hz, 3H), 2.43 (s, 3H), 1.42 (d, *J* = 1.3 Hz, 9H); **^{13}C NMR** (101 MHz, $CDCl_3$) δ 146.2, 144.3, 137.6, 136.6, 134.0, 133.3, 129.6, 127.8, 126.6, 125.1, 124.1, 123.0, 116.3, 110.6, 83.9, 37.9, 27.6, 21.5; **HRMS** (ESI) calcd. for $C_{22}H_{26}N_3O_4S$ [M+H]: 428.1644; found 428.1645.

***tert-butyl* 2-(5-((*N*,4-dimethylphenyl)sulfonamido)pyridin-2-yl)-1*H*-indole-1-carboxylate (29a)**

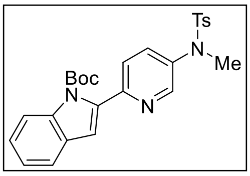

White solid (0.049 mmol, 23.3 mg, 49% yield); **^{1}H NMR** (500 MHz, $CDCl_3$) δ 8.32 (d, *J* = 2.3 Hz, 1H), 8.18 – 8.13 (m, 1H), 7.63 – 7.56 (m, 2H), 7.53 – 7.46 (m, 3H), 7.40 – 7.33 (m, 1H), 7.31 – 7.22 (m, 3H), 6.80 (d, *J* = 0.9 Hz, 1H), 3.24 (d, *J* = 1.0 Hz, 3H), 2.43 (s, 3H), 1.40 (d, *J* = 1.0 Hz, 9H); **^{13}C NMR** (126 MHz, $CDCl_3$) δ 151.8, 150.1, 146.6, 144.4, 138.6, 137.9, 137.1, 134.5, 133.6, 129.9, 129.0, 128.0, 125.4, 123.2, 123.1, 121.3, 115.2, 111.9, 83.9, 38.2, 27.9, 21.8; **HRMS** (ESI) calcd. for $C_{26}H_{28}N_3O_4S$ [M+H]: 478.1801; found 478.1803.

***N*-(6-(methoxy(phenyl)methyl)pyridin-3-yl)-*N*,4-dimethylbenzenesulfonamide (35a)**

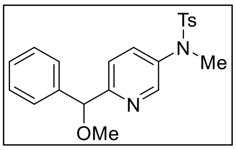

White solid (0.043 mmol, 14.9 mg, 43% yield); **^{1}H NMR** (500 MHz, $CDCl_3$) δ 8.17 (d, J = 2.5 Hz, 1H), 7.53 (dd, J = 8.5, 2.6 Hz, 1H), 7.46 (d, J = 8.5 Hz, 1H), 7.40 (dd, J = 7.6, 4.0 Hz, 4H), 7.34 (t, J = 7.5 Hz, 2H), 7.28 (d, J = 7.3 Hz, 1H), 7.23 (d, J = 8.0 Hz, 2H), 5.35 (s, 1H), 3.42 (s, 3H), 3.15 (s, 3H), 2.41 (s, 3H); **^{13}C NMR** (126 MHz, $CDCl_3$) δ 160.1, 146.3, 144.0, 140.3, 136.9, 135.0, 133.1, 129.6, 128.5, 127.8, 127.7, 126.9, 120.3, 86.0, 57.1, 37.8, 21.5. **HRMS** (ESI) calcd. for $C_{21}H_{31}N_2O_3S$ [M+H]: 384.1429; found 384.1428.

4.3. General procedure for the functionalization of 2-alkyl Zincke imines (General procedure 4)

General Procedure 4. Zincke imine (0.05 mmol), **Py-salt** (0.075 mmol), $Ir(ppy)_3$ (0.67 mg, ~10 µmol, 2 mol%) were placed in the closed-cup vial and MeCN (8 ml) were added through the septum. The reaction mixture was placed in ultrasound bath and degassed by bubbling argon through the solution for 15 min. The vial was then moved to the photoreactor and irradiated with violet light (4.8 W) for 24 h, maintaining a temperature between 0 °C and 5 °C. After the indicated time, the saturated NH_4OAc solution in anhydrous ethanol was added (2 ml) and the reaction mixture was heated up to 65 °C for 2 h. The excess of NH_4OAc was removed by extraction (AcOEt/ H_2O). The organic phase was dried over anhydrous sodium sulphate and evaporated with silica gel (dry load for the preparation of the sample for flash chromatography). The pure products were isolated by flash chromatography in hexanes/AcOEt gradient.

Flash program: time: 25 min; column: silica 4 g; flow rate: 13 mL/min; automatic peak hold: on.

entry	time [min]	hexanes [%]	AcOEt [%]
1	0	90	10
2	25	50	50

Note:

- *Several products, especially minor regioisomers, were initially isolated by the column or flash chromatography in a* hexanes*/AcOEt gradient. Due to some impurities from tailing N-methyltosylamide, further purification by semi-preparative normal phase HPLC was necessary to obtain pure compounds.*

***N*,4-dimethyl-*N*-(2-methylpyridin-3-yl)benzenesulfonamide (30b)**

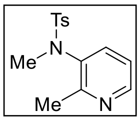

White solid (0.074 mmol, 20.4 mg, 74% yield); **^{1}H NMR** (400 MHz, $CDCl_3$) δ 8.45 (d, J = 3.0 Hz, 1H), 7.57 (d, J = 6.4 Hz, 2H), 7.31 (d, J = 8.1 Hz, 2H), 7.03 (d, J = 3.8 Hz, 2H), 3.13 (s, 3H), 2.54 (s, 3H), 2.45 (s, 3H); **^{13}C NMR** (101 MHz, $CDCl_3$) δ 159.6, 148.6, 143.9, 136.6, 135.1, 135.1, 134.8, 129.6, 127.9, 121.3, 38.6, 21.5, 21.3; **HRMS** (ESI) calcd. for $C_{14}H_{17}N_2O_2S$ [M+H]: 277.1013; found 277.1011.

N-(2-ethylpyridin-3-yl)-N,4-dimethylbenzenesulfonamide (31b)

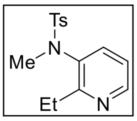

White solid (0.070 mmol, 20.4 mg, 70% yield); **^{1}H NMR** (500 MHz, $CDCl_3$) δ 8.50 (dd, *J* = 4.7, 1.7 Hz, 1H), 7.57 (d, *J* = 8.3 Hz, 2H), 7.31 (d, *J* = 7.7 Hz, 2H), 7.02 (dd, *J* = 8.0, 4.6 Hz, 1H), 6.97 (dd, *J* = 8.0, 1.7 Hz, 1H), 3.13 (s, 3H), 2.91 (s, 2H), 2.45 (s, 3H), 1.28 (t, *J* = 7.6 Hz, 3H); **^{13}C NMR** (126 MHz, $CDCl_3$) δ 164.2, 149.1, 144.1, 136.3, 135.1, 134.8, 129.9, 128.2, 121.4, 39.4, 26.8, 21.8, 13.1; **HRMS** (ESI) calcd. for $C_{15}H_{19}N_2O_2S$ [M+H]: 291.1167; found 291.1171.

N-(2-hexanoylpyridin-3-yl)-N,4-dimethylbenzenesulfonamide (32b)

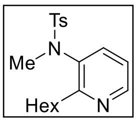

White solid (0.066 mmol, 23.6 mg, 66% yield); **^{1}H NMR** (500 MHz, $CDCl_3$) δ 8.49 (dd, *J* = 4.5, 1.9 Hz, 1H), 7.58 (d, *J* = 8.2 Hz, 2H), 7.31 (d, *J* = 8.5 Hz, 2H), 7.05 – 6.97 (m, 2H), 3.13 (s, 3H), 2.45 (s, 3H), 1.76 – 1.62 (m, 2H), 1.39 – 1.25 (m, 6H), 0.93 – 0.84 (m, 3H); **^{13}C NMR** (126 MHz, $CDCl_3$) δ 163.1, 148.8, 143.9, 136.2, 134.9, 134.6, 129.6, 128.0, 121.1, 39.2, 33.5, 31.7, 29.5, 28.8, 22.6, 21.5, 14.0; **HRMS** (ESI) calcd. for $C_{19}H_{27}N_2O_2S$ [M+H]: 347.1793; found 347.1790.

N-(6-isopropylpyridin-3-yl)-N,4-dimethylbenzenesulfonamide (33a)

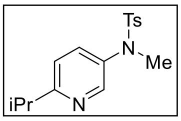

White solid (0.044 mmol, 13.4 mg, 44% yield); **^{1}H NMR** (500 MHz, $CDCl_3$) δ 8.14 (d, *J* = 1.9 Hz, 1H), 7.49 – 7.42 (m, 3H), 7.28 (s, 2H), 7.14 (d, *J* = 8.4 Hz, 1H), 3.17 (s, 3H), 3.06 (dt, *J* = 13.8, 6.9 Hz, 1H), 2.42 (s, 3H), 1.29 (d, *J* = 6.9 Hz, 6H); **^{13}C NMR** (126 MHz, $CDCl_3$) δ 166.1, 146.4, 143.9, 135.8, 135.1, 133.3, 129.6, 127.7, 120.5, 38.0, 35.9, 22.4, 21.5, **HRMS** (ESI) calcd. for $C_{16}H_{21}N_2O_2S$ [M+H]: 305.1324; found 305.1326.

N-(2-isopropylpyridin-3-yl)-N,4-dimethylbenzenesulfonamide (33b)

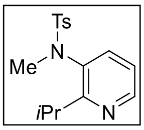

White solid (0.031 mmol, 9.4 mg, 31% yield); **^{1}H NMR** (500 MHz, $CDCl_3$) δ 8.55 (dd, J = 4.6, 1.9 Hz, 1H), 7.60 (d, J = 8.1 Hz, 2H), 7.32 (d, J = 7.3 Hz, 2H), 7.03 – 6.95 (m, 2H), 3.70 – 3.58 (m, 1H), 3.15 (s, 3H), 2.45 (s, 3H), 1.28 (d, J = 6.7 Hz, 3H), 1.19 (d, J = 6.8 Hz, 3H); **^{13}C NMR** (126 MHz, $CDCl_3$) δ 167.5, 149.1, 143.8, 135.2, 134.8, 134.8, 129.6, 128.0, 121.0, 77.2, 77.0, 76.7, 39.4, 29.6, 23.0, 21.3.

N-(6-benzylpyridin-3-yl)-N,4-dimethylbenzenesulfonamide (34b)

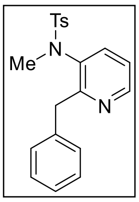

White solid (0.040 mmol, 14.1 mg, 40% yield); **^{1}H NMR** (500 MHz, $CDCl_3$) δ 8.52 (dd, *J* = 4.7, 1.6 Hz, 1H), 7.57 (d, *J* = 8.3 Hz, 2H), 7.32 (d, *J* = 8.7 Hz, 2H), 7.27 (d, *J* = 7.1 Hz, 4H), 7.17 (t, *J* = 6.5 Hz, 1H), 7.05 (dd, *J* = 8.0, 4.7 Hz, 1H), 6.86 (dd, *J* = 8.0, 1.6 Hz, 1H), 4.64 (s, 1H), 4.16 (s, 1H), 2.77 (s, 3H), 2.46 (s, 3H); **^{13}C NMR** (126 MHz, $CDCl_3$) δ 162.2, 148.8, 144.0, 139.2, 136.7, 134.9, 134.2, 129.6, 129.3, 128.3, 128.1, 126.1, 121.8, 40.8, 38.8, 21.5; **HRMS** (ESI) calcd. for $C_{20}H_{21}N_2O_2S$ [M+H]: 353.1324; found 353.1326.

N-(6-(methoxy(phenyl)methyl)pyridin-3-yl)-N,4-dimethylbenzenesulfonamide (35a)

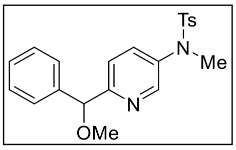

White solid (0.028 mmol, 10.7 mg, 28% yield); **^{1}H NMR** (500 MHz, $CDCl_3$) δ = 8.17 (d, *J* = 2.5, 1H), 7.53 (dd, *J* = 8.5, 2.6, 1H), 7.46 (d, *J* = 8.5, 1H), 7.40 (dd, *J* = 7.6, 4.0, 4H), 7.34 (t, *J* = 7.5, 2H), 7.28 (d, *J* = 7.3, 1H), 7.23 (d, *J* = 8.0, 2H), 5.35 (s, 1H), 3.42 (s, 3H), 3.15 (s, 3H), 2.41 (s, 3H); **^{13}C NMR** (126 MHz, $CDCl_3$) δ = 160.11, 146.38, 144.02, 140.37, 136.99, 135.08, 133.13, 129.62, 128.54, 127.89, 127.73, 126.99, 120.37, 86.03, 57.18, 37.81, 21.53. **HRMS** (ESI) calcd. for $C_{21}H_{23}N_2O_3S$ [M+H]: 383.1429; found 383.1428.

***N*-(2-(methoxy(phenyl)methyl)pyridin-3-yl)-*N*,4-dimethylbenzenesulfonamide (35b)**

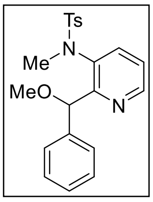

Purified by semi-preparative HPLC in hexanes/AcOEt gradient; white solid (0.28 mmol, 10.7 mg, 28% yield); **^{1}H NMR** (major rotamer) (400 MHz, $CDCl_3$) δ 8.70 (d, *J* = 4.6 Hz, 1H), 7.54 (dd, *J* = 32.6, 7.8 Hz, 5H), 7.32 (dd, *J* = 13.8, 7.6 Hz, 6H), 7.06 (dd, *J* = 8.0, 4.7 Hz, 1H), 6.72 (d, *J* = 7.9 Hz, 1H), 6.17 (s, 1H), 3.49 (s, 4H), 2.61 (s, 3H), 2.47 (s, 4H); **^{13}C NMR** (all signals are listed)(101 MHz, $CDCl_3$) δ 161.2, 149.4, 144.3, 140.1, 135.9, 134.6, 133.8, 129.8, 128.5, 128.3, 128.0, 122.4, 81.2, 80.5, 57.3, 53.5, 38.7, 21.7.

^{1}H NMR (major rotamer) (400 MHz, CD_3OD) δ 8.57 (d, *J* = 3.8 Hz, 1H), 7.57 (d, *J* = 8.0 Hz, 2H), 7.42 (dd, *J* = 11.6, 7.6 Hz, 5H), 7.36 – 7.29 (m, 2H), 7.30 – 7.25 (m, 1H), 7.25 – 7.18 (m, 1H), 6.90 (d, *J* = 8.0 Hz, 1H), 6.20 (s, 1H), 3.43 (s, 3H), 2.61 (s, 3H), 2.47 (s, 3H); **^{13}C NMR** (all signals are listed) (101 MHz, CD_3OD) δ 161.8, 149.6, 146.1, 141.1, 137.7, 136.9, 134.7, 131.0, 129.5, 129.4, 129.3, 129.2, 124.4, 82.3, 57.4, 39.0, 21.5, 21.5; **HRMS** (ESI) calcd. for $C_{21}H_{22}N_2O_3SNa$ [M+Na]: 405.1249; obtained 405.1250.

^{1}H NMR (500 MHz, DMSO-d_6, **353K**) δ 8.54 (d, *J* = 4.5 Hz, 1H), 7.67 – 7.60 (m, 2H), 7.47 (d, *J* = 8.1 Hz, 2H), 7.42 (d, *J* = 7.6 Hz, 2H), 7.32 (dd, *J* = 8.3, 6.7 Hz, 2H), 7.29 – 7.23 (m, 2H), 7.11 (d, *J* = 7.6 Hz, 1H), 5.98 (s, 1H), 3.32 (s, 3H), 3.05 (s, 3H), 2.45 (s, 3H), **^{13}C NMR** (126 MHz, DMSO-d_6, **353K**) δ 168.5, 157.8, 153.1, 149.0, 145.5, 144.2, 143.4, 138.9, 136.8, 136.3, 132.2, 89.1, 65.7, 48.1, 30.0.

4.4. Scale-up protocol

Reaction set-up

Photochemical reactions were irradiated with two Kessil lamps (maximum at 390 nm, 40 W when 100% power was applied), placed at opposite sites (**Supplementary Figure 14**) with cooling by fans ($T_{reaction}$ ~30°C).

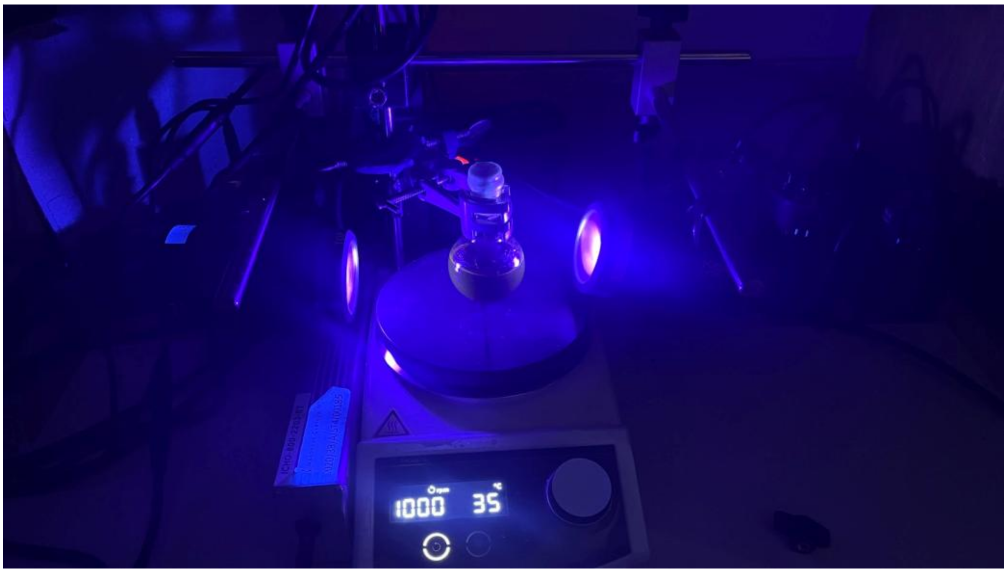

Supplementary Figure 14. *Photoreactor setup (390 nm, 20% power).*

Reaction protocol

Zincke imine **2** (0.25 mmol), **Py-salt** (0.375 mmol), $Ir(ppy)_3$ (3.4 mg, ~0.05 mmol, 2 mol%) were placed in a round bottom flask and MeCN (20 ml) and DMSO (20 ml) were added through the septum. The reaction mixture was placed in ultrasound bath and degassed by bubbling argon through the solution for 15 min. The flask was

then mounted to the photoreactor and irradiated with violet light (2 x 8 W) for 24 h maintaining a temperature of around 30 ° C with a dedicated cooling system. Then a saturated solution of NH_4OAc in anhydrous ethanol was added (10 ml) and the reaction was heated at to 65 °C for 2 h. DMSO and an excess of NH_4OAc were removed by extraction (AcOEt/ H_2O). The organic phase was dried over anhydrous sodium sulphate and evaporated with silica gel (dry load for the preparation of the sample for flash chromatography). The pure products were isolated by flash chromatography on hexanes/ AcOEt gradient (white solid, mixture of regioisomers ~5:1, 0.21 mmol, 69.3 mg, 82%).

4.5. One-pot procedure

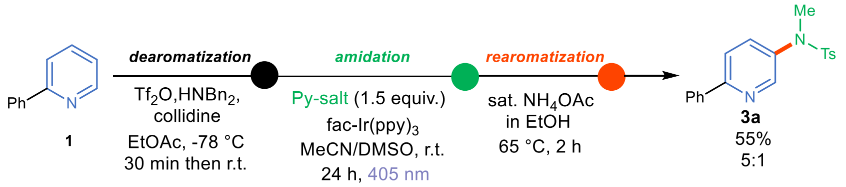

In the round bottom flask 2-phenylpyridine (**1**, 0.3 mmol) was dissolved in anhydrous AcOEt (3 ml) and cooled to -78 °C (acetone/dry ice bath) followed by the addition of trifluoromethanesulfonic anhydride (0.3 mmol) and stirred at -78 °C for 1 h. Then, a solution of dibenzylamine (0.36 mmol, 1.2 equiv.) in anhydrous AcOEt (0.5 ml) was added dropwise and stirring was continued for another 30 min at -78 °C. The reaction mixture was removed from the cryogenic bath and allowed to stir while the mixture was heated to room temperature.

To each of six 10 ml closed cup vials, **Py-salt** (0.075 mmol) and $Ir(ppy)_3$ (0.067 mg, ~10 μmol, 2 mol%) were added. The reaction mixture containing Zincke imine (from the previous step) was equally divided between six vials and to each DMSO (3 ml) and MeCN (4 ml) were added. The reaction mixture was placed in ultrasound bath and degassed by bubbling argon through the solution for 15 min. Vials were then transferred to the photoreactor and irradiated with violet light (2.4 W) for 24 h maintaining a temperature between 0 °C to 5 °C with a dedicated cooling system. After the indicated time, a saturated solution of NH_4OAc in anhydrous ethanol was added (2 ml) to each vial and the reactions were heated up to 65 °C for 2 h. An excess of NH_4OAc from the combined reaction mixtures was removed by extraction (AcOEt/ H_2O). The organic phase was dried over anhydrous sodium sulphate and evaporated with silica gel (dry load for the preparation of the sample for flash chromatography). The pure products were isolated by flash chromatography in a hexanes/AcOEt gradient (95:5 to 70:30) (white solid, mixture of regioisomers ~5:1, 0.165 mmol, 55.8 mg, 55% total yield).

4.6 Other functionalizations

General protocol for deprotection of the NTs (according to Rodríguez et al. protocol)[38]

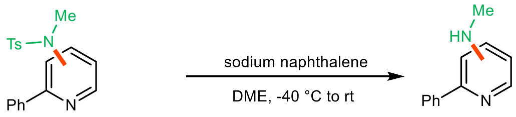

To the solution of compounds **3a** or **3b** (38 mg, 0.11 mmol) in anhydrous DME (2 ml), sodium naphthalene (0.8 ml; 0.5 M solution prepared from 516 mg of naphthalene and 71 mg of sodium in 6 ml of DME) was added dropwise at -40 °C. The reaction mixture was stirred for 2 h at this temperature and then for 30 min at room temperature. The reaction was quenched with water (5 ml) and extracted with AcOEt (3 x 5 ml). The combined organic phases were washed with water (10 ml) and brine (10 ml) and dried with anhydrous sodium sulphate. The desired product was purified by chromatography in a DCM / AcOEt gradient (from 0% AcOEt to 50% AcOEt) (easily identified a shiny spot on TLC under 365 nm irradiation).

***N*-methyl-6-phenylpyridin-3-amine (36)**

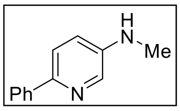

82% yield (17 mg, 0.092 mmol), white solid; **^{1}H NMR** (400 MHz, $CDCl_3$) δ 8.12 (dd, *J* = 2.9, 0.7 Hz, 1H), 7.94 – 7.86 (m, 2H), 7.58 (dd, *J* = 8.6, 0.7 Hz, 1H), 7.47 – 7.38 (m, 2H), 7.35 – 7.27 (m, 1H), 6.95 (dd, *J* = 8.6, 2.9 Hz, 1H), 3.83 (s, 1H), 2.91 (s, 3H); **^{13}C NMR** (101 MHz, $CDCl_3$) δ 146.6, 144.0, 139.6, 135.4, 128.5, 127.4, 125.8, 120.6, 118.8, 30.4; **HRMS** (ESI) calcd. for $C_{12}H_{13}N_2$ [M+H]: 185.1079; obtained 185.1078.

***N*-methyl-2-phenylpyridin-3-amine (37)**

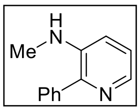

Reaction was started from 19 mg of **3b**; 78% yield (8.1 mg, 0.044 mmol), white solid; **^{1}H NMR** (400 MHz, $CDCl_3$) δ 8.05 (dd, *J* = 4.7, 1.4 Hz, 1H), 7.65 – 7.57 (m, 2H), 7.51 – 7.44 (m, 2H), 7.42 – 7.36 (m, 1H), 7.16 (dd, *J* = 8.2, 4.7 Hz, 1H), 6.96 (dd, *J* = 8.2, 1.4 Hz, 1H), 4.17 (s, 1H), 2.80 (d, *J* = 4.9 Hz, 3H); **^{13}C NMR** (101 MHz, $CDCl_3$) δ 142.8, 138.6, 137.8, 129.0, 128.8, 128.4, 123.3, 116.5, 30.50; **HRMS** (ESI) calcd. for $C_{12}H_{13}N_2$ [M+H] 185.1079; found 185.1083.

Preparation of the amide 38.

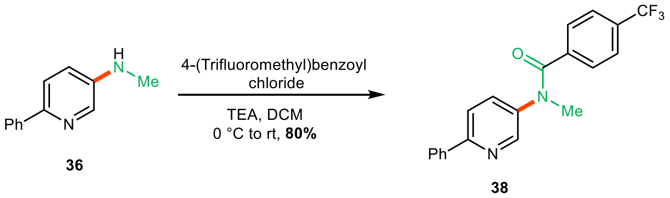

_N_-methyl-_N_-(6-phenylpyridin-3-yl)-4-(trifluoromethyl)benzamide (38)

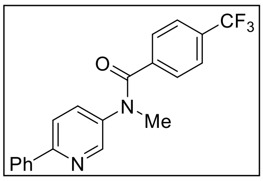

To the solution of compound **36** (17 mg, 0.092 mmol) in anhydrous DCM (0.5 ml), TEA (26 µl, 0.18 mmol) and *p*-CF_3 benzoyl chloride (14 µl, 0.092 mmol) were added at 0 °C. The reaction mixture was stirred for 30 min at 0 °C and then for 4 h at room temperature. The reaction mixture was quenched with water (5 ml) and extracted with AcOEt (3 x 5 ml). The combined organic phases were washed with water (10 ml) and brine (10 ml) and dried with anhydrous sodium sulphate. The desired product was purified by chromatography in a hexanes / AcOEt gradient (from 0% AcOEt to 20% AcOEt), yielding the product as a white solid (26.5 mg, 0.074 mmol, 80%). **^{1}H NMR** (500 MHz, $CDCl_3$) δ 8.39 (d, *J* = 2.7 Hz, 1H), 7.98 – 7.90 (m, 2H), 7.66 (d, *J* = 8.4 Hz, 1H), 7.53 – 7.39 (m, 8H), 3.56 (s, 3H); **^{13}C NMR** (126 MHz, $CDCl_3$) δ 169.3, 155.6, 147.7, 139.4, 138.8, 138.0, 134.5, (132.4, 132.1, 131.8, 131.6), 129.5, 129.1, 129.0, 126.9, (125.4, 125.4, 125.3, 125.3), 120.5, 38.57; **HRMS** (ESI) calcd. for $C_{20}H_{15}N_2O_3Na$ [M+Na] 379.1034; found 379.1031.

Preparation of *N*-oxide 39

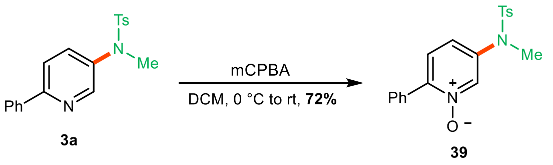

5-((*N*,4-dimethylphenyl)sulfonamido)-2-phenylpyridine 1-oxide (39)

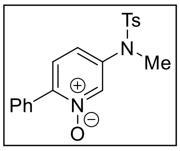

To the solution of **3a** (20 mg, 0.06 mmol) in anhydrous DCM (2 ml), mCPBA [≤77%, Sigma-Aldrich] (27 mg , ~0.12 mmol, ~2 equiv.) was added at 0 °C. The reaction mixture was stirred for 30 min at 0 °C and then for 18 h at room temperature. The reaction was directly transferred to the preprepared column (silica-gel/DCM). The product was eluted with 50% AcOEt-DCM mixture, yielding a white solid (16.2 mg, 0.046 mmol, 77%); **^{1}H NMR** (500 MHz, $CDCl_3$) δ 8.00 (d, *J* = 2.0 Hz, 1H), 7.84 – 7.77 (m, 2H), 7.57 – 7.51 (m, 2H), 7.47 (dddd, *J* = 8.8, 7.1, 5.8, 1.8 Hz, 3H), 7.43 – 7.37 (m, 2H), 7.31 (d, *J* = 8.0 Hz, 2H), 3.19 (s, 3H), 2.43 (s, 3H); **^{13}C NMR** (126 MHz, $CDCl_3$) δ 147.4, 144.9, 139.7, 137.0, 132.8, 131.9, 130.1, 129.9, 129.4, 128.5, 127.8, 126.6, 124.7, 37.6, 21.7; **HRMS** (ESI) calcd. for $C_{19}H_{19}N_2O_3S$ [M+H] 355.1116; found 355.1117.

Preparation of the methylpyridinium iodide 40

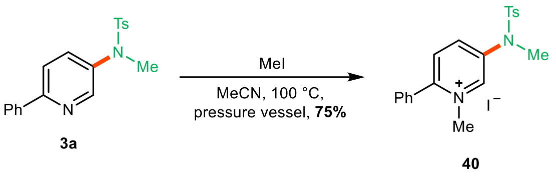

5-((*N*,4-dimethylphenyl)sulfonamido)-1-methyl-2-phenylpyridin-1-ium iodide (40)

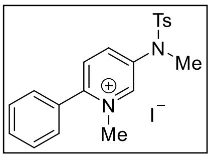

To the solution of **3a** (30 mg, 0.09 mmol) in anhydrous MeCN (1 ml) placed in a pressure vessel, MeI (110 µl, 1.77 mmol, 20 equiv.) was added. The reaction mixture was stirred for 8 h at 100 °C. After the indicated time, the reaction mixture was cooled down and solvent and an excess of methyl iodide were removed under reduced pressure. The semi-solid residue was then dissolved in the DCM / acetone mixture (2 ml, v/v 10:1) and transferred to the preprepared column (silica gel/DCM/10% acetone). The pure product was eluted with 40% acetone in the DCM mixture yielding a white solid (32 mg, 0.067 mmol, 75%); **^{1}H NMR** (600 MHz, DMSO-d_6) δ 9.21 (d, J = 2.4 Hz, 1H), 8.42 (dd, J = 8.7, 2.4 Hz, 1H), 8.04 (d, J = 8.7 Hz, 1H), 7.71 – 7.67 (m, 2H), 7.67 – 7.64 (m, 2H), 7.64 – 7.60 (m, 3H), 7.46 (d, J = 8.2 Hz, 2H), 4.12 (s, 3H), 3.21 (s, 3H), 2.41 (s, 3H); **^{13}C NMR** (151 MHz, DMSO-d_6) δ 153.0, 145.3, 143.9, 141.0, 140.2, 132.5, 131.6, 131.6, 130.7, 130.1, 129.8, 129.4, 128.3, 48.0, 37.2, 21.5; **HRMS** (ESI) for $C_{20}H_{21}N_2O_2S^+$ [M$^+$] 353.1324; found 353.1329.

4.7 Meta-meta (amination-bromination) difunctionalisation of Zincke imine

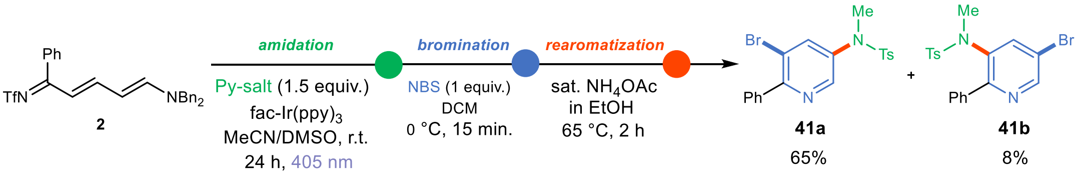

"One-pot" protocol

The reaction was carried out in 3 identical vials. For each: 2Ph-Zincke imine (**2a**) (25.07 mg, 0.05 mmol), **Py-salt** (0.075 mmol, 1.5 eq.), Ir(ppy)$_3$ (0.67 mg, ~10 µmol, 2 mol%) were placed in the closed cup vial and MeCN (4 ml) and DMSO (4 ml) were added through the septum. The reaction mixture was degassed by bubbling argon through the solution using an ultrasound bath for 15 min. The vial was transferred to the photoreaction and irradiated with violet light (2.4 W) for 24 h while maintaining a temperature between 0 °C and 5 °C with a dedicated cooling system. After the indicated time, the vial was placed in an ice bath and NBS (9.21 mg, 0.05 mol, 1.0 eq.) was added and then the ice bath was removed and the reaction was stirred at room temperature for 2 h. The saturated ammonium acetate solution in anhydrous ethanol was then added (2 ml) and the reaction was heated to 65 °C for 2 h. All vials were combined and DMSO and excess of ammonium acetate were removed by extraction (ethyl acetate/ water). The organic phase was dried over anhydrous sodium sulphate and evaporated with silica gel (dry load for the preparation of the sample for flash chromatography). Pure products were isolated by flash chromatography in the hexanes / AcOEt gradient (0-10% AcOEt).

***N*-(5-bromo-6-phenylpyridin-3-yl)-*N*,4-dimethylbenzenesulfonamide (41a)**

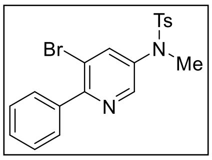

White solid (42 mg, 0.10 mmol, 65%); **^{1}H NMR** (500 MHz, $CDCl_3$) δ 8.31 (d, J = 2.3 Hz, 1H), 7.87 (d, J = 2.3 Hz, 1H), 7.71 – 7.65 (m, 2H), 7.55 – 7.50 (m, 2H), 7.49 – 7.43 (m, 3H), 7.34 – 7.28 (m, 2H), 3.21 (s, 3H), 2.44 (s, 3H); **^{13}C NMR** (126 MHz, $CDCl_3$) δ 156.4, 145.1,

144.5, 139.0, 138.8, 137.5, 133.1, 129.9, 129.4, 129.1, 128.1, 127.9, 118.8, 37.9, 21.7; **HRMS** (ESI) calcd. for $C_{19}H_{17}N_2O_2BrSNa$ [M+Na] 439.0092; found 439.0088.

***N*-(5-bromo-2-phenylpyridin-3-yl)-*N*,4-dimethylbenzenesulfonamide (41b)**

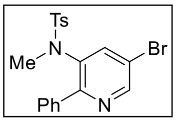

White solid (5.2 mg, 0.012 mmol, 8%; **^{1}H NMR** (400 MHz, $CDCl_3$) δ 8.70 (d, *J* = 2.1 Hz, 1H), 7.63 – 7.54 (m, 3H), 7.47 (d, *J* = 8.3 Hz, 2H), 7.41 (dd, *J* = 5.1, 2.0 Hz, 3H), 7.28 (s, 2H), 3.02 (s, 3H), 2.45 (s, 3H); **^{13}C NMR** (101 MHz, $CDCl_3$) δ 157.3, 150.1, 144.4, 138.9, 137.6, 136.8, 134.7, 129.8, 129.0, 128.9, 128.4, 128.1, 118.4, 38.8, 21.7; **HRMS** (ESI) calcd. for $C_{19}H_{17}N_2O_2BrSNa$ [M+Na] 439.0092; found 439.0093.

4.8 Unsuccessful functionalisation of Zincke imines

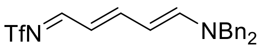

S40b

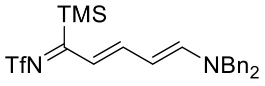

S45b

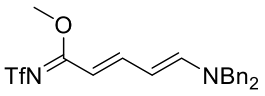

S43b

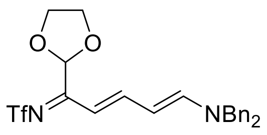

S46b

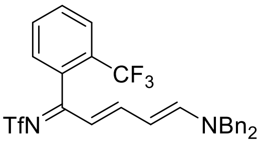

S34b

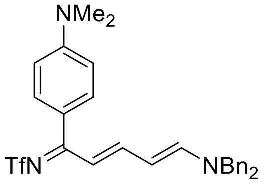

S35b

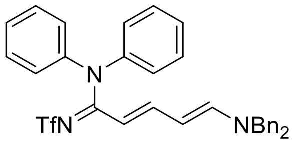

S36b

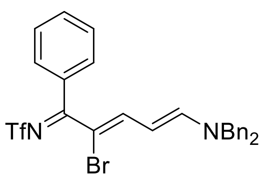

S37b

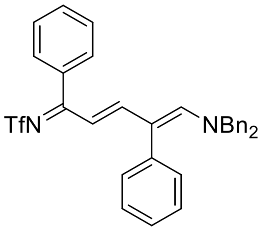

S42b

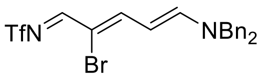

S48b

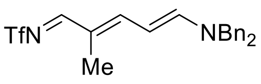

S41b

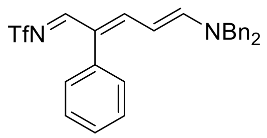

S39b

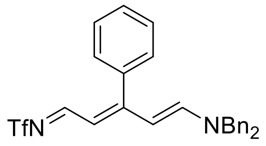

S38b

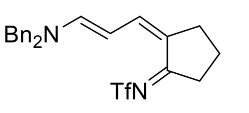

S47b

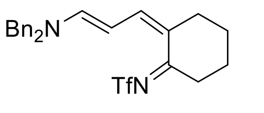

S44b

4.9 *N*-aminopyridinium salts scope

Supplementary Table 11. Scope of the *N*-aminopyridinium salts.

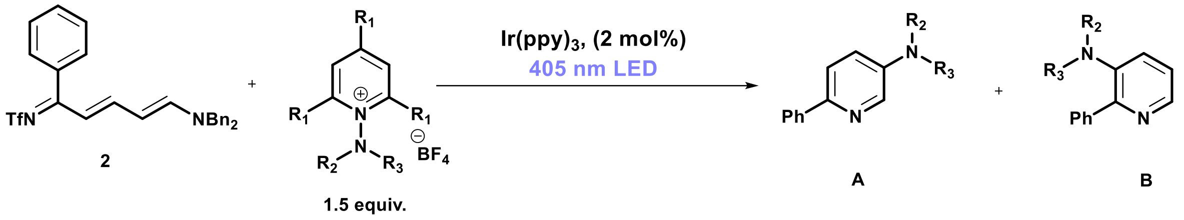

entry	R1	R2	R3	solvent	concentration [mmol/ml]	irradiation power [W]	irradiation time [h]	yield A+B [%]	ratio A to B
1	Me	Me	Ts	MeCN/DMSO (1:1)	0.006	2.4	24	99	4.8:1
2	Me	Me	Cbz	MeCN/DMSO (1:1)	0.006	2.4	24	nd	nd
3	Me	Me	Boc	MeCN/DMSO (1:1)	0.006	2.4	24	<10**	nd
4	Me	H	Ts	MeCN/DMSO (1:1)	0.006	2.4	24	nd	nd
5	H	H	Ts	MeCN/DMSO (1:1)	0.006	2.4	24	nd	nd
6	H	Me	Ts	MeCN/DMSO (1:1)	0.006	2.4	24	34%	8:1
7	H	Me	$COCF_3$	MeCN/DMSO (1:1)	0.006	2.4	24	nd	nd
8	Me	H	Boc	MeCN/DMSO (1:1)	0.006	2.4	24	nd	nd

*Entry 1-8 optimized conditions for the model reaction

**Based on ^{1}H NMR spectra of the crude reaction mixture with 2,4,6-trimethoxybenzene as an internal standard; These results should be treated as a preliminary data.

***Other variations, including solvents, concentrations, and irradiation power did not improve results

5. DFT Calculations

5.1. Computational Details

DFT calculations were carried out using the G16 programme package[26] using the ωB97xD functional[27]. Geometry optimisations and frequency calculations were computed with the Def2SVP basis set without symmetry restrictions[28,29]. The nature of all the stationary points was characterised by frequency calculations as minima (no imaginary frequencies) or transition states (one imaginary frequency). Transition states were relaxed to reactants and products, and IRC calculations were performed to further validate the connectivity. Additionally, the solvation energy was obtained from single-point calculations using ωB97xD/Def2TZVPP and the implicit solvent model (acetonitrile)[30]. The solvation free energy was then obtained by the difference between the energy calculated with the SMD model – the energy in gas phase. The standard state was corrected from 1atm to 1M by adding 1.89 kcal/mol when needed.

The potential energies were further refined using the DLPNO-CCSD(T)[31,32] method in ORCA[33-35]. Combination of Def2TZVPP and Ri-C auxiliary basis set (Def2-TZVPP/C) and RIJCOSX (Def2/J)[28,36]. The tightSCF option was also selected.

ORCA input:

```
! DLPNO-CCSD(T) Def2-TZVPP Def2-TZVPP/C Def2/J RIJCOSX GRIDX5 TightSCF
%maxcore 5000
%pal
nprocs 24
end
%scf
maxiter 2000
end
%mdci
UseFullLMP2Guess false
end
* xyz charge multiplicity structure.xyz*
```

3D representations were created using the CylView 1.0 program[37].

5.2. Comparison of free energy profiles for the regioselective addition of the N-centred radical

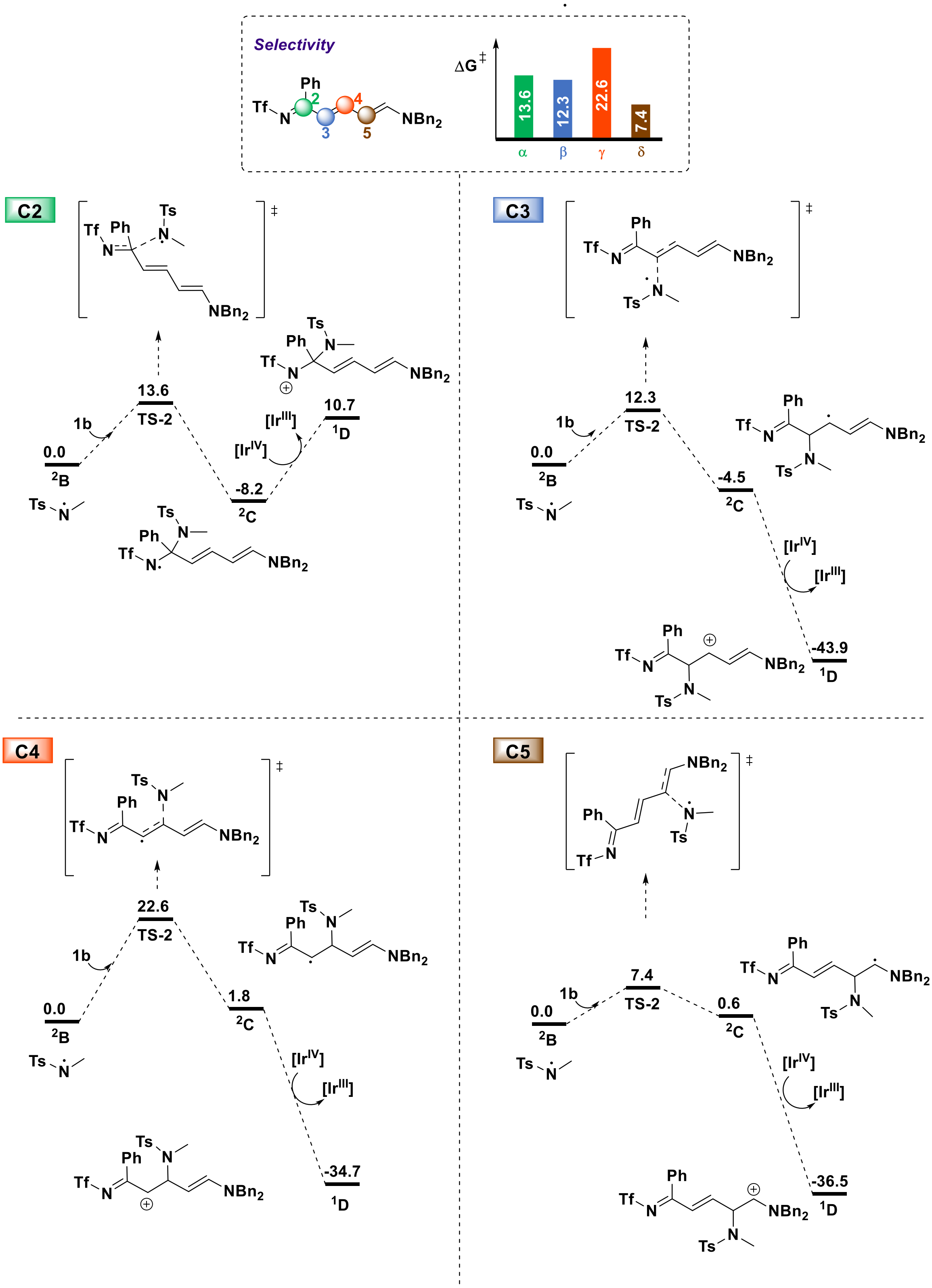

Supplementary Figure 15. *Selective radical addition and Ir-mediated oxidation for C2, C3, C4, and C5 of the Zincke intermediate. Energies in kcal/mol.*

5.3. Ring closing mechanism

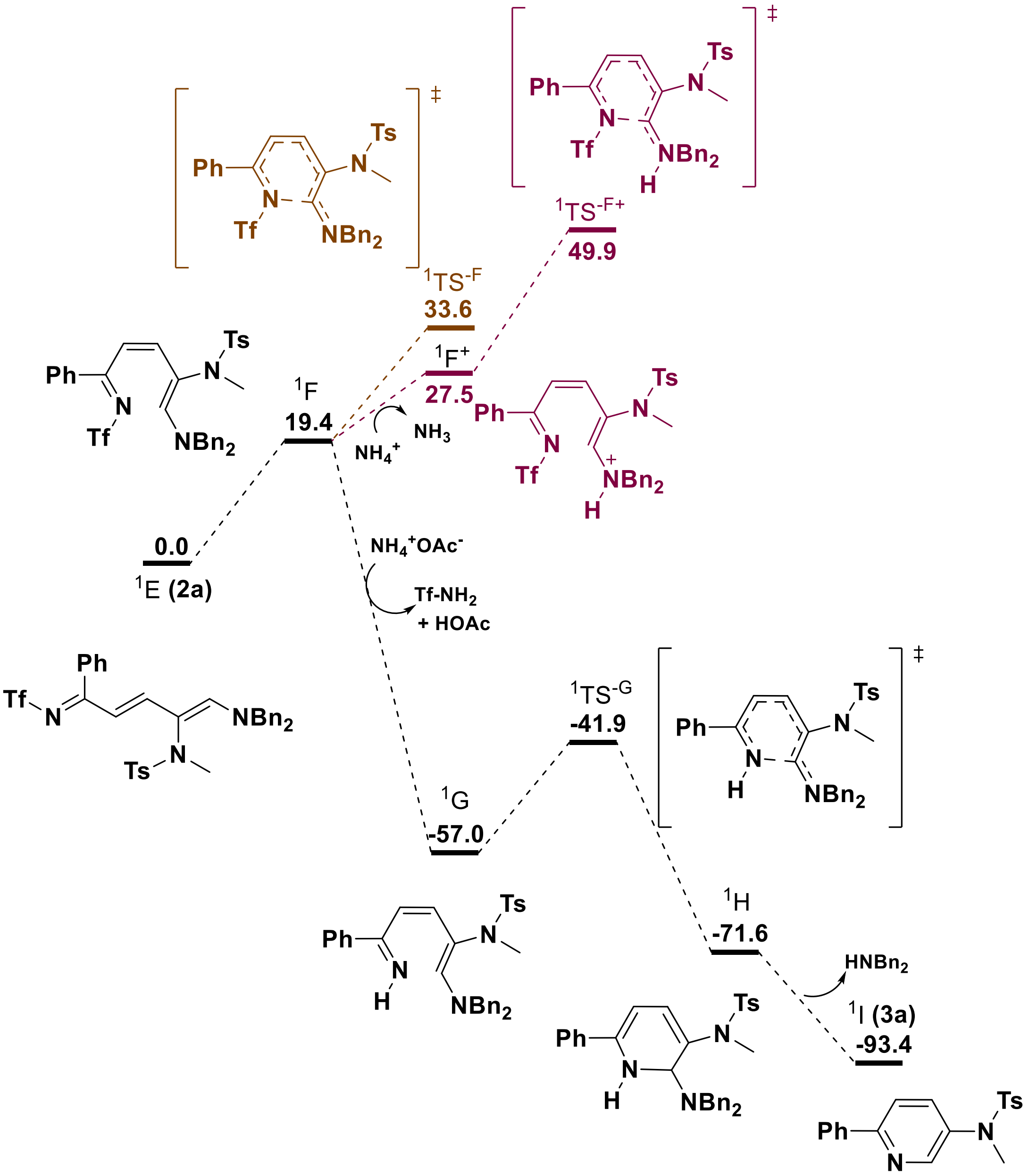

Supplementary Figure 16. *Ring closing free energy profile from 2a to 3a. Energies in kcal/mol.*

5.4. Alkyl vs Aryl selectivity

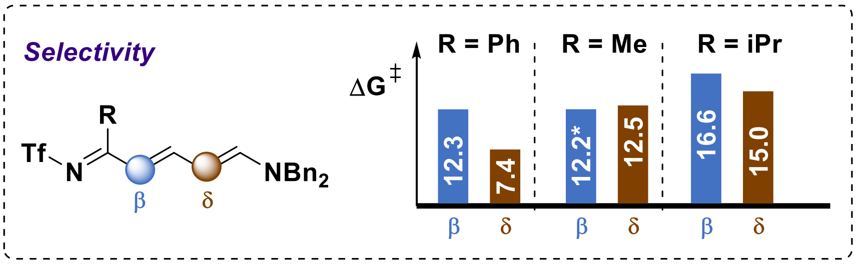

Estimated from a long-range adduct - barrierless in potential energy

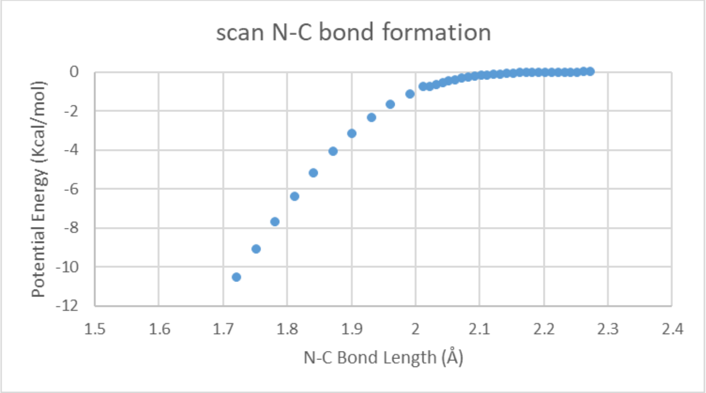

Supplementary Figure 17. *Selectivity of C3 vs C5 of Ph, Me and iPr substituted Zincke imines (top). Relaxed scan of the C-N bond for the C3 functionalization of Me-substituted Zincke imine.*

6. ^{1}H and ^{13}C NMR spectra

6.1. Pyridines

^{1}H NMR spectrum of pyridine S2a ($CDCl_3$, 298 K)

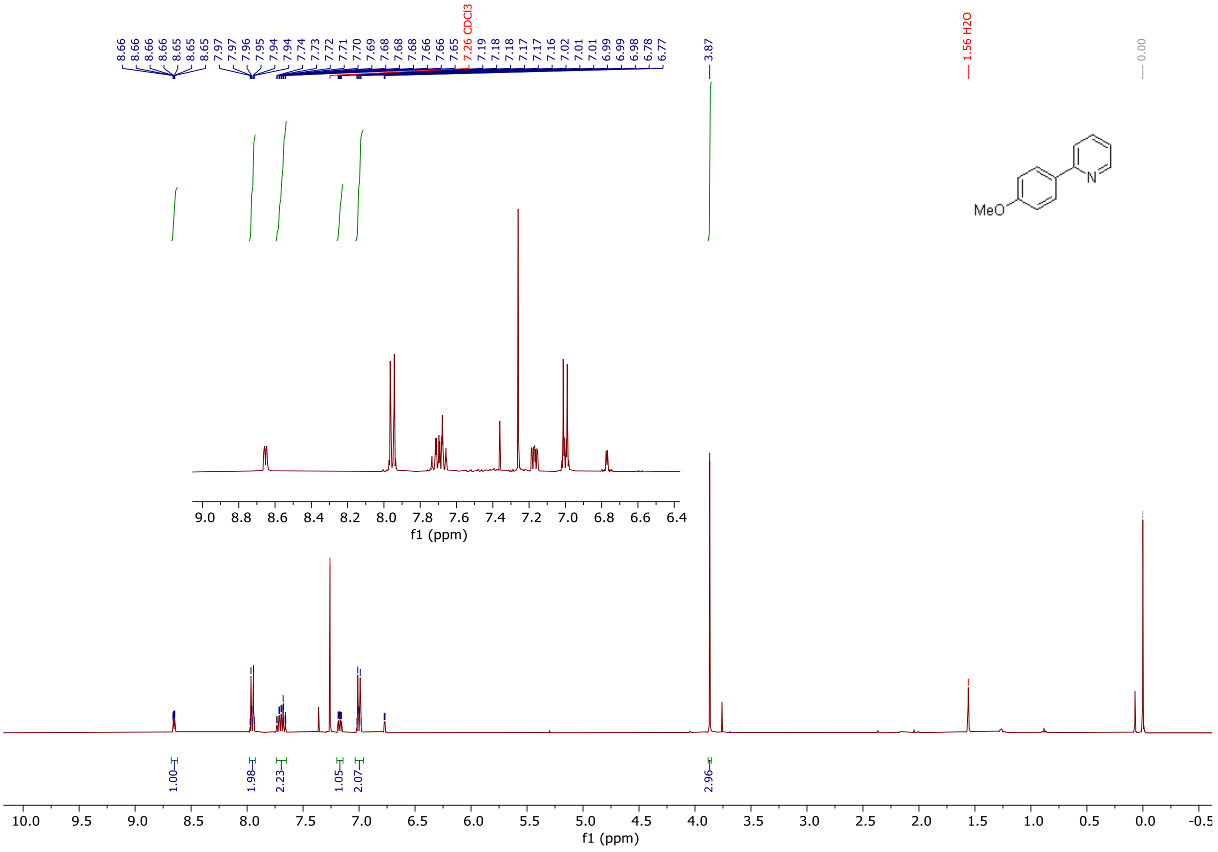

^{1}H NMR spectrum of pyridine S3a ($CDCl_3$, 298 K)

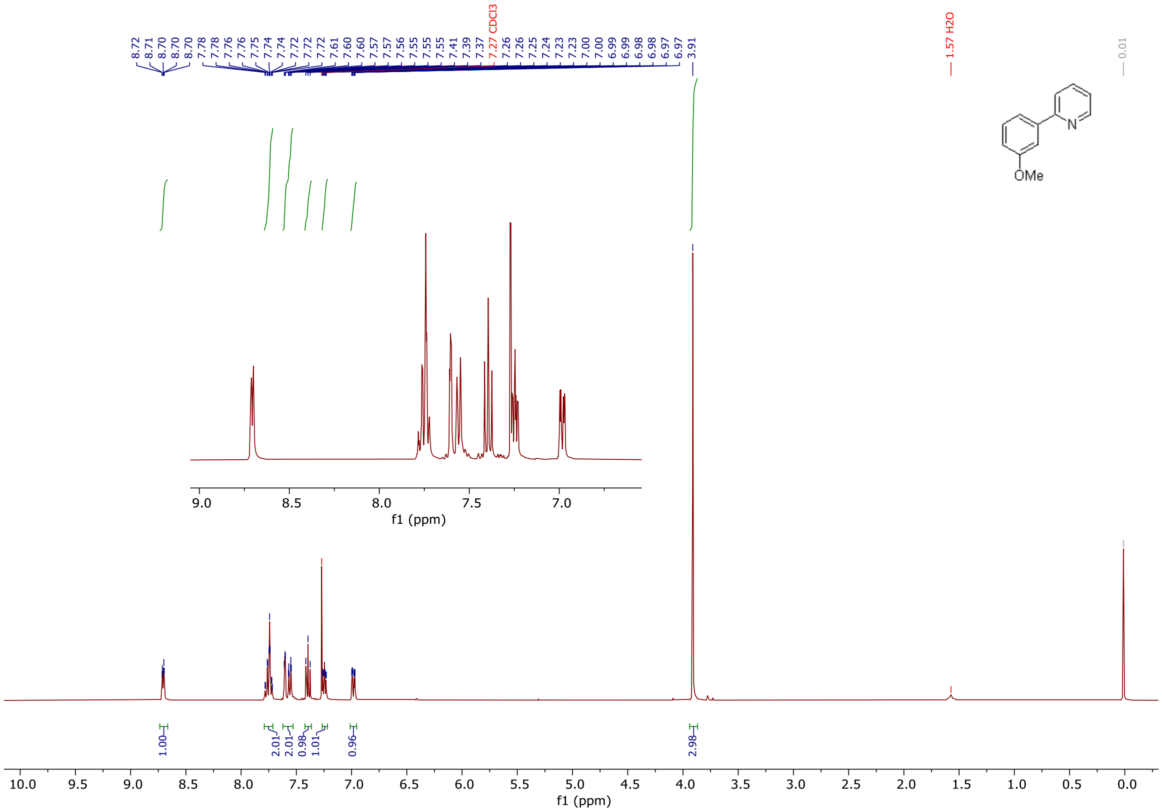

^{1}H NMR spectrum of pyridine S4a ($CDCl_3$, 298 K)

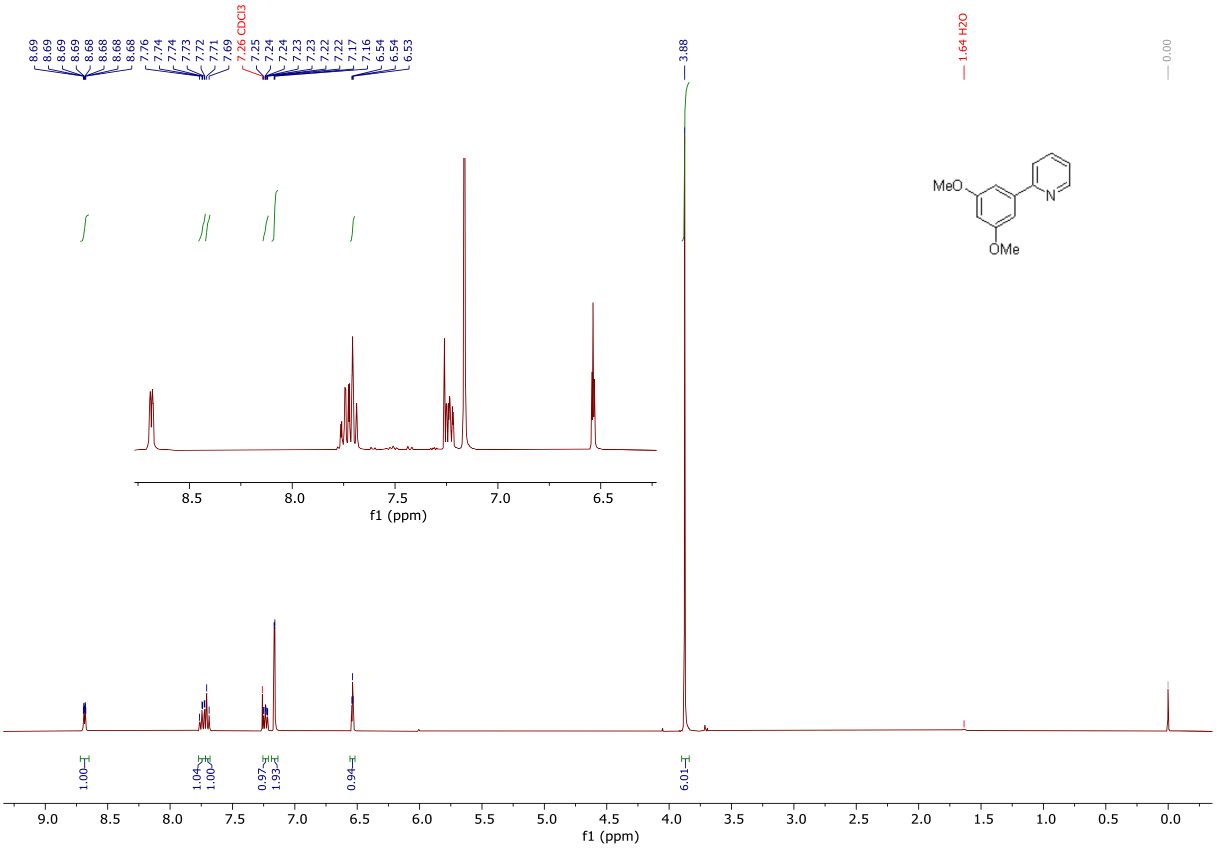

^{1}H NMR spectrum of pyridine S5a ($CDCl_3$, 298 K)

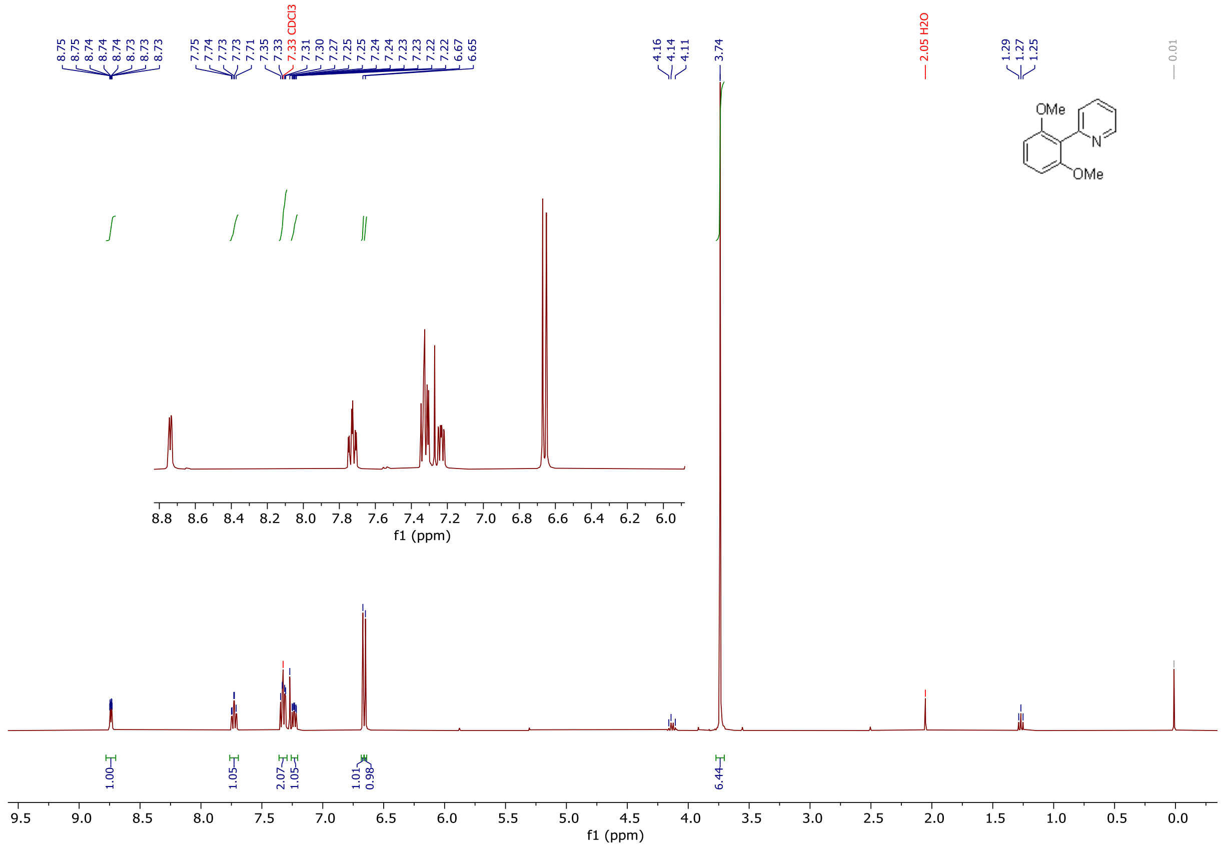

^{1}H NMR spectrum of pyridine S6a ($CDCl_3$, 298 K)

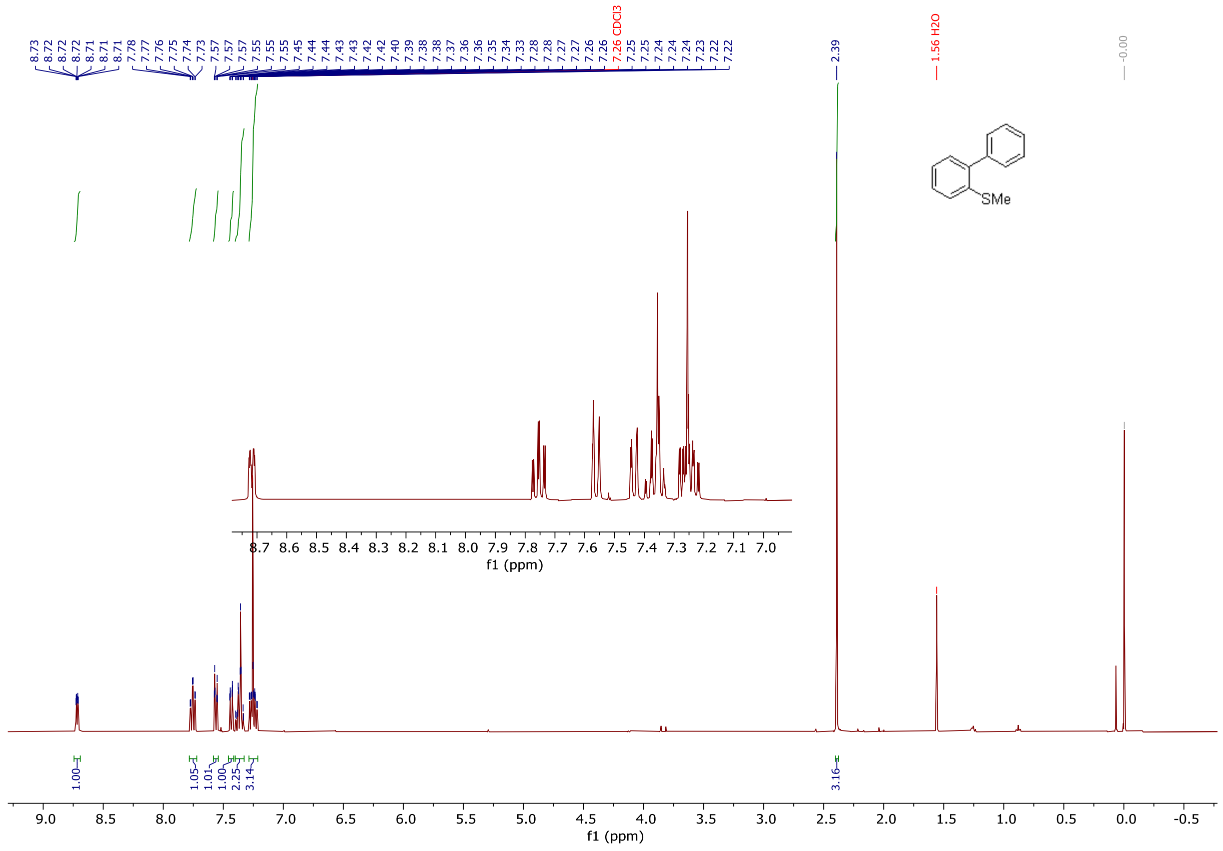

^{1}H NMR spectrum of pyridine S7a ($CDCl_3$, 298 K)

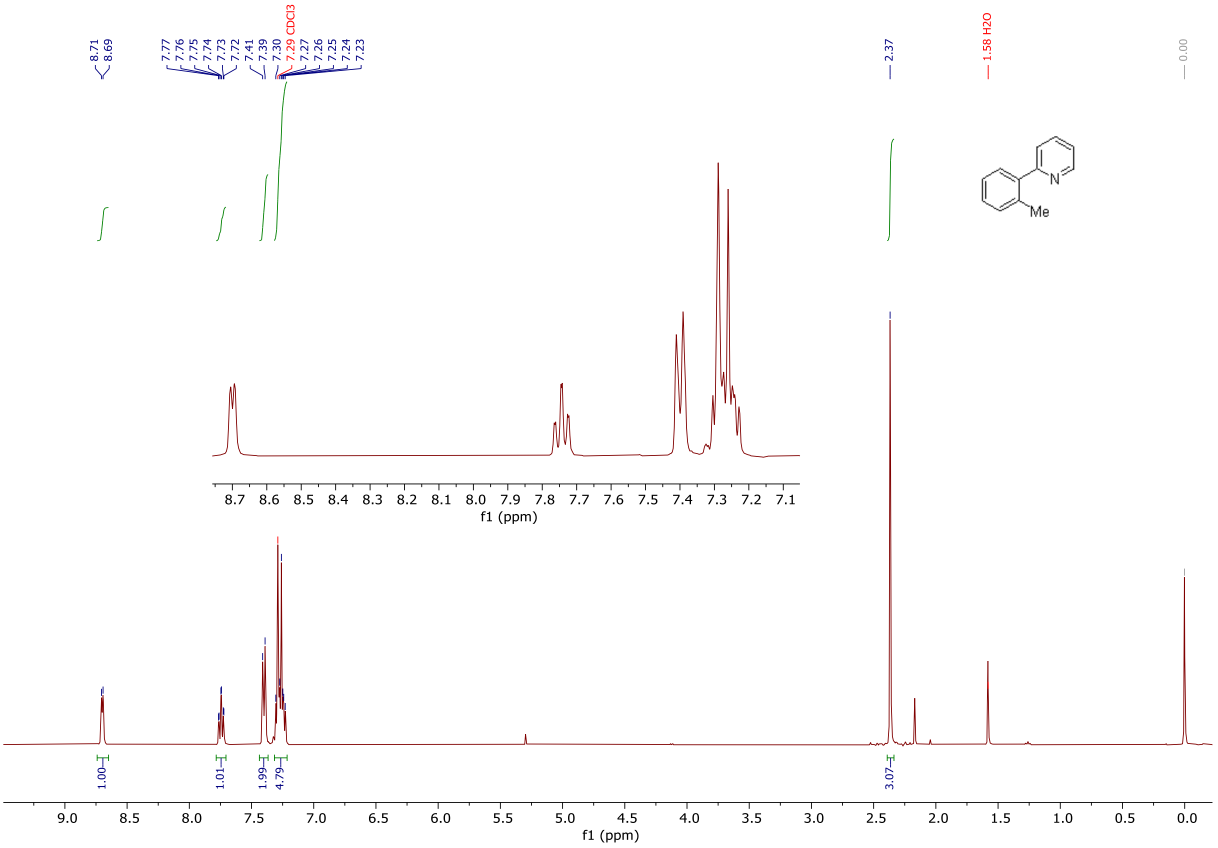

^{1}H NMR spectrum of pyridine S8a ($CDCl_3$, 298 K)

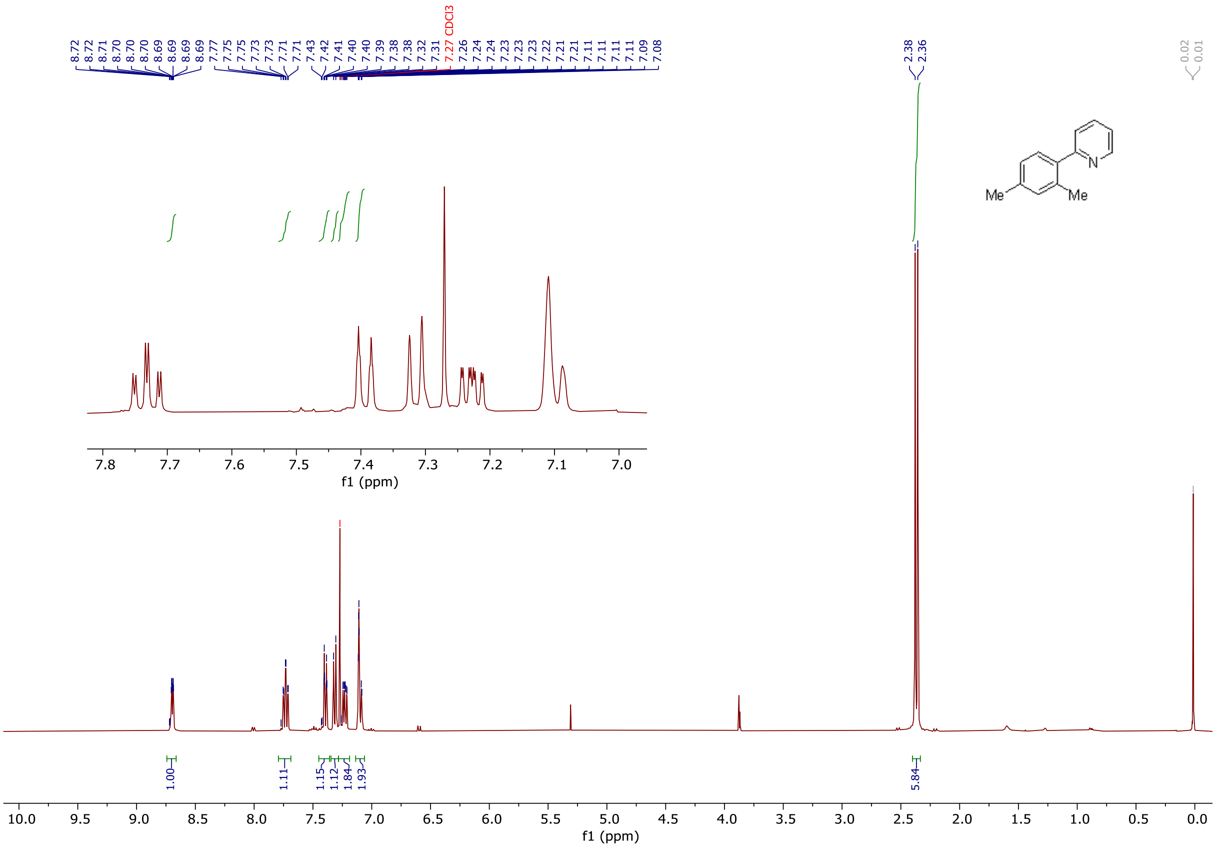

^{1}H NMR spectrum of pyridine S9a ($CDCl_3$, 298 K)

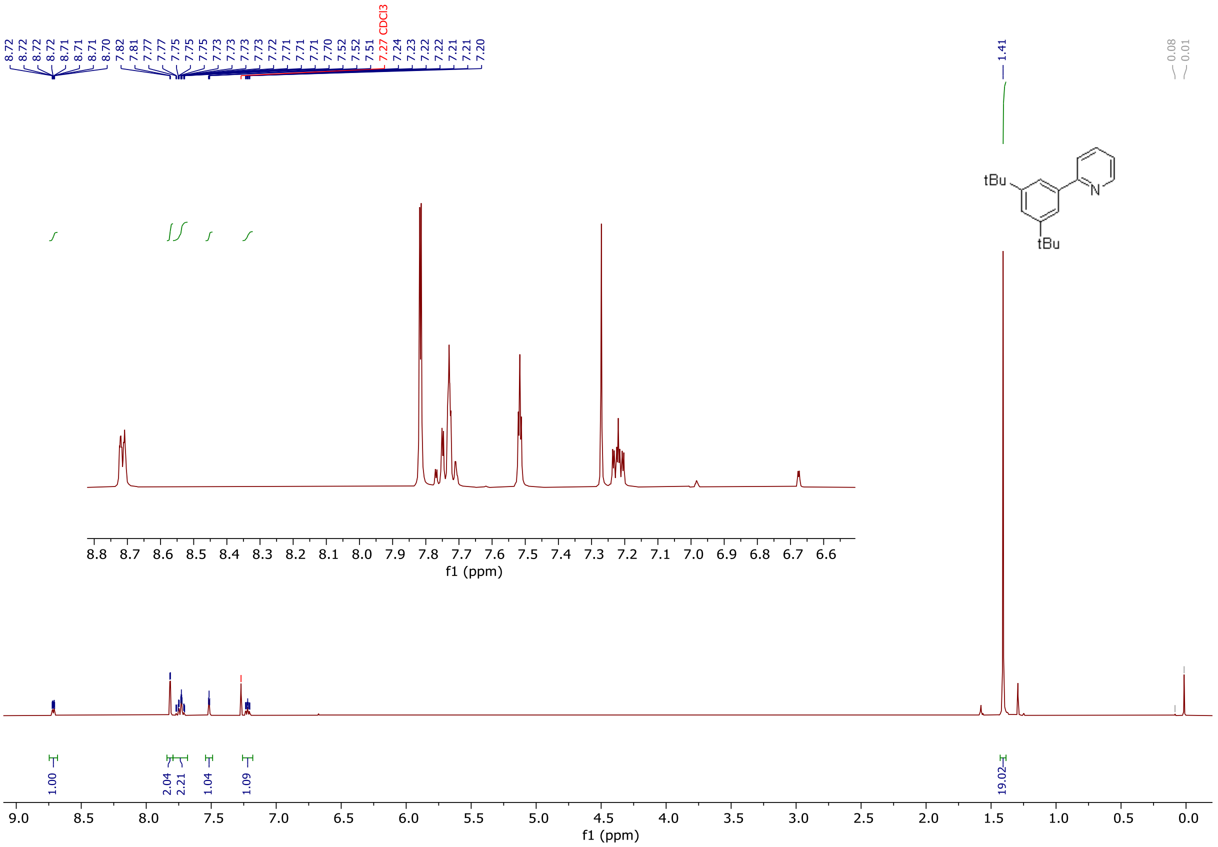

^{1}H NMR spectrum of pyridine S10a ($CDCl_3$, 298 K)

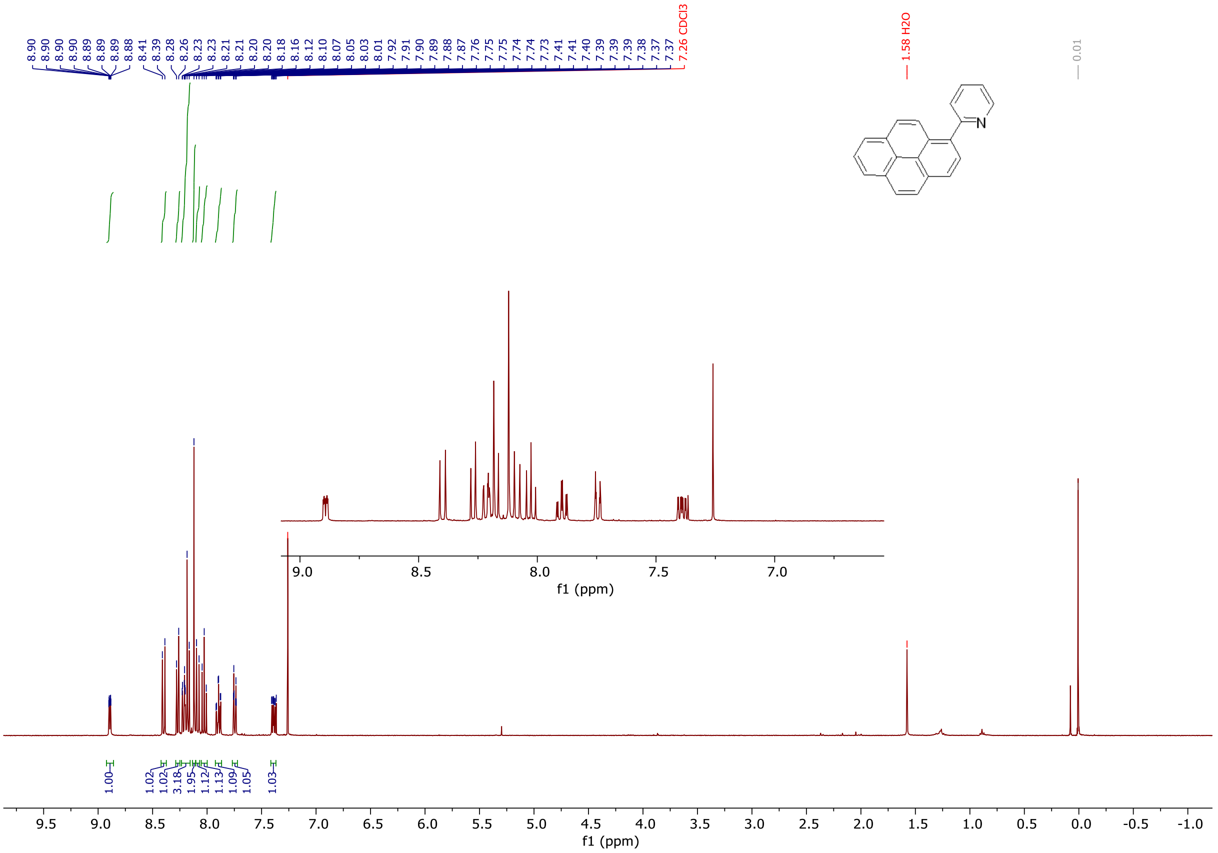

^{1}H NMR spectrum of pyridine S11a ($CDCl_3$, 298 K)

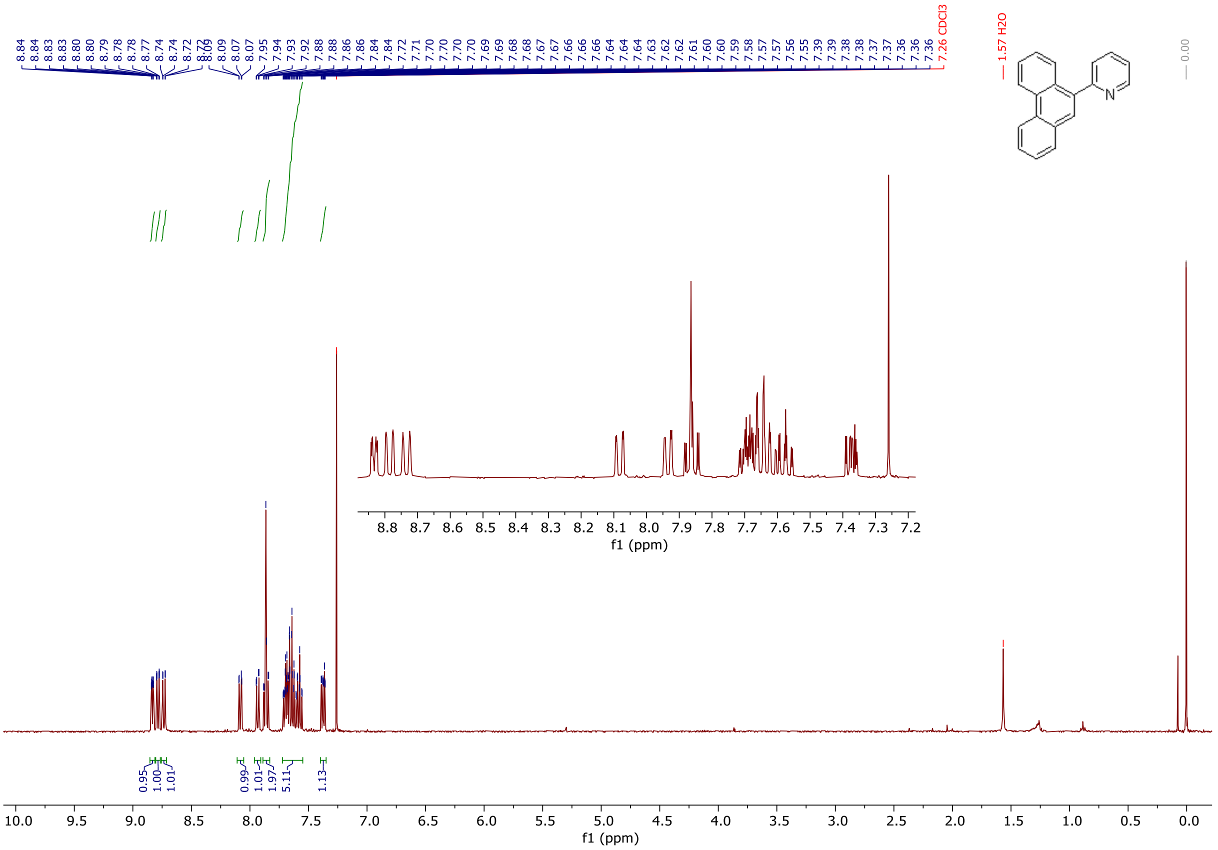

^{1}H NMR spectrum of pyridine S12a ($CDCl_3$, 298 K)

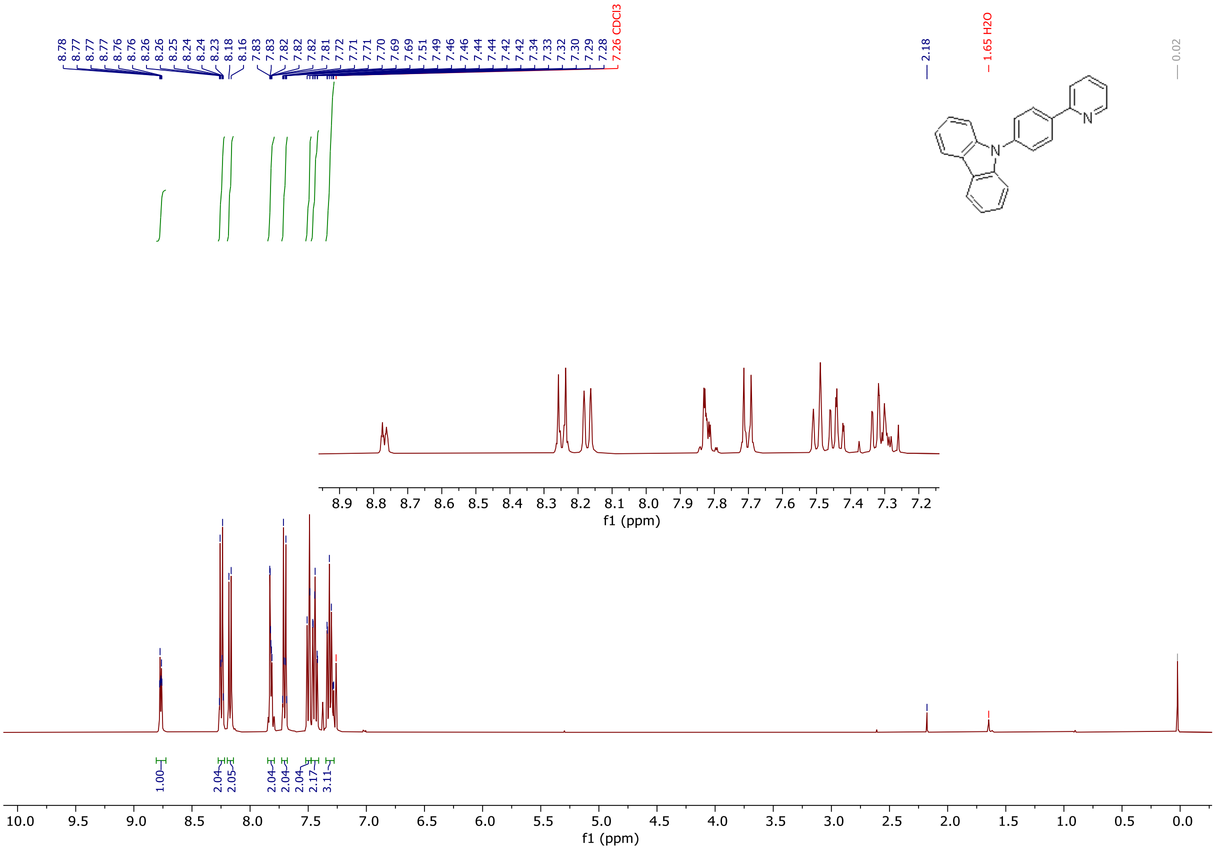

^{1}H NMR spectrum of pyridine S13a ($CDCl_3$, 298 K)

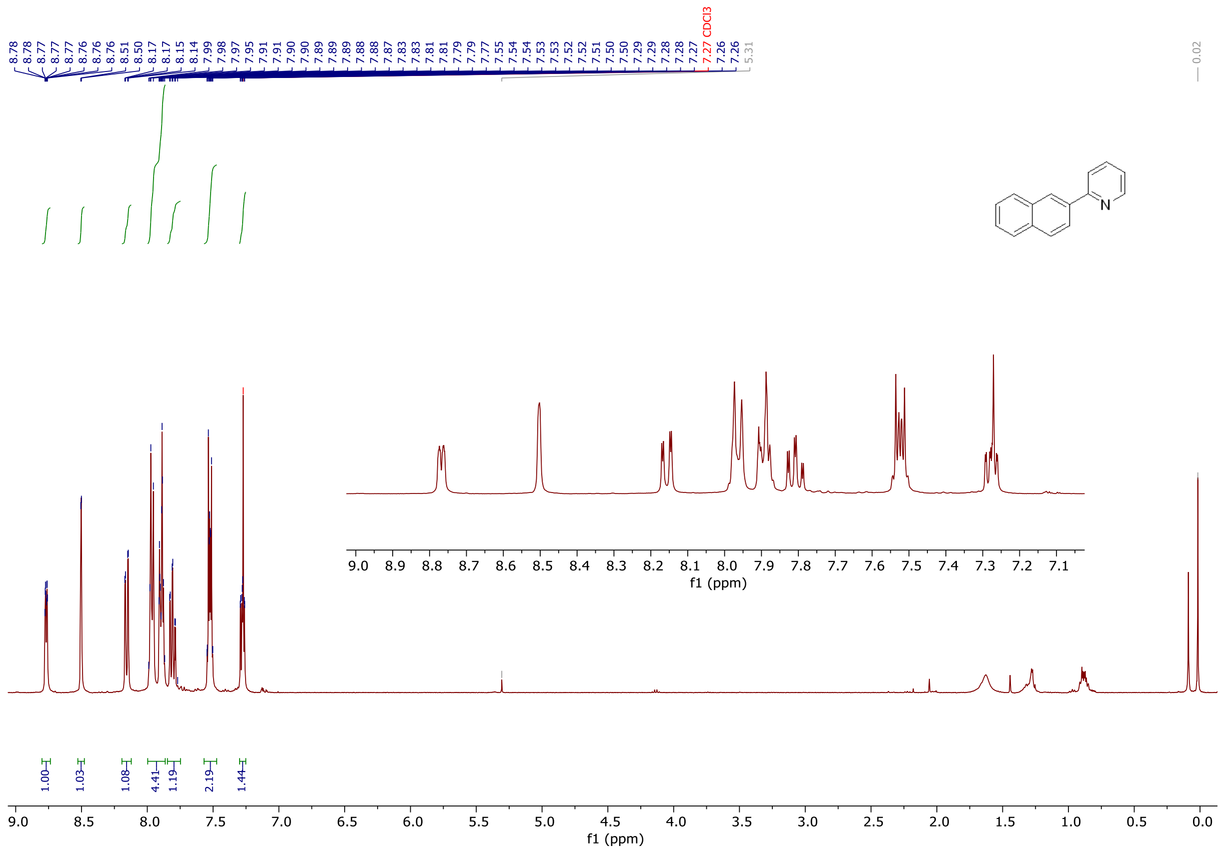

^{1}H NMR spectrum of pyridine S14a ($CDCl_3$, 298 K)

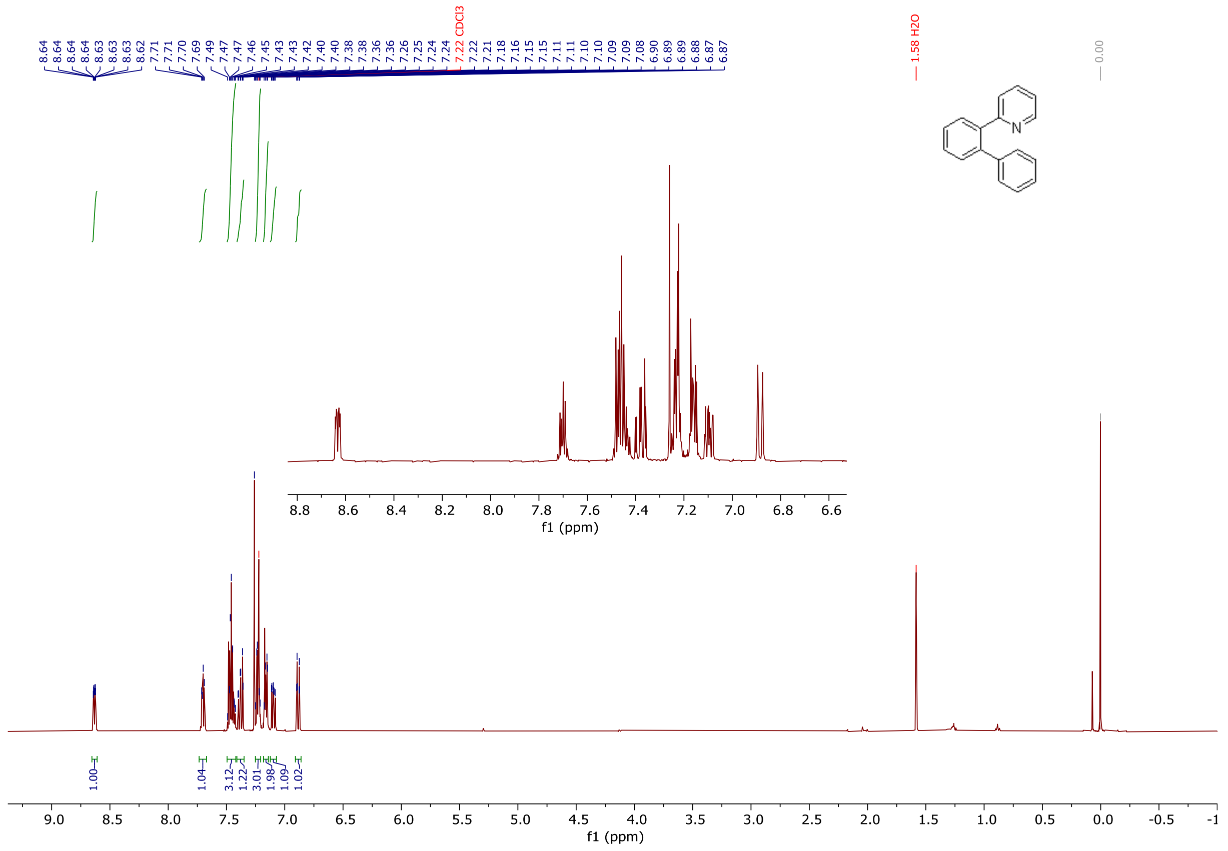

^{1}H NMR spectrum of pyridine S15a ($CDCl_3$, 298 K)

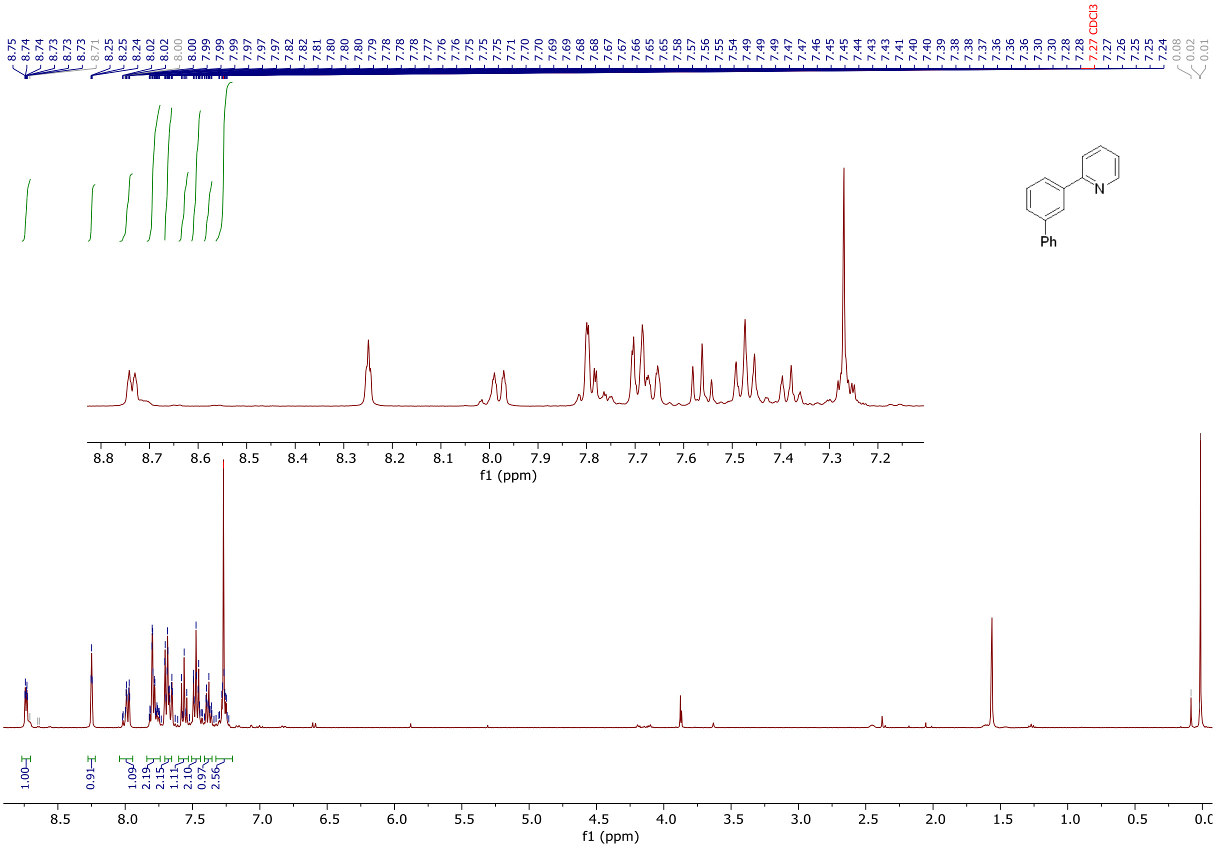

^{1}H NMR spectrum of pyridine S16a ($CDCl_3$, 298 K)

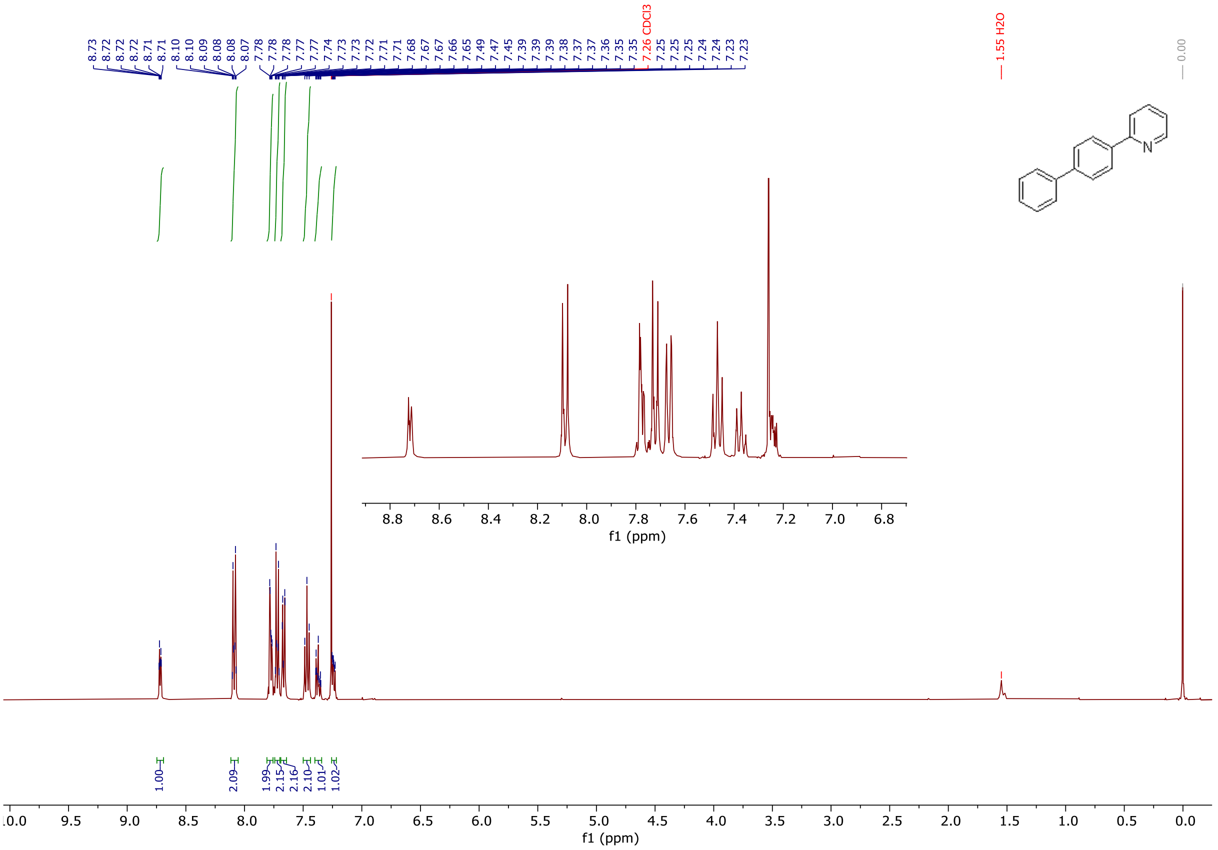

^{1}H NMR spectrum of pyridine S17a (CDCl$_3$, 298 K)

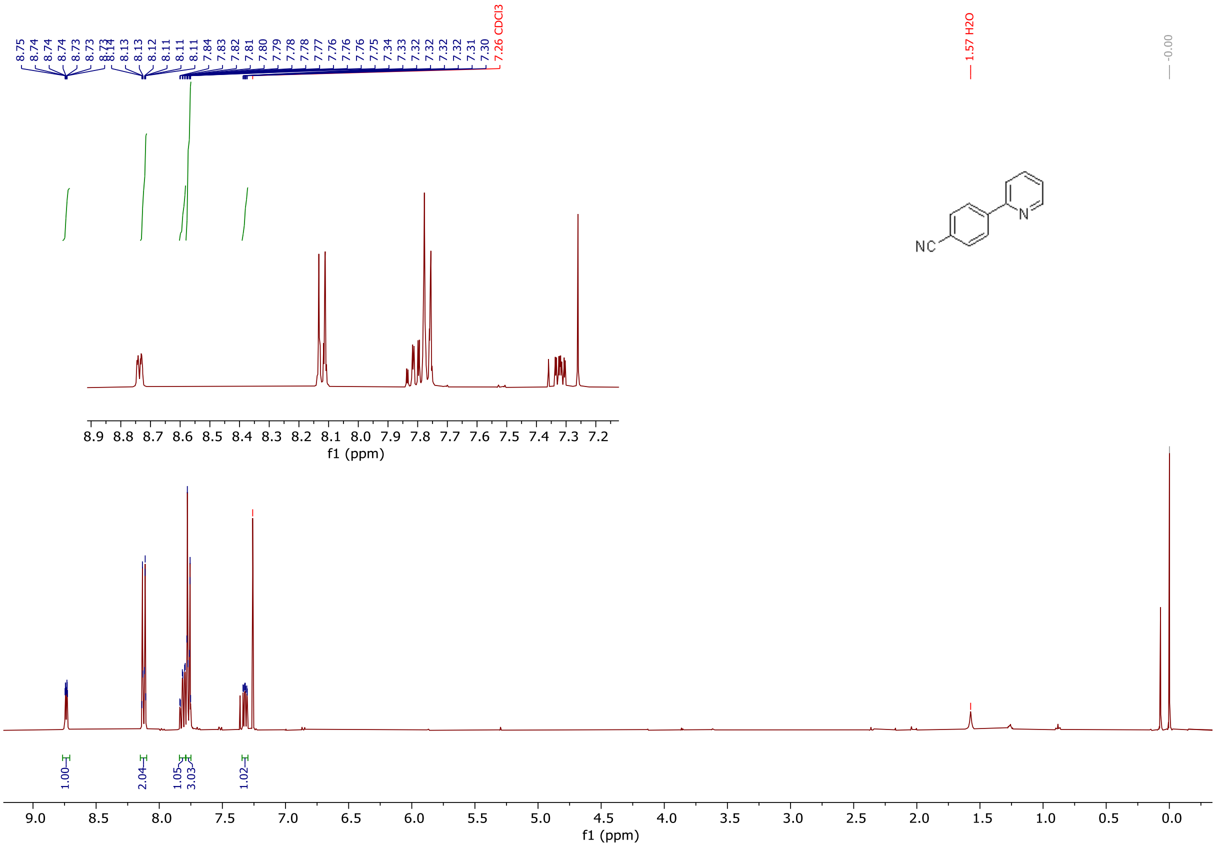

^{1}H NMR spectrum of pyridine S18a (CDCl$_3$, 298 K)

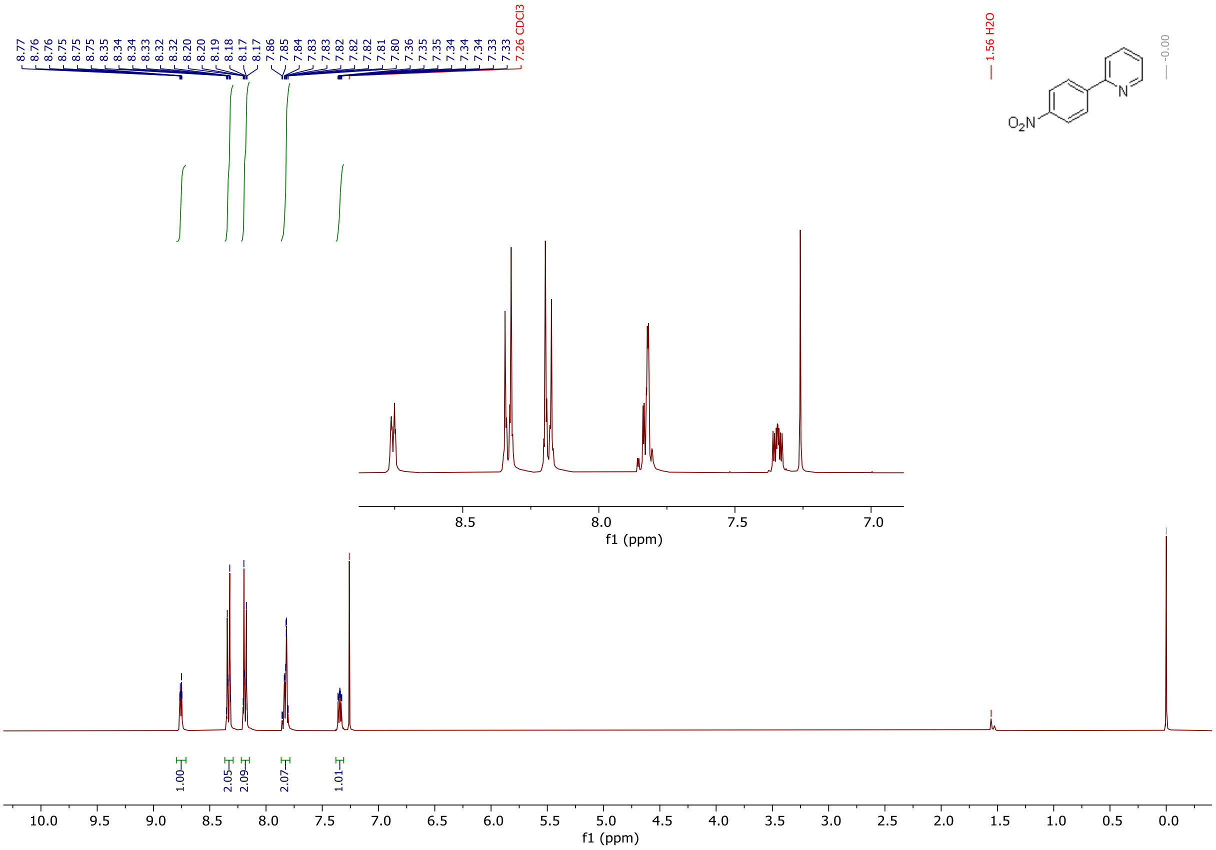

^{1}H NMR spectrum of pyridine S19a ($CDCl_3$, 298 K)

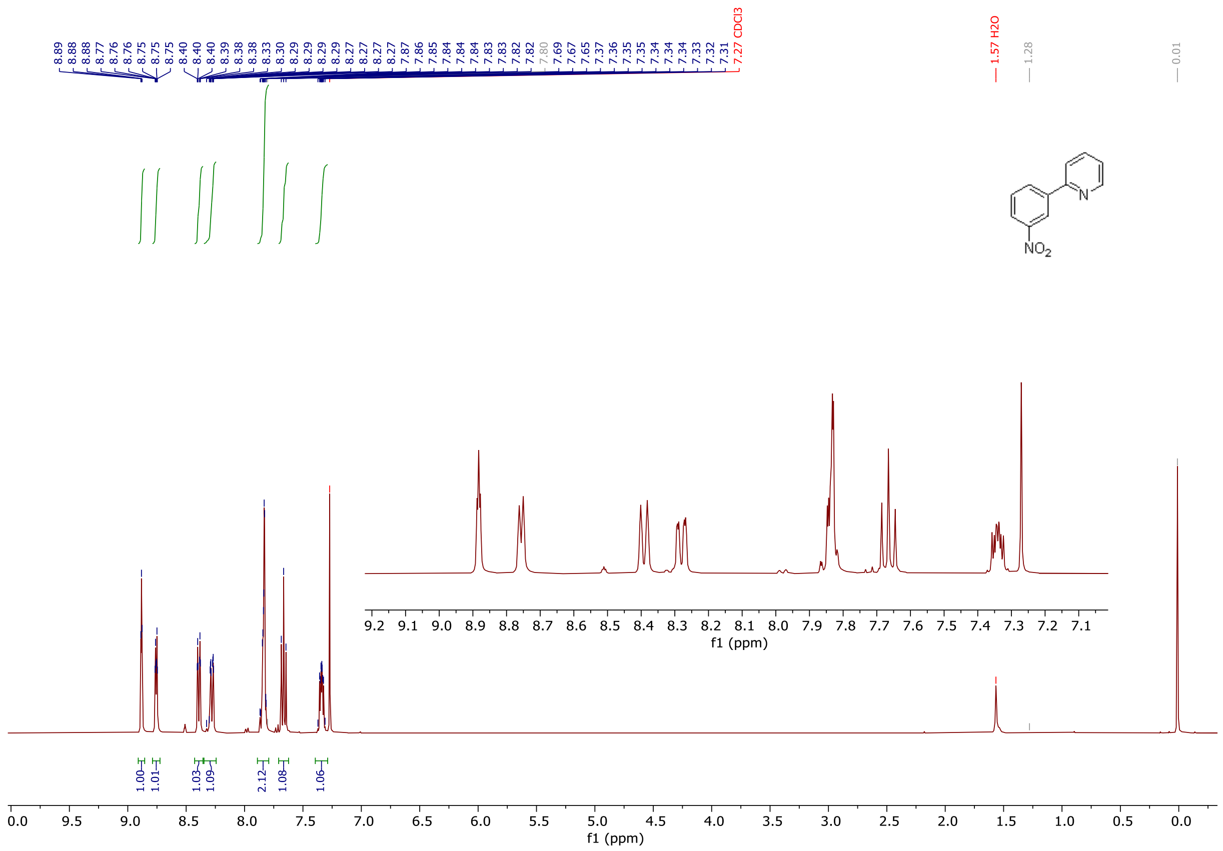

^{1}H NMR spectrum of pyridine S20a ($CDCl_3$, 298 K)

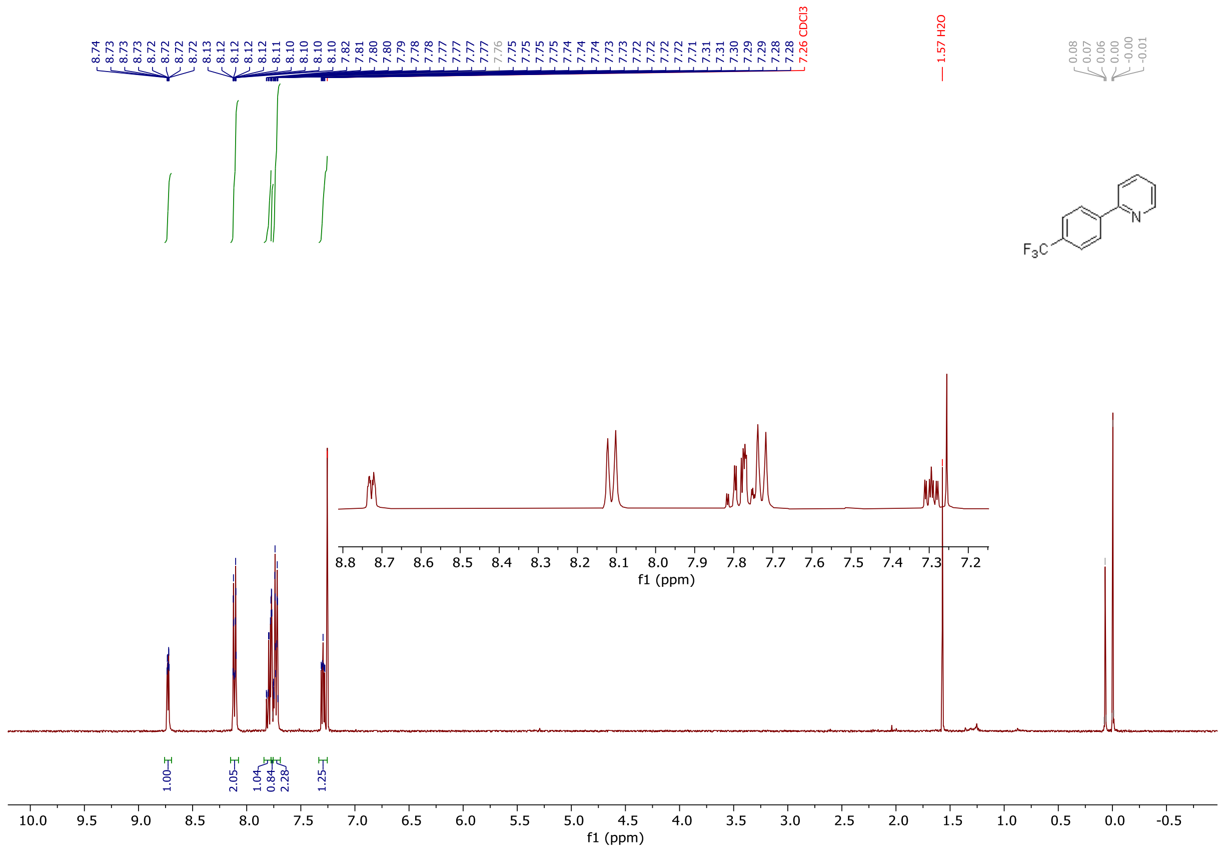

^{1}H NMR spectrum of pyridine S21a ($CDCl_3$, 298 K)

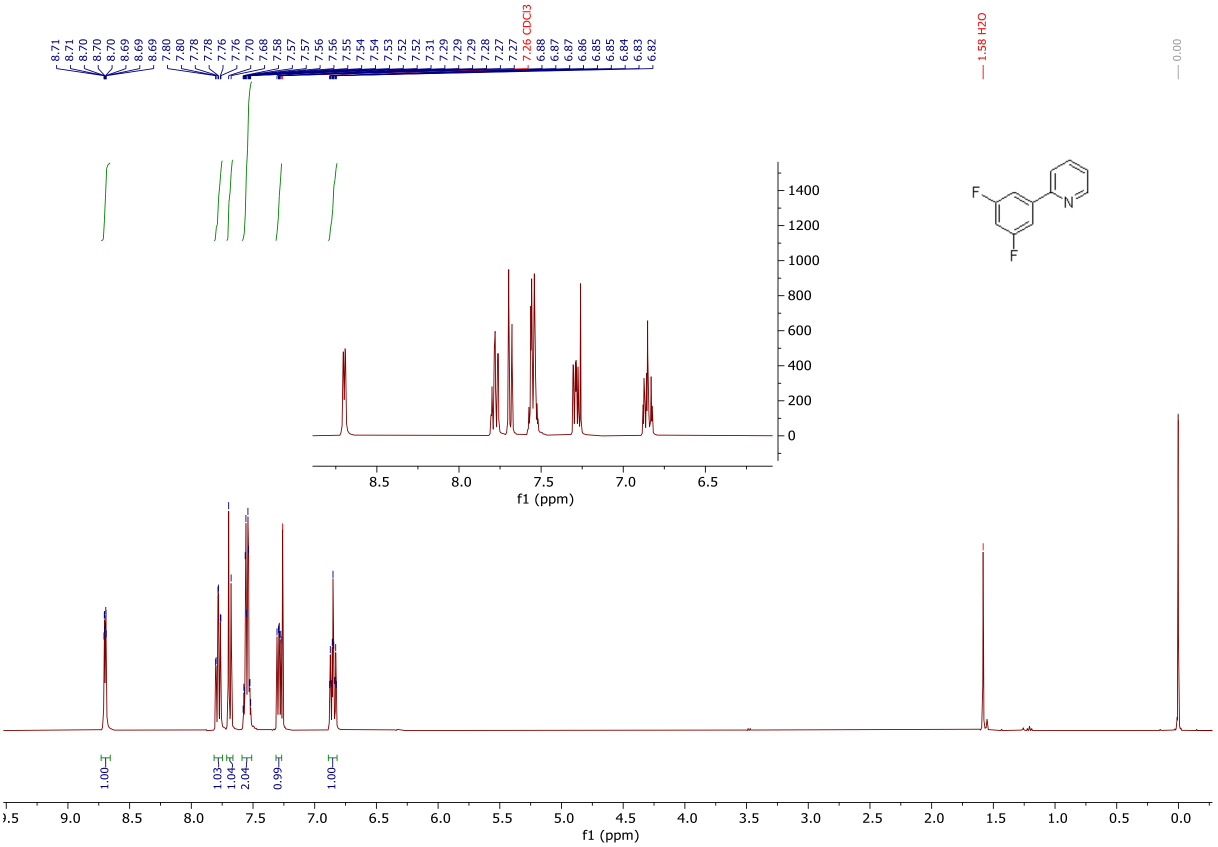

^{1}H NMR spectrum of pyridine S22a ($CDCl_3$, 298 K)

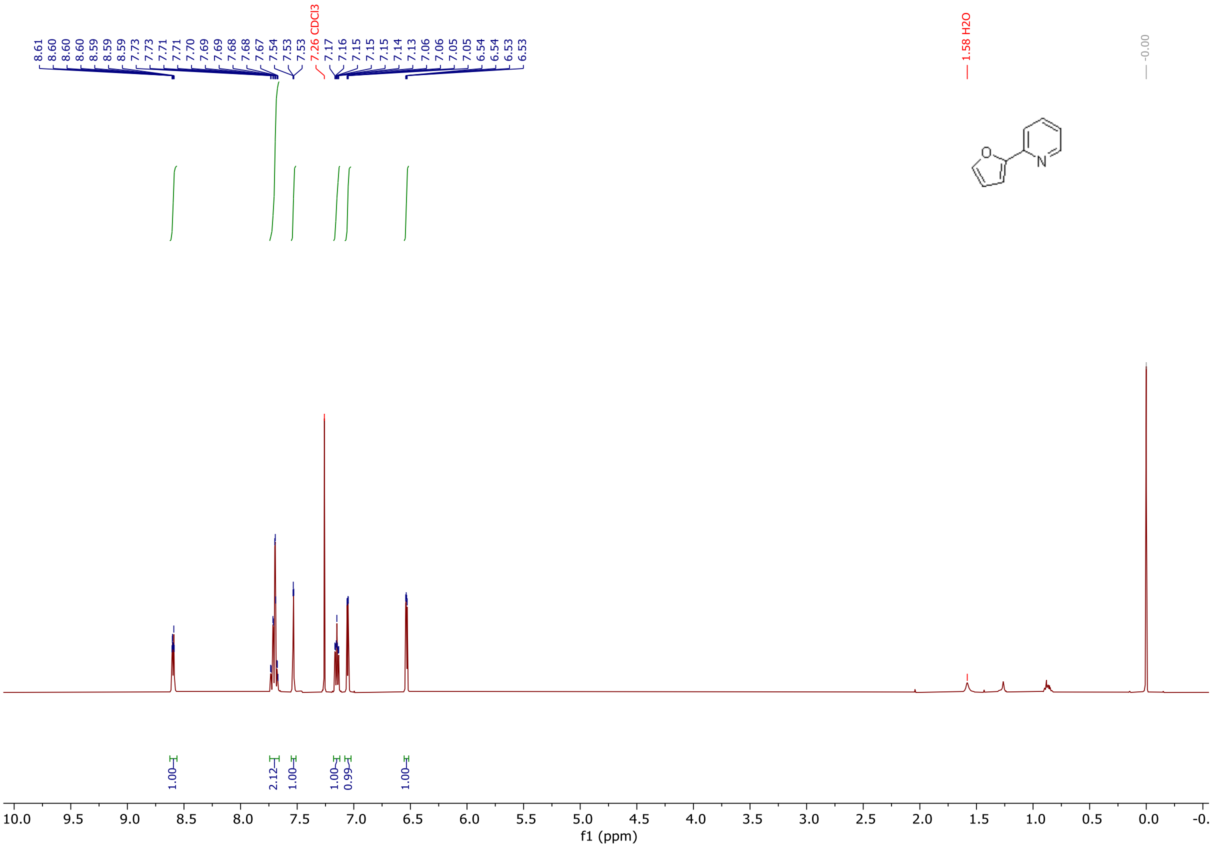

^{1}H NMR spectrum of pyridine S23a ($CDCl_3$, 298 K)

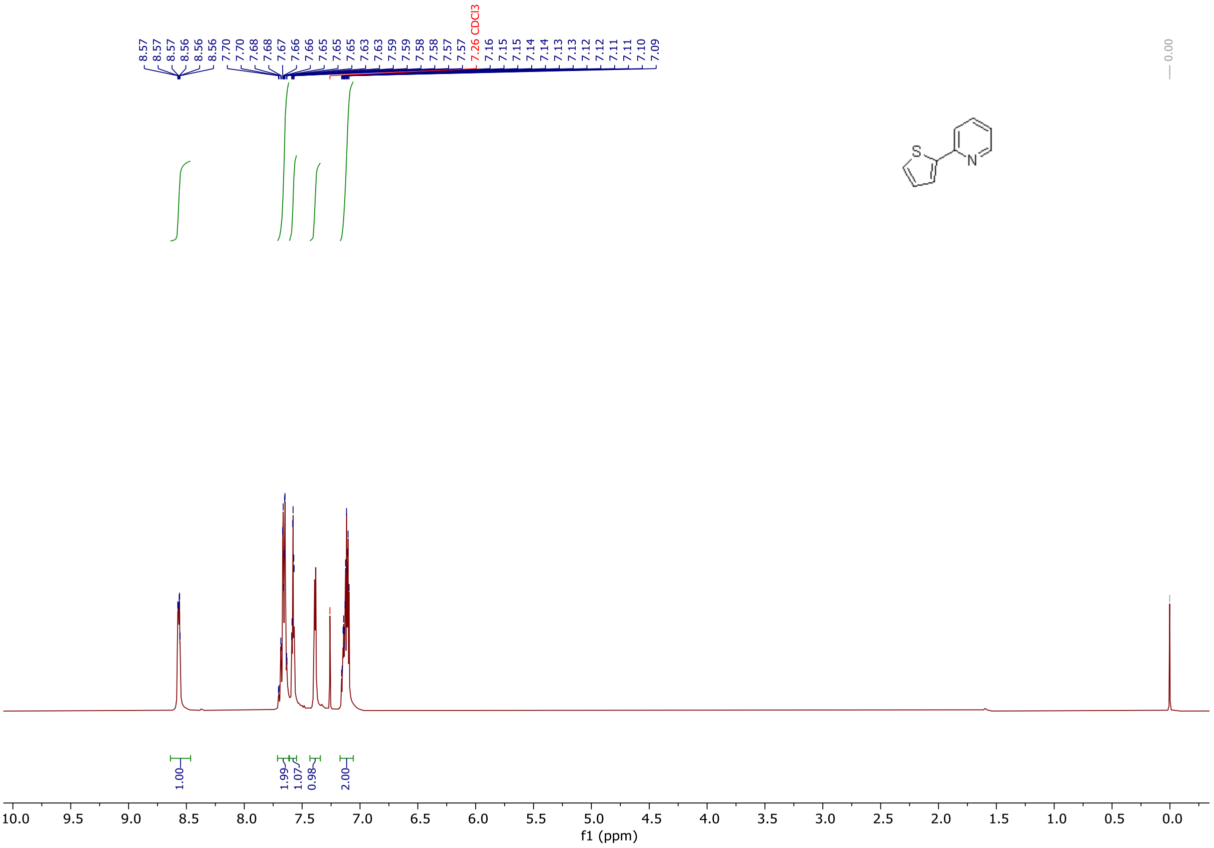

^{1}H NMR spectrum of pyridine S24a ($CDCl_3$, 298 K)

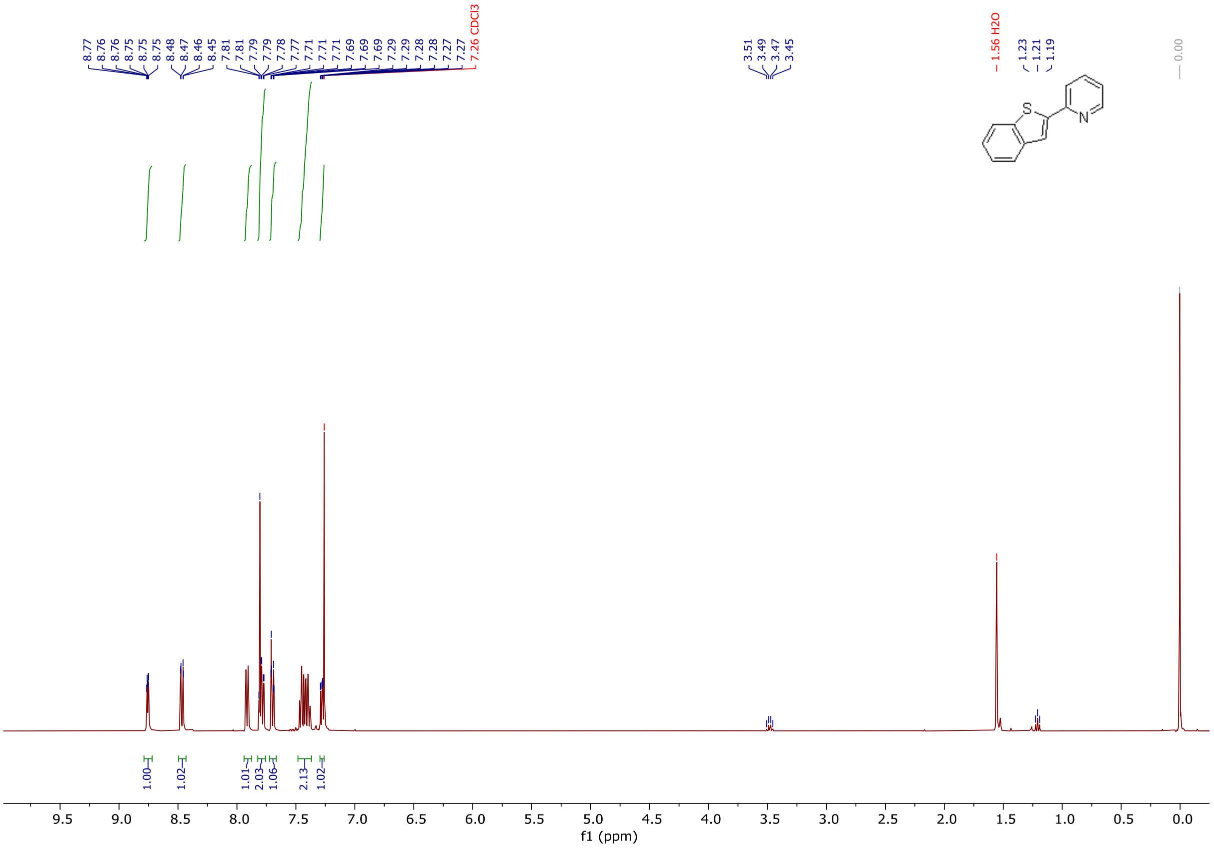

^{1}H NMR spectrum of pyridine S25a ($CDCl_3$, 298 K)

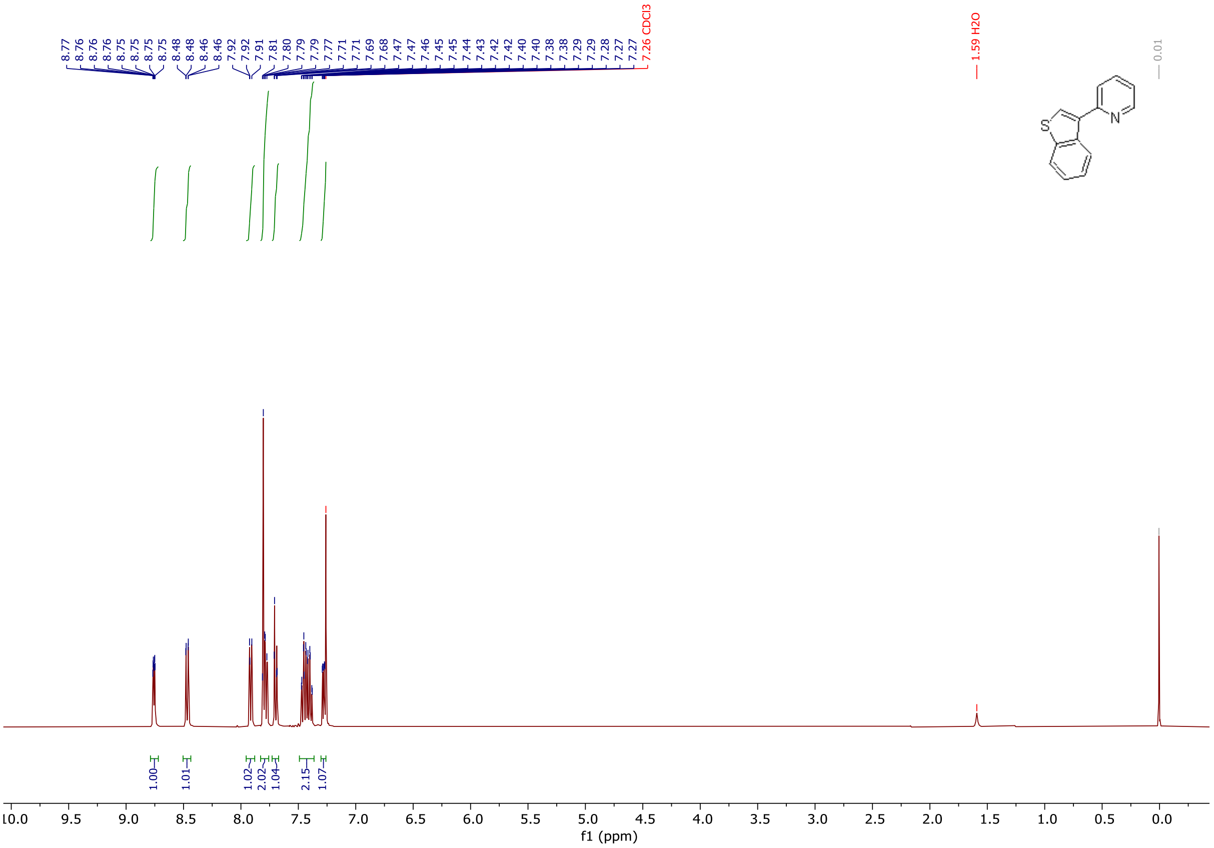

^1H NMR spectrum of pyridine S26a ($CDCl_3$, 298 K)

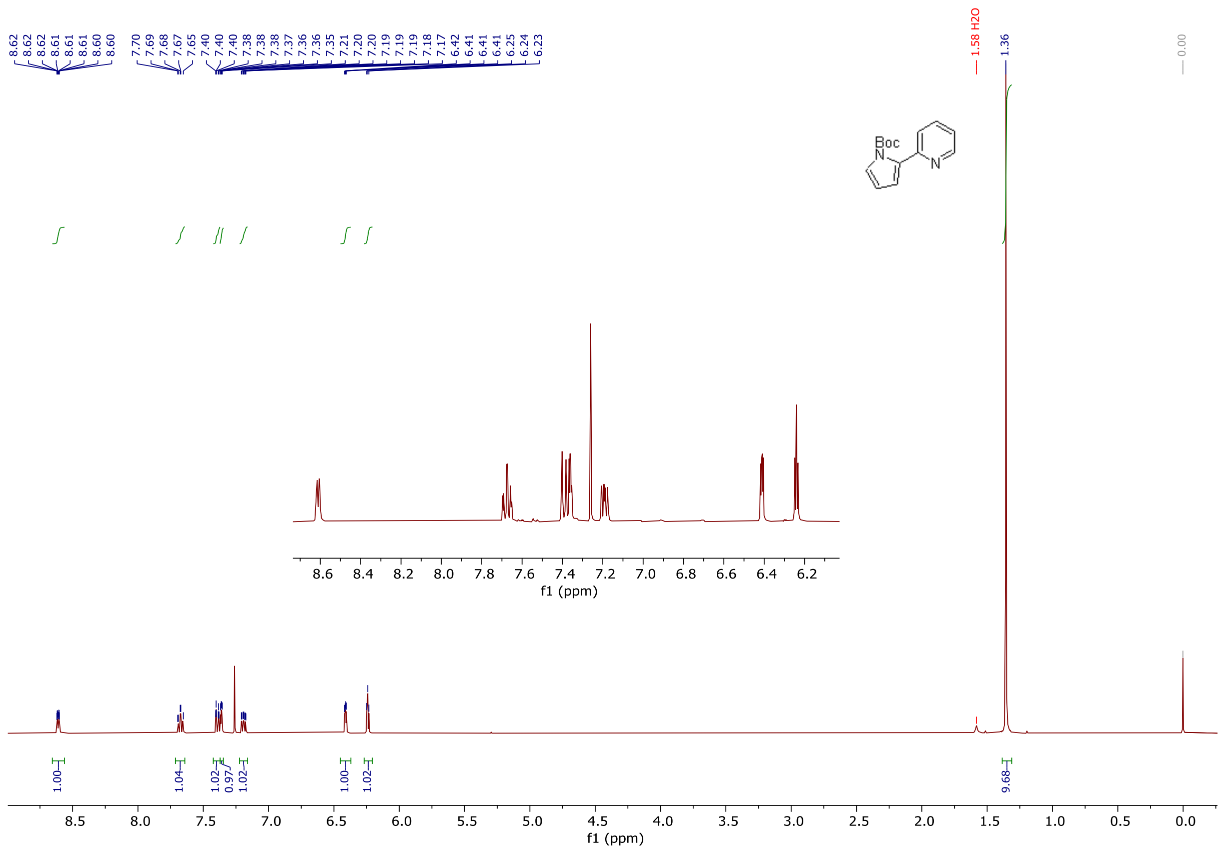

^1H NMR spectrum of pyridine S27a ($CDCl_3$, 298 K)

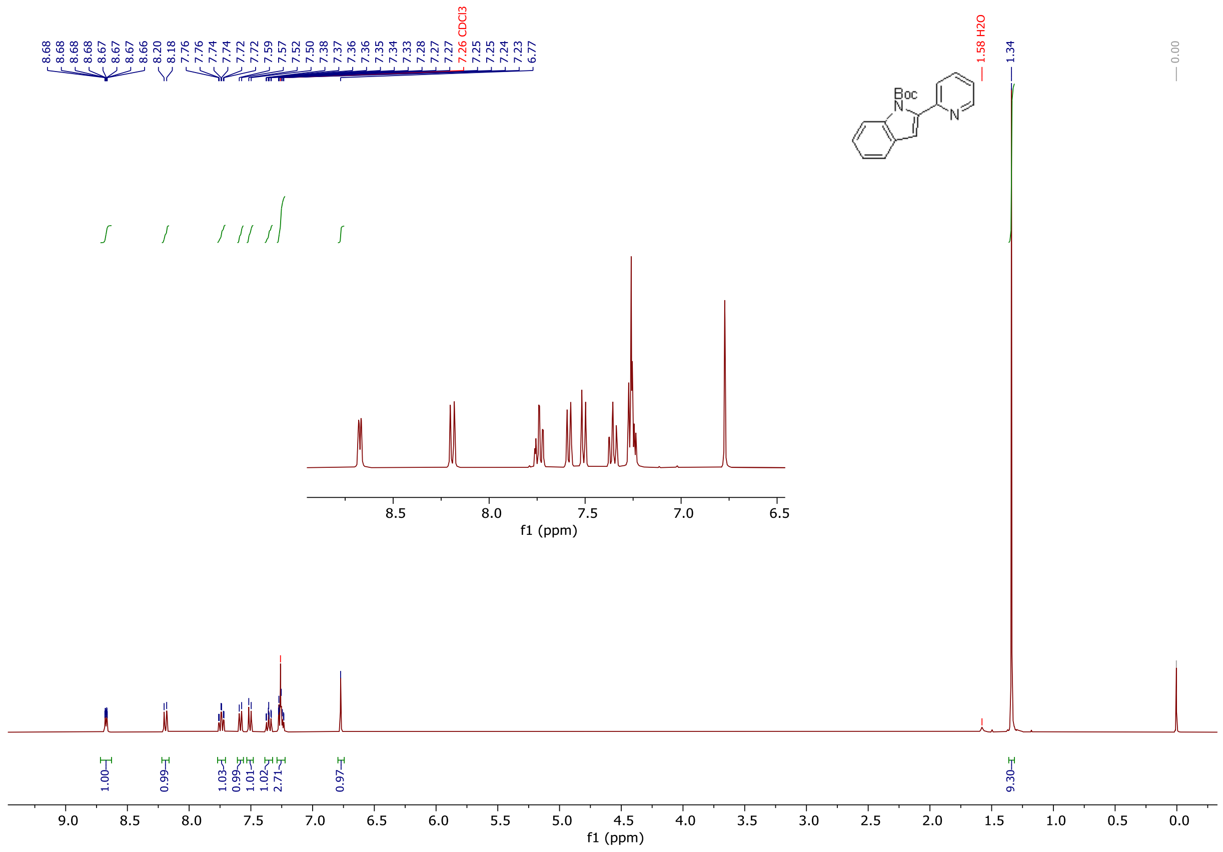

^{1}H NMR spectrum of pyridine S28a ($CDCl_3$, 298 K)

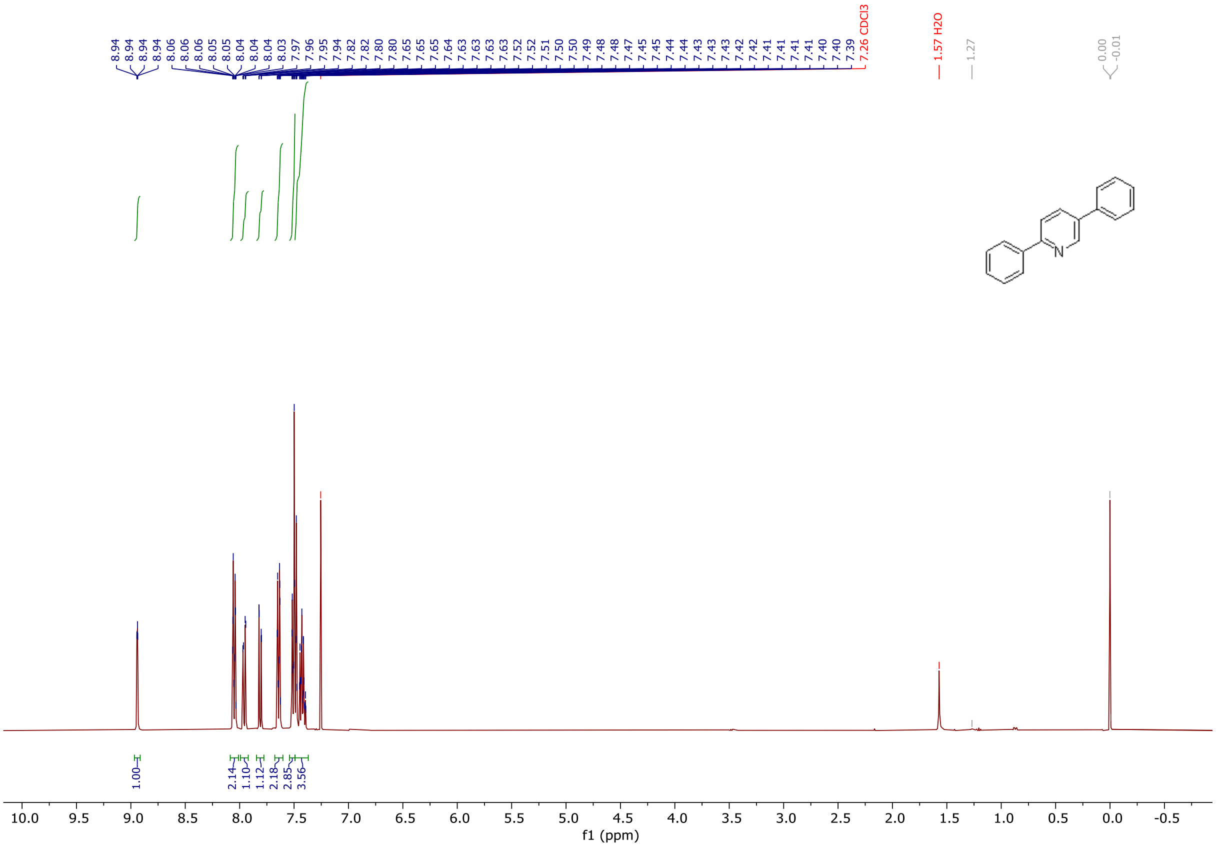

^{1}H NMR spectrum of pyridine S29a ($CDCl_3$, 298 K)

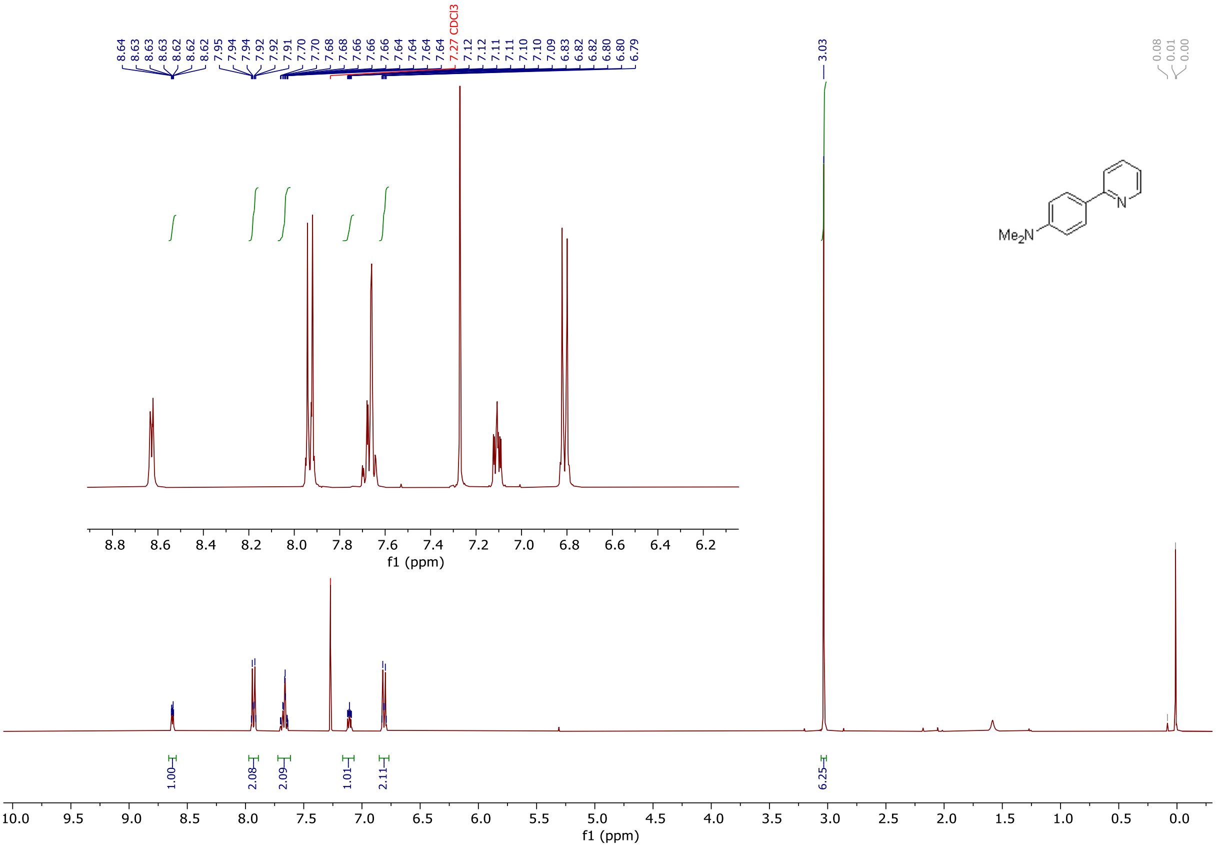

^{1}H NMR spectrum of pyridine S30a ($CDCl_3$, 298 K)

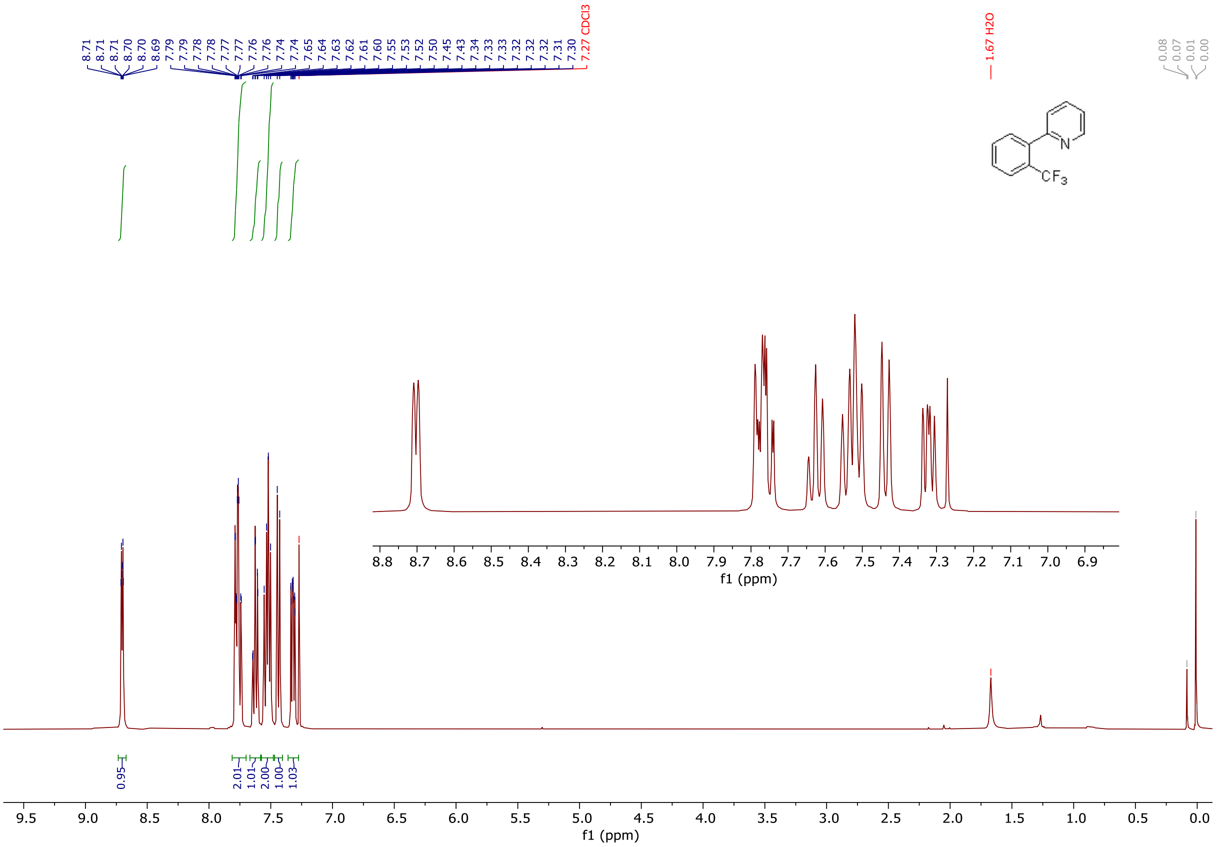

^{1}H NMR spectrum of pyridine S31a ($CDCl_3$, 298 K)

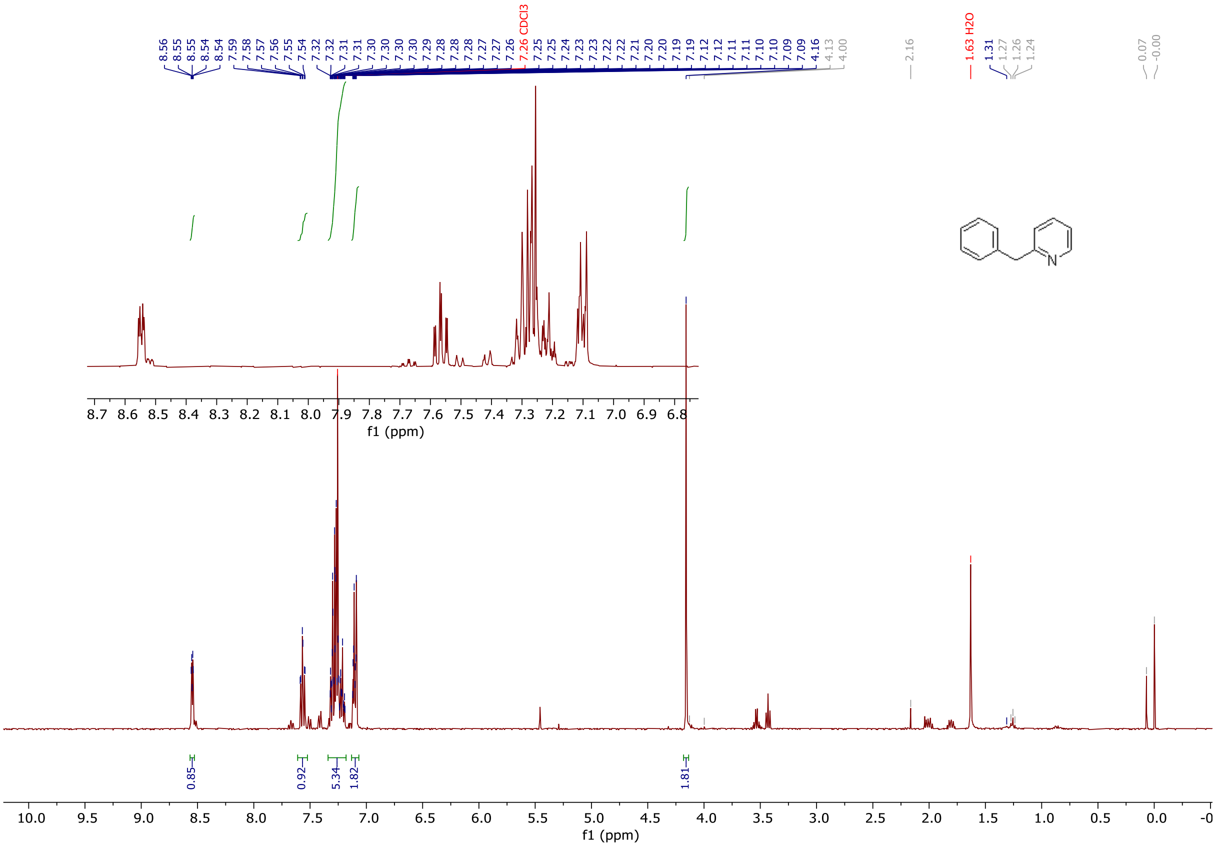

^{1}H NMR spectrum of pyridine S32a ($CDCl_3$, 298 K)

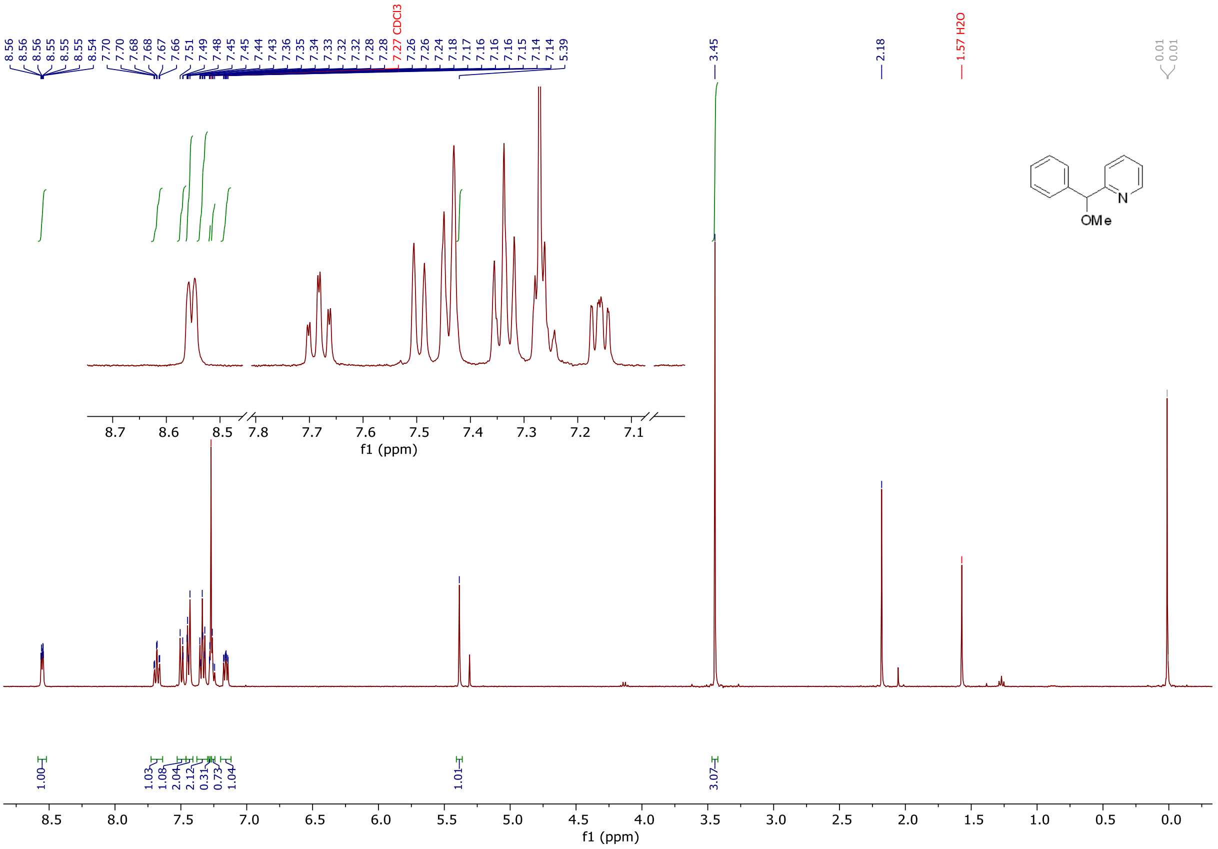

^{1}H NMR spectrum of pyridine S33a ($CDCl_3$, 298 K)

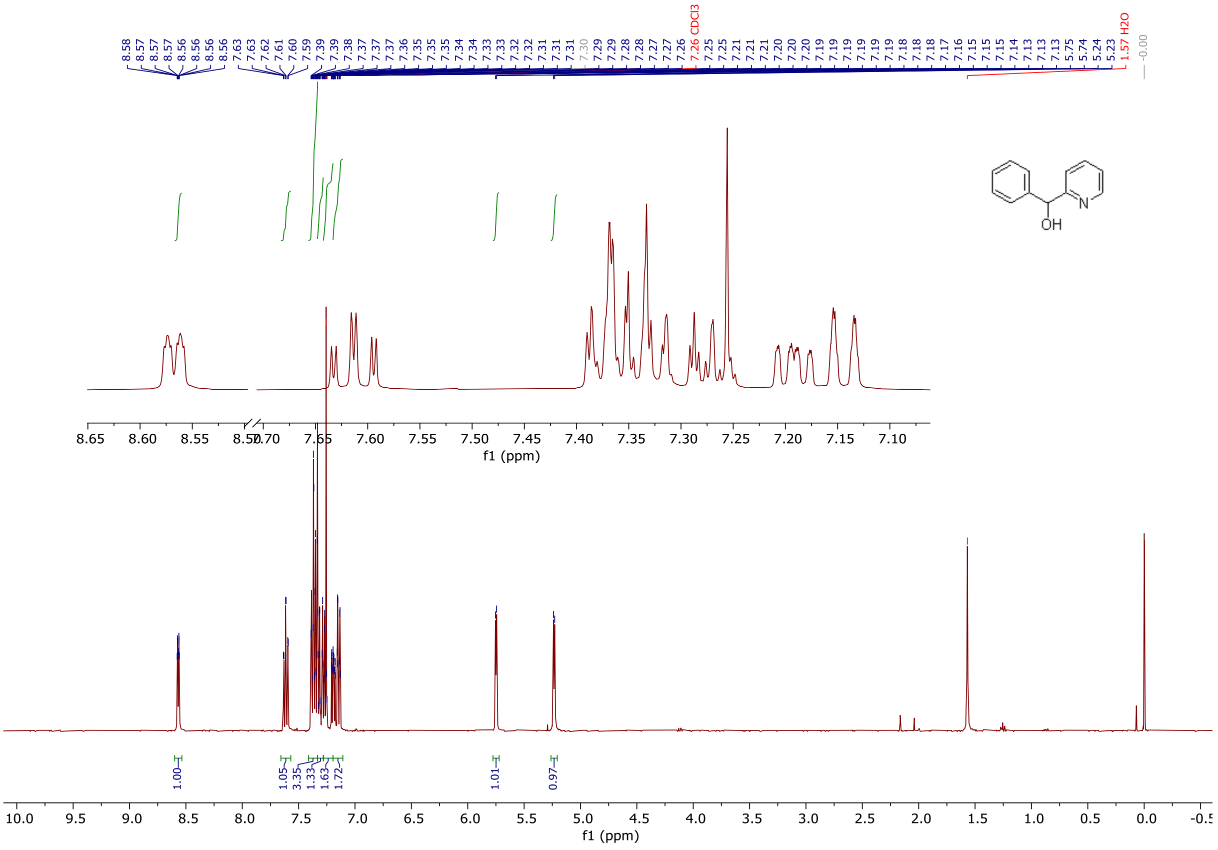

^{1}H NMR spectrum of pyridine S34a ($CDCl_3$, 298 K)

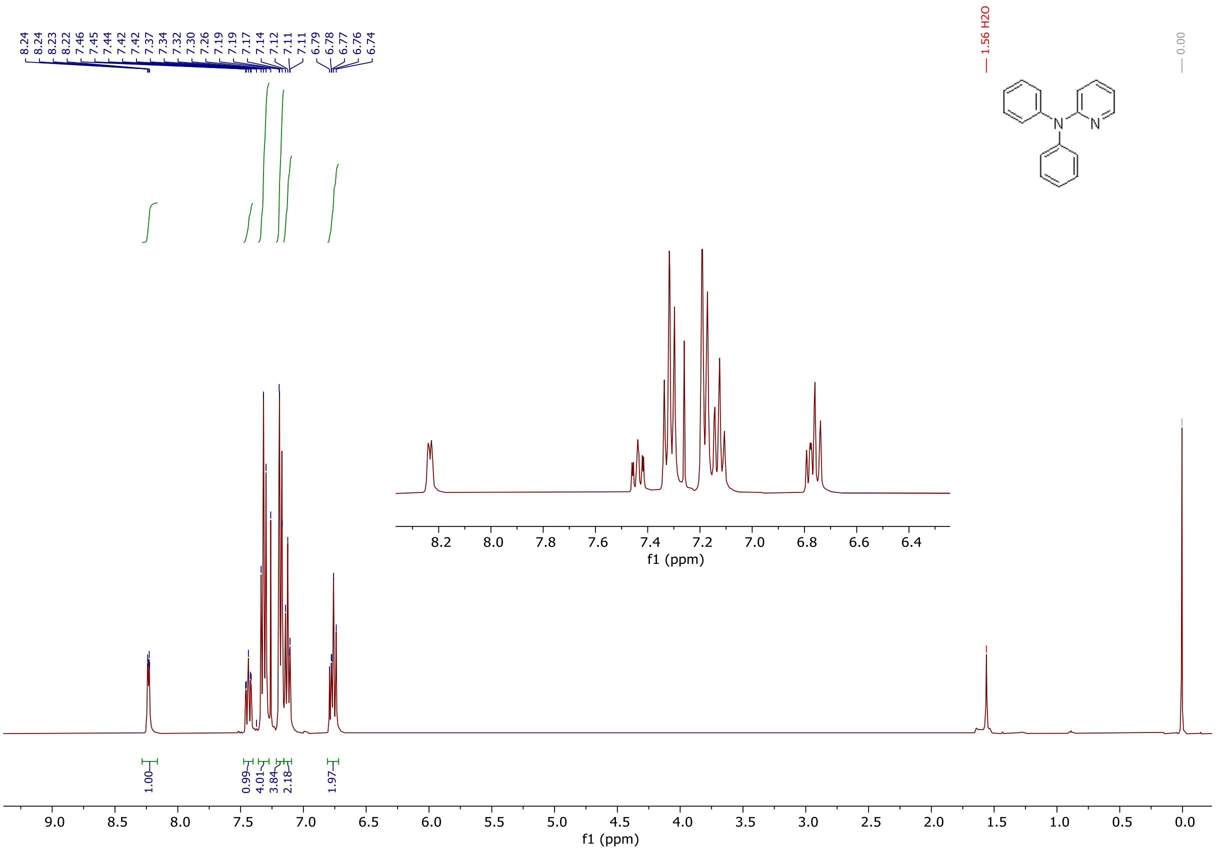

^{1}H NMR spectrum of pyridine S35a ($CDCl_3$, 298 K)

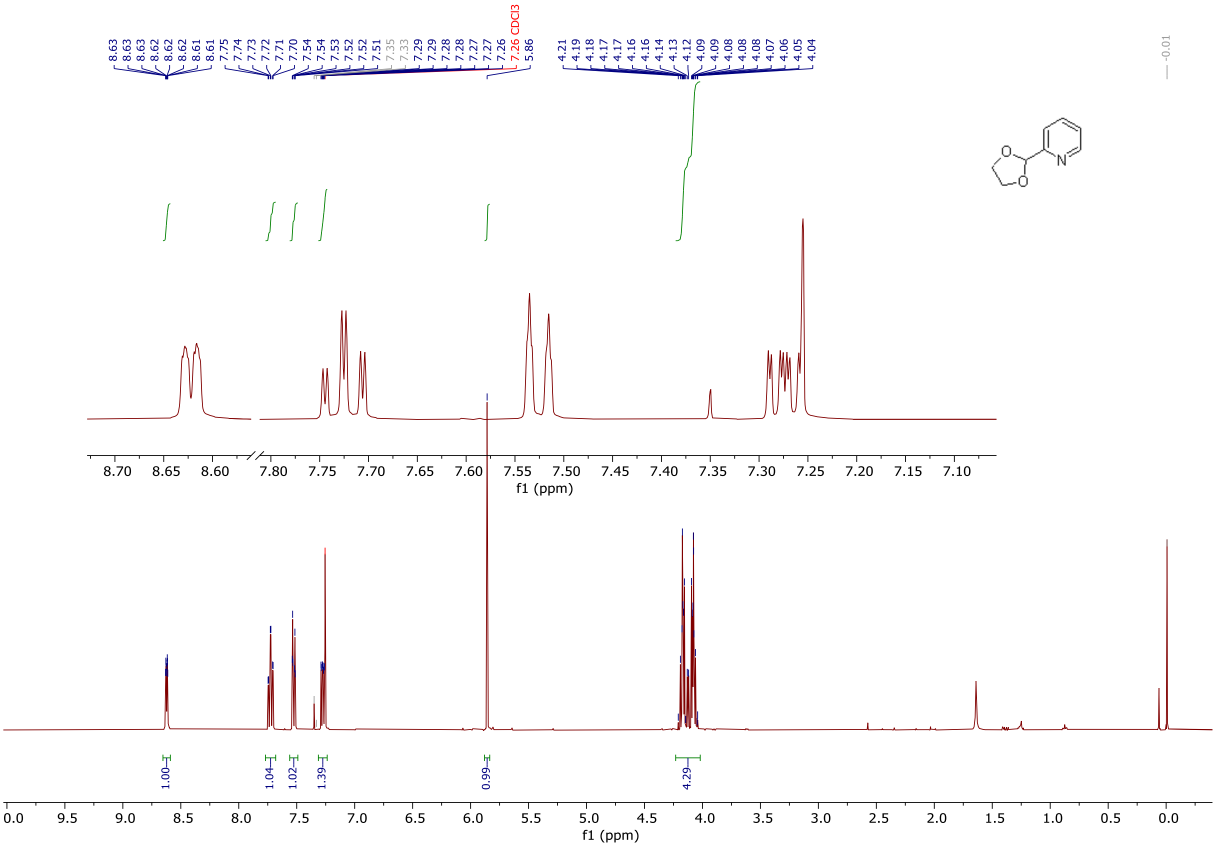

6.2. Zincke imines

^{1}H NMR spectrum of Zincke imine 2 ($CDCl_3$, 298 K)

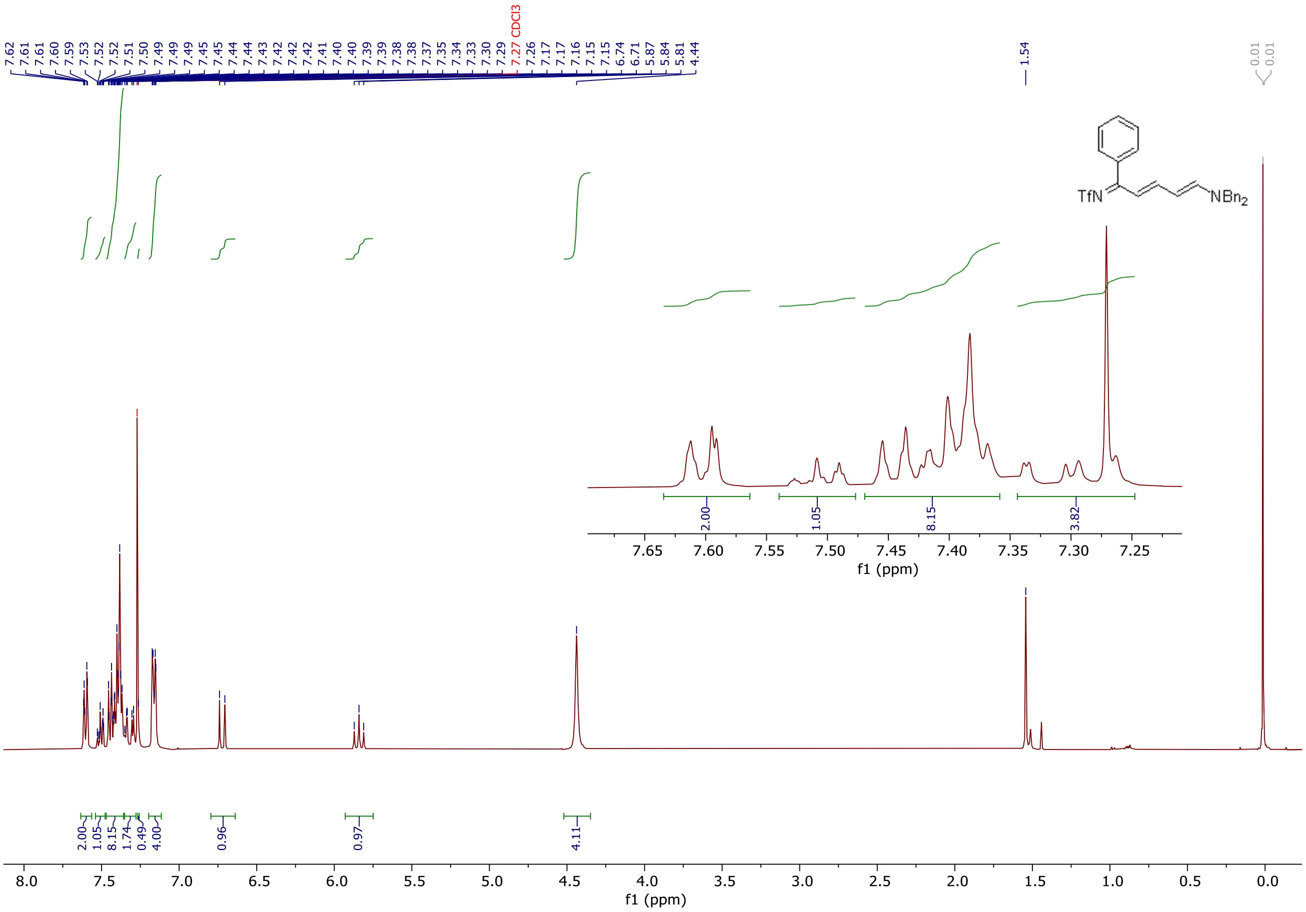

^{1}H NMR spectrum of Zincke imine S2b ($CDCl_3$, 298 K)

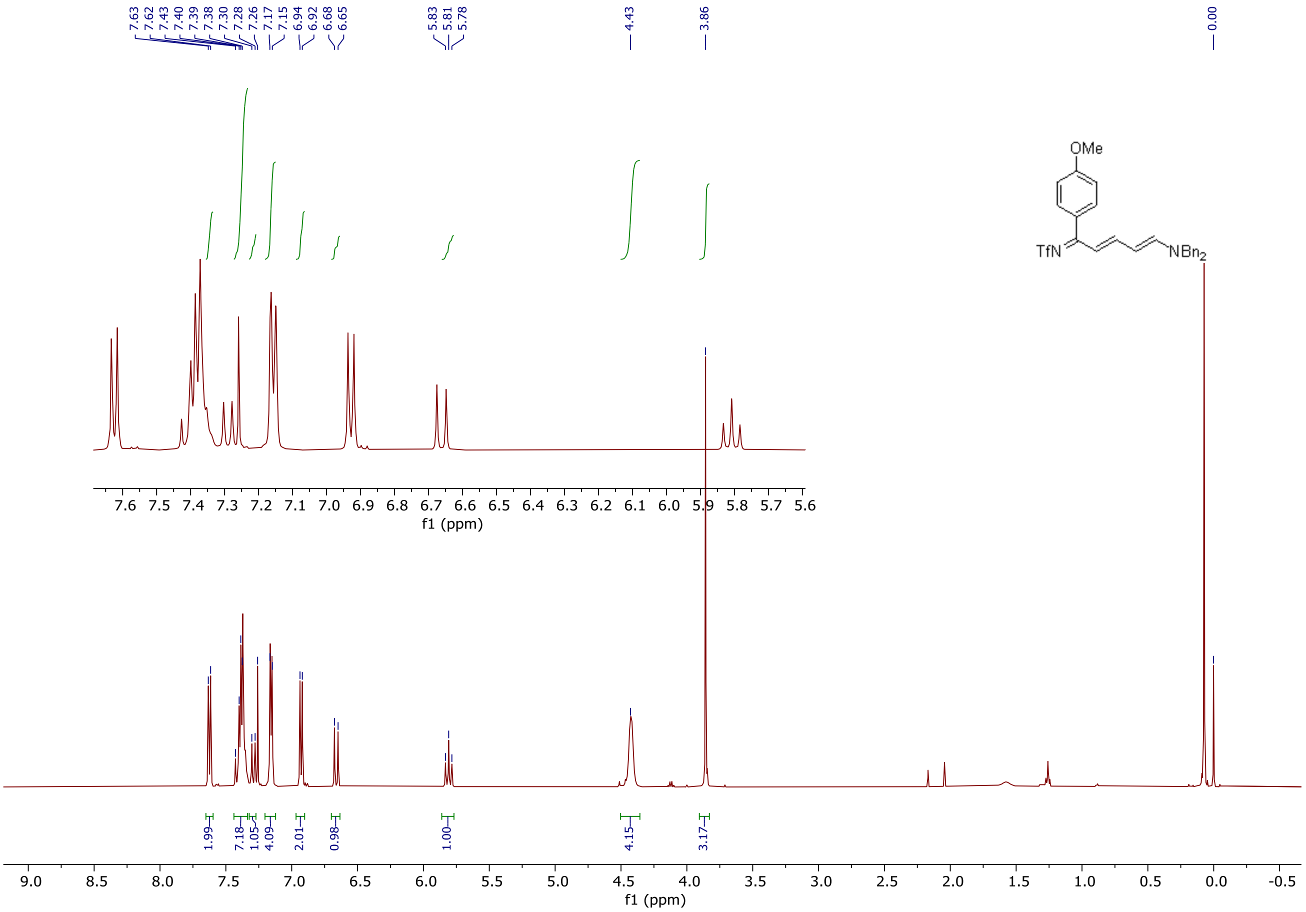

^{13}C NMR spectrum of Zincke imine S2b ($CDCl_3$, 298 K)

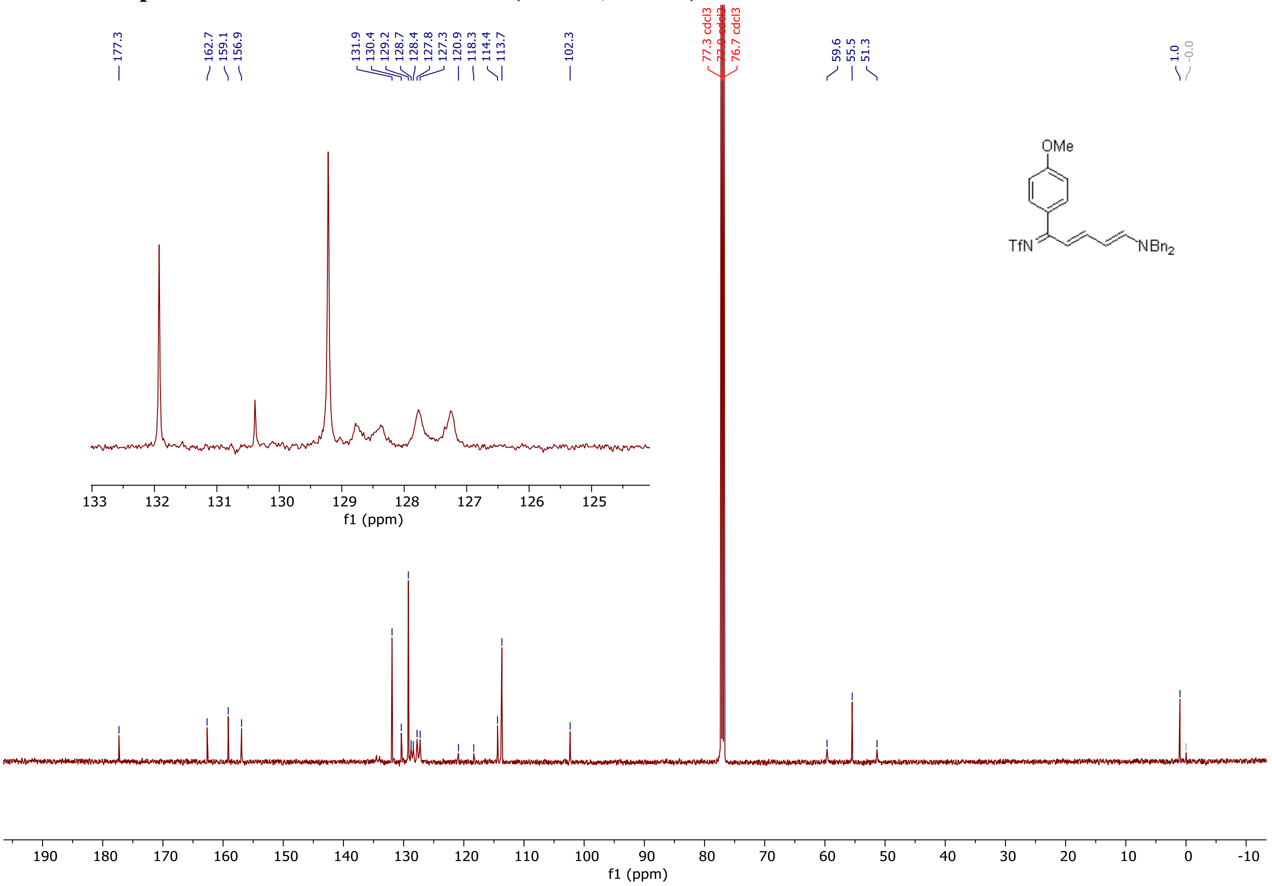

^{1}H NMR spectrum of Zincke imine S3b ($CDCl_3$, 298 K)

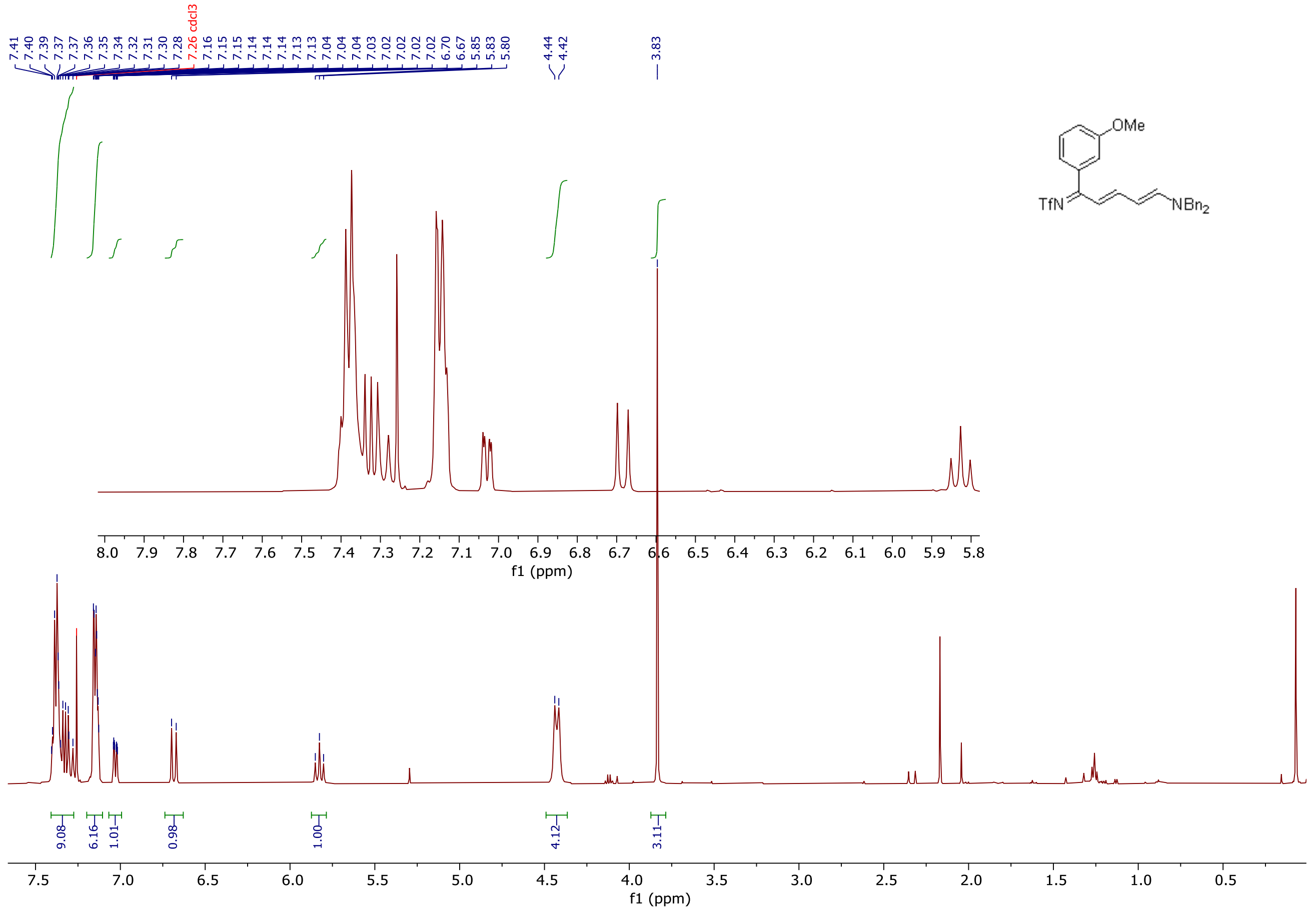

^{13}C NMR spectrum of Zincke imine S3b ($CDCl_3$, 298 K)

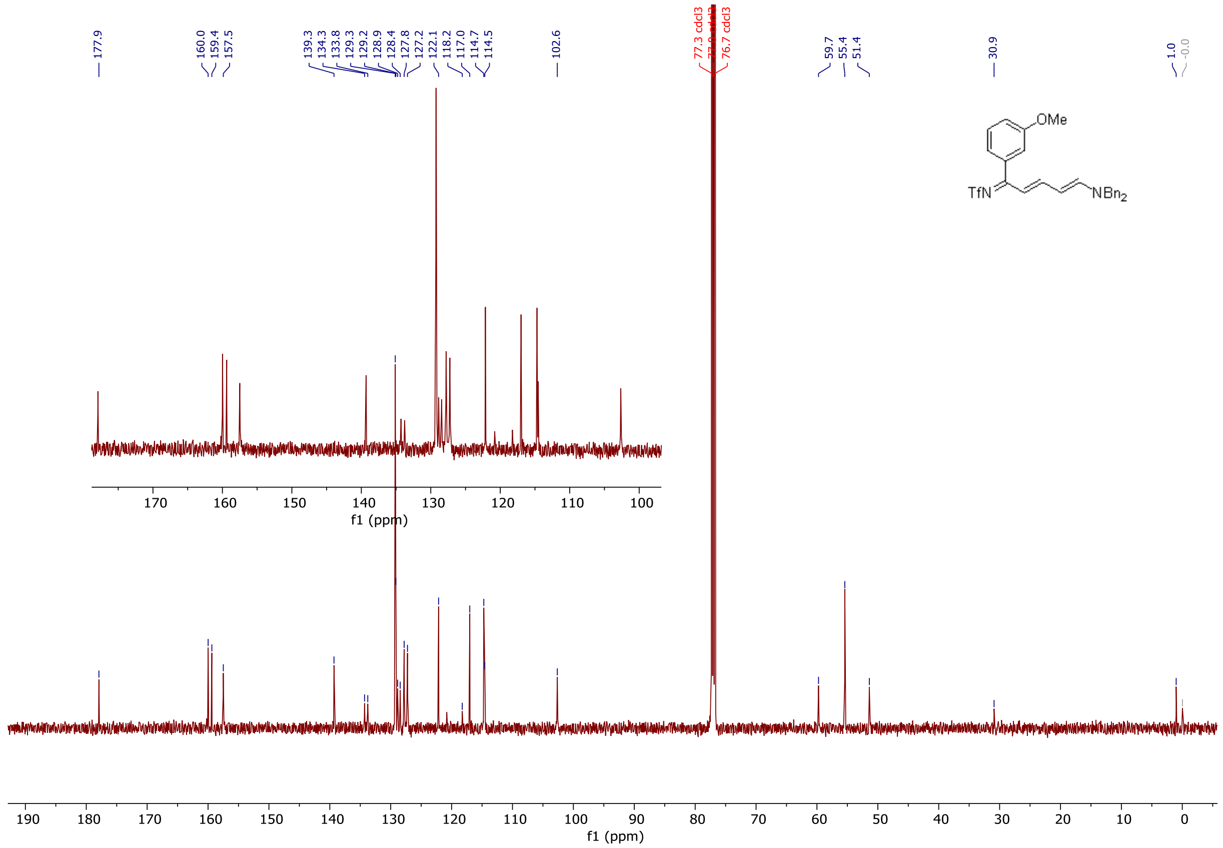

^{1}H NMR spectrum of Zincke imine S4b ($CDCl_3$, 298 K)

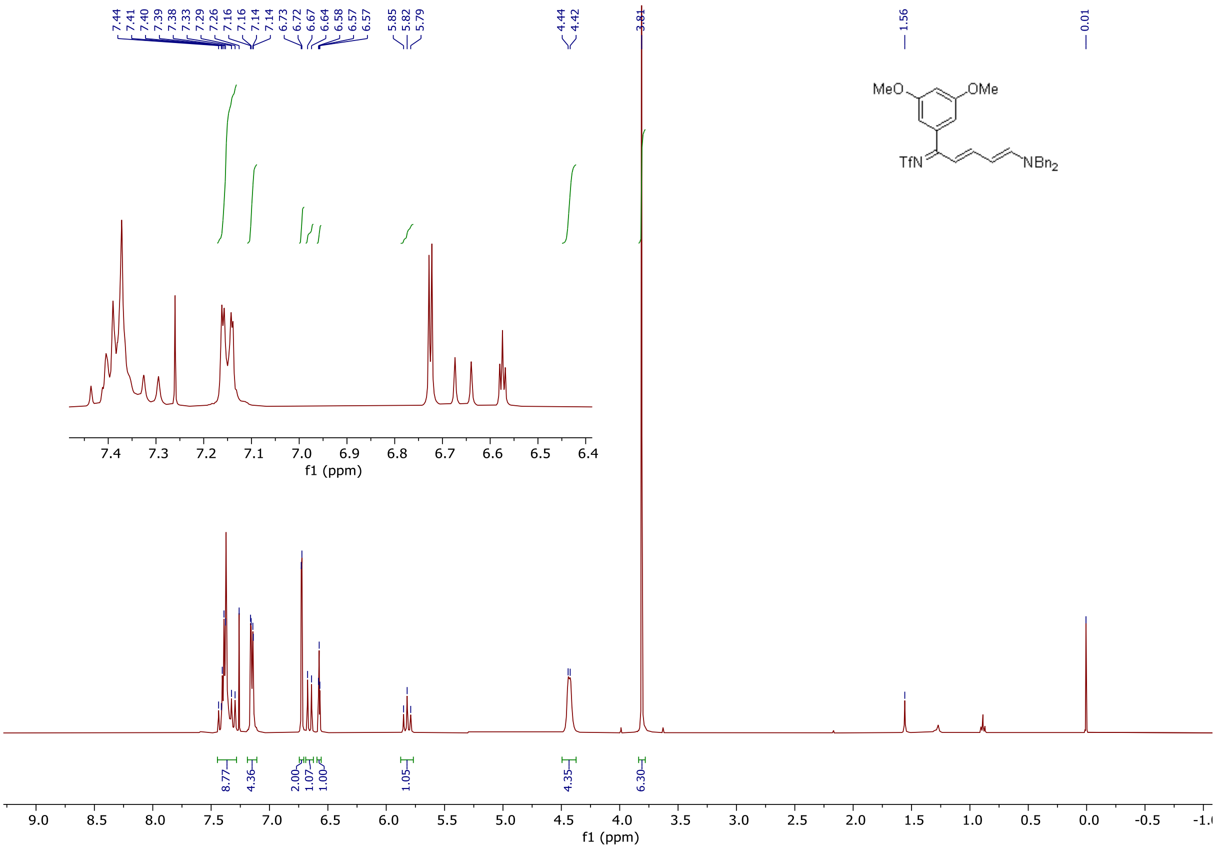

^{13}C NMR spectrum of Zincke imine S4b ($CDCl_3$, 298 K)

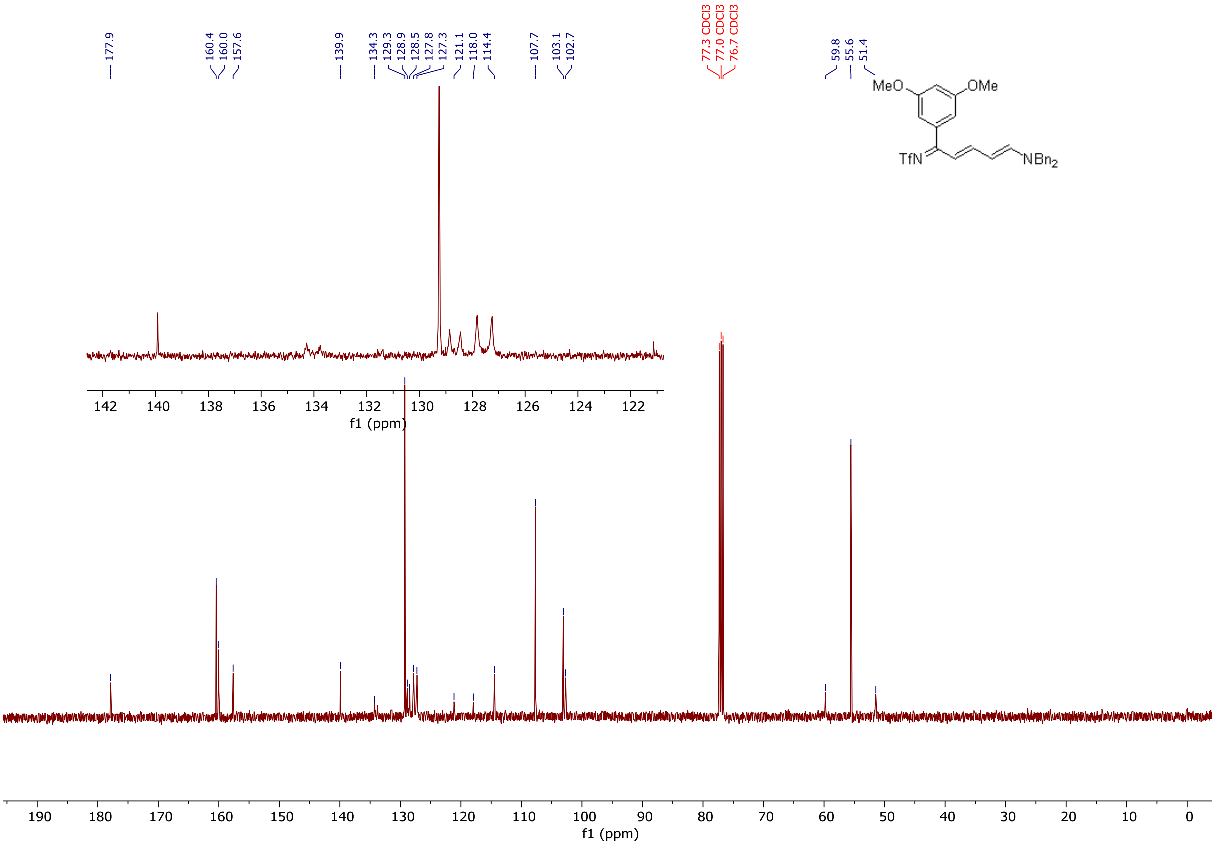

^{1}H NMR spectrum of Zincke imine S5b ($CDCl_3$, 298 K)

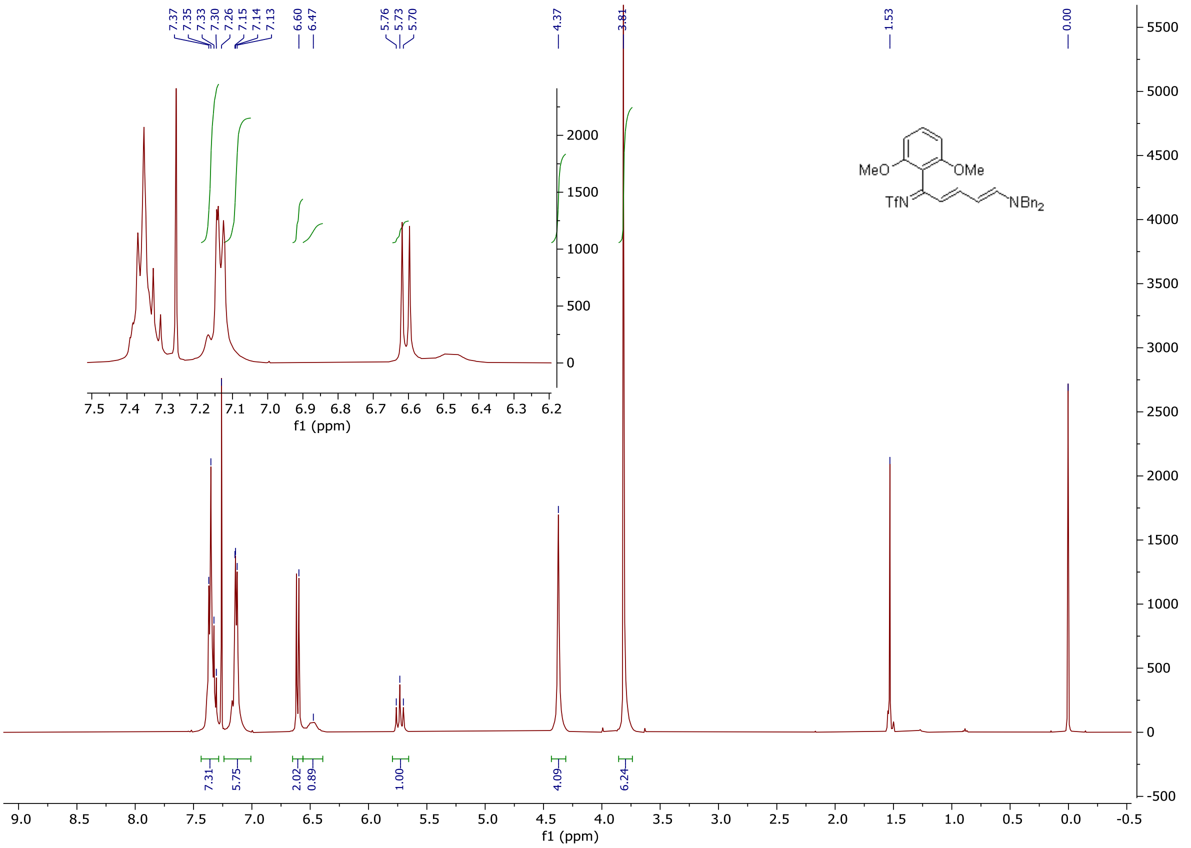

^{13}C NMR spectrum of Zincke imine S5b ($CDCl_3$, 298 K)

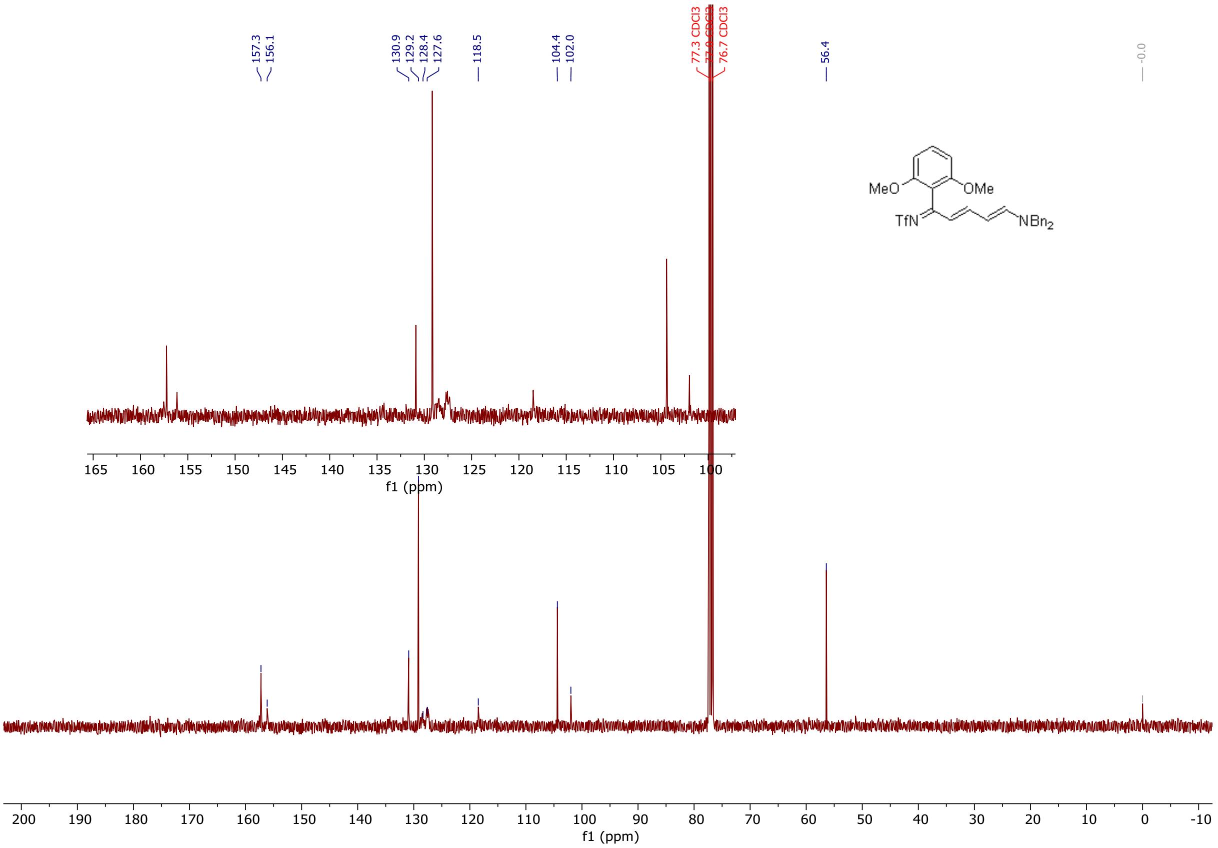

^{1}H NMR spectrum of Zincke imine S6b (CDCl$_3$, 298 K)

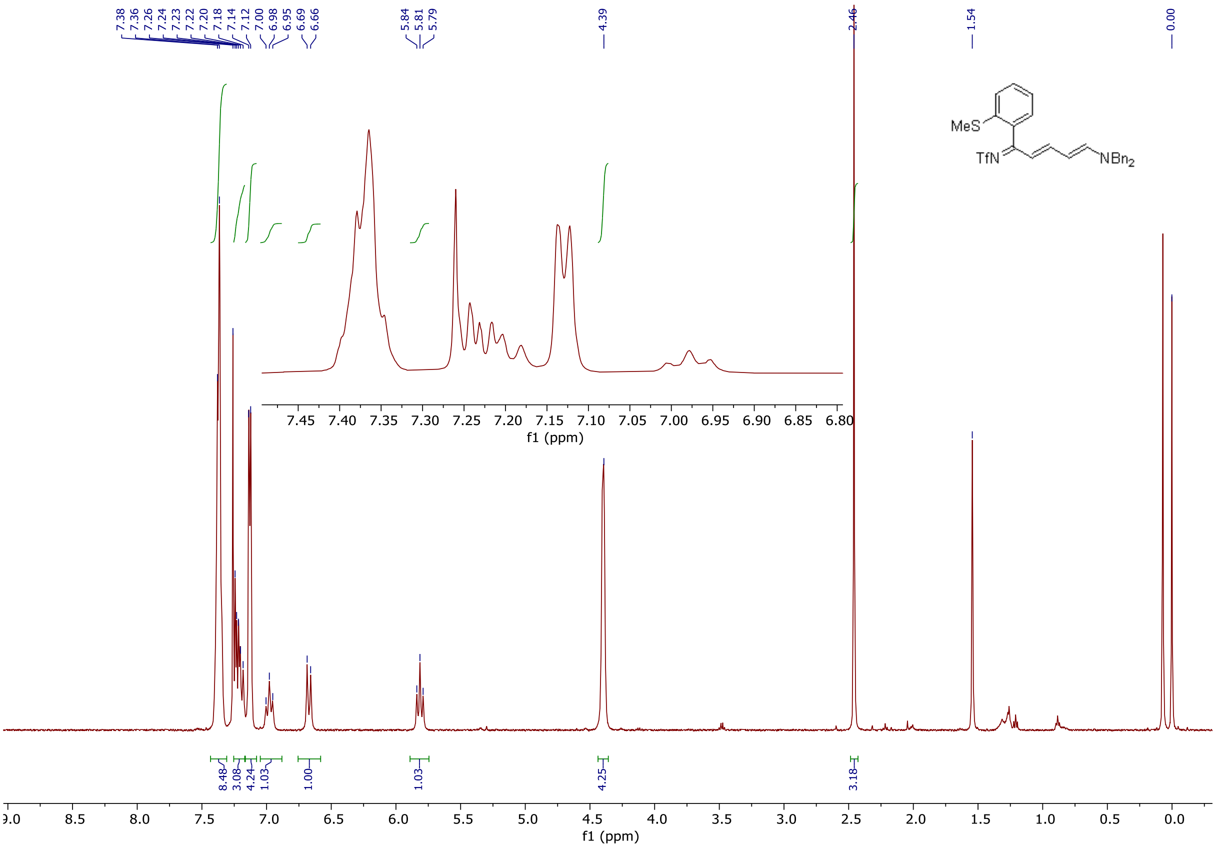

^{13}C NMR spectrum of Zincke imine S6b (CDCl$_3$, 298 K)

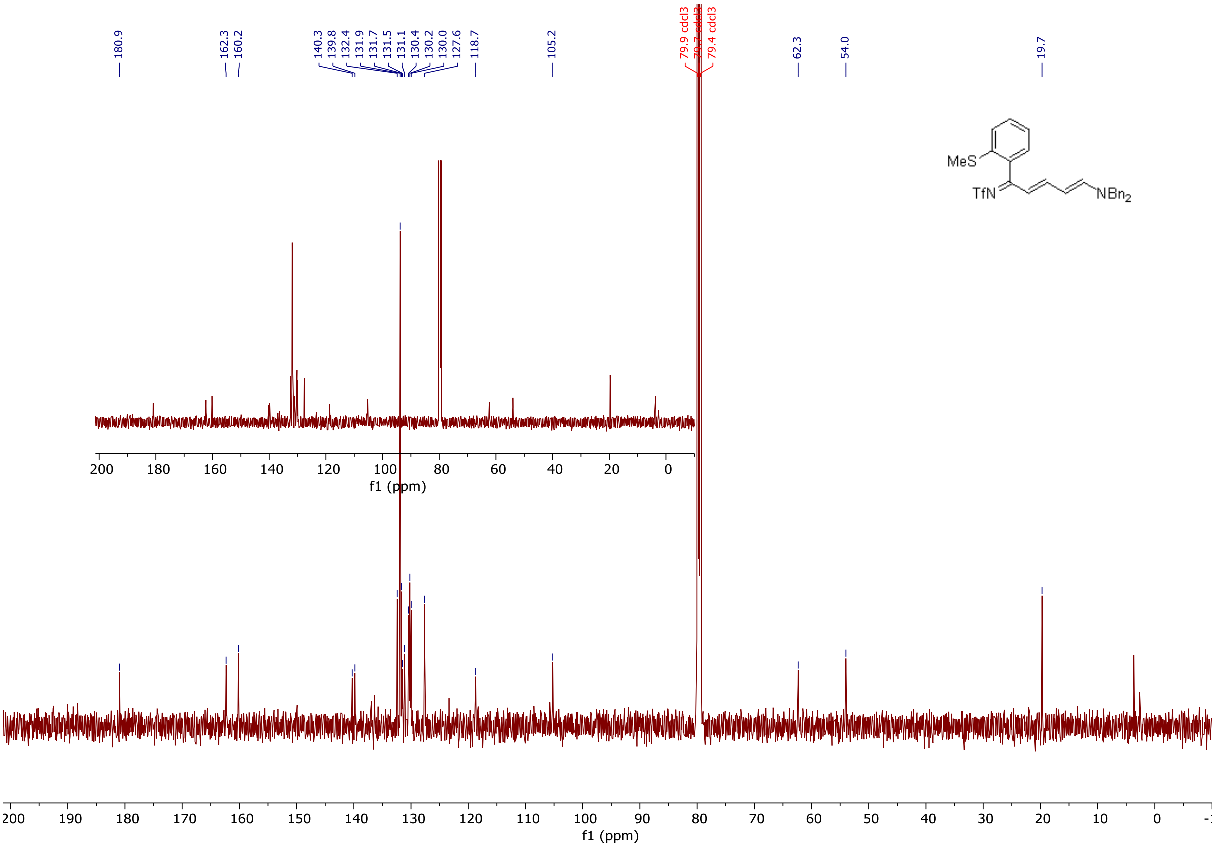

^{1}H NMR spectrum of Zincke imine S7b ($CDCl_3$, 298 K)

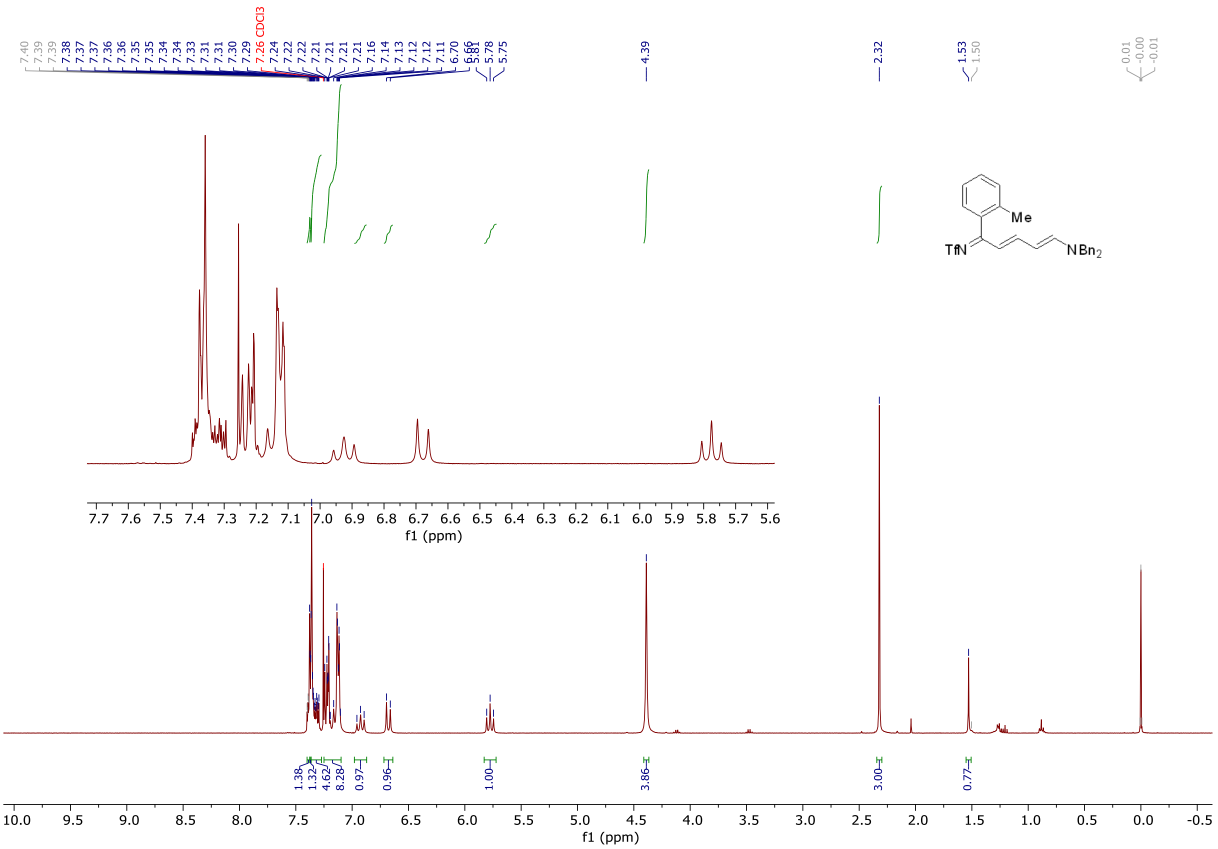

^{13}C NMR spectrum of Zincke imine S7b ($CDCl_3$, 298 K)

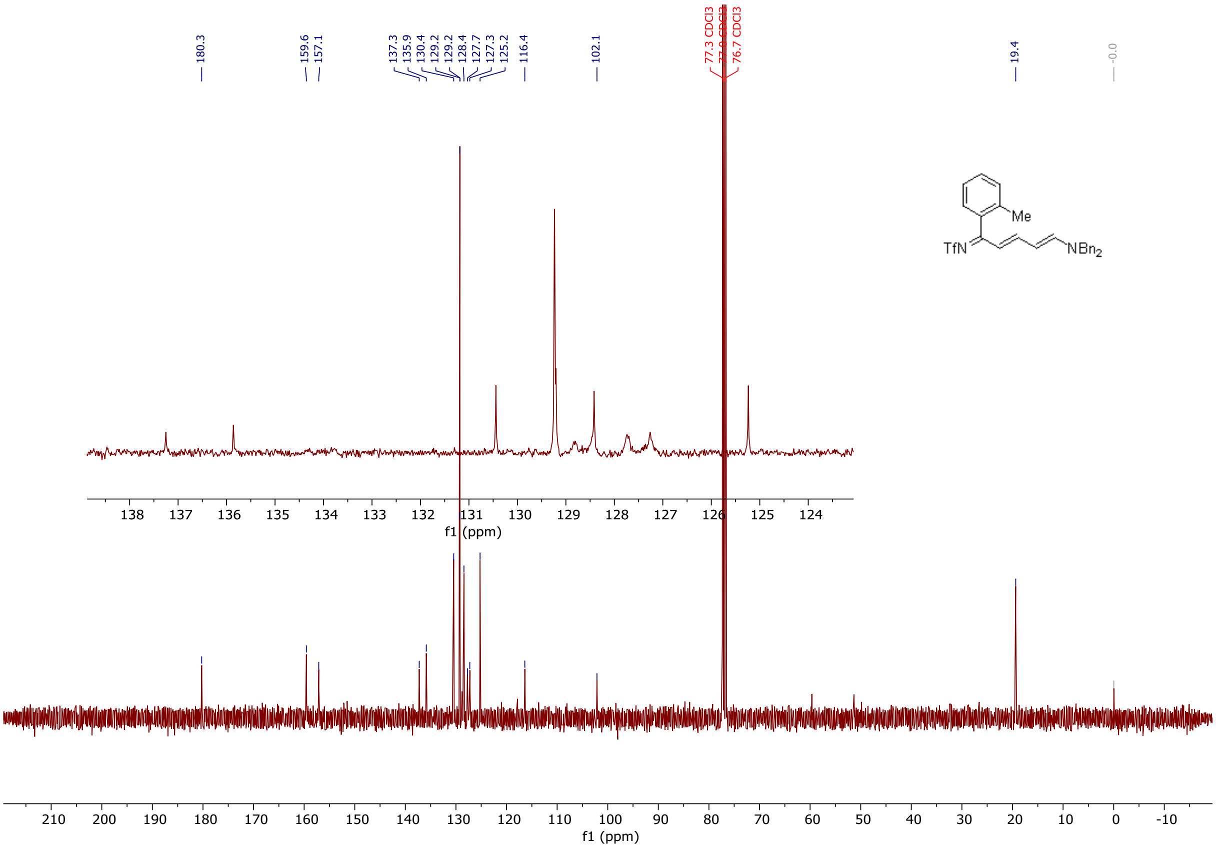

^{1}H NMR spectrum of Zincke imine S8b ($CDCl_3$, 298 K)

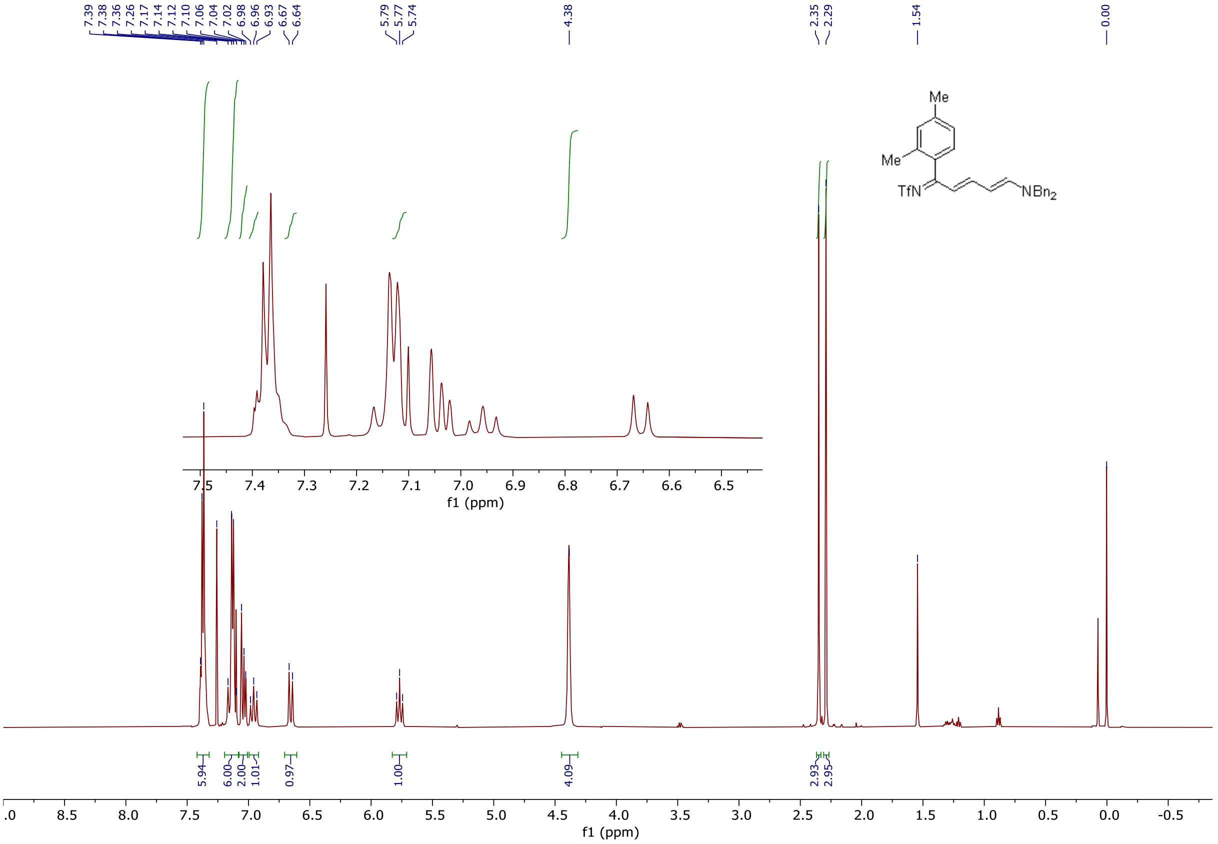

^{13}C NMR spectrum of Zincke imine S8b ($CDCl_3$, 298 K)

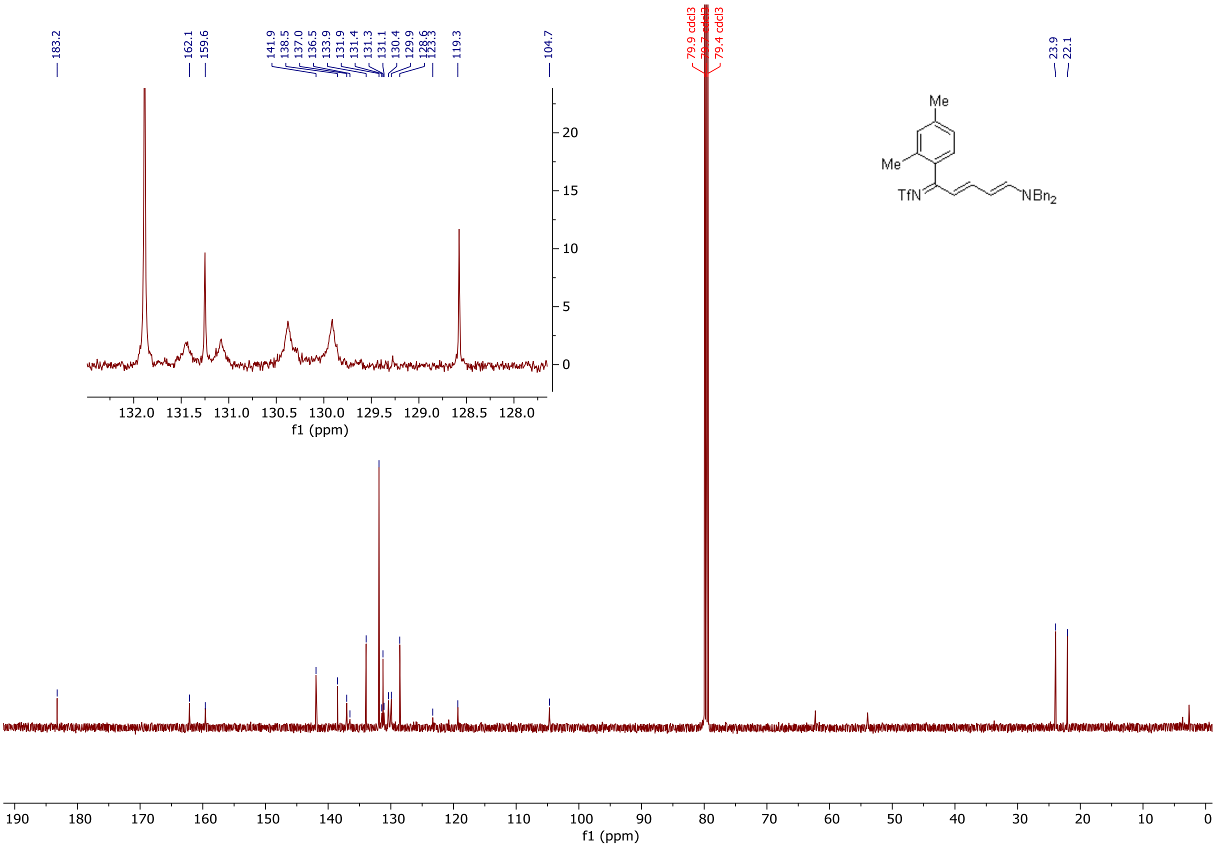

^{1}H NMR spectrum of Zincke imine S9b ($CDCl_3$, 298 K)

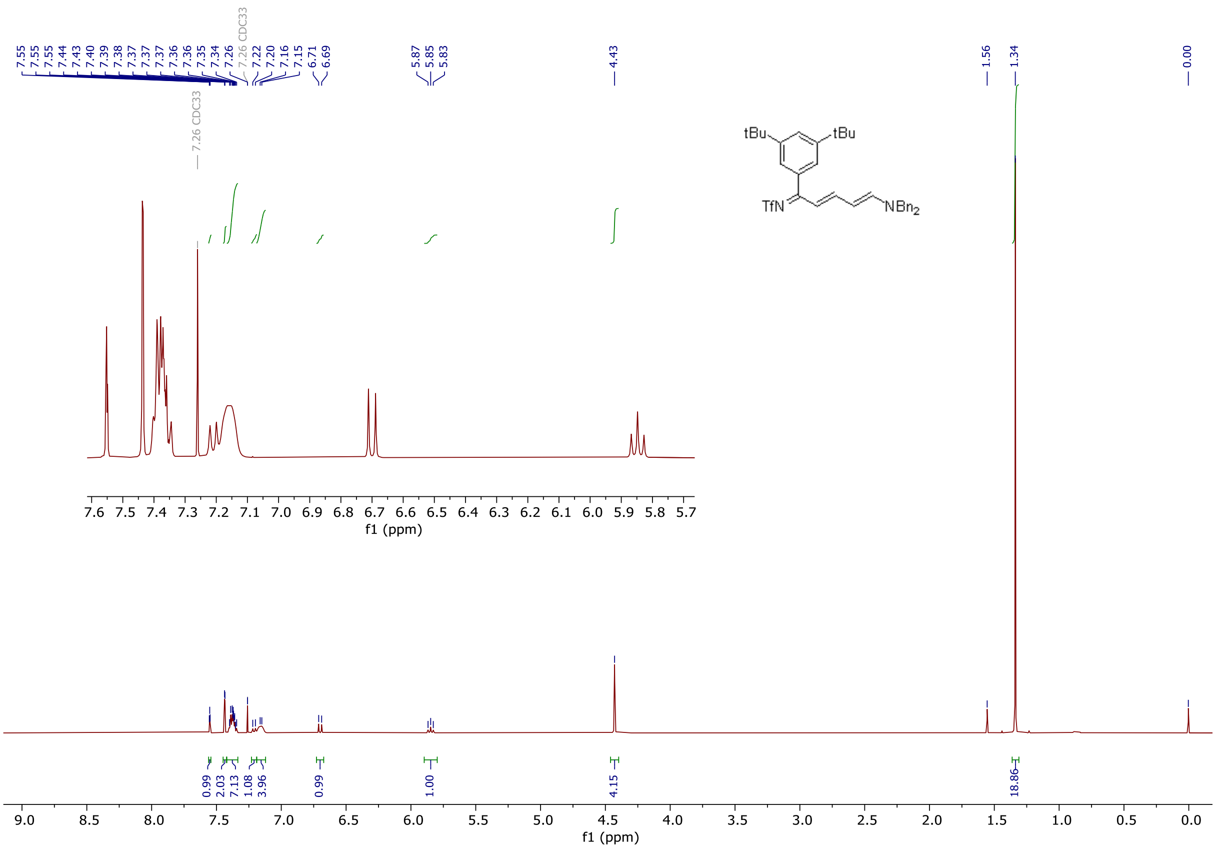

^{13}C NMR spectrum of Zincke imine S9b ($CDCl_3$, 298 K)

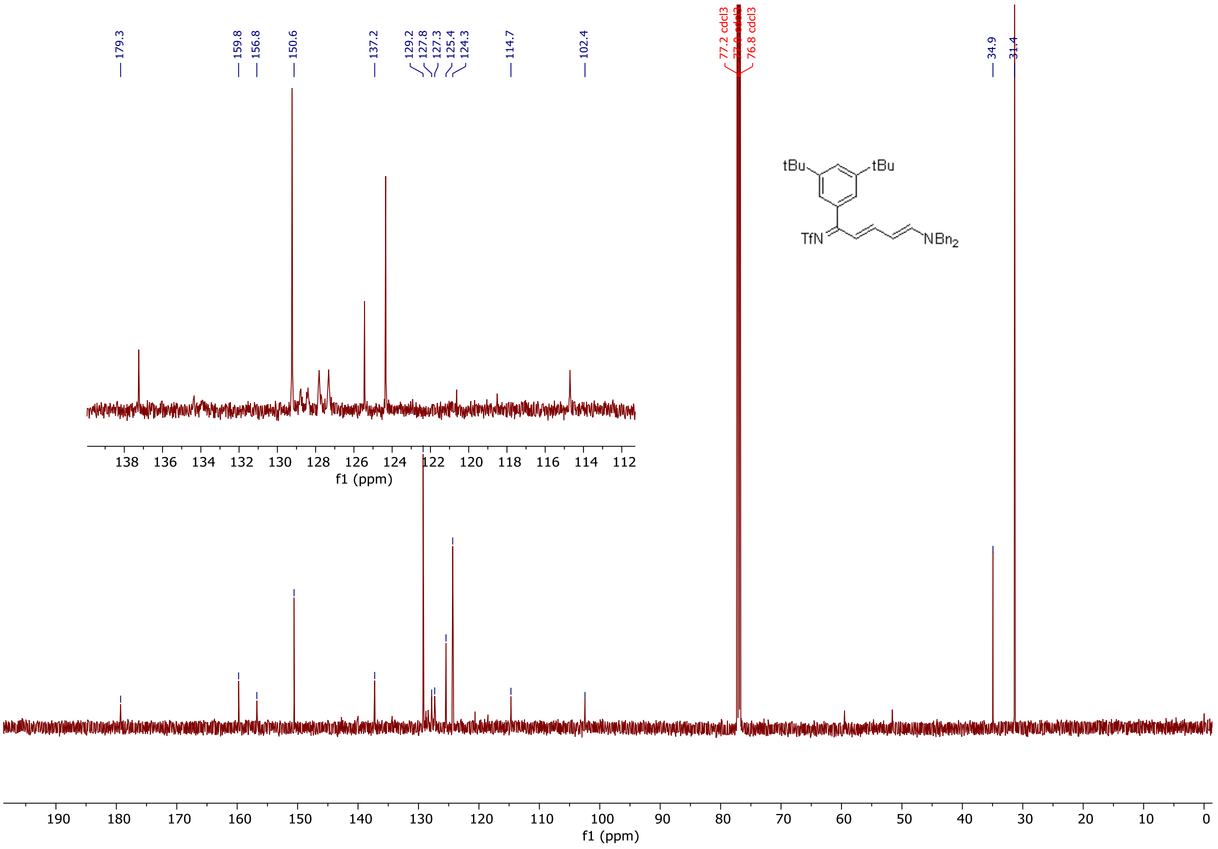

^{1}H NMR spectrum of Zincke imine S10b ($CDCl_3$, 298 K)

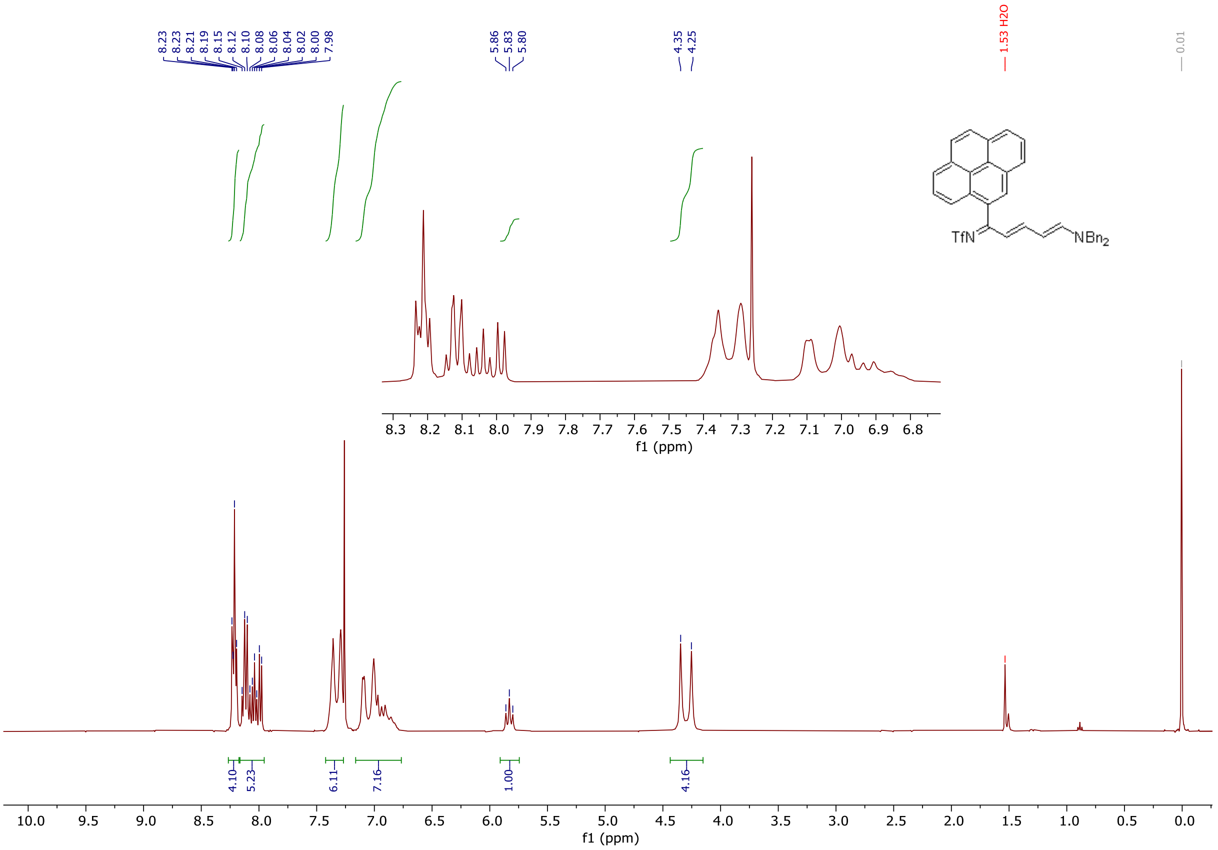

^{13}C NMR spectrum of Zincke imine S10b ($CDCl_3$, 298 K)

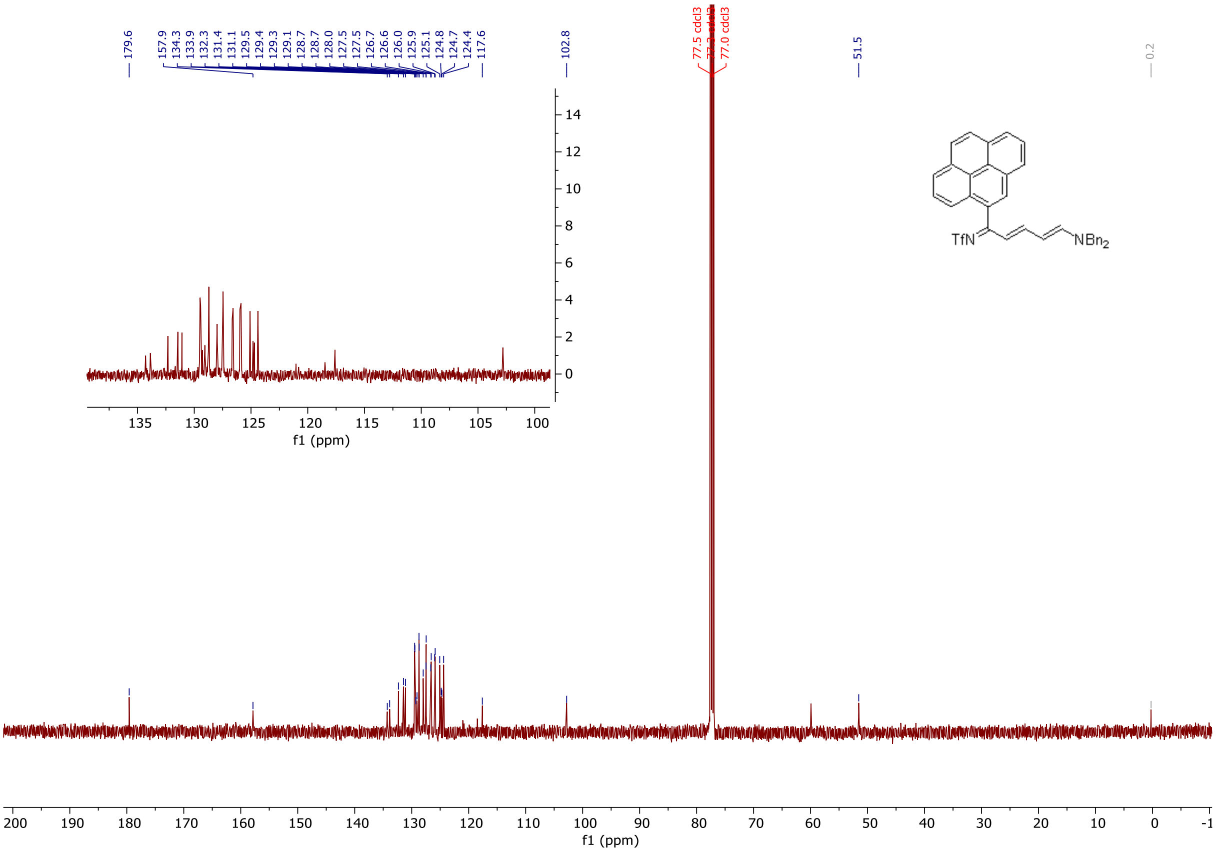

^{1}H NMR spectrum of Zincke imine S11b ($CDCl_3$, 298 K)

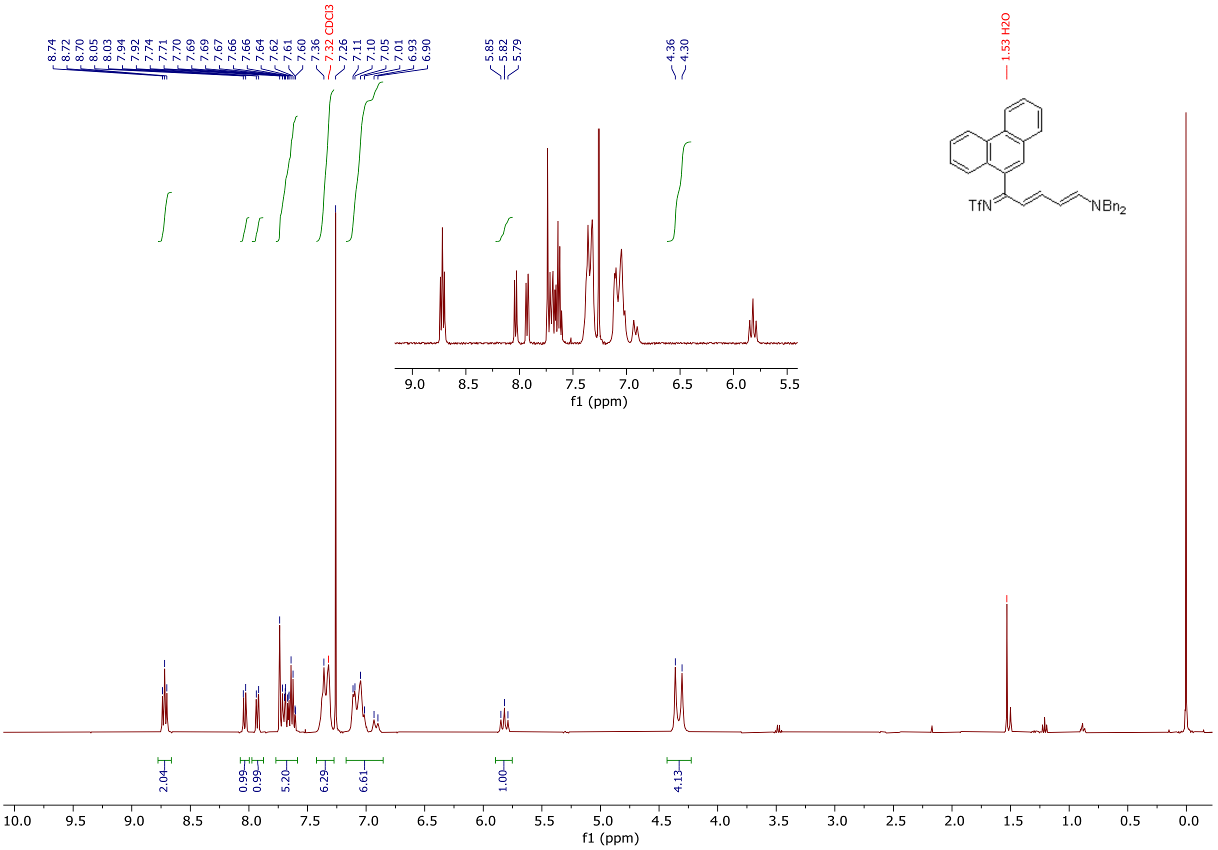

^{13}C NMR spectrum of Zincke imine S11b ($CDCl_3$, 298 K)

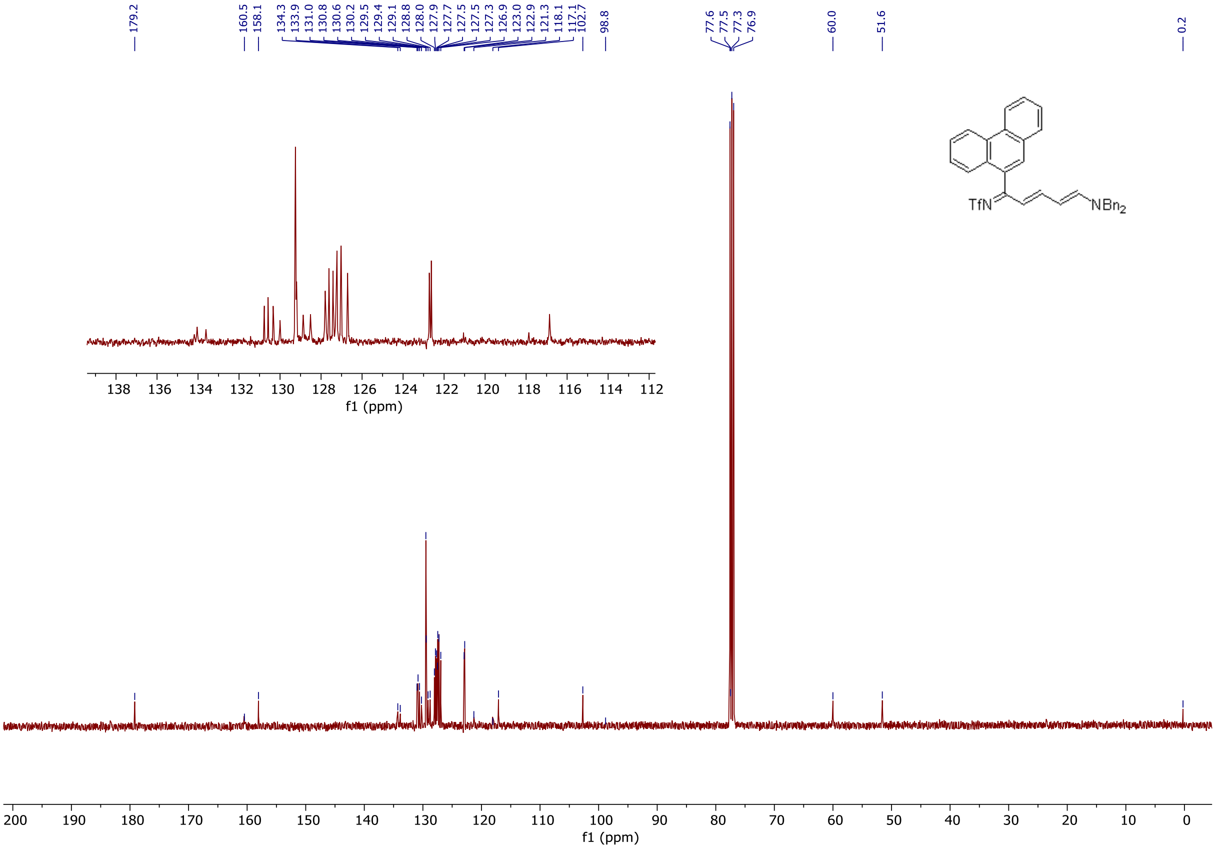

^{1}H NMR spectrum of Zincke imie S12b ($CDCl_3$, 298 K)

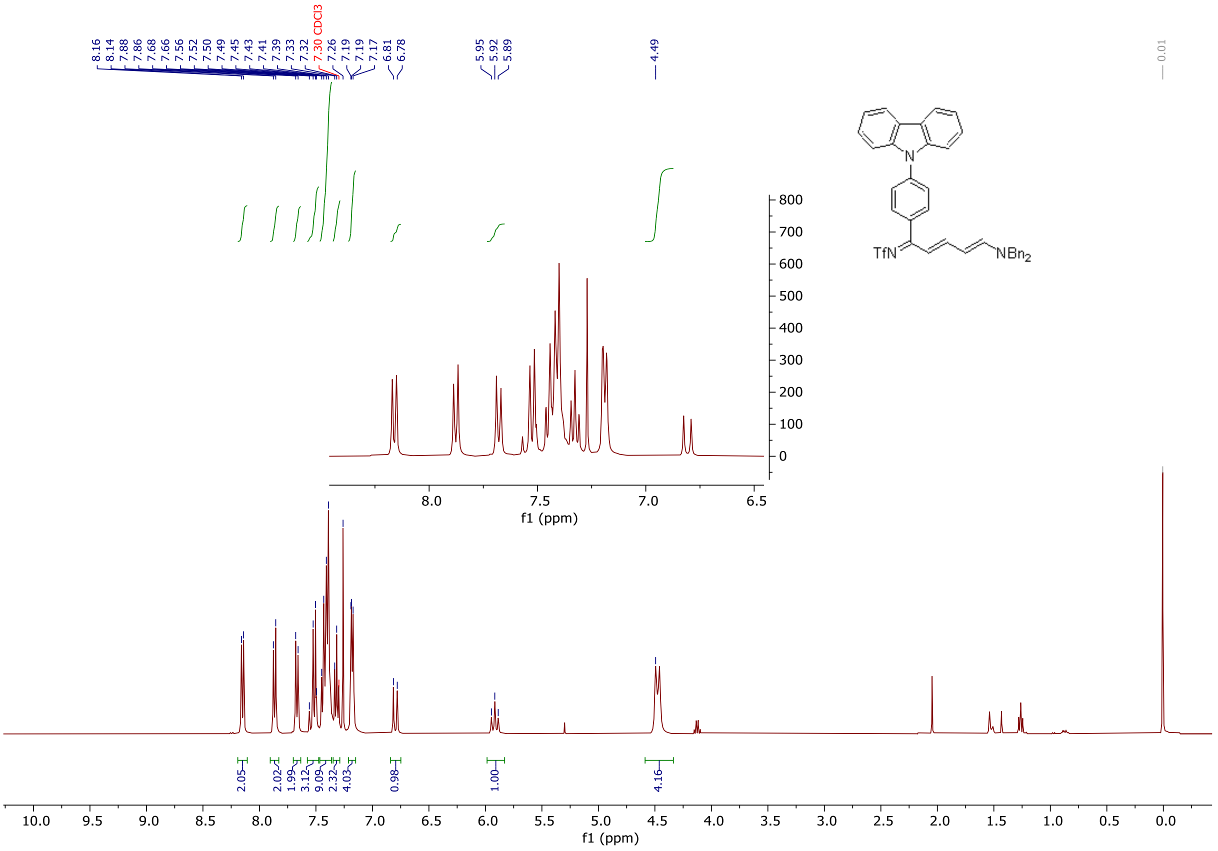

^{13}C NMR spectrum of Zincke imine S12b ($CDCl_3$, 298 K)

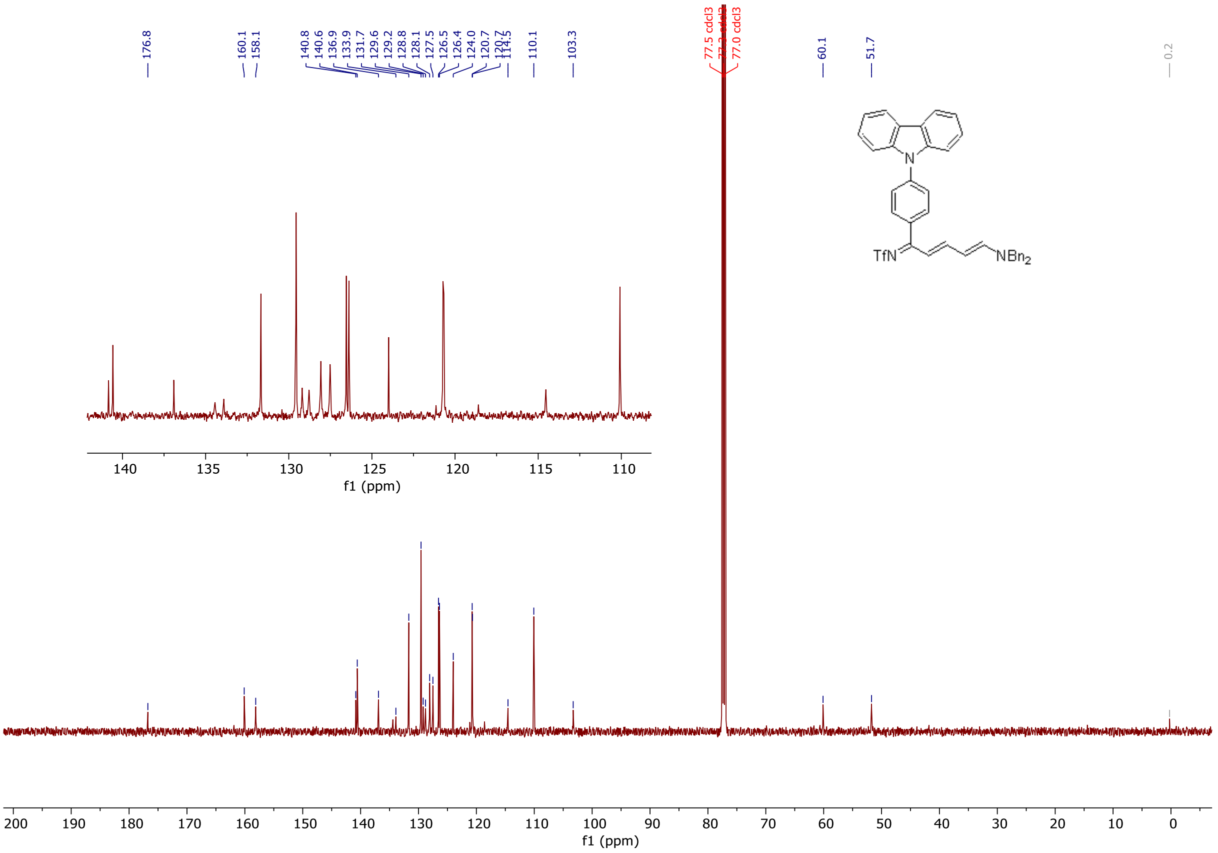

^{1}H NMR spectrum of Zincke imine S13b ($CDCl_3$, 298 K)

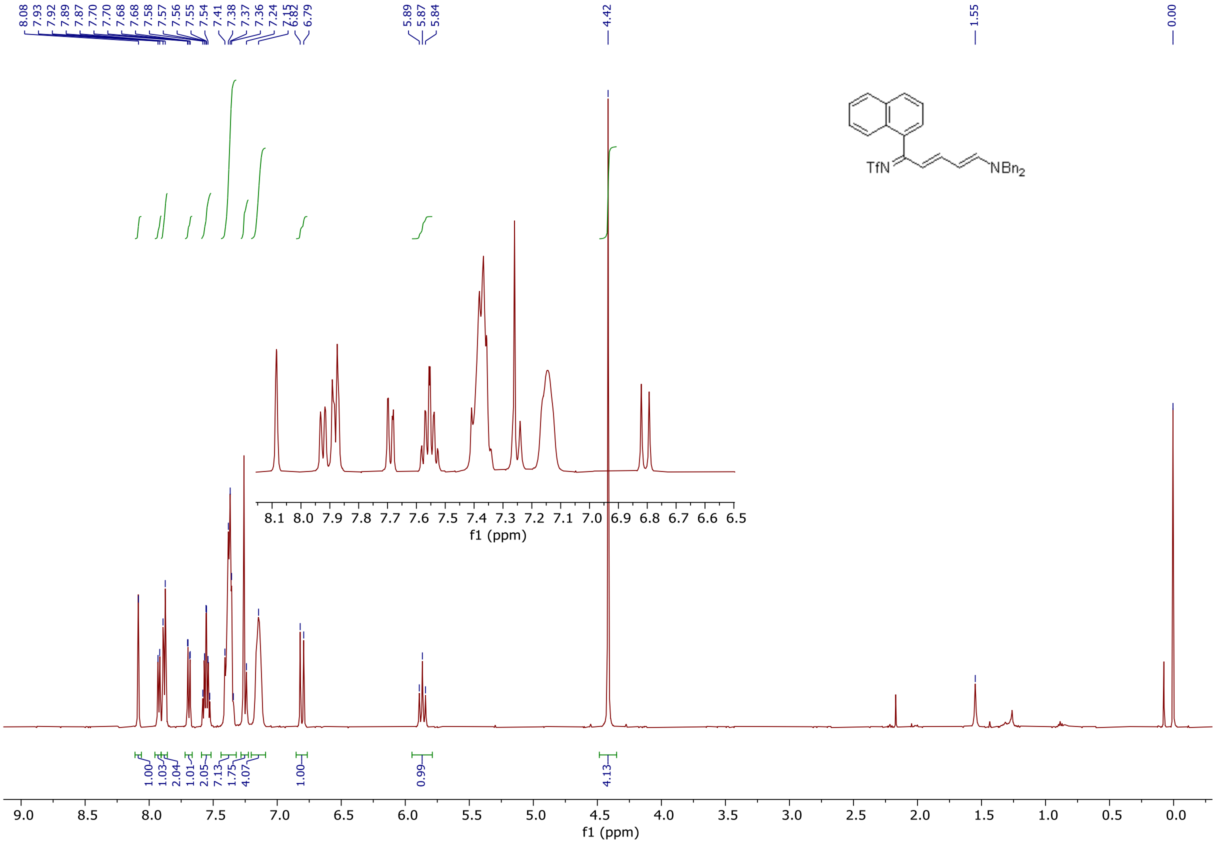

^{13}C NMR spectrum of Zincke imine S13b ($CDCl_3$, 298 K)

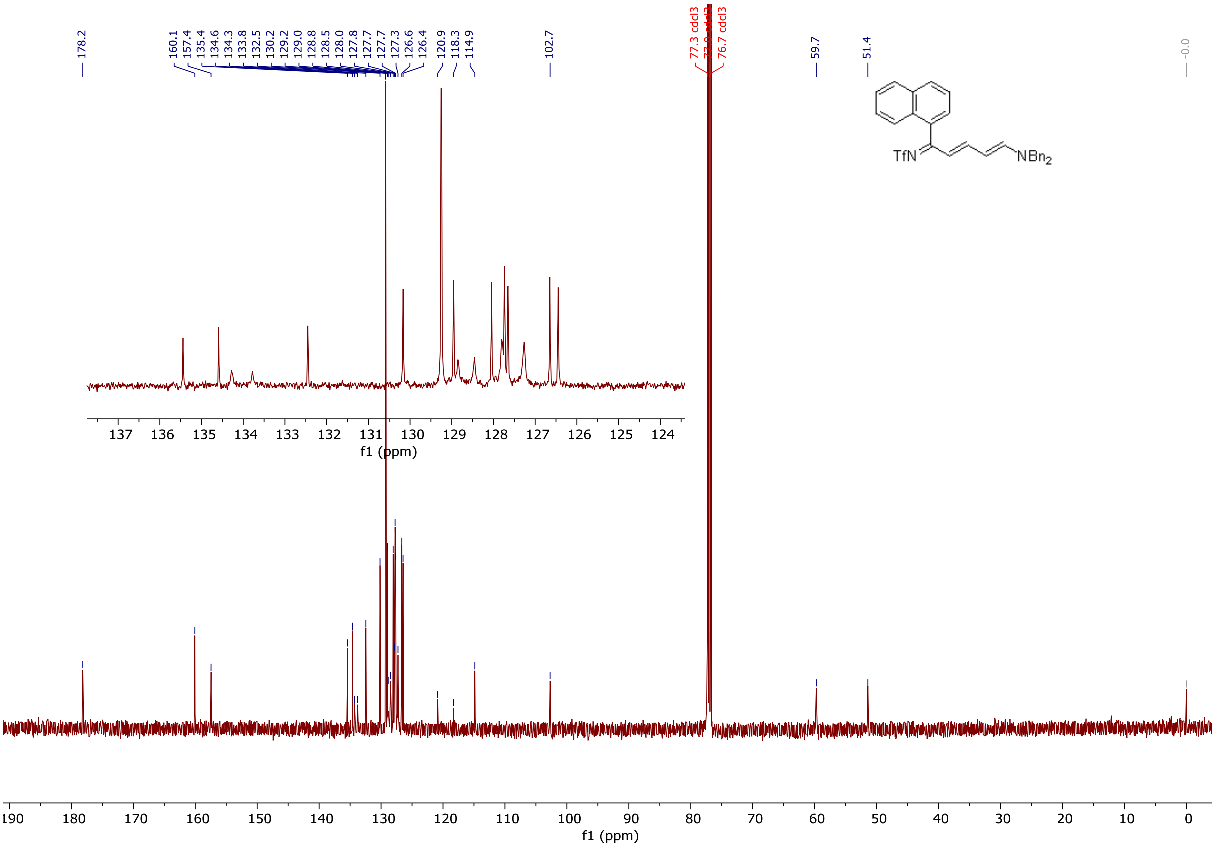

^{1}H NMR spectrum of Zincke imine S14b ($CDCl_3$, 298 K)

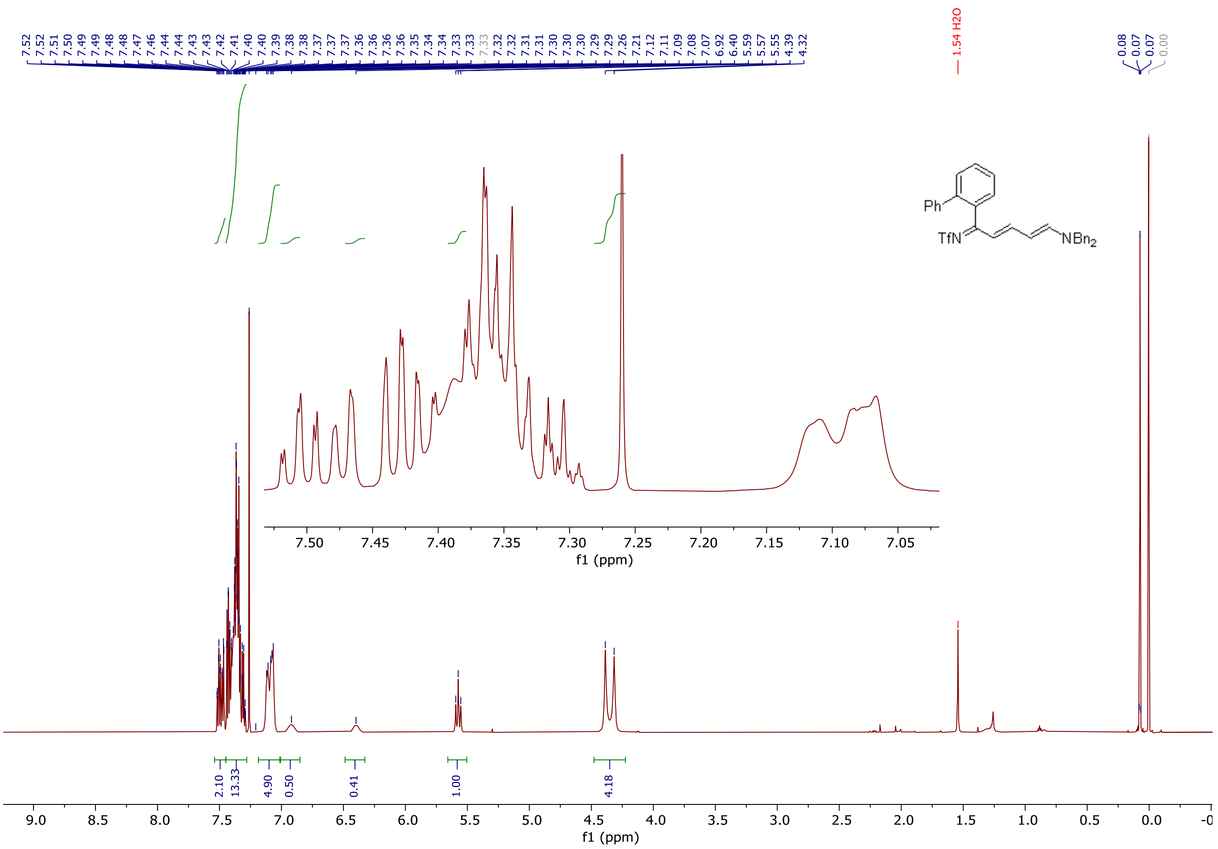

^{13}C NMR spectrum of Zincke imine S14b ($CDCl_3$, 298 K)

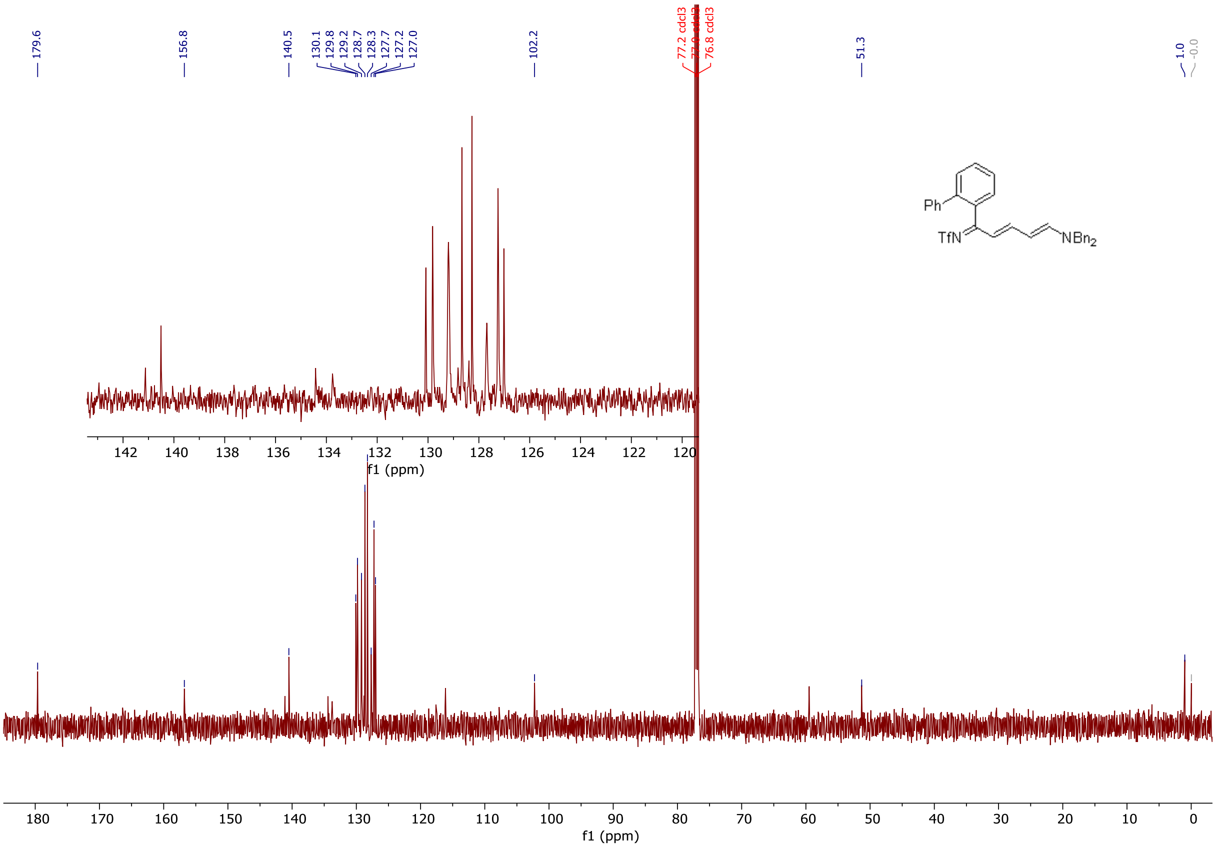

^{1}H NMR spectrum of Zincke imine S15b ($CDCl_3$, 298 K)

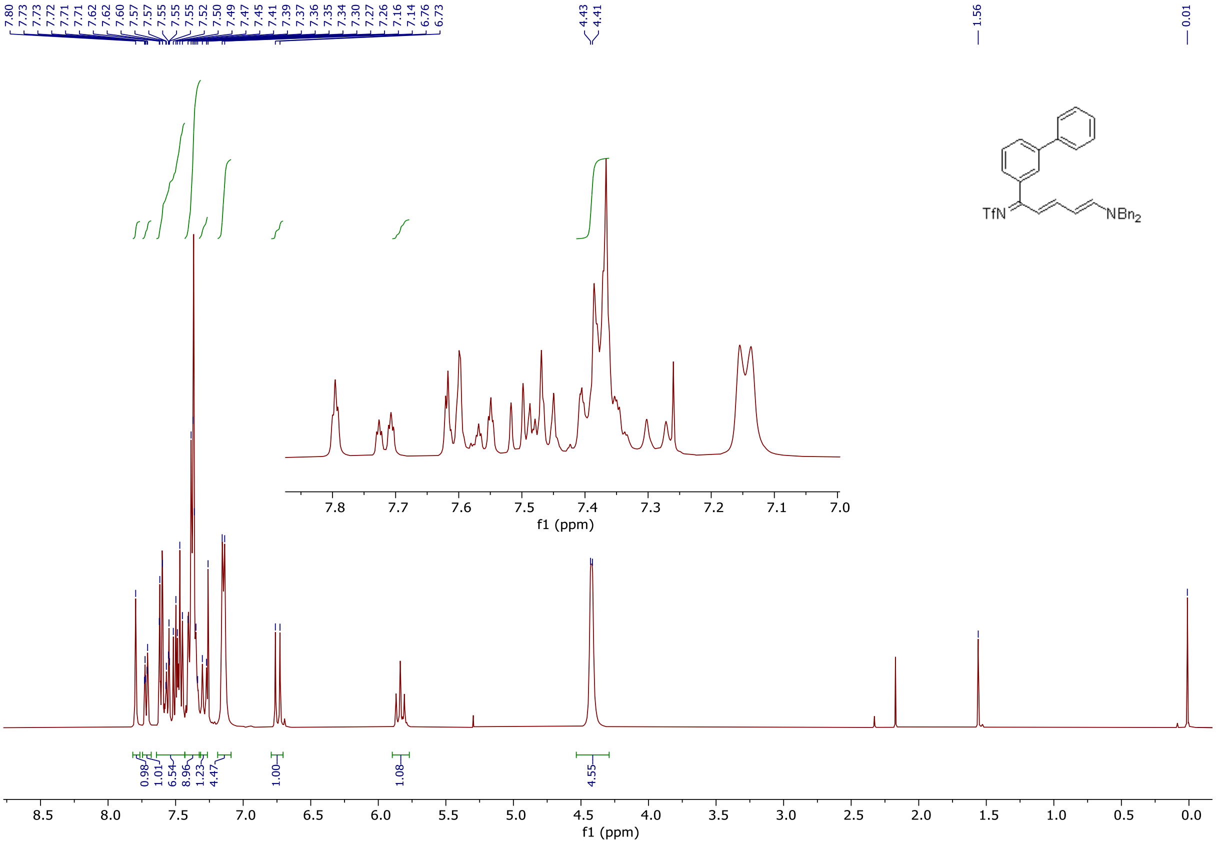

^{13}C NMR spectrum of Zincke imine S15b ($CDCl_3$, 298 K)

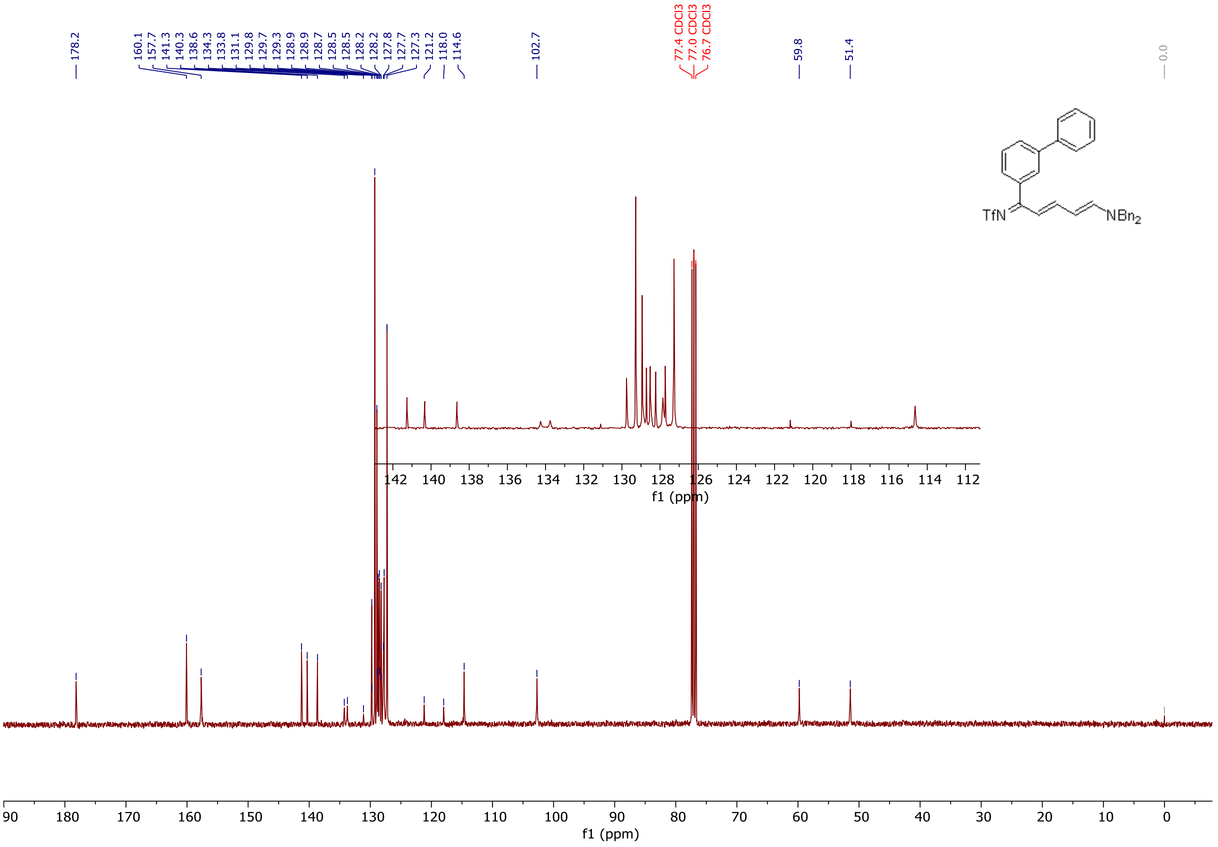

^{1}H NMR spectrum of Zincke imine S16b ($CDCl_3$, 298 K)

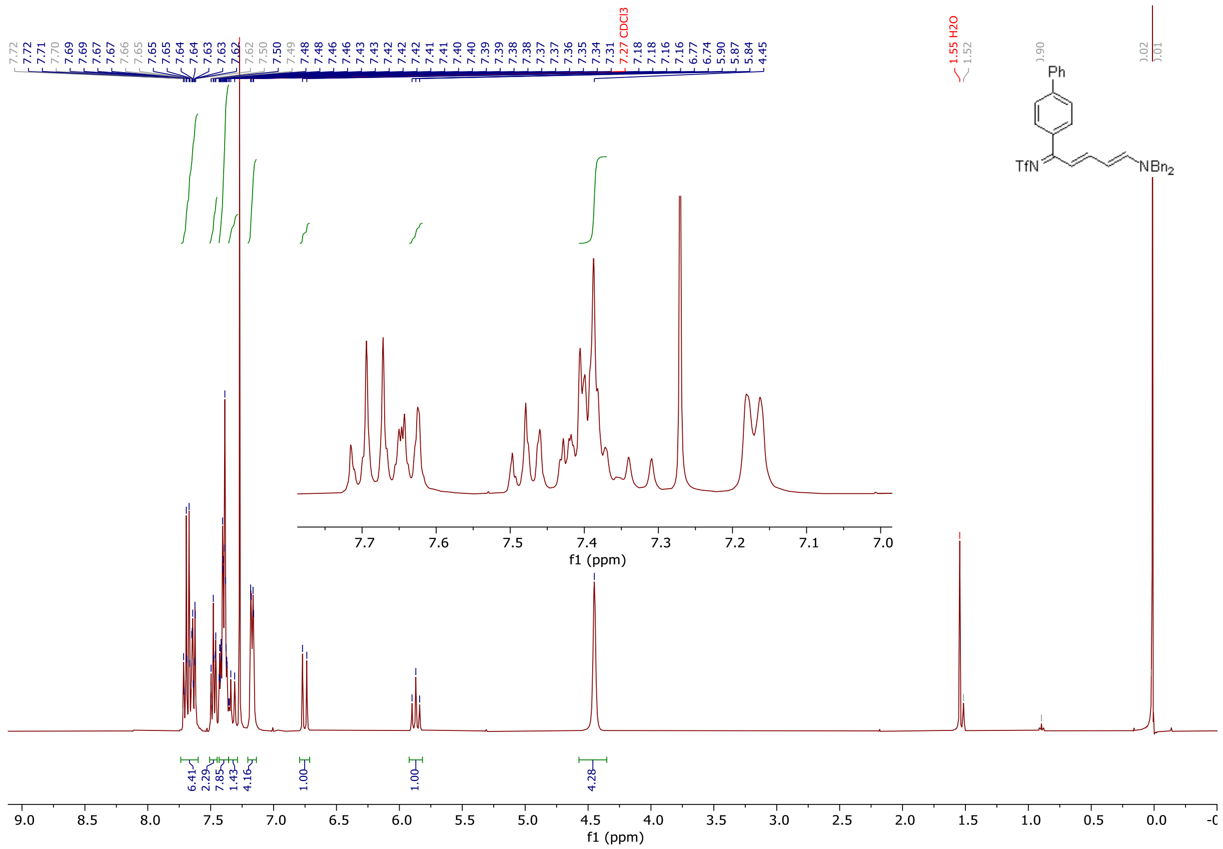

^{13}C NMR spectrum of Zincke imine S16b ($CDCl_3$, 298 K)

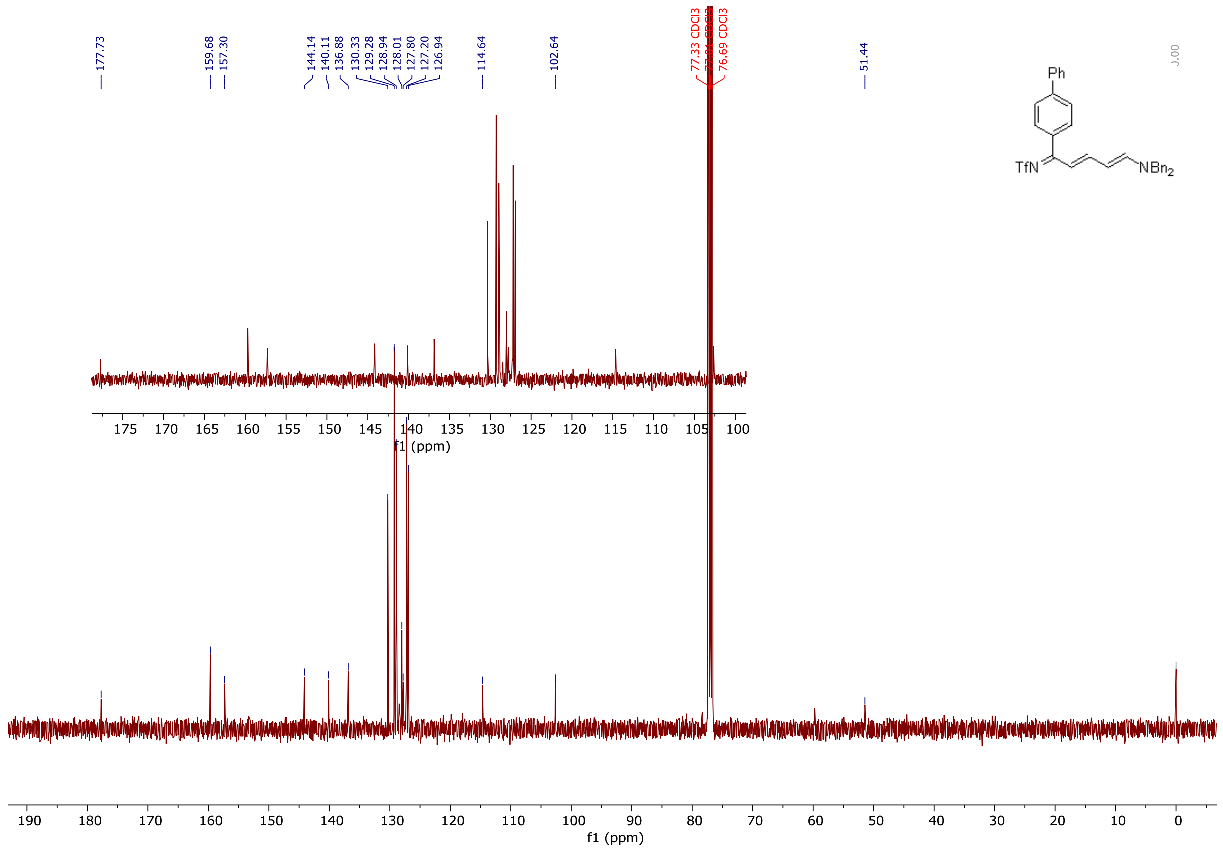

^{1}H NMR spectrum of Zincke imine S17b ($CDCl_3$, 298 K)

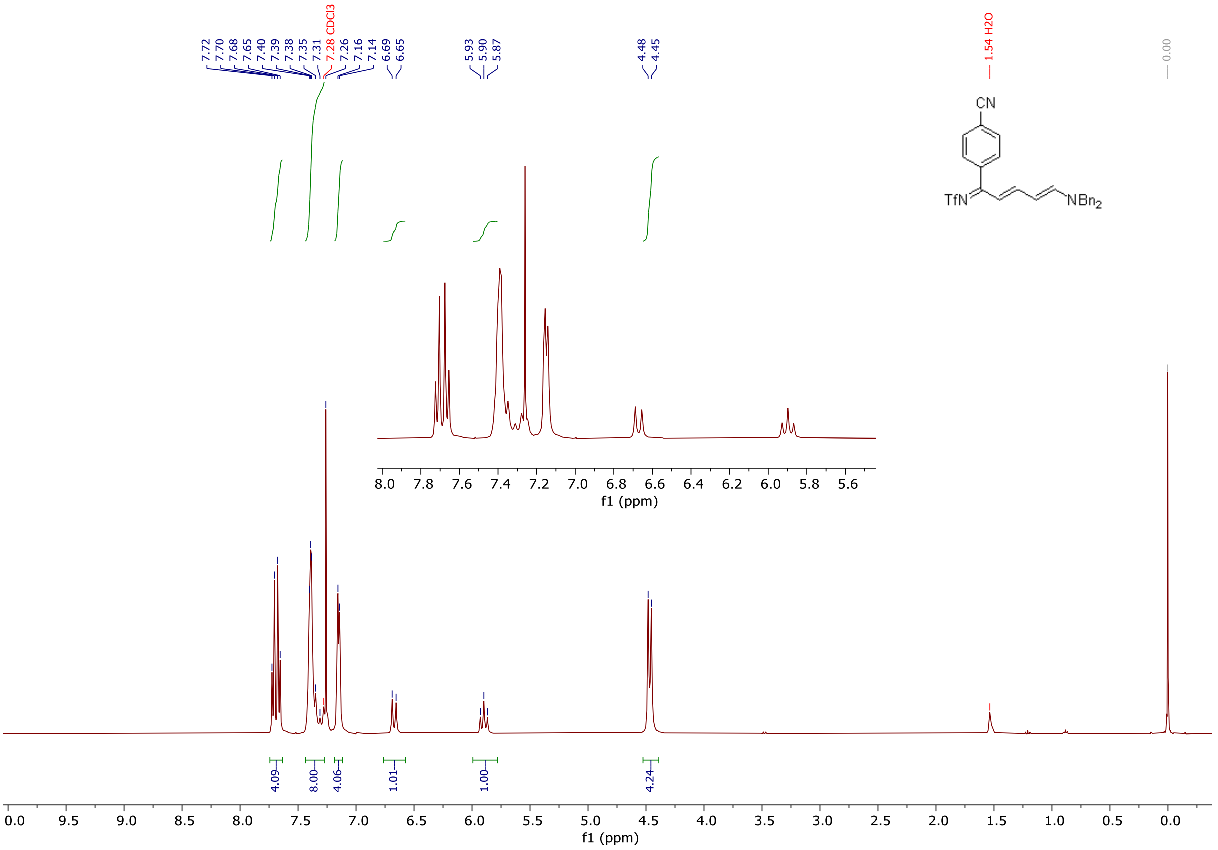

^{13}C NMR spectrum of Zincke imine S17b ($CDCl_3$, 298 K)

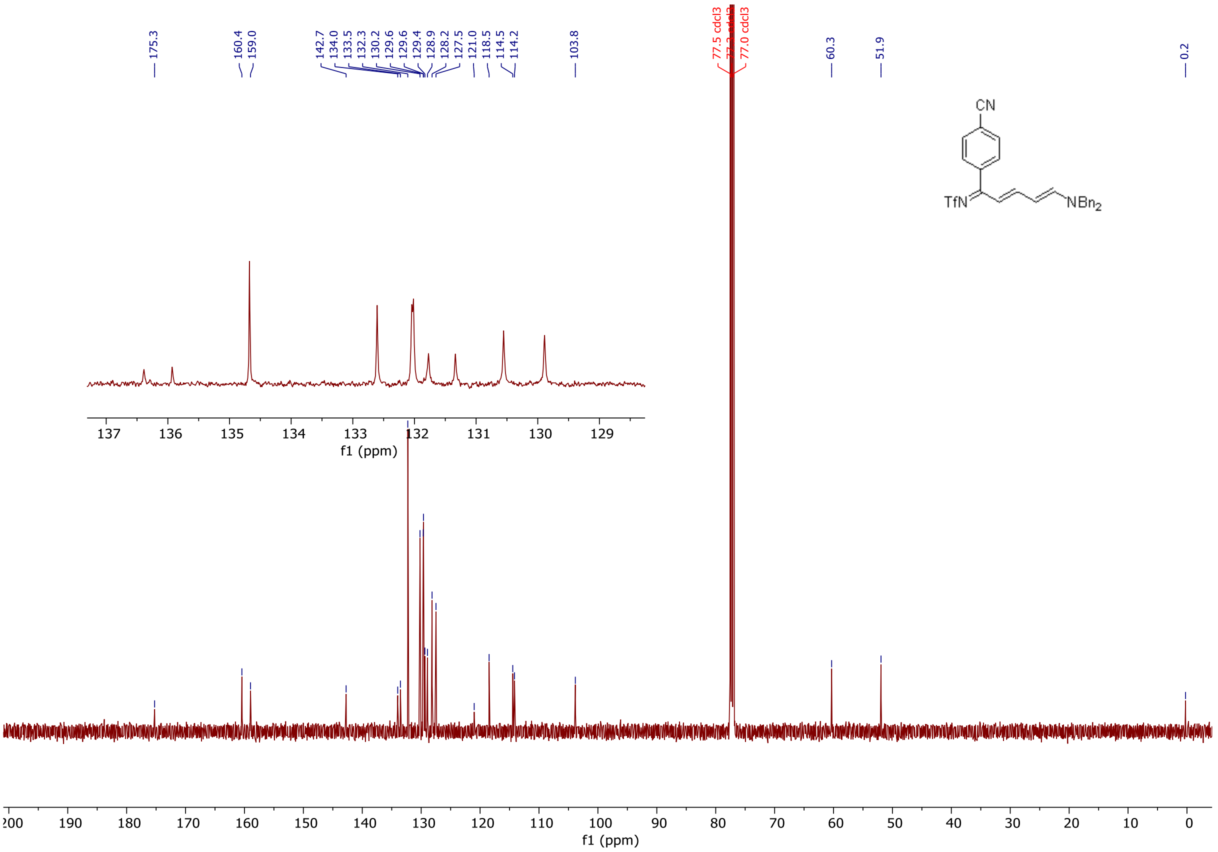

^{1}H NMR spectrum of Zincke imine S18b ($CDCl_3$, 298 K)

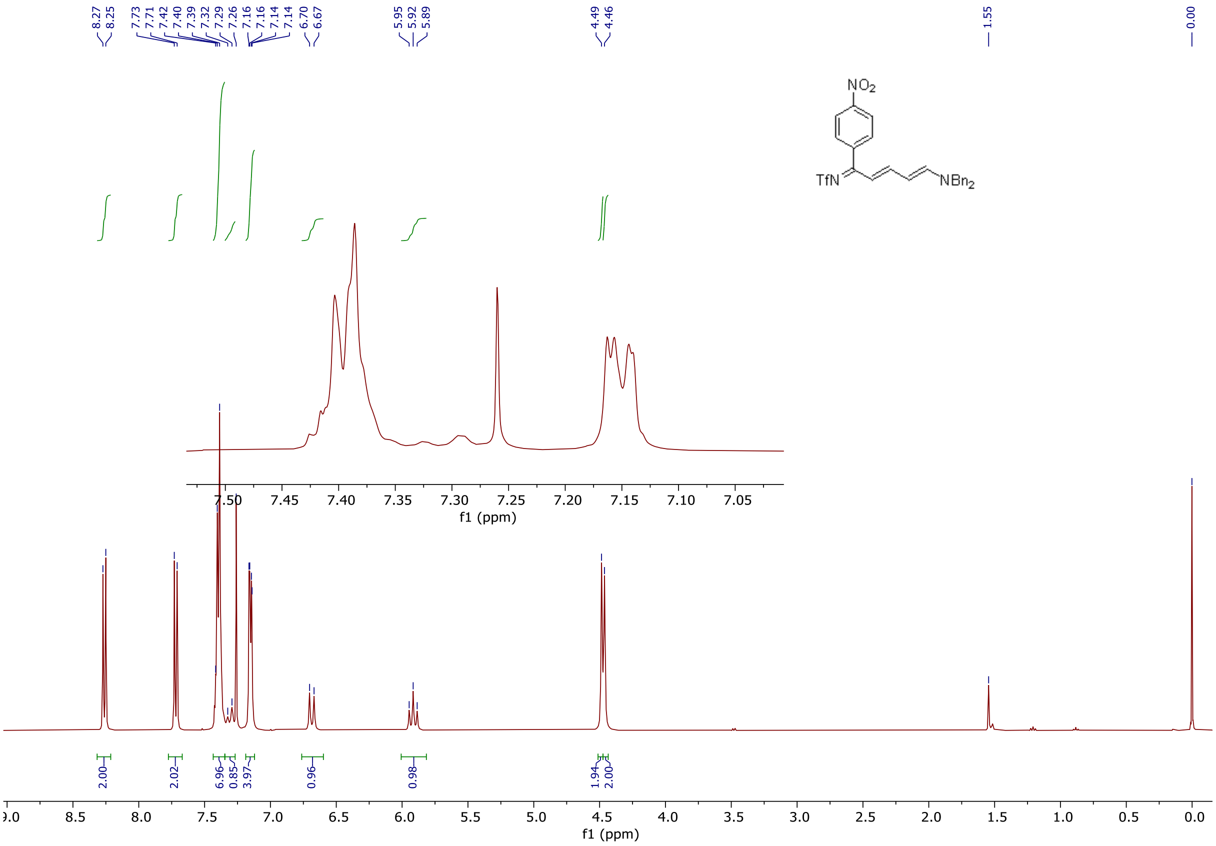

^{13}C NMR spectrum of Zincke imine S18b ($CDCl_3$, 298 K)

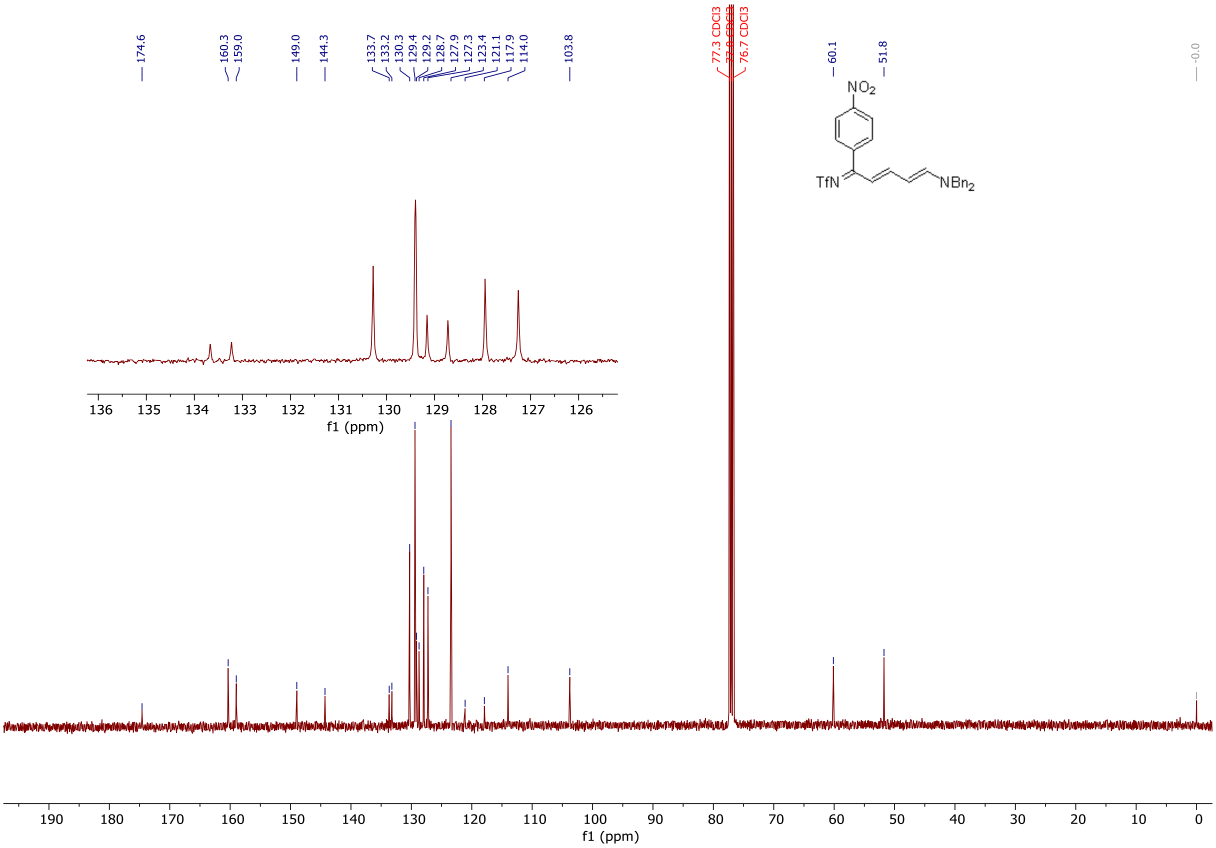

^{1}H NMR spectrum of Zincke imine S19b ($CDCl_3$, 298 K)

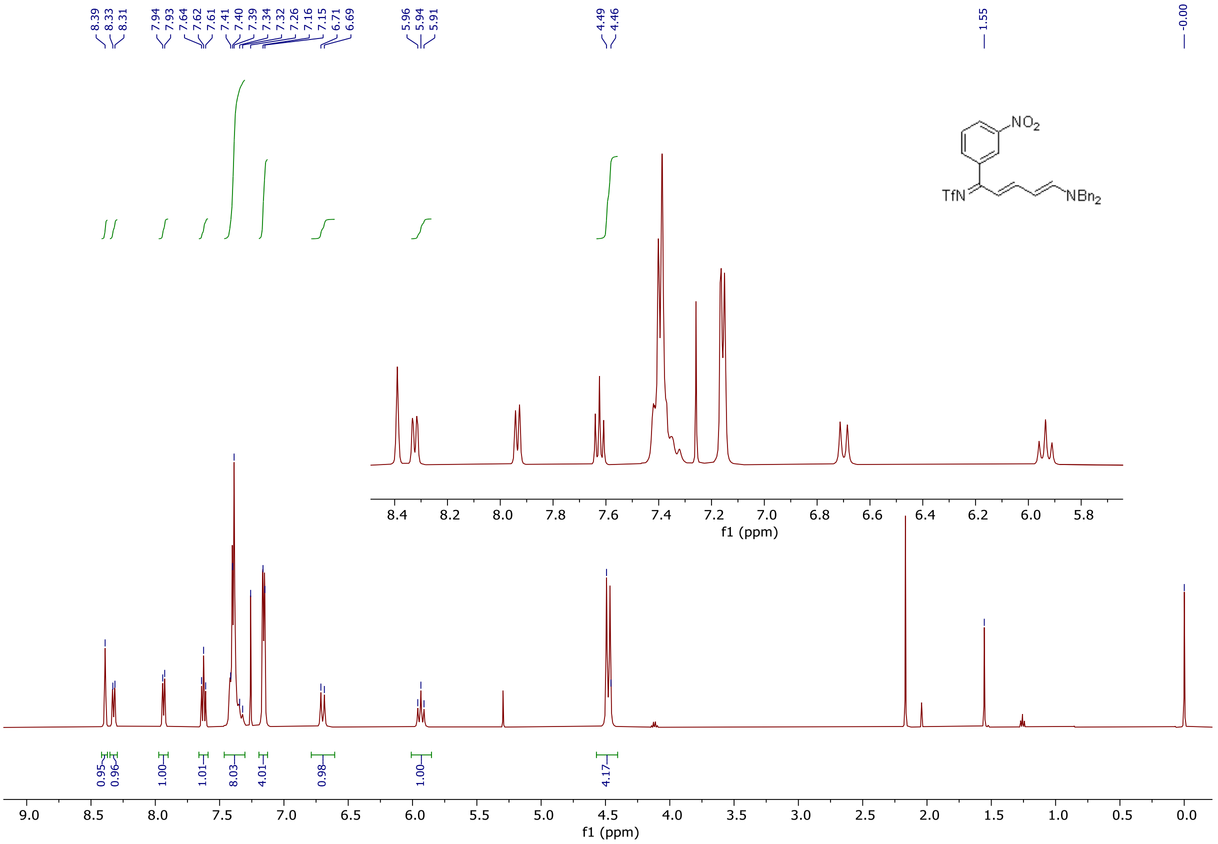

^{13}C NMR spectrum of Zincke imine S19b ($CDCl_3$, 298 K)

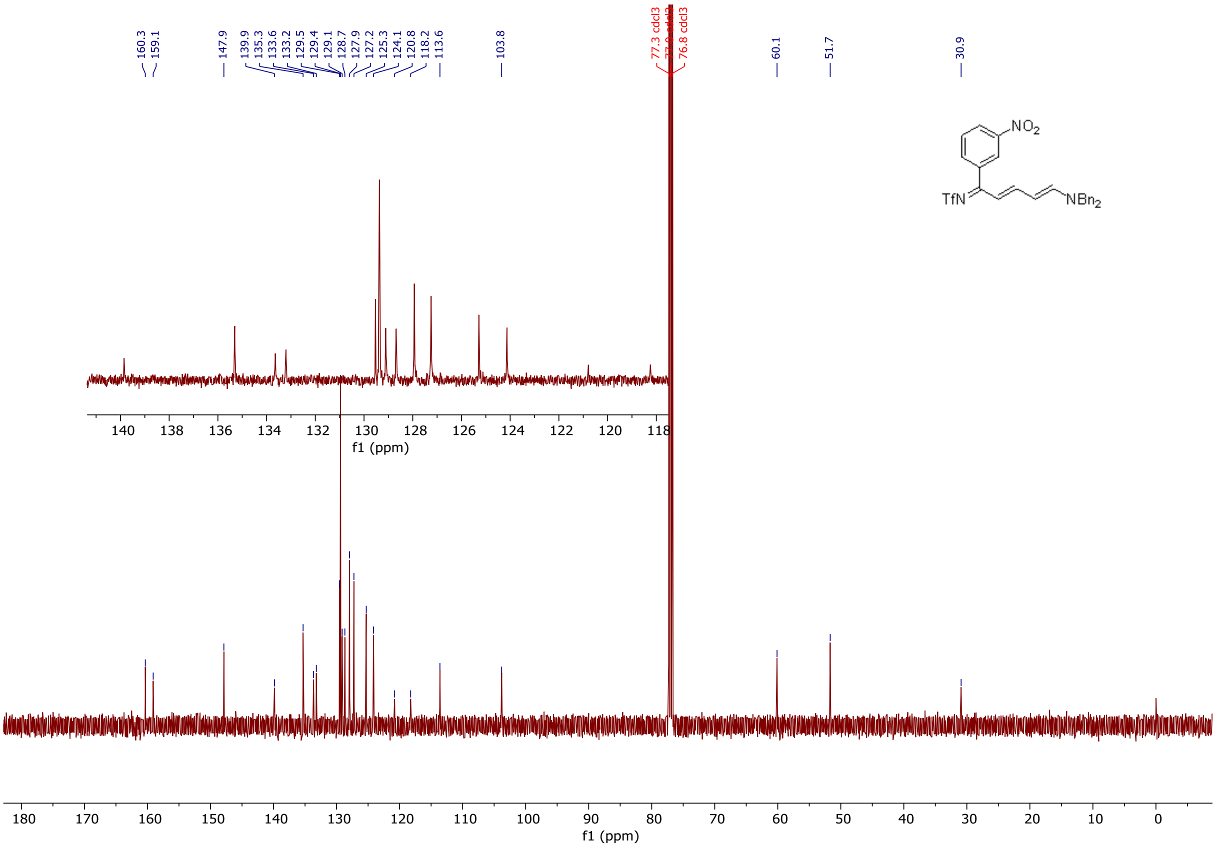

^{1}H NMR spectrum of Zincke imine S20b ($CDCl_3$, 298 K)

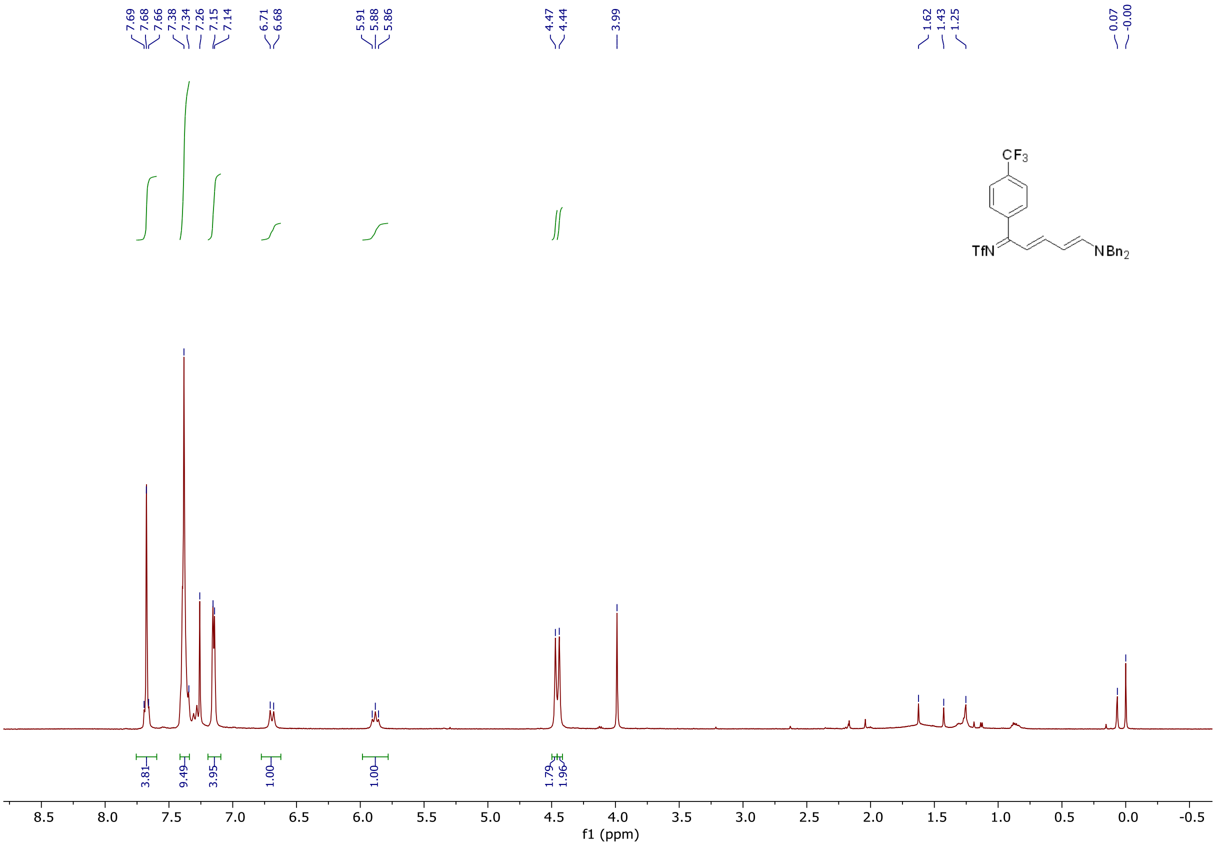

^{13}C NMR spectrum of Zincke imine S20b ($CDCl_3$, 298 K)

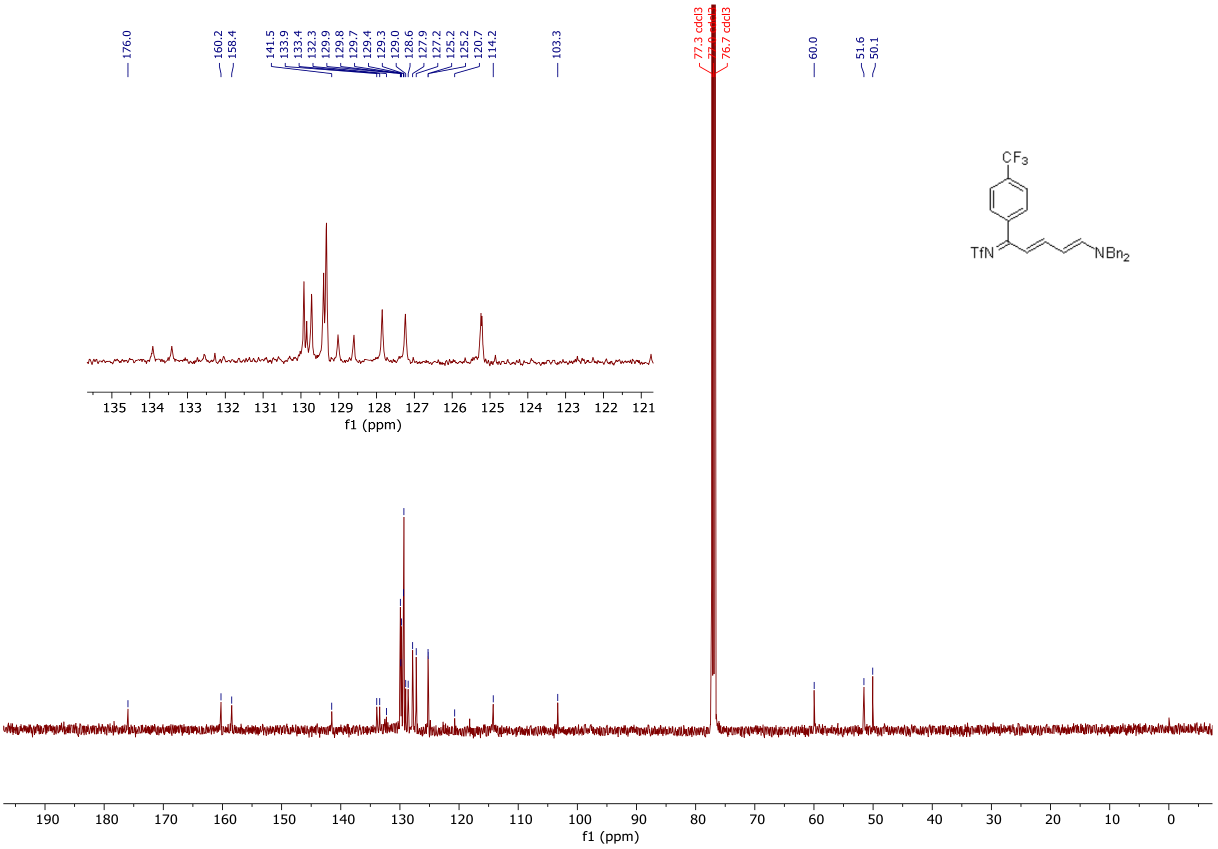

^{1}H NMR spectrum of Zincke imine S21b ($CDCl_3$, 298 K)

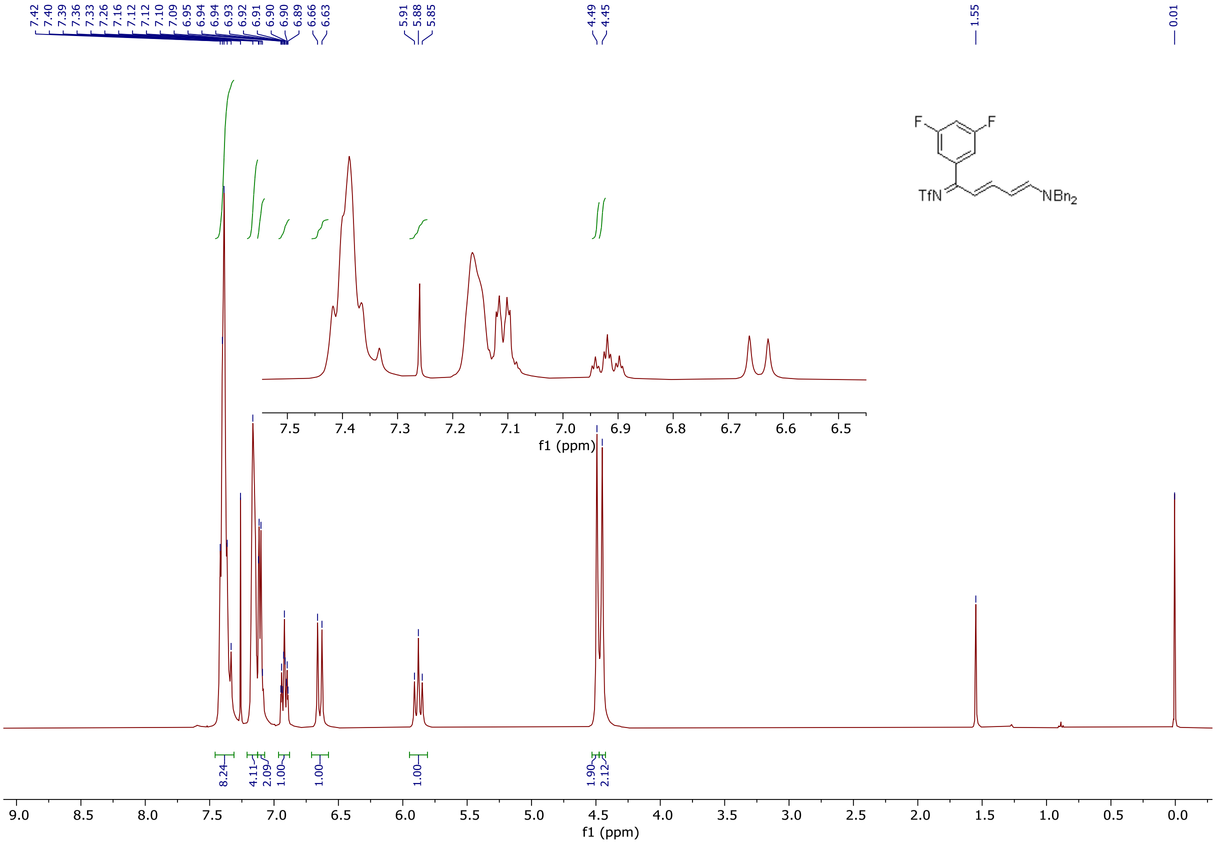

^{13}C NMR spectrum of Zincke imine S21b ($CDCl_3$, 298 K)

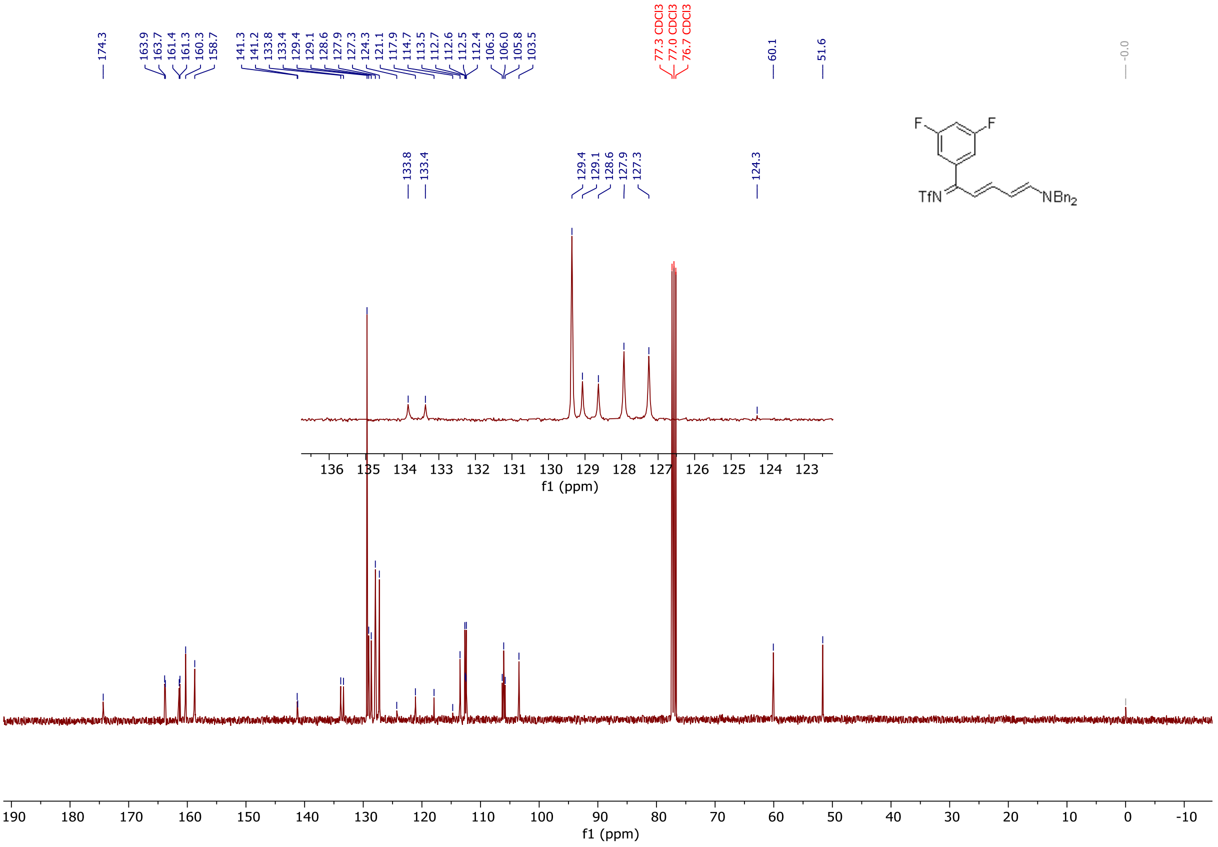

^{1}H NMR spectrum of Zincke imine S22b ($CDCl_3$, 298 K)

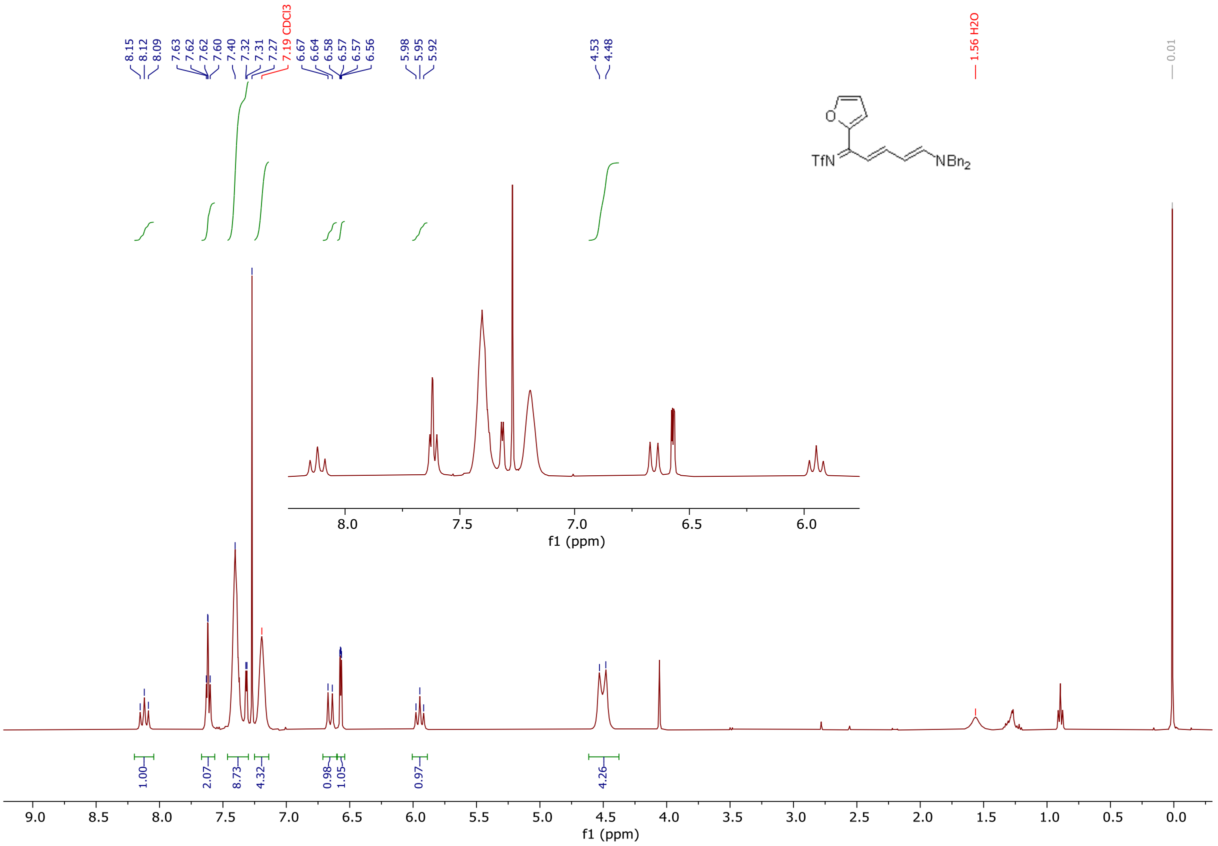

^{13}C NMR spectrum of Zincke imine S22b ($CDCl_3$, 298 K)

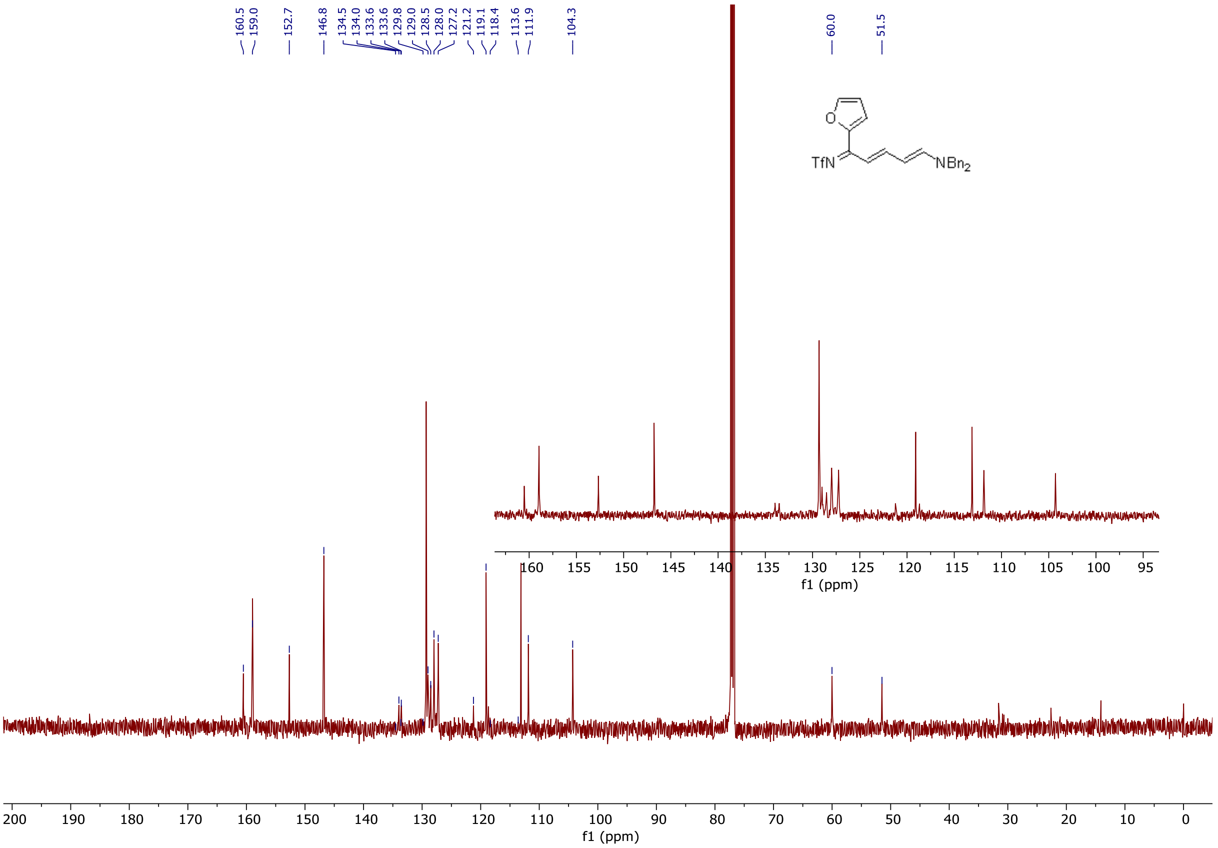

^{1}H NMR spectrum of Zincke imine S23b ($CDCl_3$, 298 K)

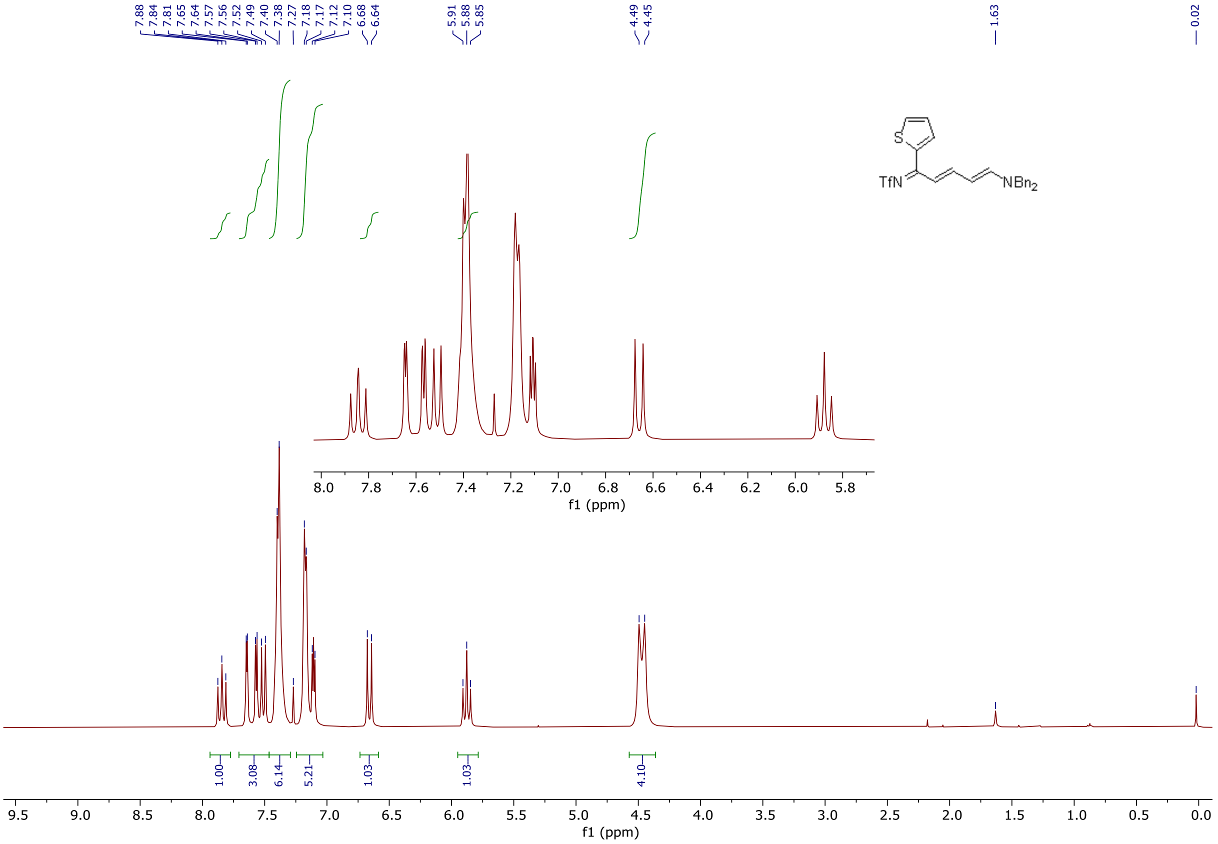

^{13}C NMR spectrum of Zincke imine S23b ($CDCl_3$, 298 K)

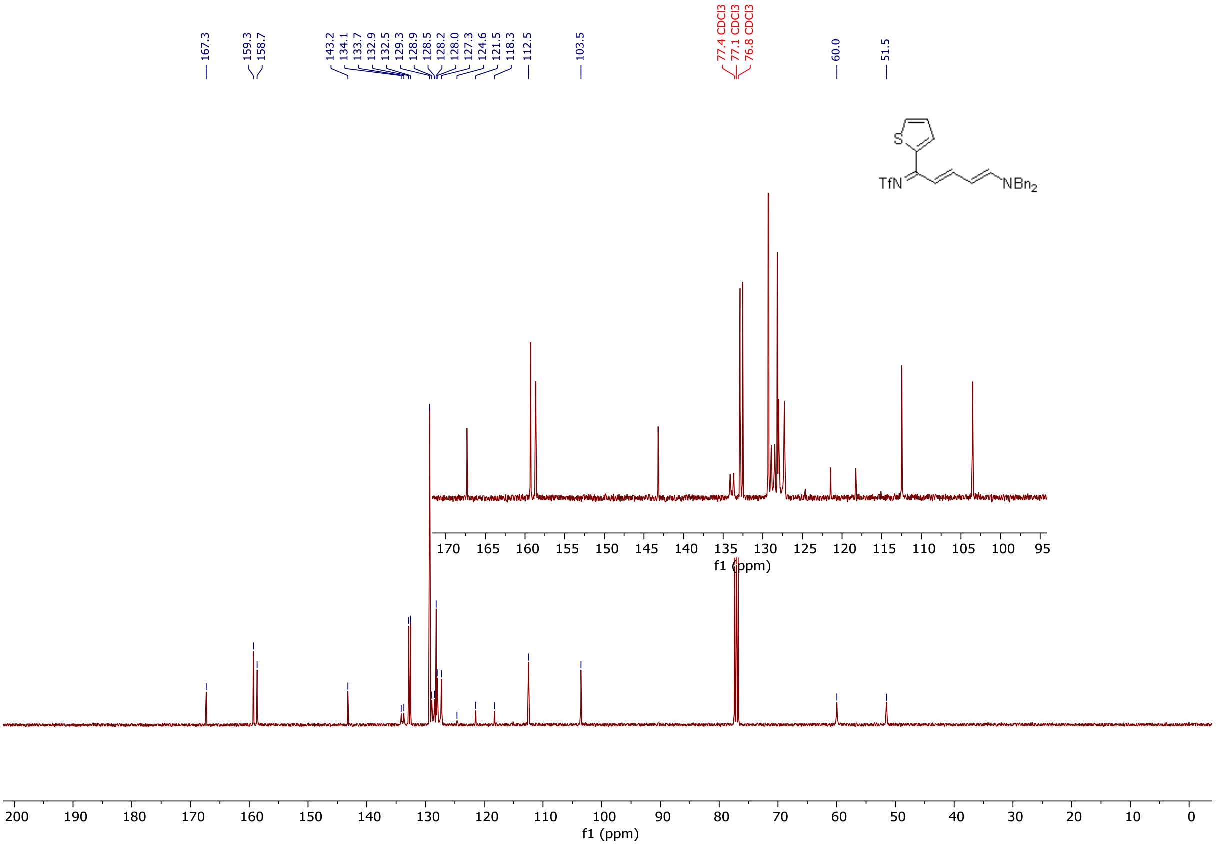

^{1}H NMR spectrum of Zincke imine S24b ($CDCl_3$, 298 K)

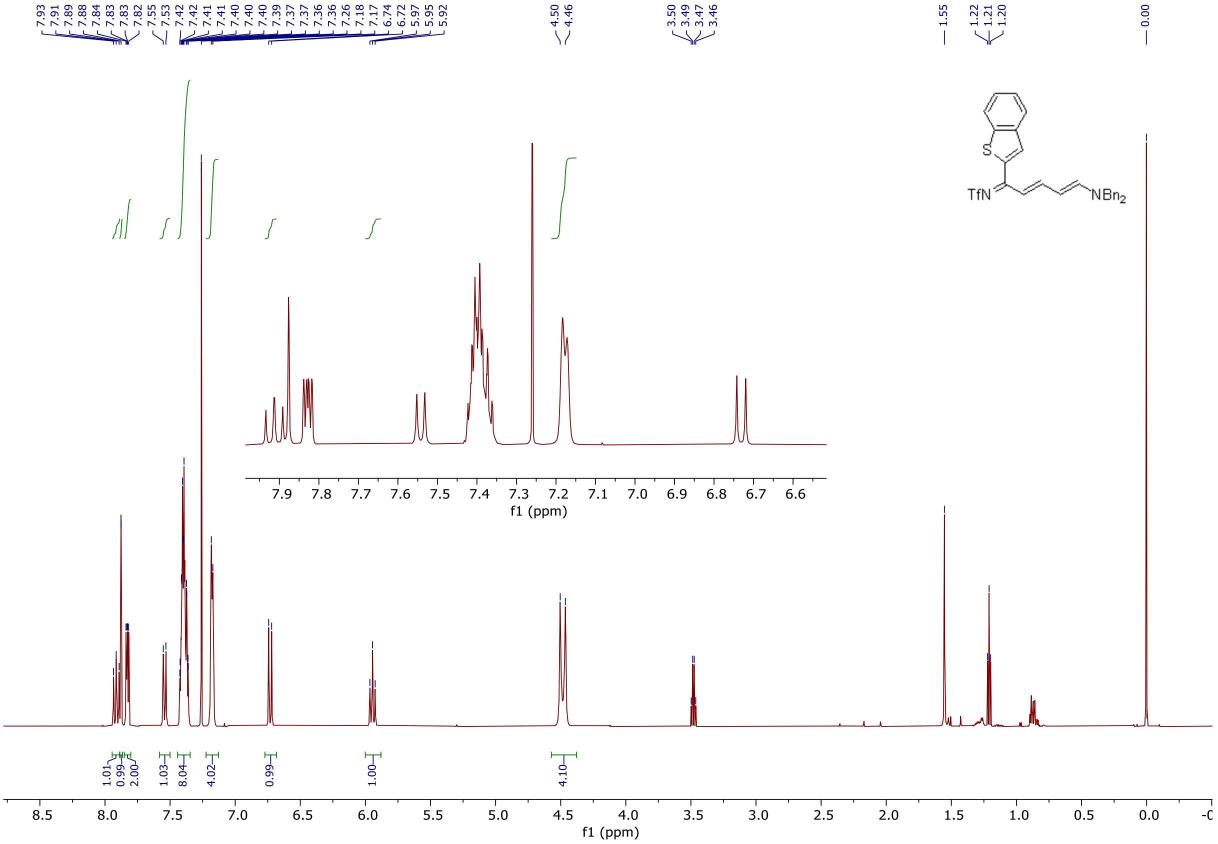

^{13}C NMR spectrum of Zincke imine S24b ($CDCl_3$, 298 K)

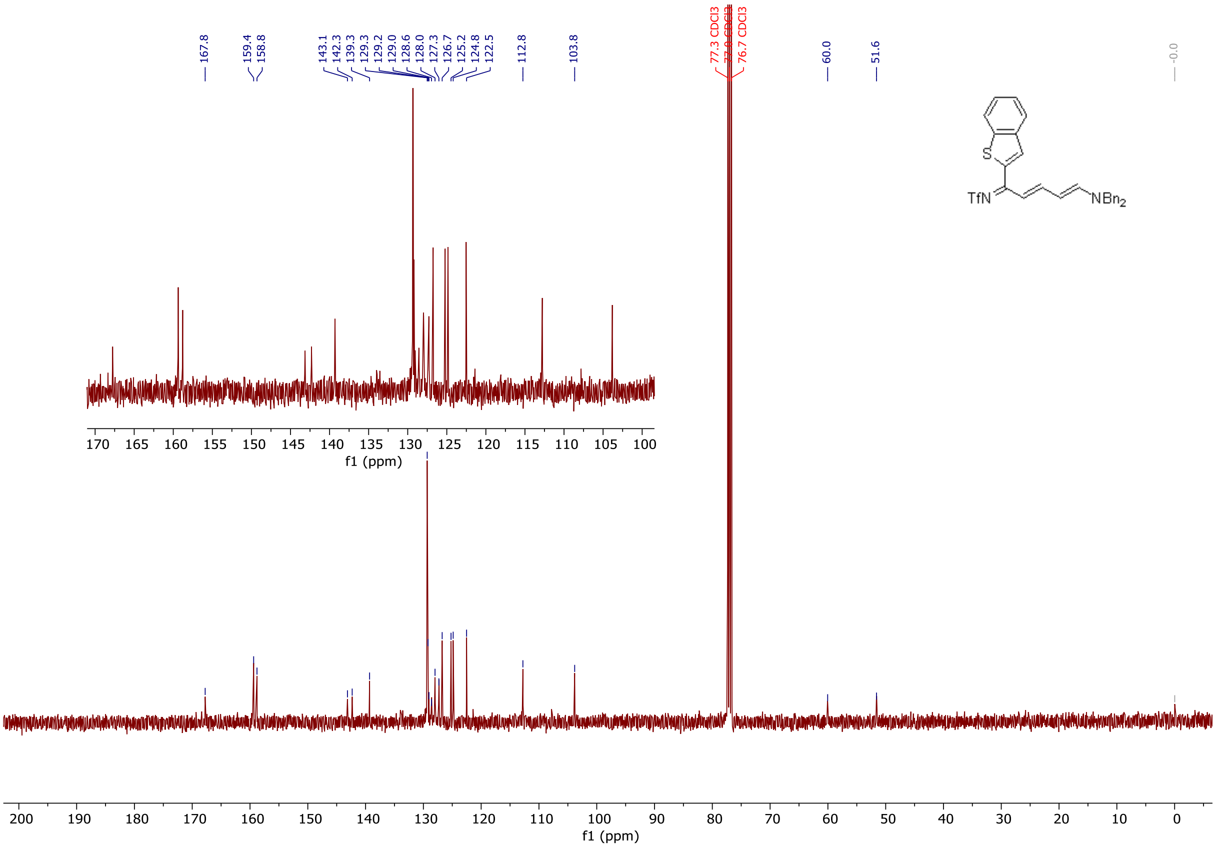

^{1}H NMR spectrum of Zincke imine S25b ($CDCl_3$, 298 K)

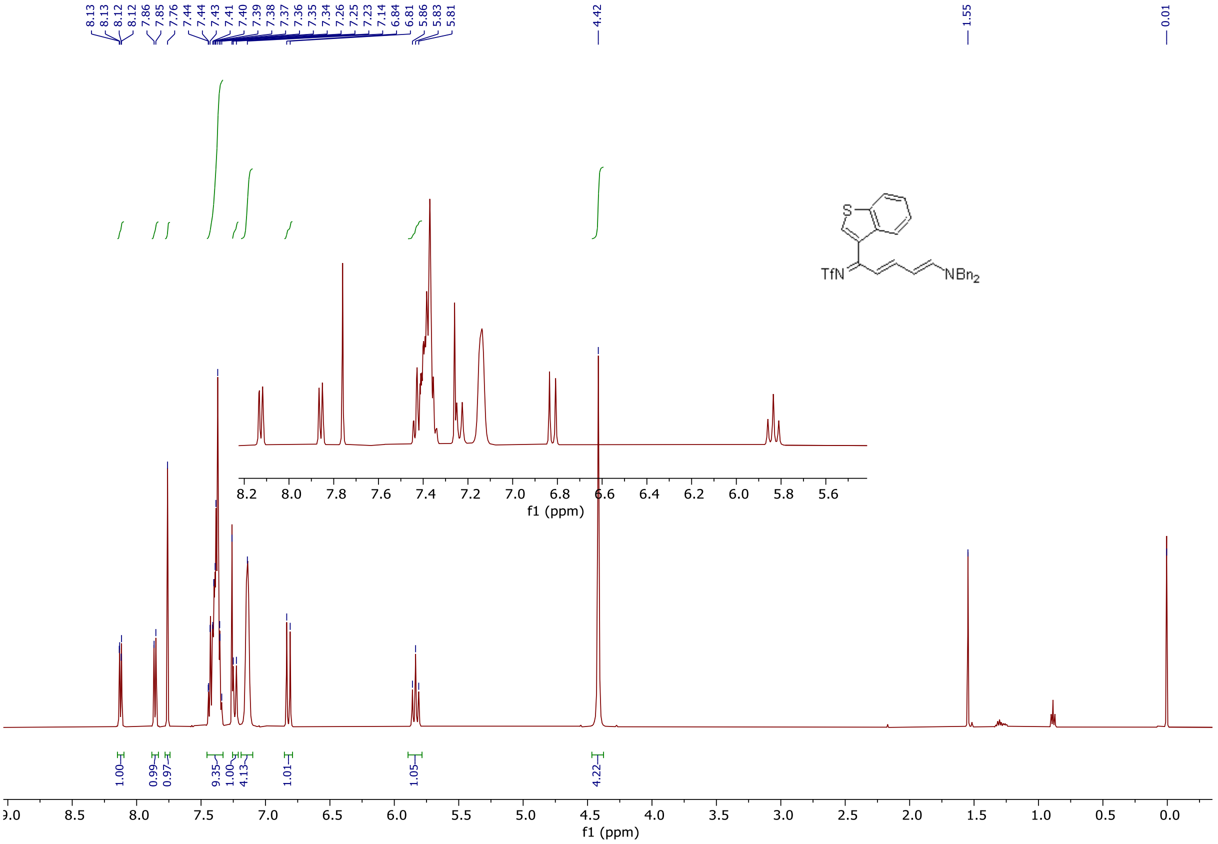

^{13}C NMR spectrum of Zincke imine S25b ($CDCl_3$, 298 K)

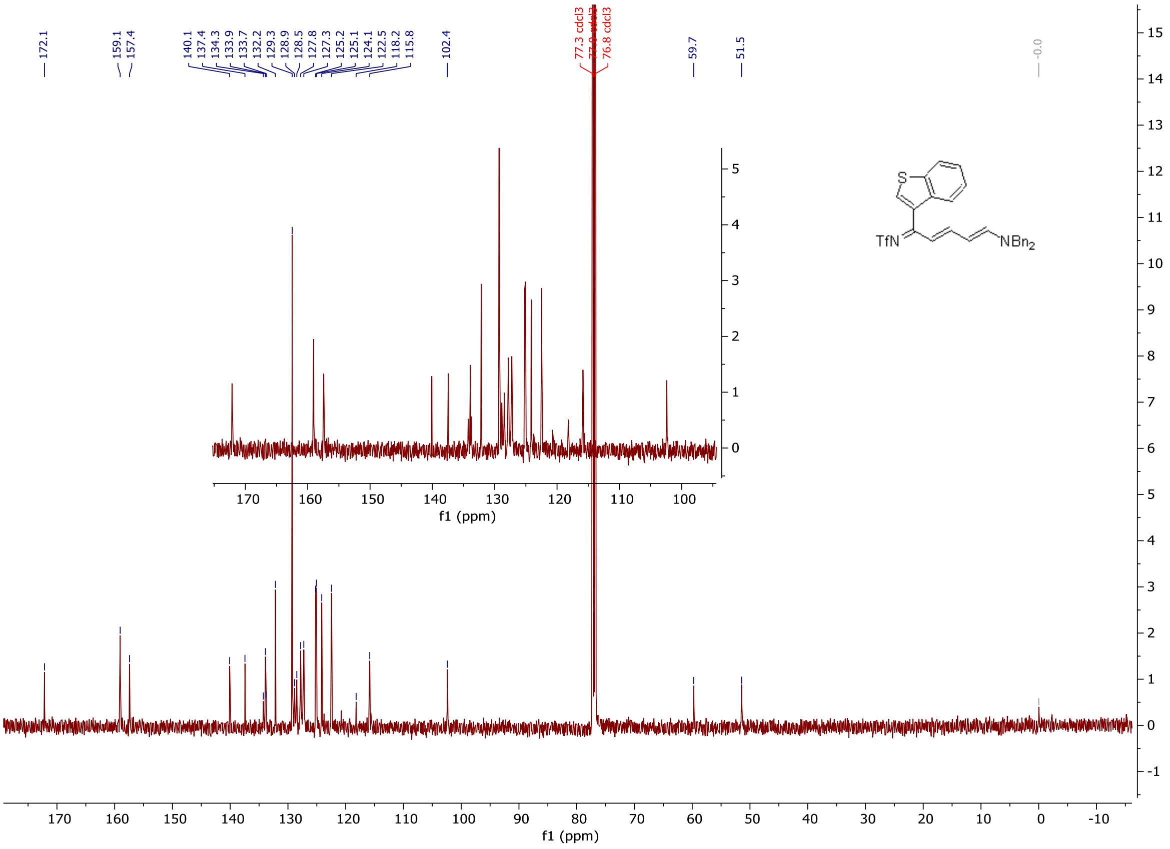

^{1}H NMR spectrum of Zincke imine S26b ($CDCl_3$, 298 K)

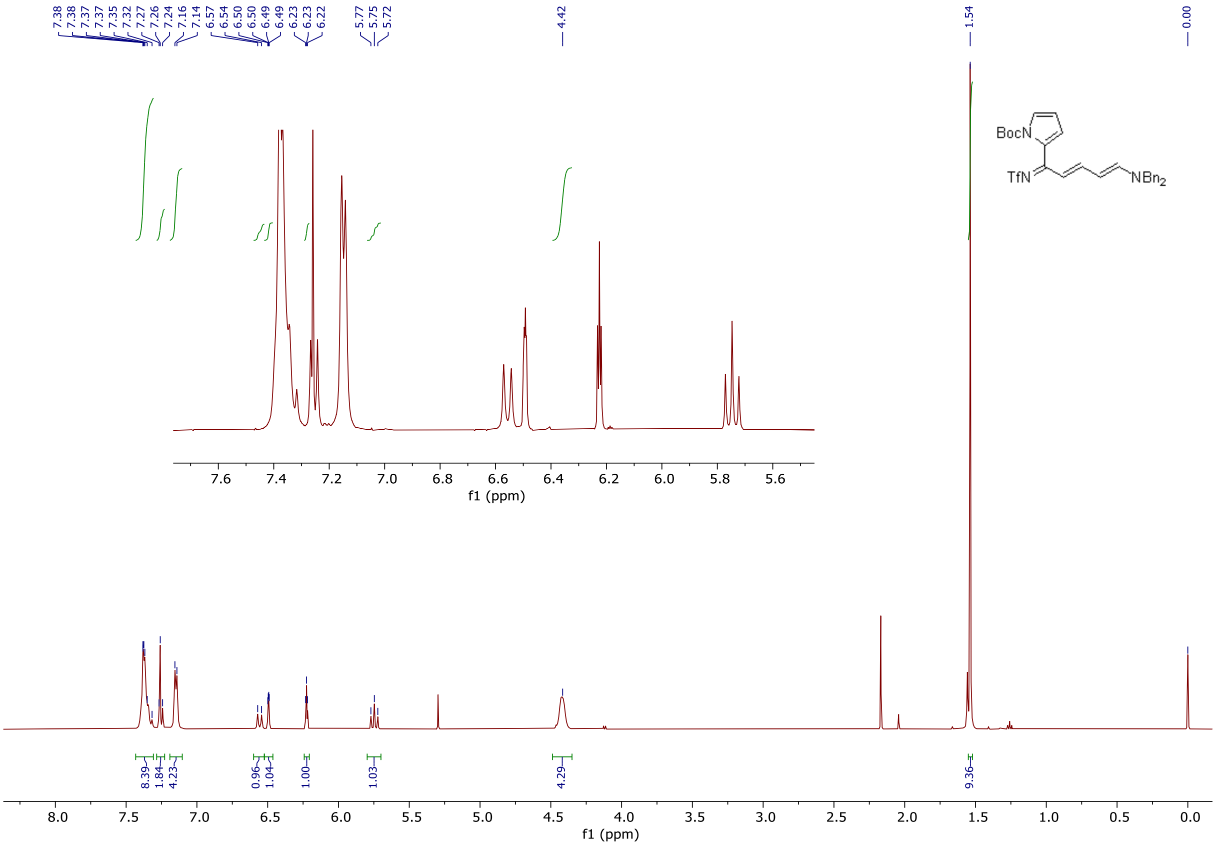

^{13}C NMR spectrum of Zincke imine S26b ($CDCl_3$, 298 K)

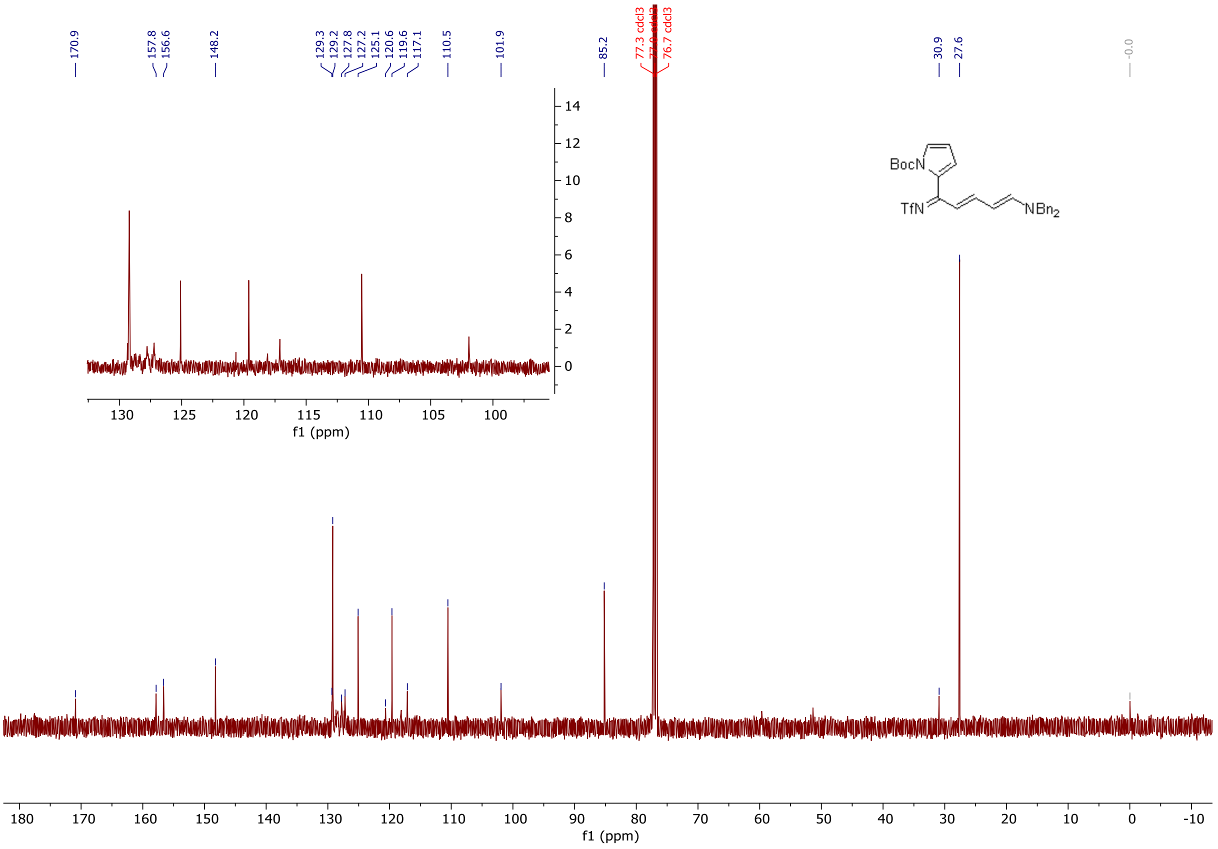

^{1}H NMR spectrum of Zincke imine S27b (CDCl$_3$, 298 K)

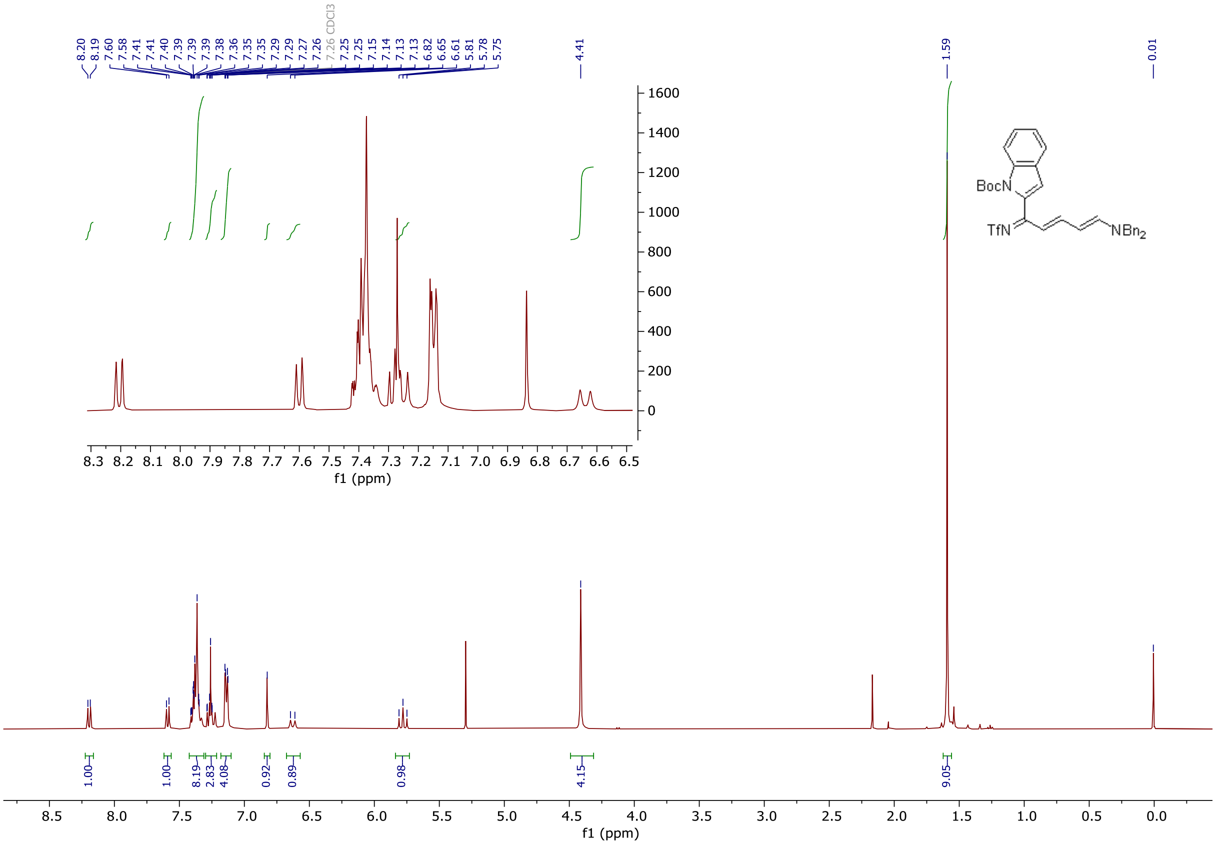

^{13}C NMR spectrum of Zincke imine S27b (CDCl$_3$, 298 K)

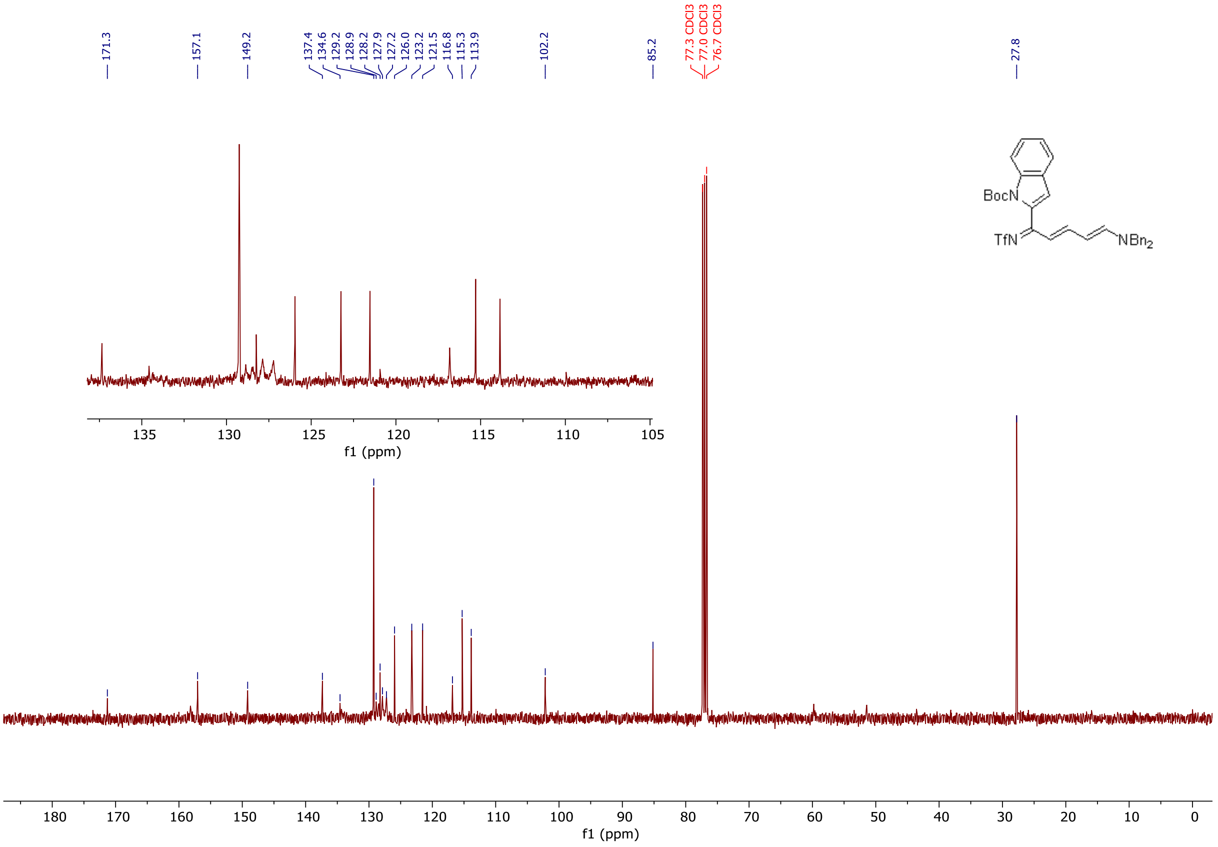

^{1}H NMR spectrum of Zincke imine S28b ($CDCl_3$, 298 K)

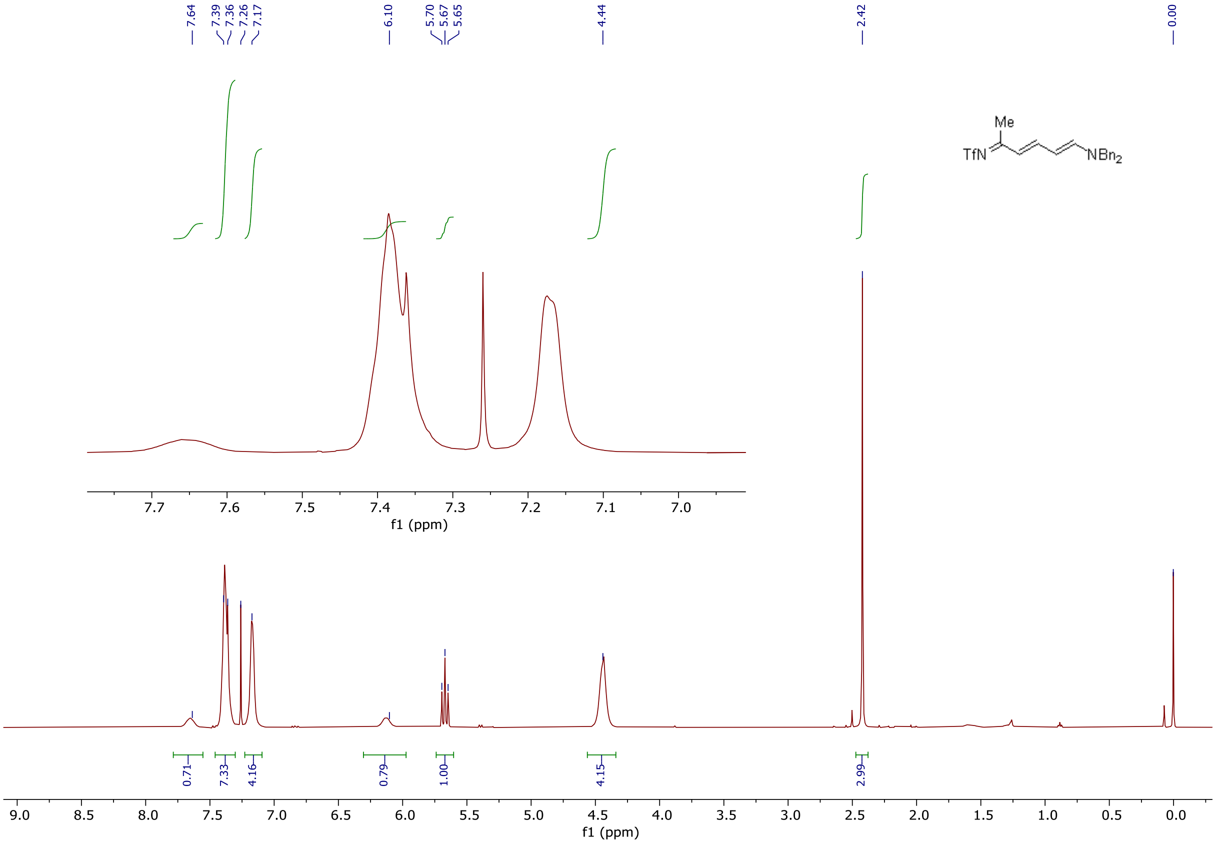

^{13}C NMR spectrum of Zincke imine S28b ($CDCl_3$, 298 K)

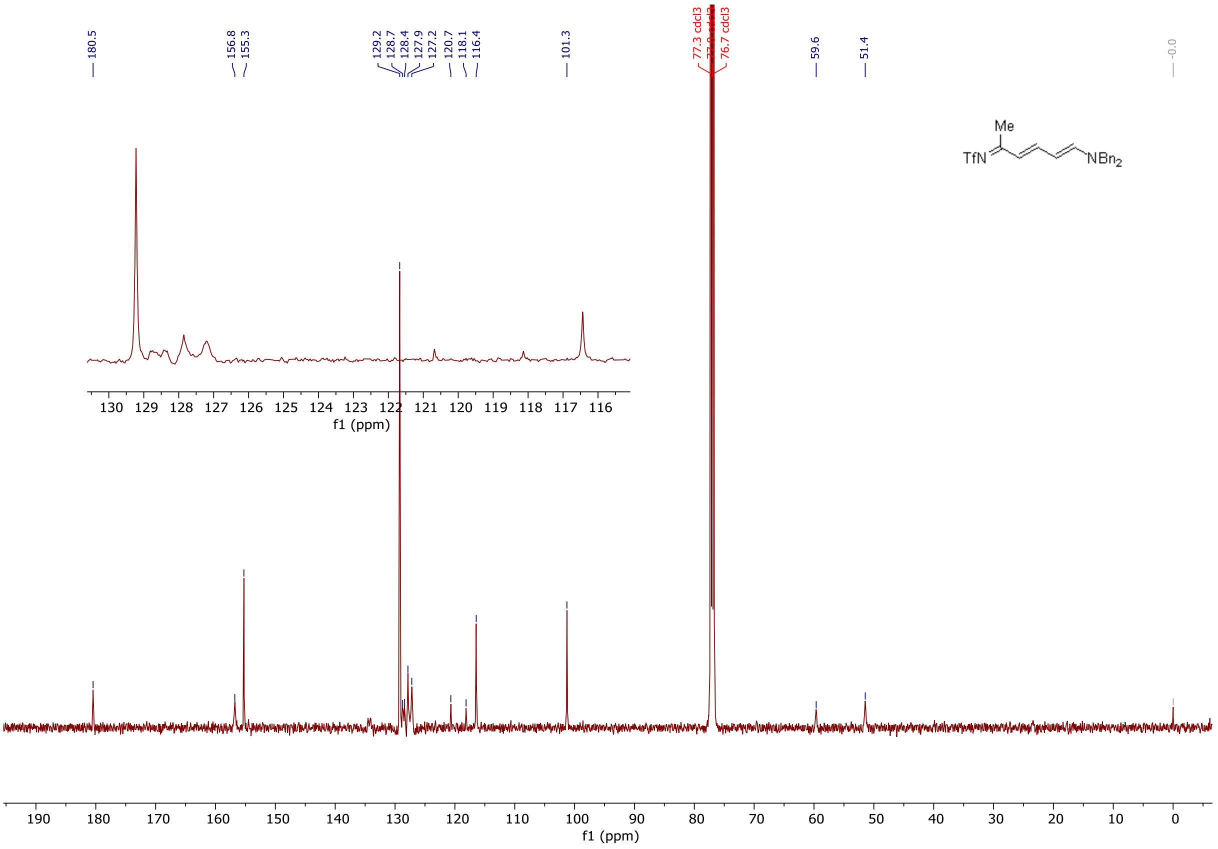

^{1}H NMR spectrum of Zincke imine S29b ($CDCl_3$, 298 K)

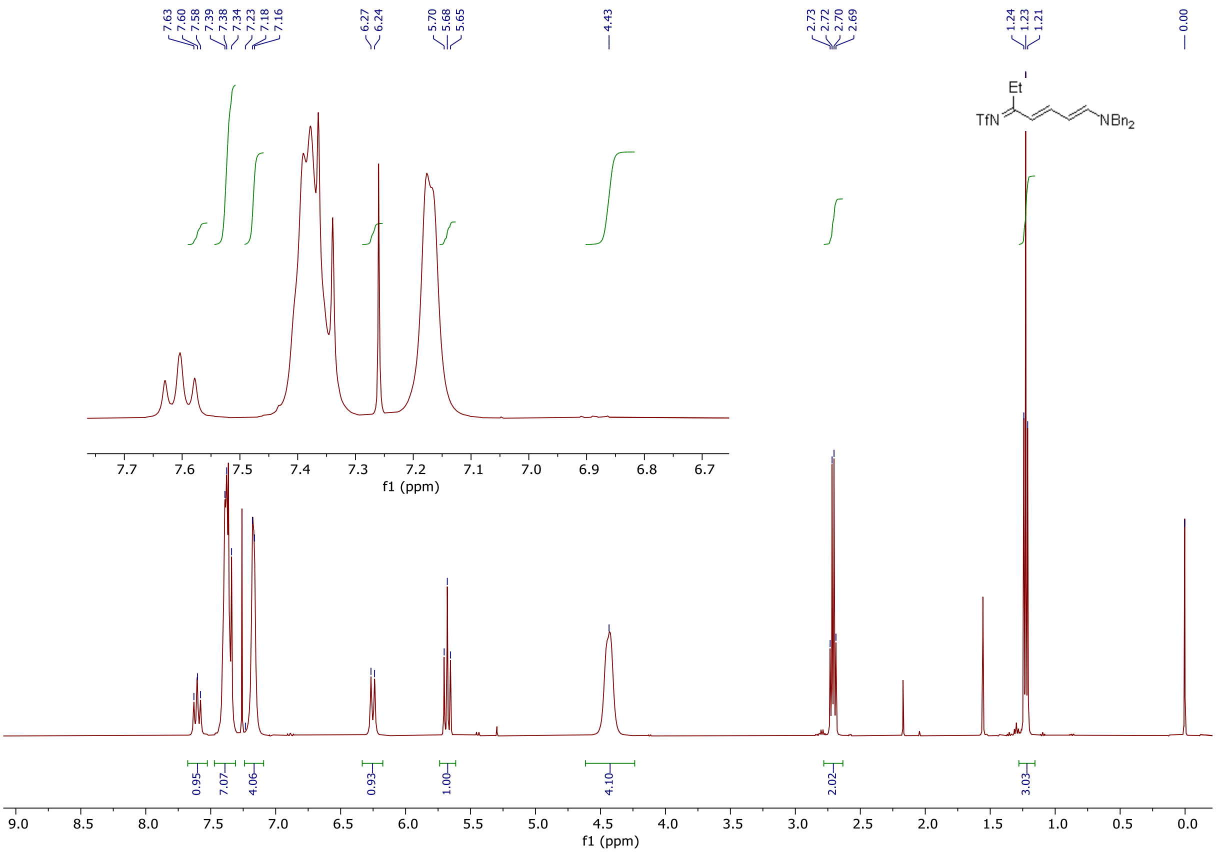

^{13}C NMR spectrum of Zincke imine S29b ($CDCl_3$, 298 K)

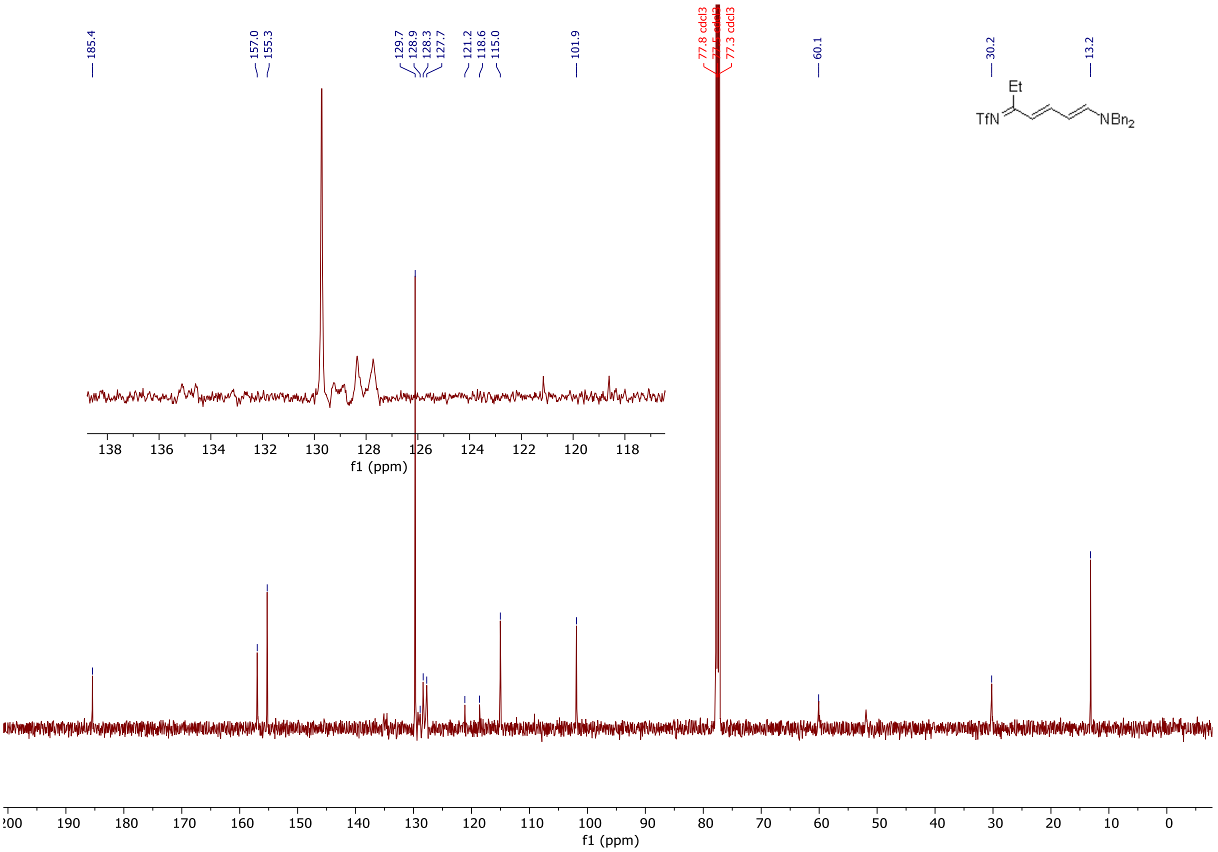

^{1}H NMR spectrum of Zincke imine S30b ($CDCl_3$, 298 K)

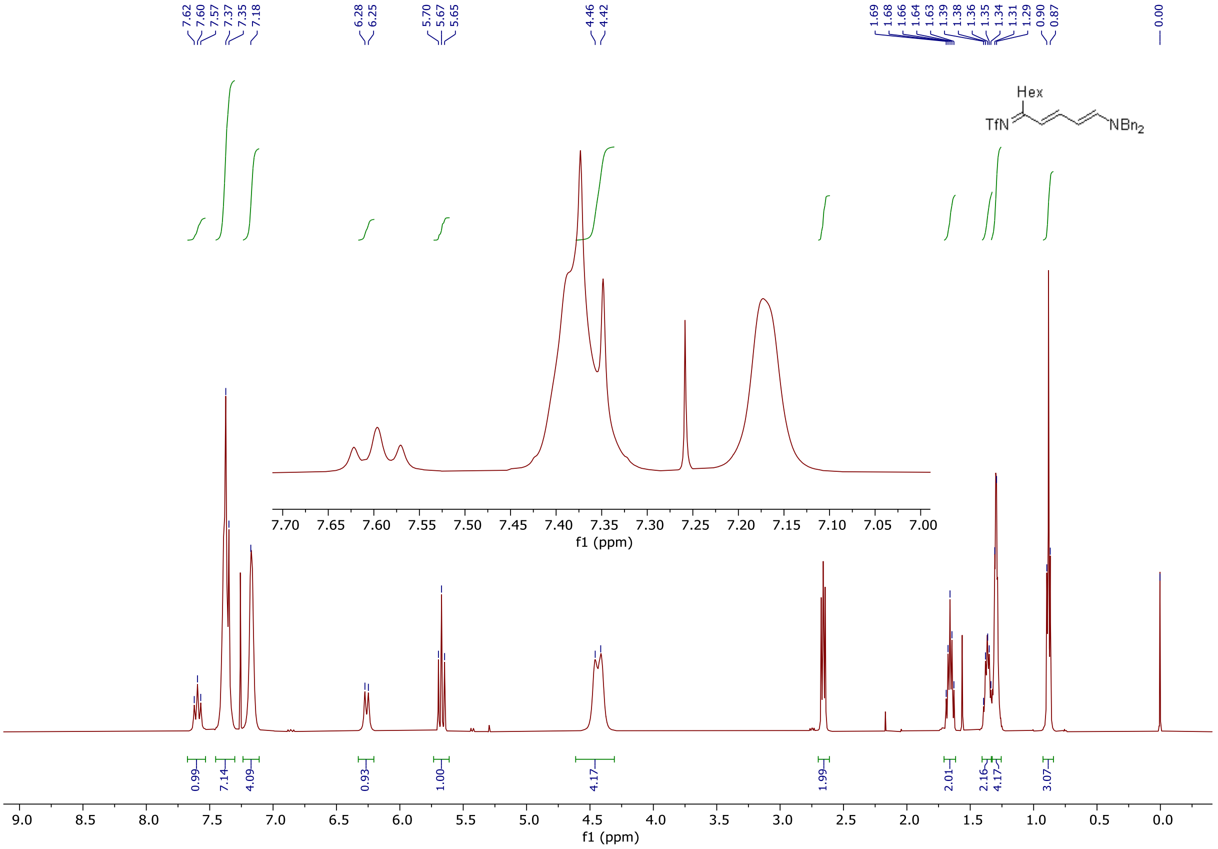

^{13}C NMR spectrum of Zincke imine S30b ($CDCl_3$, 298 K)

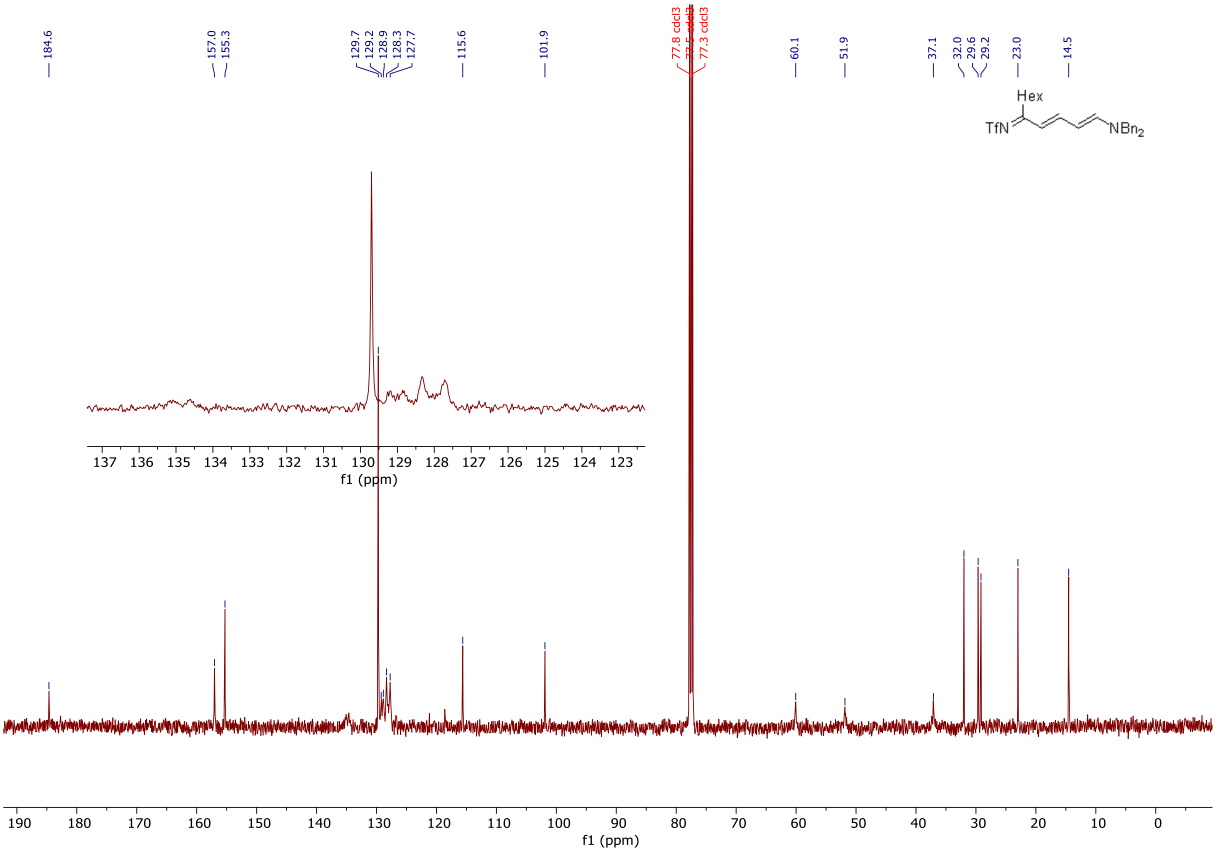

^{1}H NMR spectrum of Zincke imine S31b ($CDCl_3$, 298 K)

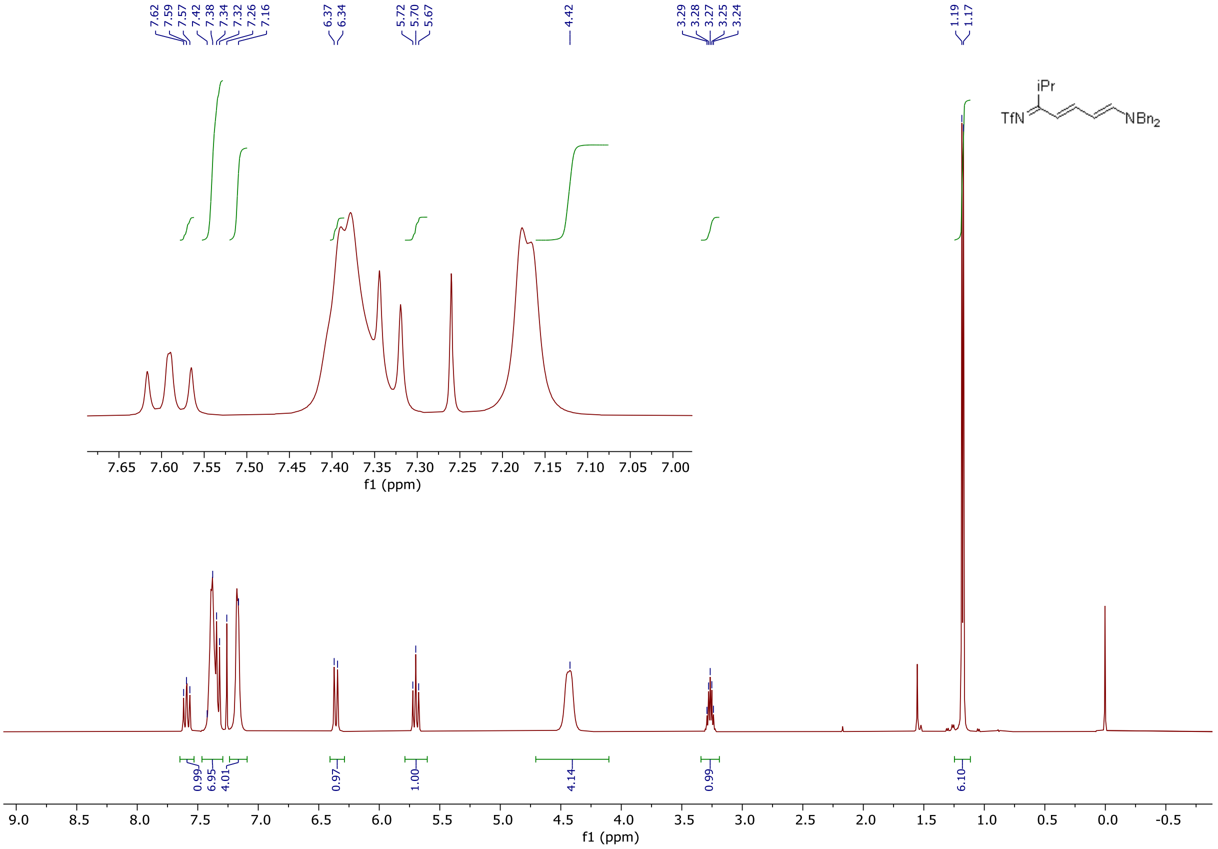

^{13}C NMR spectrum of Zincke imine S31b ($CDCl_3$, 298 K)

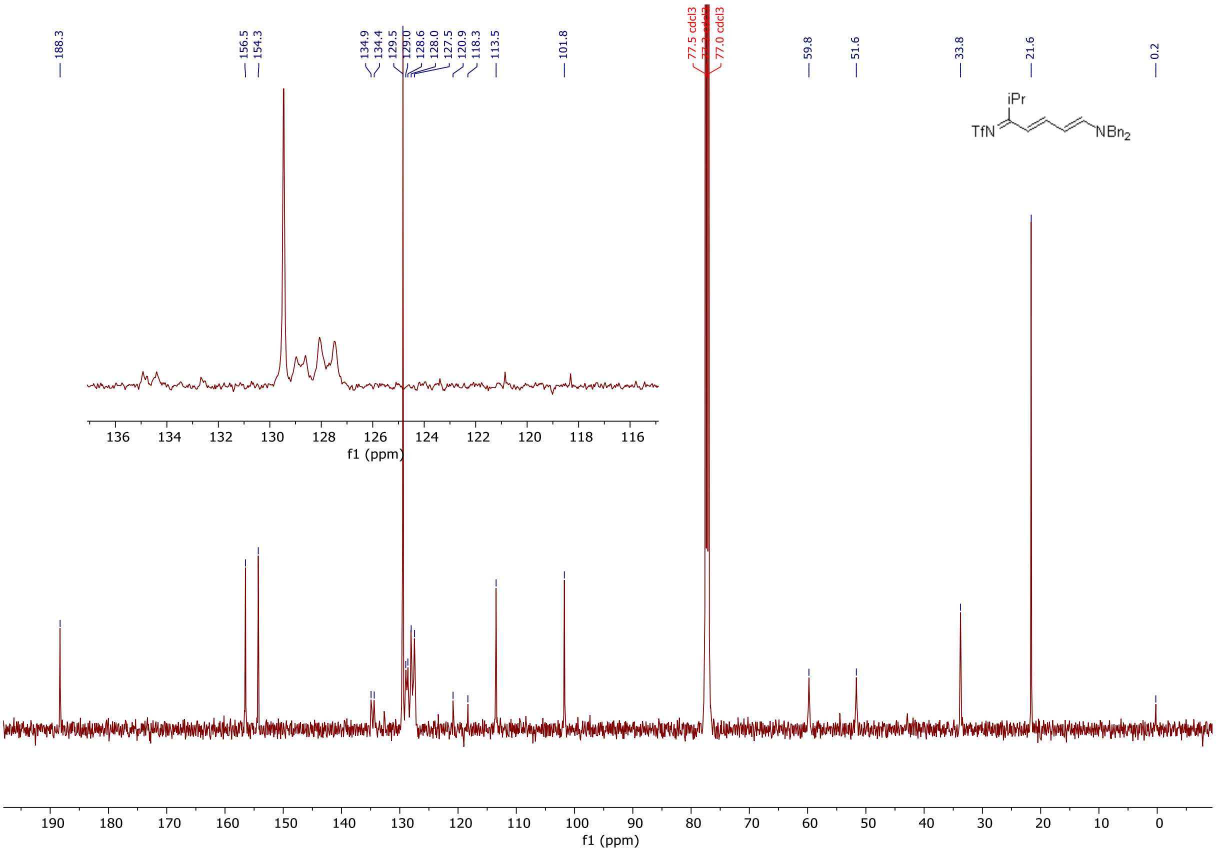

^{1}H NMR spectrum of Zincke imine S32b ($CDCl_3$, 298 K)

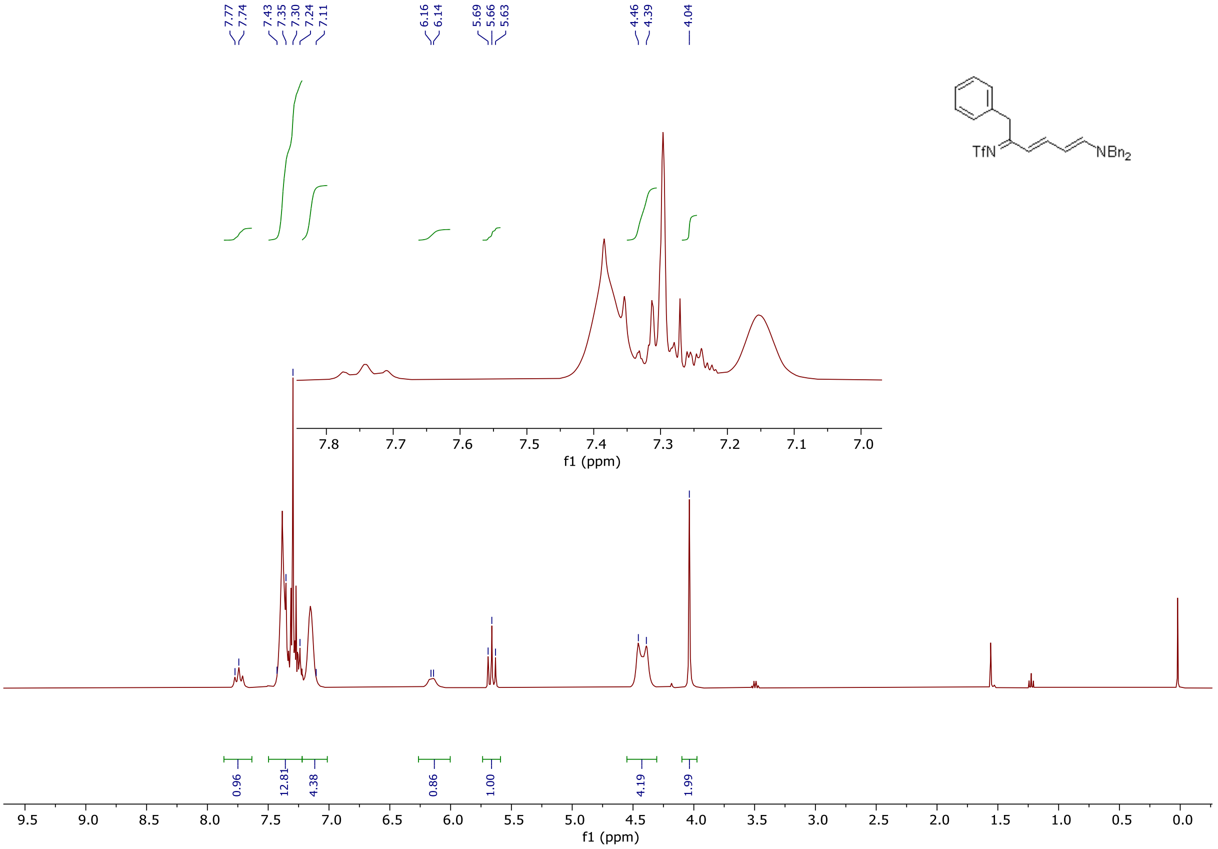

^{13}C NMR spectrum of Zincke imine S32b ($CDCl_3$, 298 K)

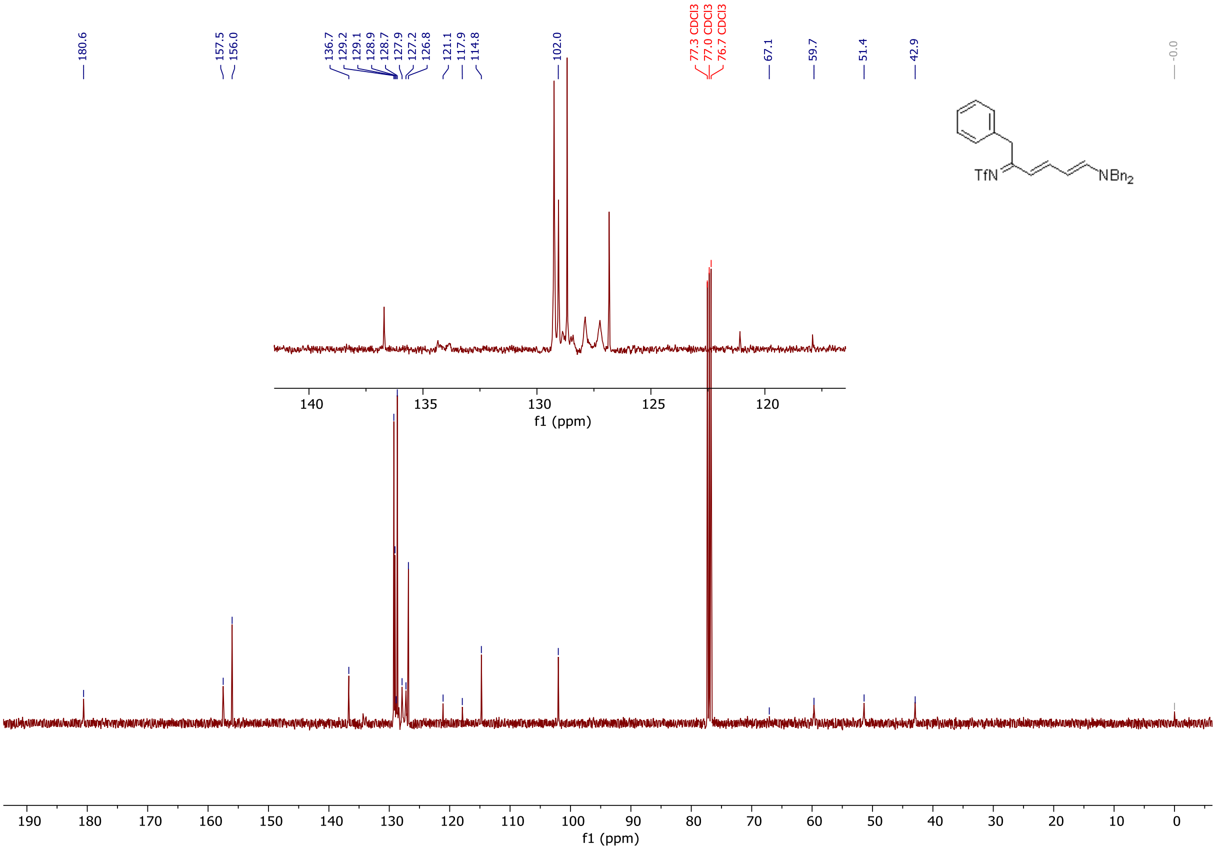

^{1}H NMR spectrum of Zincke imine S33b ($CDCl_3$, 298 K)

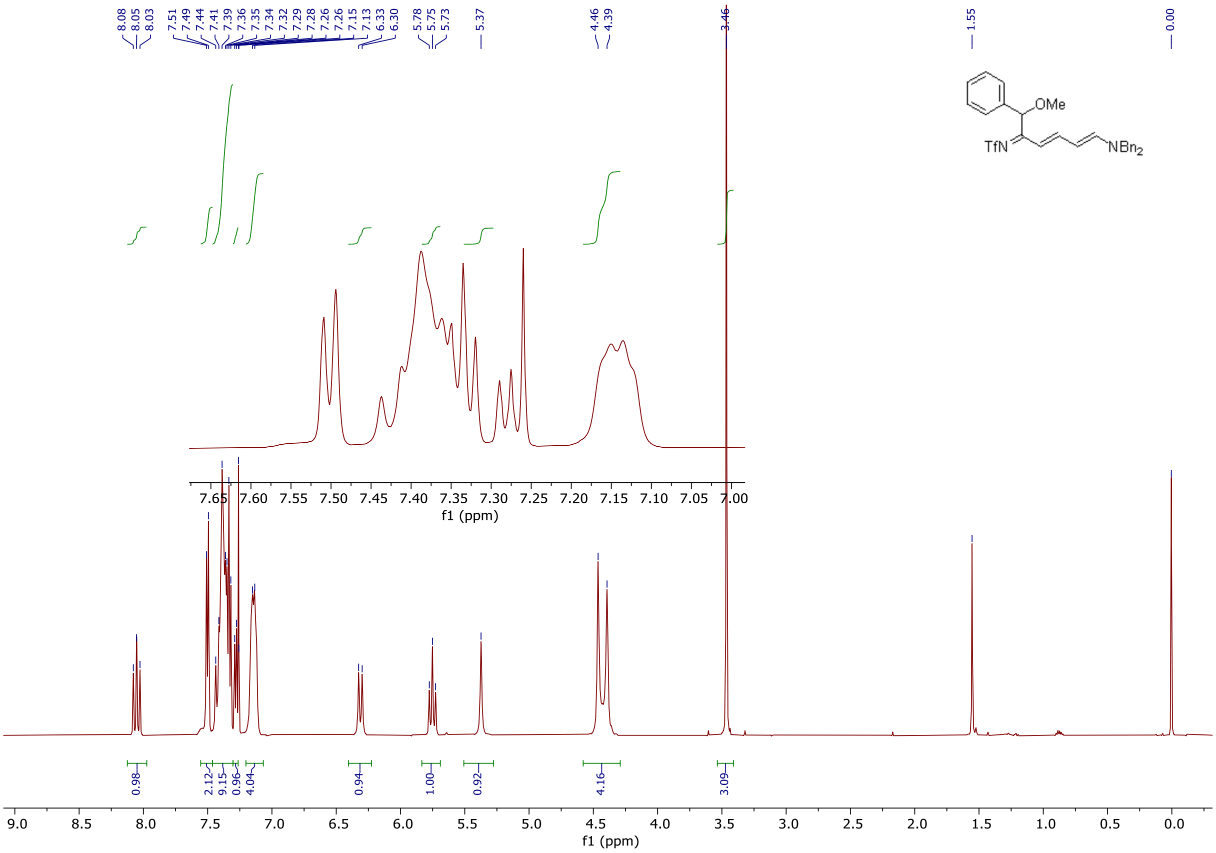

^{13}C NMR spectrum of Zincke imine S33b ($CDCl_3$, 298 K)

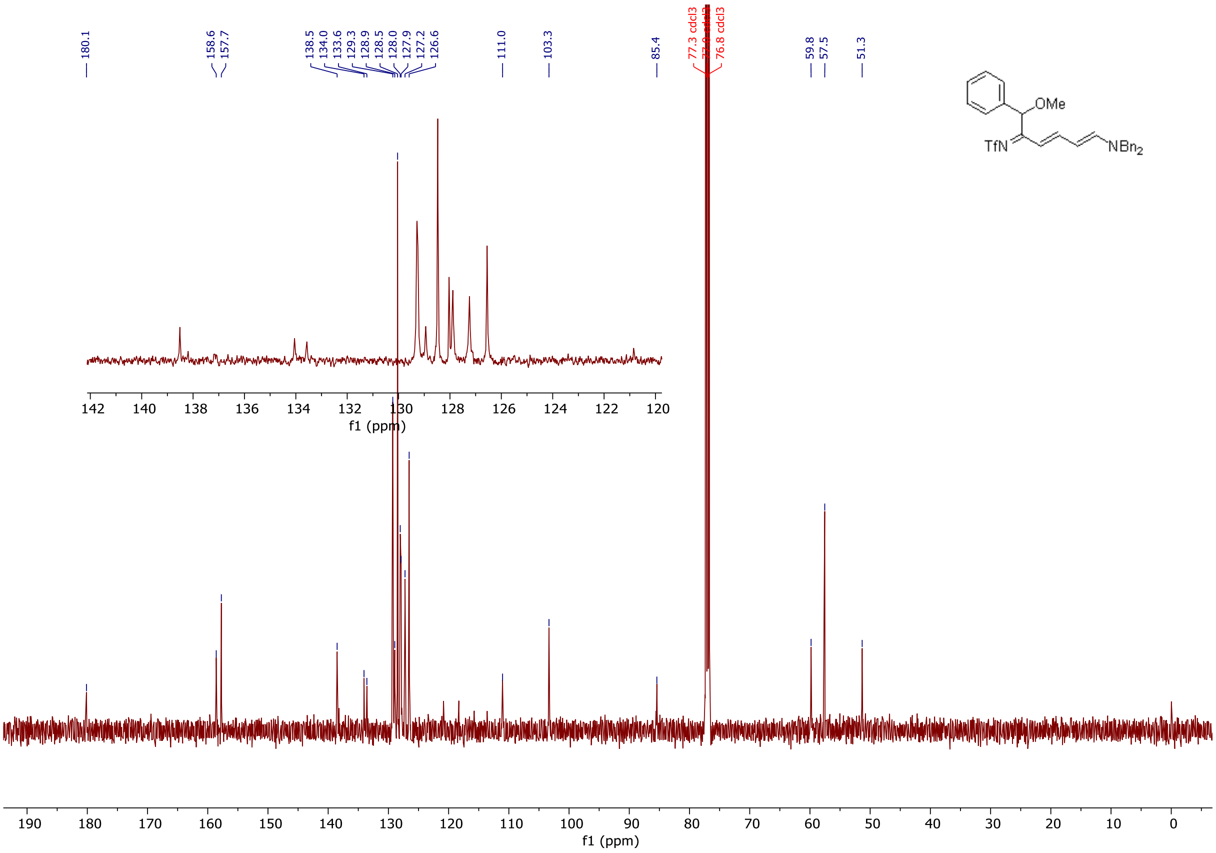

^{1}H NMR spectrum of Zincke imine S34b (CDCl₃, 298 K)

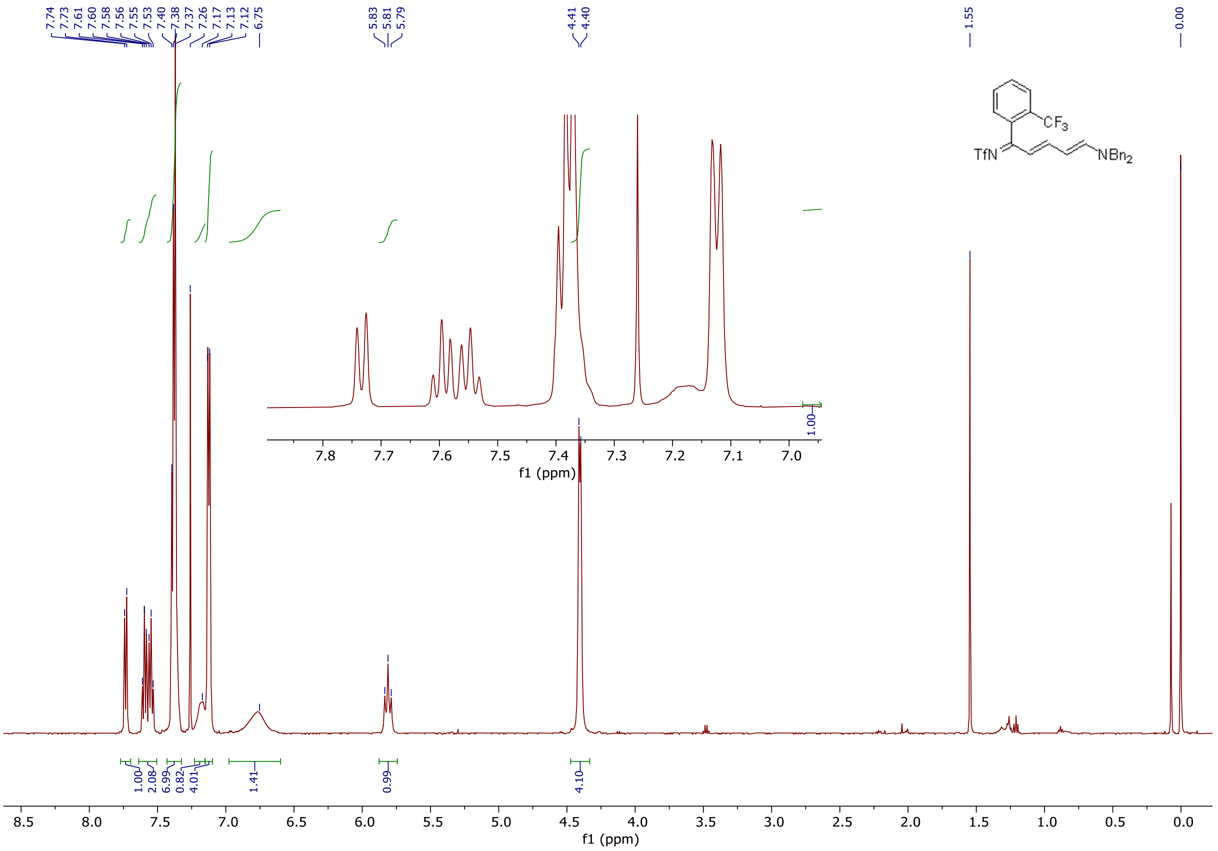

^{13}C NMR spectrum of Zincke imine S34b (CDCl₃, 298 K)

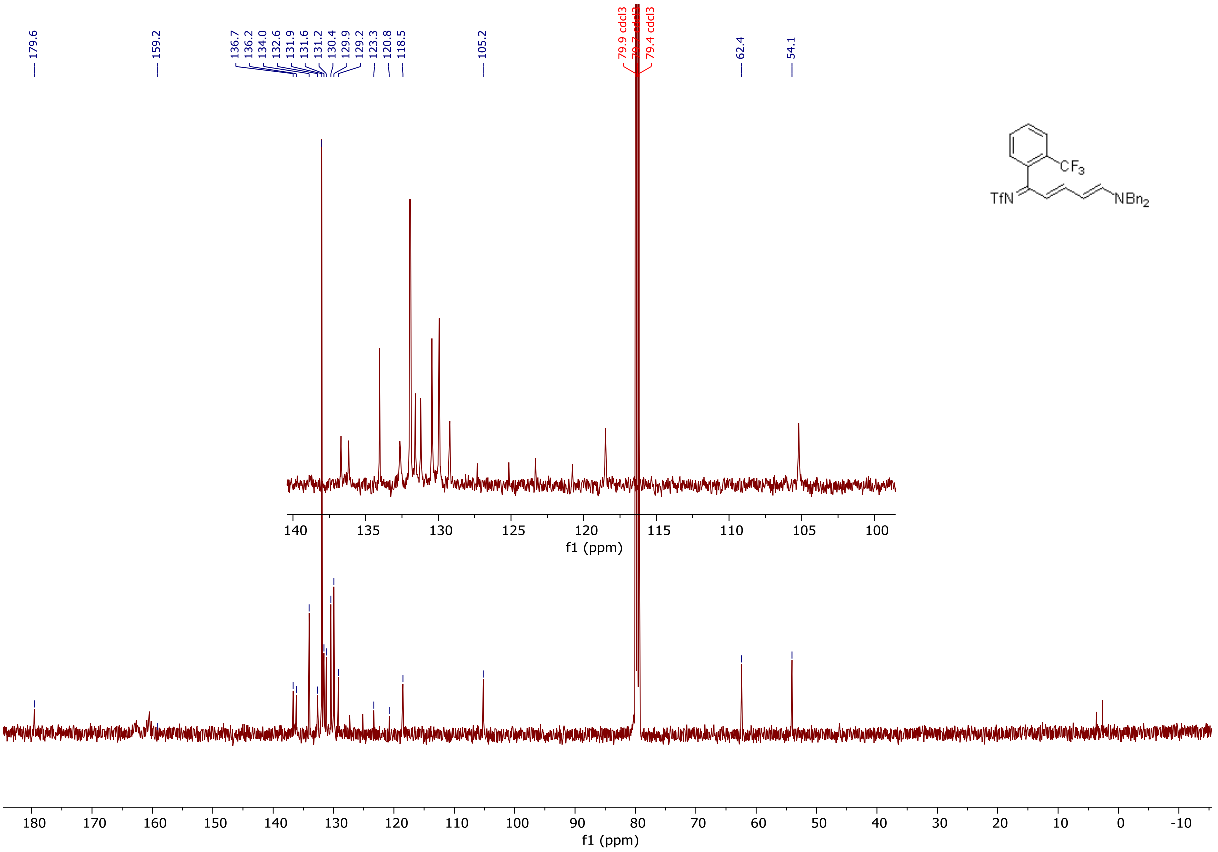

^{1}H NMR spectrum of Zincke imine S35b ($CDCl_3$, 298 K)

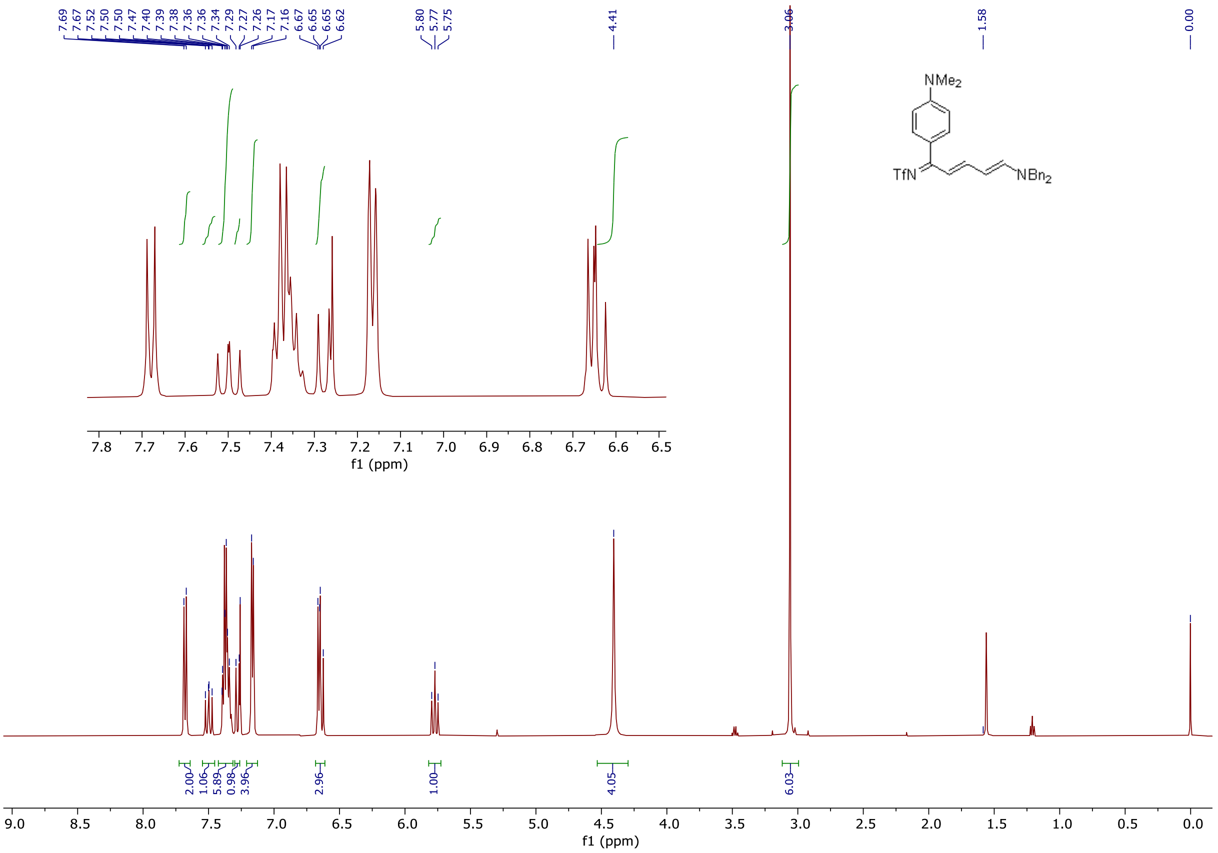

^{13}C NMR spectrum of Zincke imine S35b ($CDCl_3$, 298 K)

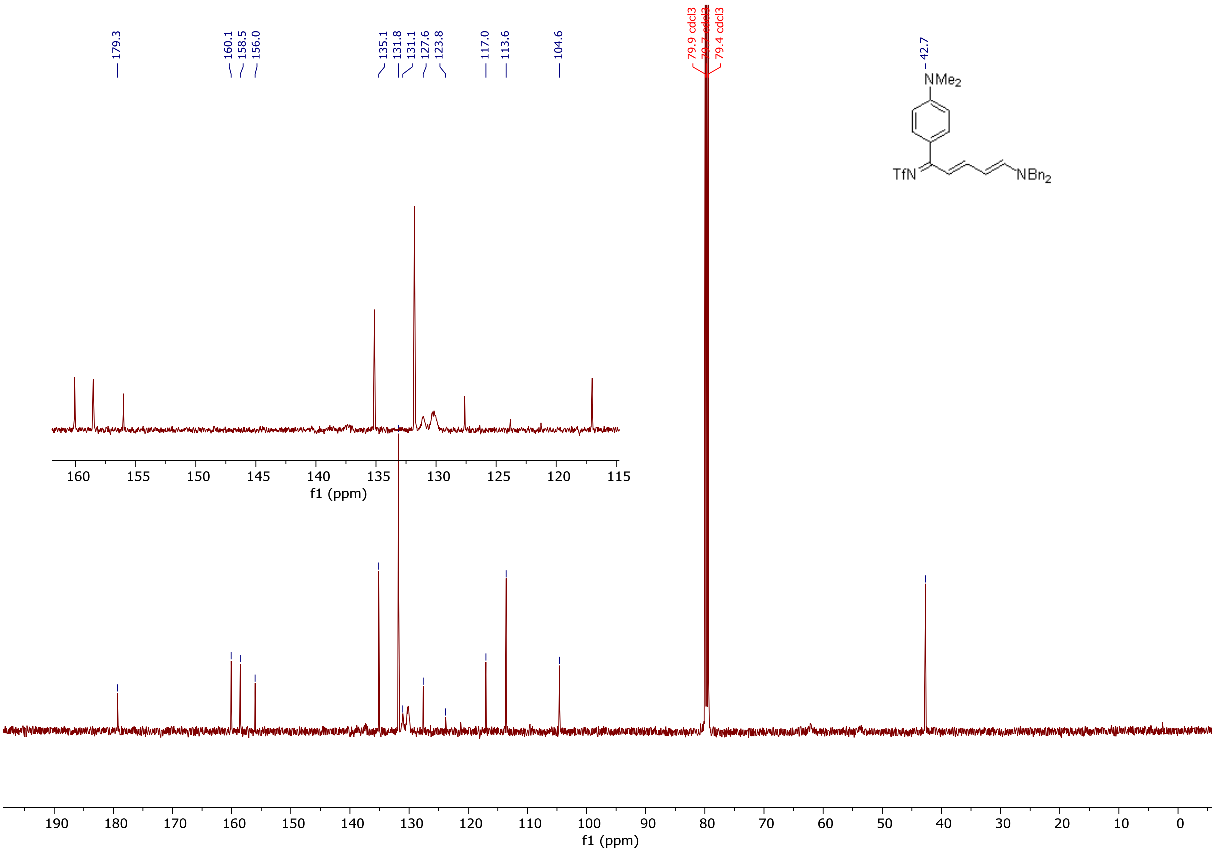

^{1}H NMR spectrum of Zincke imine S36b ($CDCl_3$, 298 K)

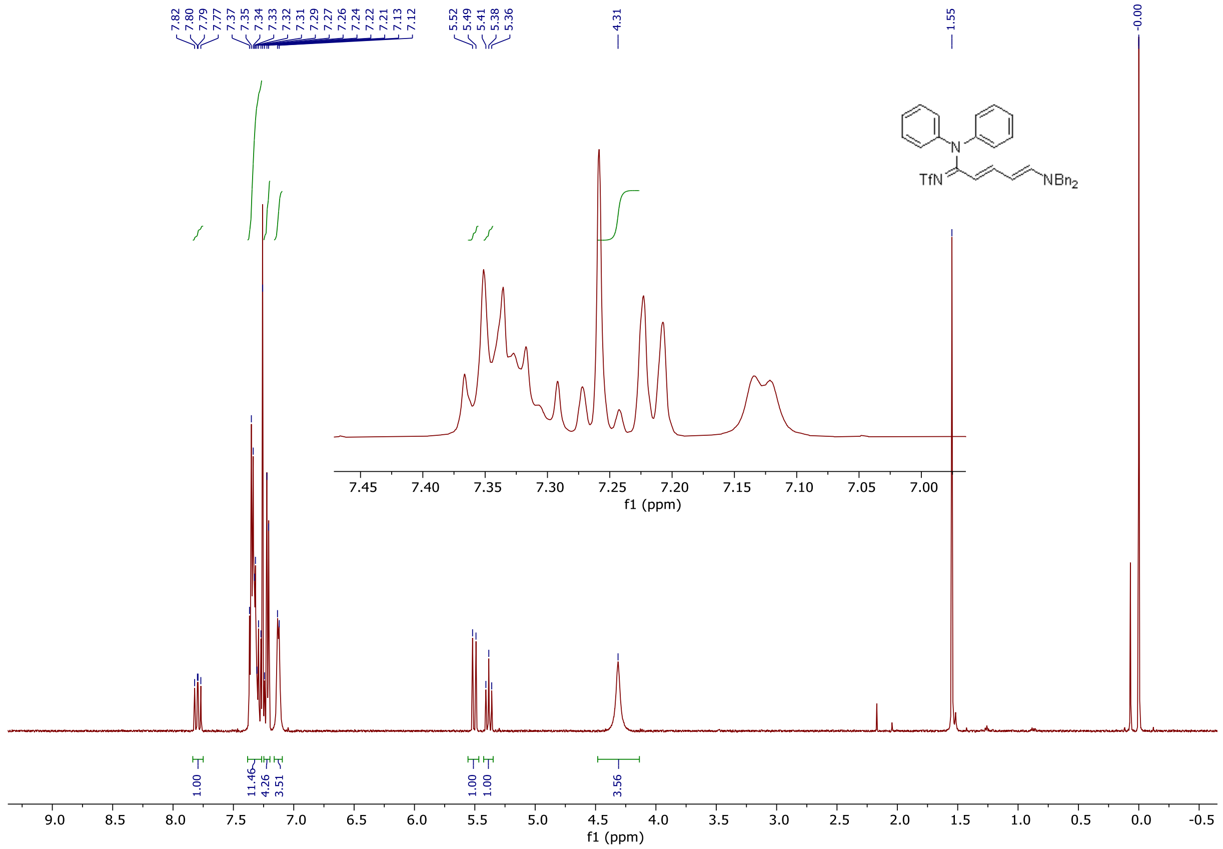

^{13}C NMR spectrum of Zincke imine S36b ($CDCl_3$, 298 K)

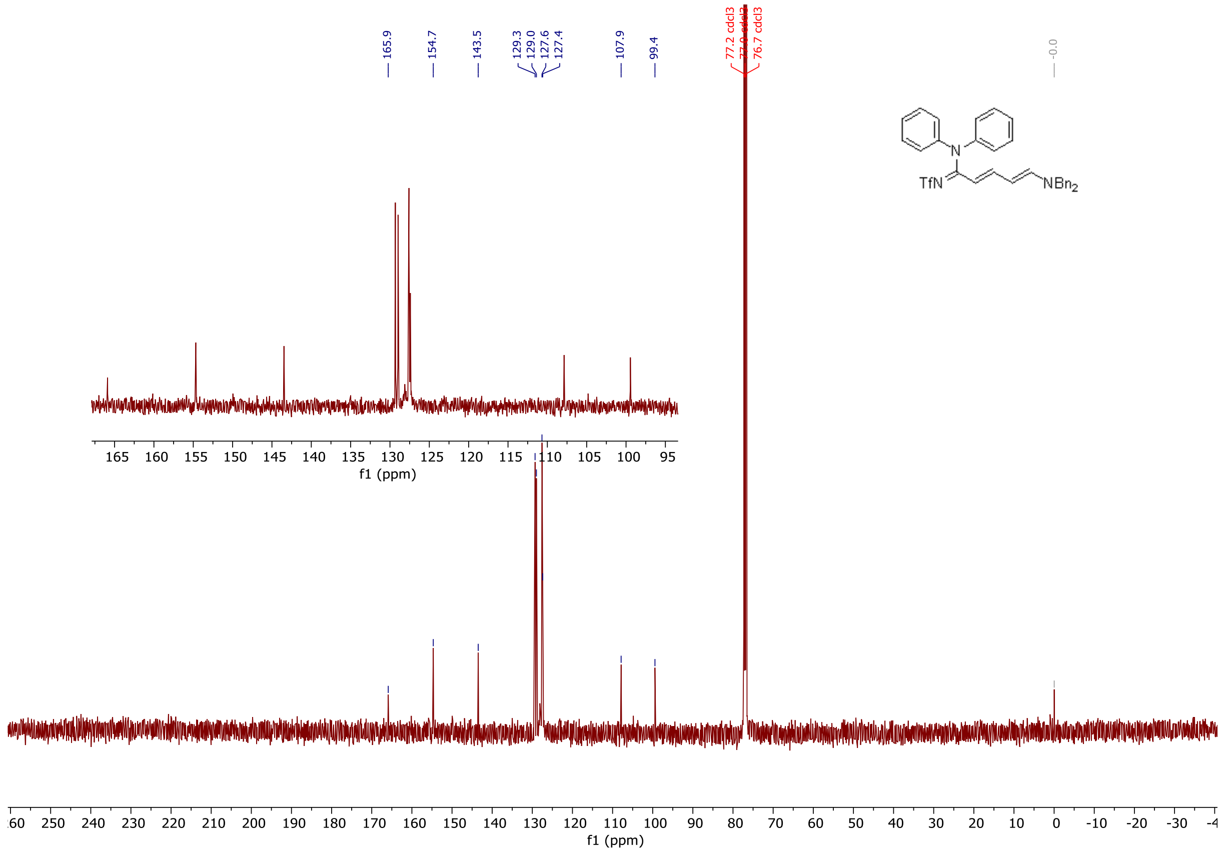

^{1}H NMR spectrum of Zincke imine S37b ($CDCl_3$, 298 K)

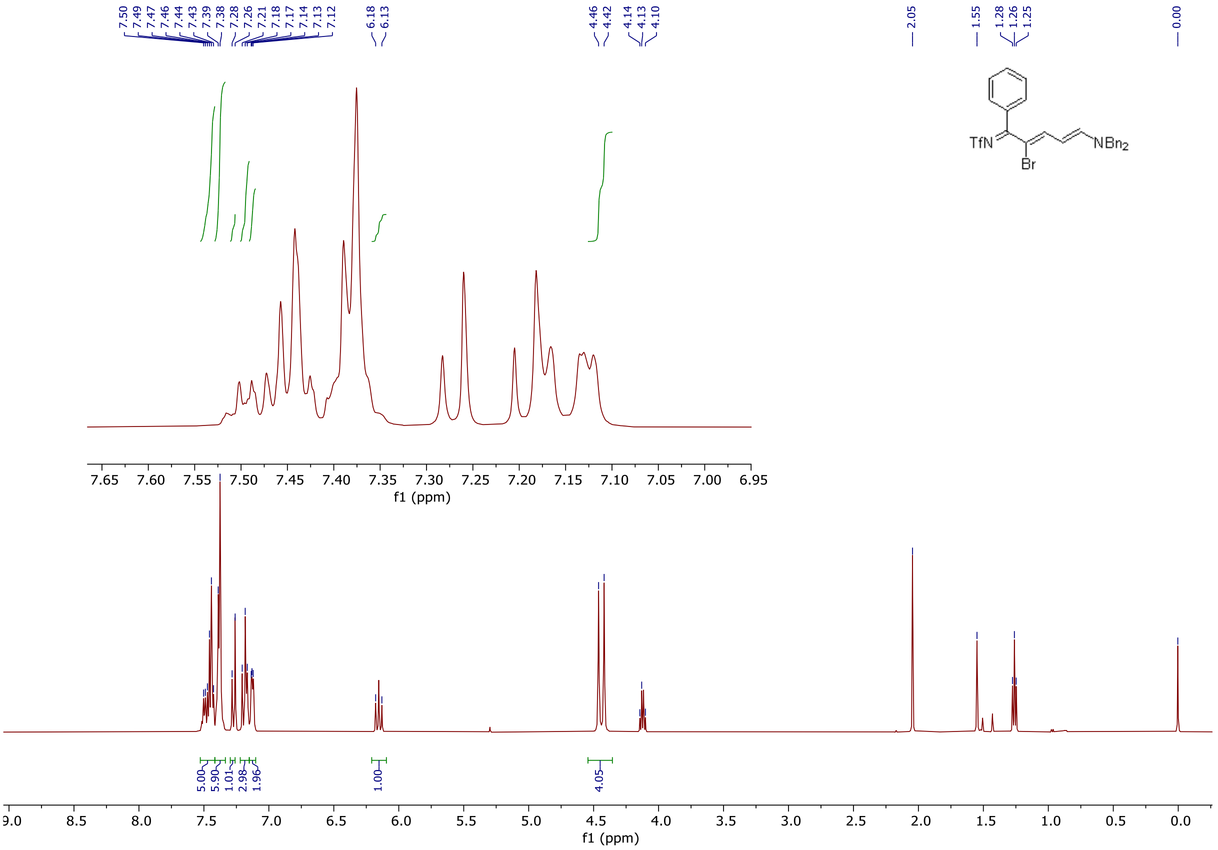

^{13}C NMR spectrum of Zincke imine S37b ($CDCl_3$, 298 K)

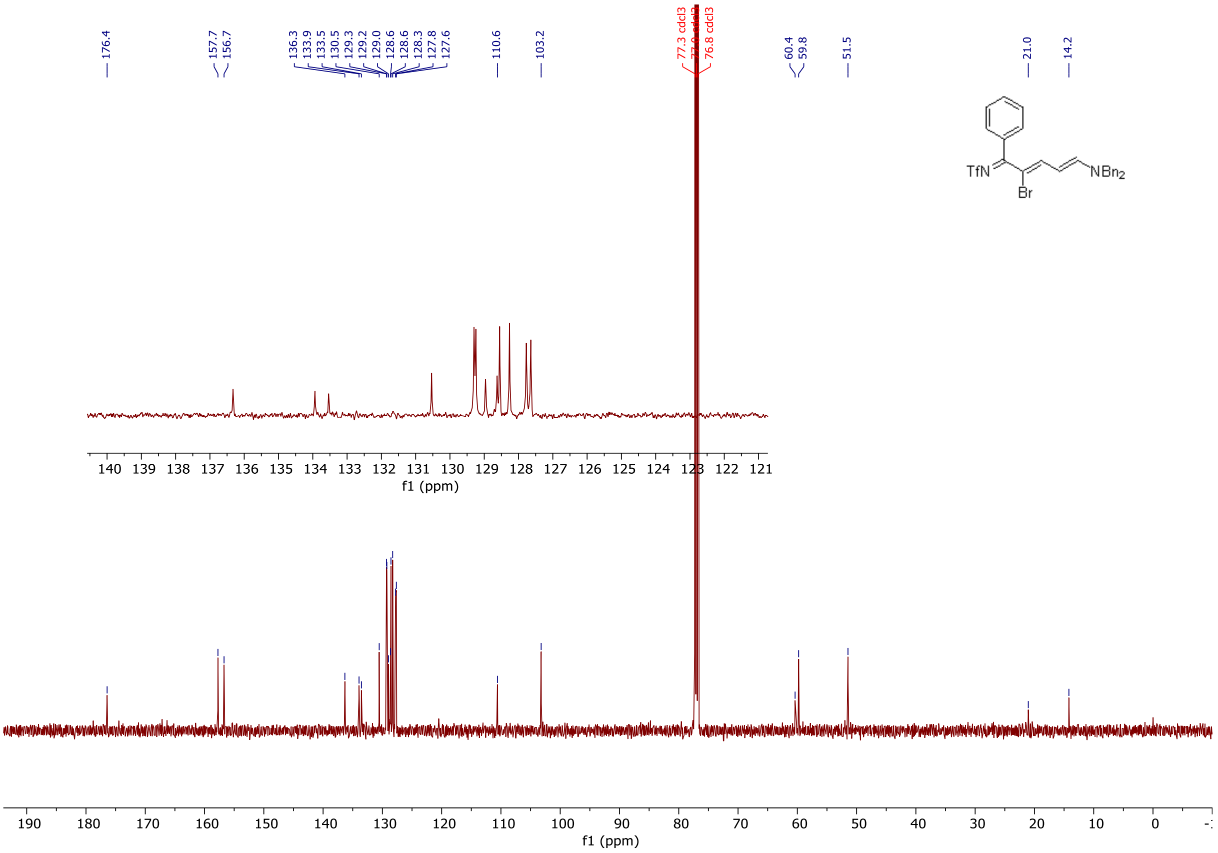

^{1}H NMR spectrum of Zincke imine S38b ($CDCl_3$, 298 K)

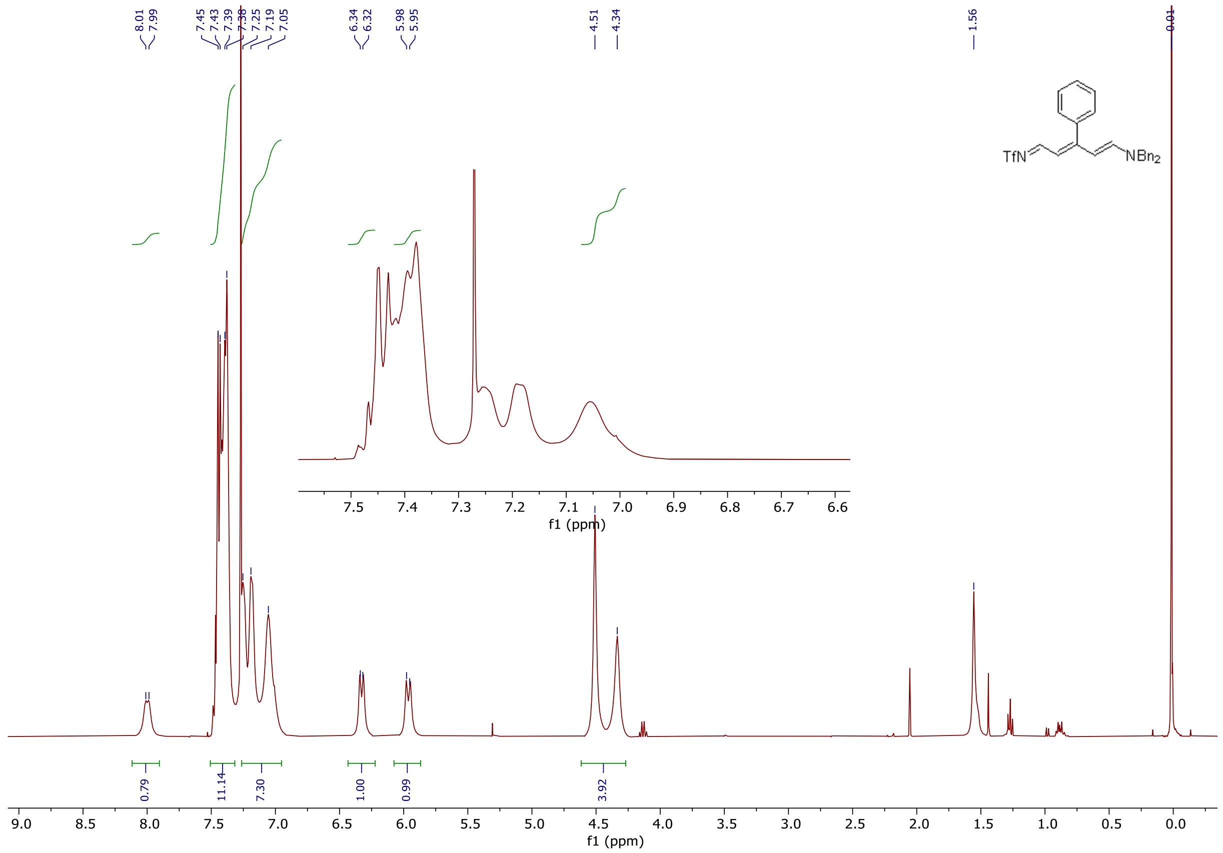

^{1}H NMR spectrum of Zincke imine S39b ($CDCl_3$, 298 K)

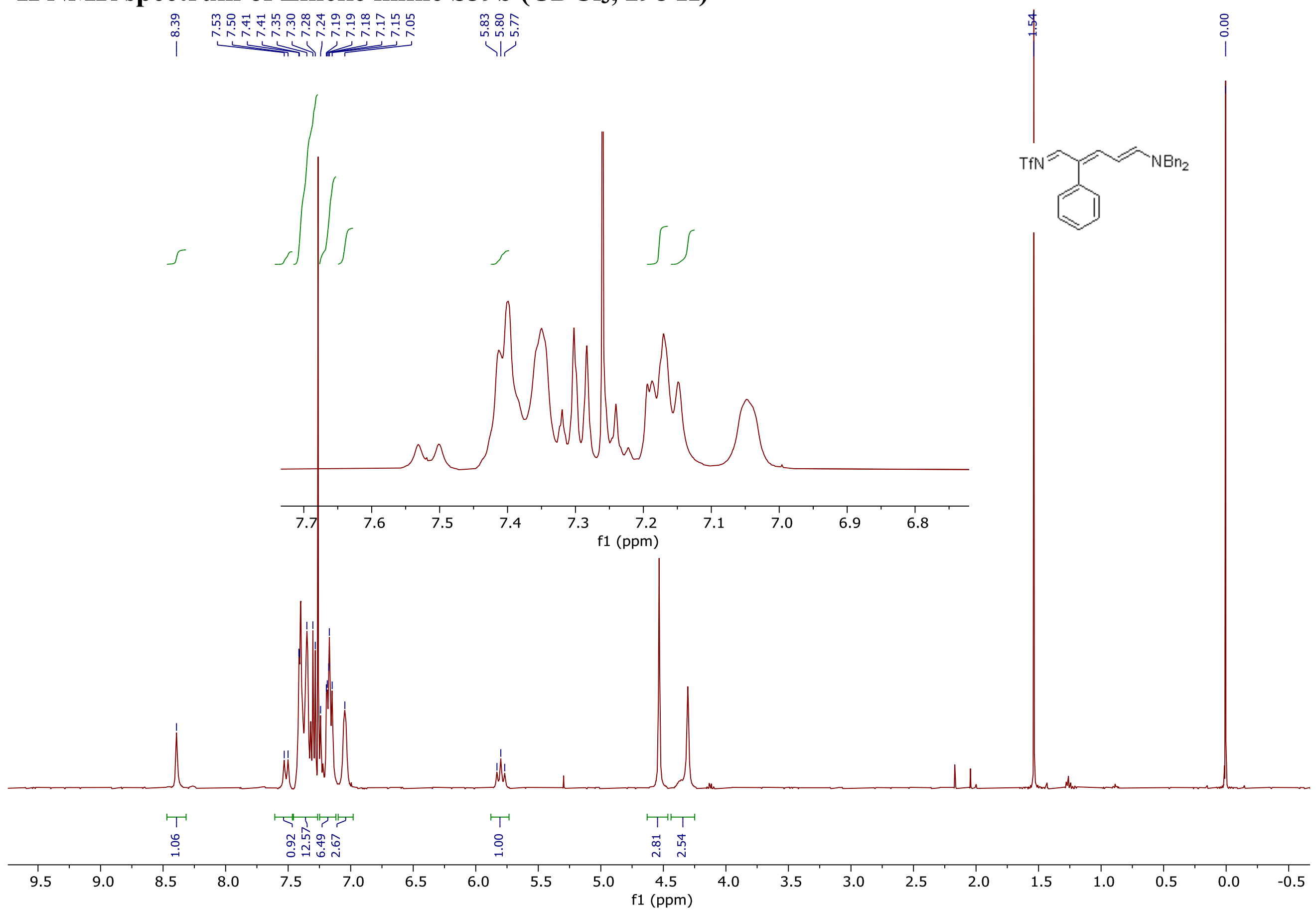

^{1}H NMR spectrum of Zincke imine S40b ($CDCl_3$, 298 K)

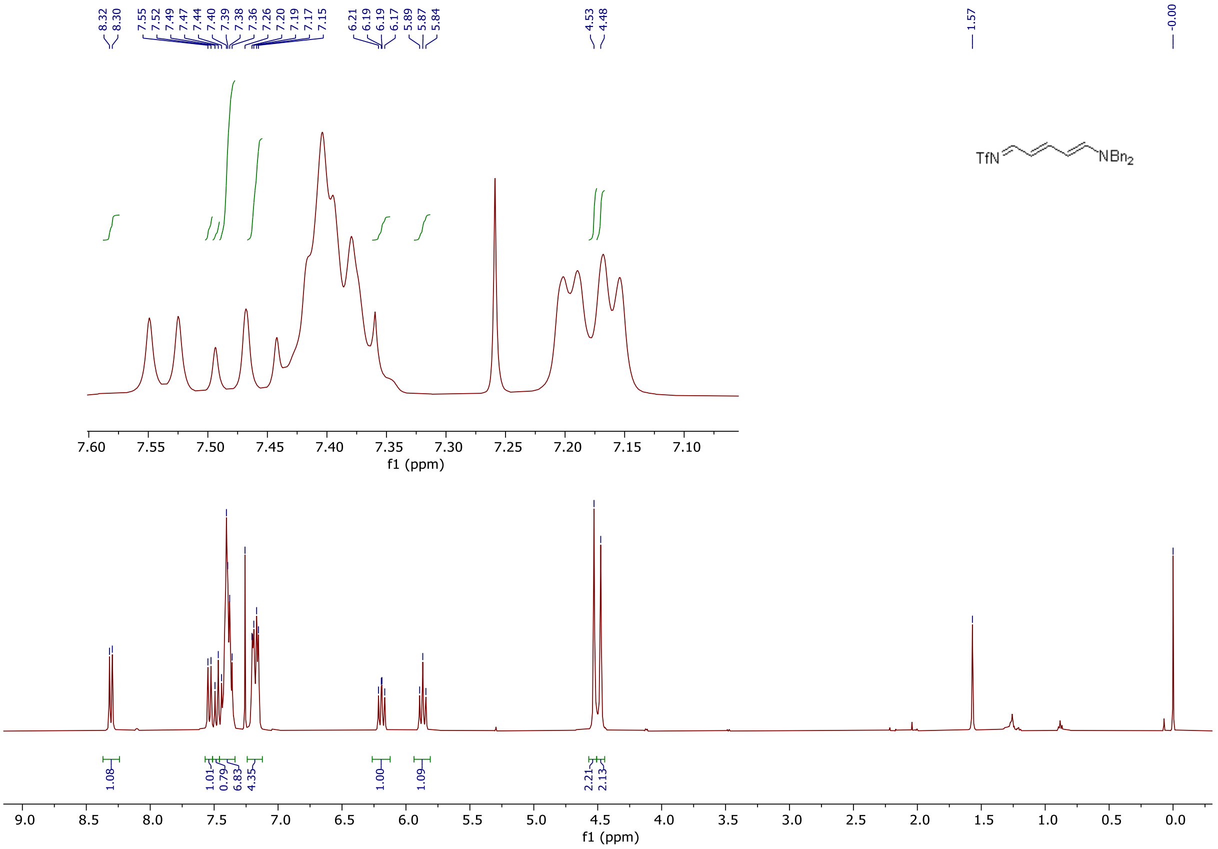

^{13}C NMR spectrum of Zincke imine S40b ($CDCl_3$, 298 K)

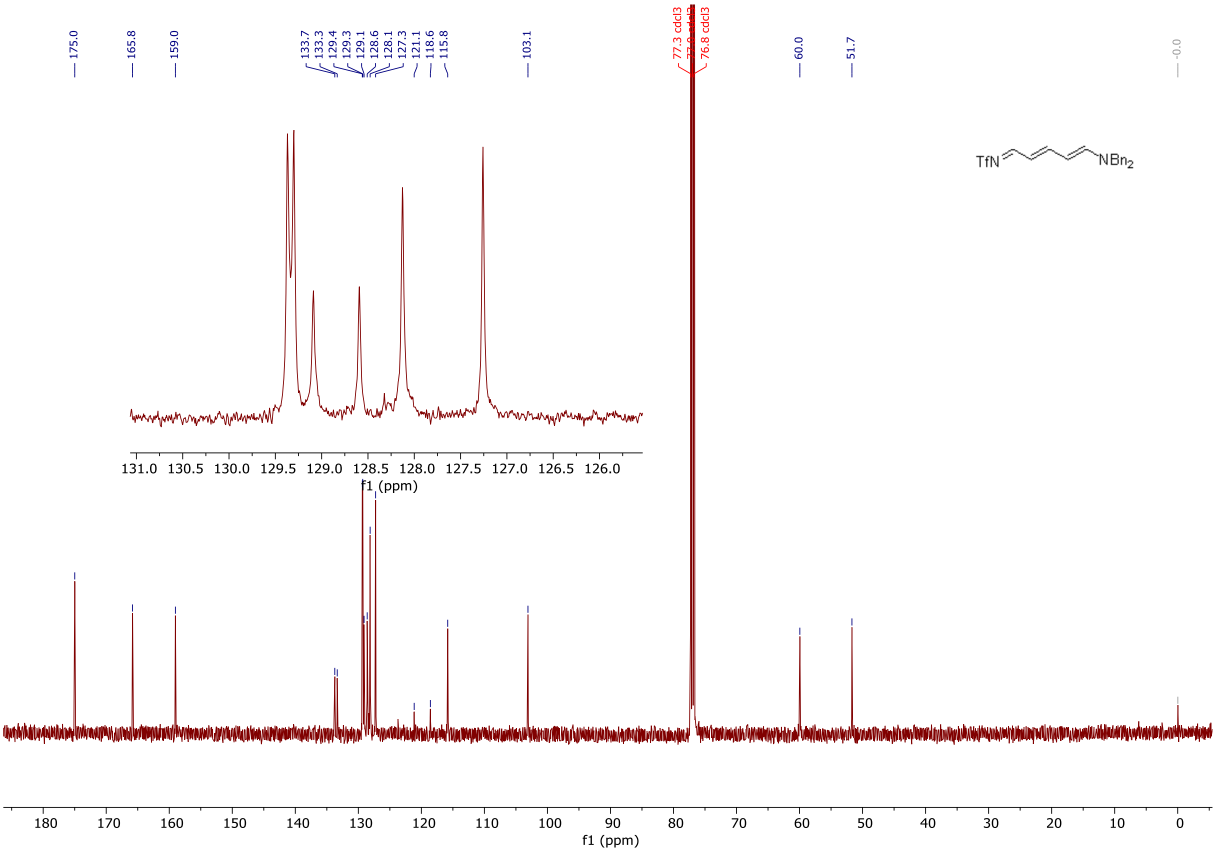

^{1}H NMR spectrum of Zincke imine S41b ($CDCl_3$, 298 K)

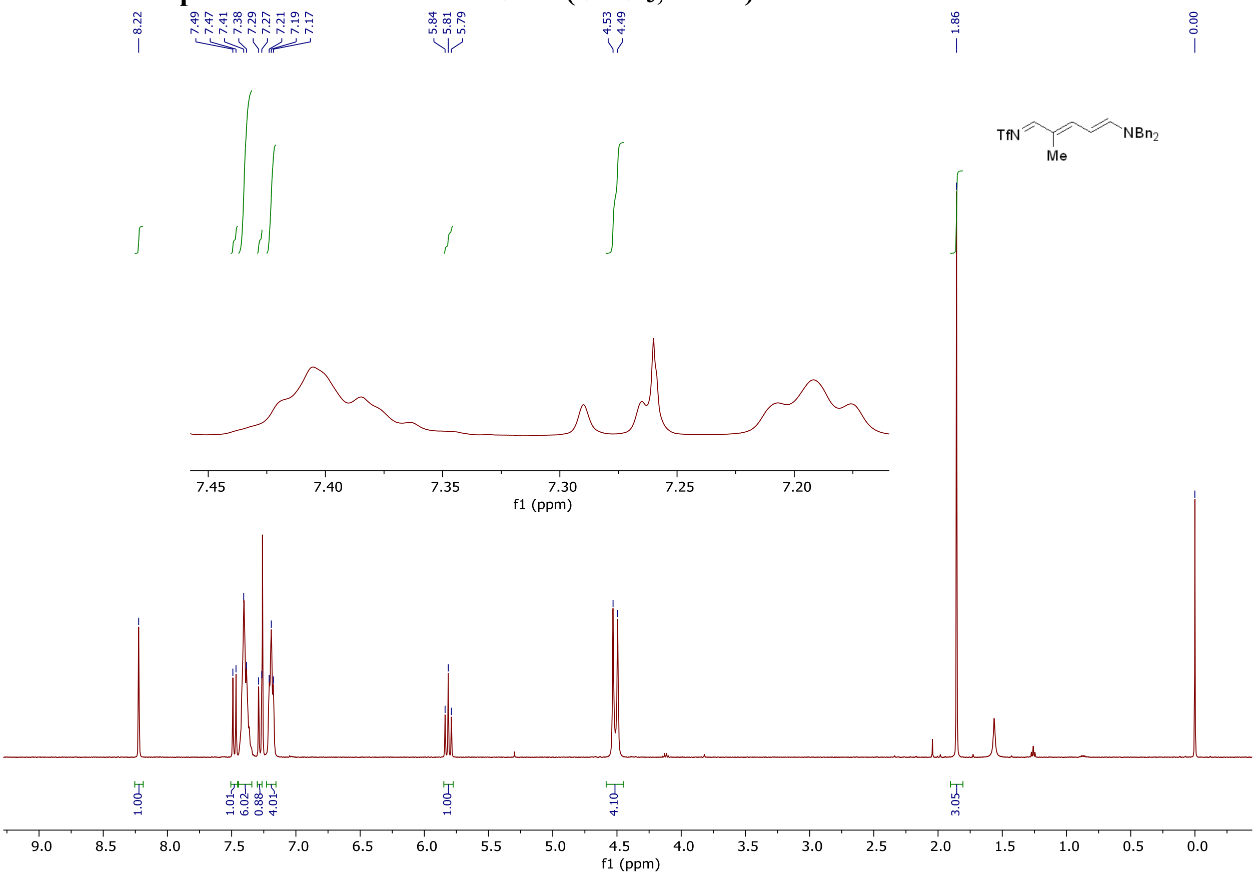

^{13}C NMR spectrum of Zincke imine S41b ($CDCl_3$, 298 K)

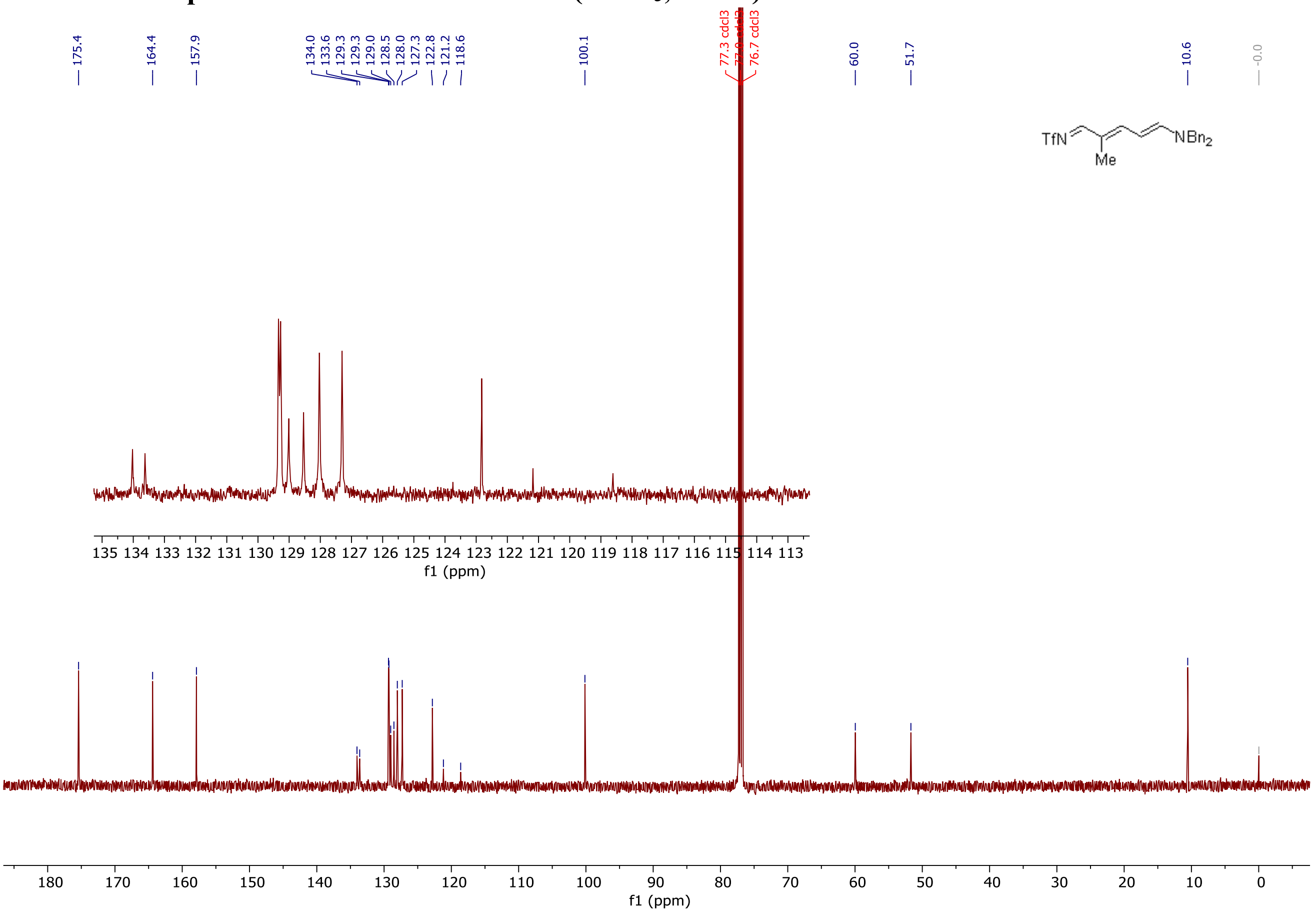

^{1}H NMR spectrum of Zincke imine S42b ($CDCl_3$, 298 K)

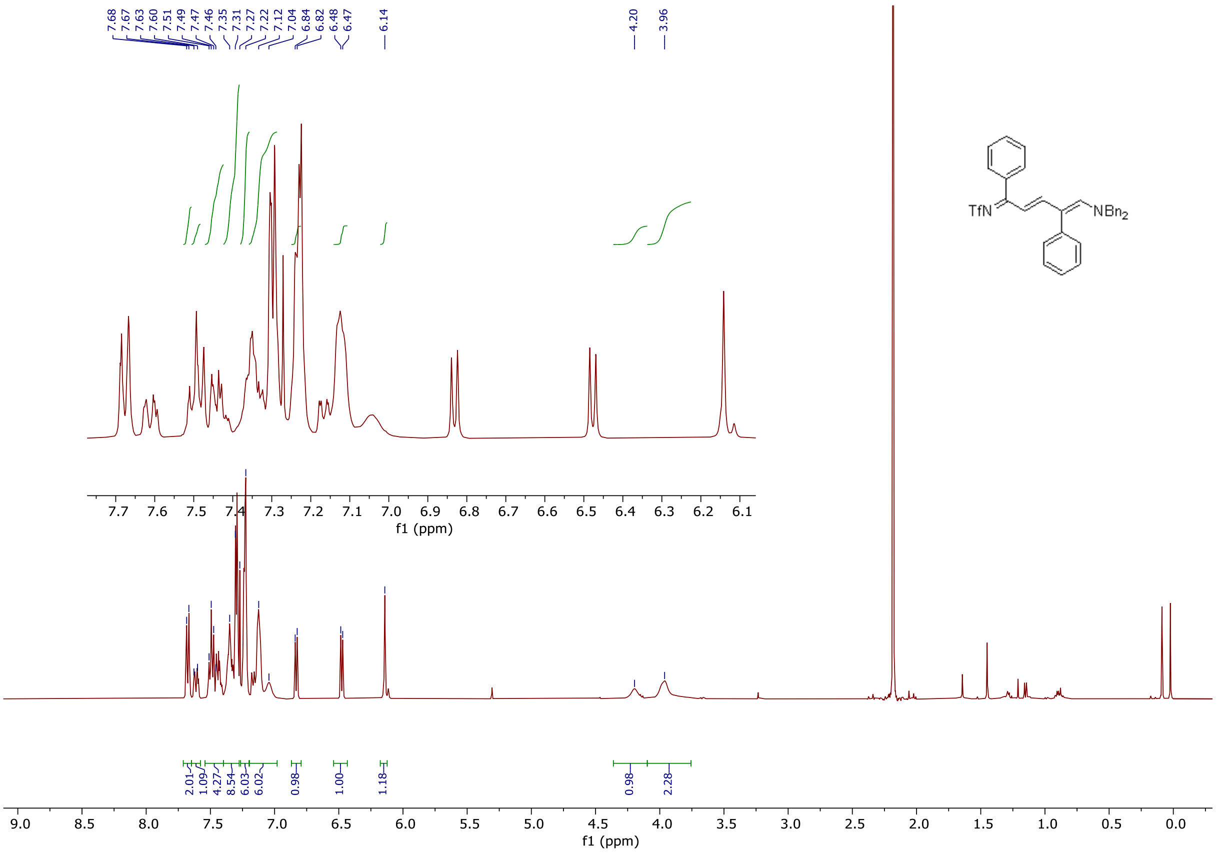

^{13}C NMR spectrum of Zincke imine S42b ($CDCl_3$, 298 K)

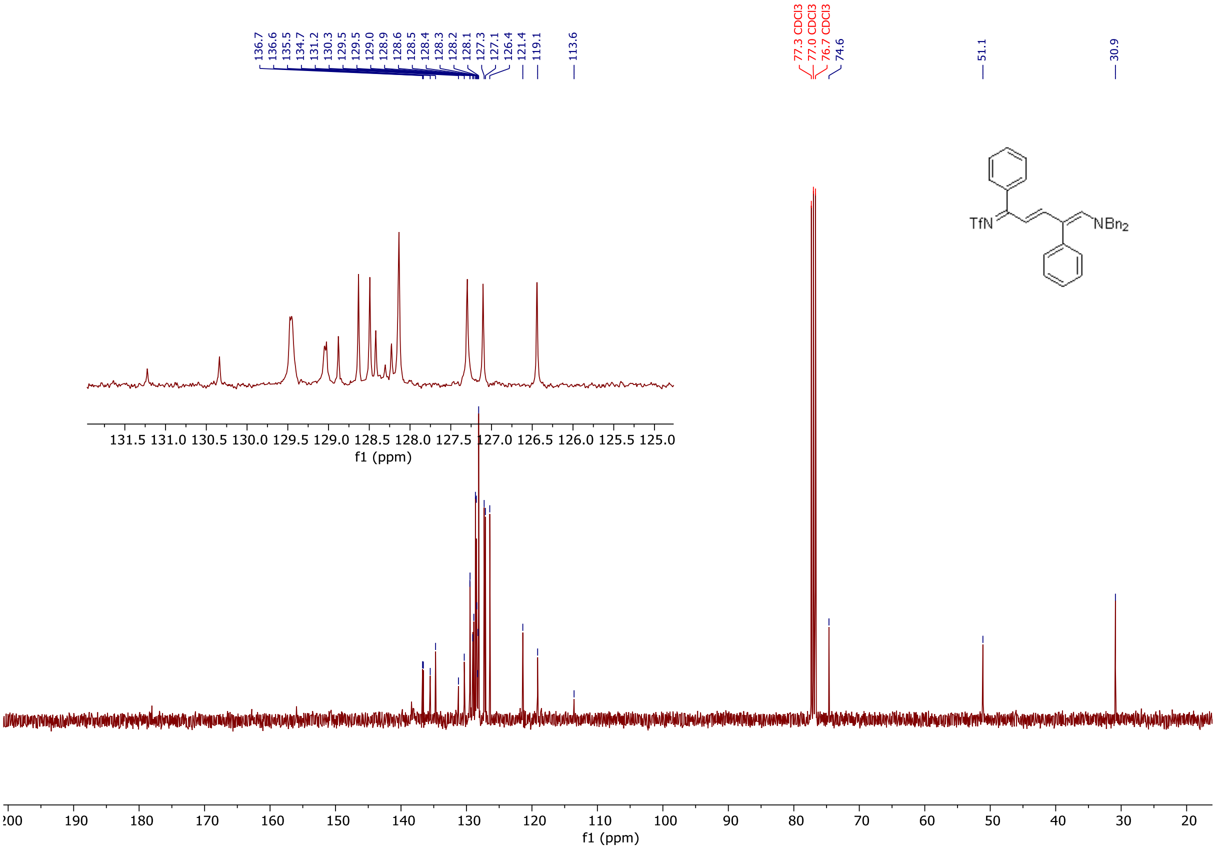

^{1}H NMR spectrum of Zincke imine S43b ($CDCl_3$, 298 K)

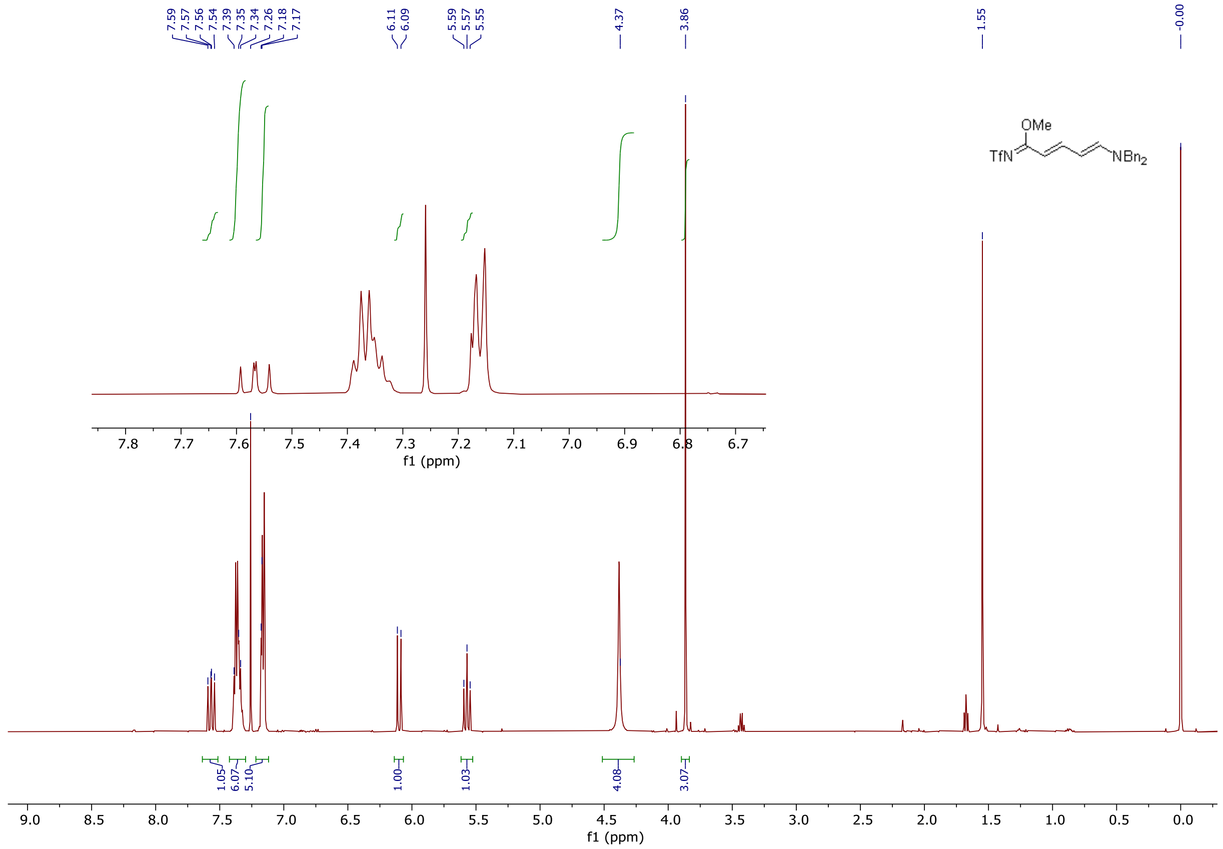

^{13}C NMR spectrum of Zincke imine S43b ($CDCl_3$, 298 K)

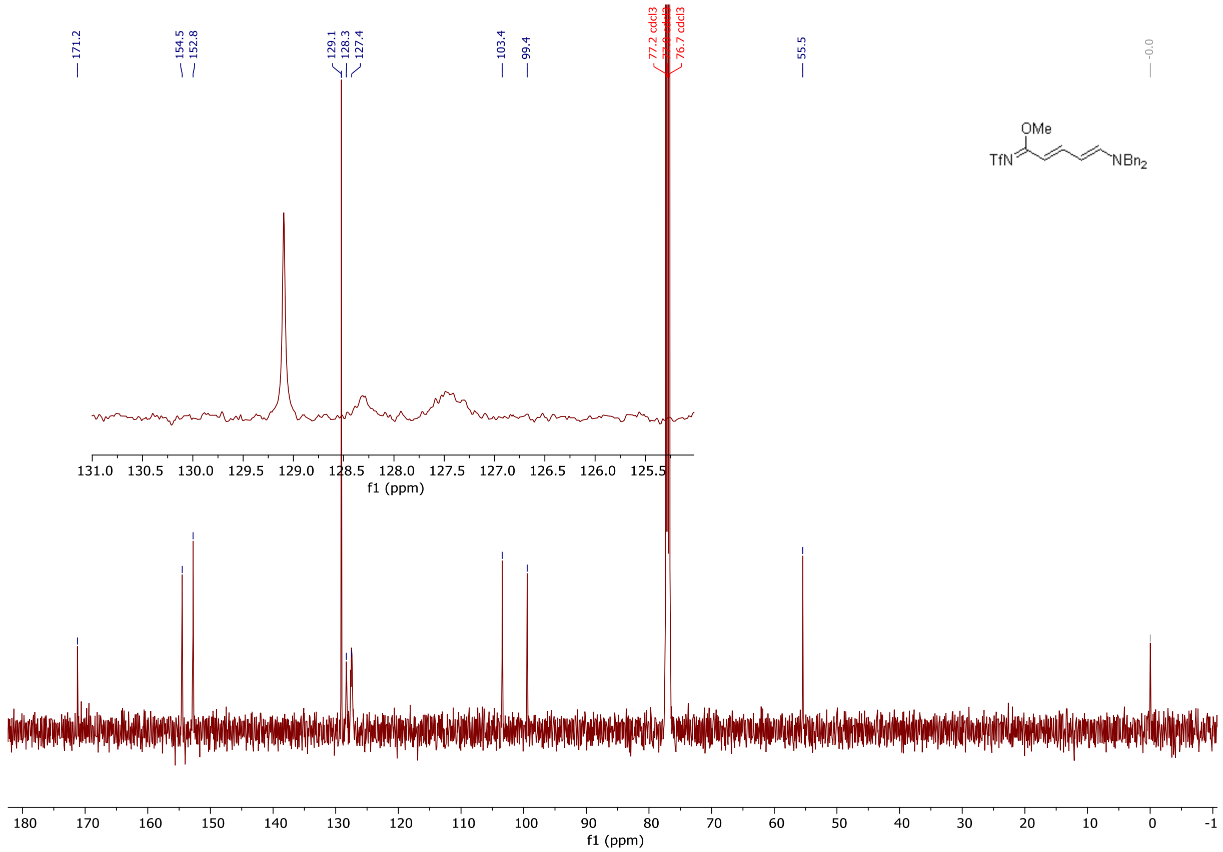

^{1}H NMR spectrum of Zincke imine S44b ($CDCl_3$, 298 K)

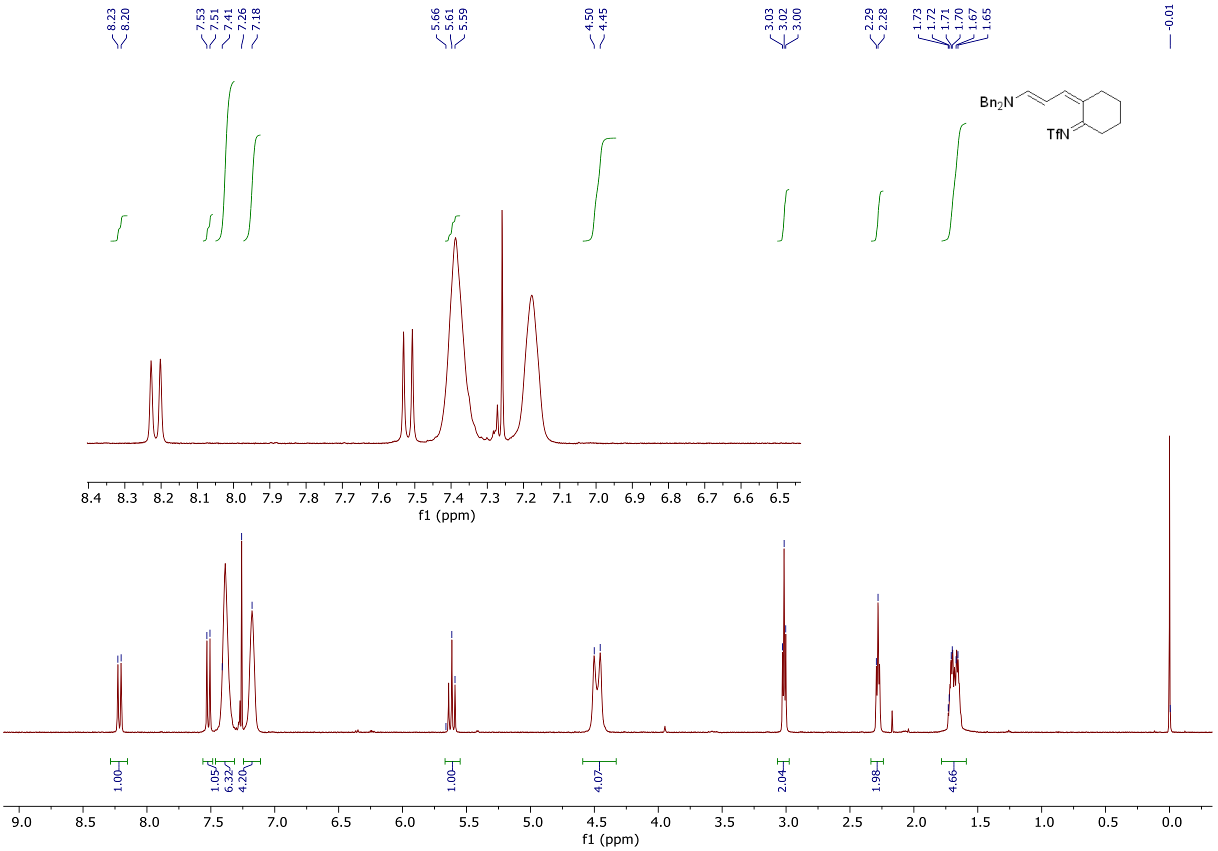

^{13}C NMR spectrum of Zincke imine S44b ($CDCl_3$, 298 K)

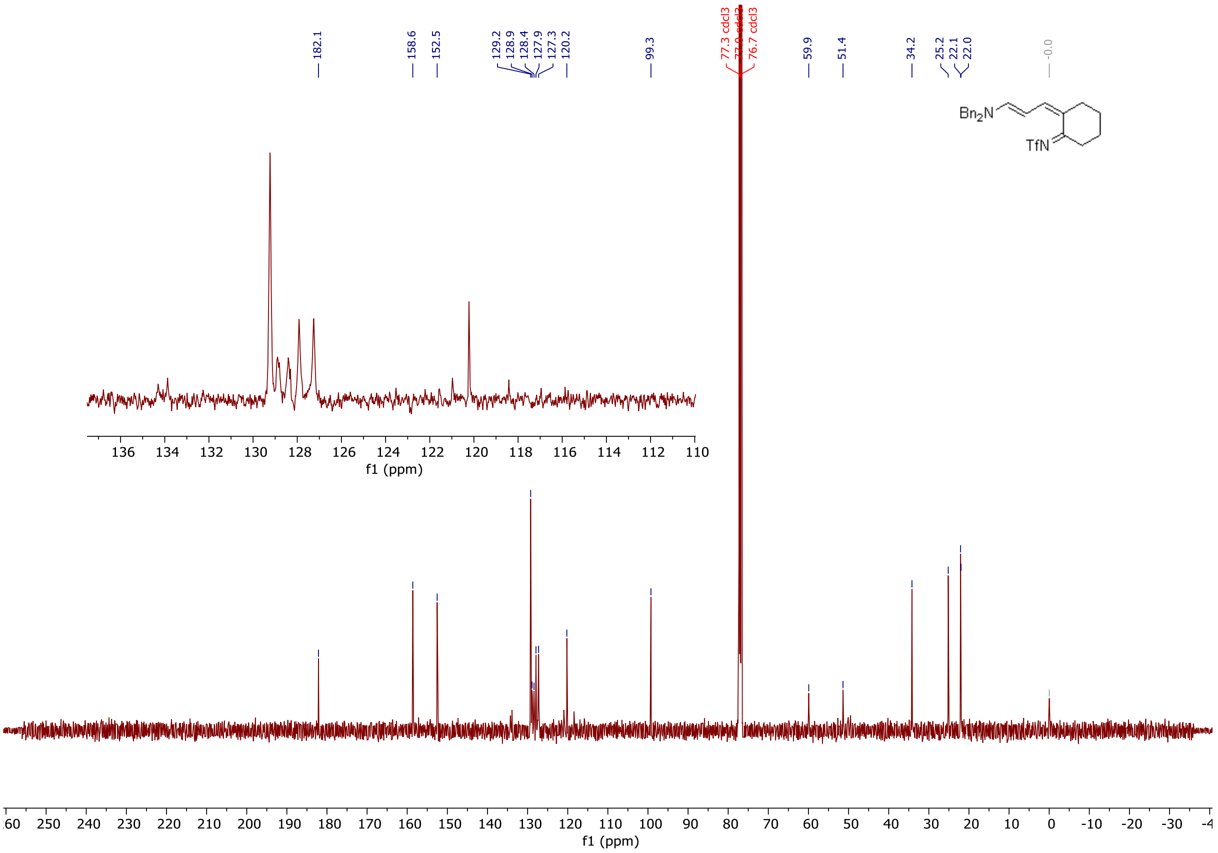

^{1}H NMR spectrum of Zincke imine S45b ($CDCl_3$, 298 K)

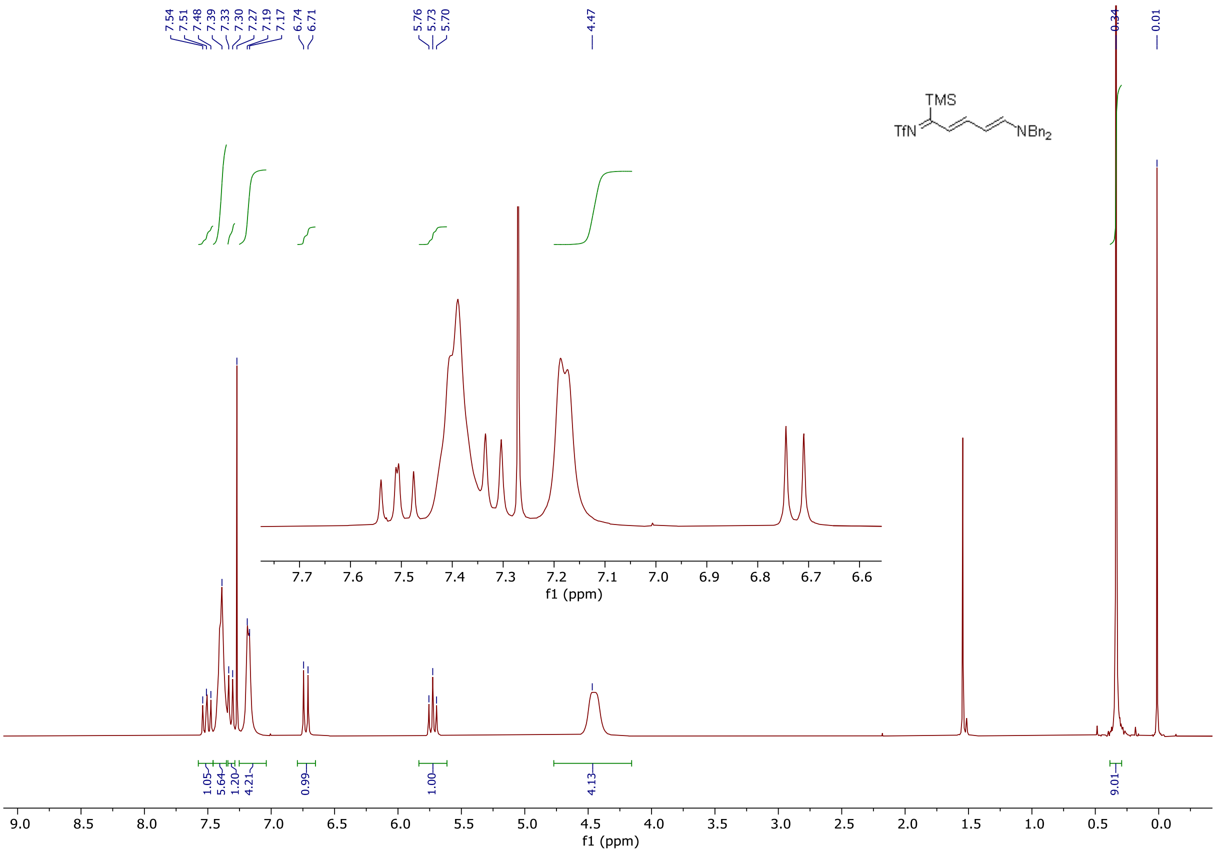

^{13}C NMR spectrum of Zincke imine S45b ($CDCl_3$, 298 K)

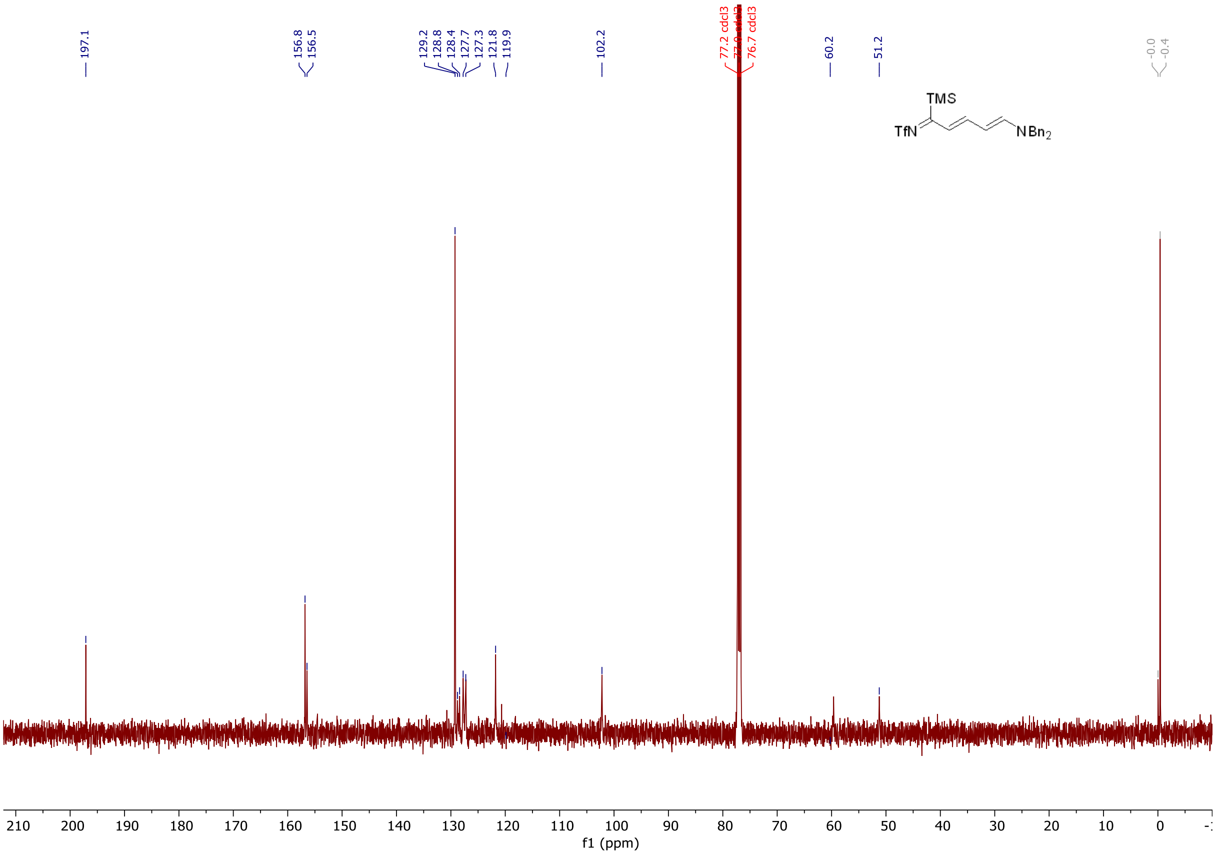

^{1}H NMR spectrum of Zincke imine S46b ($CDCl_3$, 298 K)

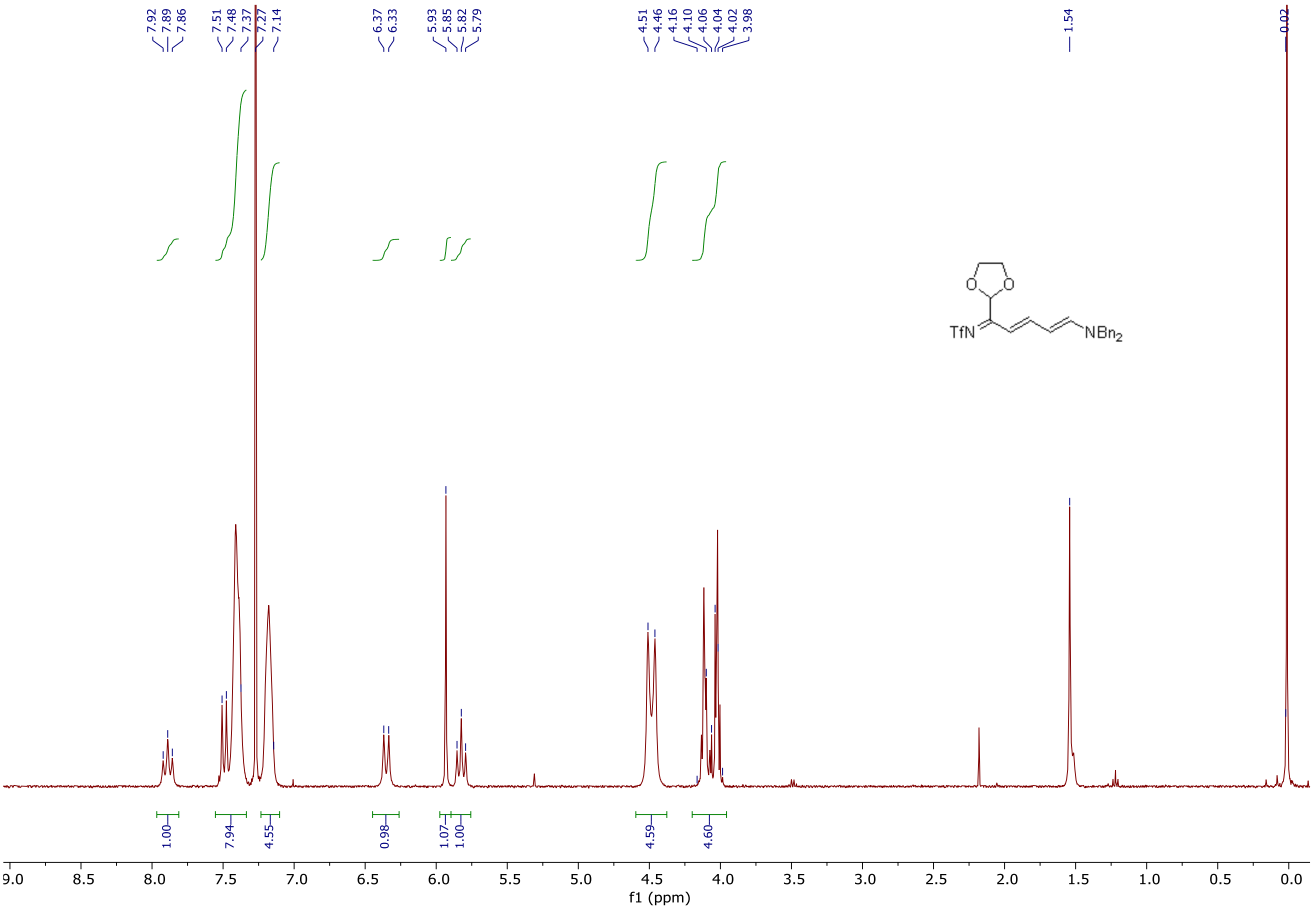

^{1}H NMR spectrum of Zincke imine S47b ($CDCl_3$, 298 K)

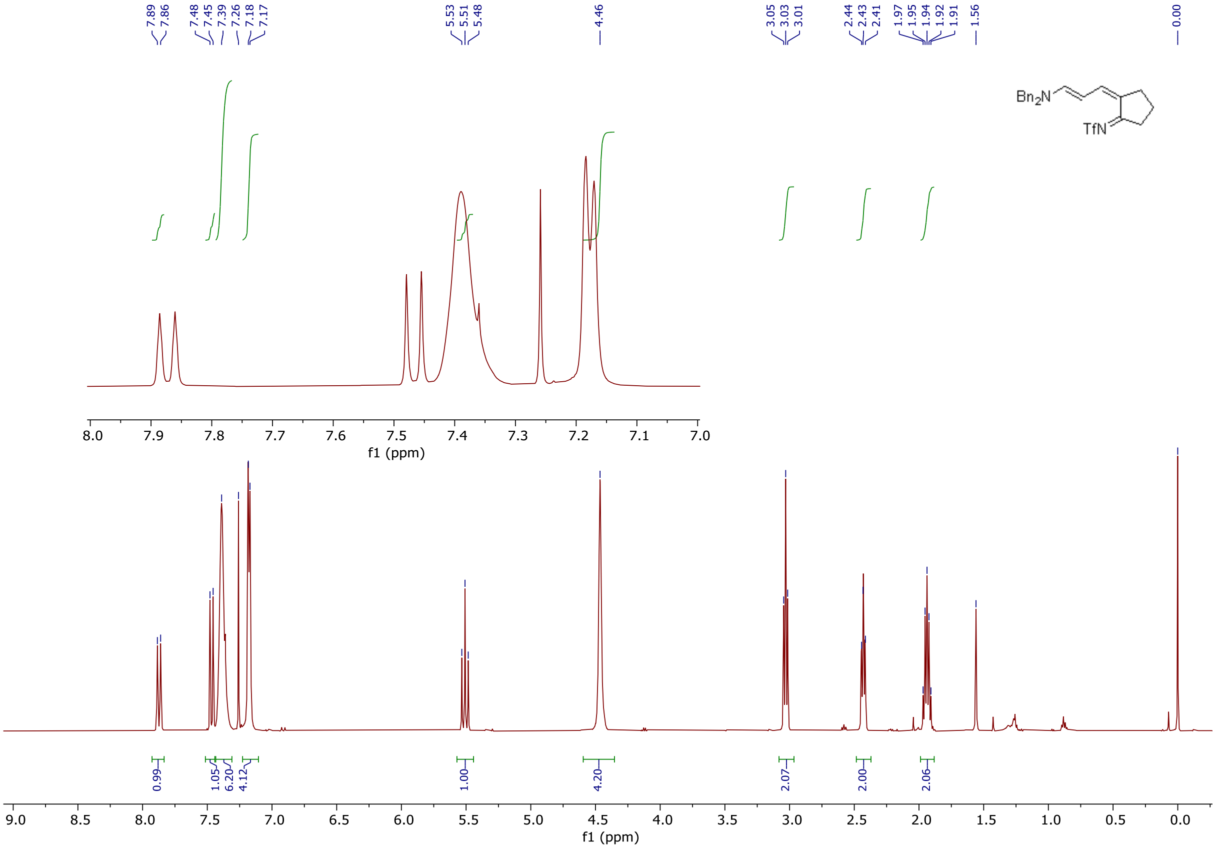

^{13}C NMR spectrum of Zincke imine S47b ($CDCl_3$, 298 K)

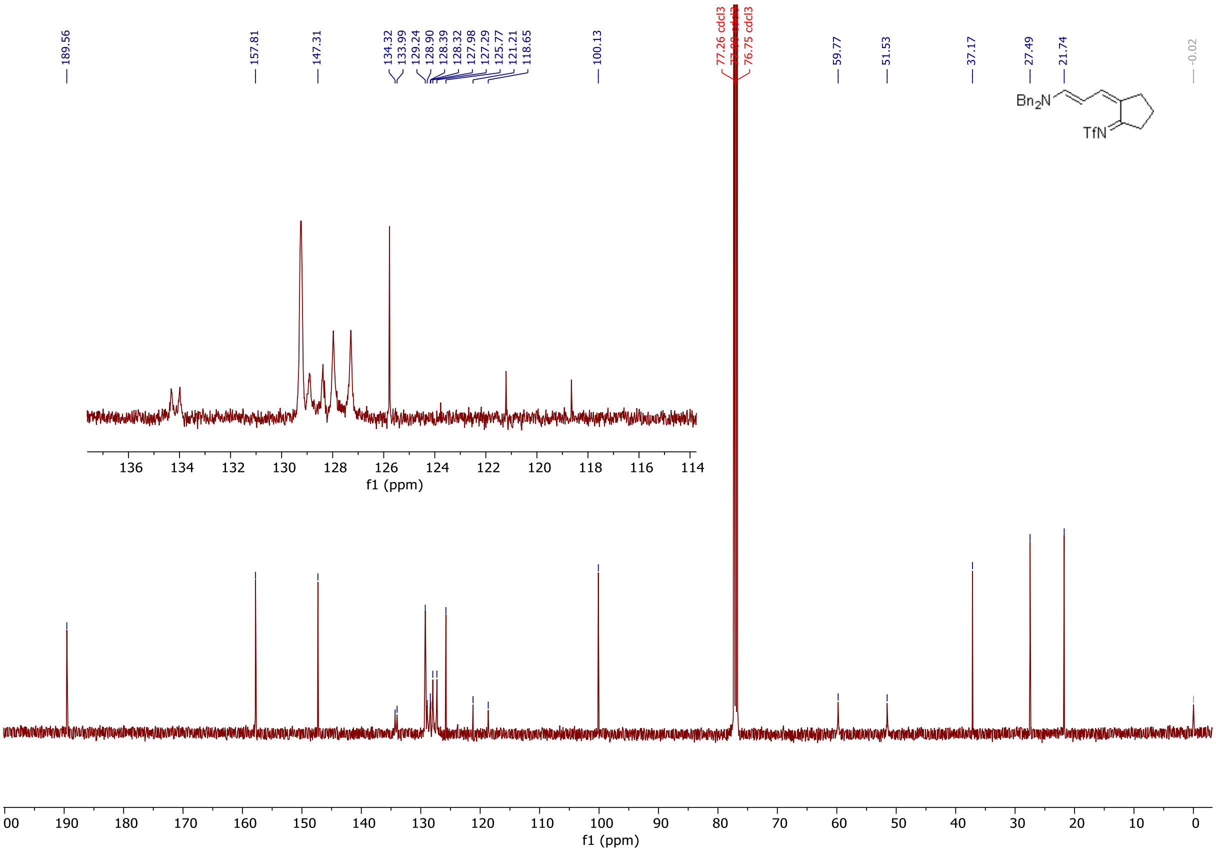

^{1}H NMR spectrum of Zincke imine S48b ($CDCl_3$, 298 K)

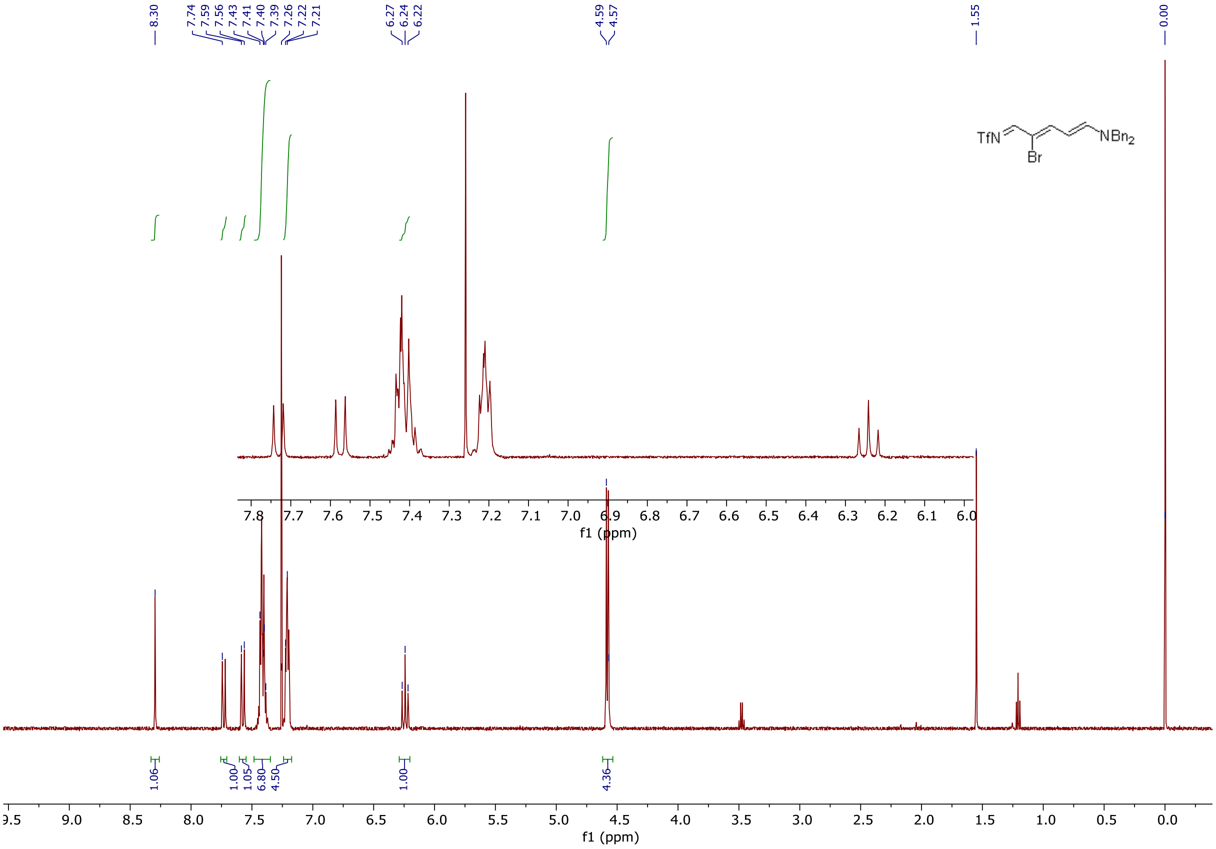

^{13}C NMR spectrum of Zincke imine S48b ($CDCl_3$, 298 K)

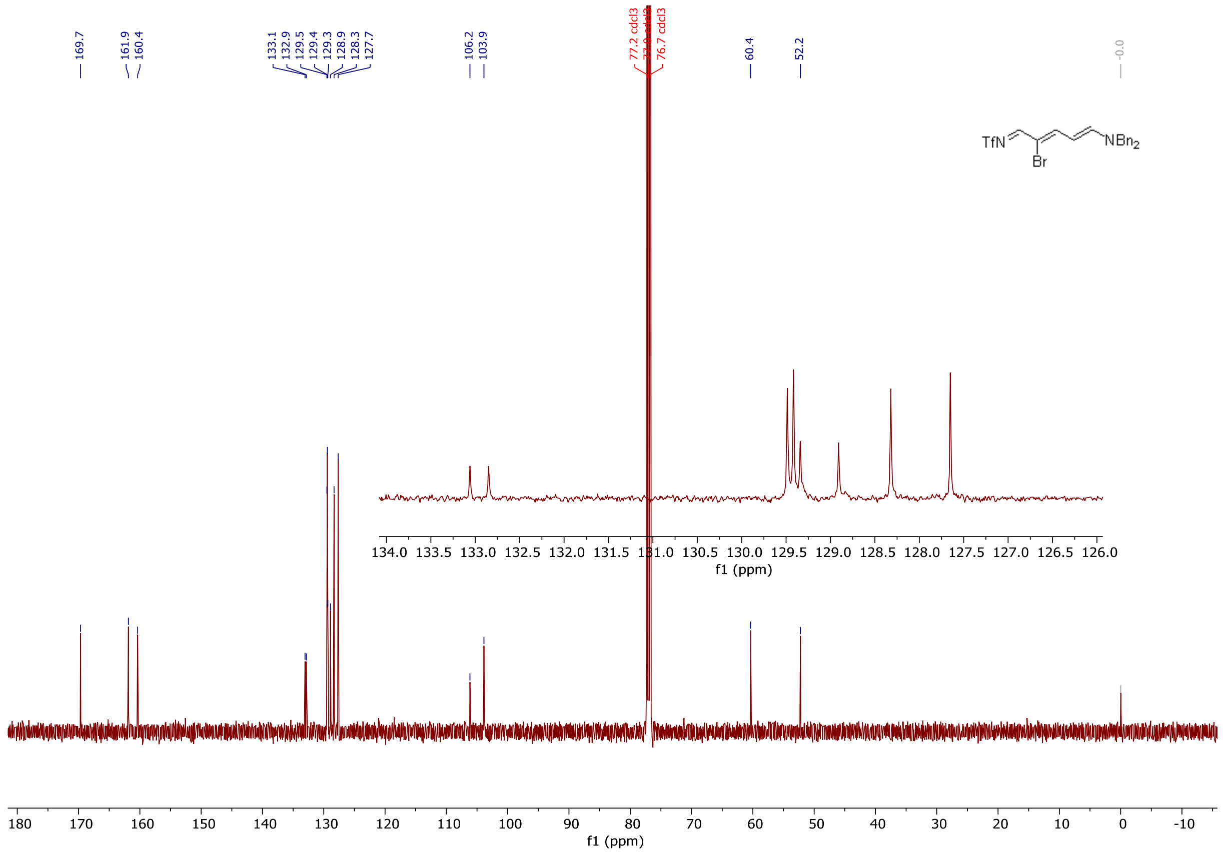

^{1}H NMR spectrum of compound S49b ($CDCl_3$, 298 K)

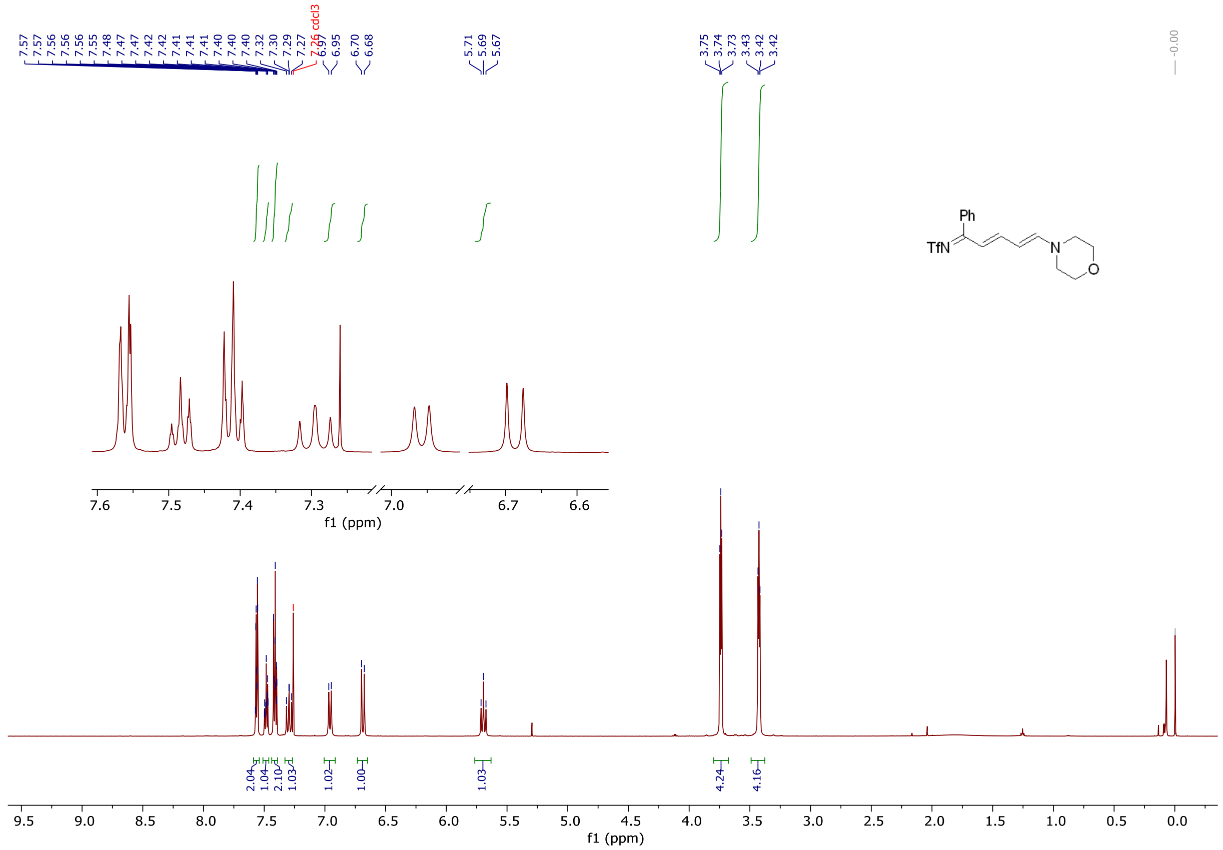

^{1}H NMR spectrum of compound S50b (CDCl$_3$, 298 K)

6.3. Functionalised pyridines

^{1}H NMR spectrum of compound 3a (CDCl$_3$, 298 K)

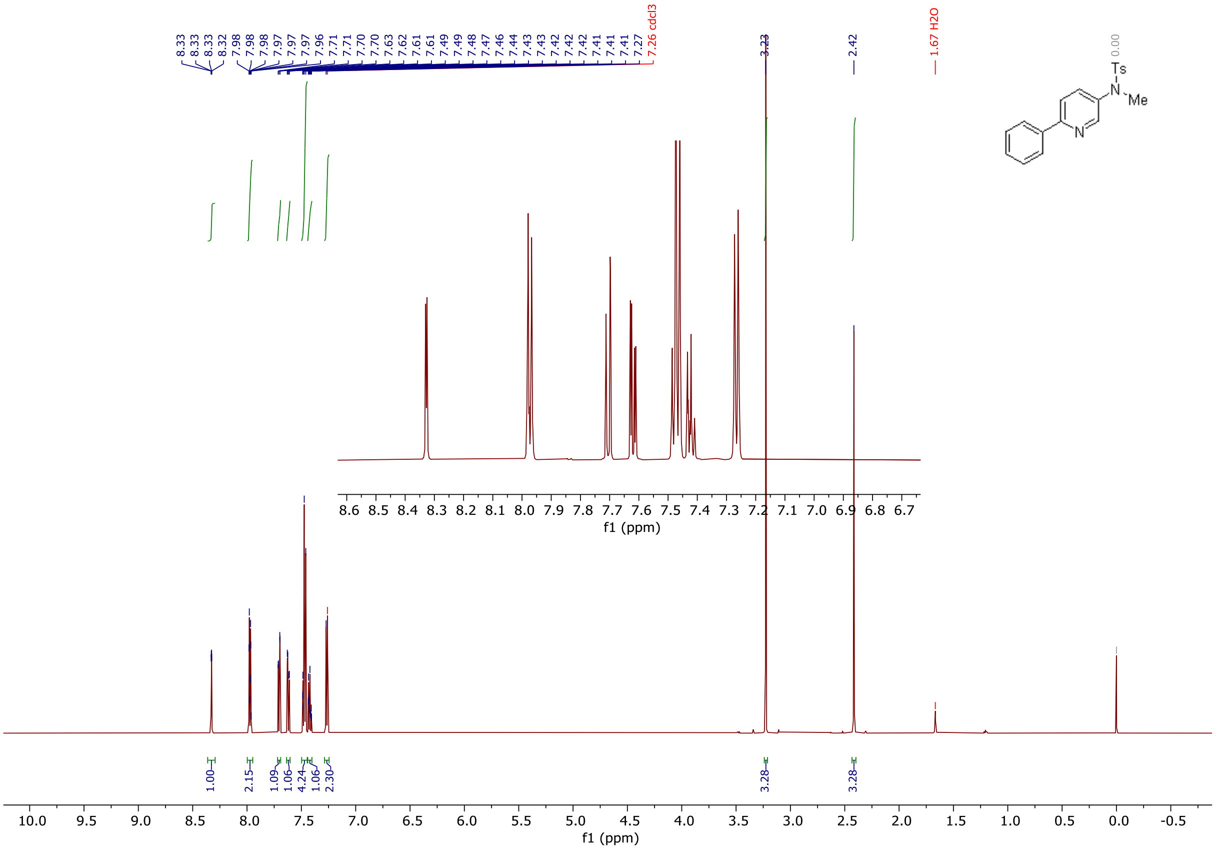

^{1}H-^{1}H COSY spectrum of compound 3a (CDCl$_3$, 298 K)

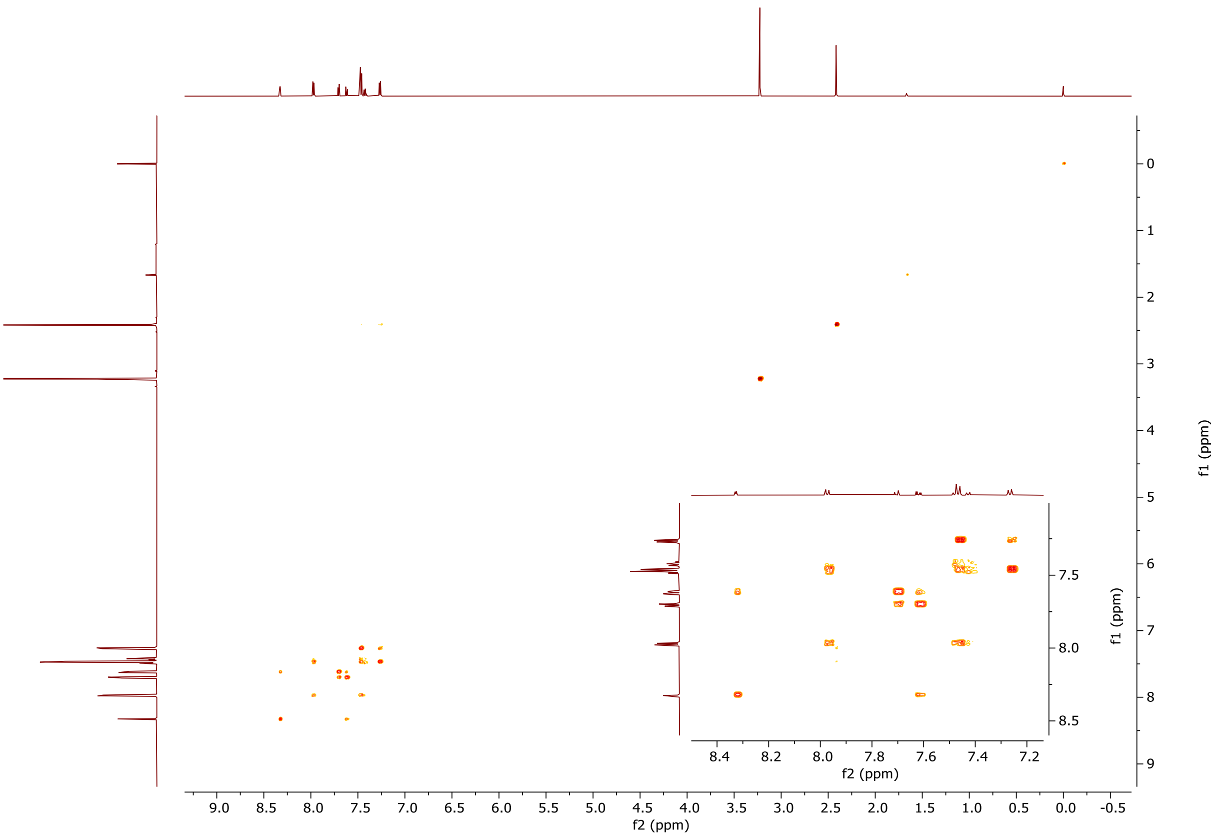

^{13}C NMR spectrum of compound 3a (CDCl$_3$, 298 K)

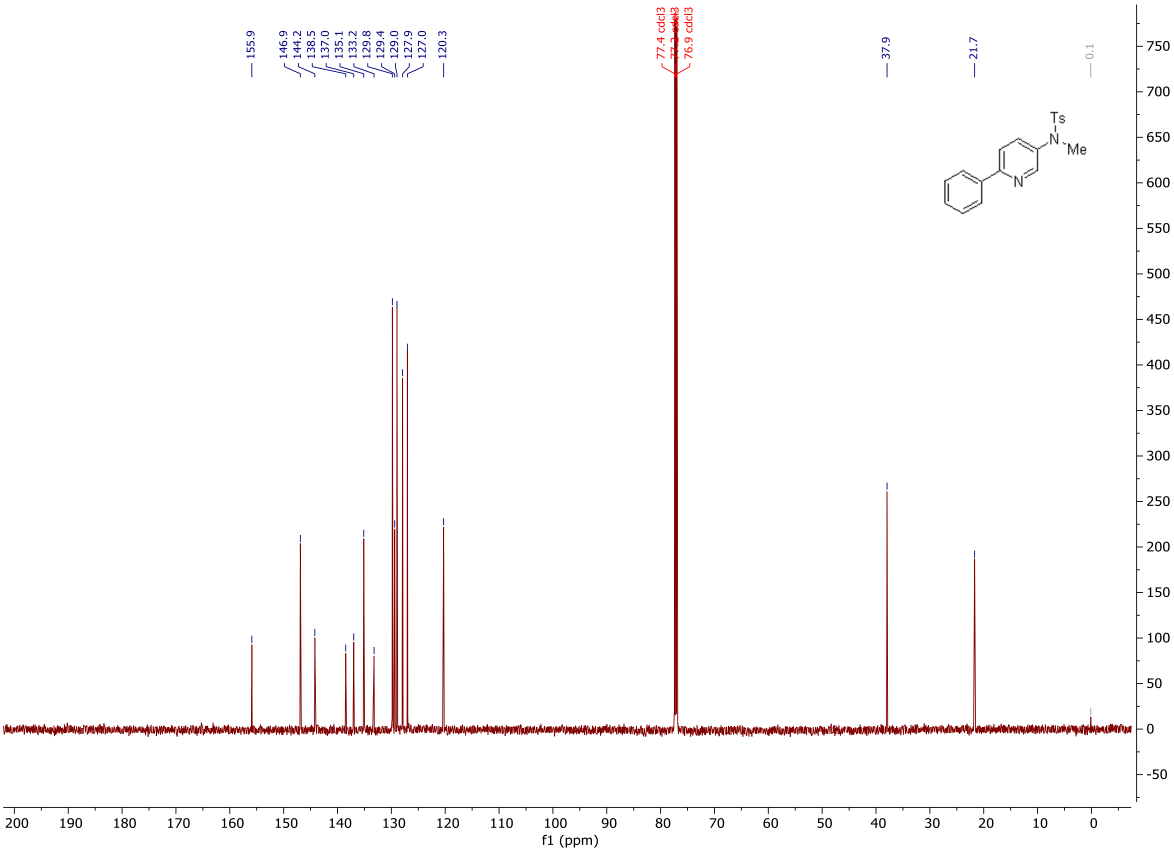

^{1}H NMR spectrum of compound 3b ($CDCl_3$, 298 K)

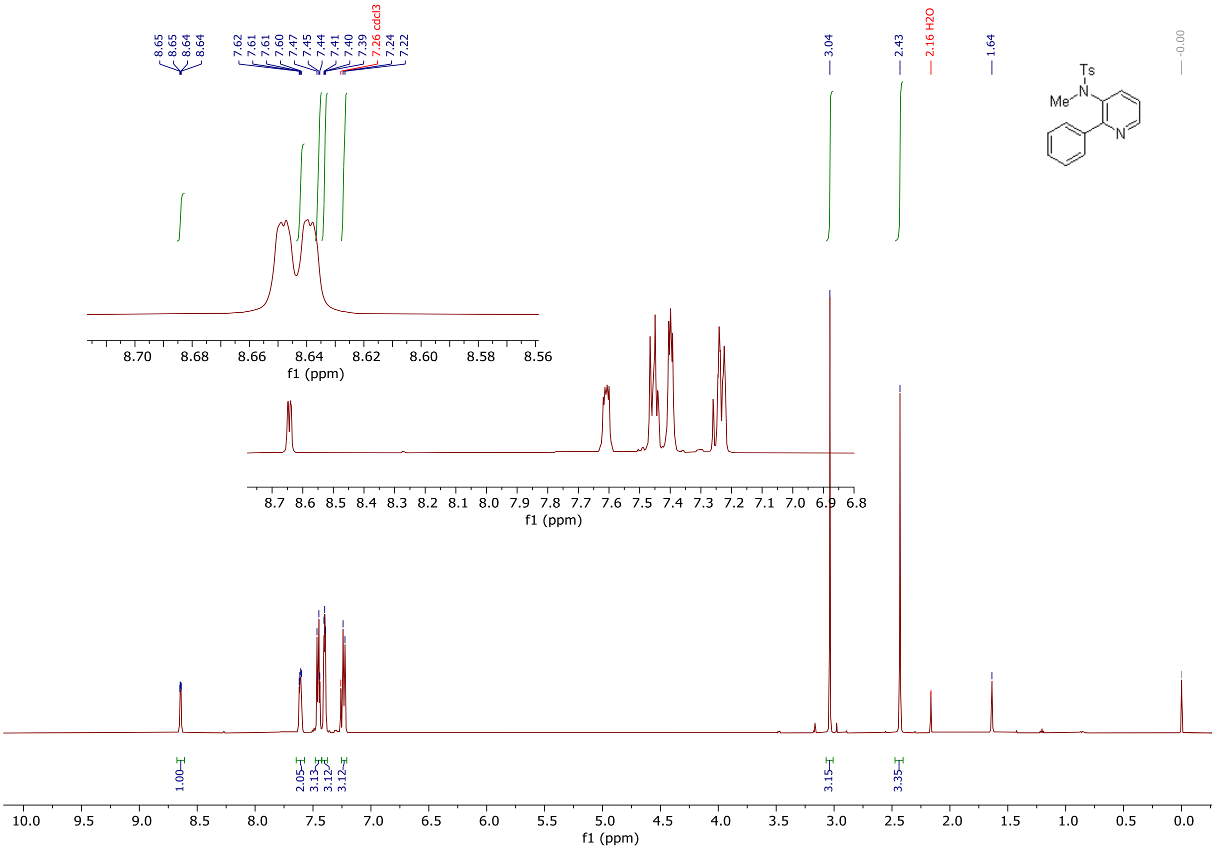

^{1}H-^{1}H COSY spectrum of compound 3b ($CDCl_3$, 298 K)

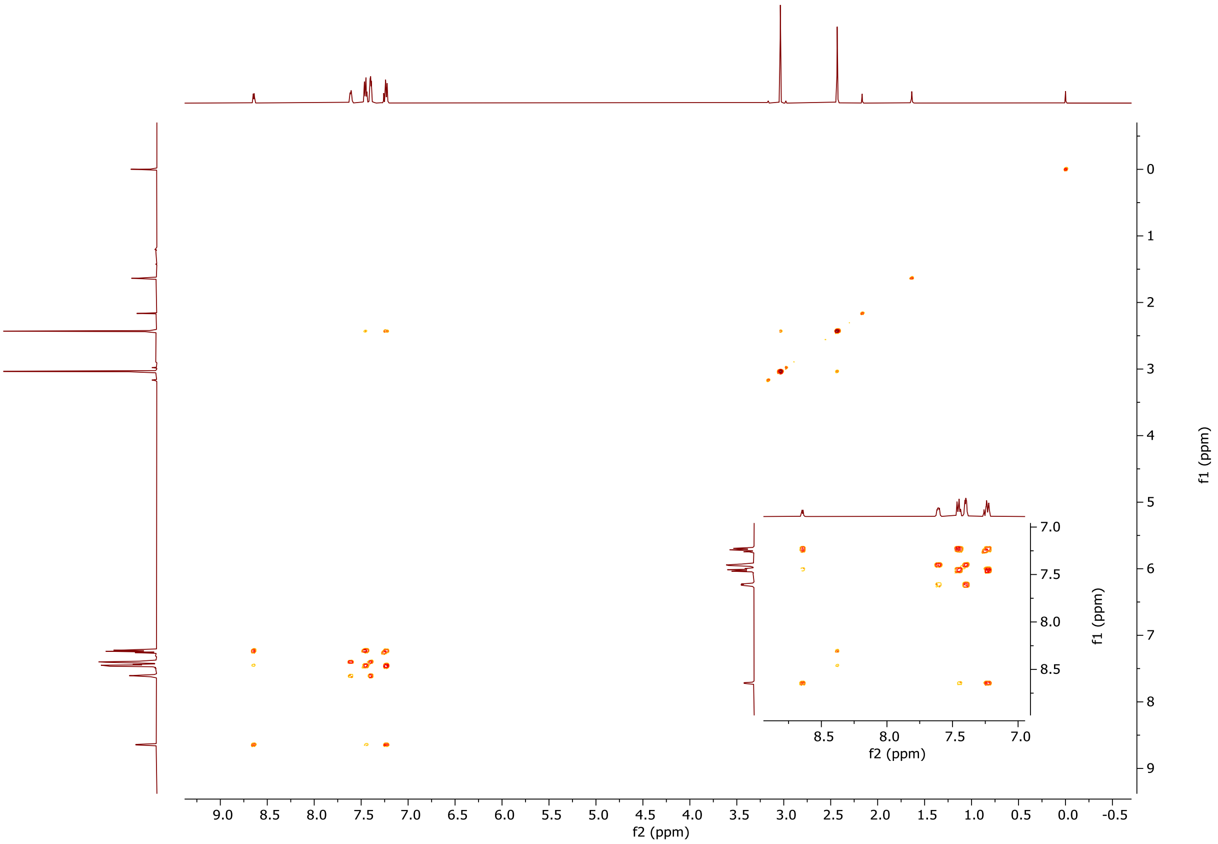

^{13}C NMR spectrum of compound 3b ($CDCl_3$, 298 K)

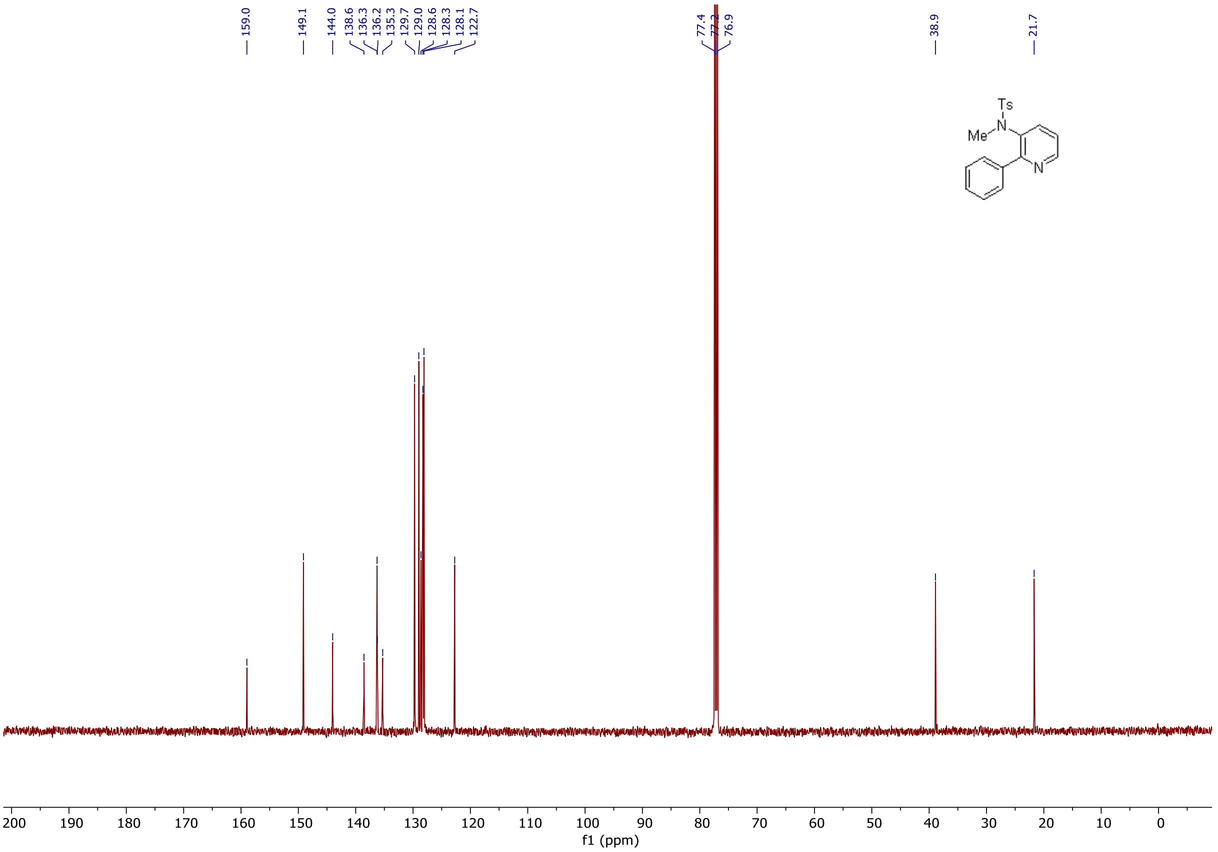

^{1}H NMR spectrum of compound 3c ($CDCl_3$, 298 K)

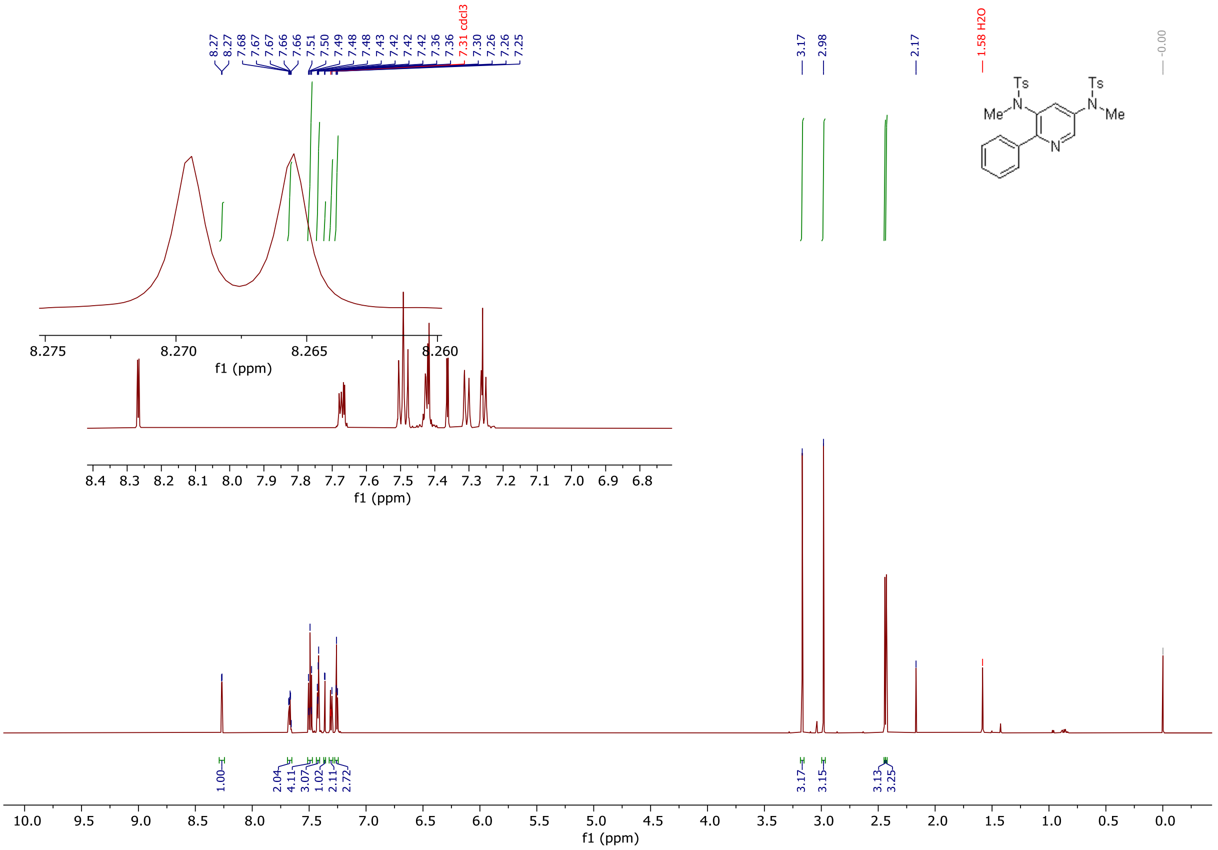

^{1}H-^{1}H COSY spectrum of compound 3c ($CDCl_3$, 298 K)

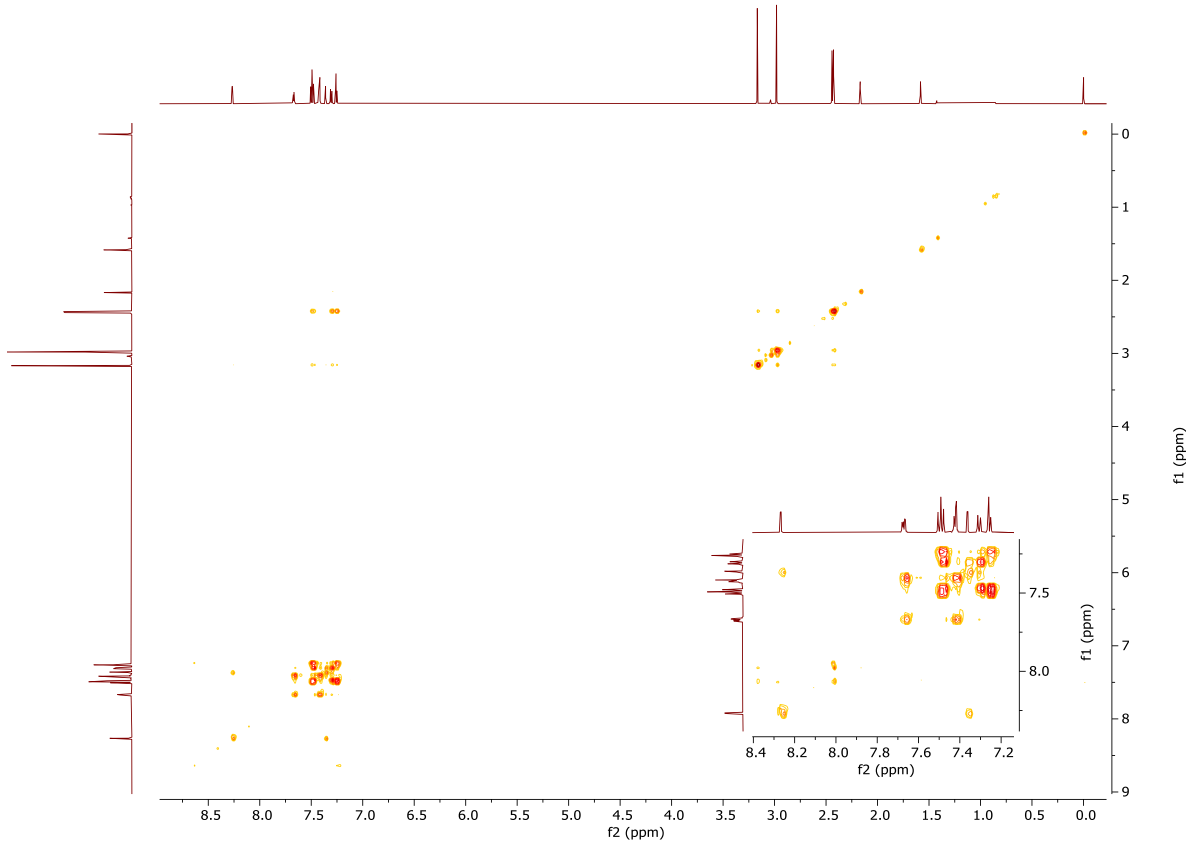

^{13}C NMR spectrum of compound 3c ($CDCl_3$, 298 K)

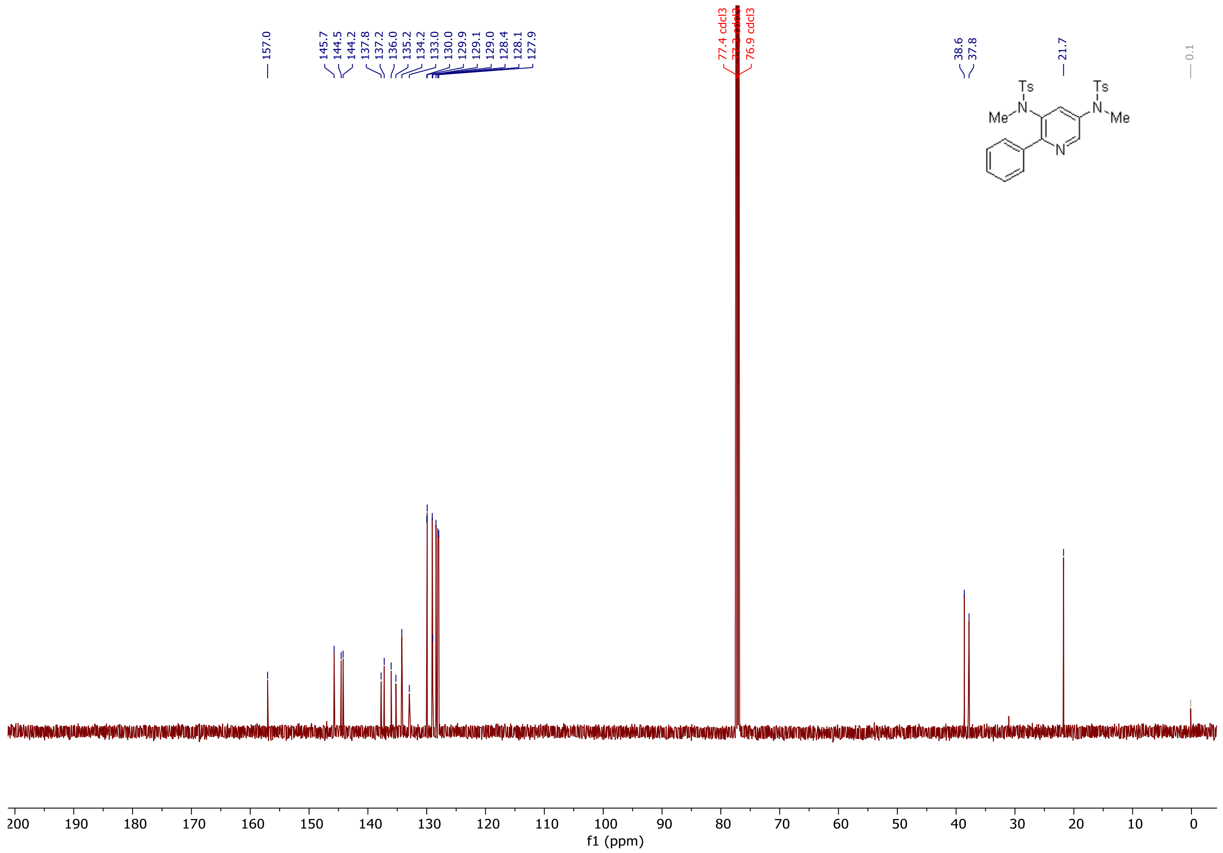

^{1}H NMR spectrum of compound 2a ($CDCl_3$, 298 K)

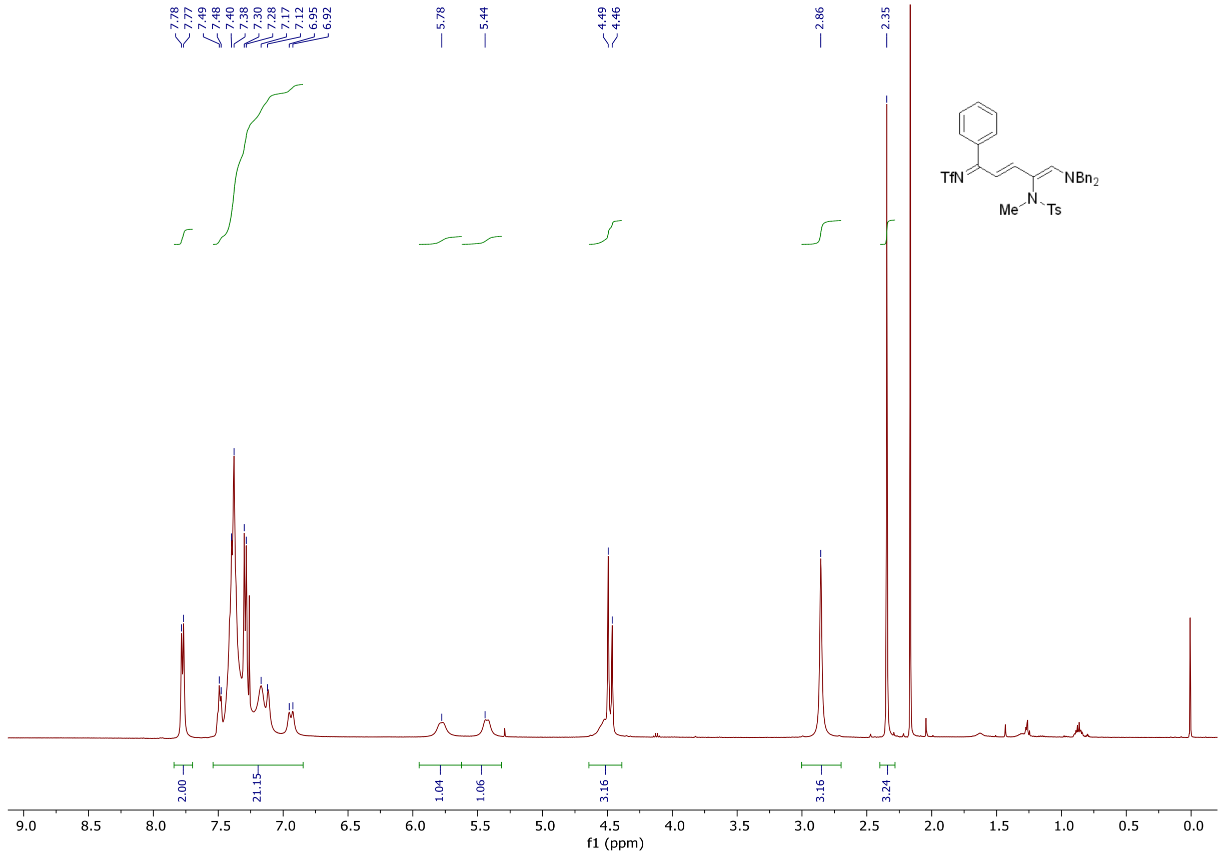

^{13}C NMR spectrum of compound 2a ($CDCl_3$, 298 K)

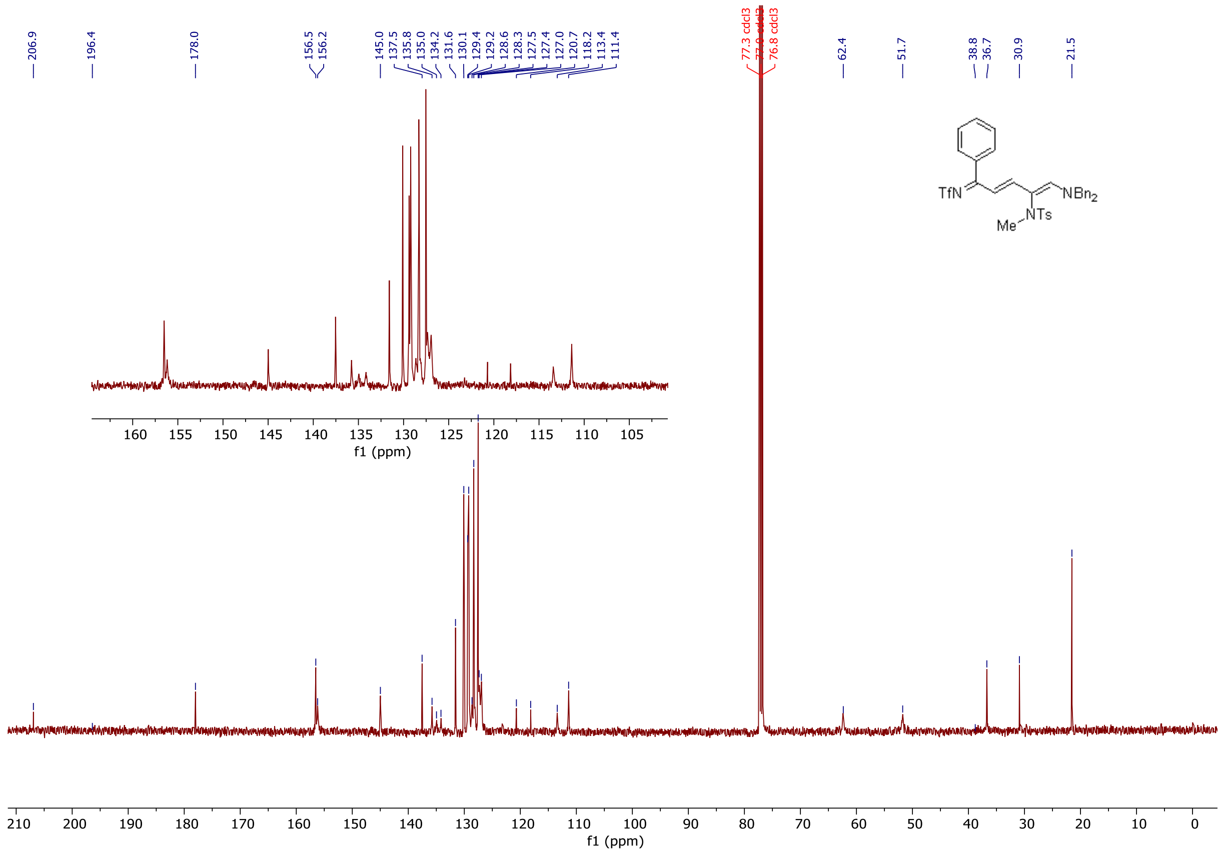

^{1}H-^{1}H COSY spectrum of compound 2a ($CDCl_3$, 298 K)

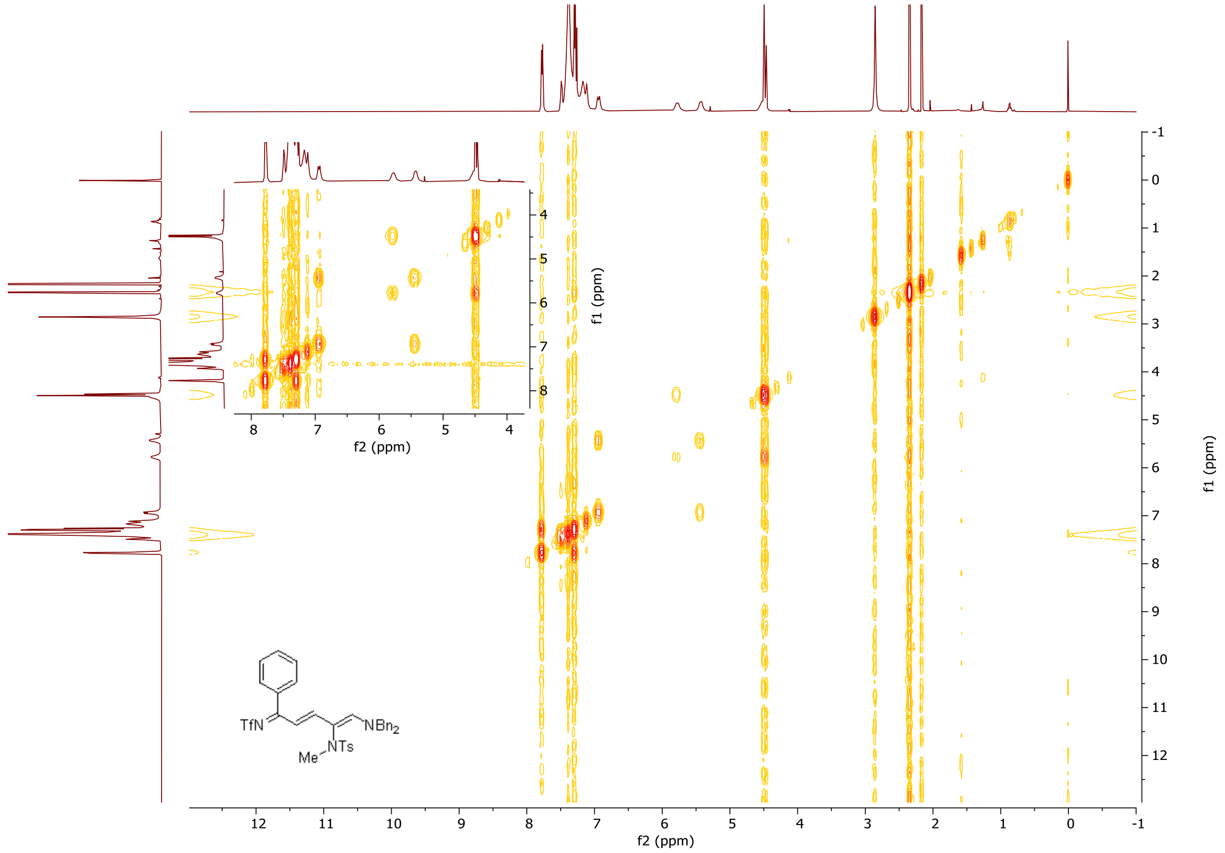

^{1}H NMR spectrum of compound 4a ($CDCl_3$, 298 K)

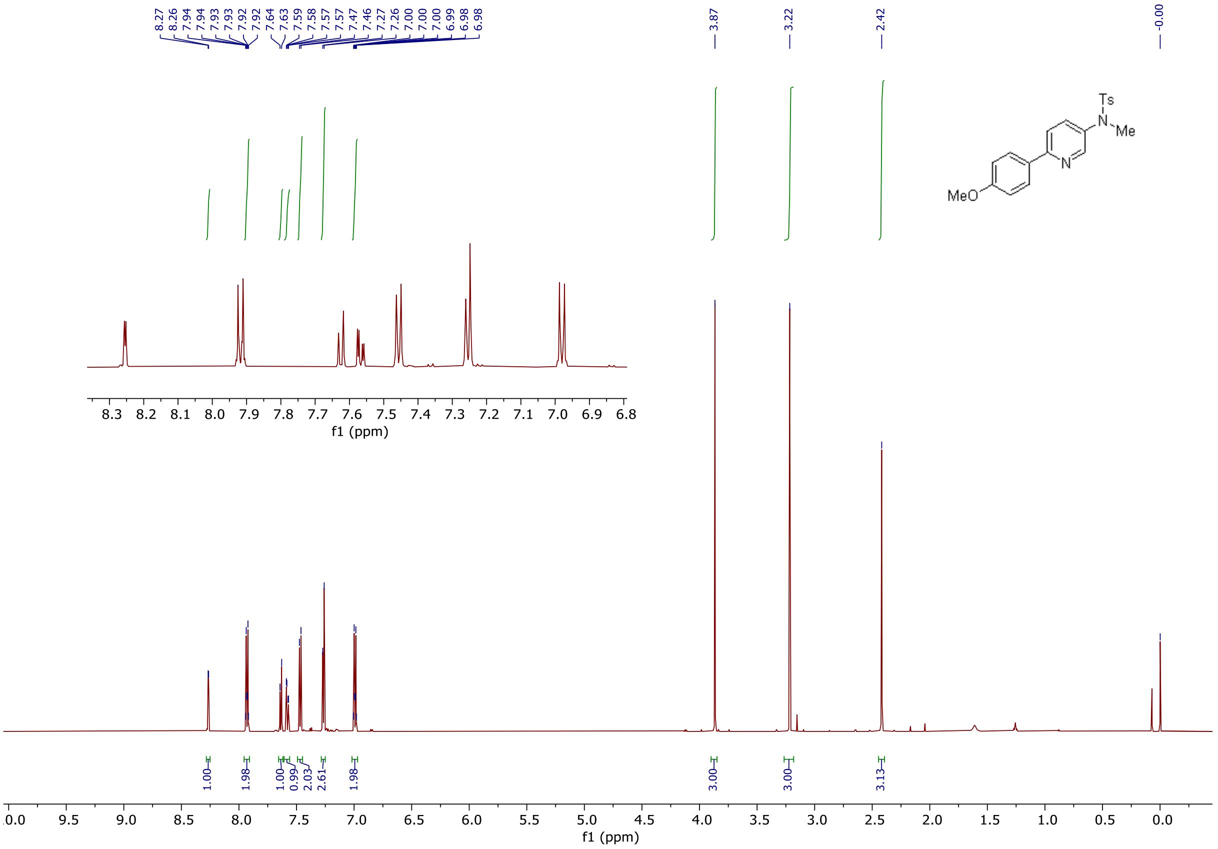

^{13}C NMR spectrum of compound 4a ($CDCl_3$, 298 K)

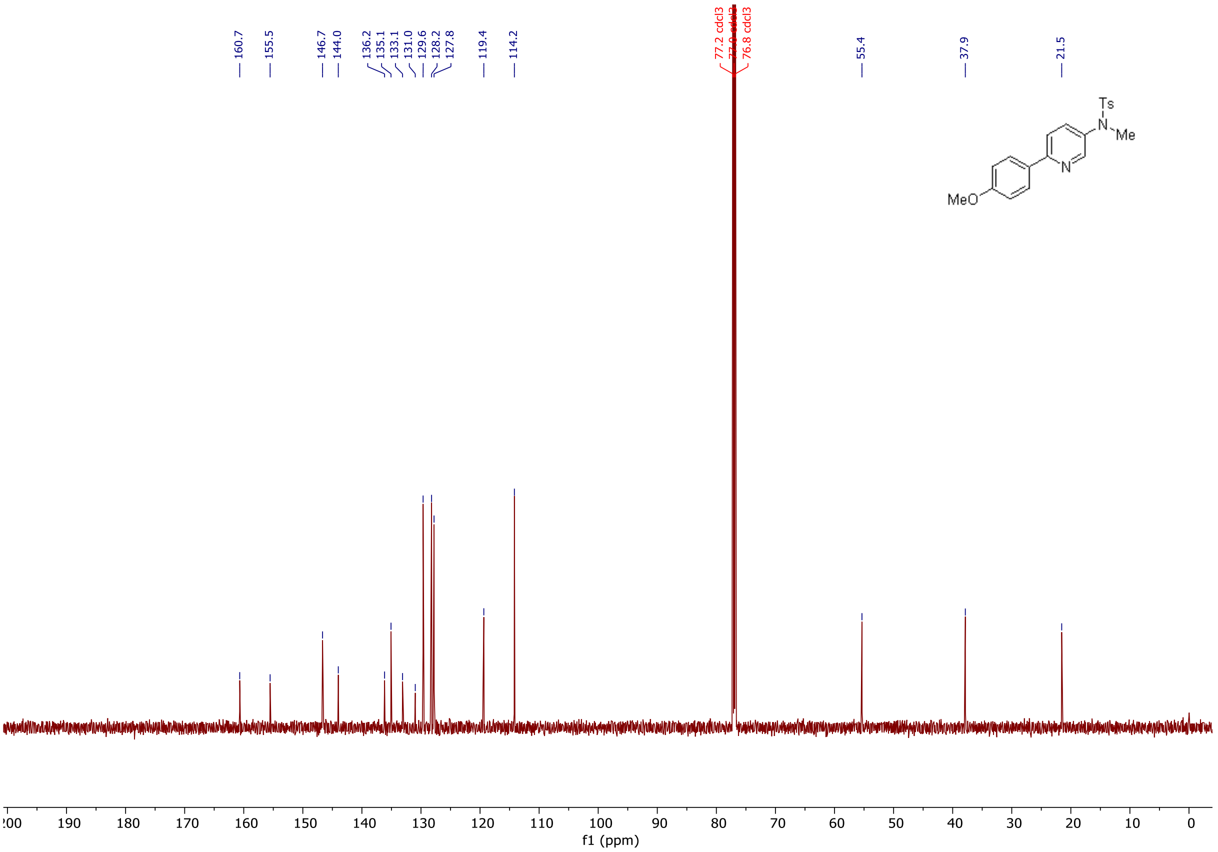

^{1}H NMR spectrum of compound 4b ($CDCl_3$, 298 K)

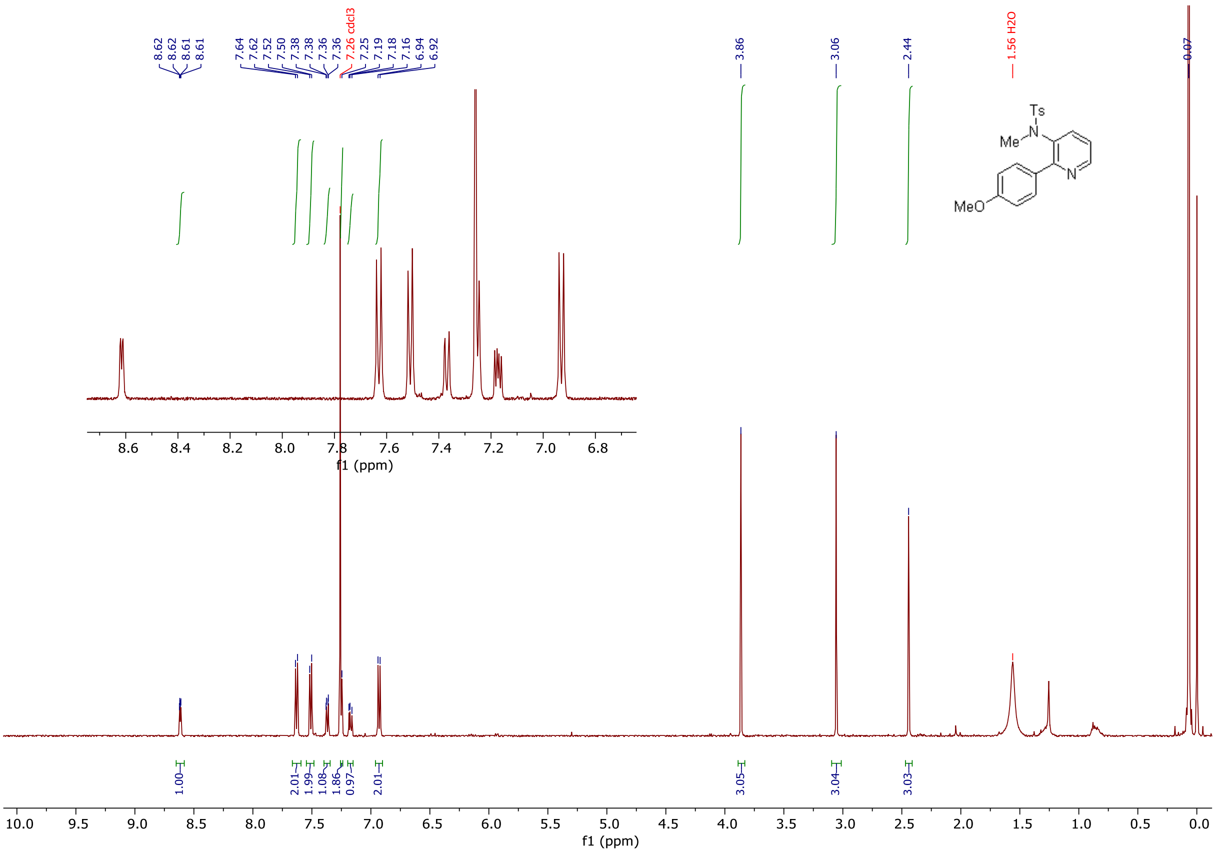

^{13}C NMR spectrum of compound 4b ($CDCl_3$, 298 K)

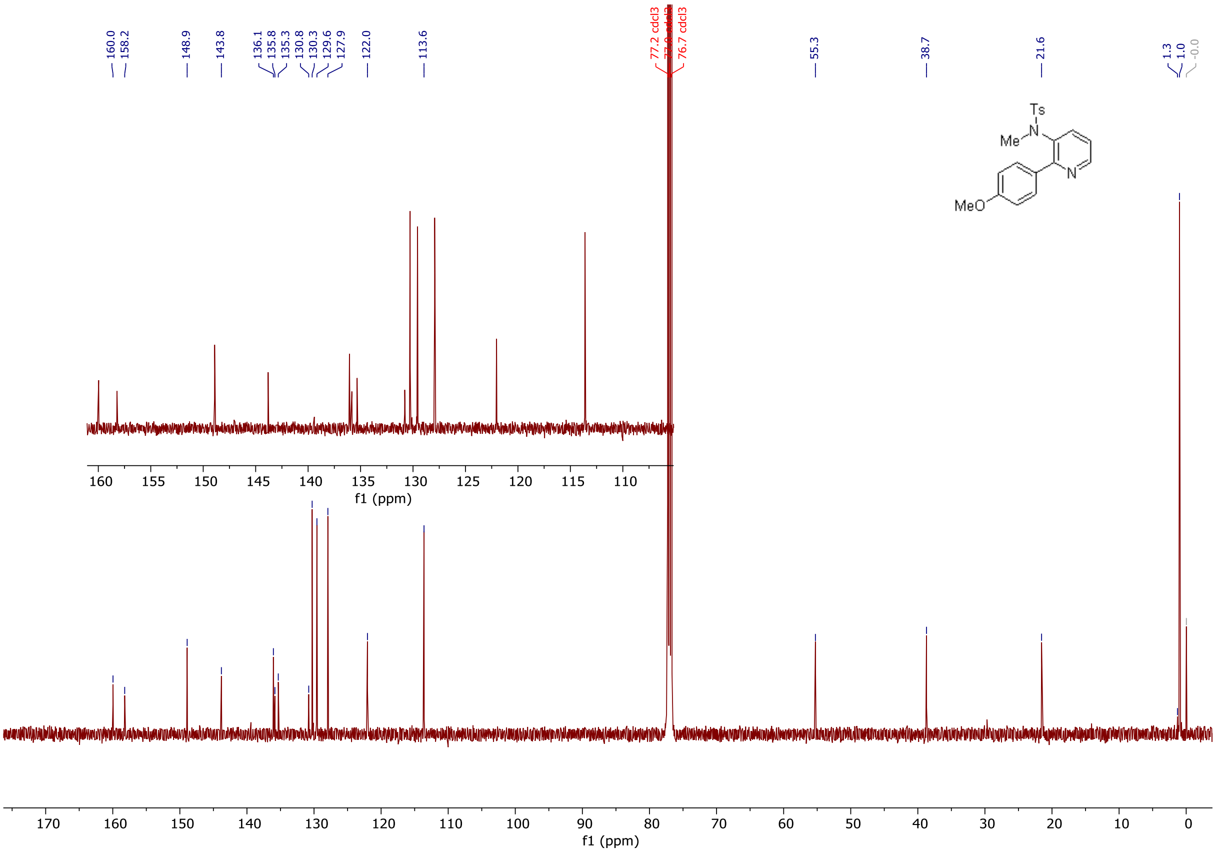

^{1}H NMR spectrum of compound 5a ($CDCl_3$, 298 K)

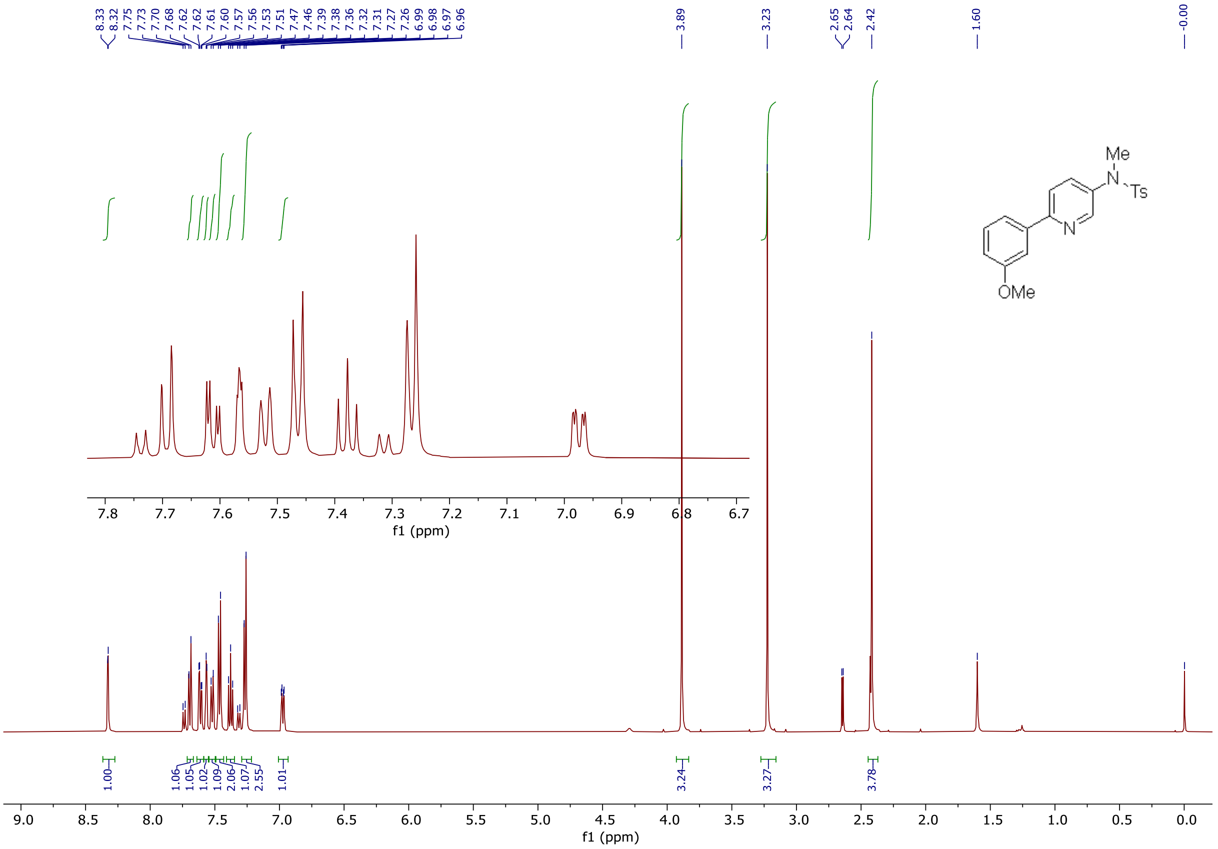

^{13}C NMR spectrum of compound 5a ($CDCl_3$, 298 K)

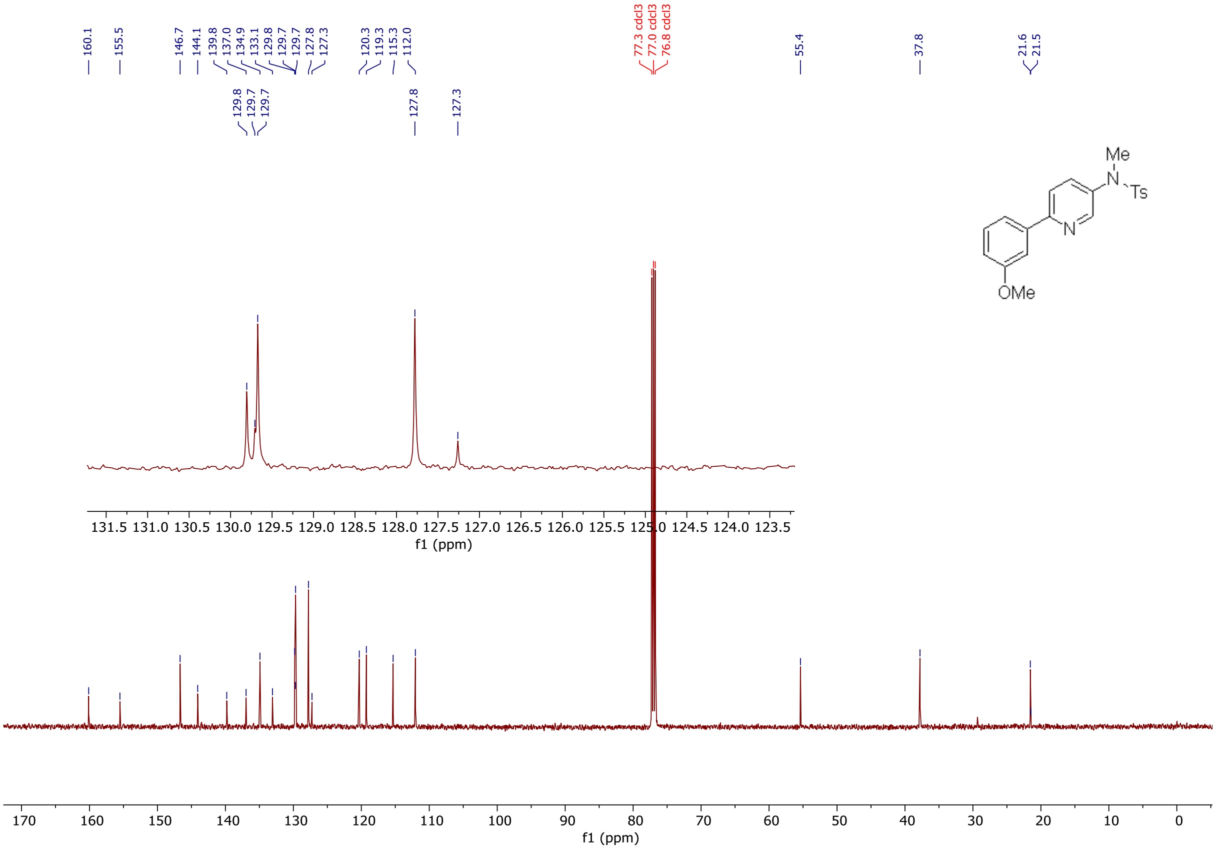

^{1}H NMR spectrum of compound 5b ($CDCl_3$, 298 K)

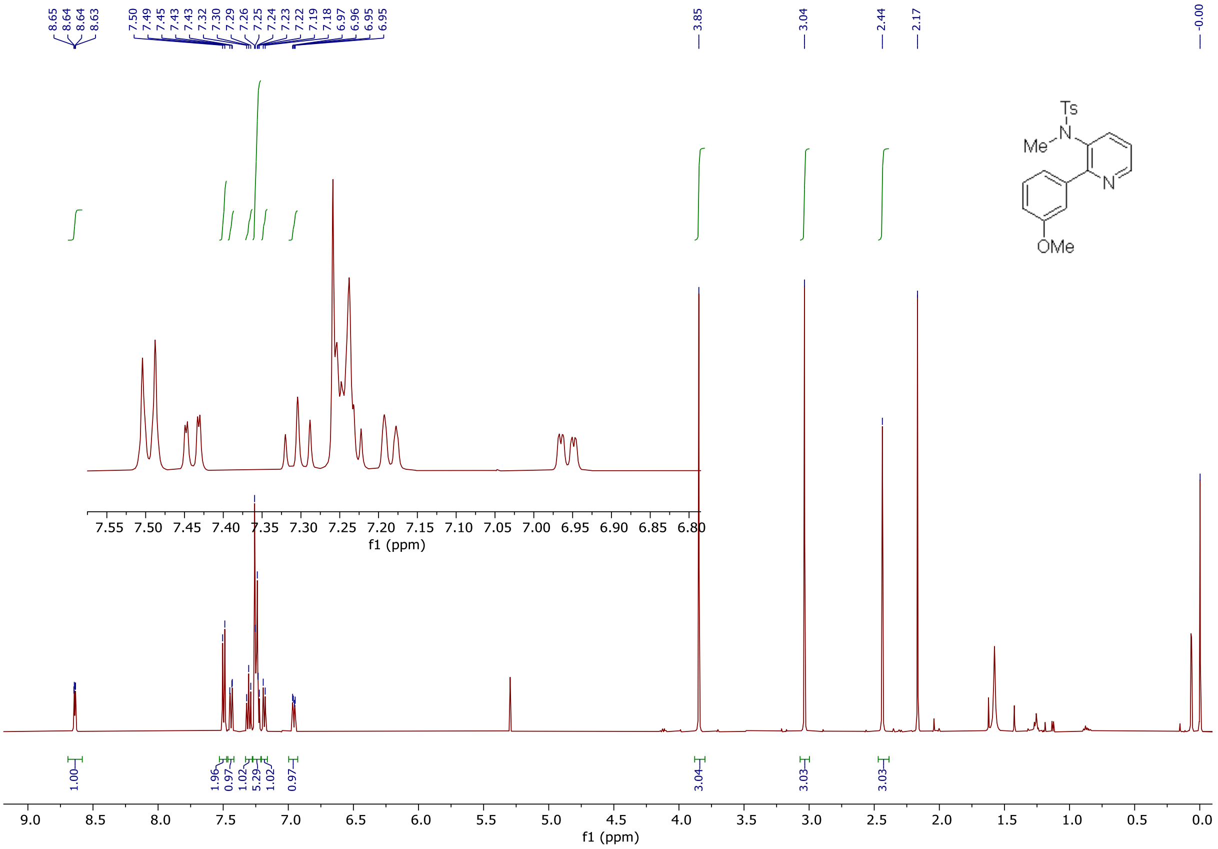

^{13}C NMR spectrum of compound 5b ($CDCl_3$, 298 K)

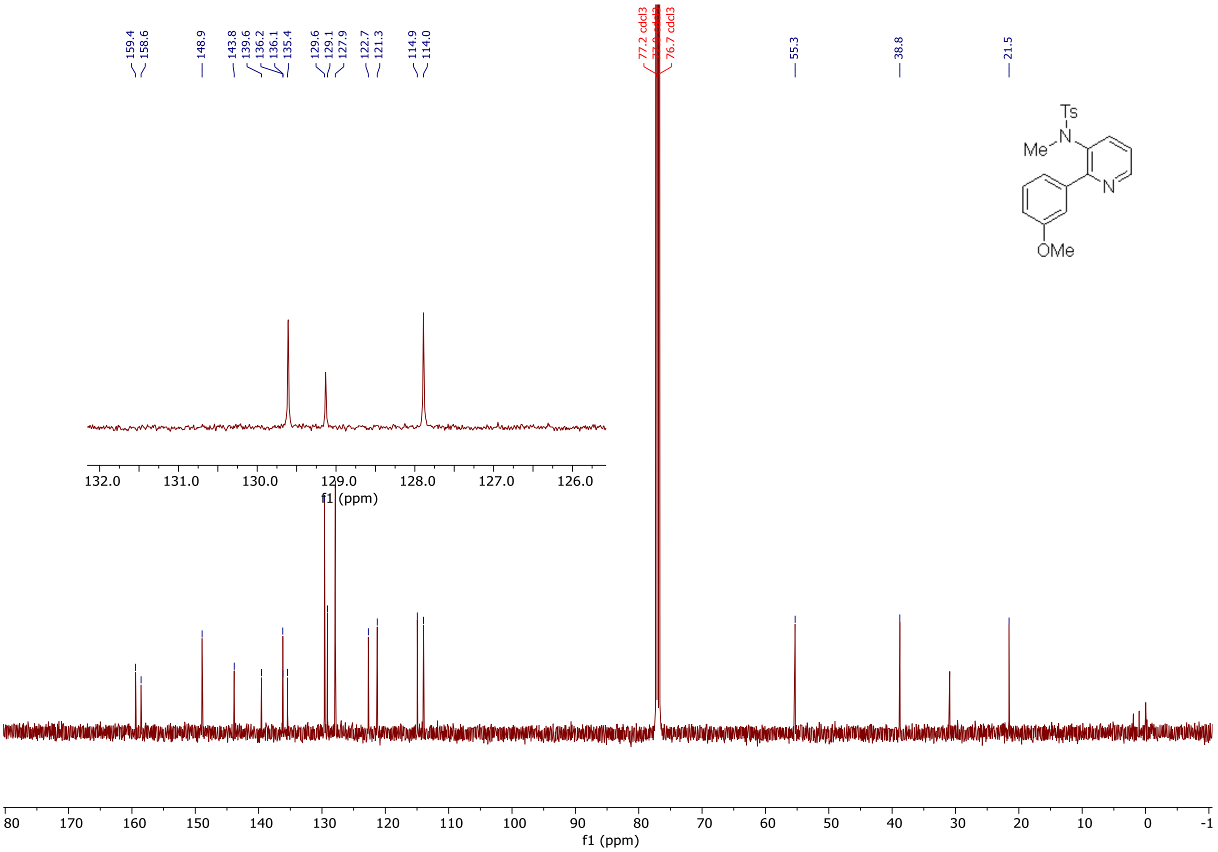

^{1}H NMR spectrum of compound 6a ($CDCl_3$, 298 K)

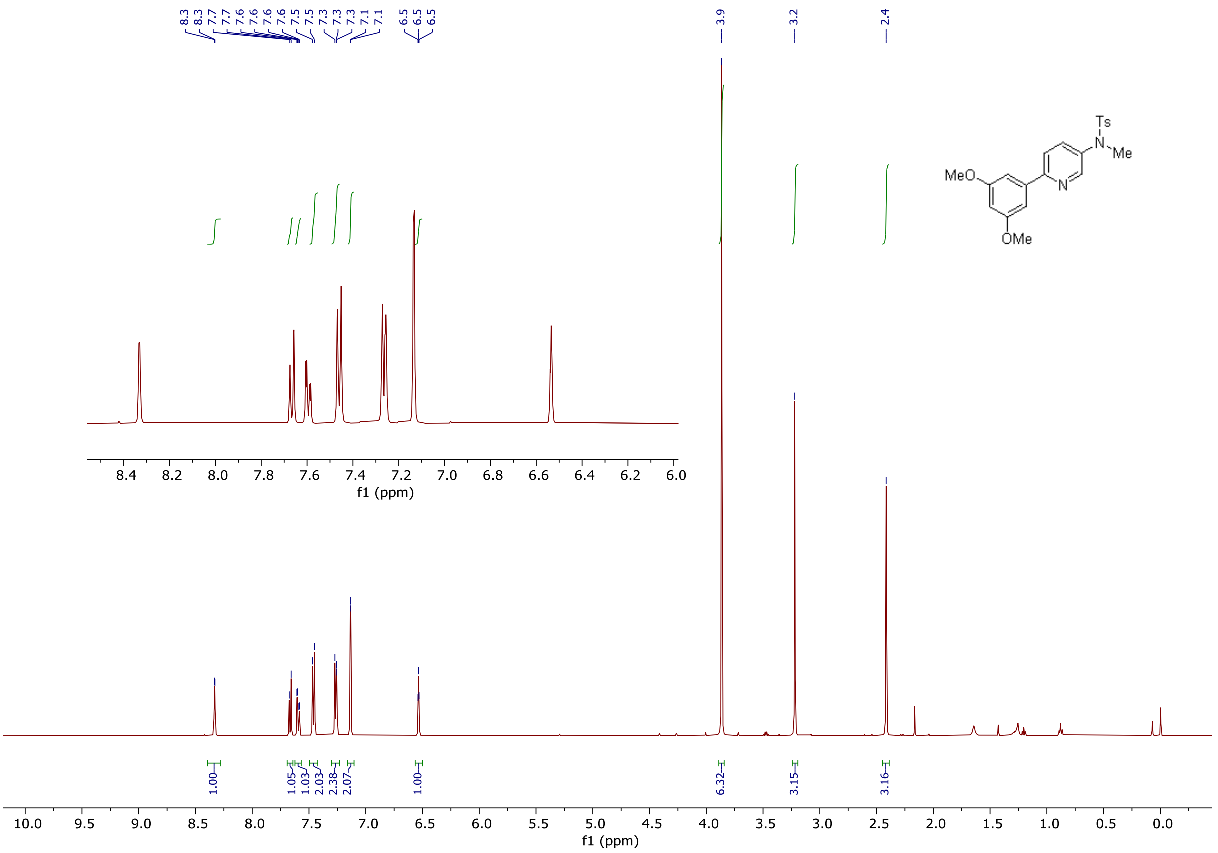

^{13}C NMR spectrum of compound 6a ($CDCl_3$, 298 K)

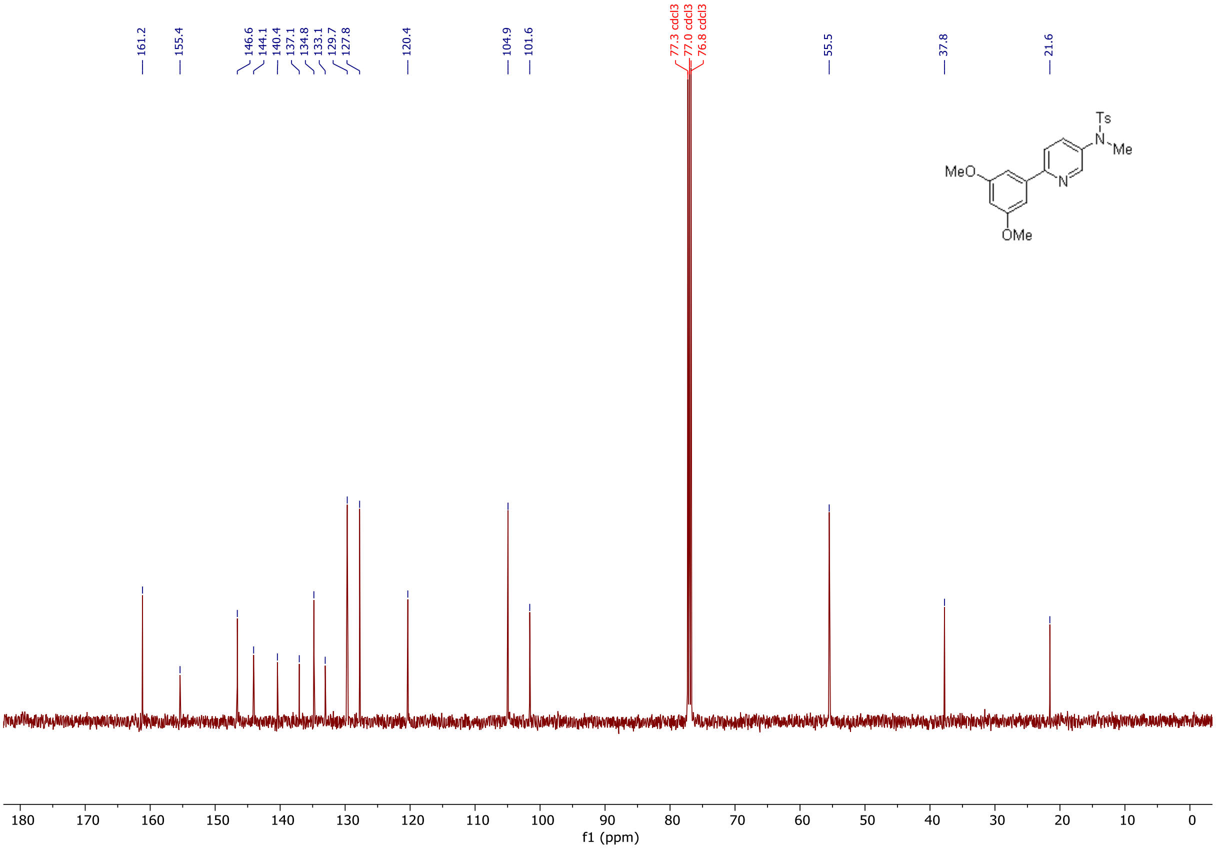

^{1}H NMR spectrum of compound 6b ($CDCl_3$, 298 K)

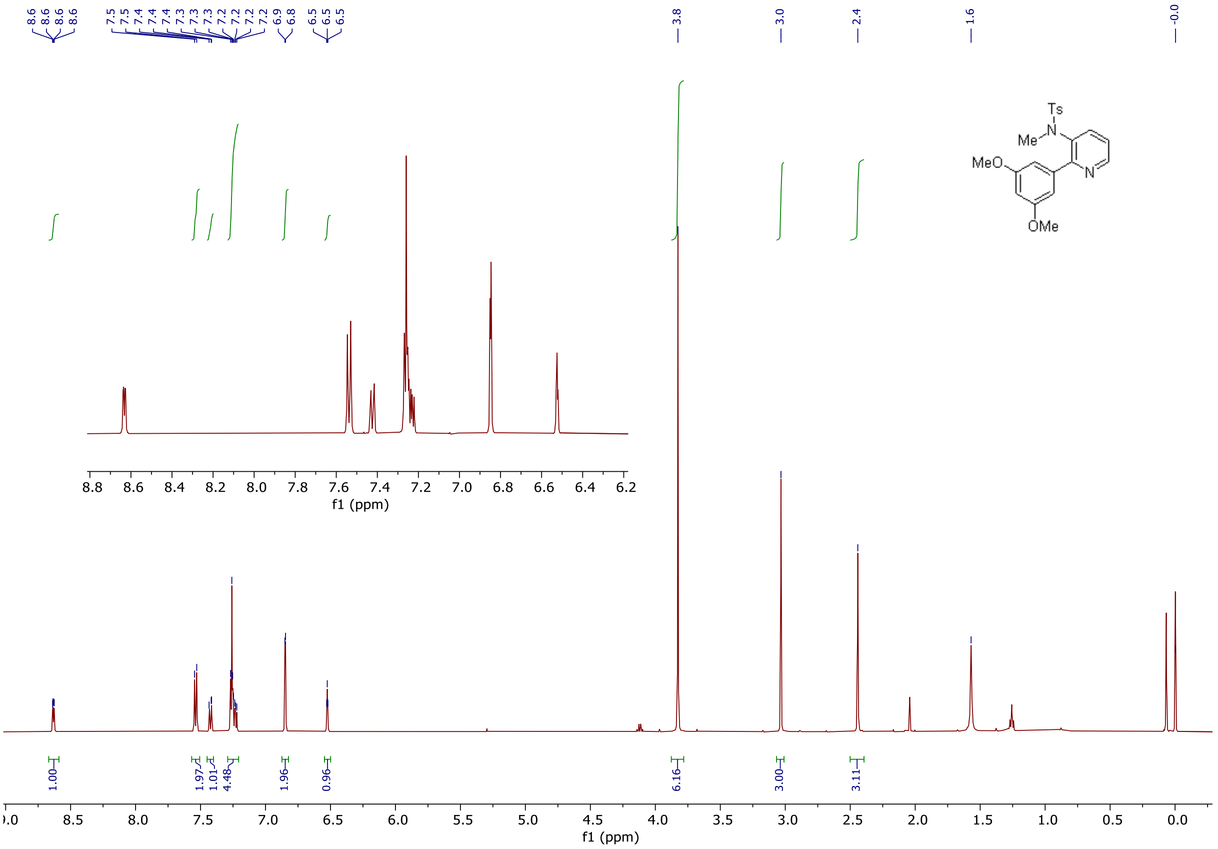

^{13}C NMR spectrum of compound 6b ($CDCl_3$, 298 K)

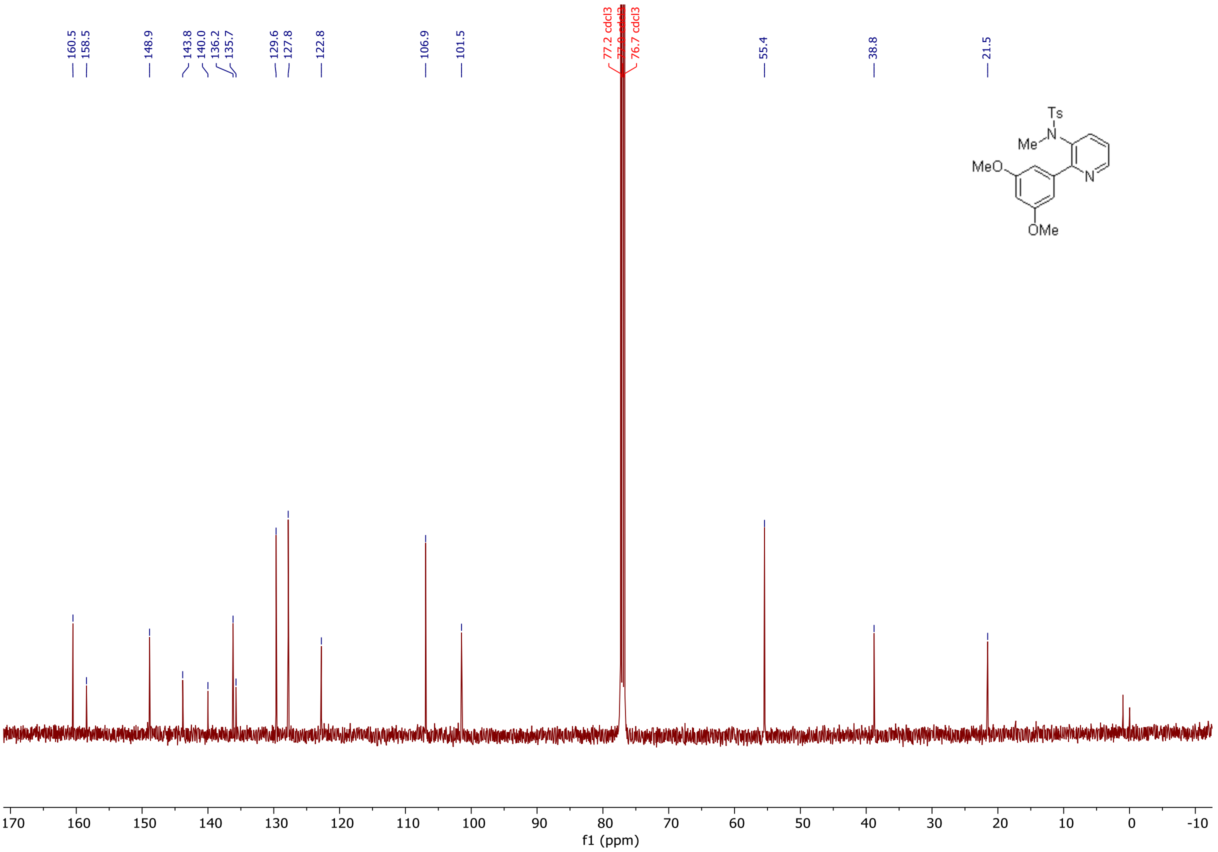

^{1}H NMR spectrum of compound 7a ($CDCl_3$, 298 K)

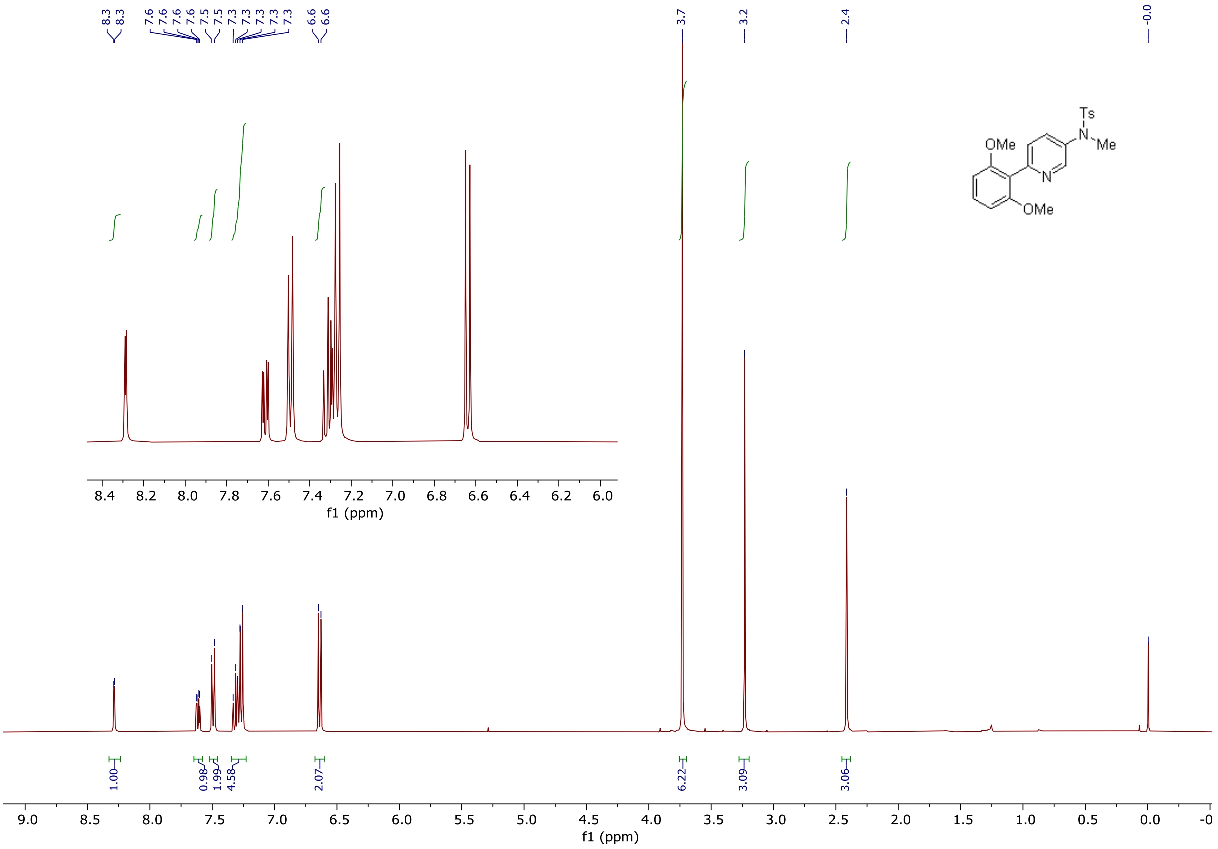

^{13}C NMR spectrum of compound 7a ($CDCl_3$, 298 K)

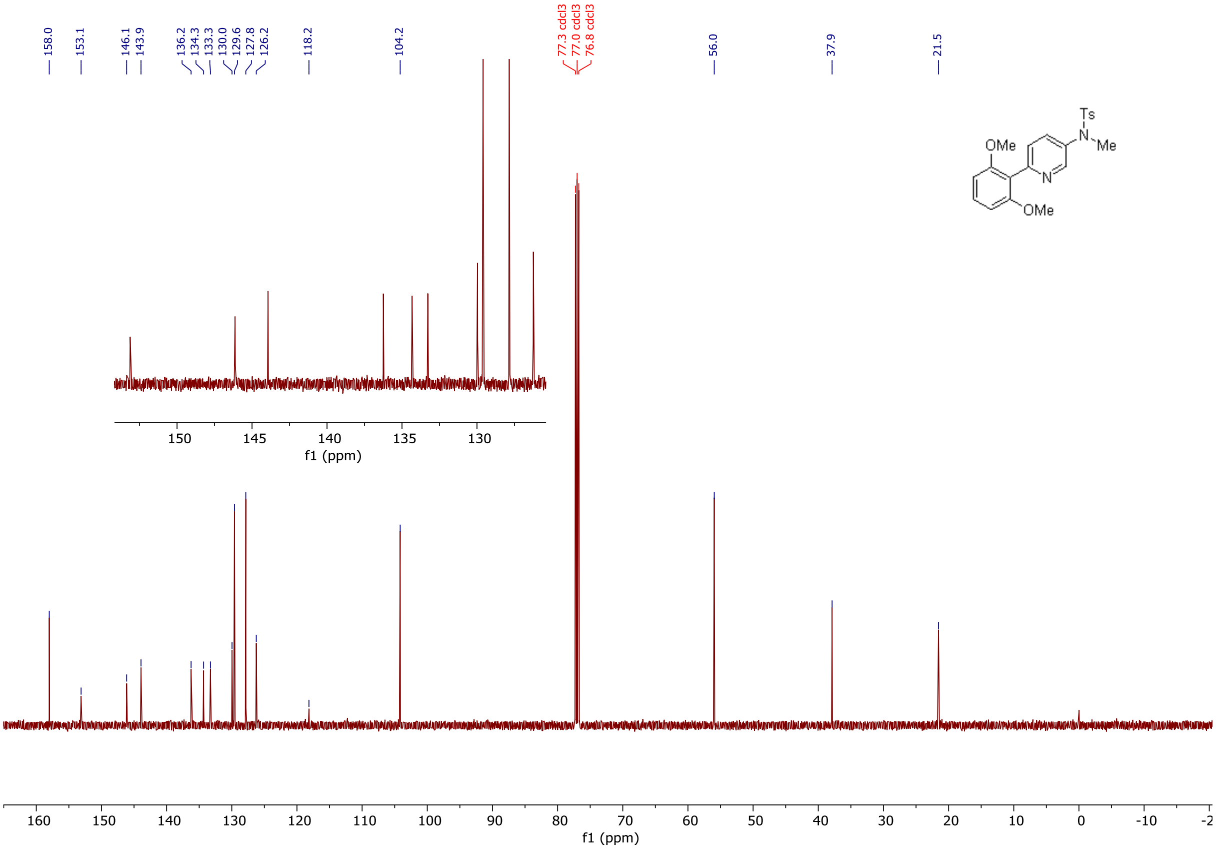

^{1}H NMR spectrum of compound 8a ($CDCl_3$, 298 K)

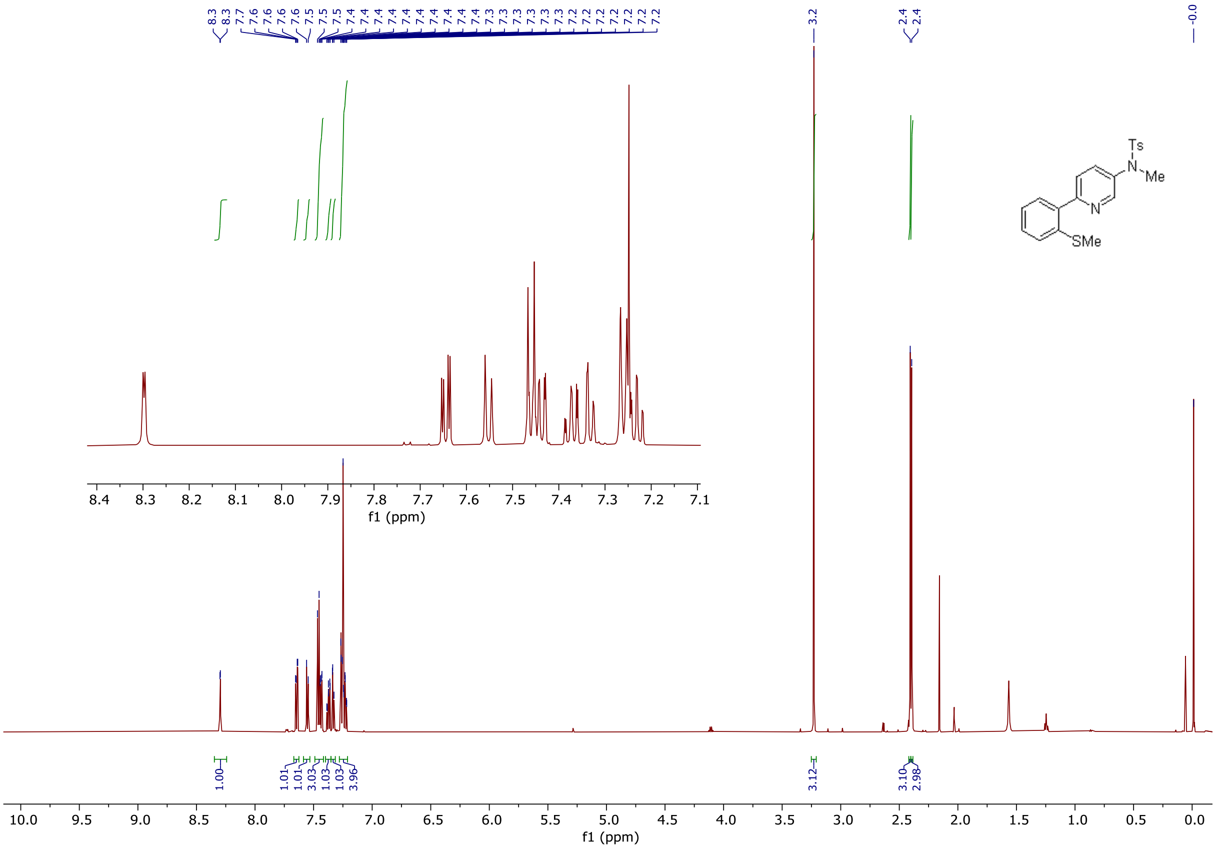

^{13}C NMR spectrum of compound 8a ($CDCl_3$, 298 K)

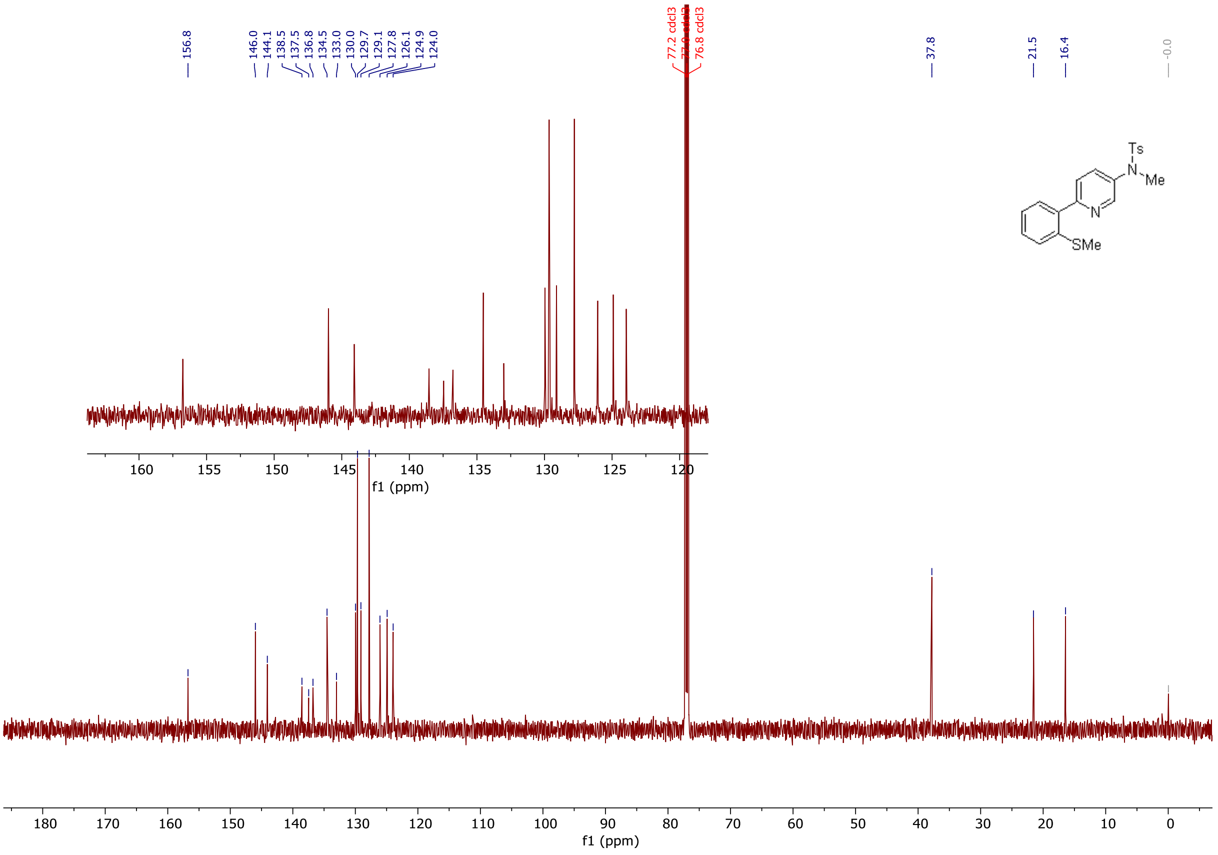

^{1}H NMR spectrum of compound 9a ($CDCl_3$, 298 K)

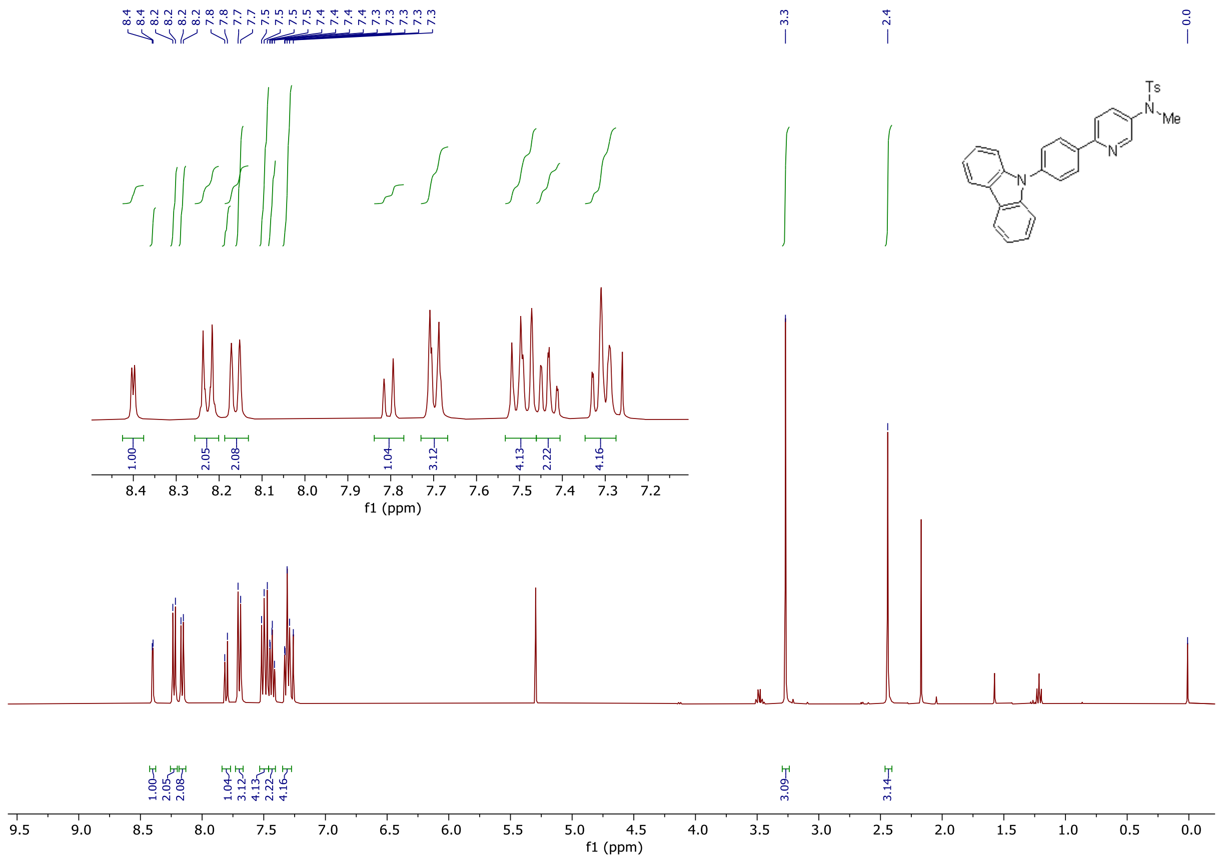

^{13}C NMR spectrum of compound 9a ($CDCl_3$, 298 K)

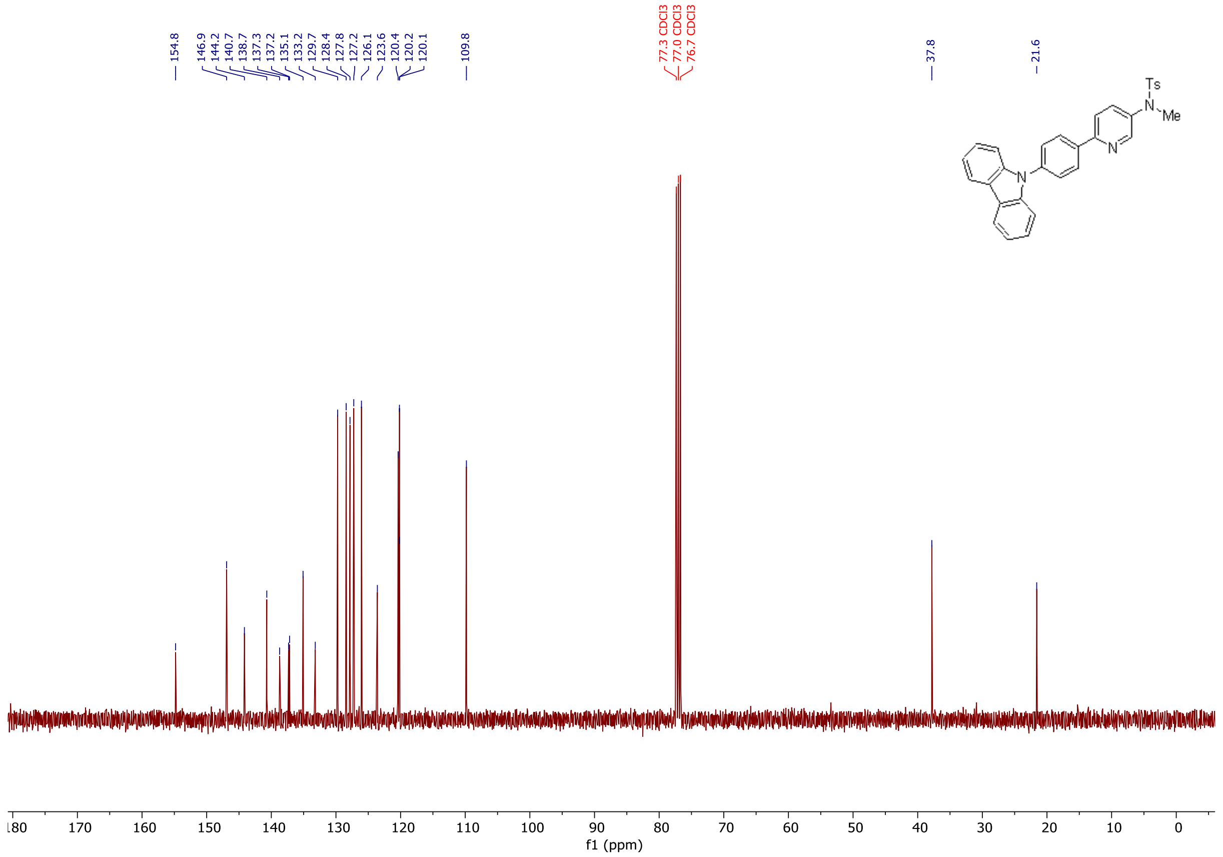

^{1}H NMR spectrum of compound 9b ($CDCl_3$, 298 K)

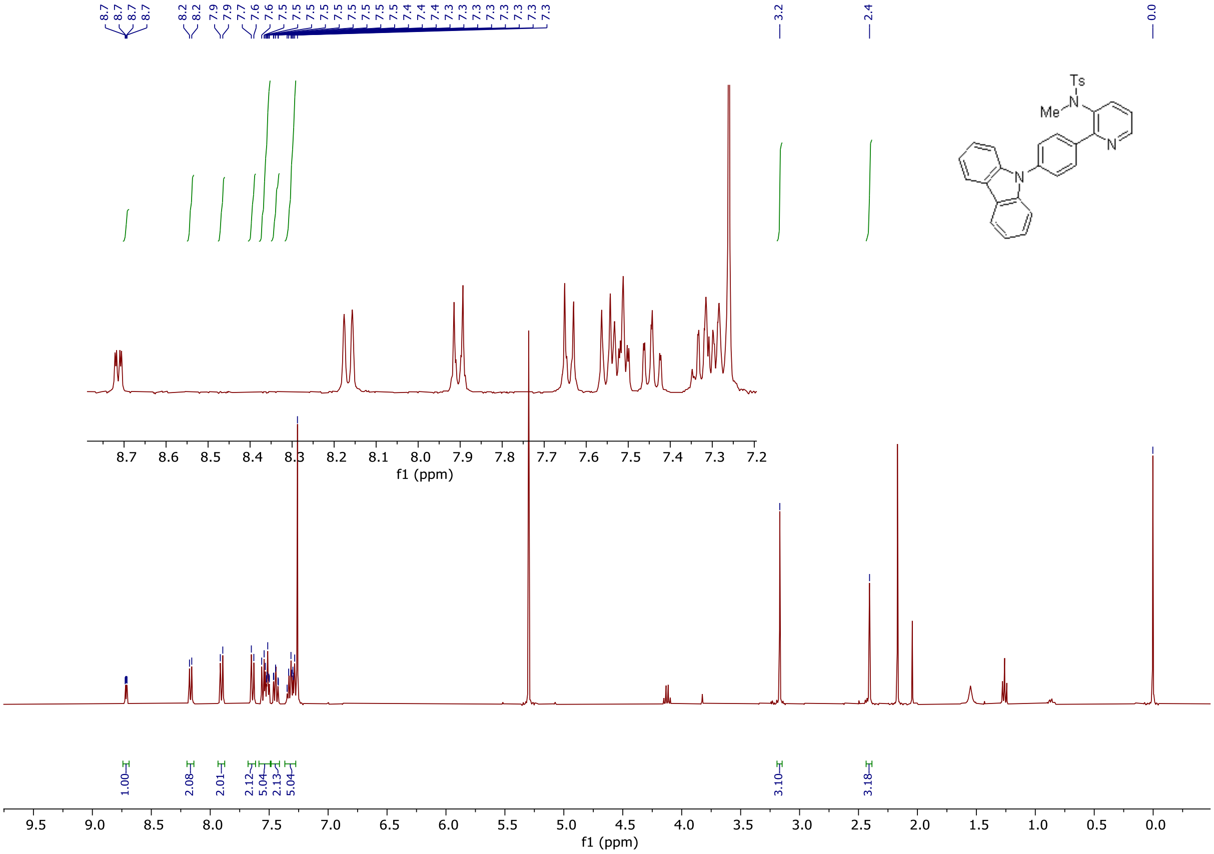

^{13}C NMR spectrum of compound 9b ($CDCl_3$, 298 K)

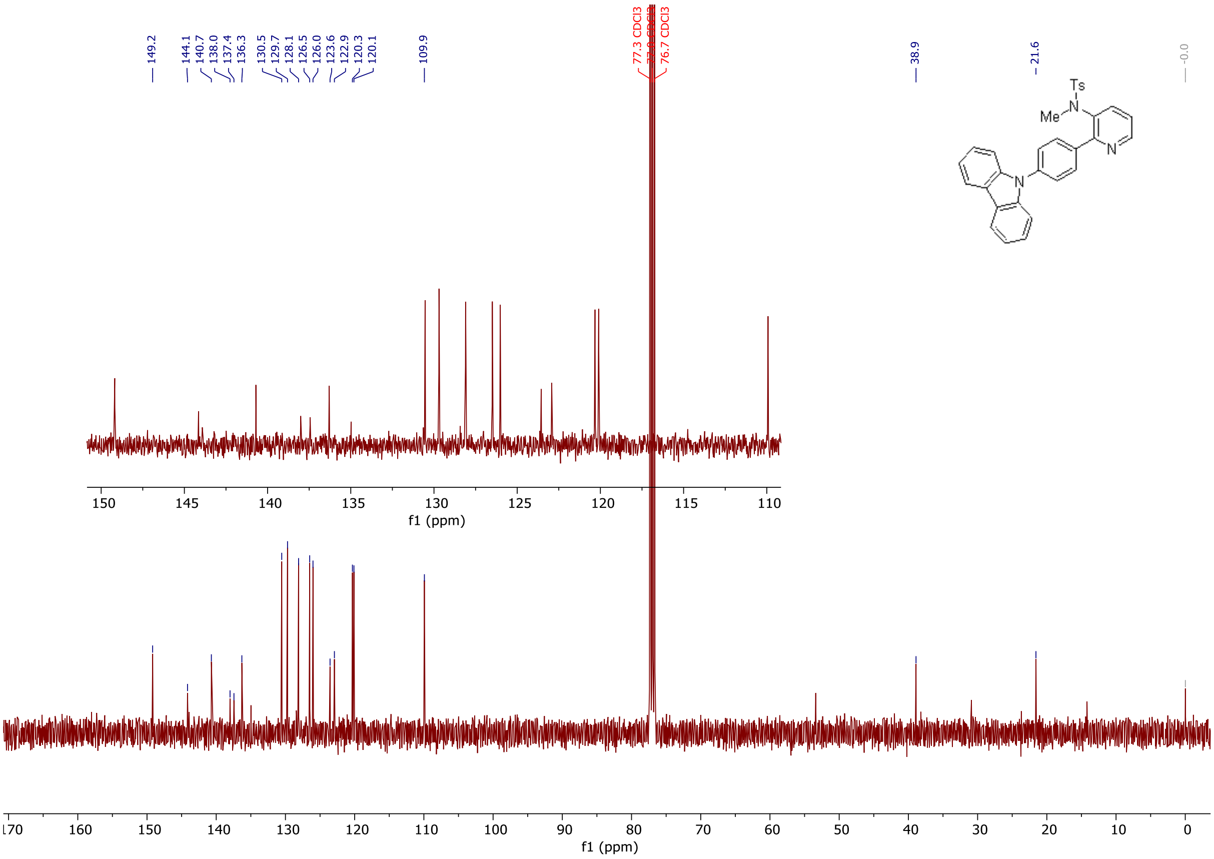

^{1}H NMR spectrum of compound 10a ($CDCl_3$, 298 K)

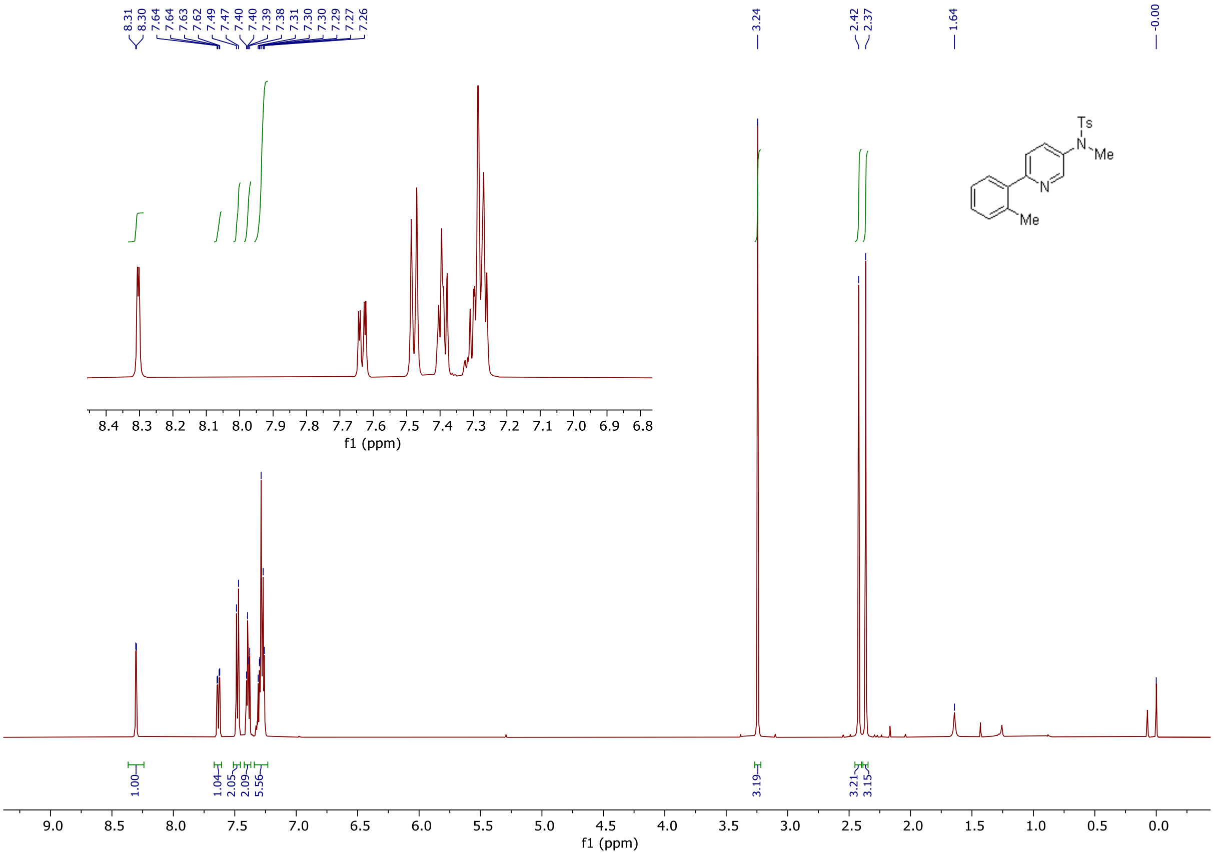

^{13}C NMR spectrum of compound 10a ($CDCl_3$, 298 K)

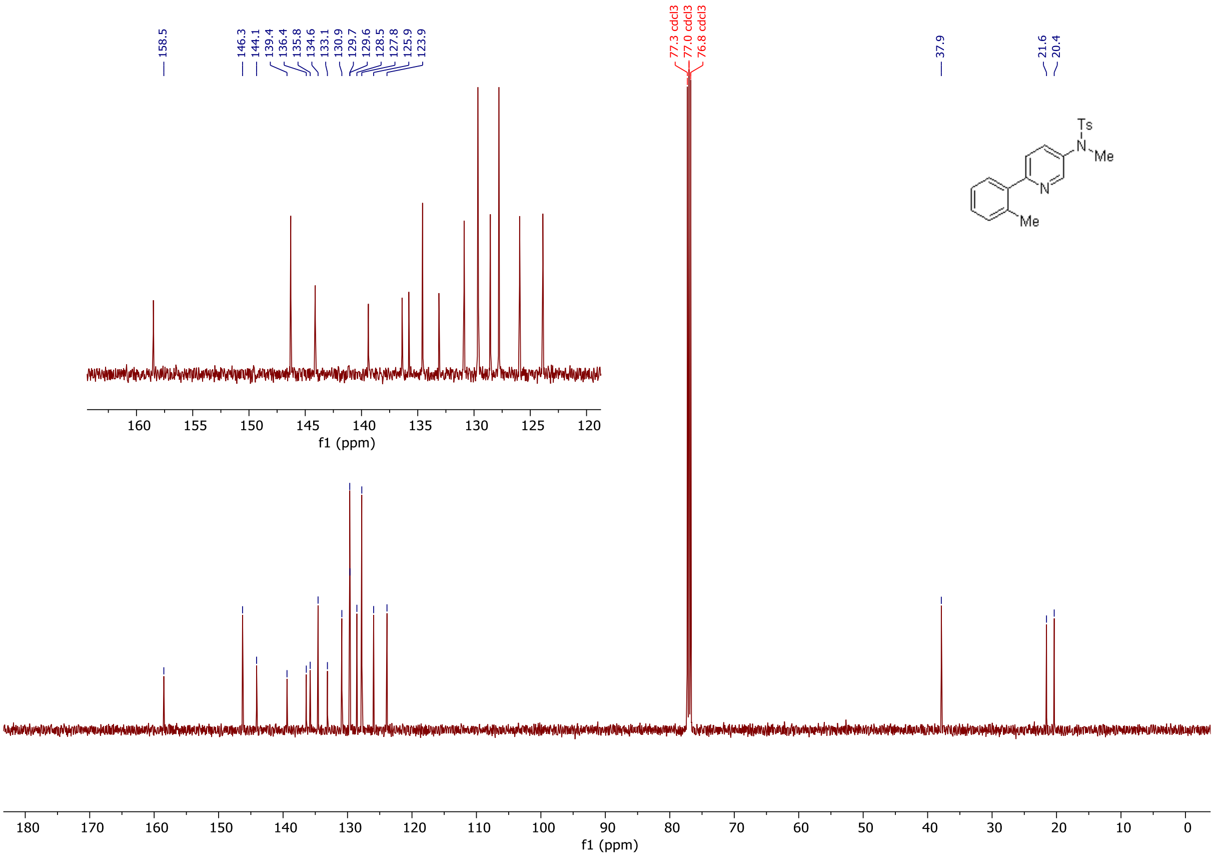

^{1}H NMR spectrum of compound 11a ($CDCl_3$, 298 K)

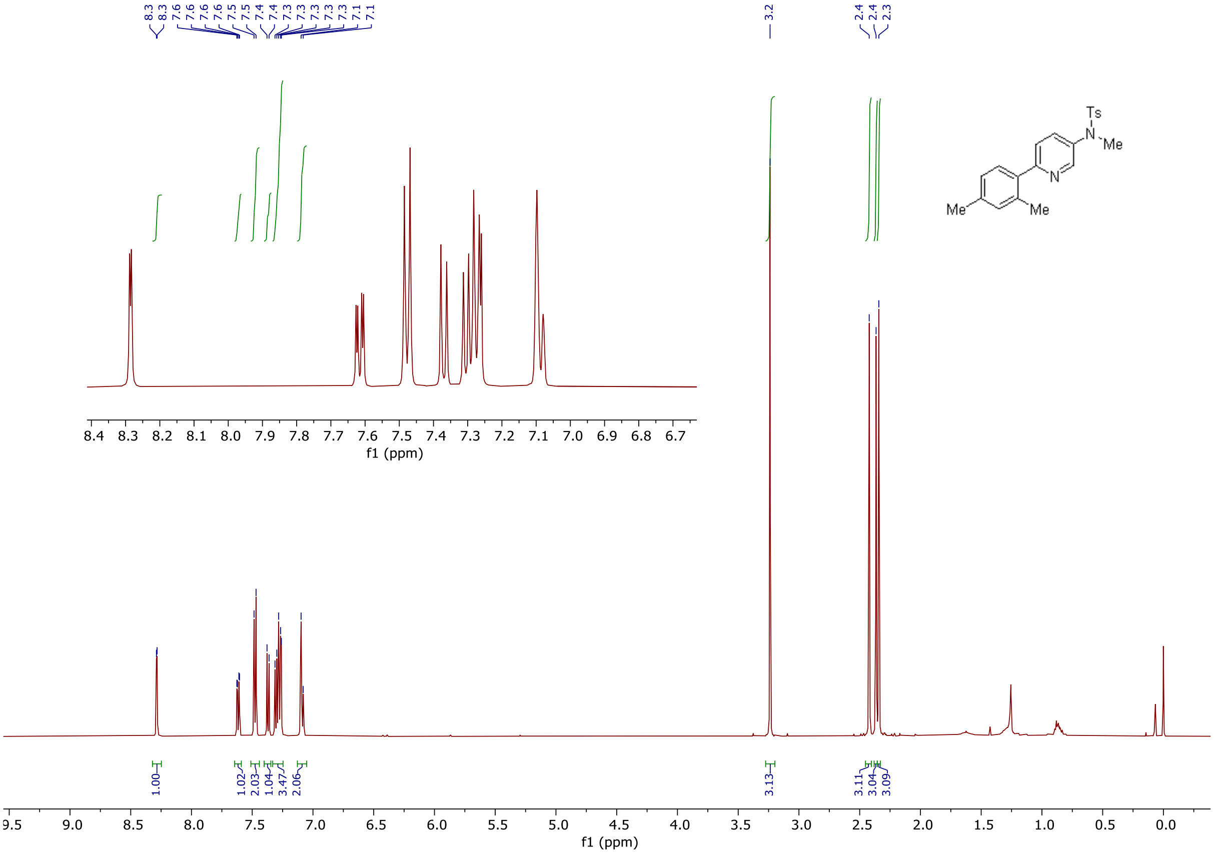

^{13}C NMR spectrum of compound 11a ($CDCl_3$, 298 K)

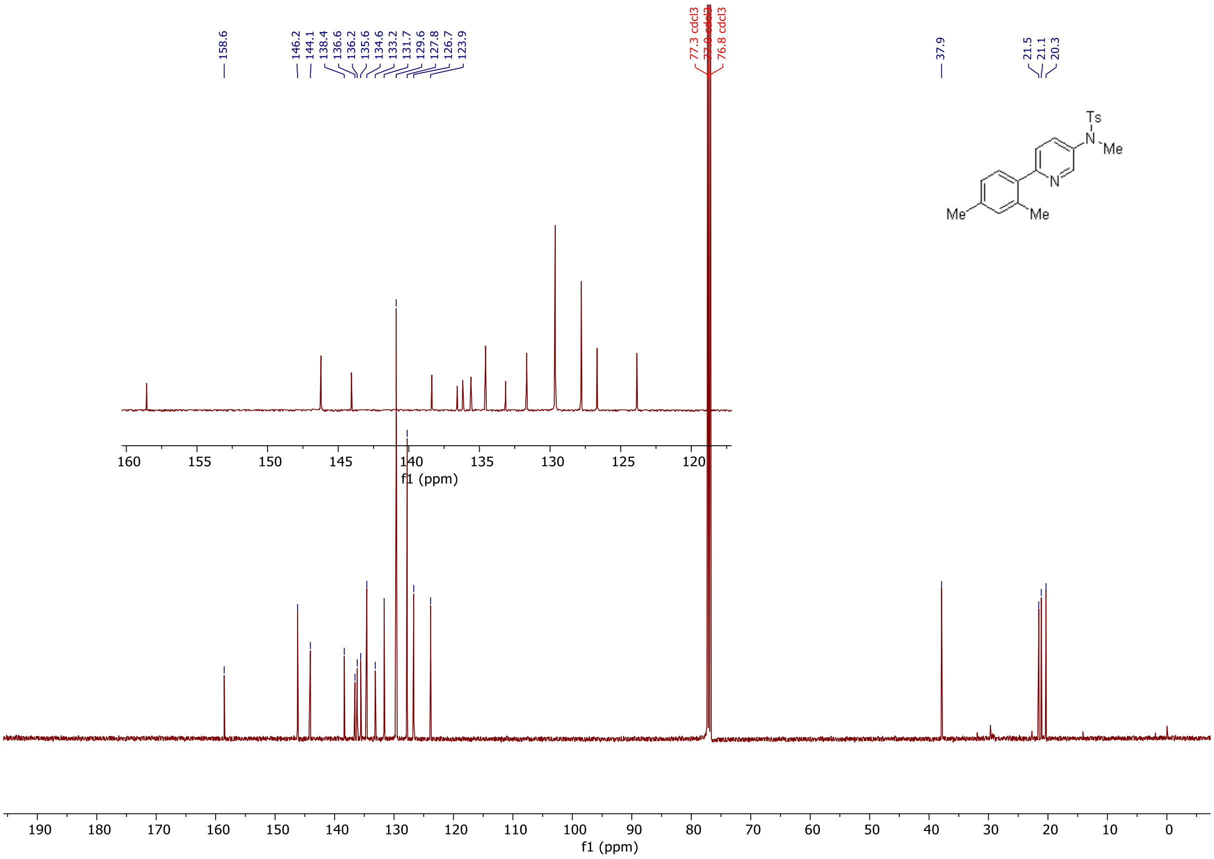

^{1}H NMR spectrum of compound 12a ($CDCl_3$, 298 K)

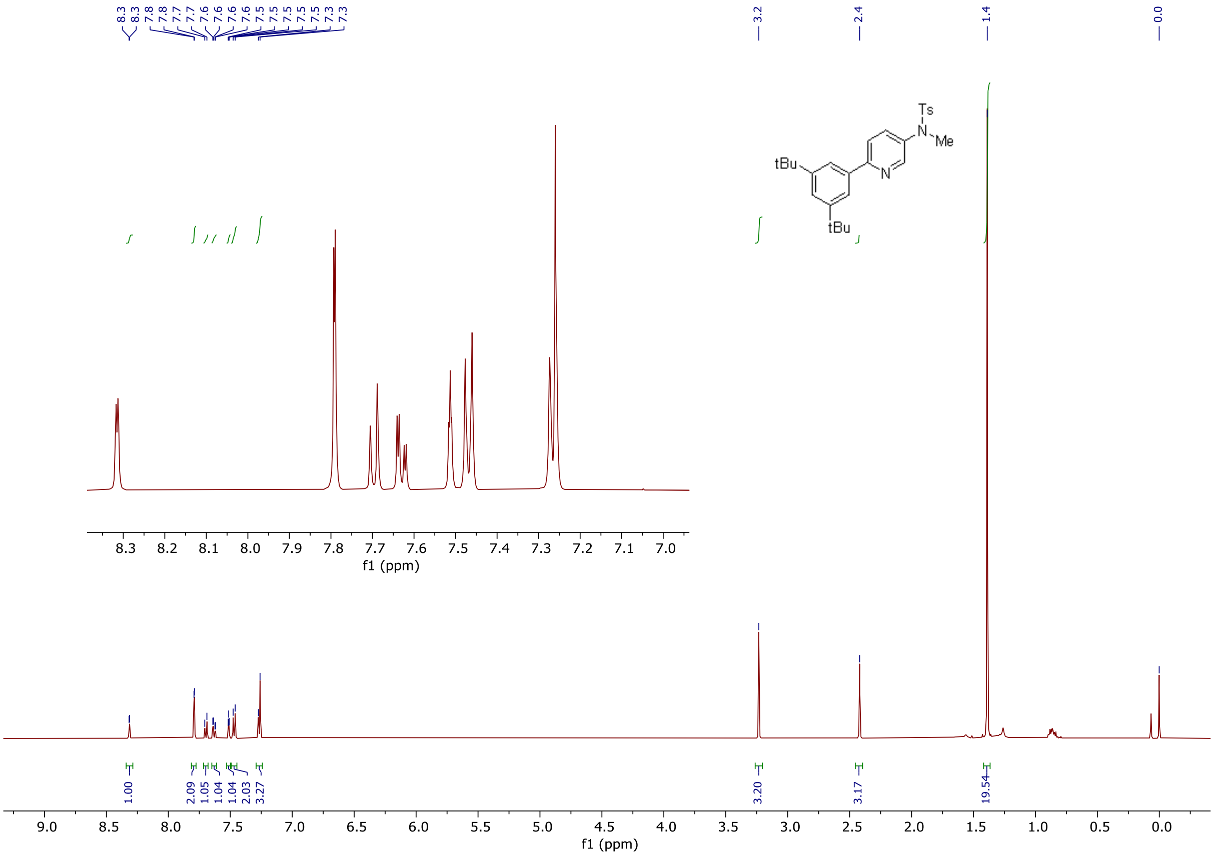

^{13}C NMR spectrum of compound 12a ($CDCl_3$, 298 K)

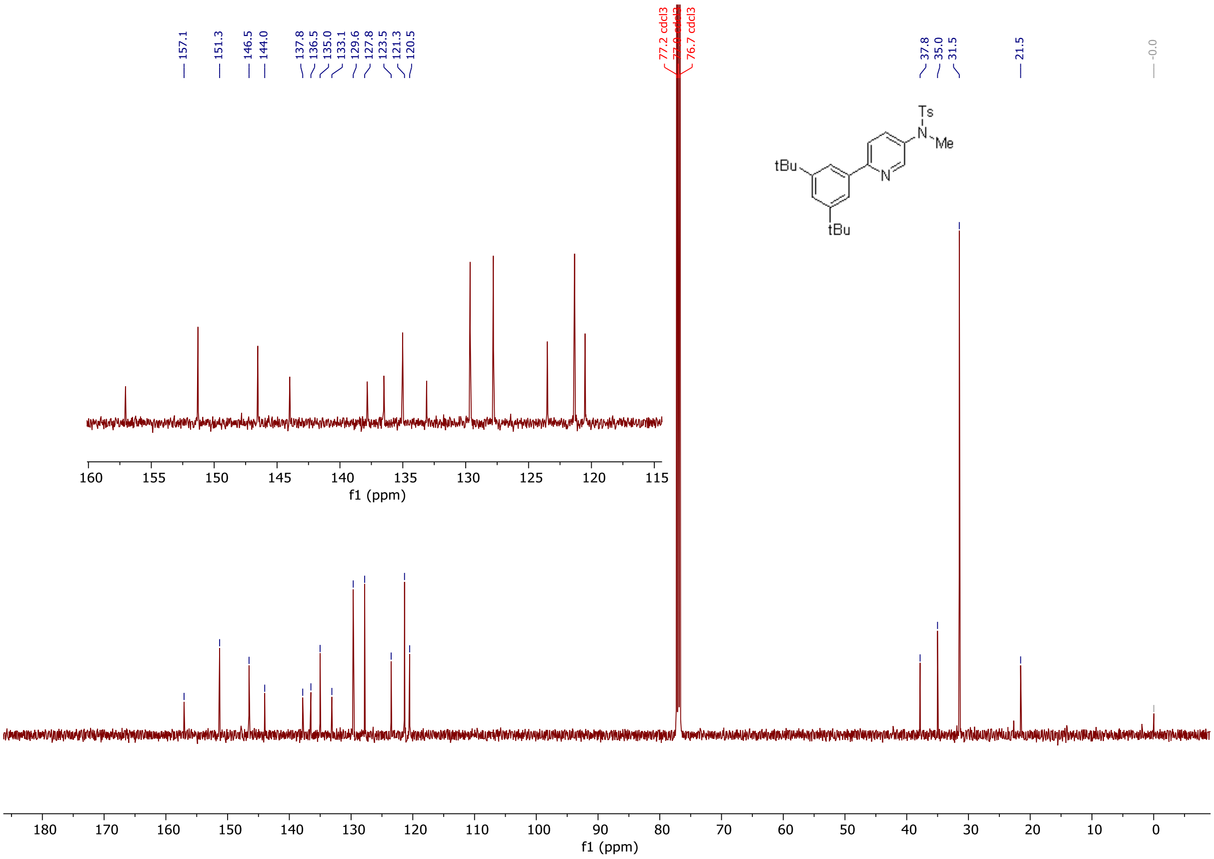

^{1}H NMR spectrum of compound 12b ($CDCl_3$, 298 K)

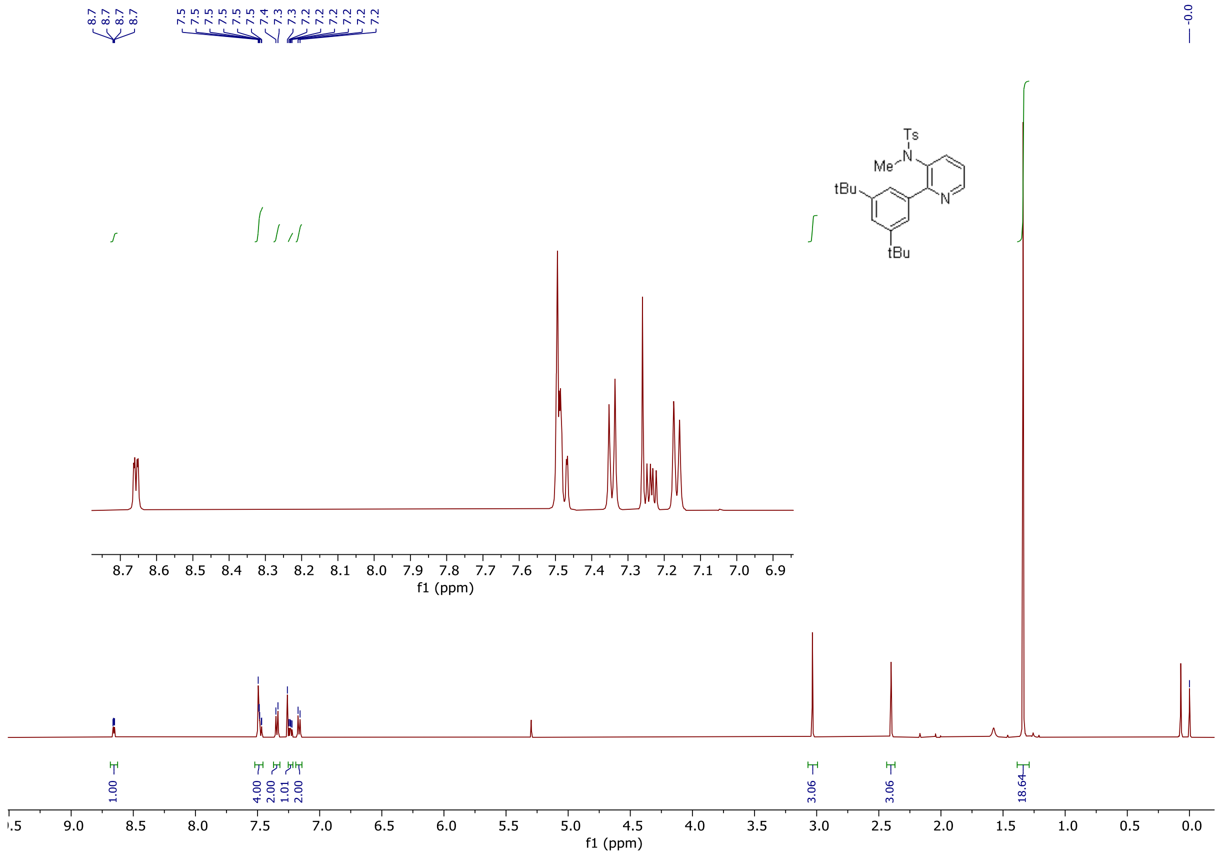

^{13}C NMR spectrum of compound 12b ($CDCl_3$, 298 K)

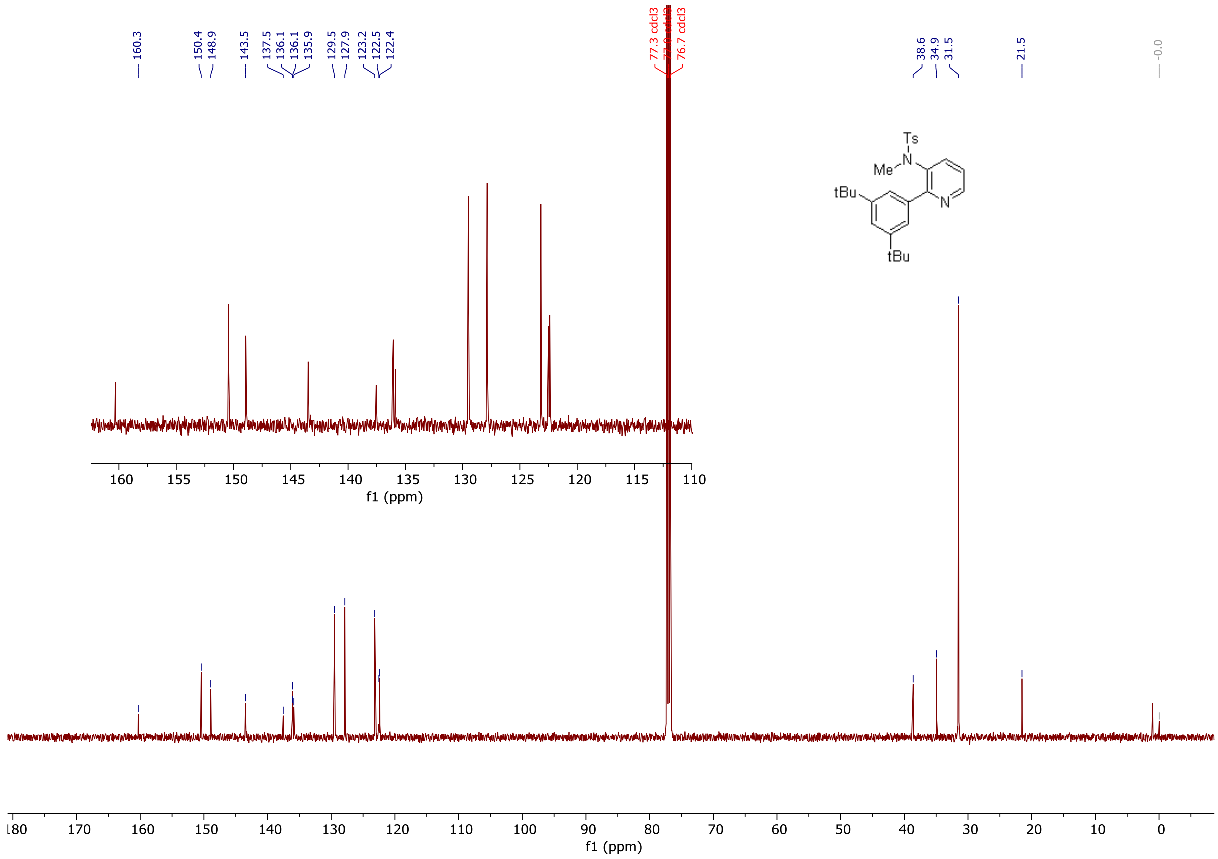

^{1}H NMR spectrum of compound 13a ($CDCl_3$, 298 K)

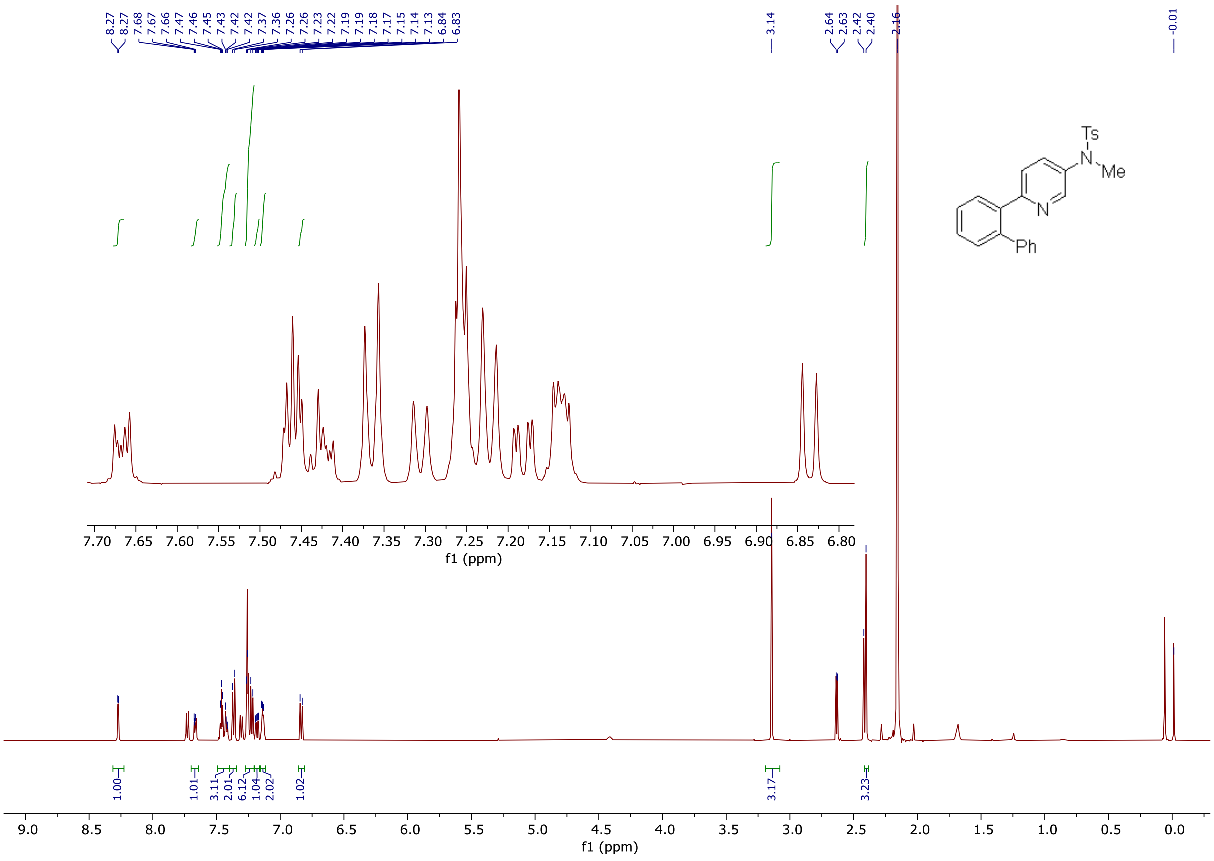

^{13}C NMR spectrum of compound 13a ($CDCl_3$, 298 K)

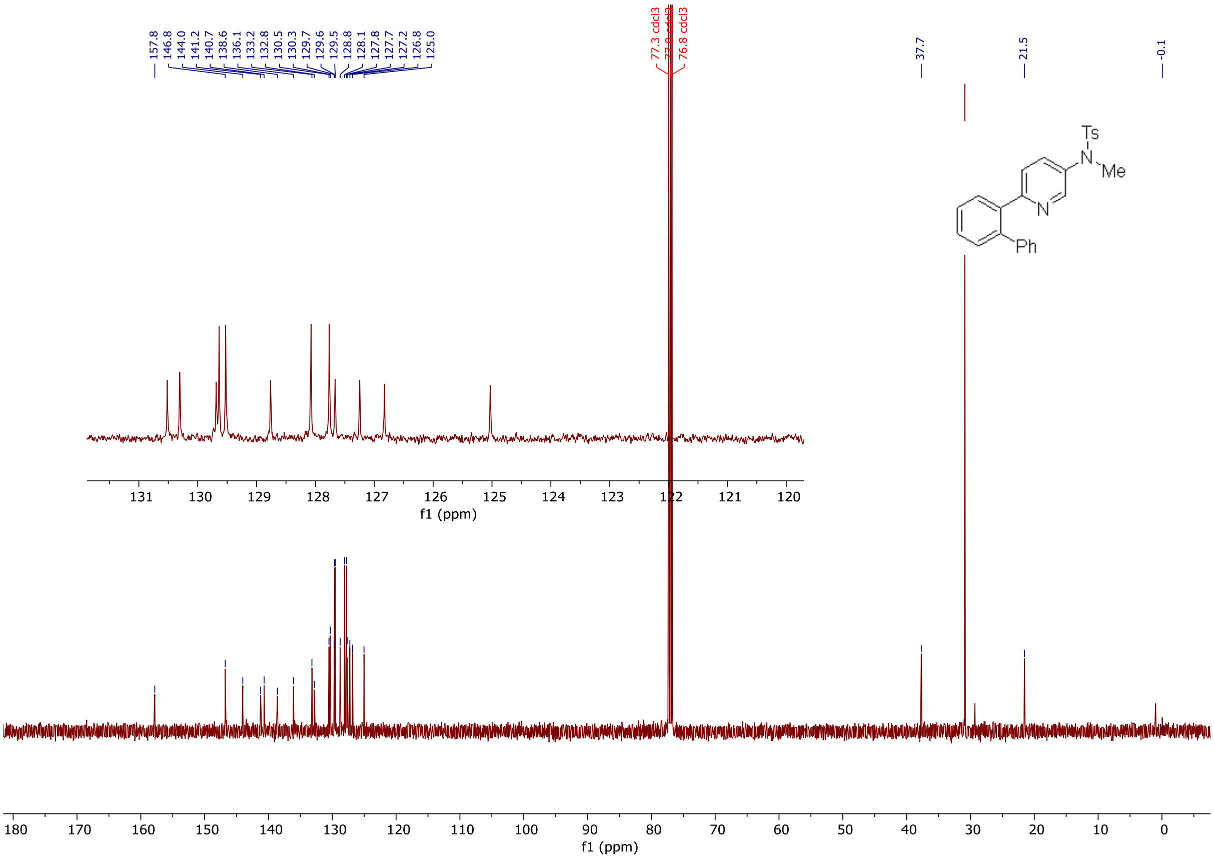

^{1}H NMR spectrum of compound 14a ($CDCl_3$, 298 K)

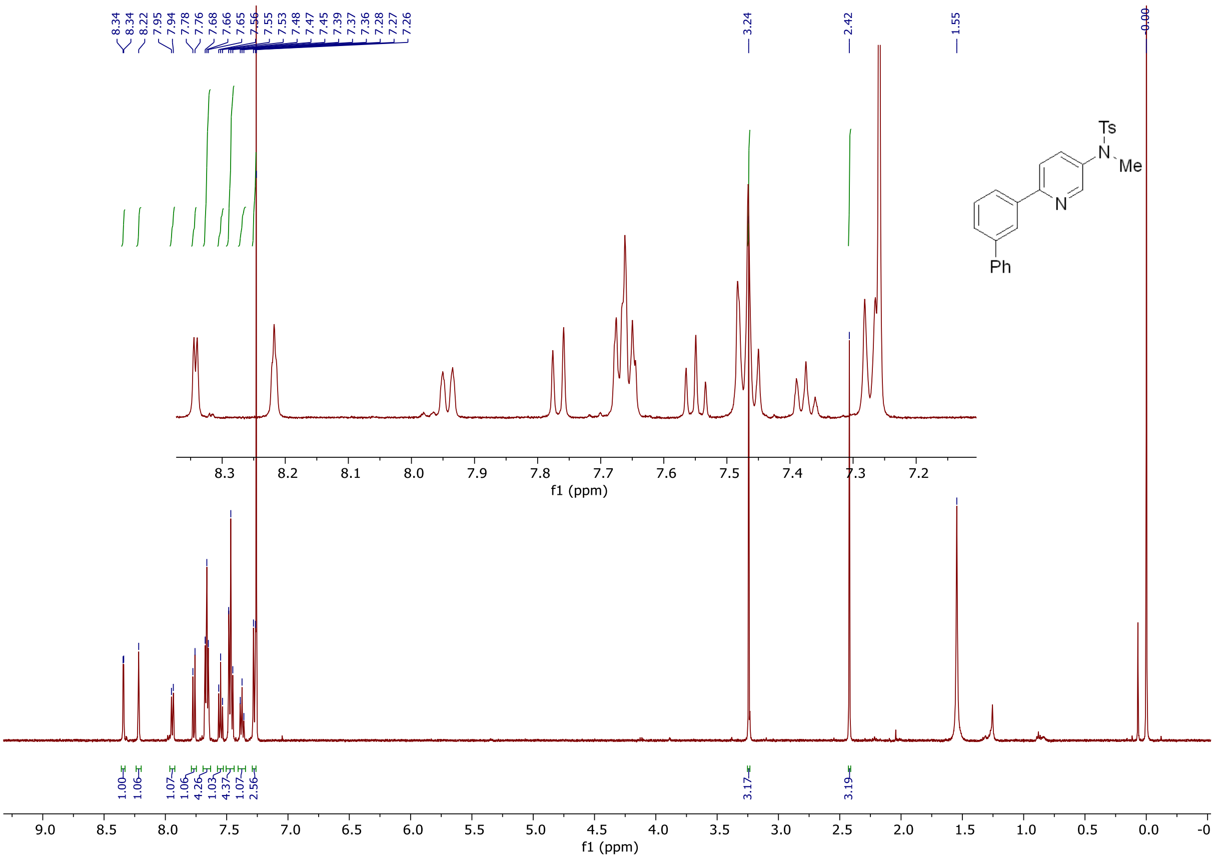

^{13}C NMR spectrum of compound 14a ($CDCl_3$, 298 K)

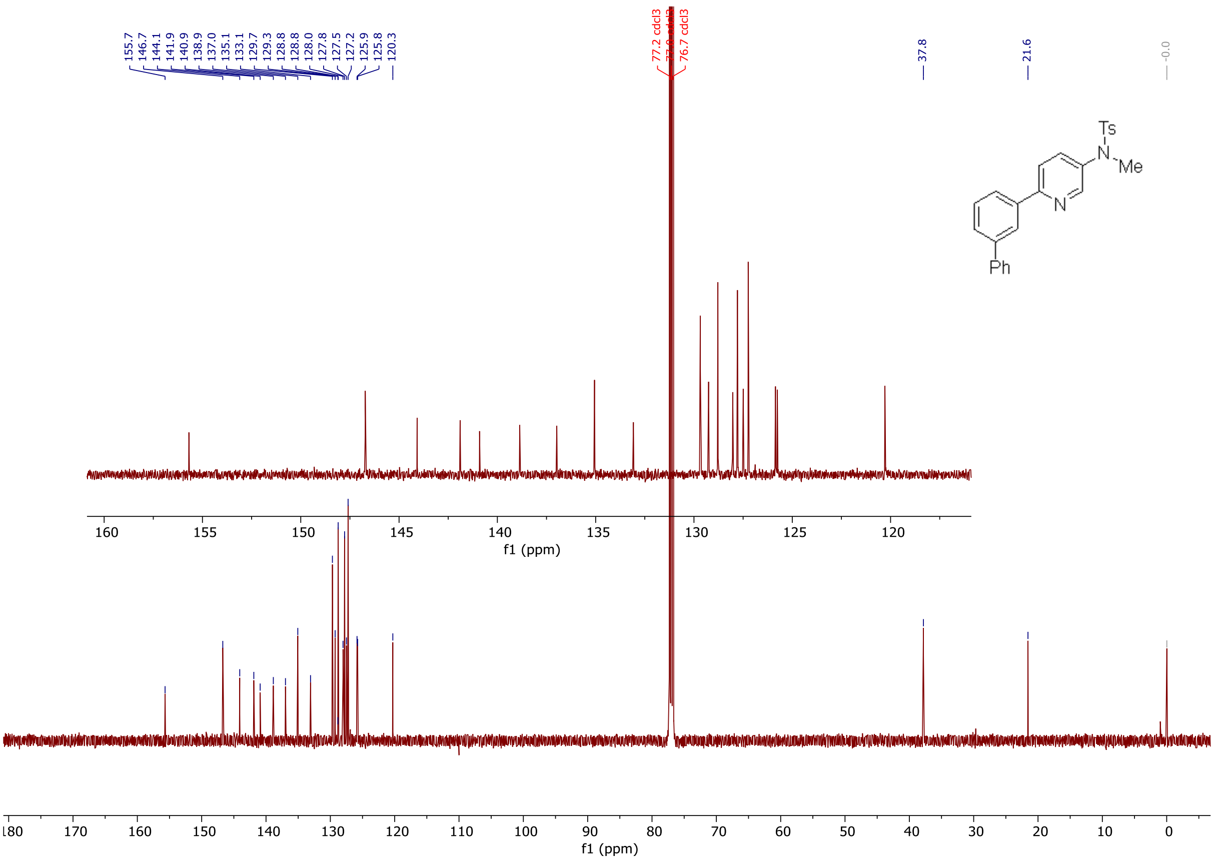

^{1}H NMR spectrum of compound 14b ($CDCl_3$, 298 K)

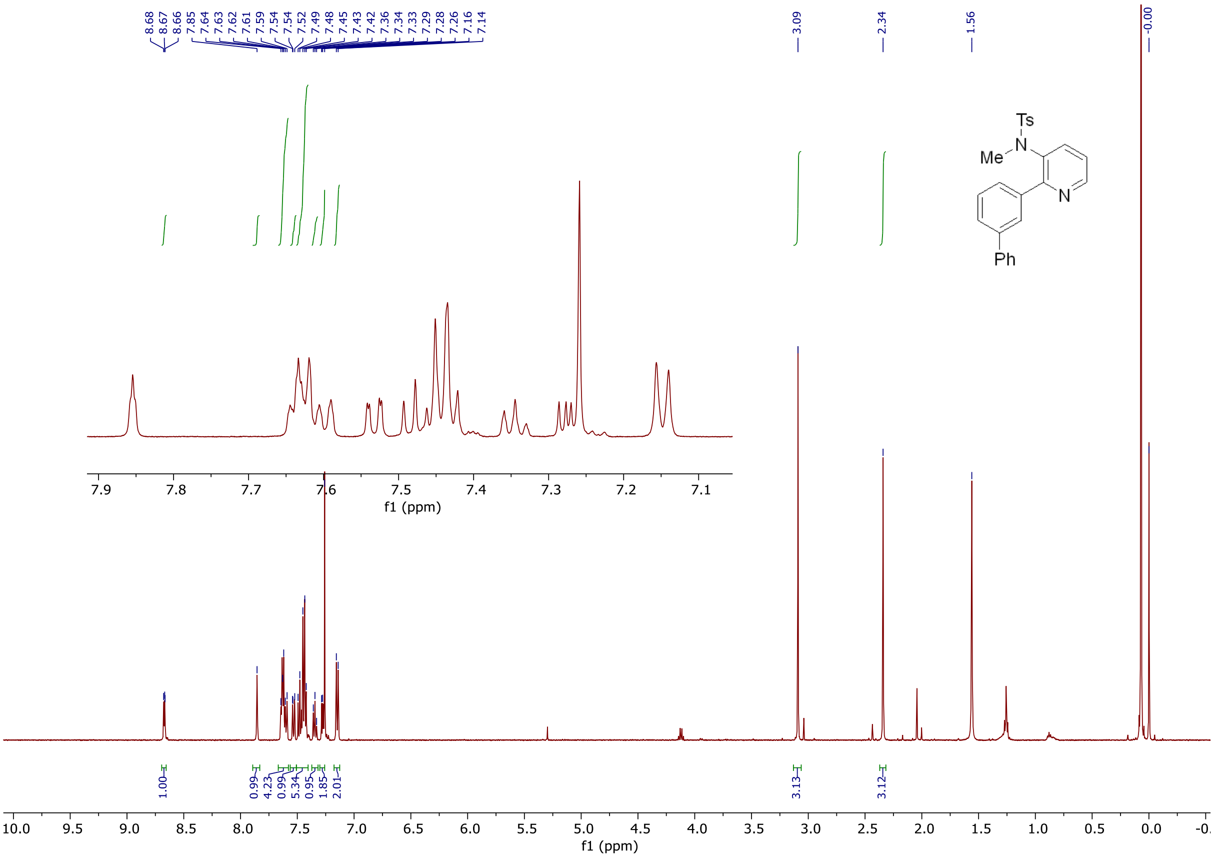

^{13}C NMR spectrum of compound 14b ($CDCl_3$, 298 K)

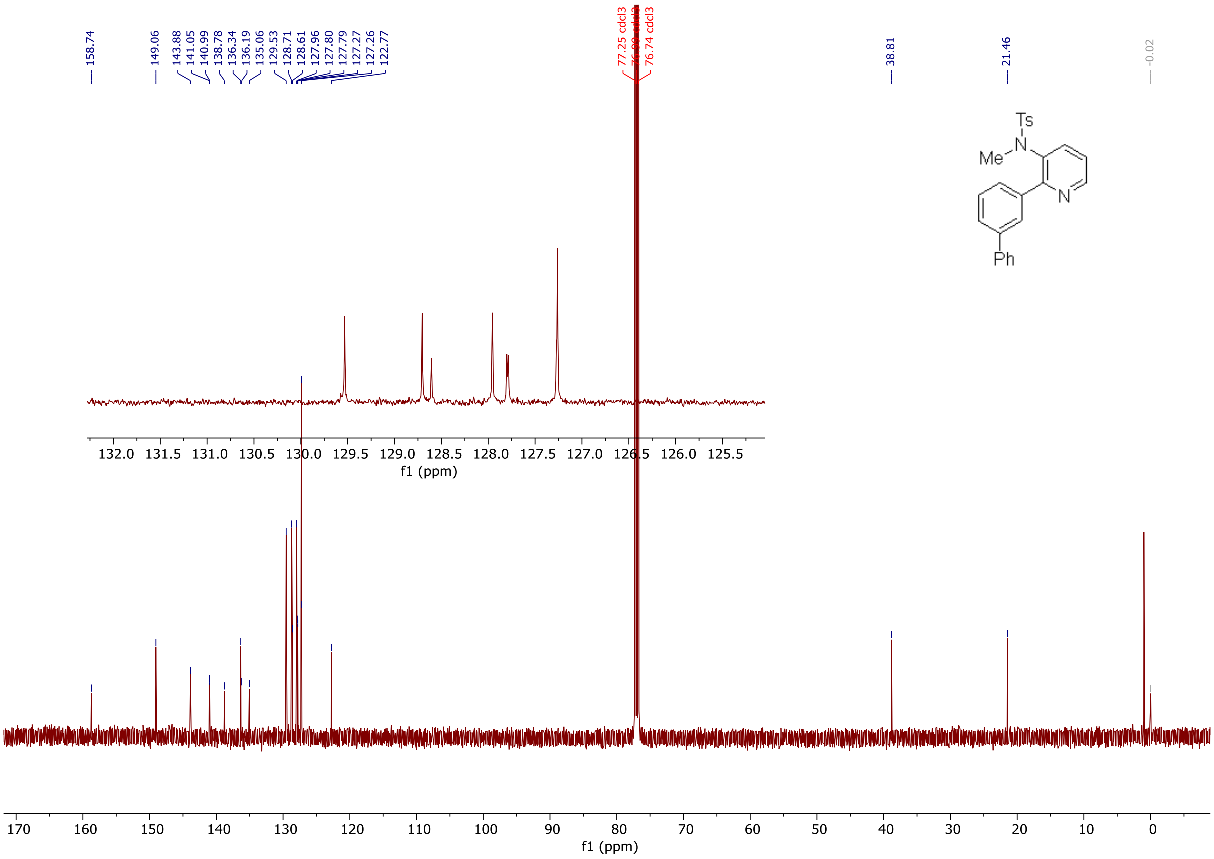

^{1}H NMR spectrum of compound 15a ($CDCl_3$, 298 K)

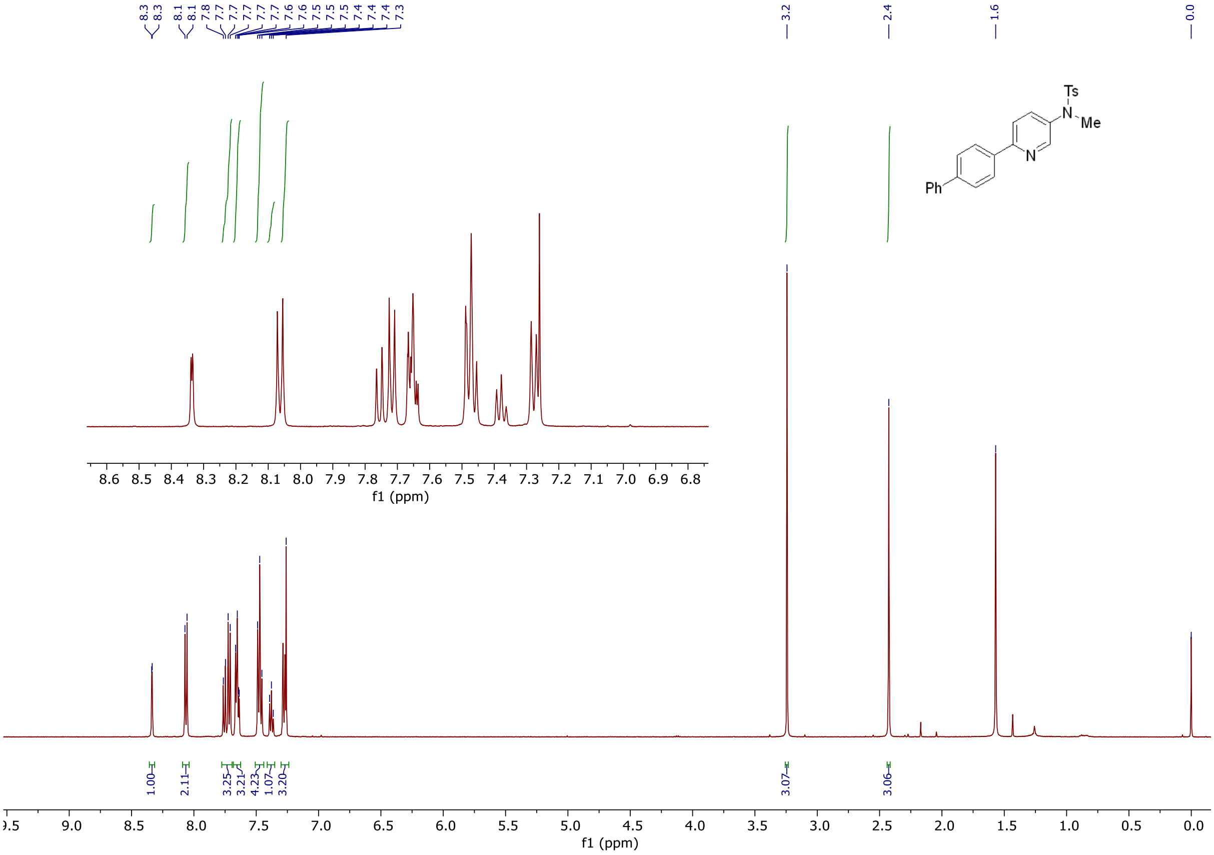

^{13}C NMR spectrum of compound 15a ($CDCl_3$, 298 K)

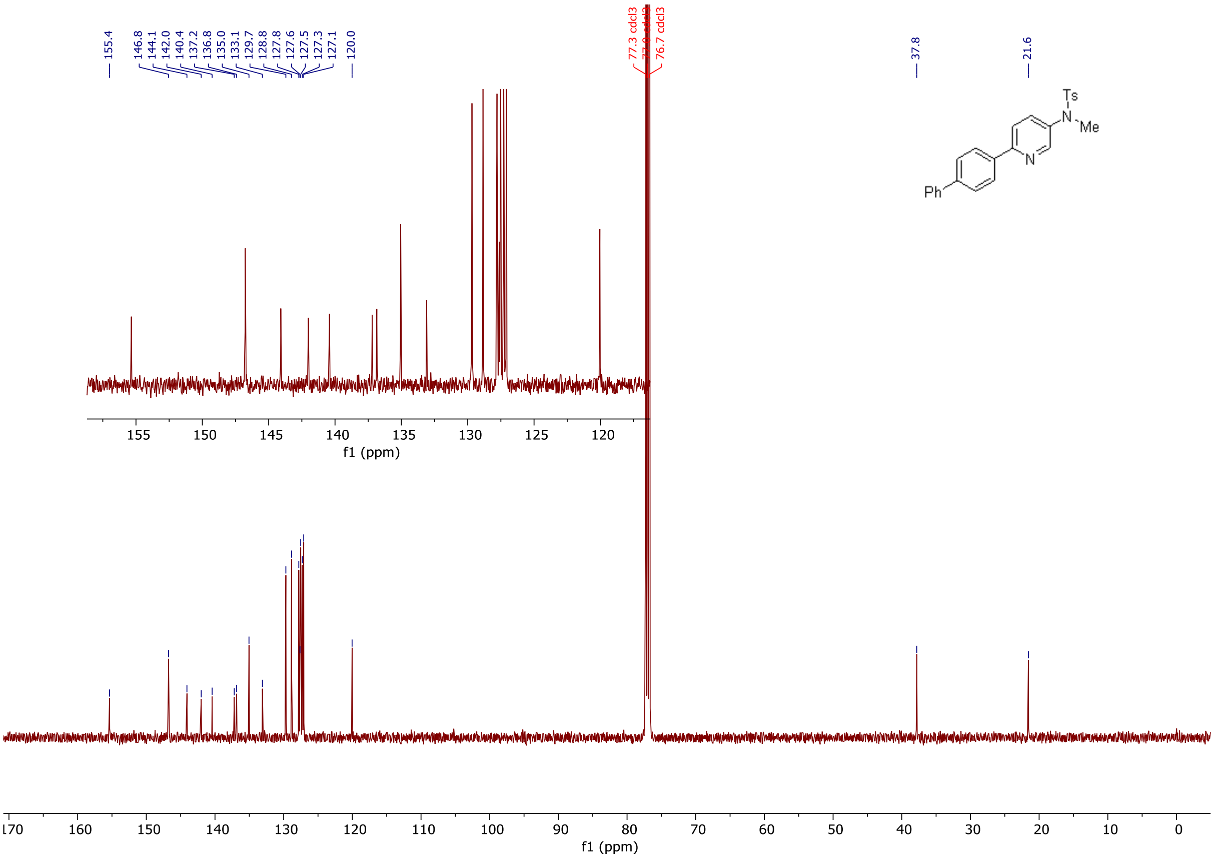

^{1}H NMR spectrum of compound 15b ($CDCl_3$, 298 K)

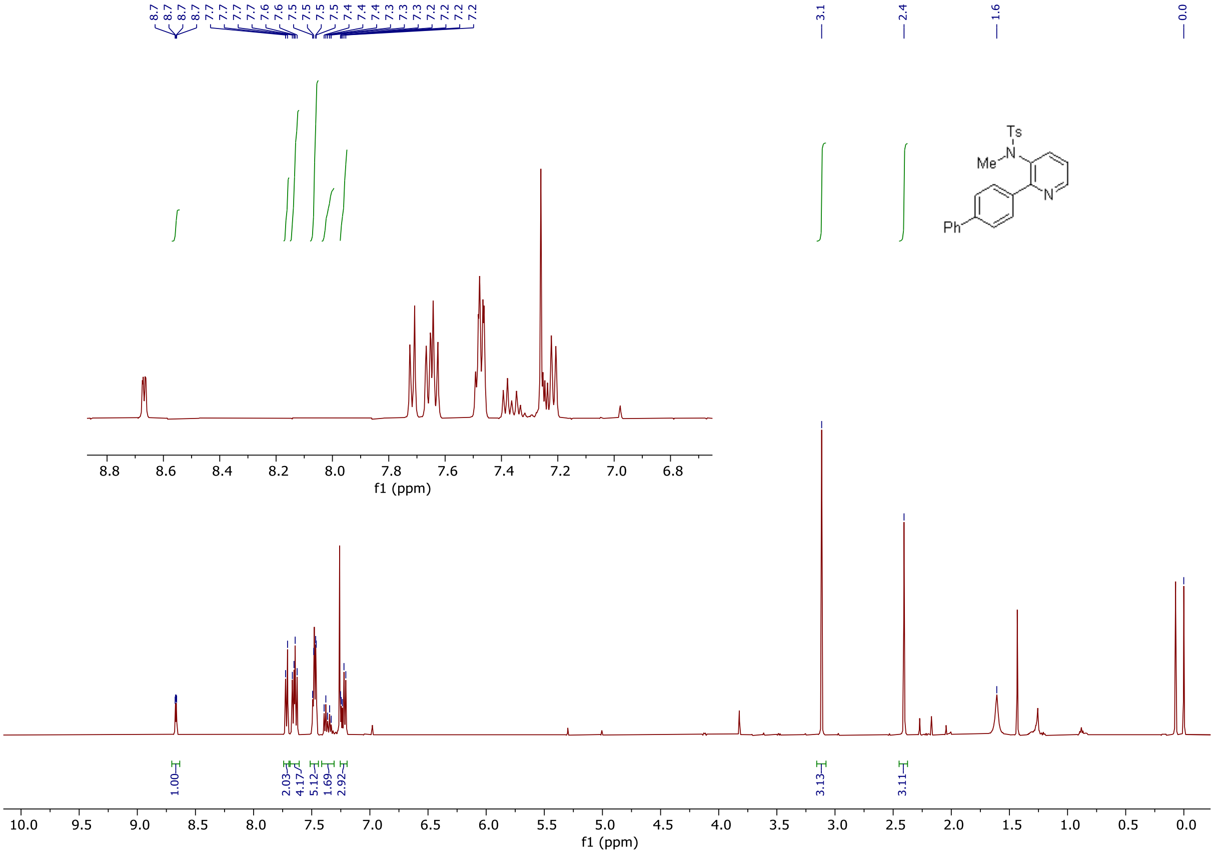

^{13}C NMR spectrum of compound 15b ($CDCl_3$, 298 K)

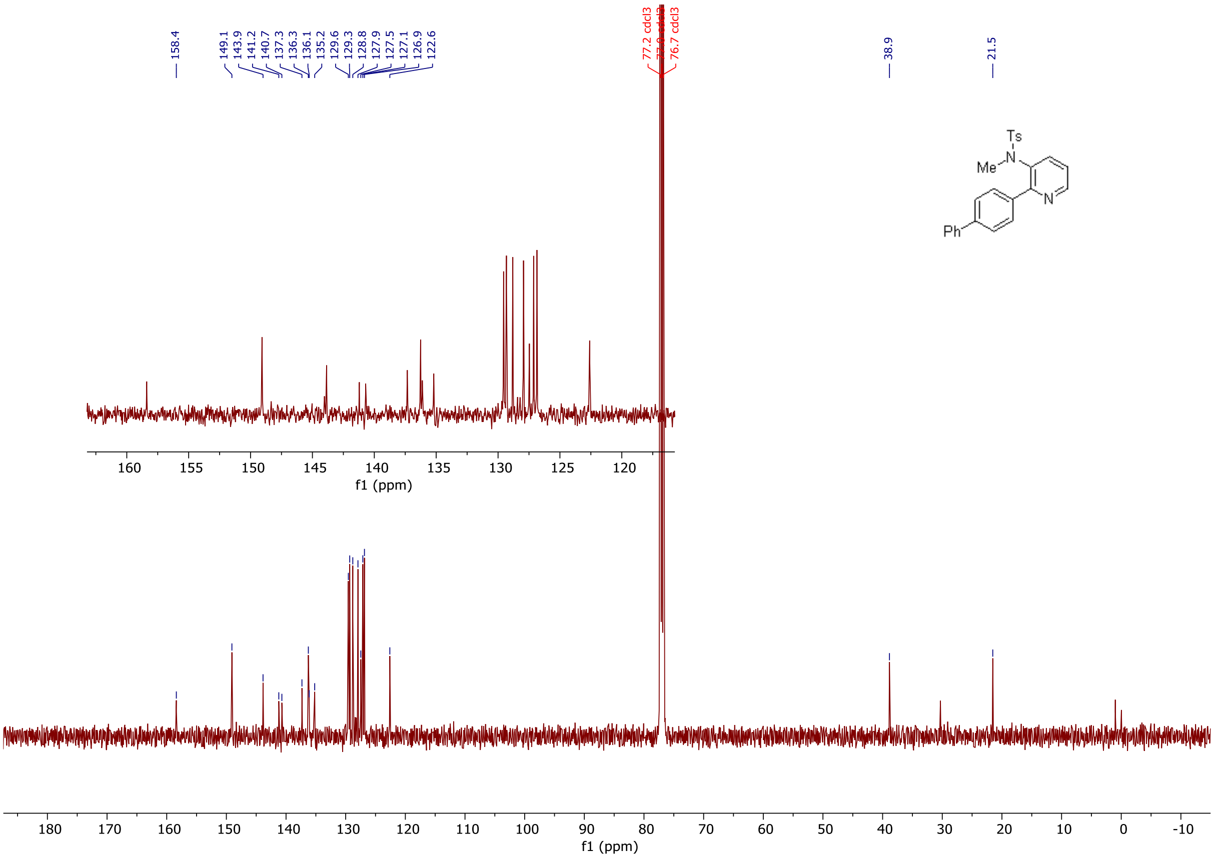

^{1}H NMR spectrum of compound 16a ($CDCl_3$, 298 K)

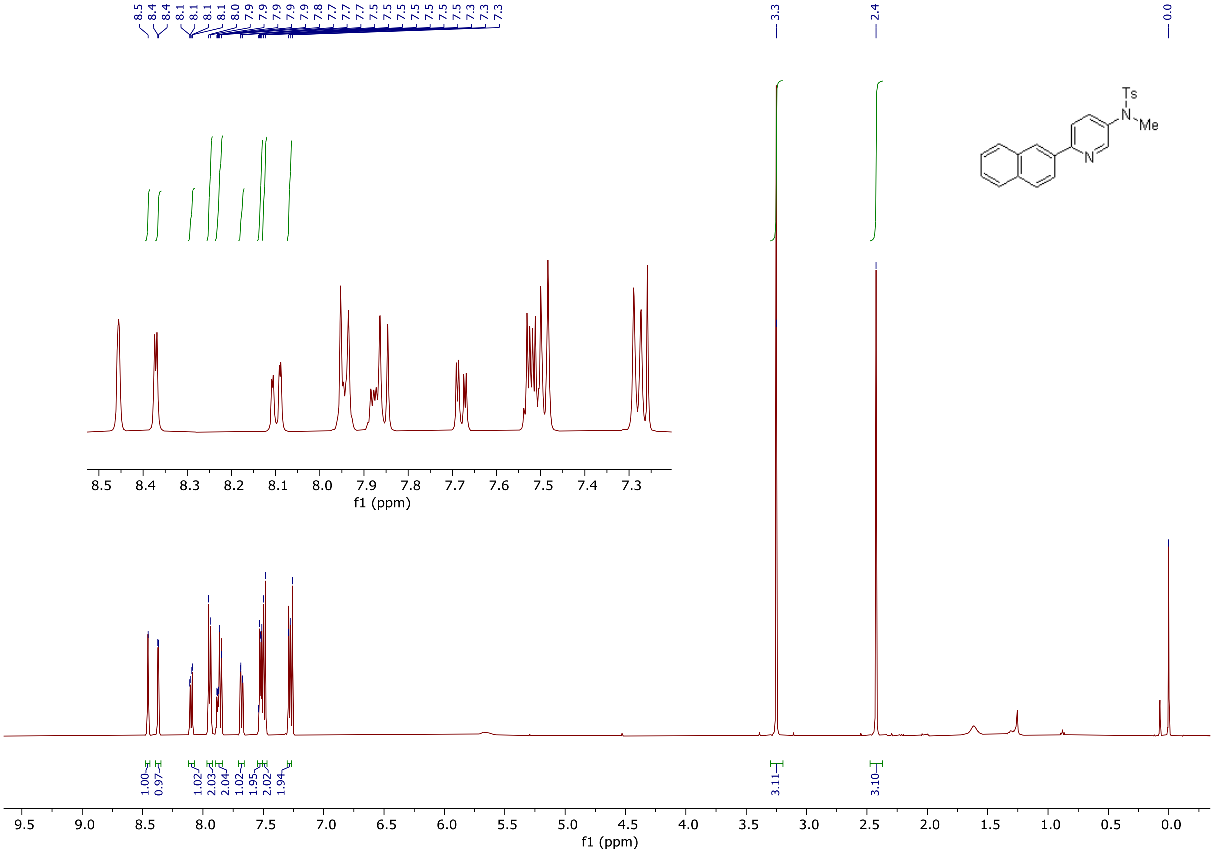

^{13}C NMR spectrum of compound 16a ($CDCl_3$, 298 K)

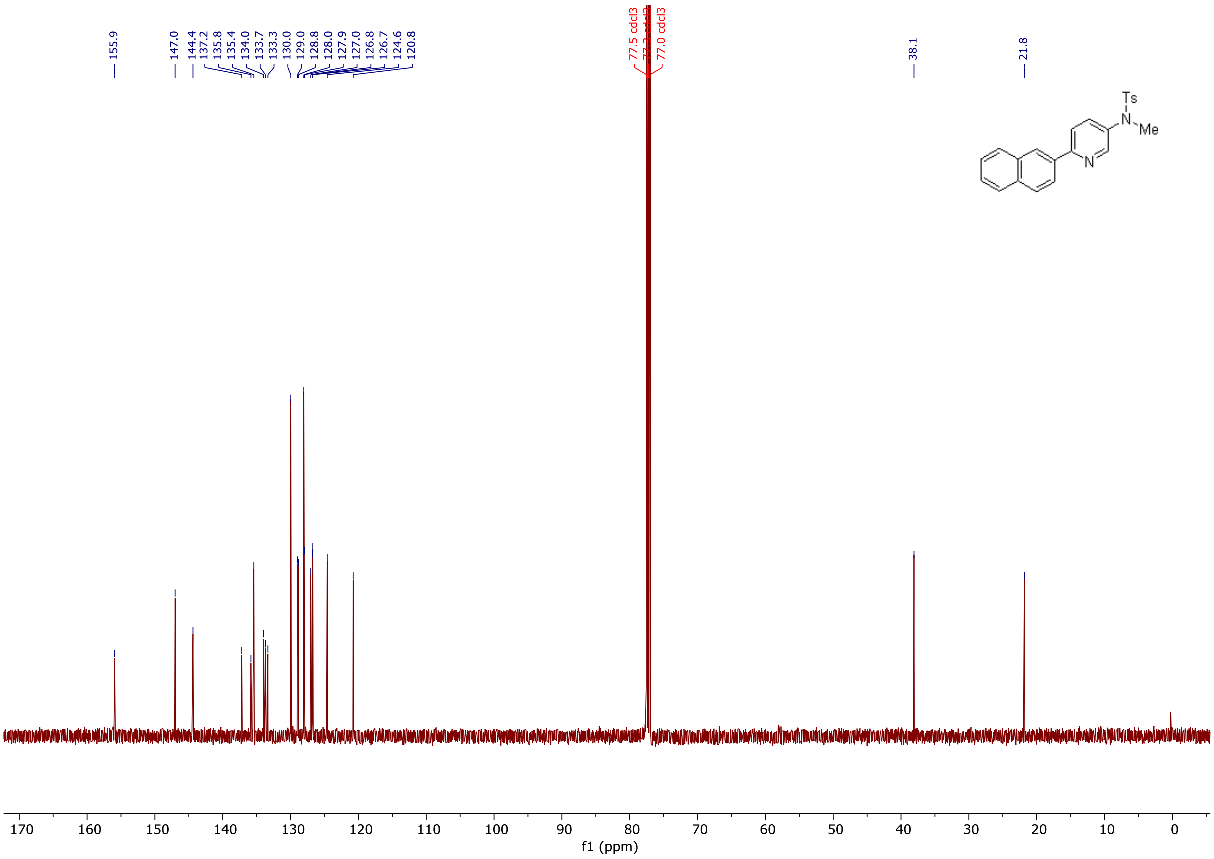

^{1}H NMR spectrum of compound 17a ($CDCl_3$, 298 K)

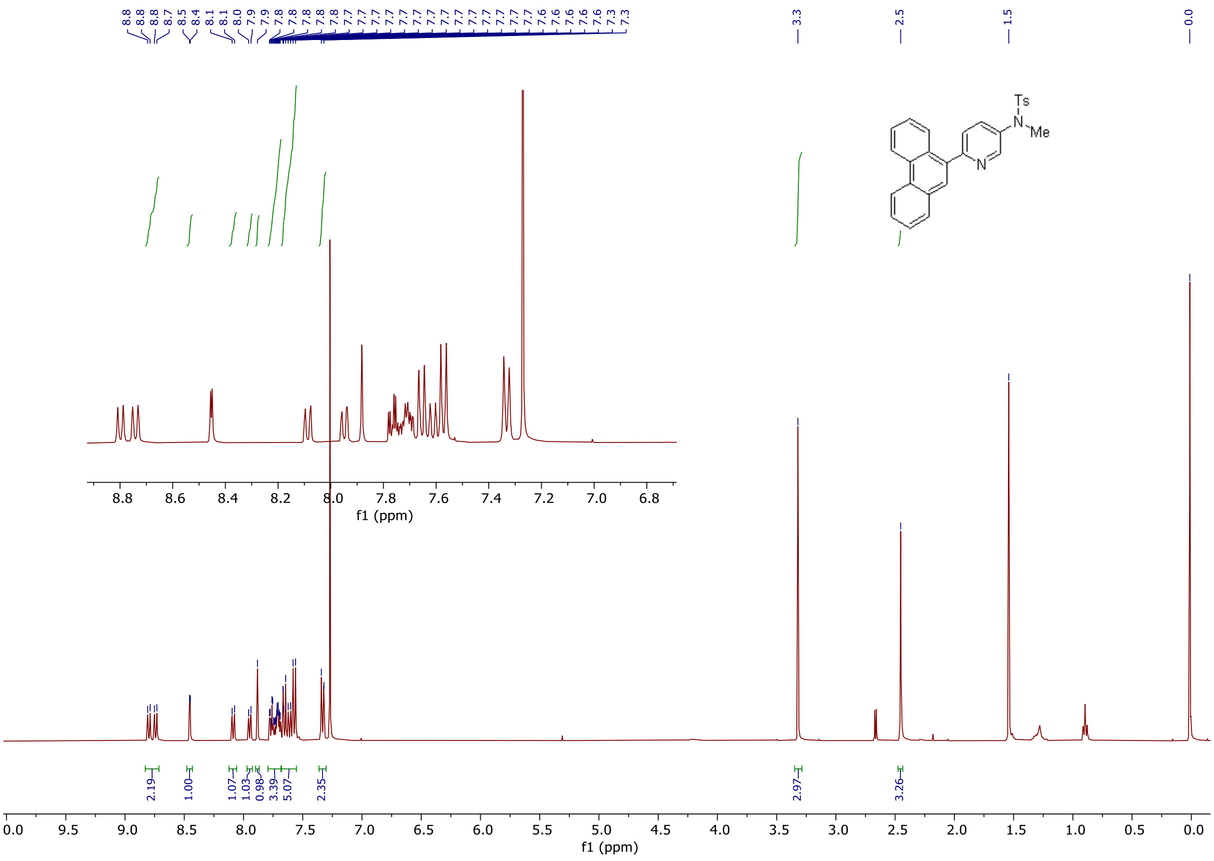

^{13}C NMR spectrum of compound 17a ($CDCl_3$, 298 K)

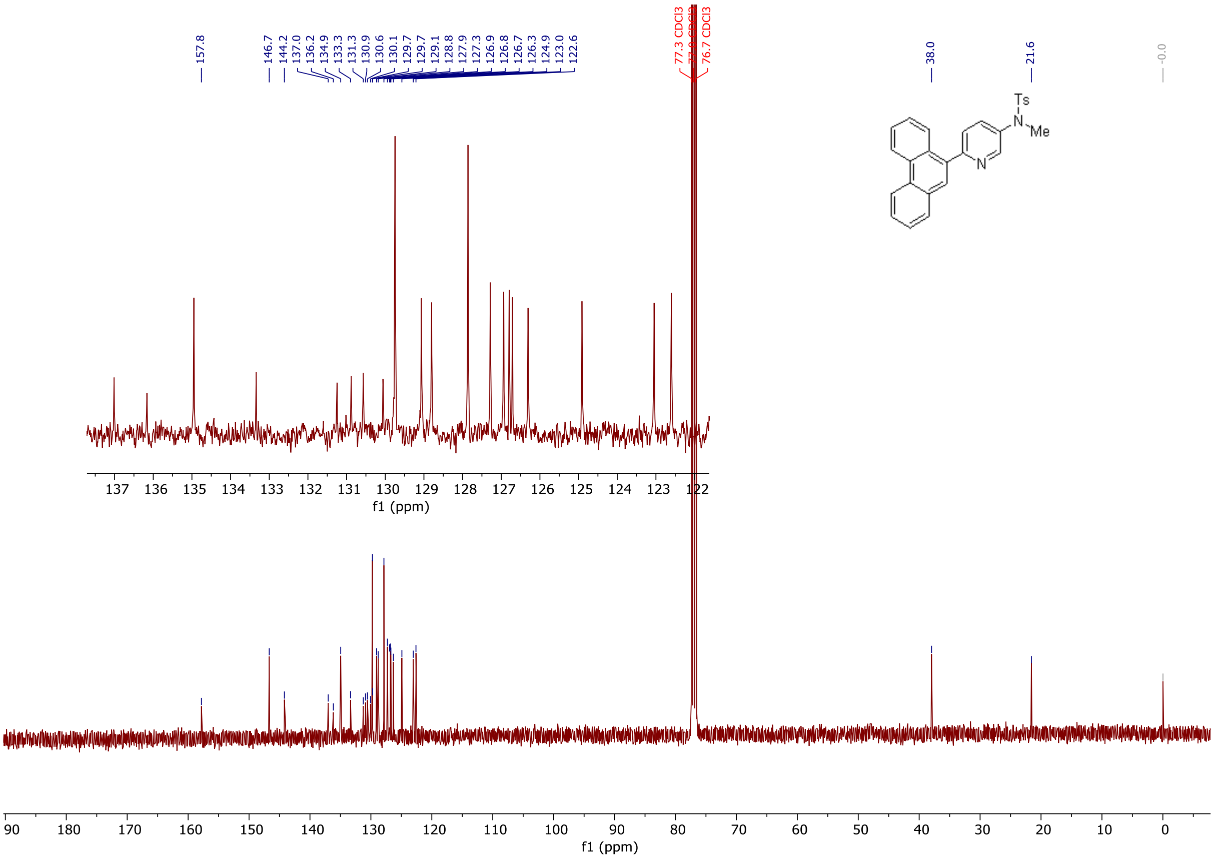

^{1}H NMR spectrum of compound 18a ($CDCl_3$, 298 K)

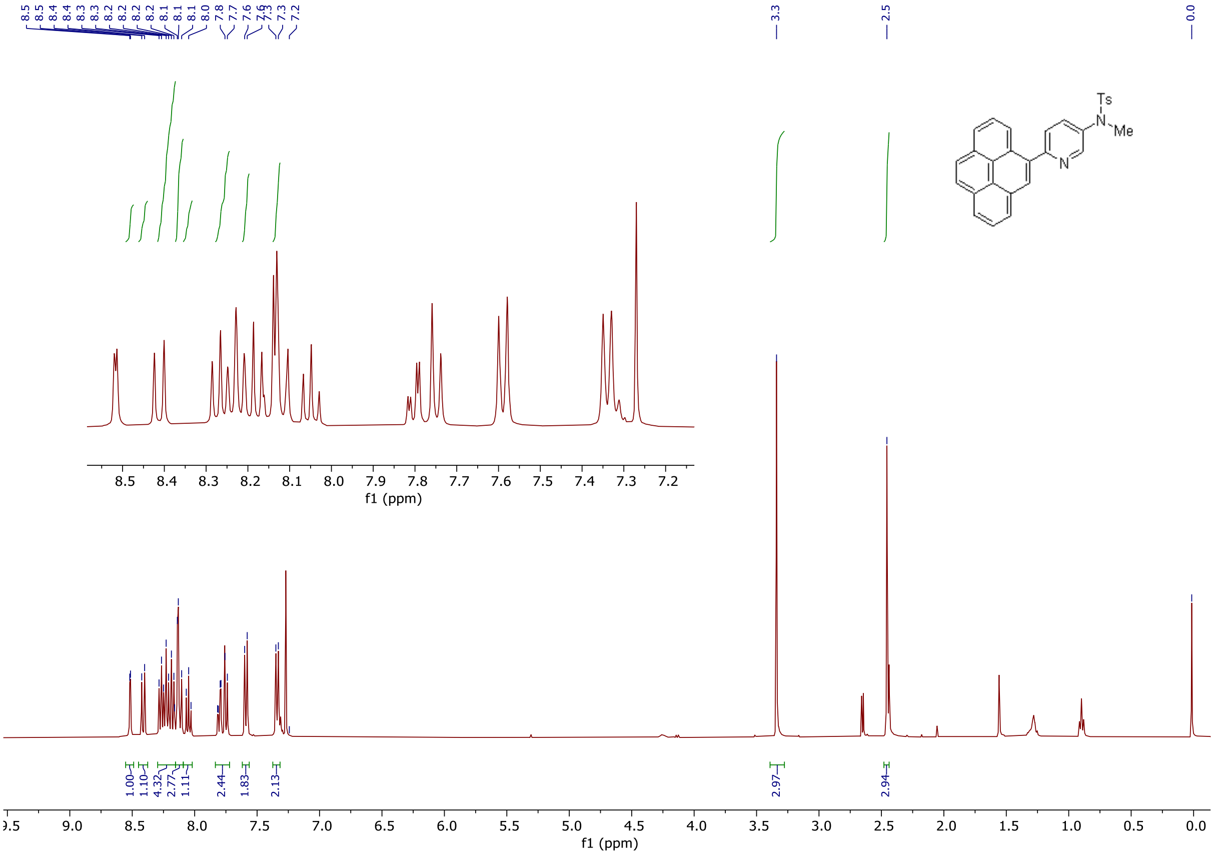

^{13}C NMR spectrum of compound 18a ($CDCl_3$, 298 K)

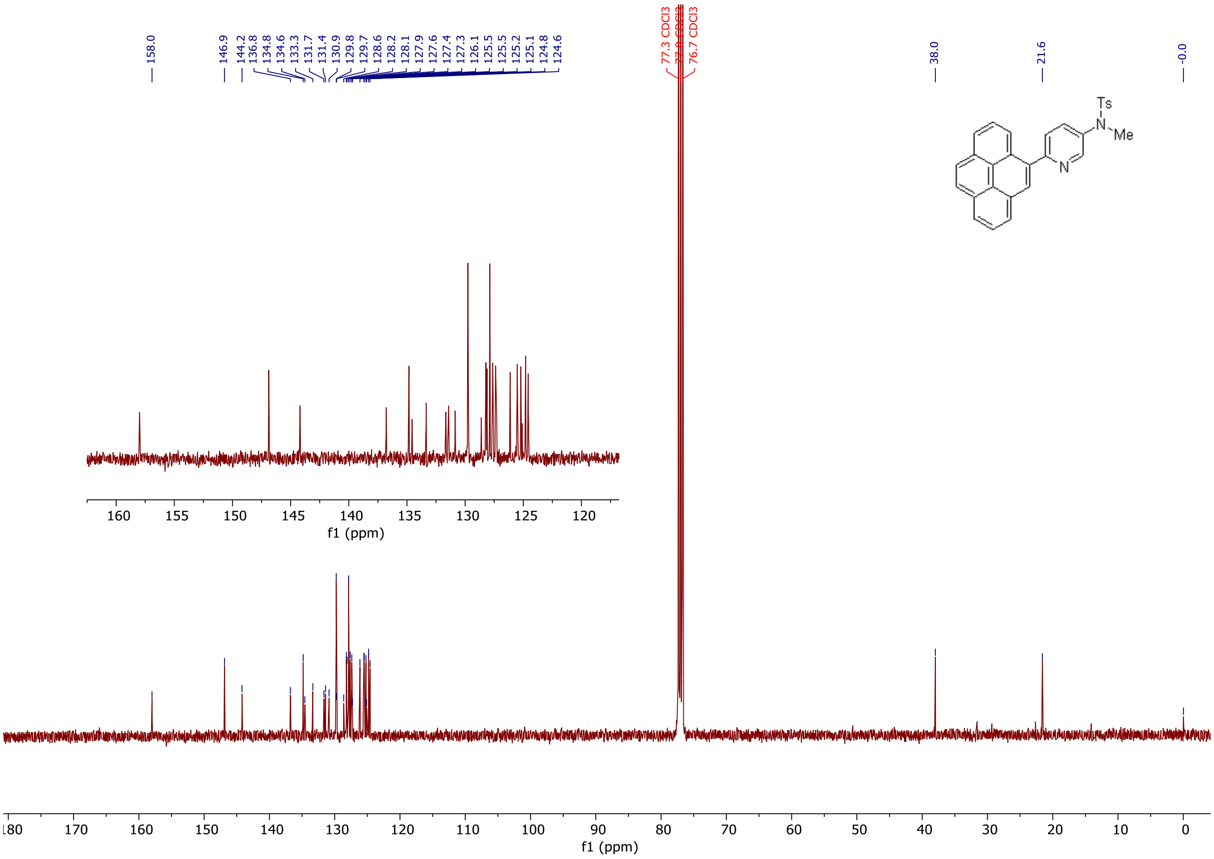

^{1}H NMR spectrum of compound 19a ($CDCl_3$, 298 K)

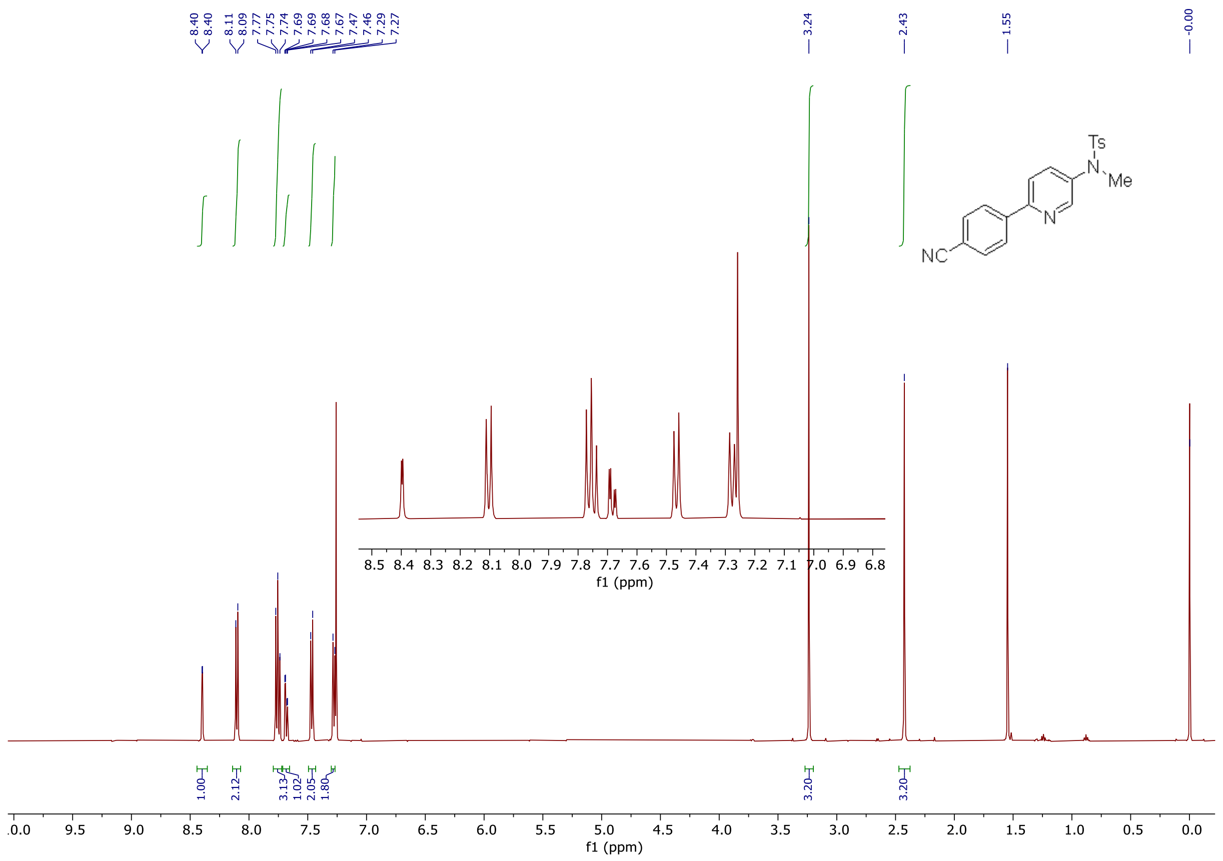

^{13}C NMR spectrum of compound 19a ($CDCl_3$, 298 K)

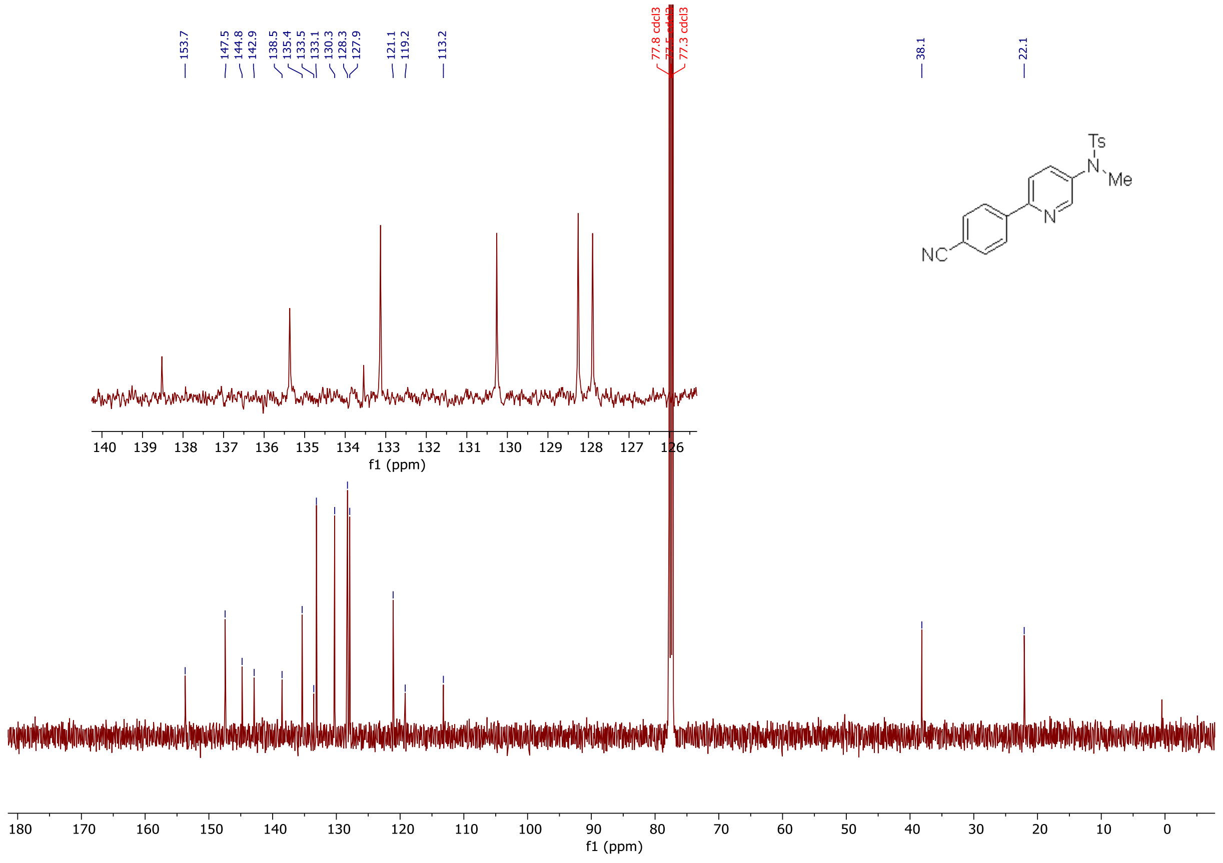

^{1}H NMR spectrum of compound 20a ($CDCl_3$, 298 K)

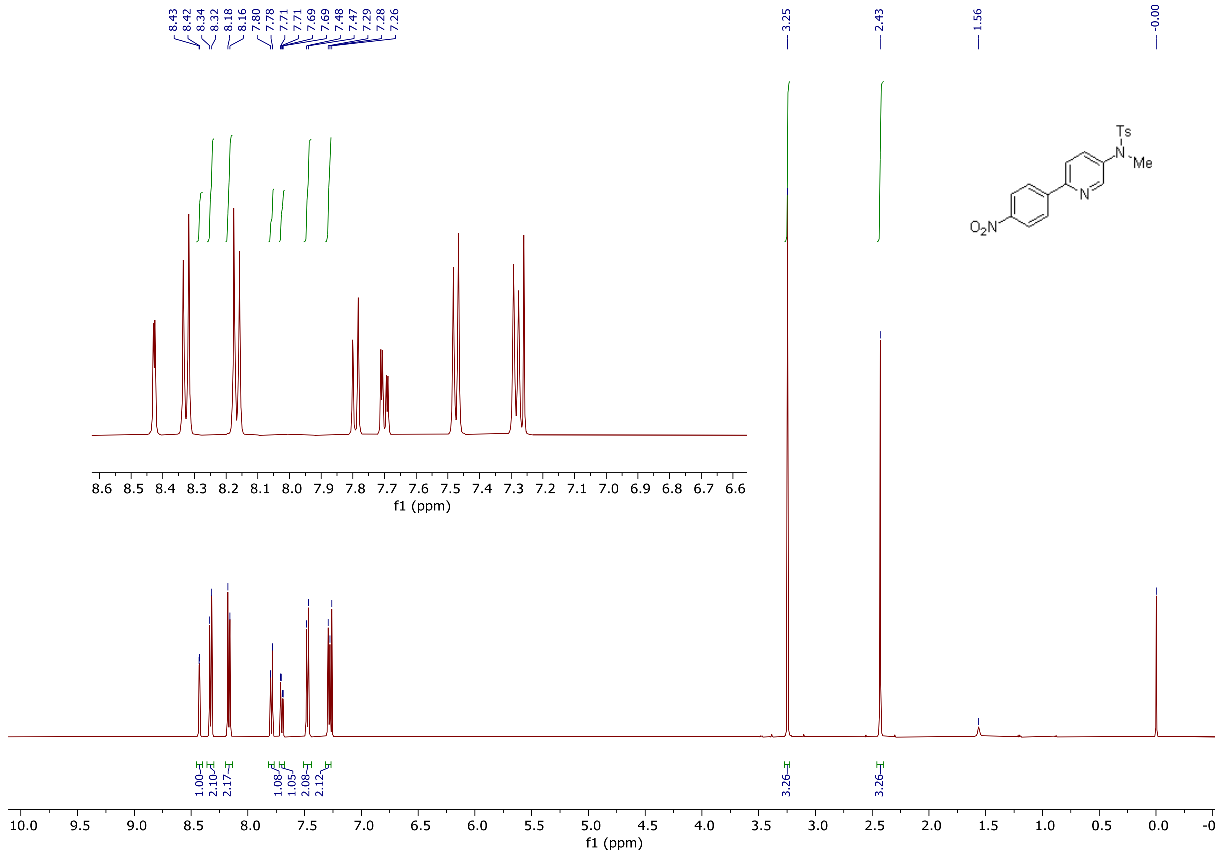

^{13}C NMR spectrum of compound 20a ($CDCl_3$, 298 K)

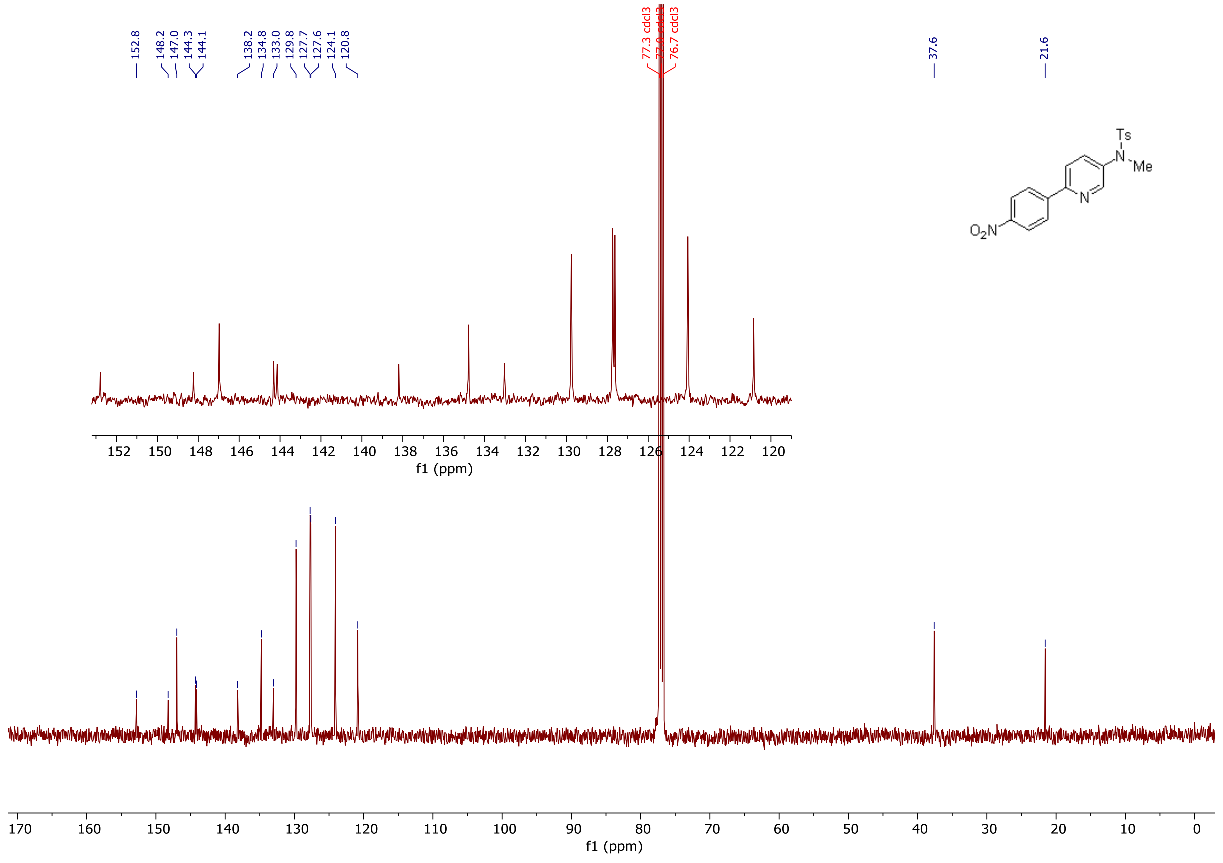

^{1}H NMR spectrum of compound 21a ($CDCl_3$, 298 K)

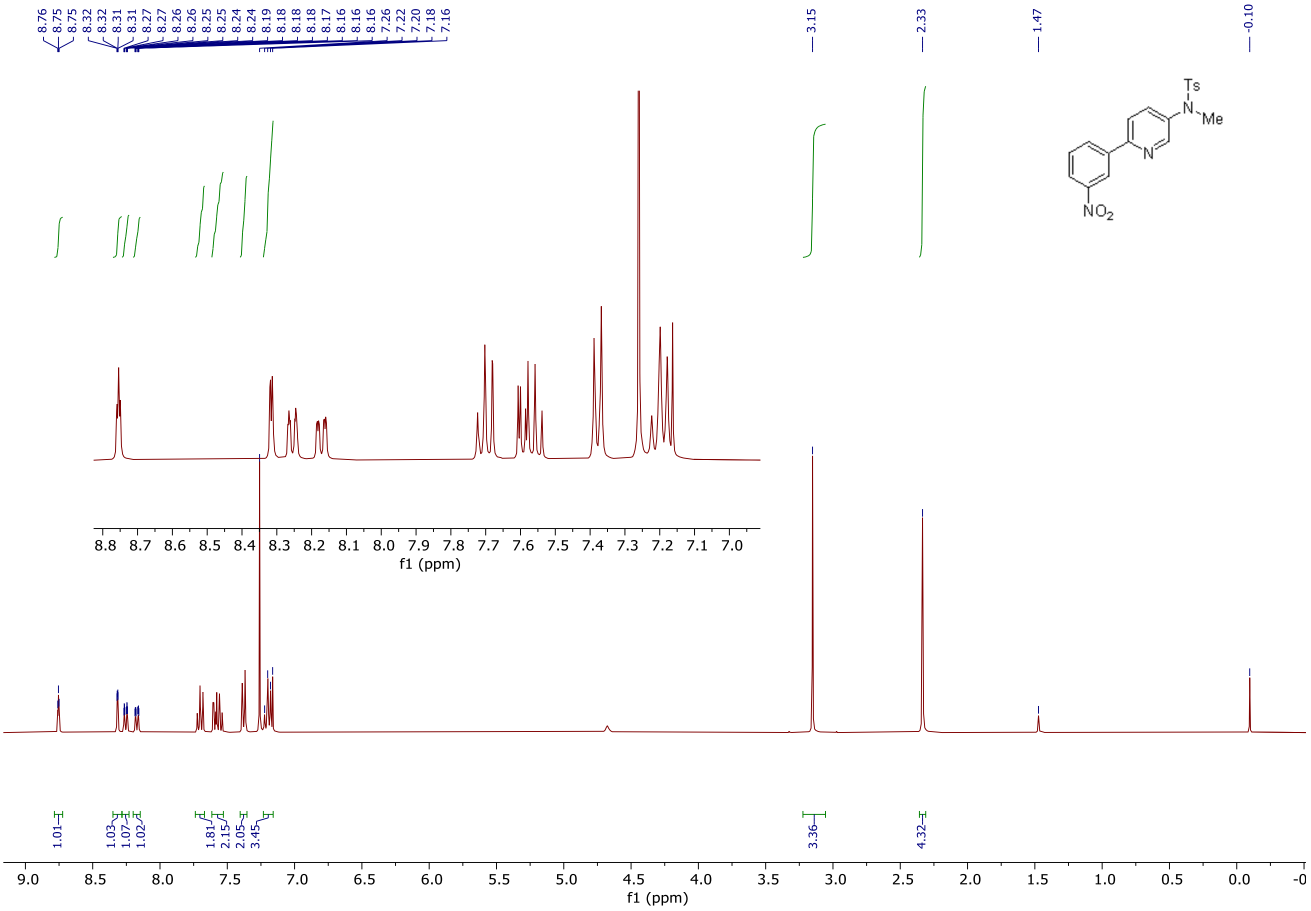

^{13}C NMR spectrum of compound 21a ($CDCl_3$, 298 K)

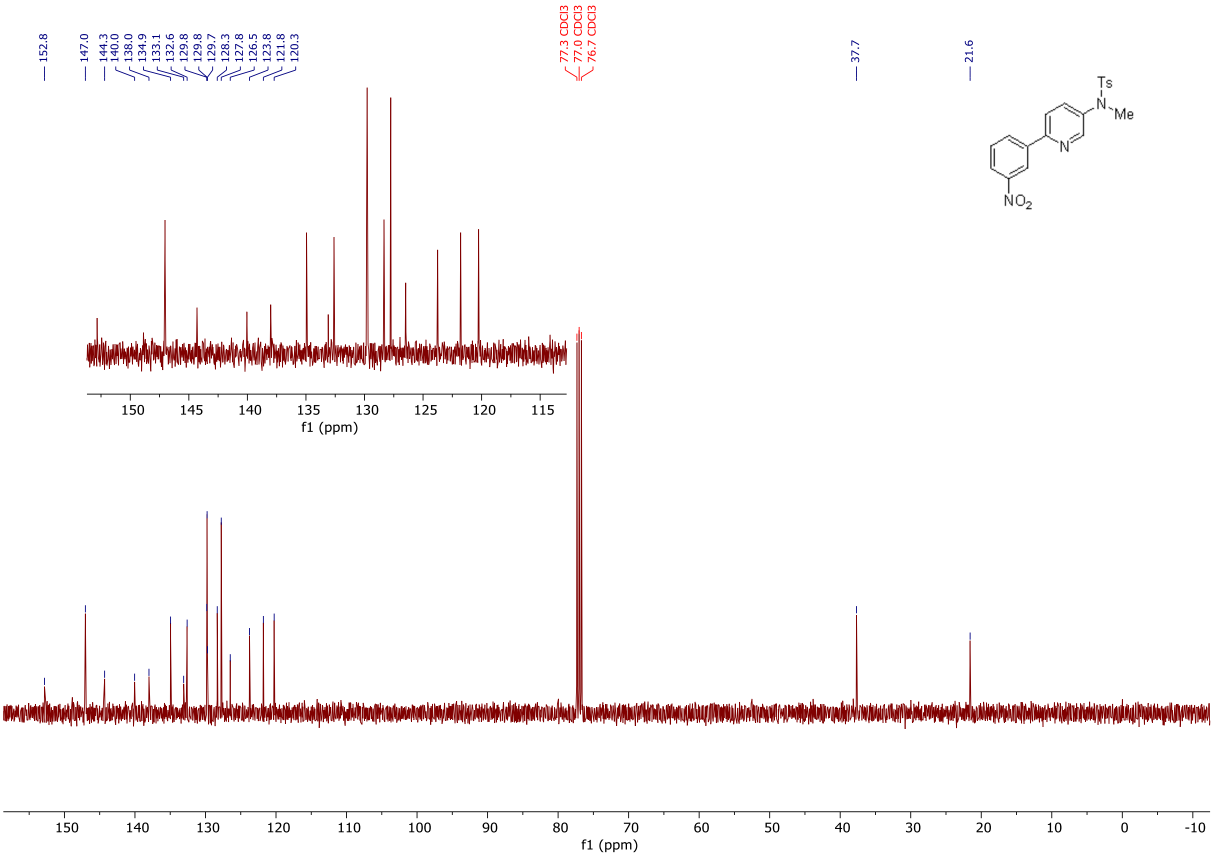

^{1}H NMR spectrum of compound 22a ($CDCl_3$, 298 K)

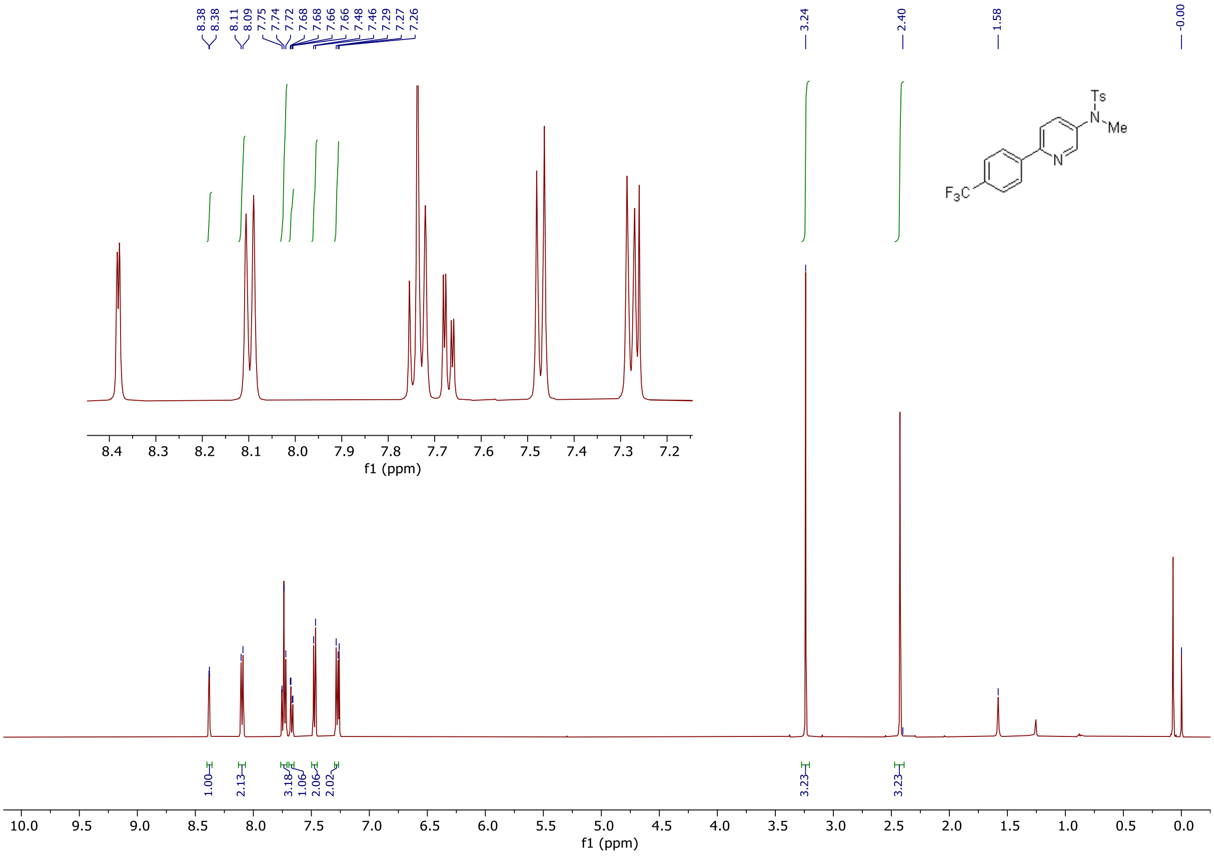

^{13}C NMR spectrum of compound 22a ($CDCl_3$, 298 K)

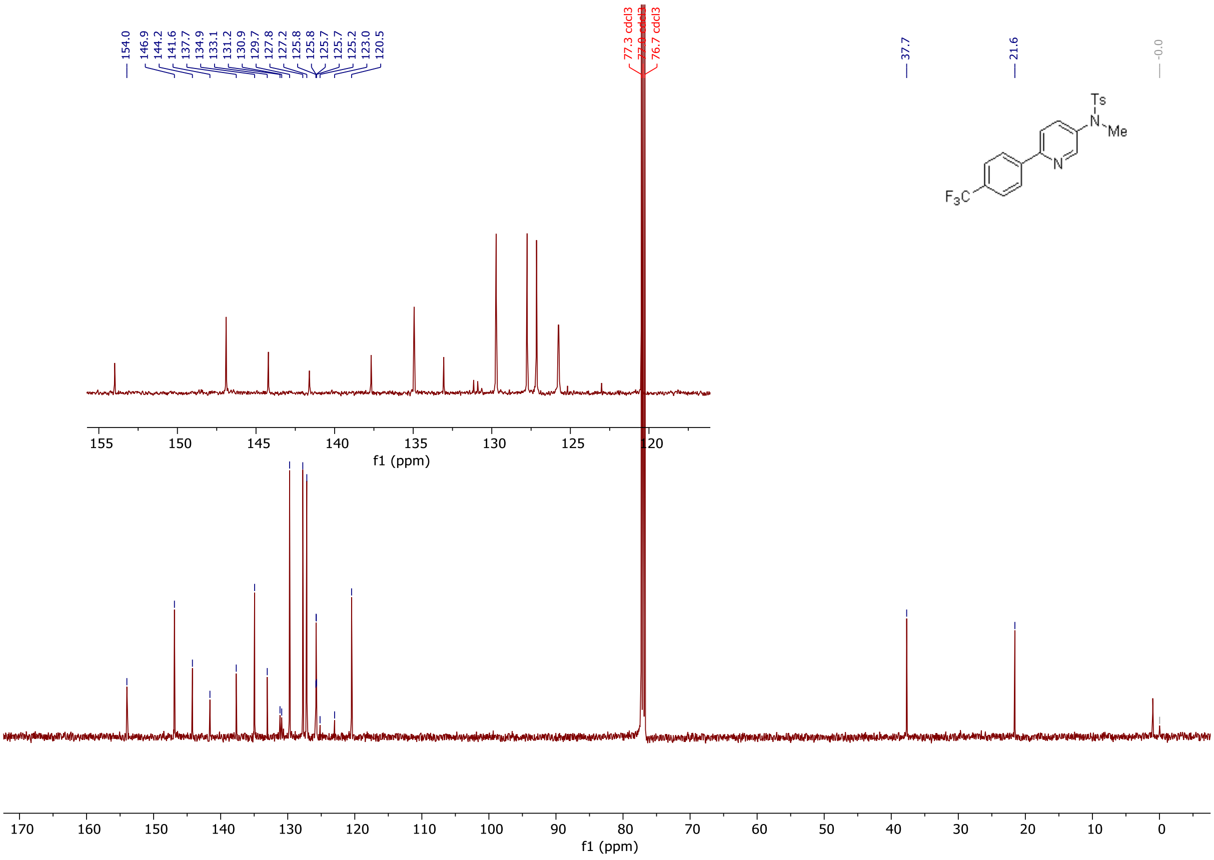

^{1}H NMR spectrum of compound 23a ($CDCl_3$, 298 K)

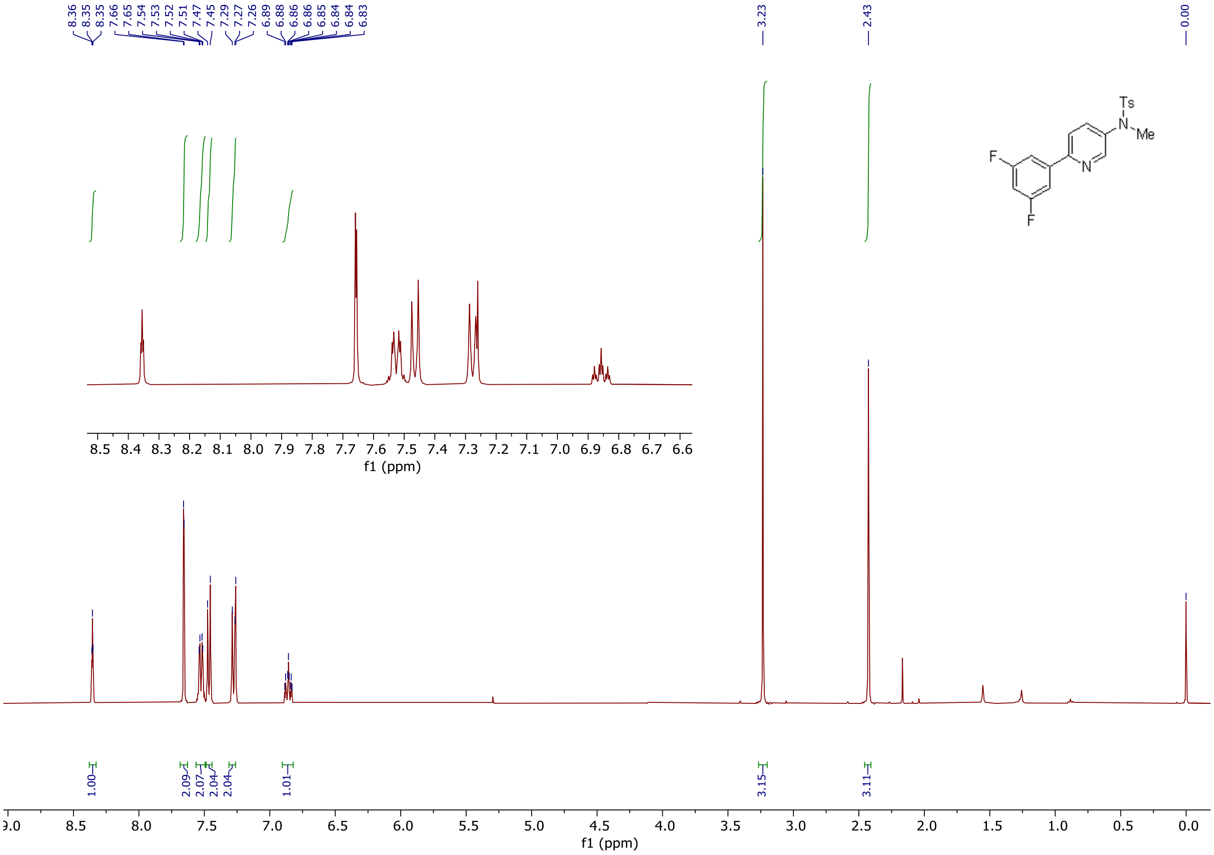

^{13}C NMR spectrum of compound 23a ($CDCl_3$, 298 K)

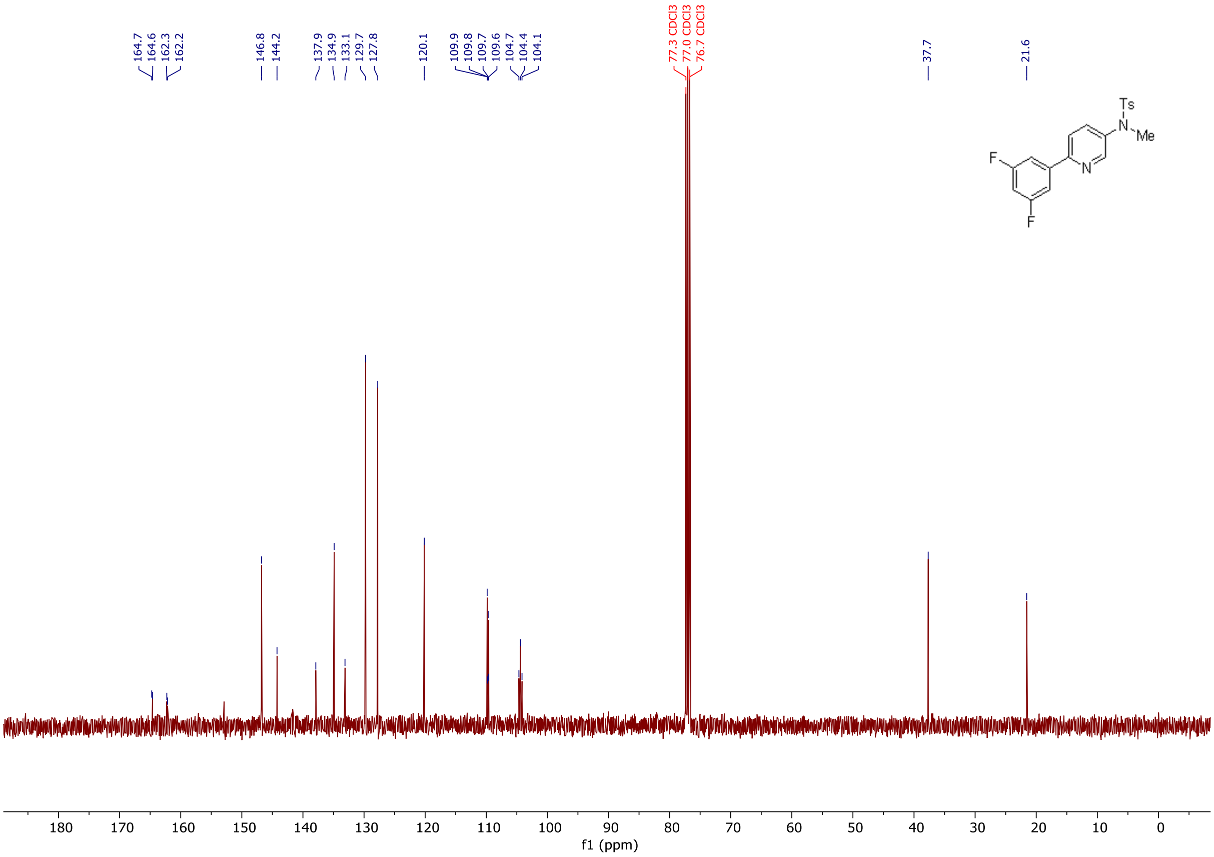

^{1}H NMR spectrum of compound 24a ($CDCl_3$, 298 K)

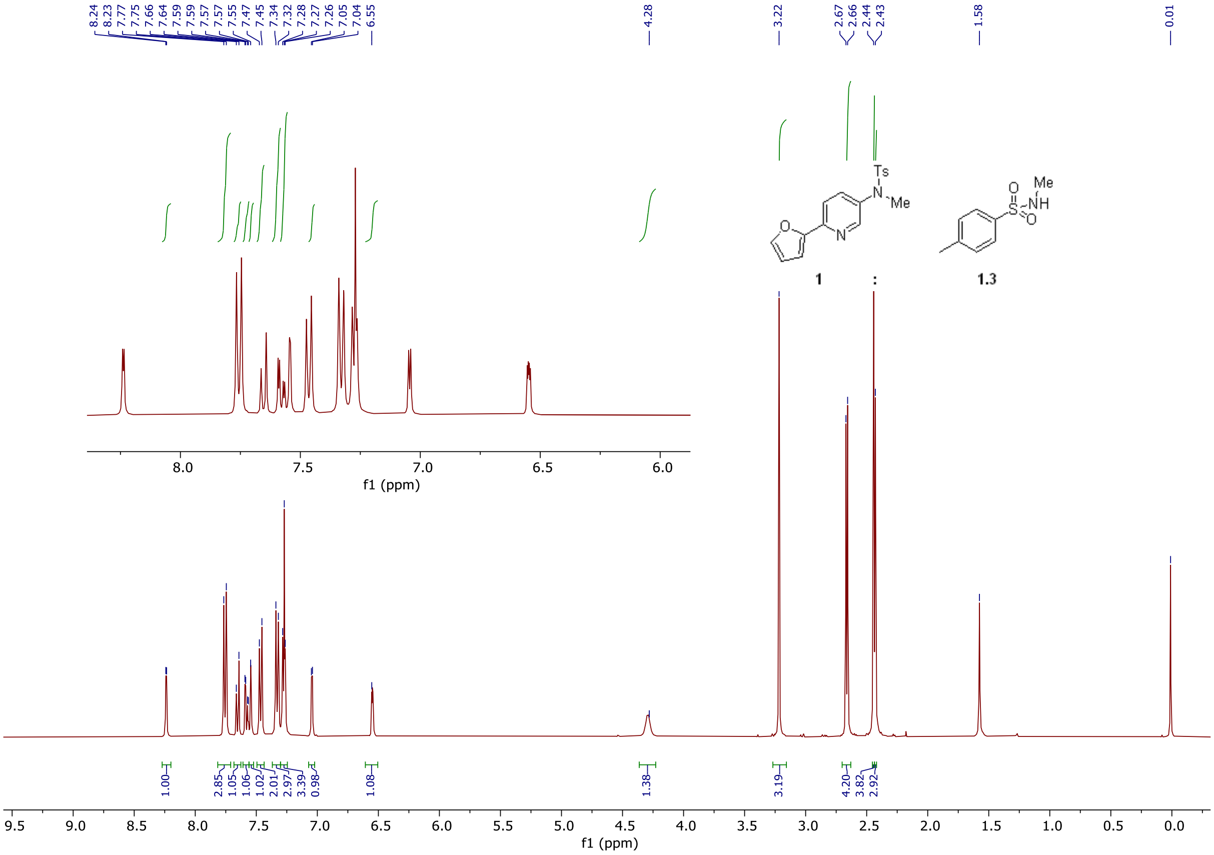

^{13}C NMR spectrum of compound 24a ($CDCl_3$, 298 K)

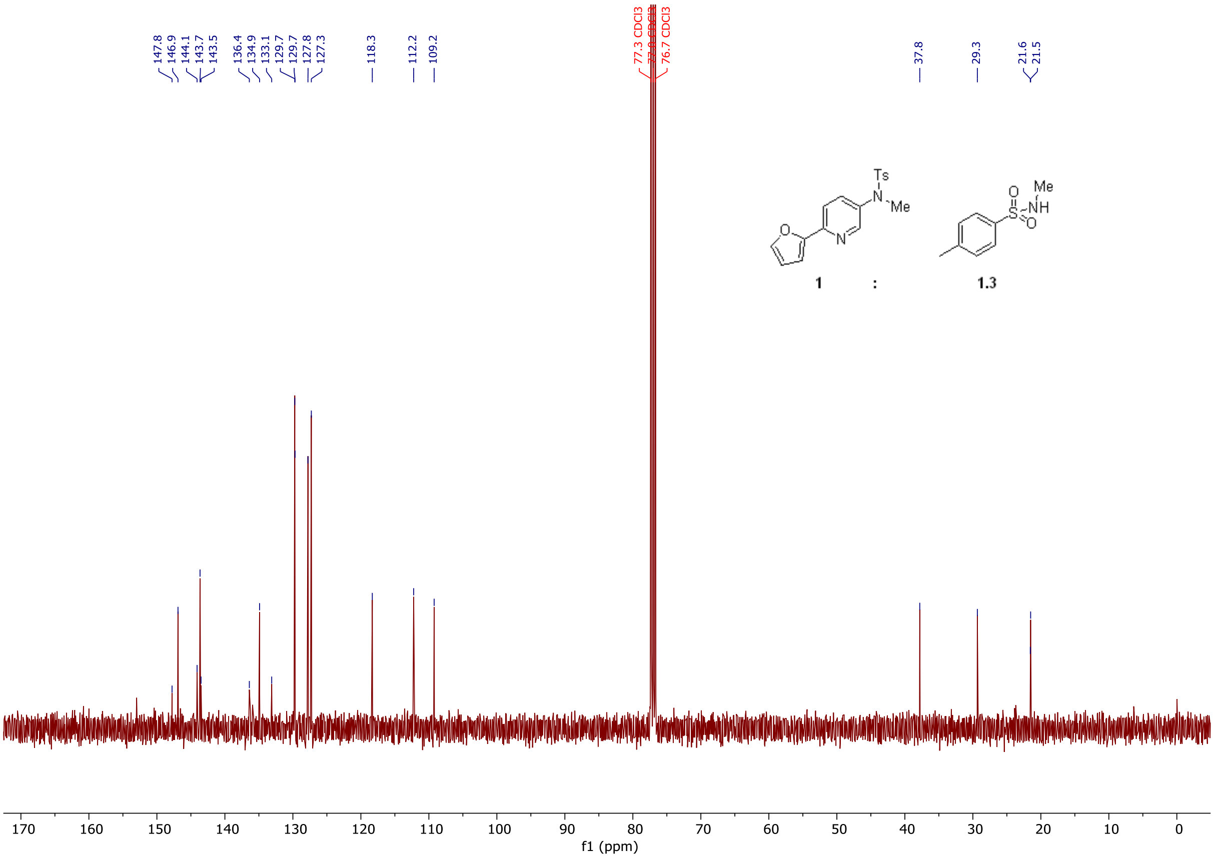

^{1}H NMR spectrum of compound 24b ($CDCl_3$, 298 K)

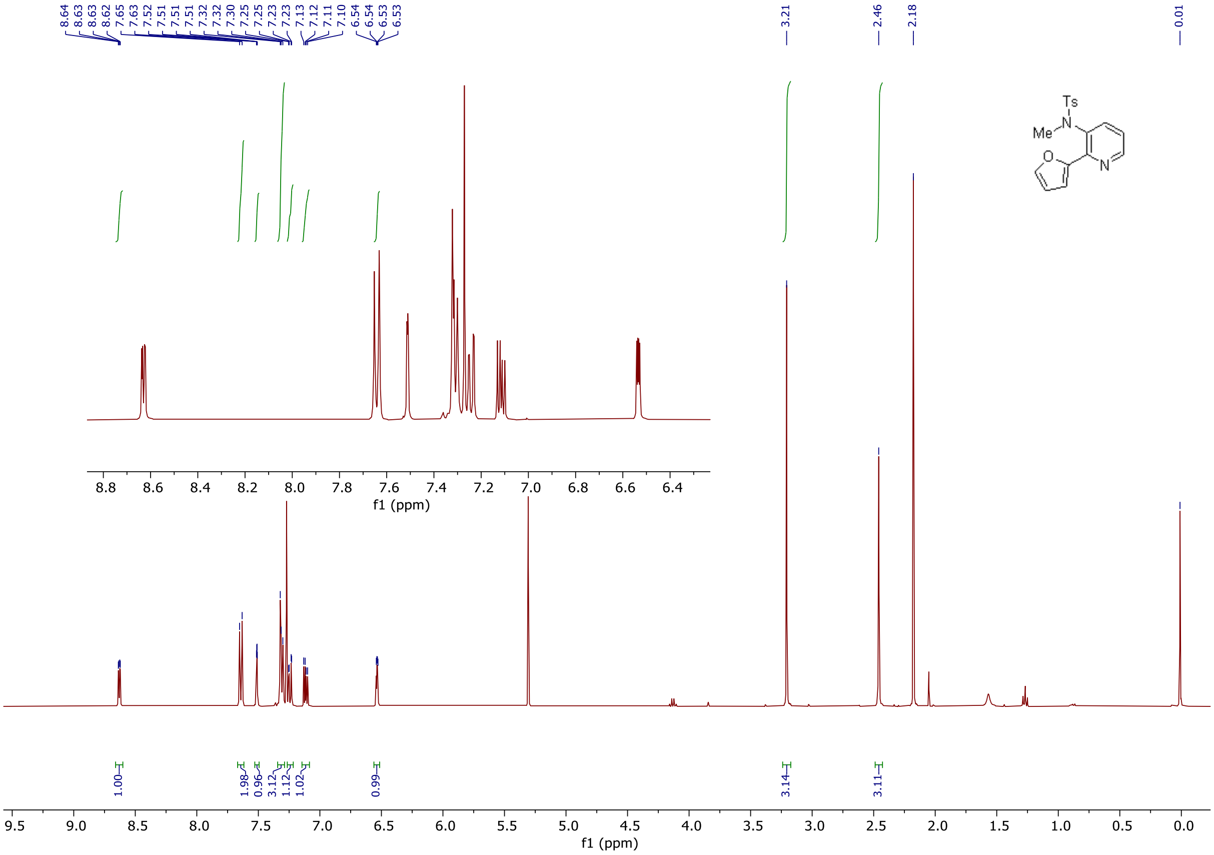

^{13}C NMR spectrum of compound 24b ($CDCl_3$, 298 K)

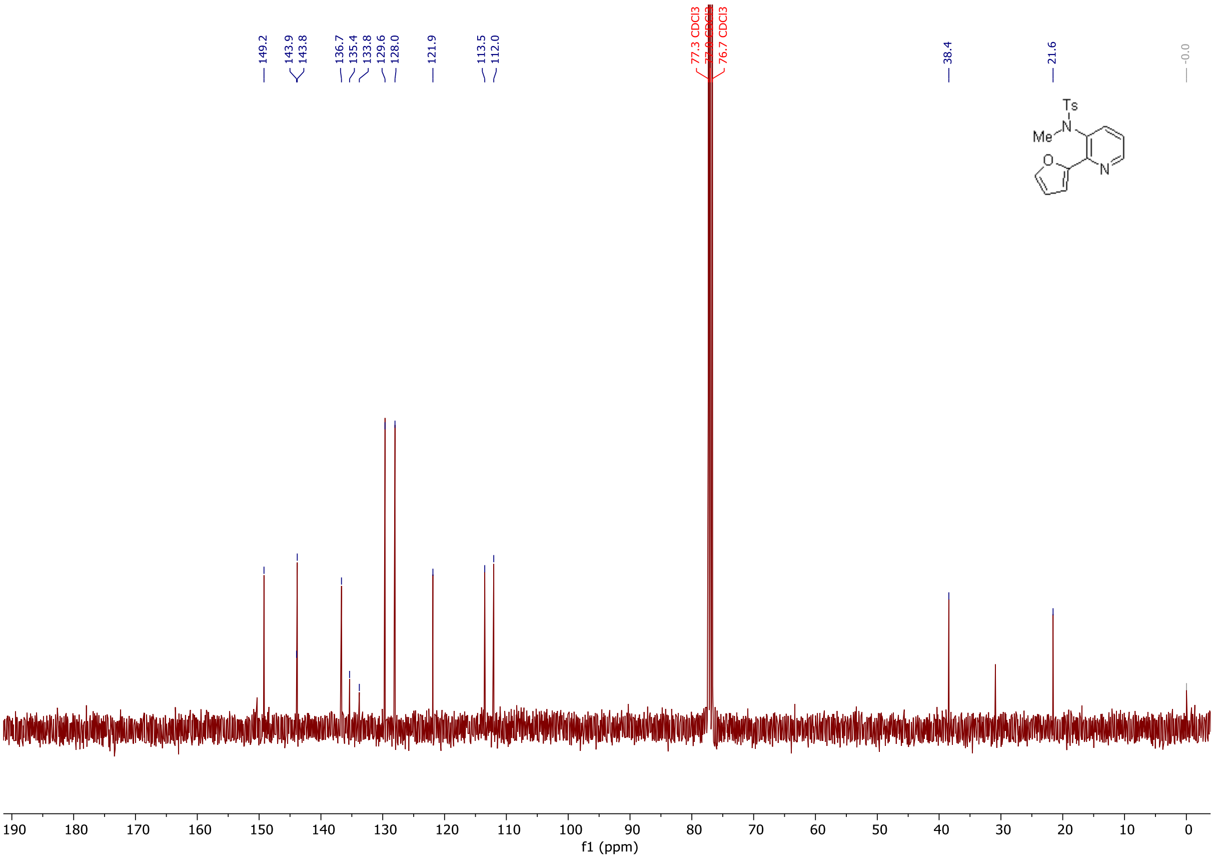

^{1}H NMR spectrum of compound 25a ($CDCl_3$, 298 K)

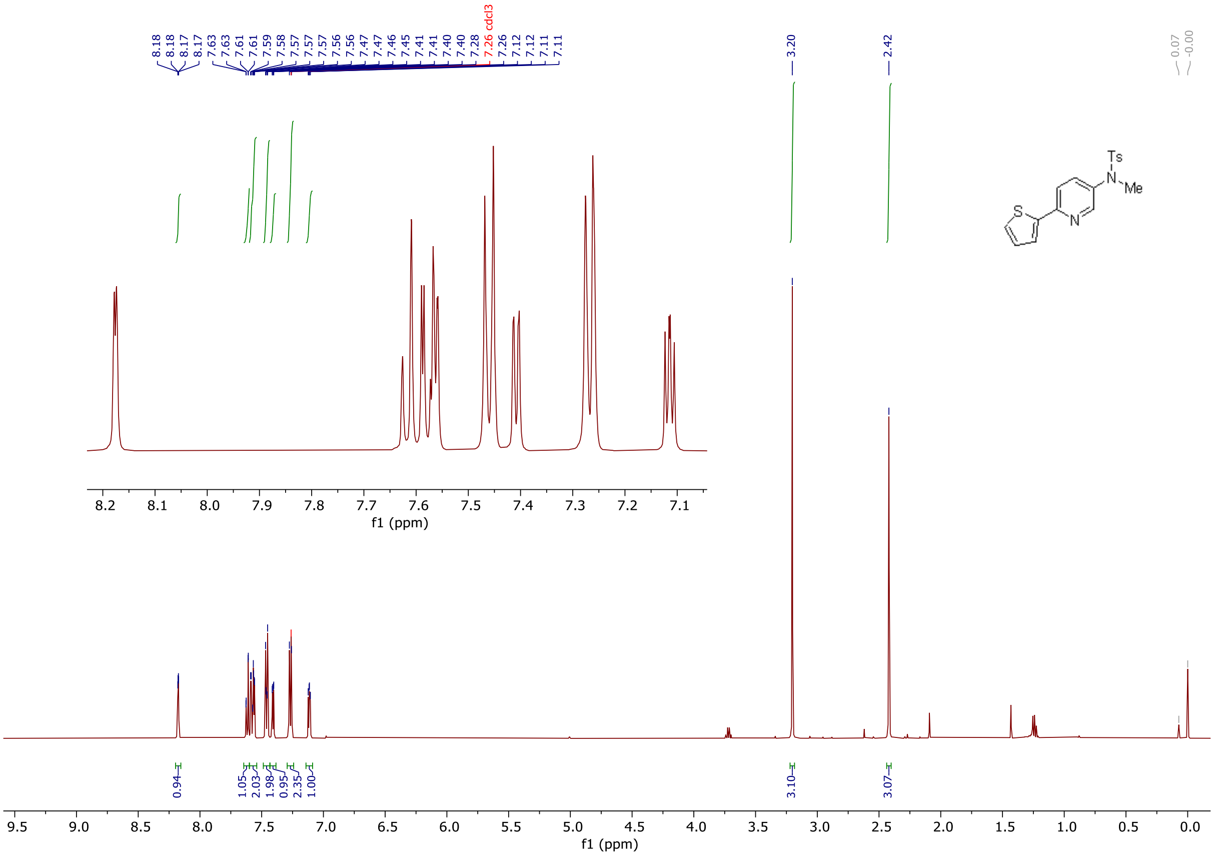

^{13}C NMR spectrum of compound 25a ($CDCl_3$, 298 K)

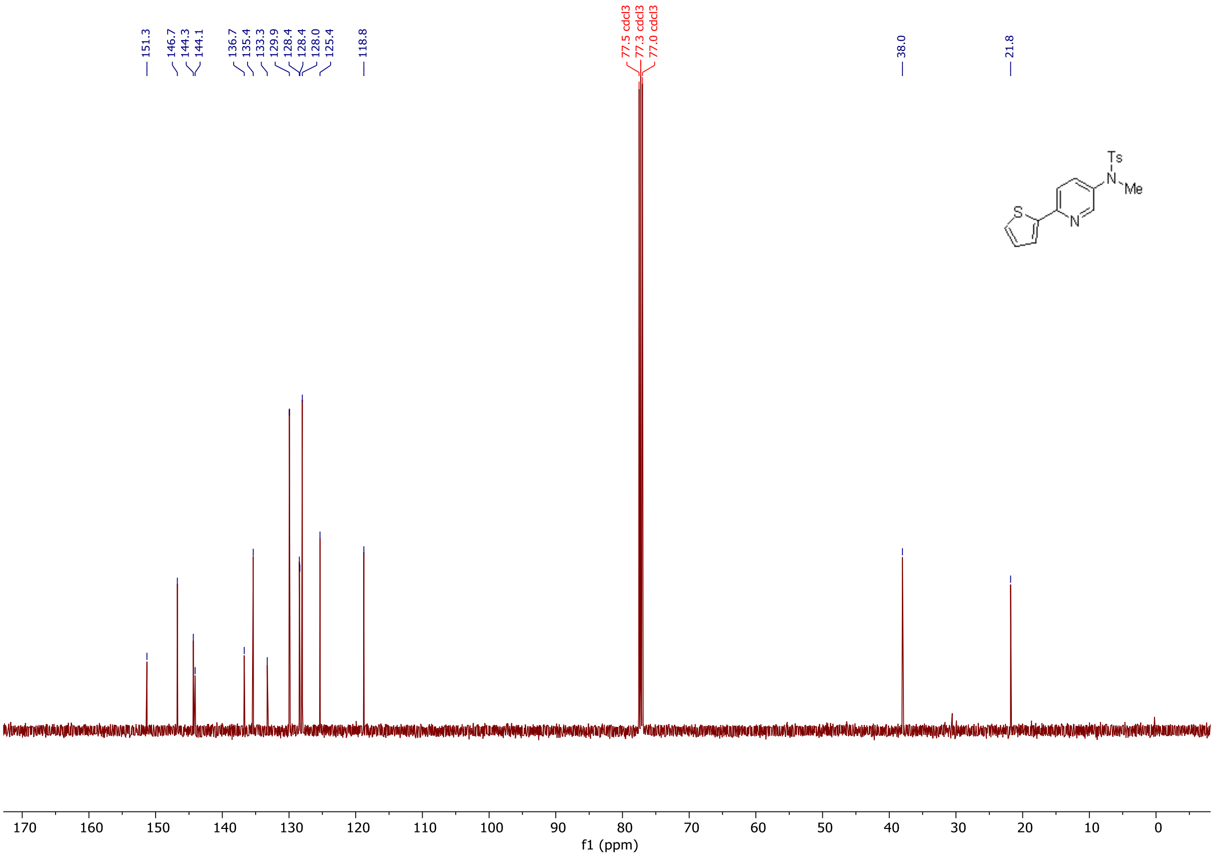

^{1}H NMR spectrum of compound 26a ($CDCl_3$, 298 K)

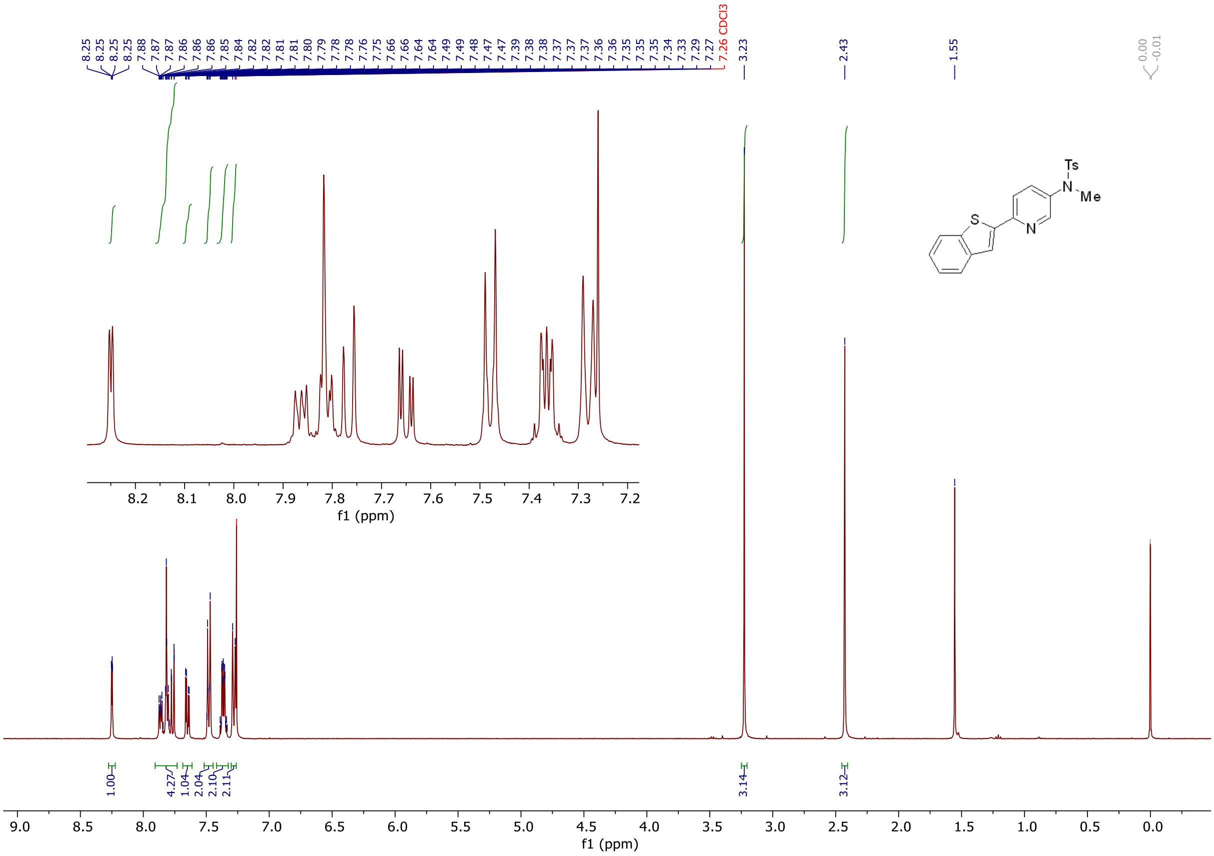

^{13}C NMR spectrum of compound 26a ($CDCl_3$, 298 K)

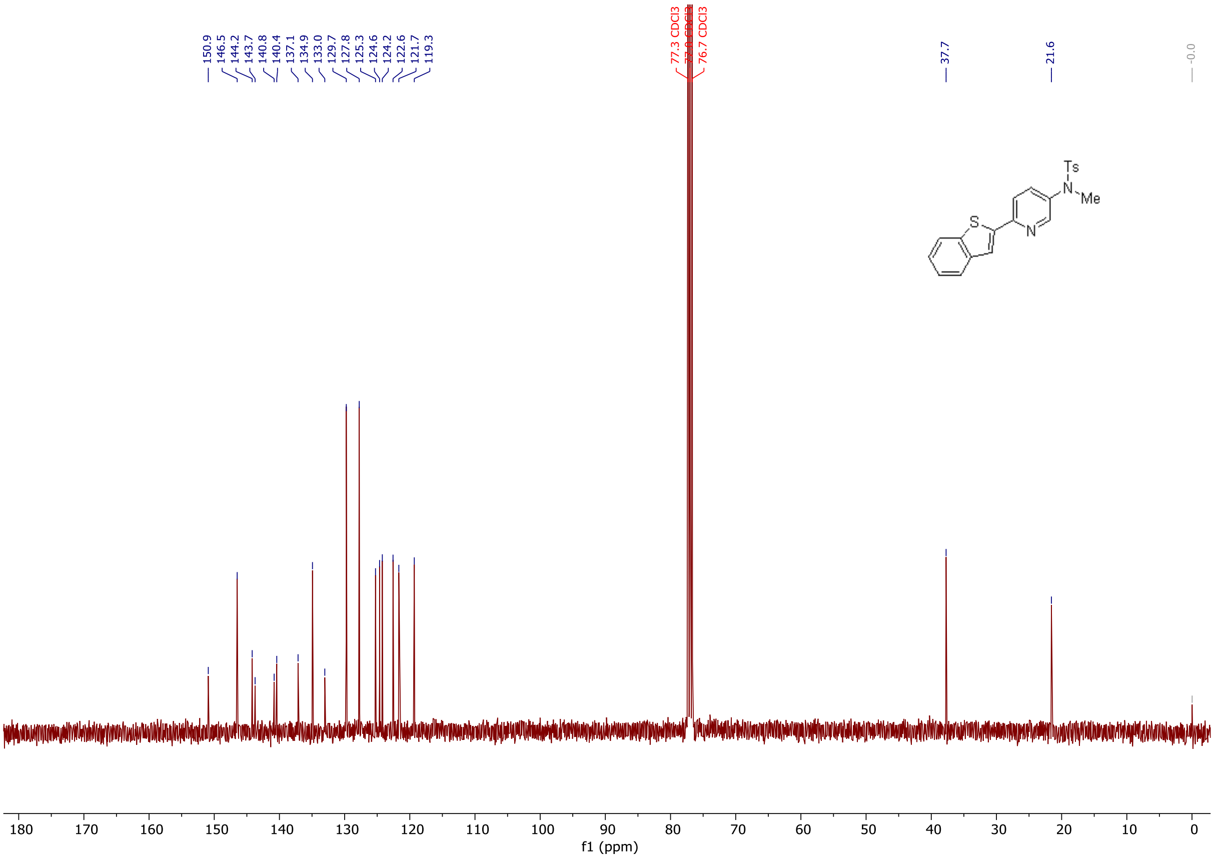

^{1}H NMR spectrum of compound 27a ($CDCl_3$, 298 K)

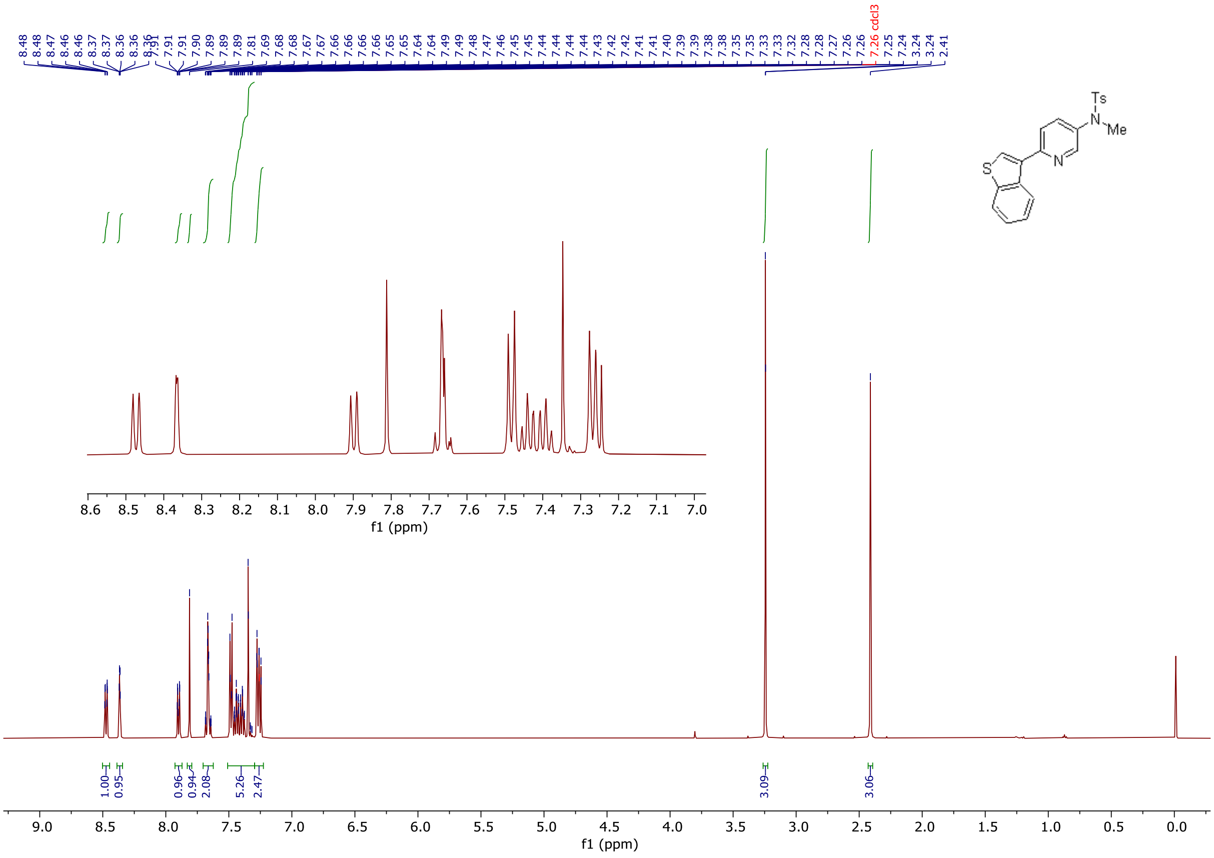

^{13}C NMR spectrum of compound 27a ($CDCl_3$, 298 K)

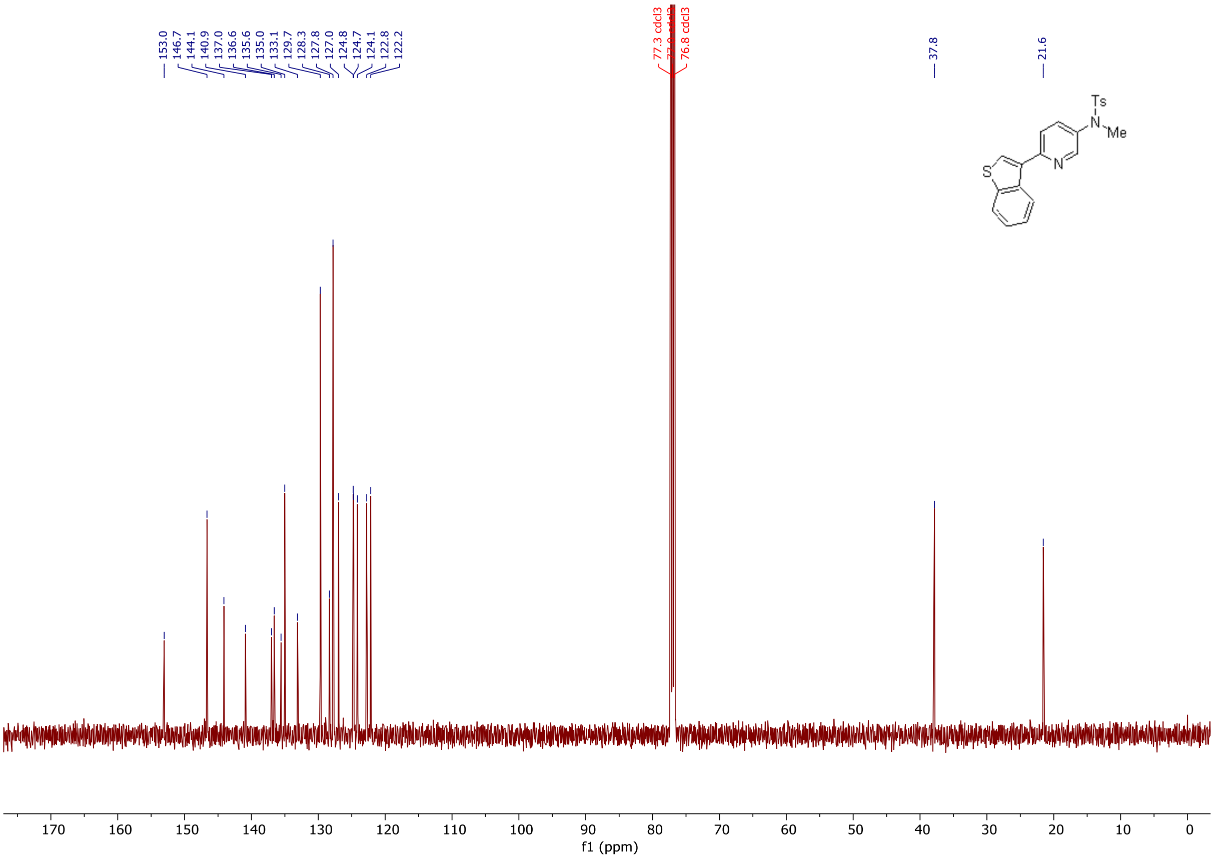

^{1}H NMR spectrum of compound 28a ($CDCl_3$, 298 K)

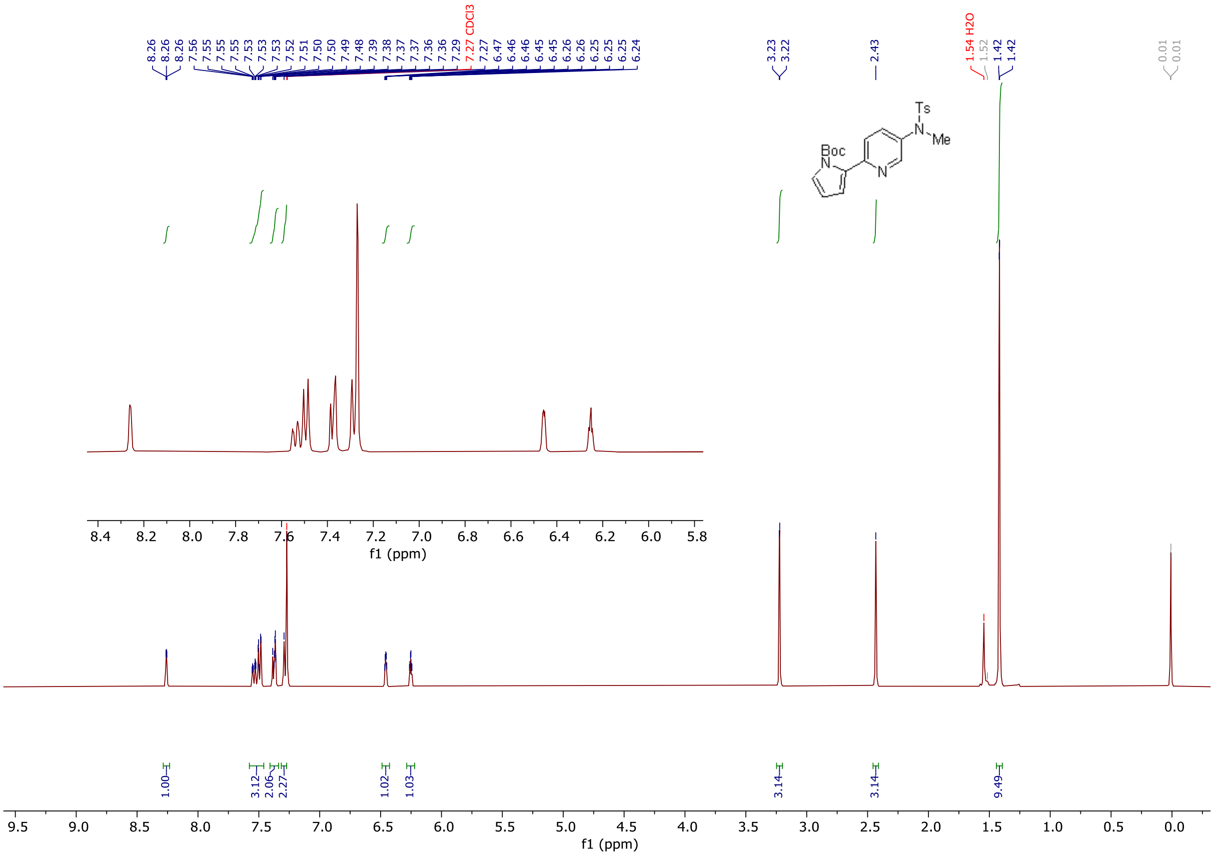

^{13}C NMR spectrum of compound 28a ($CDCl_3$, 298 K)

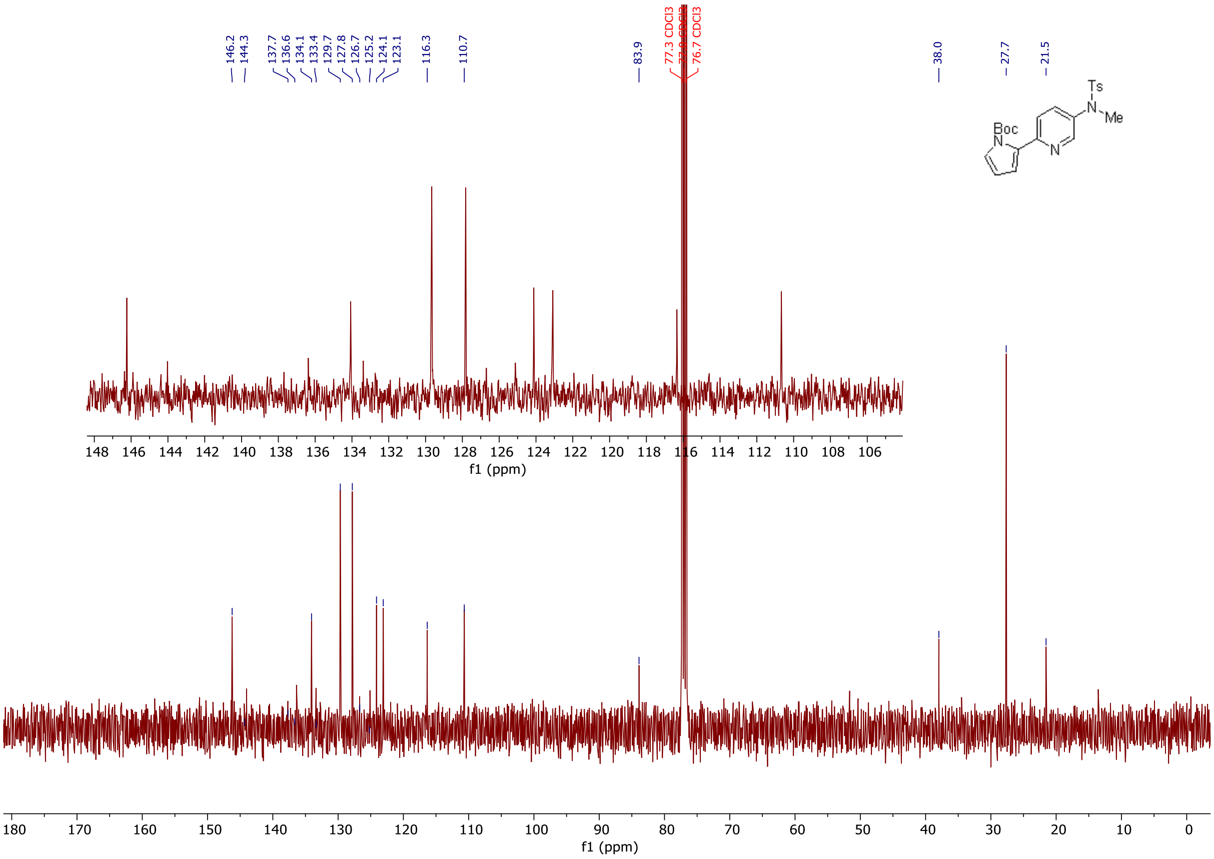

^{1}H NMR spectrum of compound 29a ($CDCl_3$, 298 K)

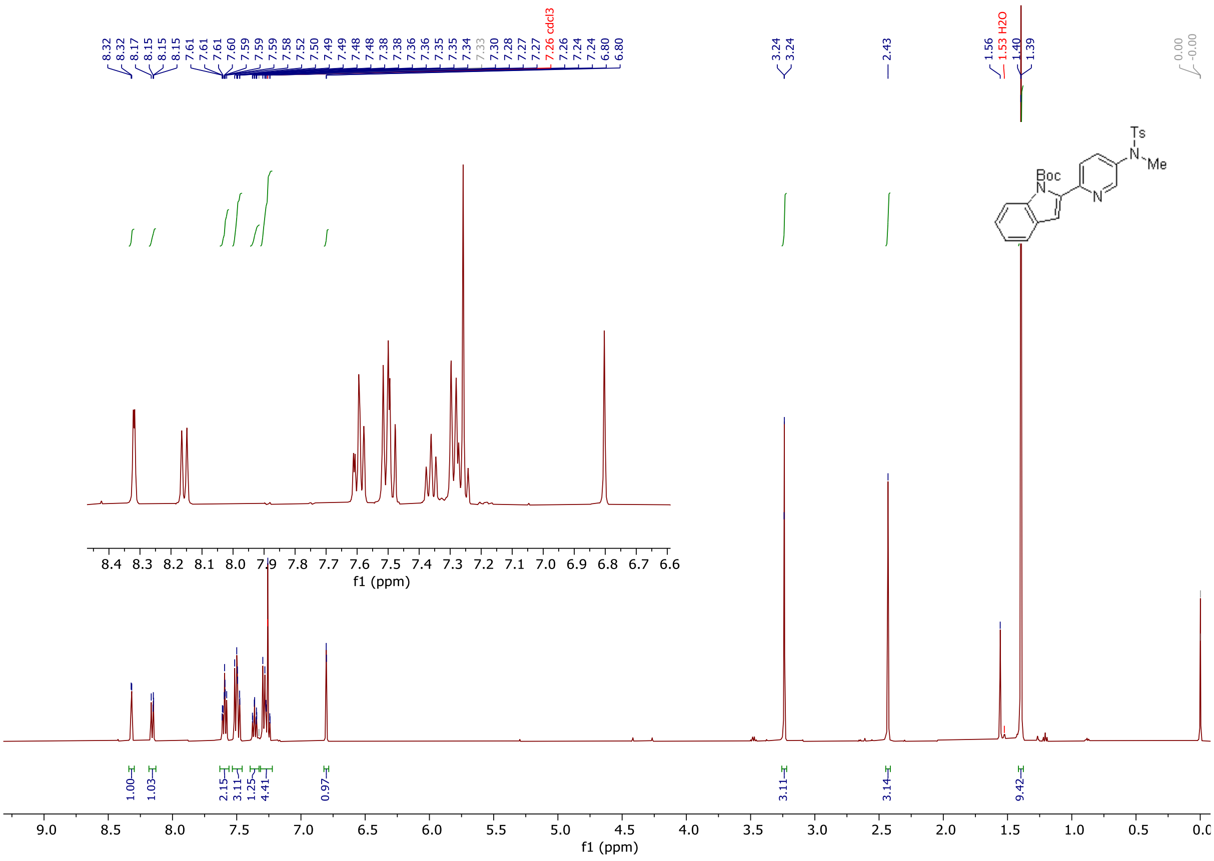

^{13}C NMR spectrum of compound 29a ($CDCl_3$, 298 K)

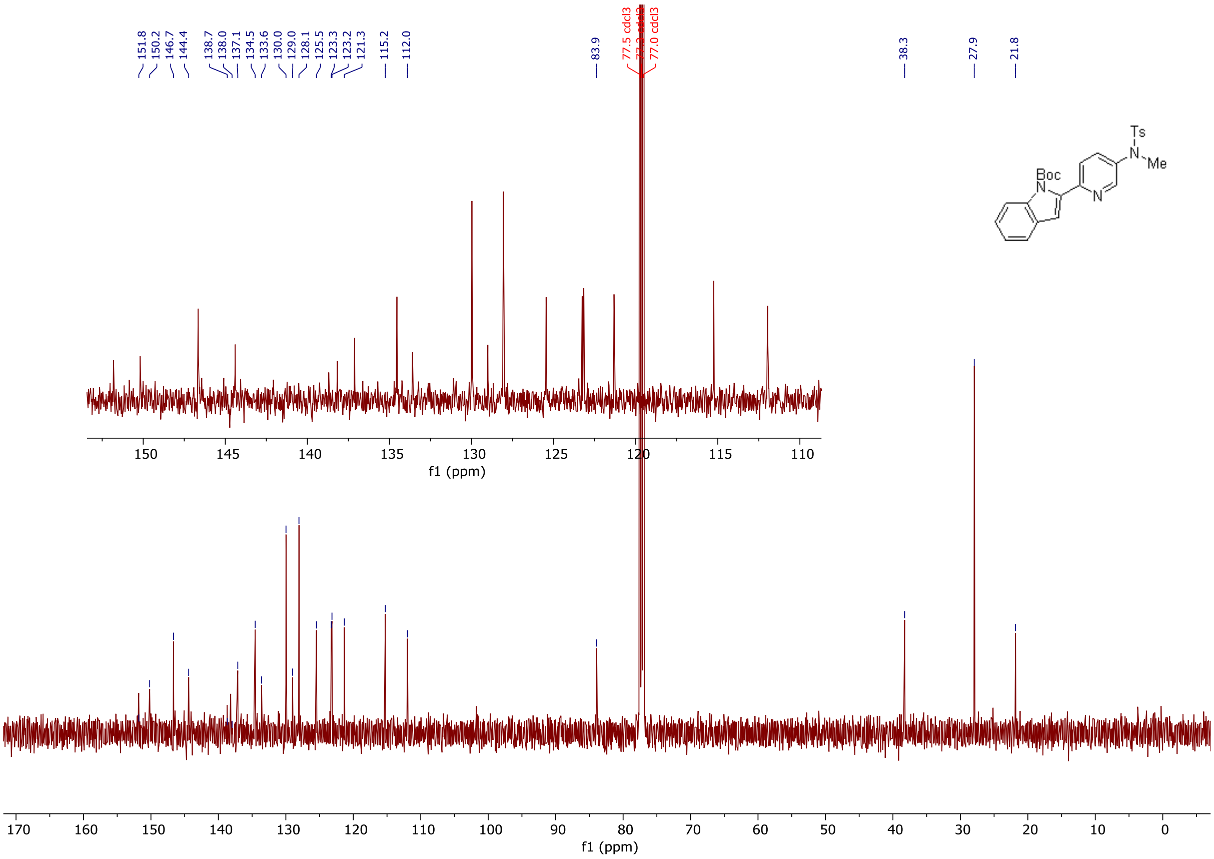

^{1}H NMR spectrum of compound 30b ($CDCl_3$, 298 K)

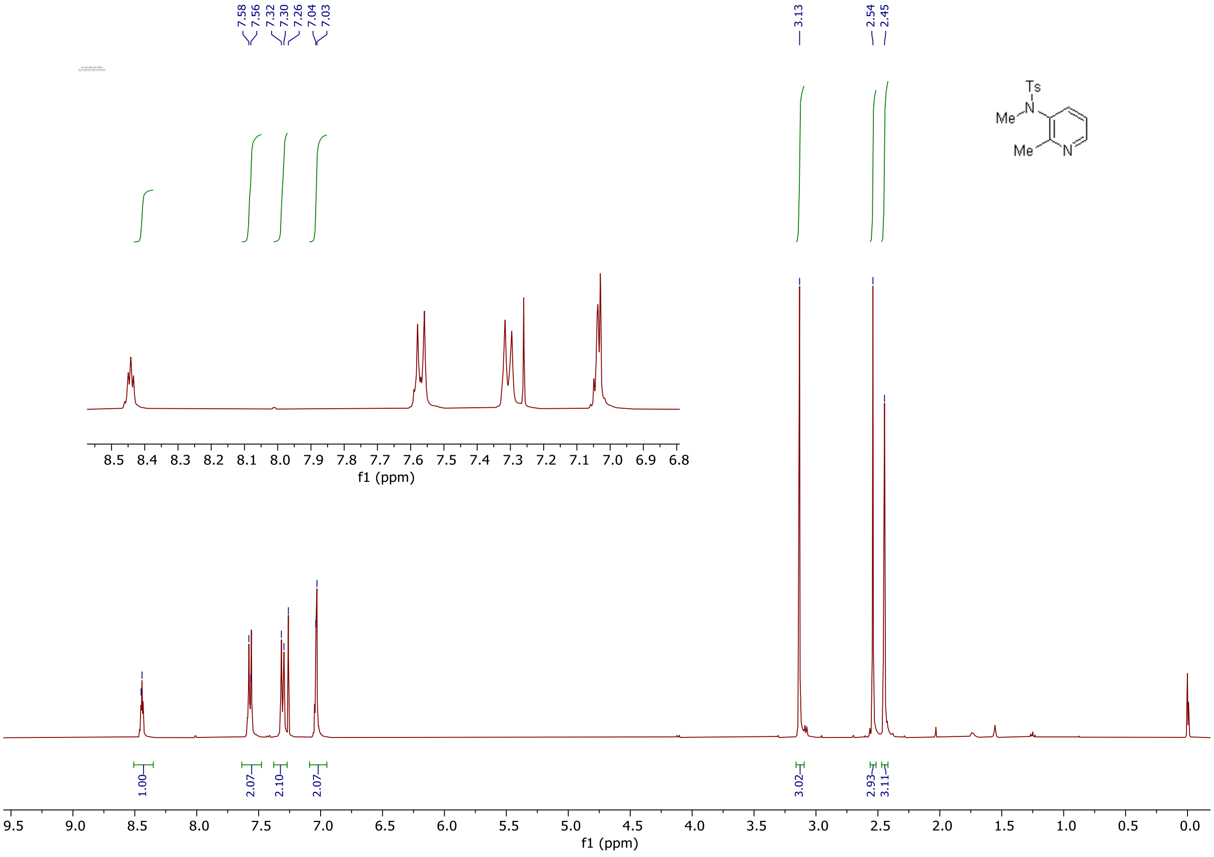

^{13}C NMR spectrum of compound 30b ($CDCl_3$, 298 K)

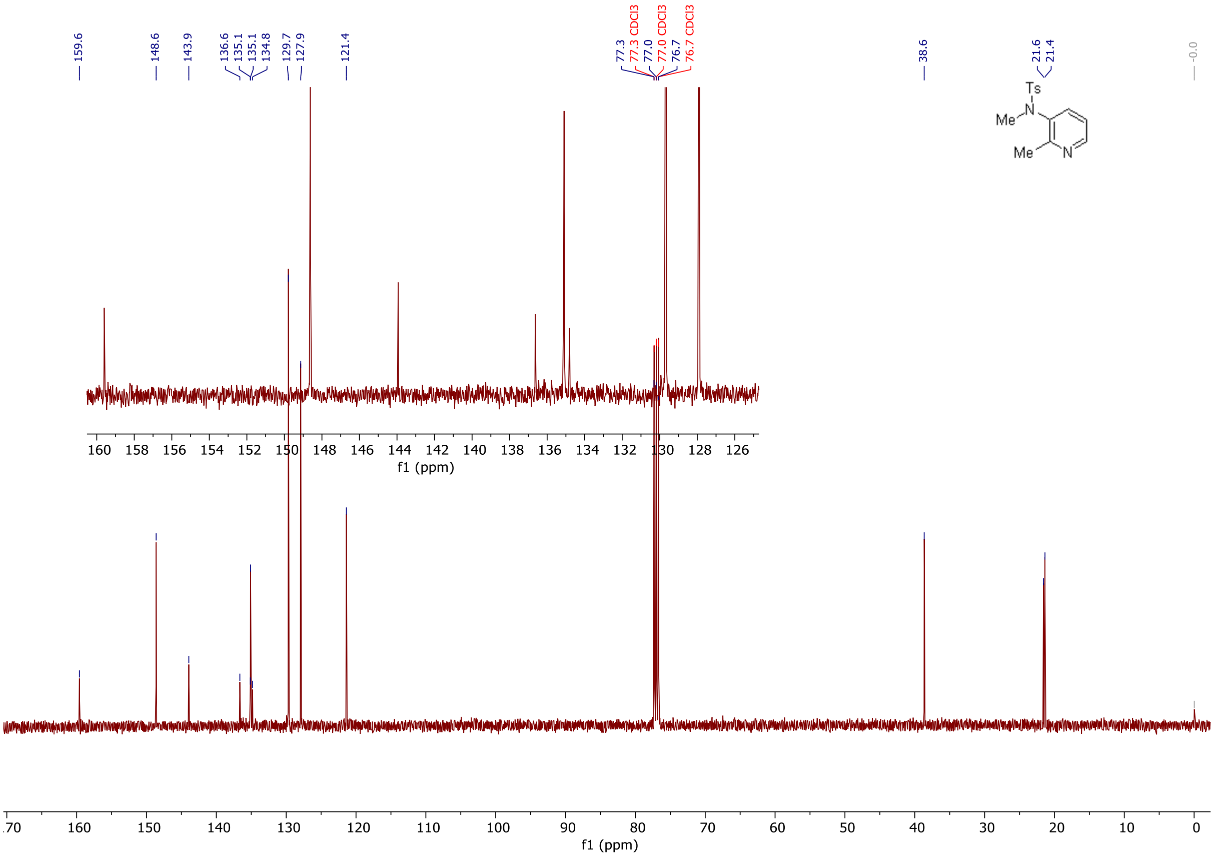

^{1}H NMR spectrum of compound 31b ($CDCl_3$, 298 K)

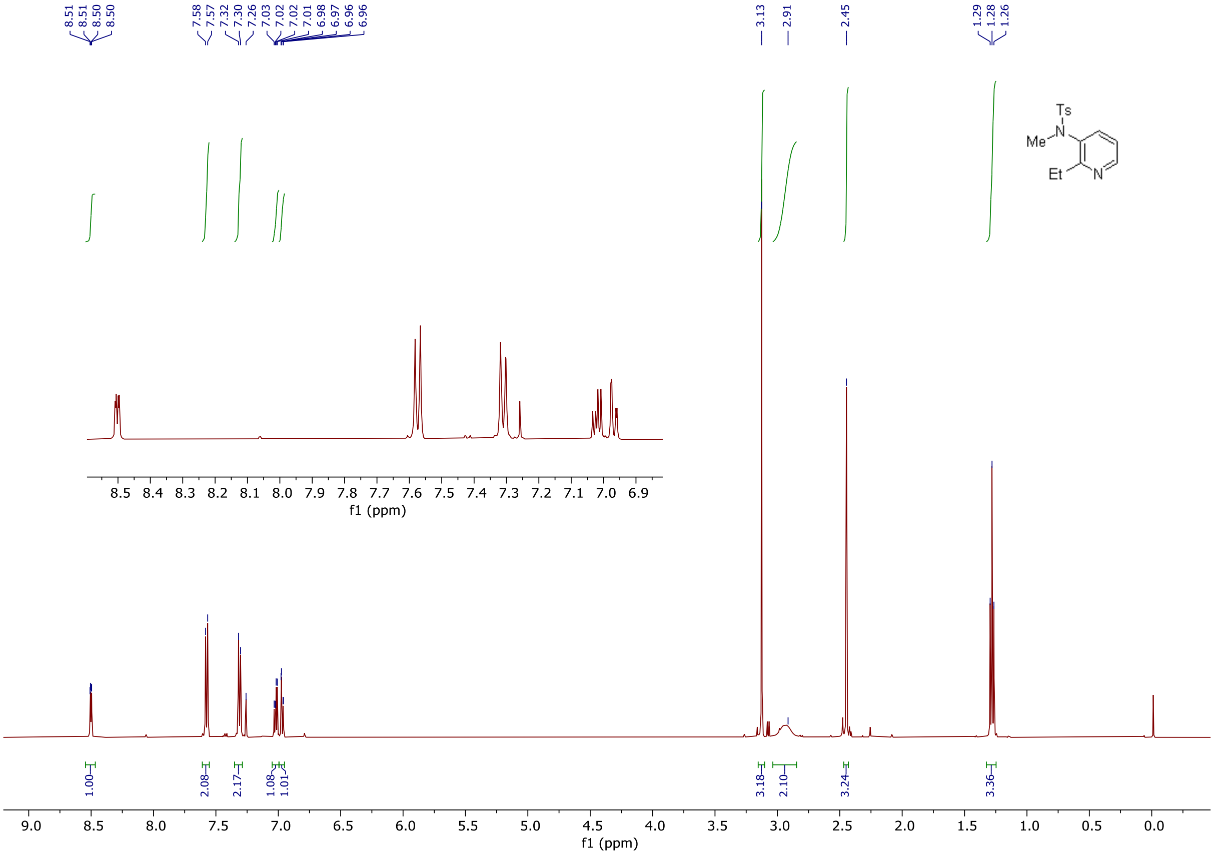

^{13}C NMR spectrum of compound 31b ($CDCl_3$, 298 K)

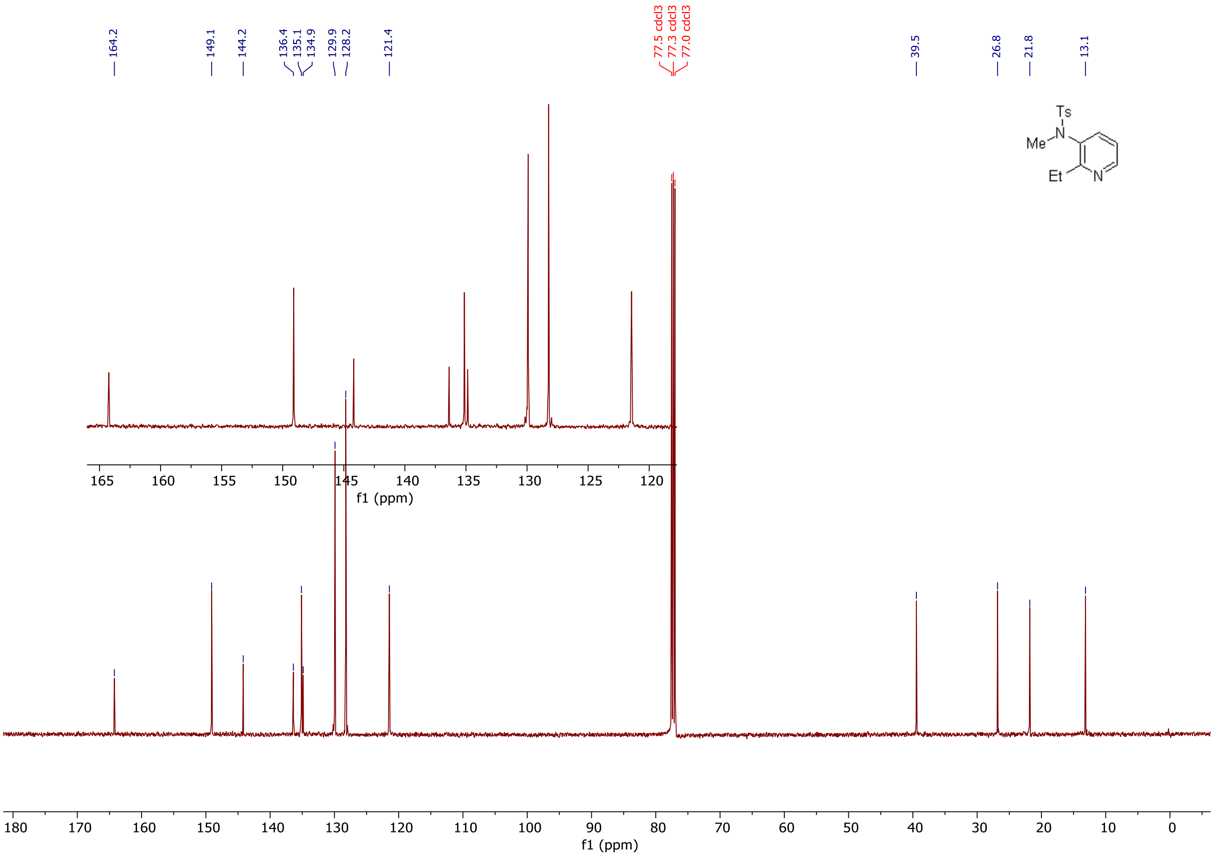

^{1}H NMR spectrum of compound 32b ($CDCl_3$, 298 K)

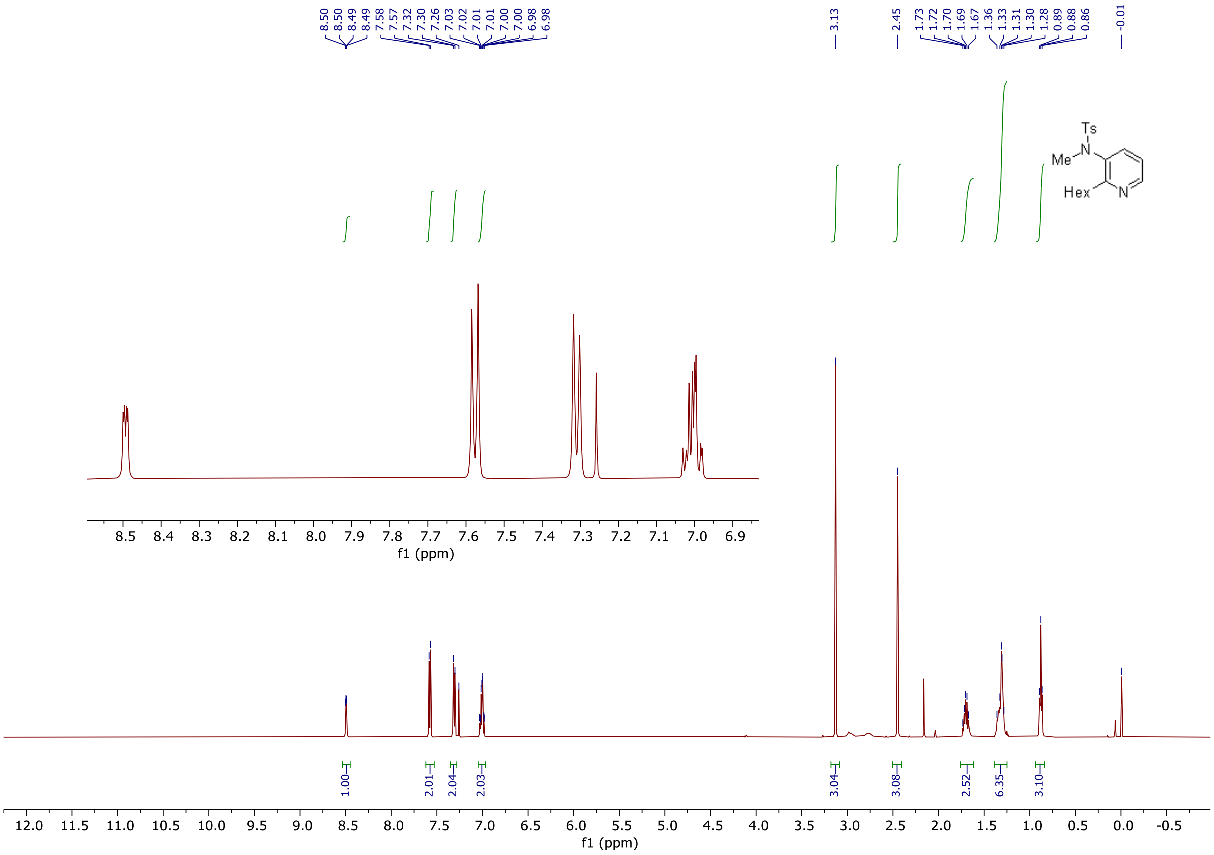

^{13}C NMR spectrum of compound 32b ($CDCl_3$, 298 K)

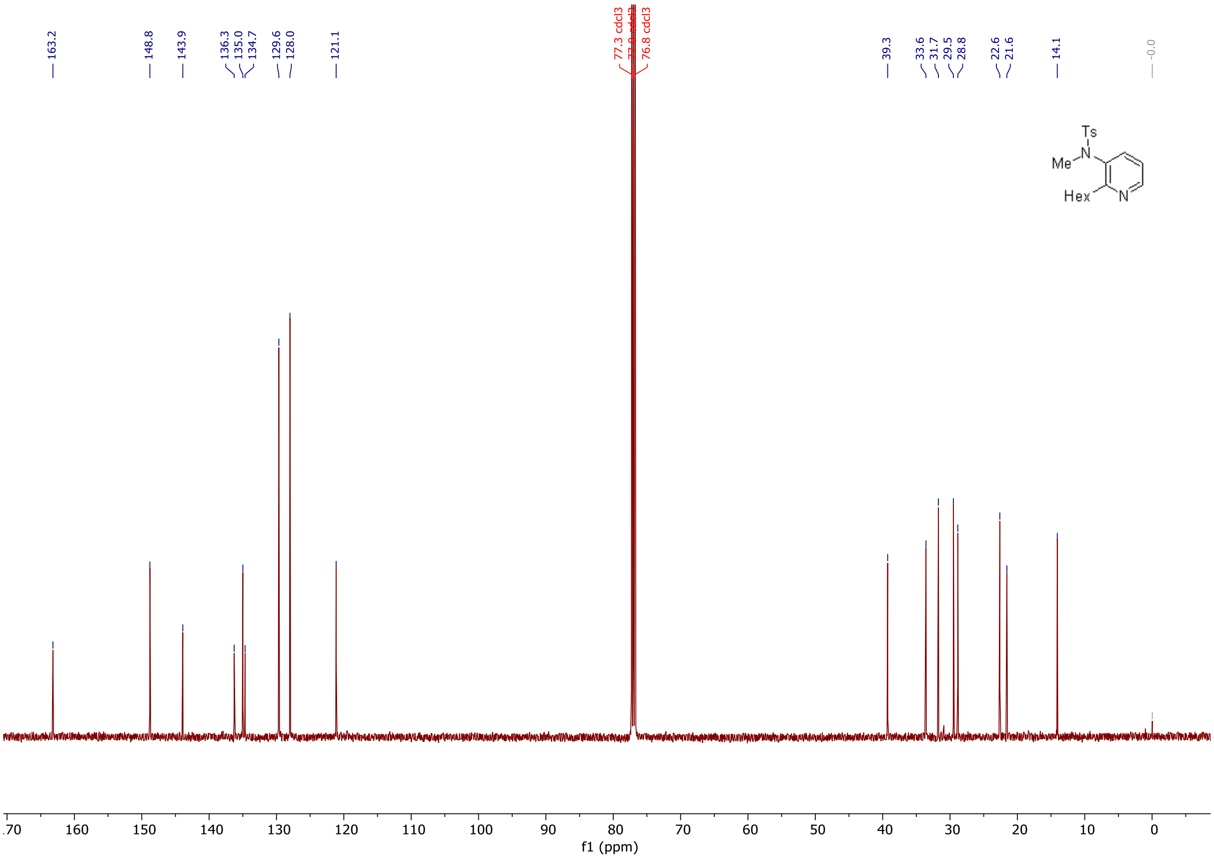

^{1}H NMR spectrum of compound 33a ($CDCl_3$, 298 K)

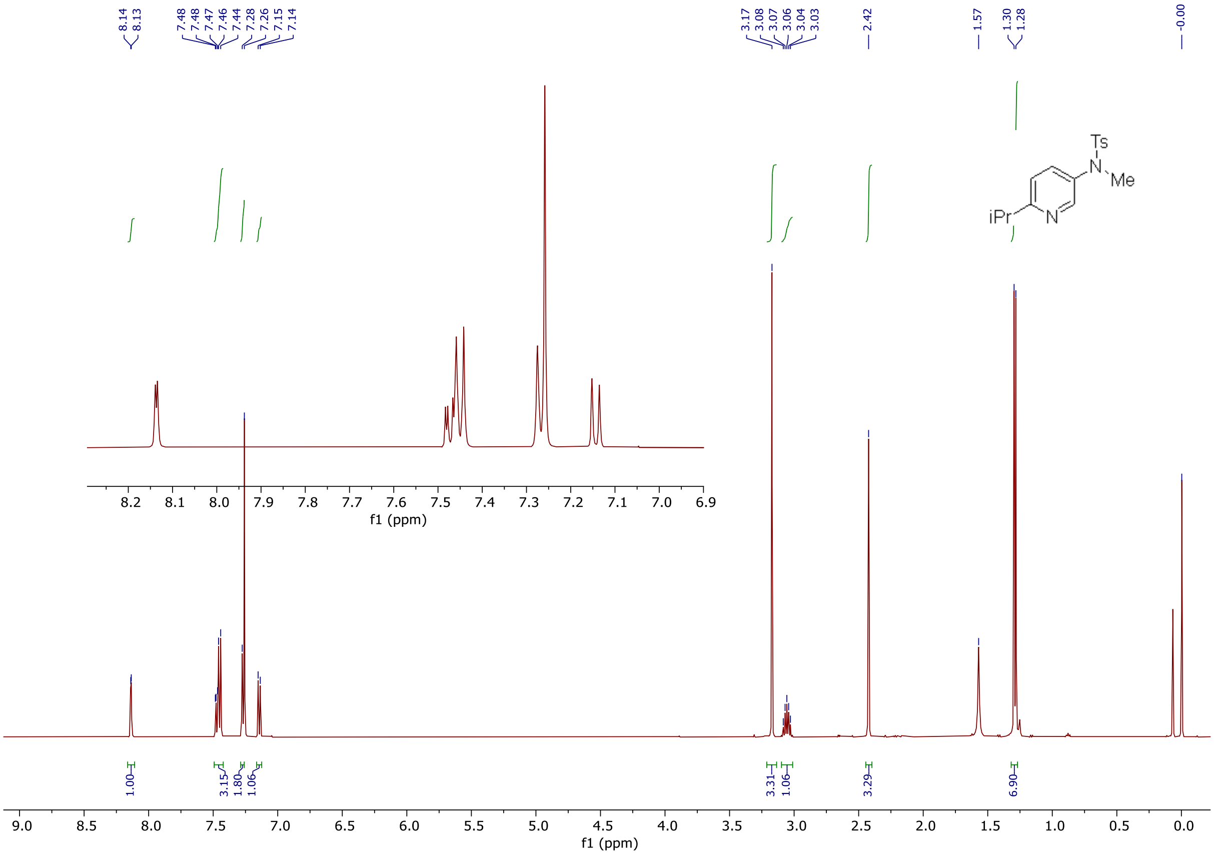

^{13}C NMR spectrum of compound 33a ($CDCl_3$, 298 K)

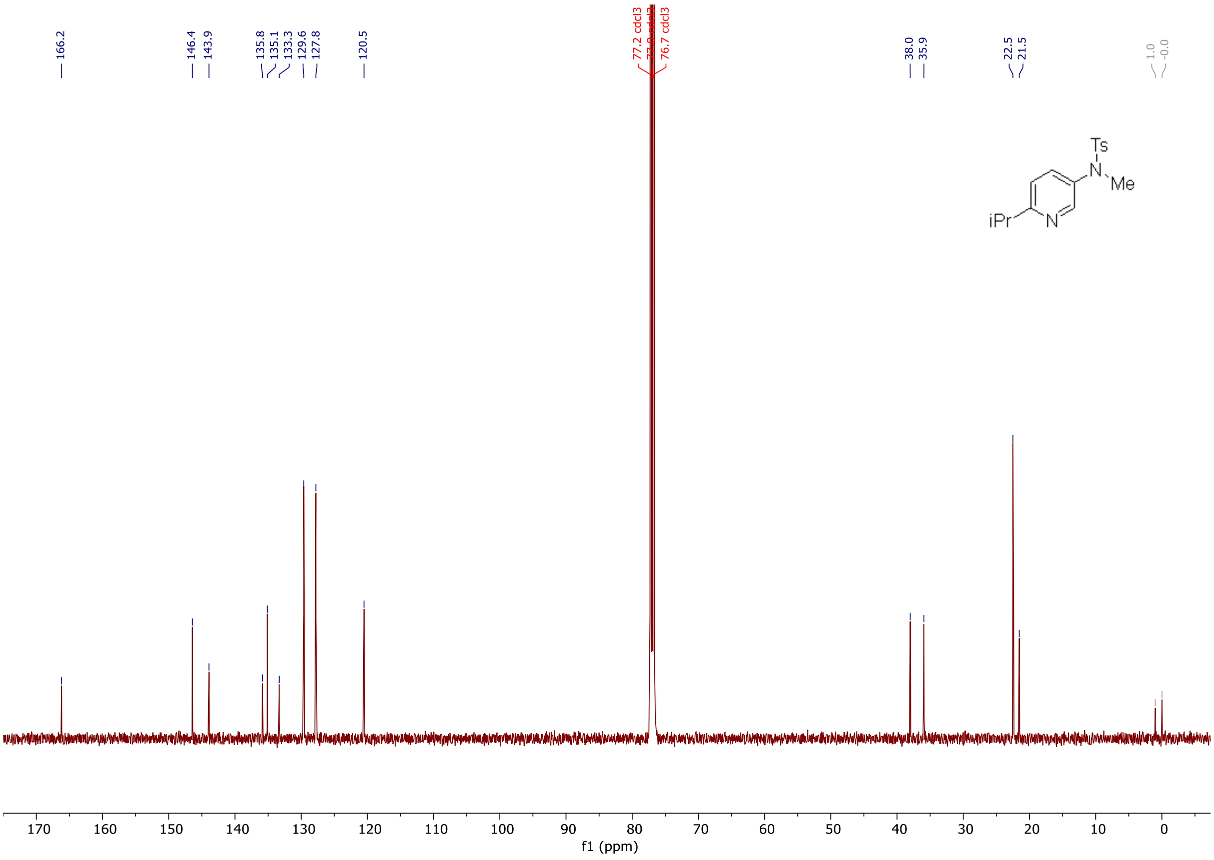

^{1}H NMR spectrum of compound 33b ($CDCl_3$, 298 K)

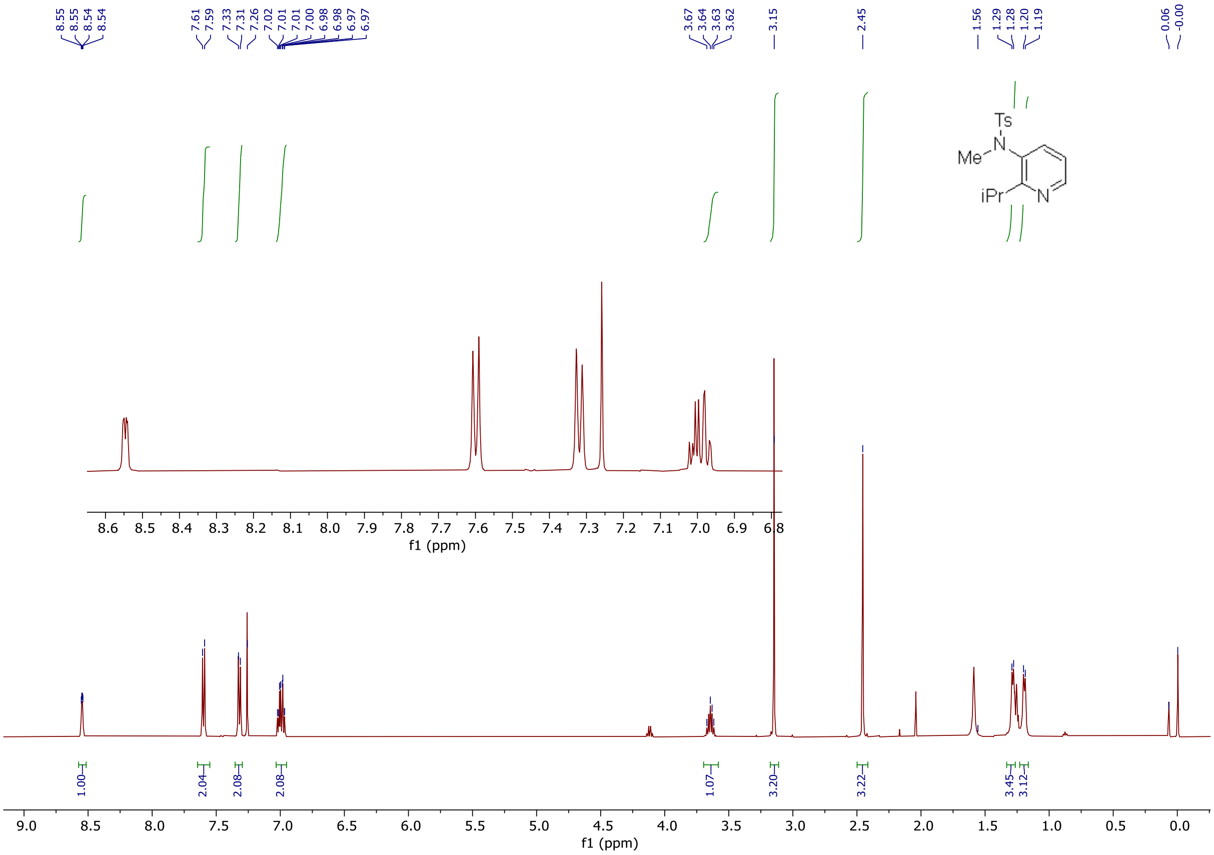

^{13}C NMR spectrum of compound 33b ($CDCl_3$, 298 K)

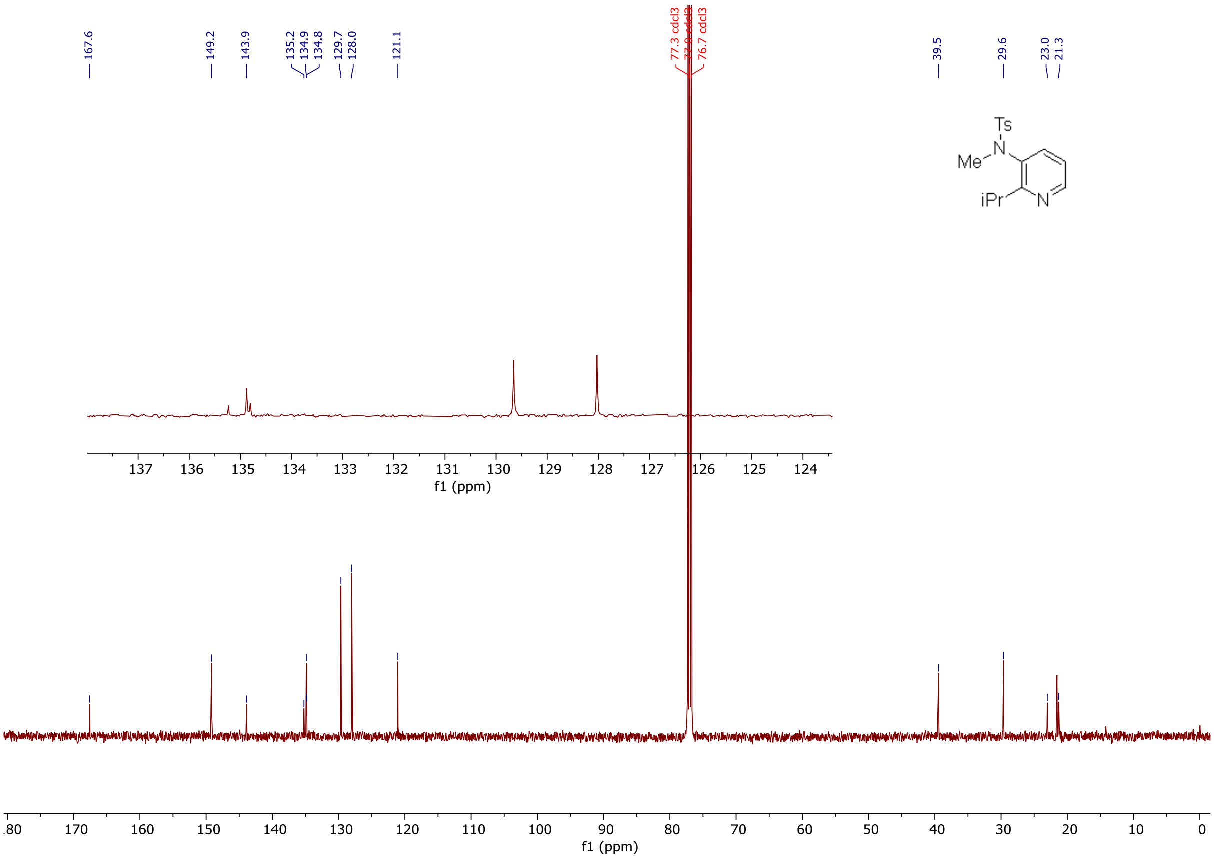

^{1}H NMR spectrum of compound 34b ($CDCl_3$, 298 K)

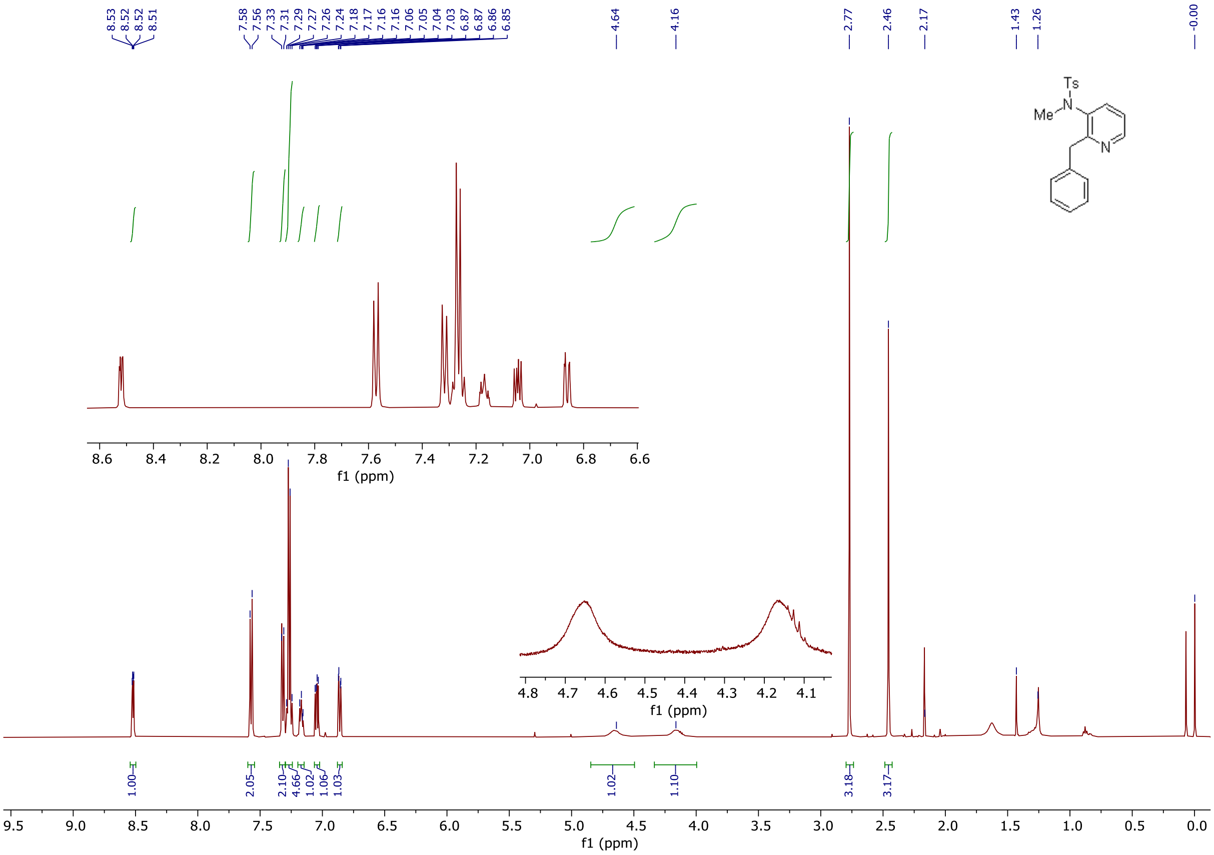

^{13}C NMR spectrum of compound 34b ($CDCl_3$, 298 K)

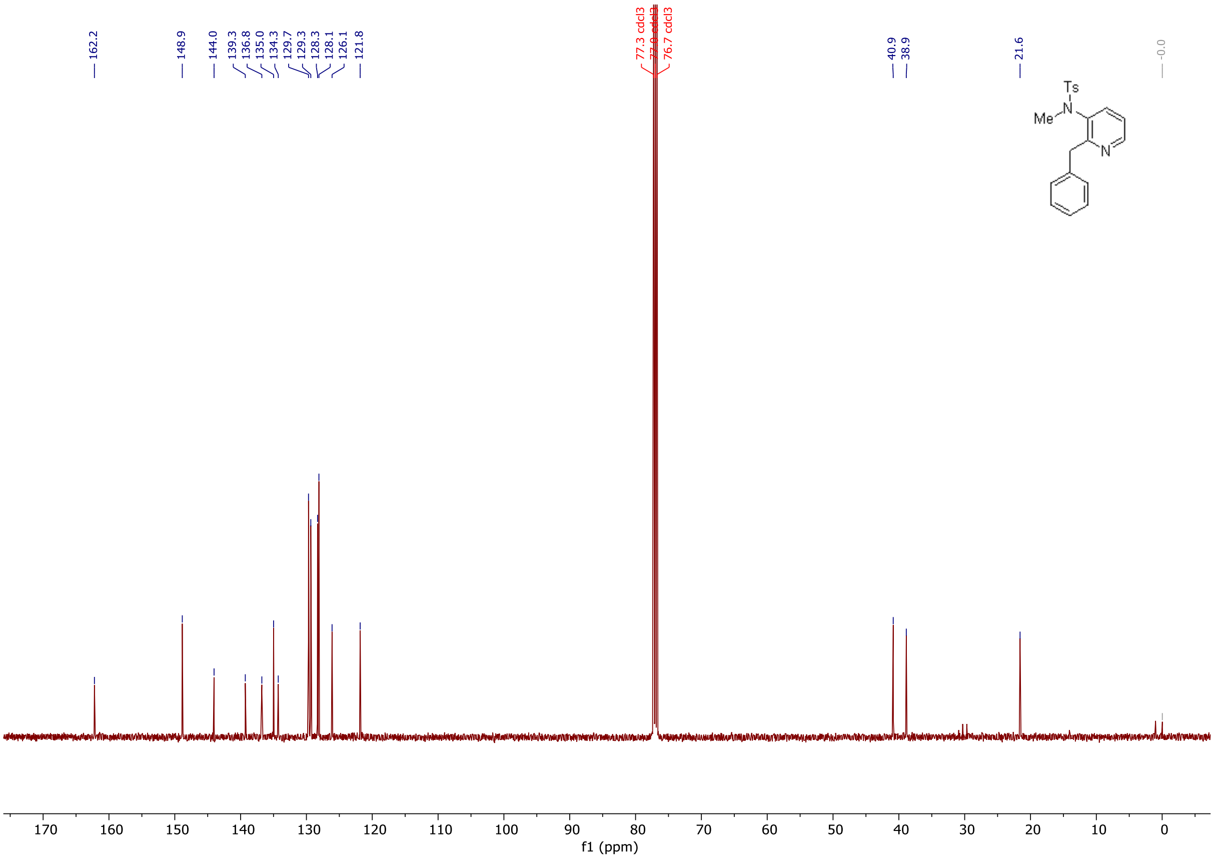

^{1}H NMR spectrum of compound 35a ($CDCl_3$, 298 K)

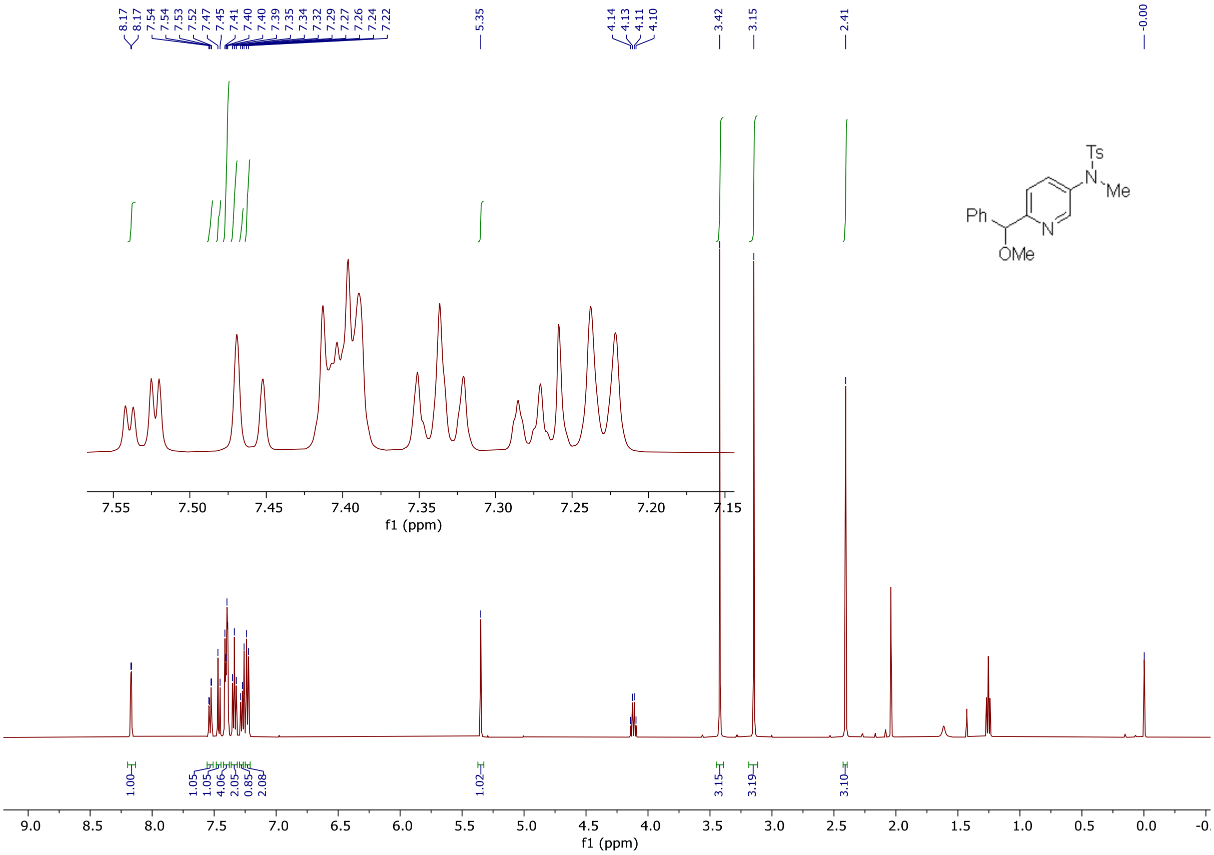

^{13}C NMR spectrum of compound 35a ($CDCl_3$, 298 K)

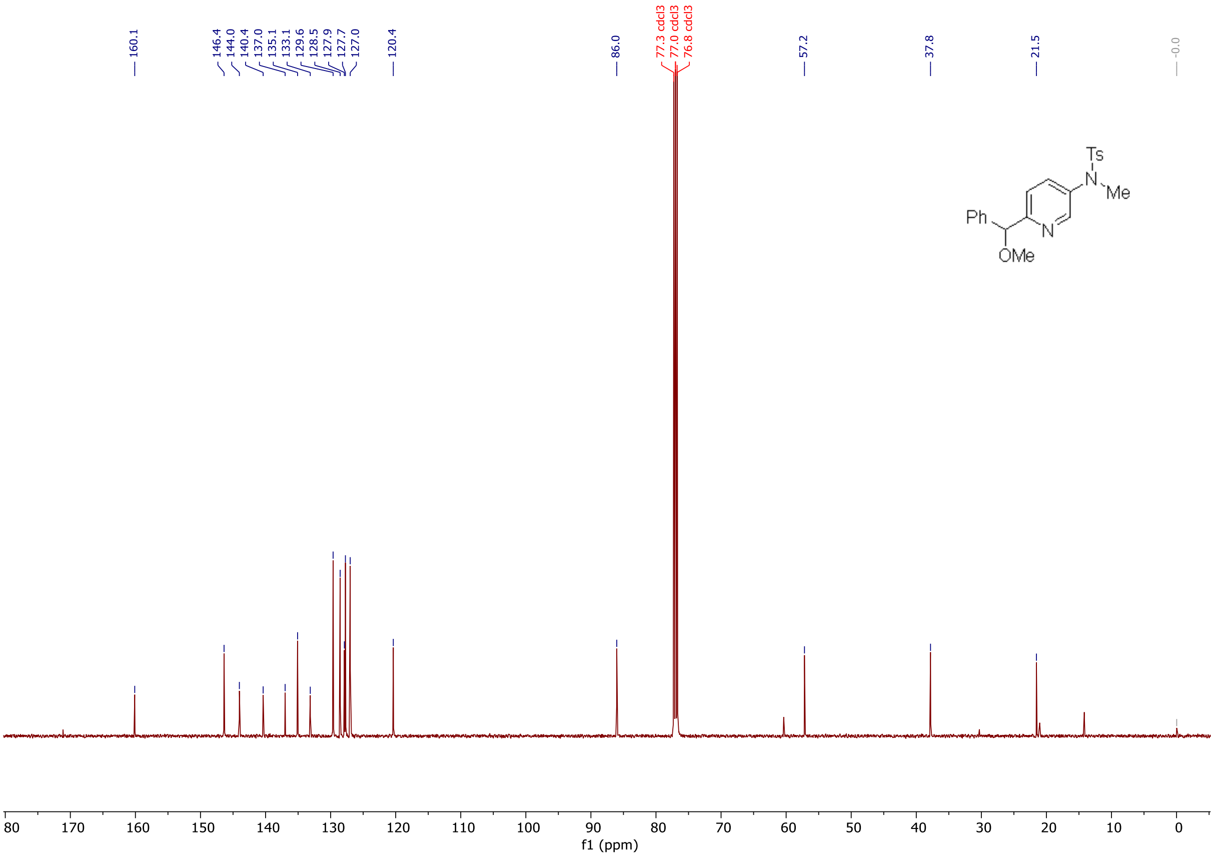

^{1}H NMR spectrum of compound 35b ($CDCl_3$, 298 K)

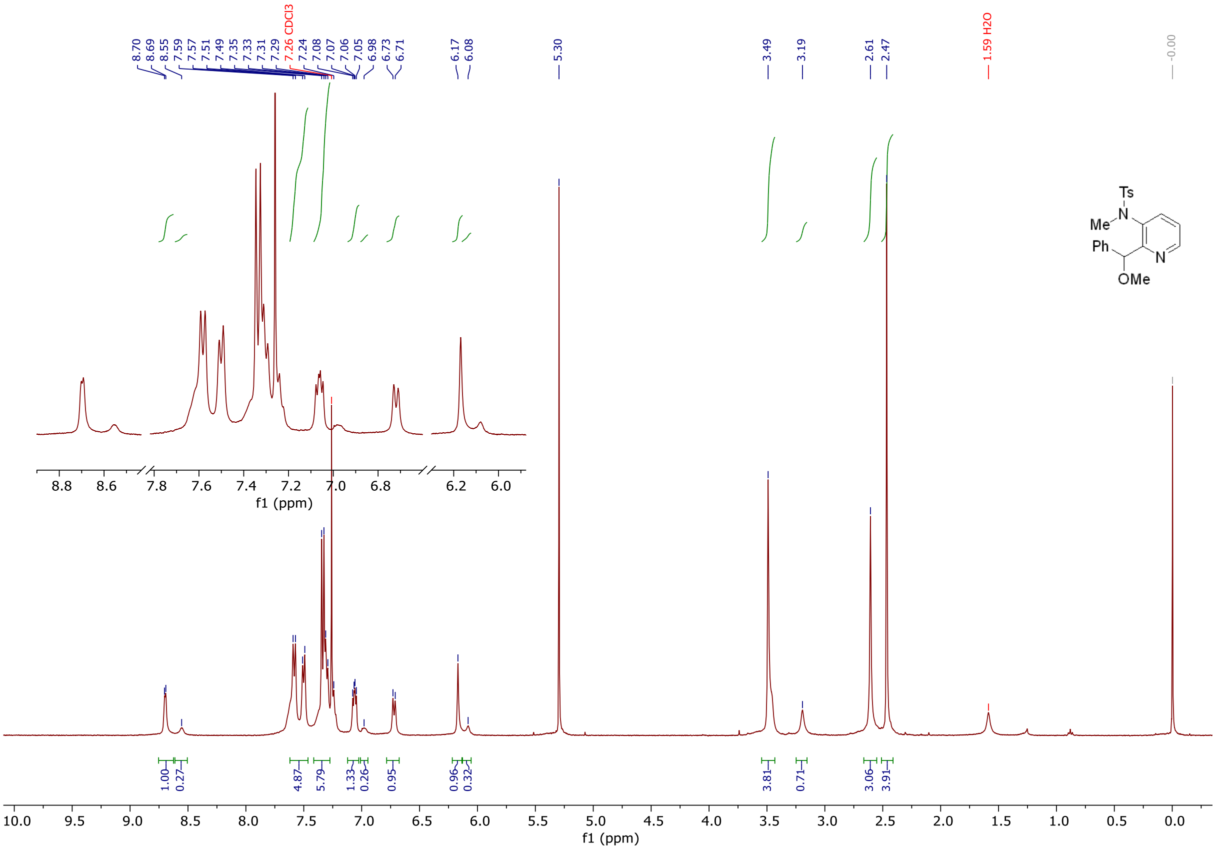

^{13}C NMR spectrum of compound 35b ($CDCl_3$, 298 K)

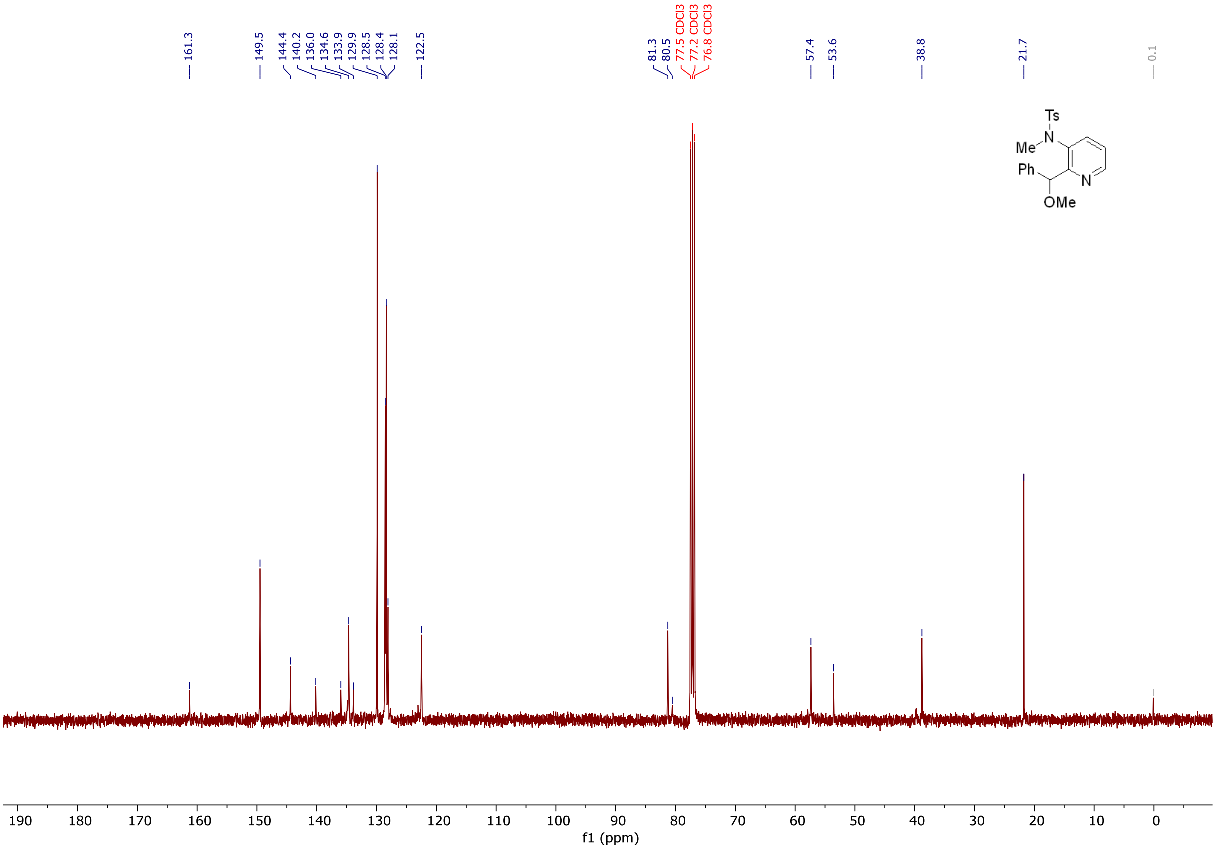

^{1}H NMR spectrum of compound 35b (CD_3OD, 298 K)

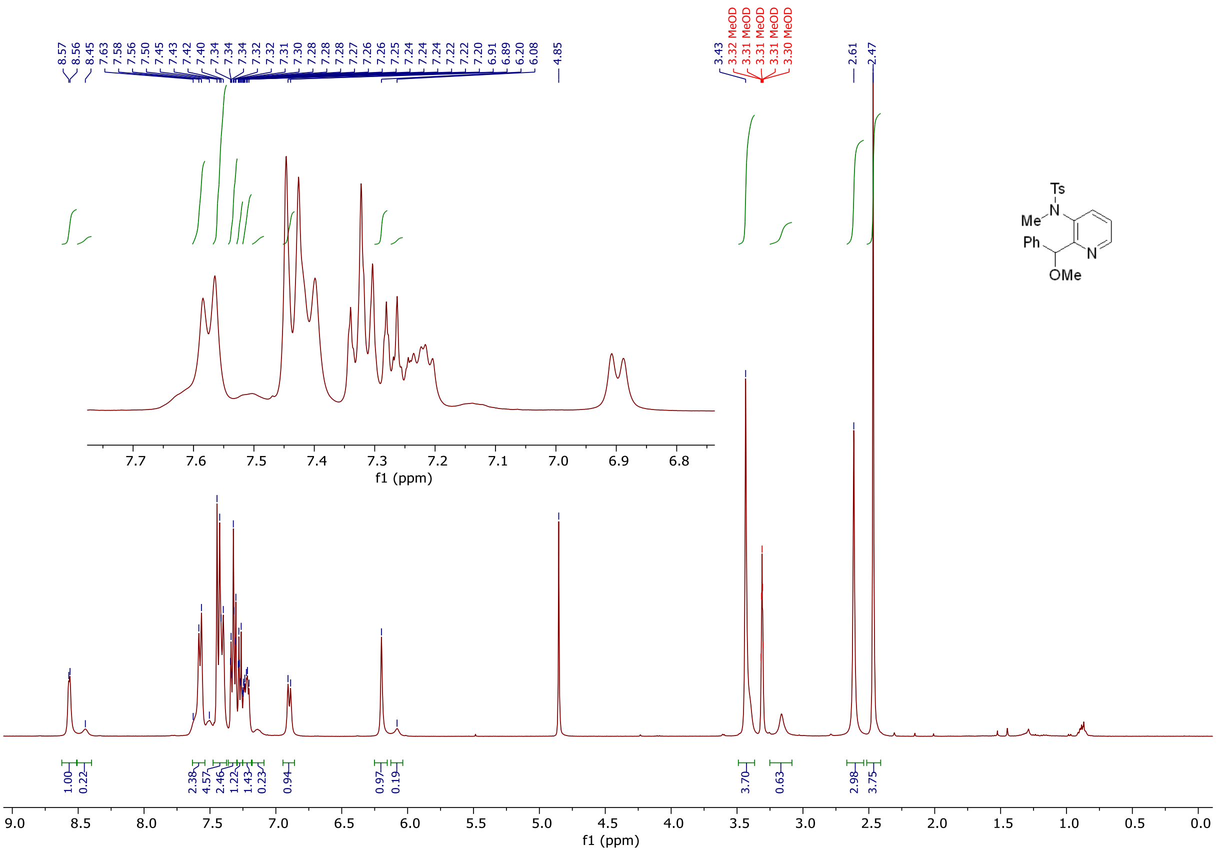

^{13}C NMR spectrum of compound 35b (CD_3OD, 298 K)

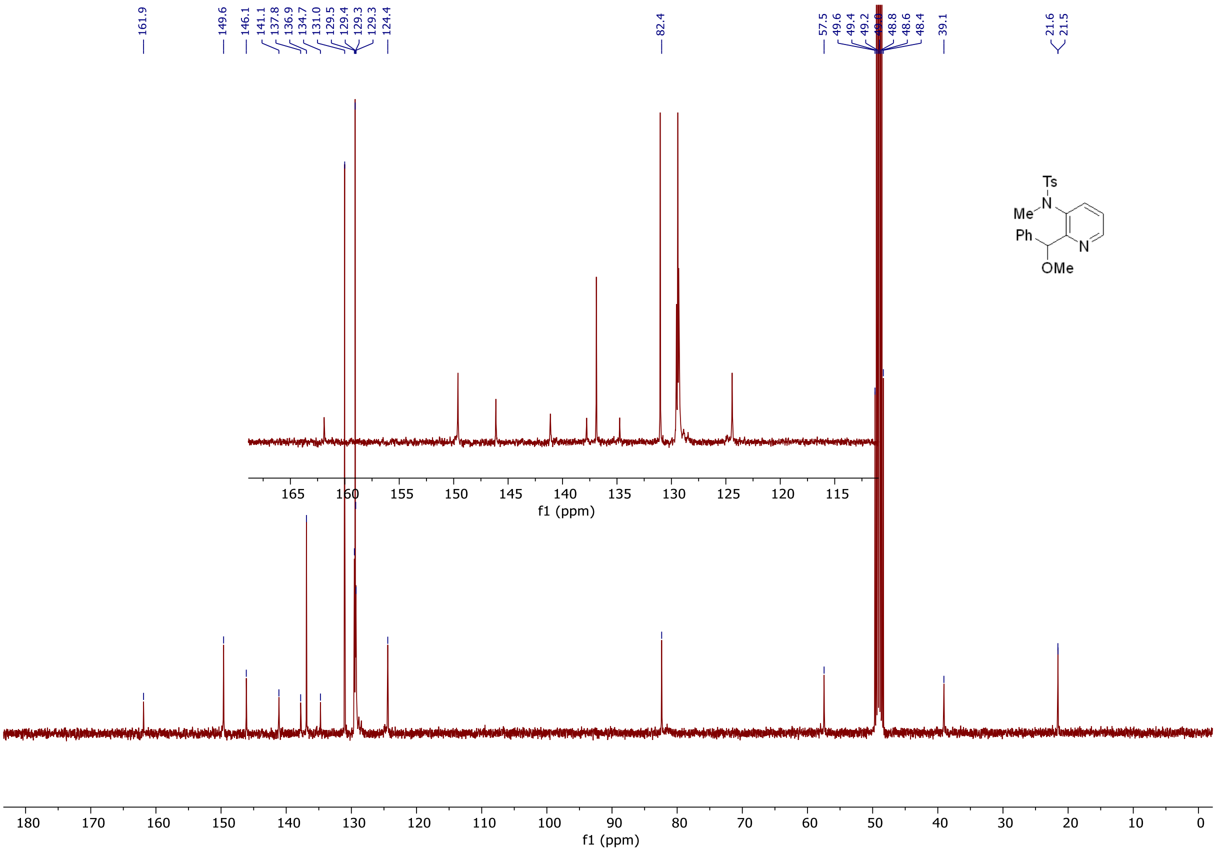

^{1}H NMR spectrum of compound 35b (DMSO-d_6, 298 K)

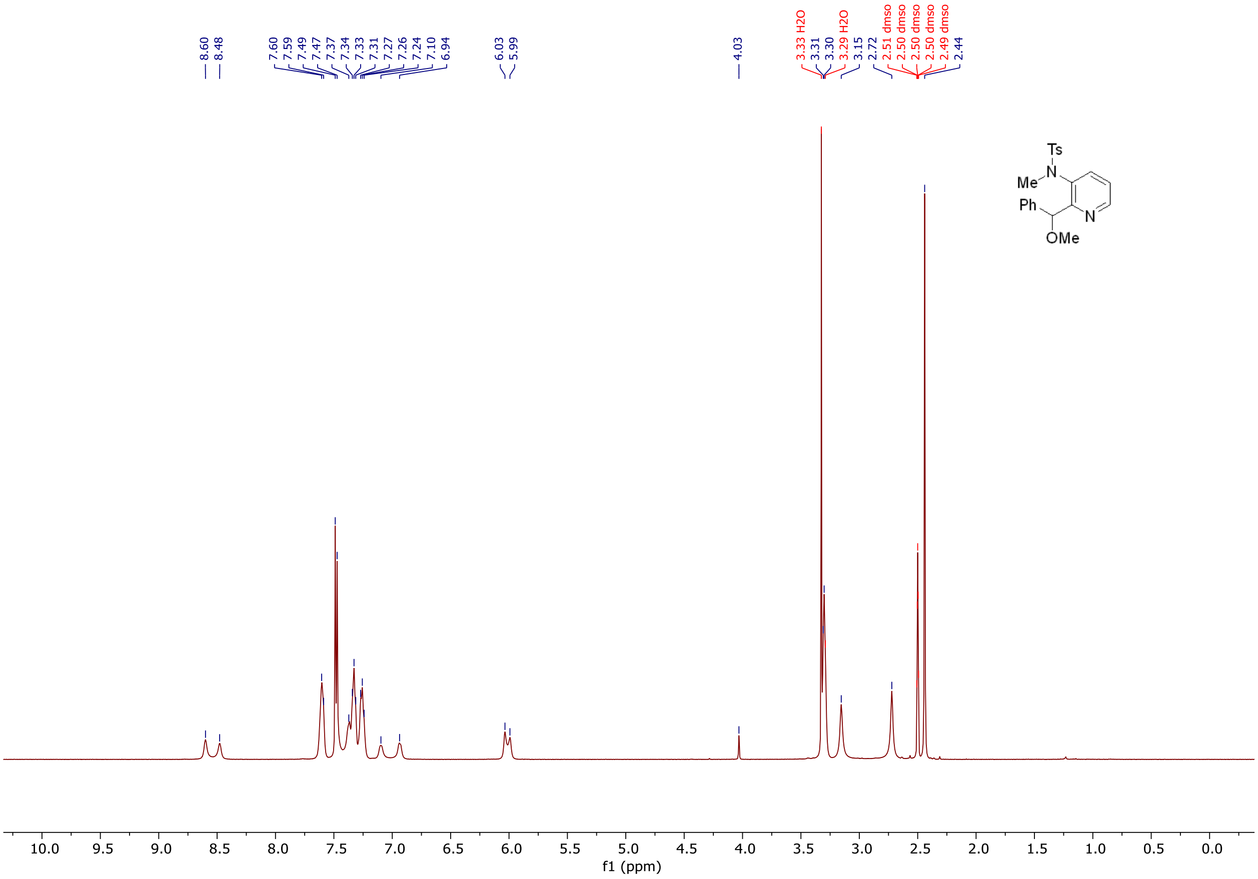

^{1}H NMR spectrum of compound 35b (DMSO-d_6, 353 K)

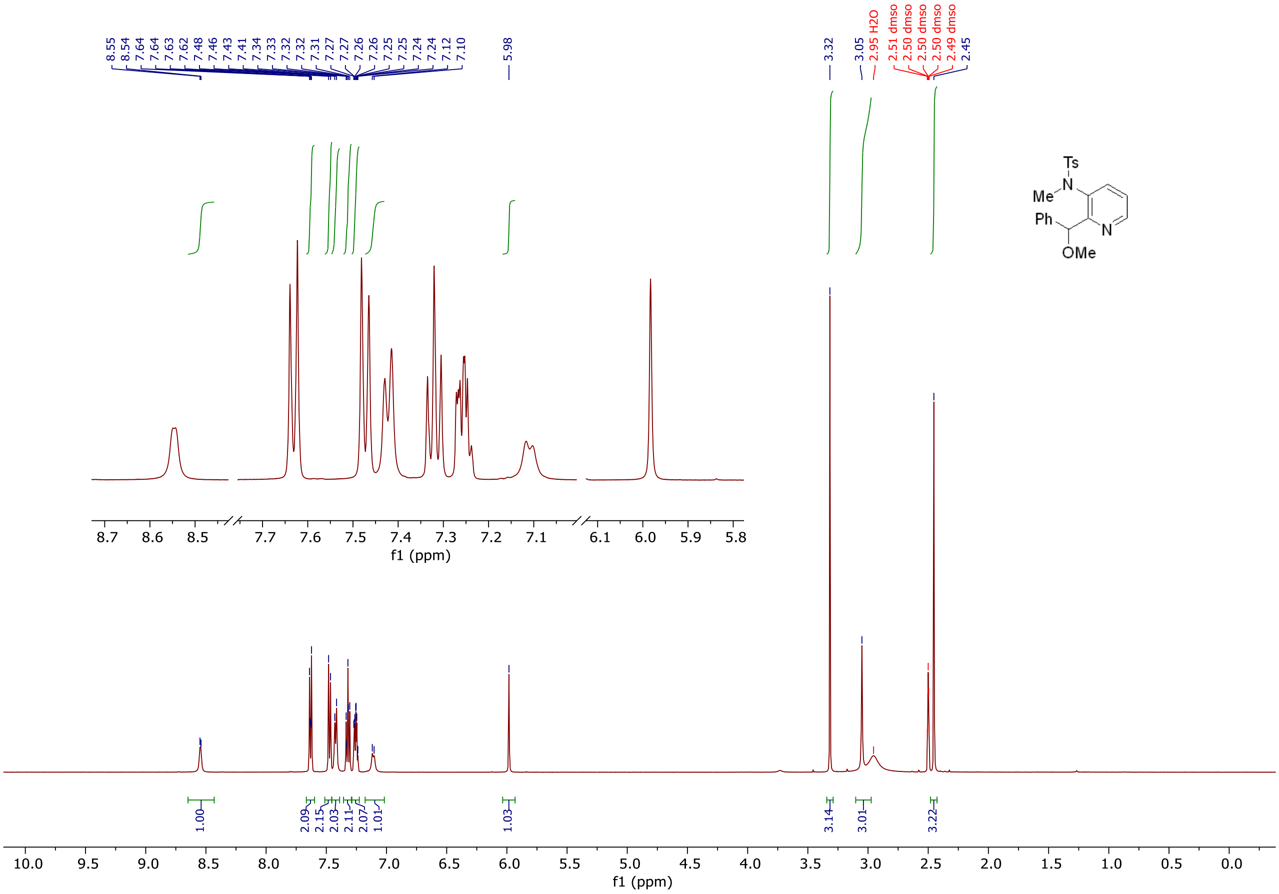

^{13}C NMR spectrum of compound 35b (DMSO-d$_6$, 353 K)

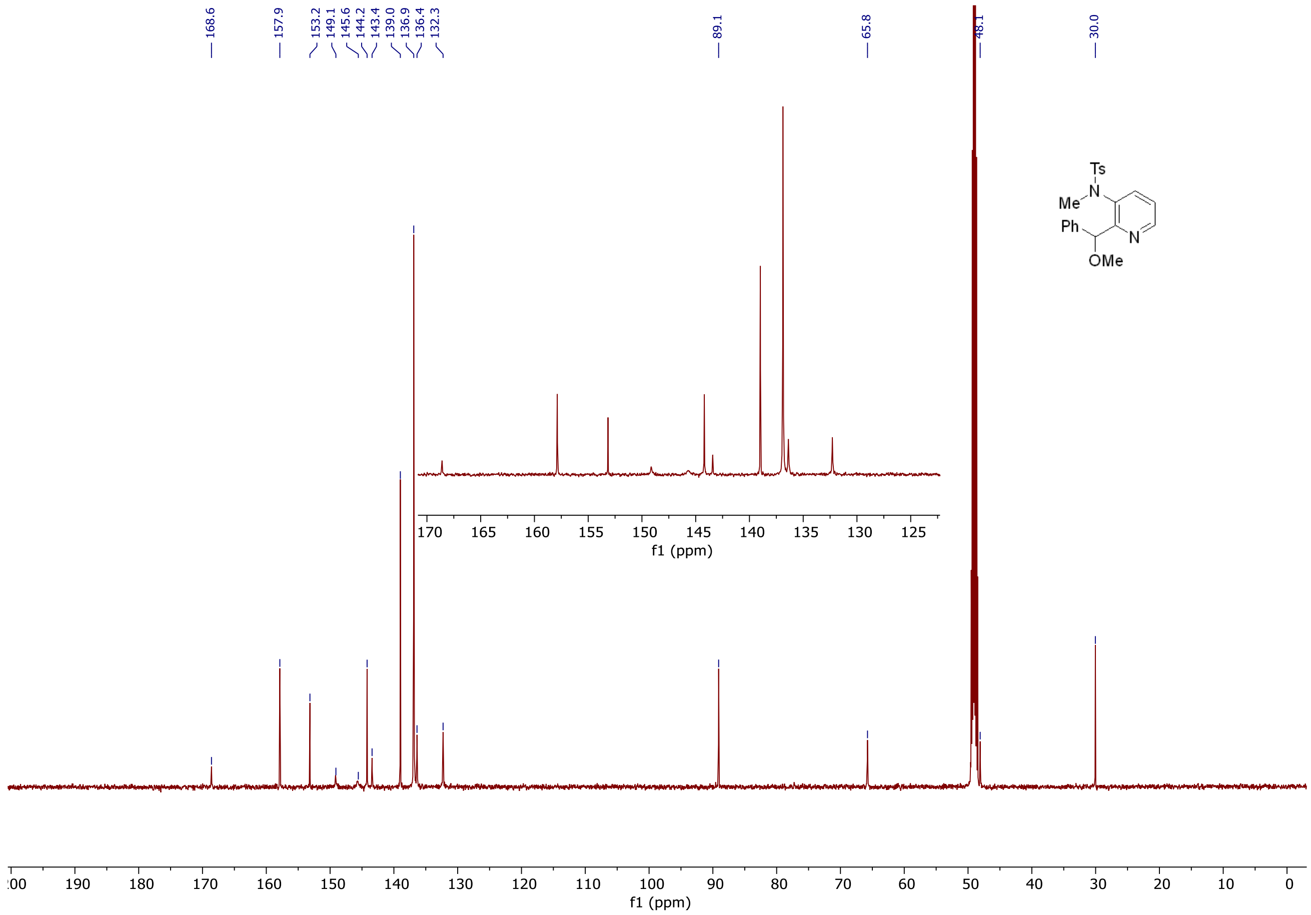

LR-MS chromatogram of compound 35b

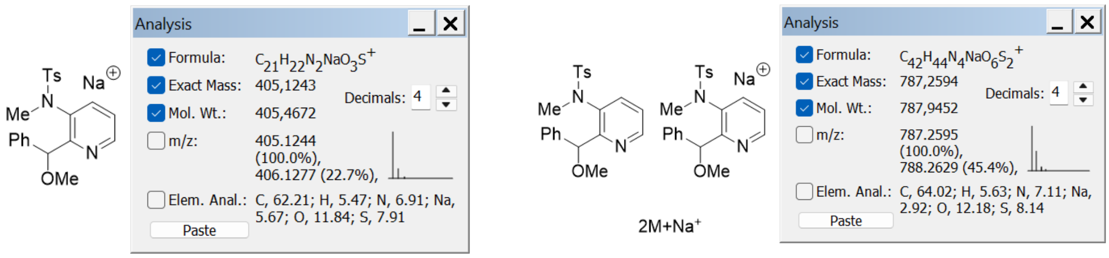

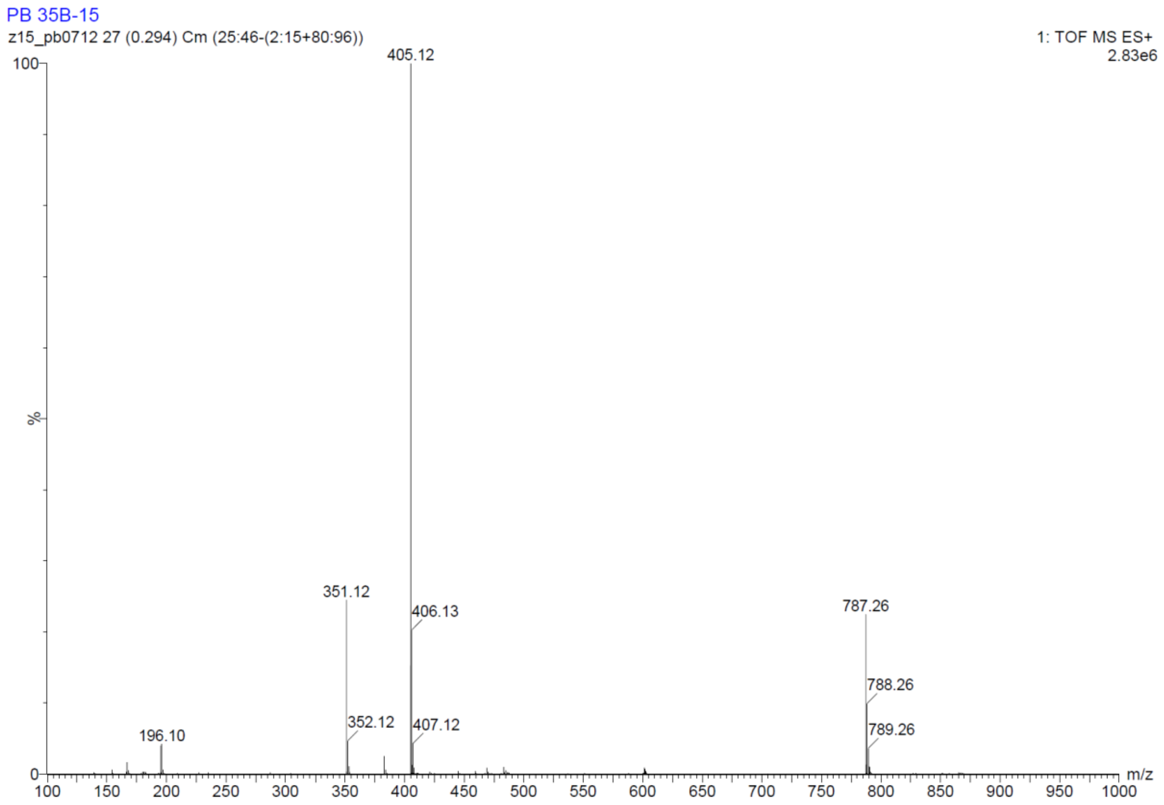

GC-FID chromatogram of compound 35b

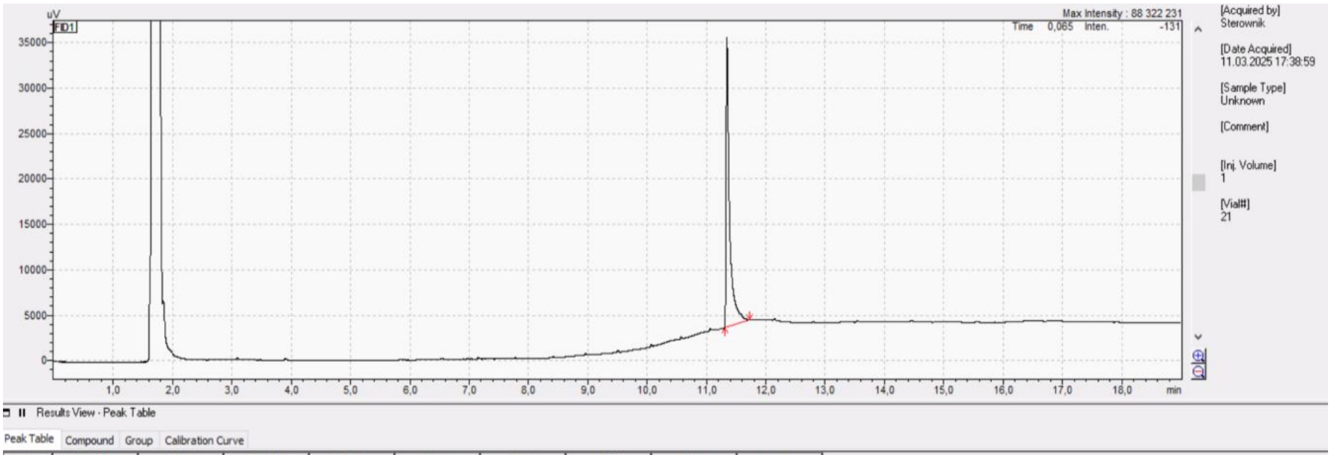

Peak#	Ret. Time	Area	Height	Mark	Conc.	Unit	ID#	Name	Area%
1	11,345	130842	31542		100,000				100,000
Total		130842	31542		100,000				100,000

^{1}H NMR spectrum of salt Py-salt (DMSO-d_6, 298 K)

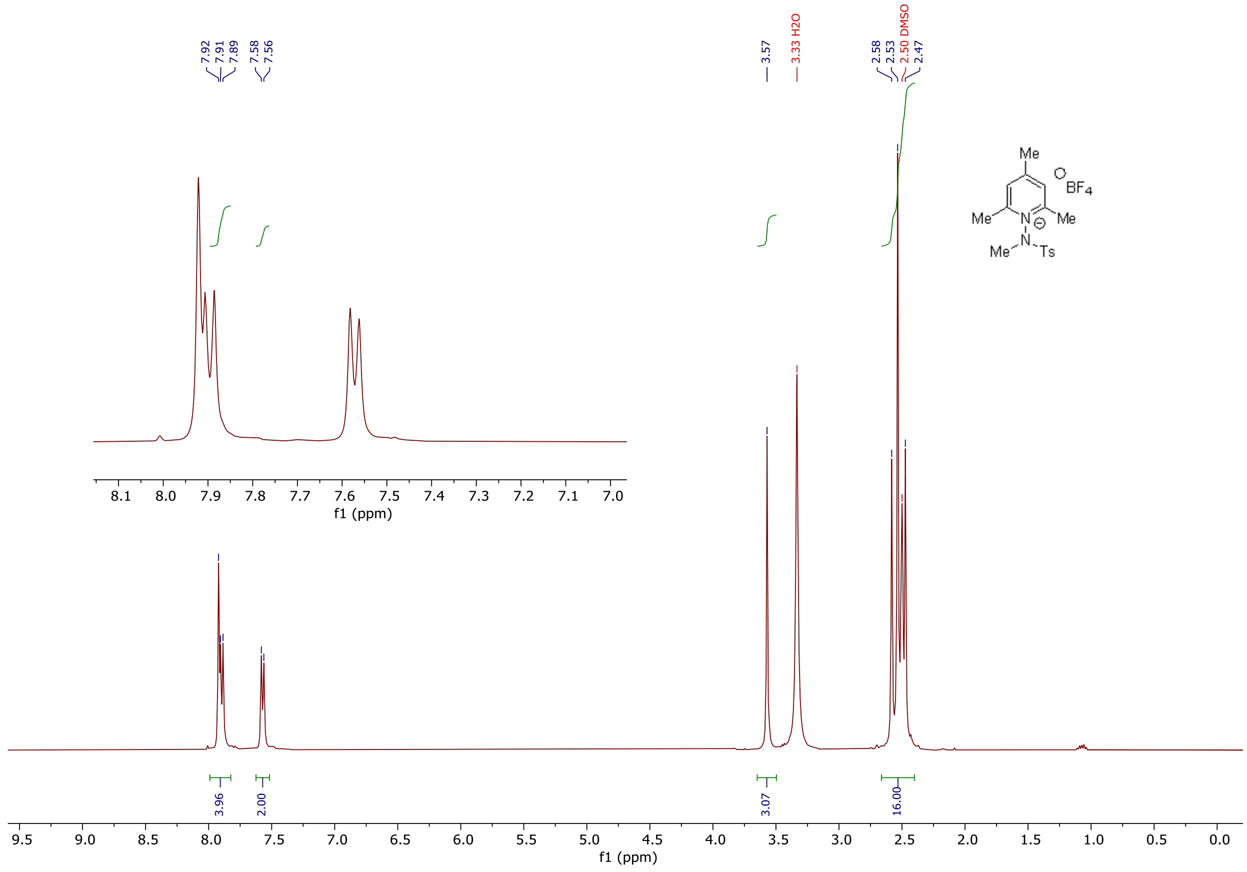

^{1}H NMR spectrum of compound 36 ($CDCl_3$, 298 K)

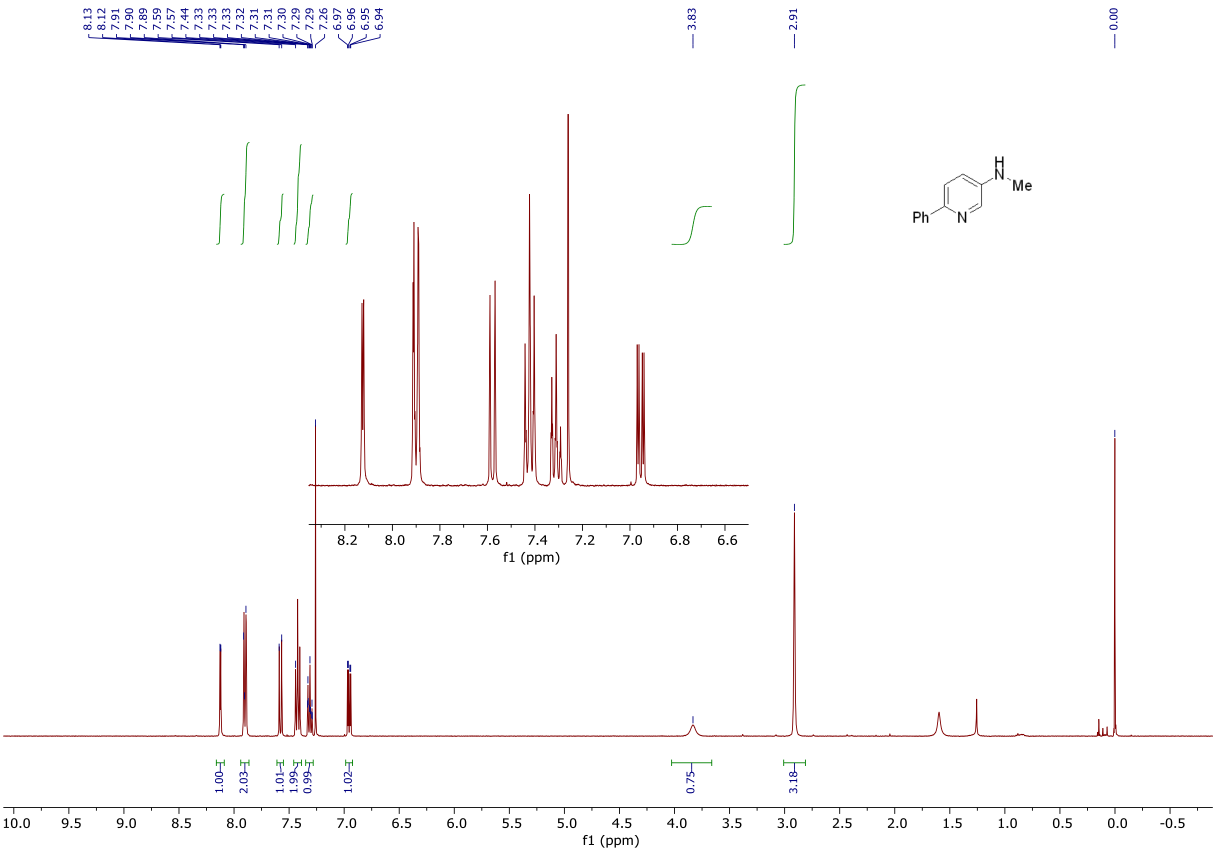

^{13}C NMR spectrum of compound 36 ($CDCl_3$, 298 K)

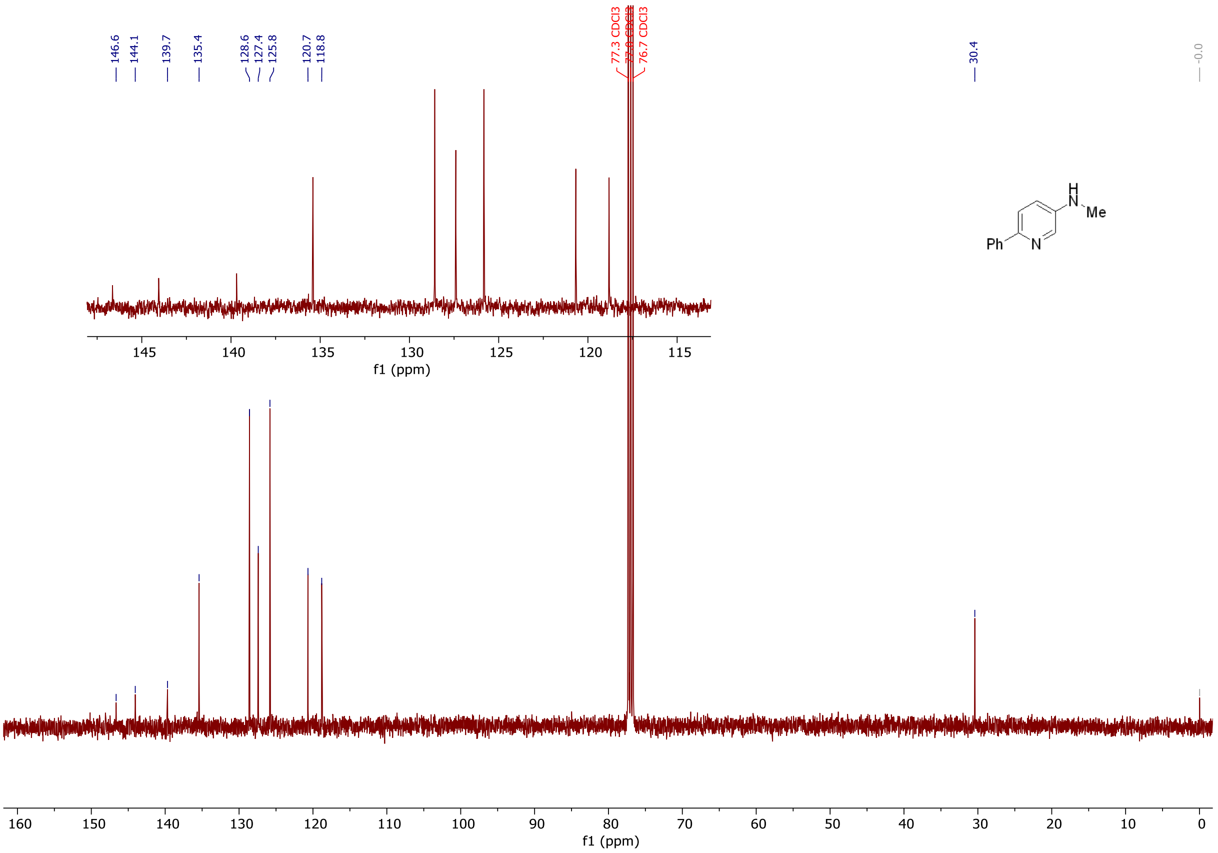

^{1}H NMR spectrum of compound 37 ($CDCl_3$, 298 K)

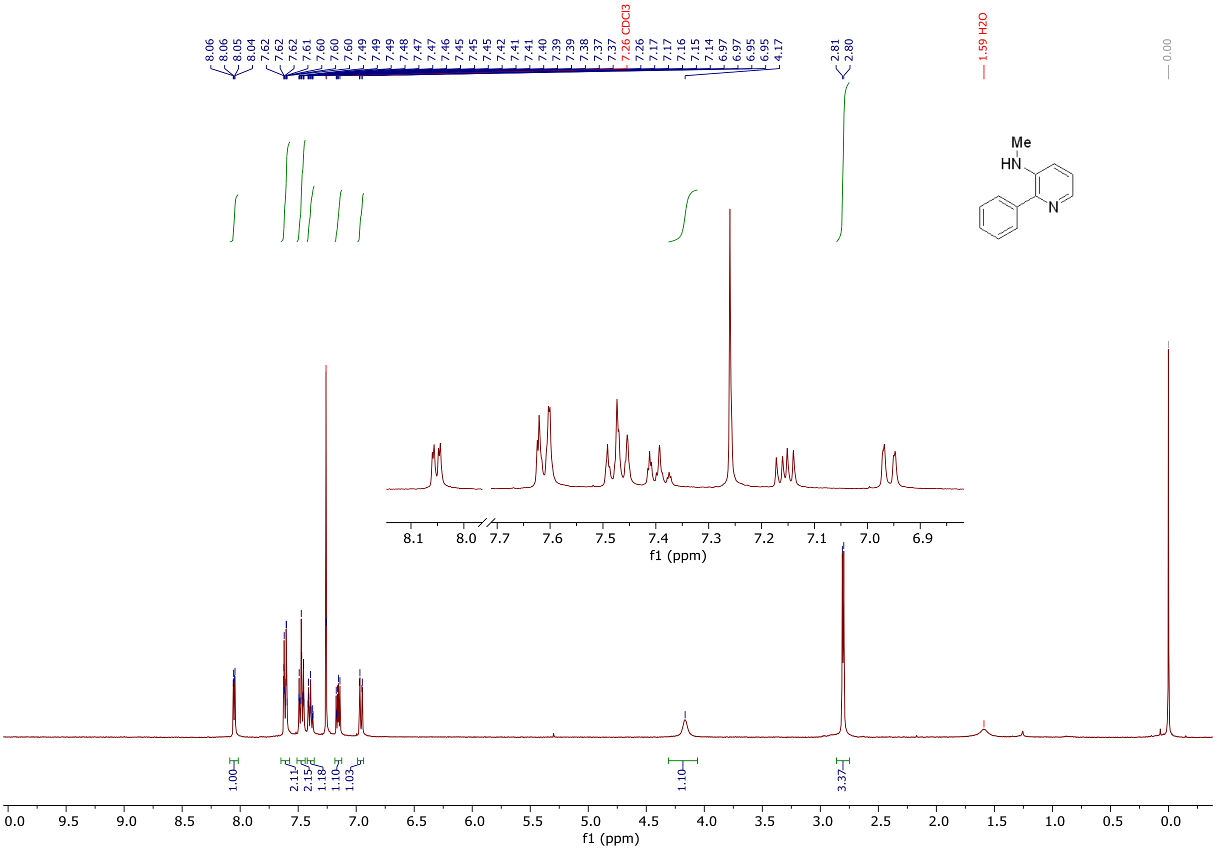

^{13}C NMR spectrum of compound 37 ($CDCl_3$, 298 K)

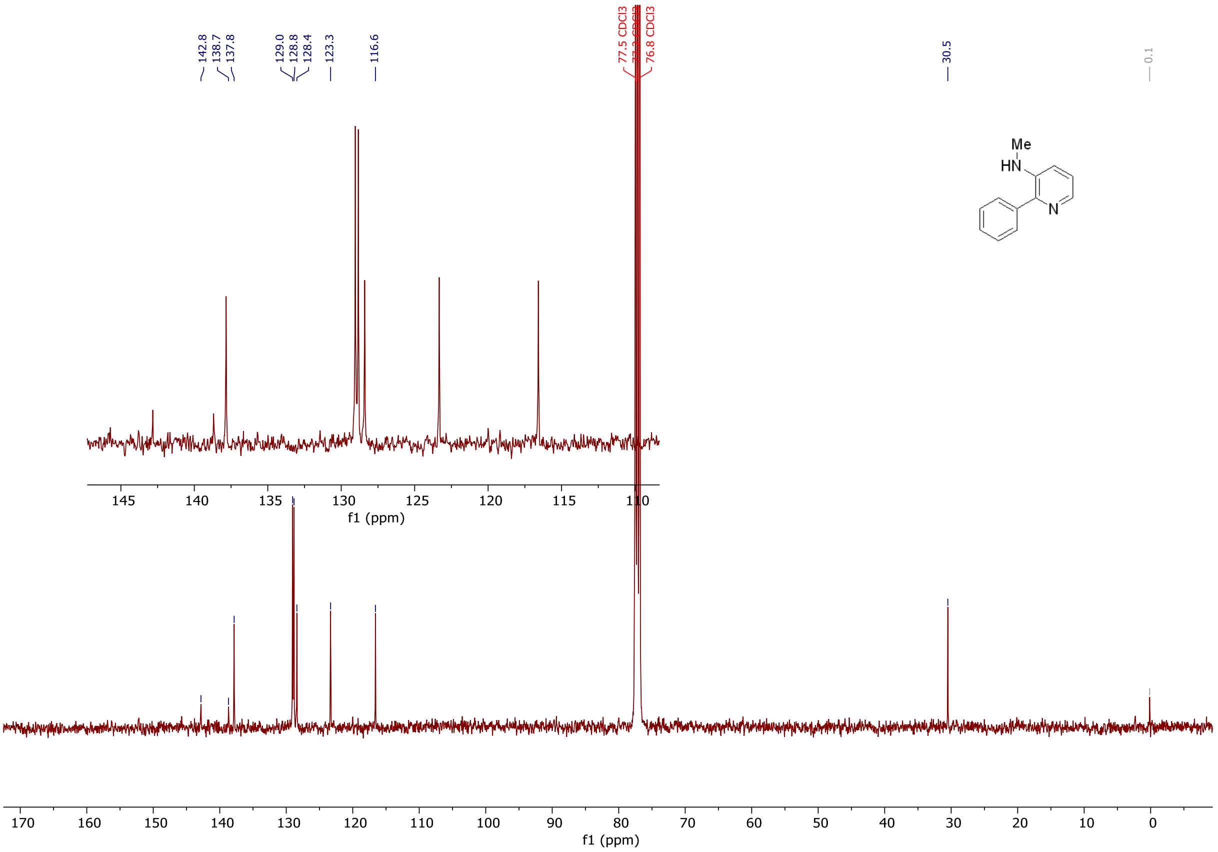

^{1}H NMR spectrum of compound 38 ($CDCl_3$, 298 K)

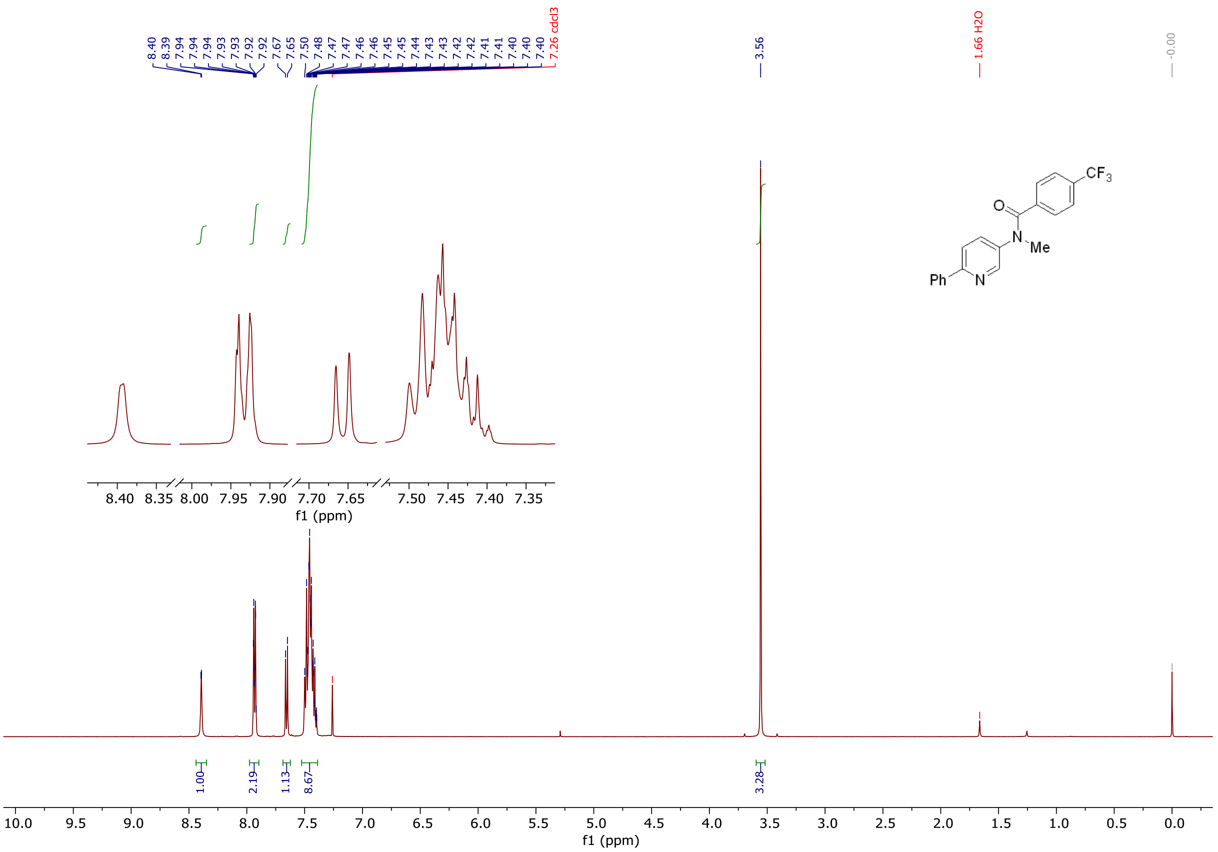

^{13}C NMR spectrum of compound 38 ($CDCl_3$, 298 K)

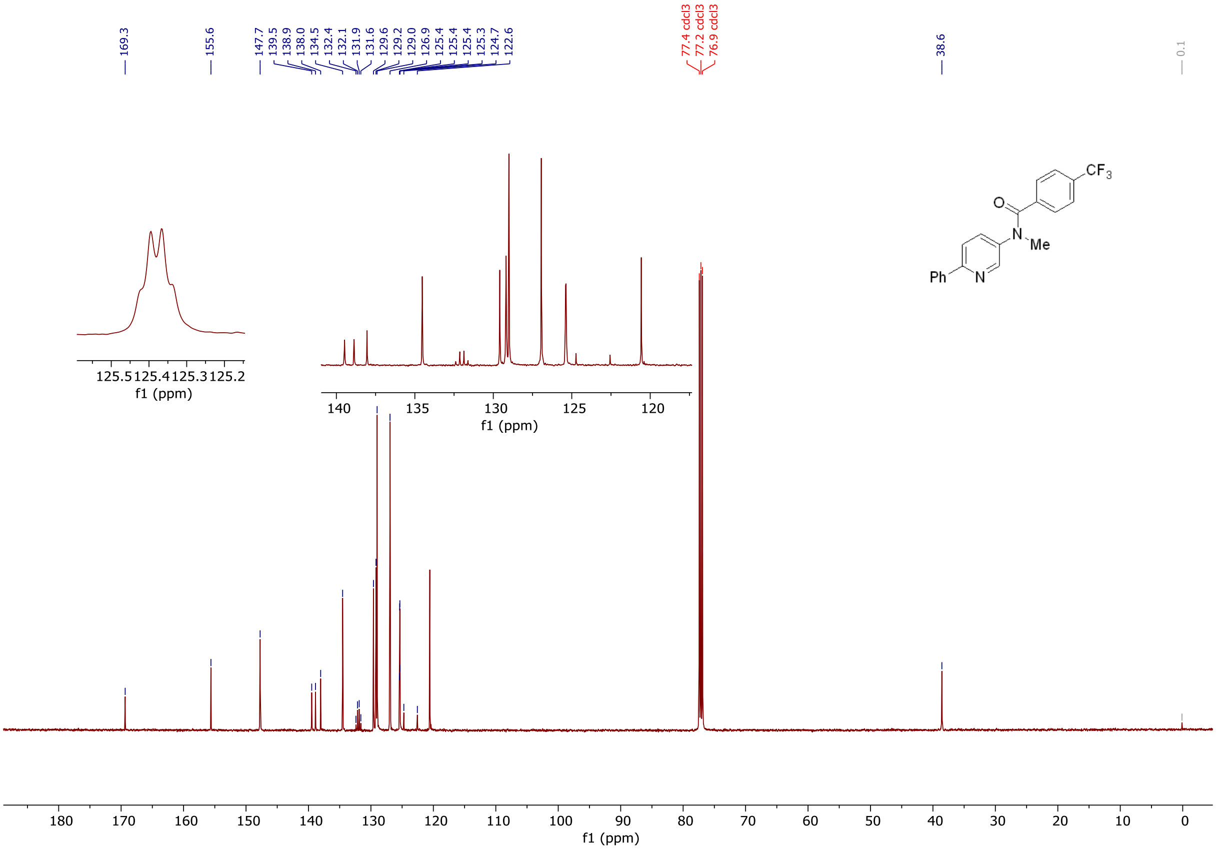

^{19}F NMR spectrum of compound 38 ($CDCl_3$, 298 K)

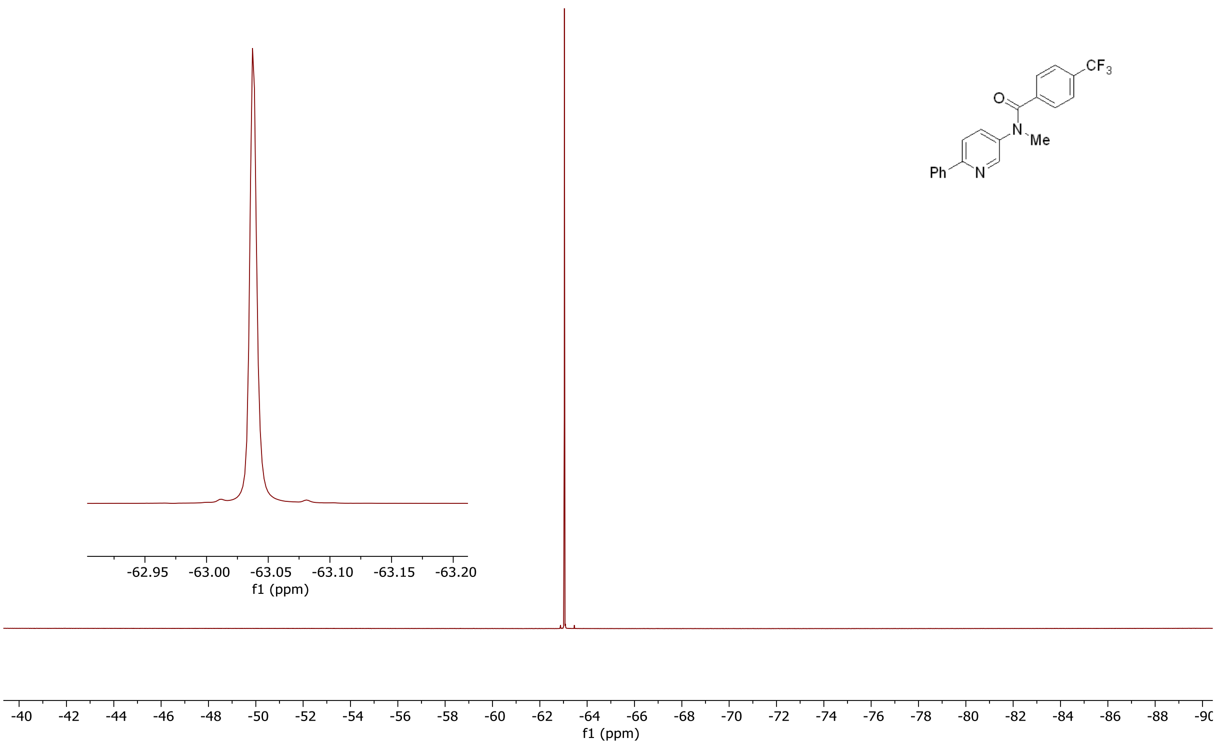

^{1}H NMR spectrum of compound 39 ($CDCl_3$, 298 K)

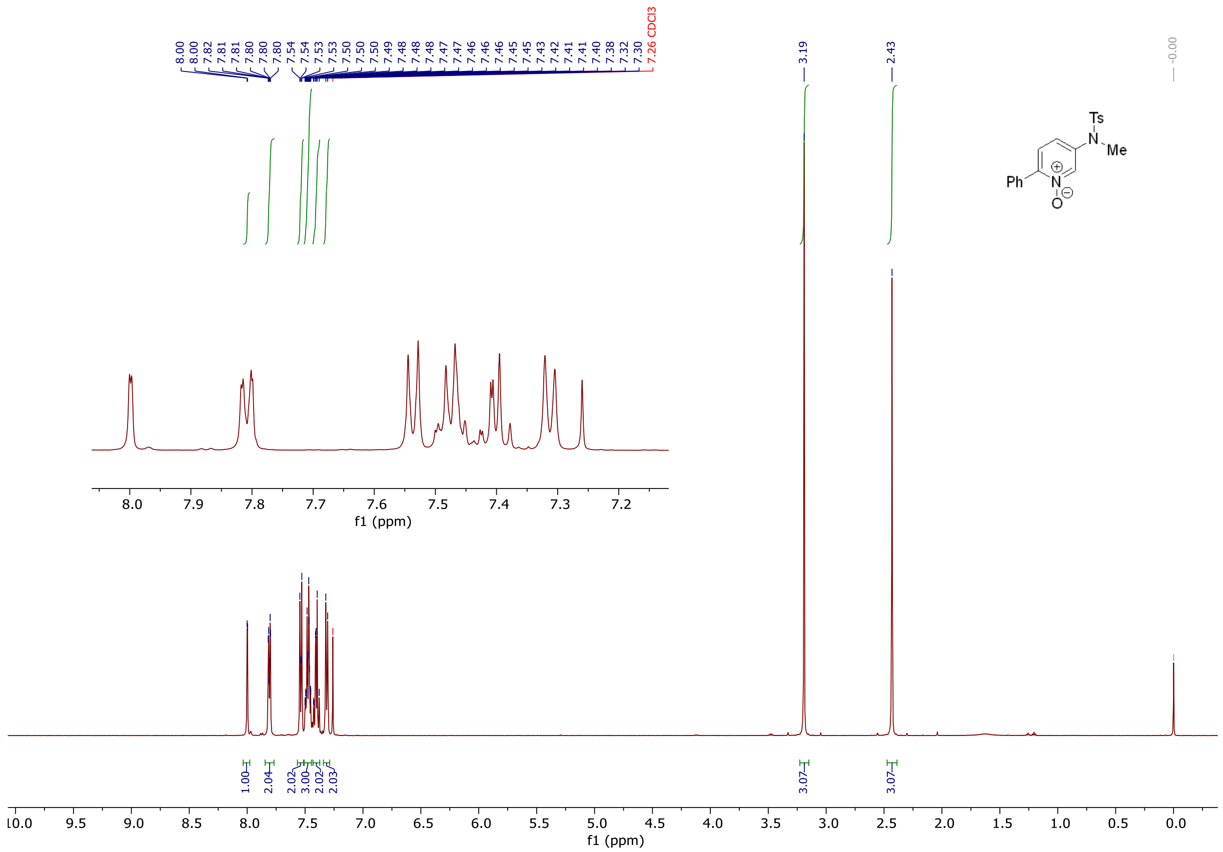

^{13}C NMR spectrum of compound 39 ($CDCl_3$, 298 K)

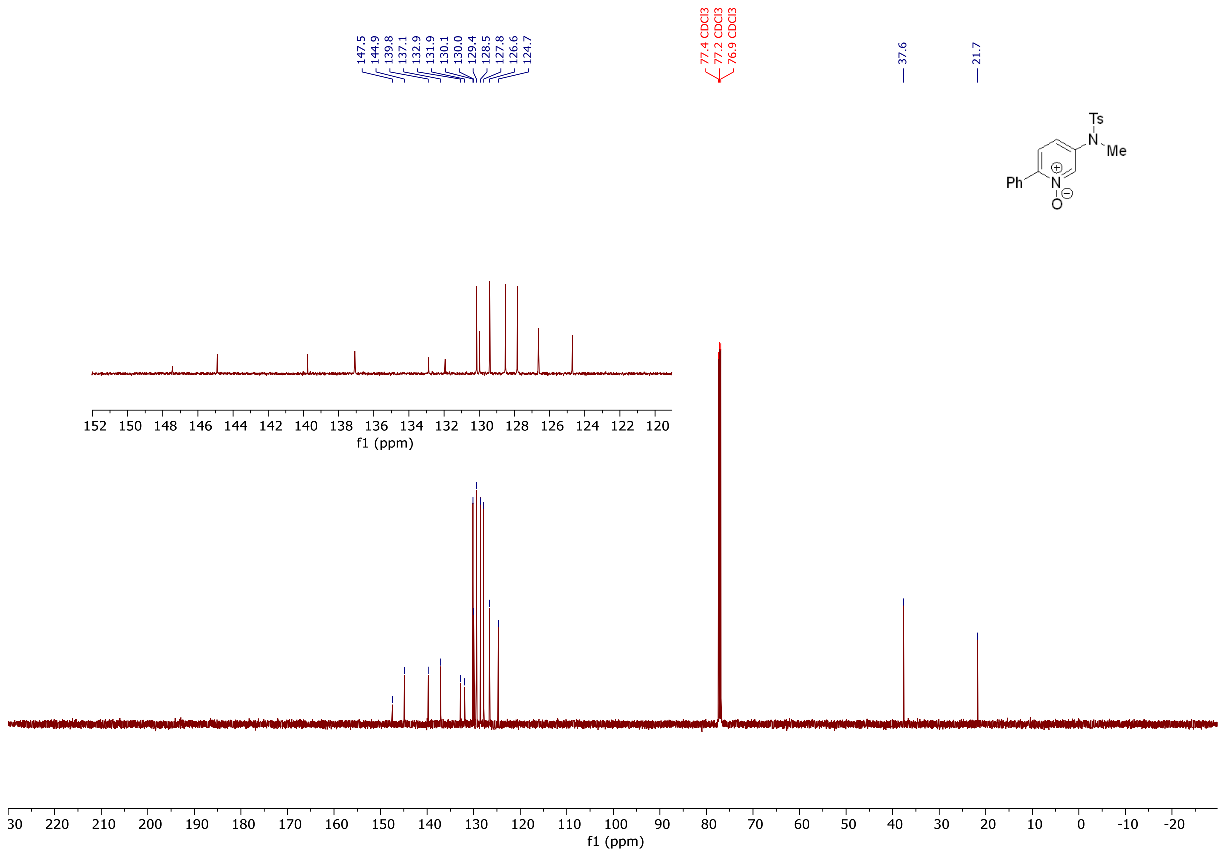

^{1}H NMR spectrum of compound 40 (DMSO-d_6, 298 K)

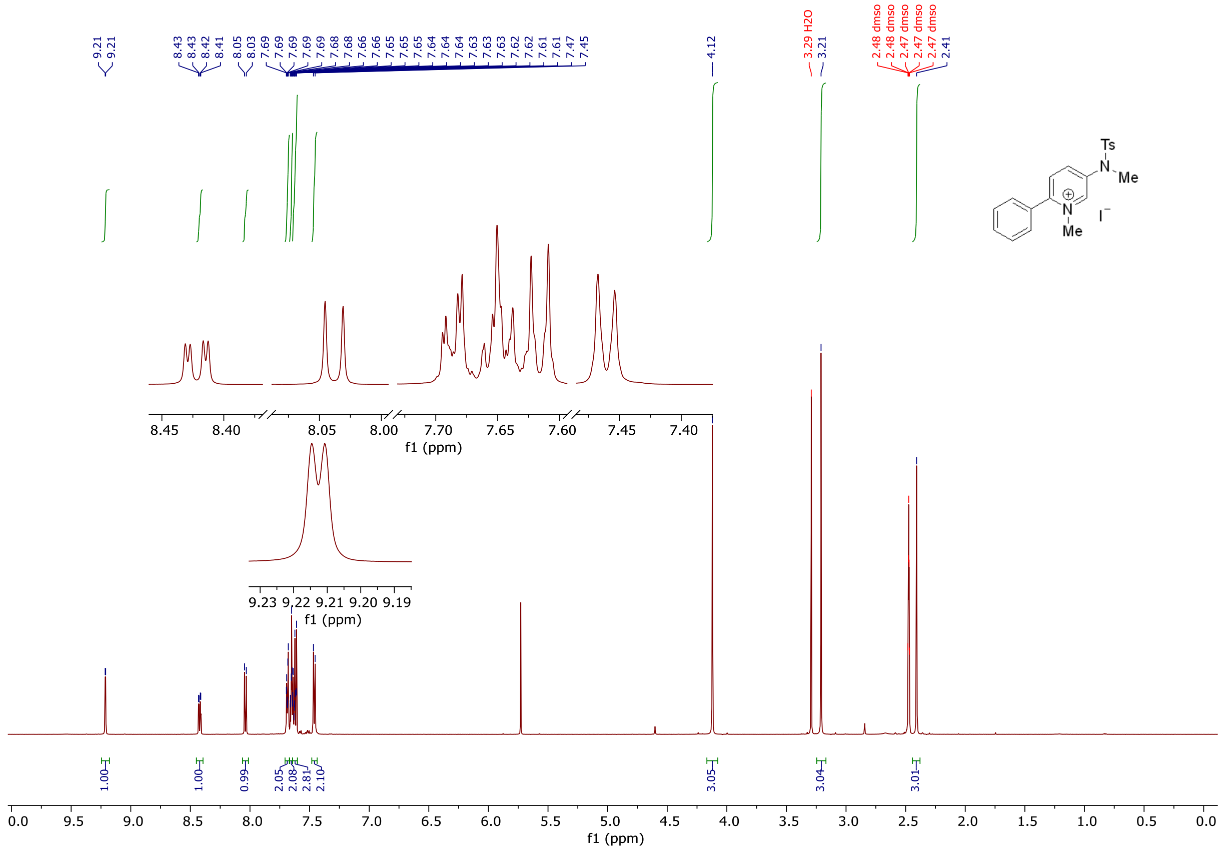

^{13}C NMR spectrum of compound 40 (DMSO-d_6, 298 K)

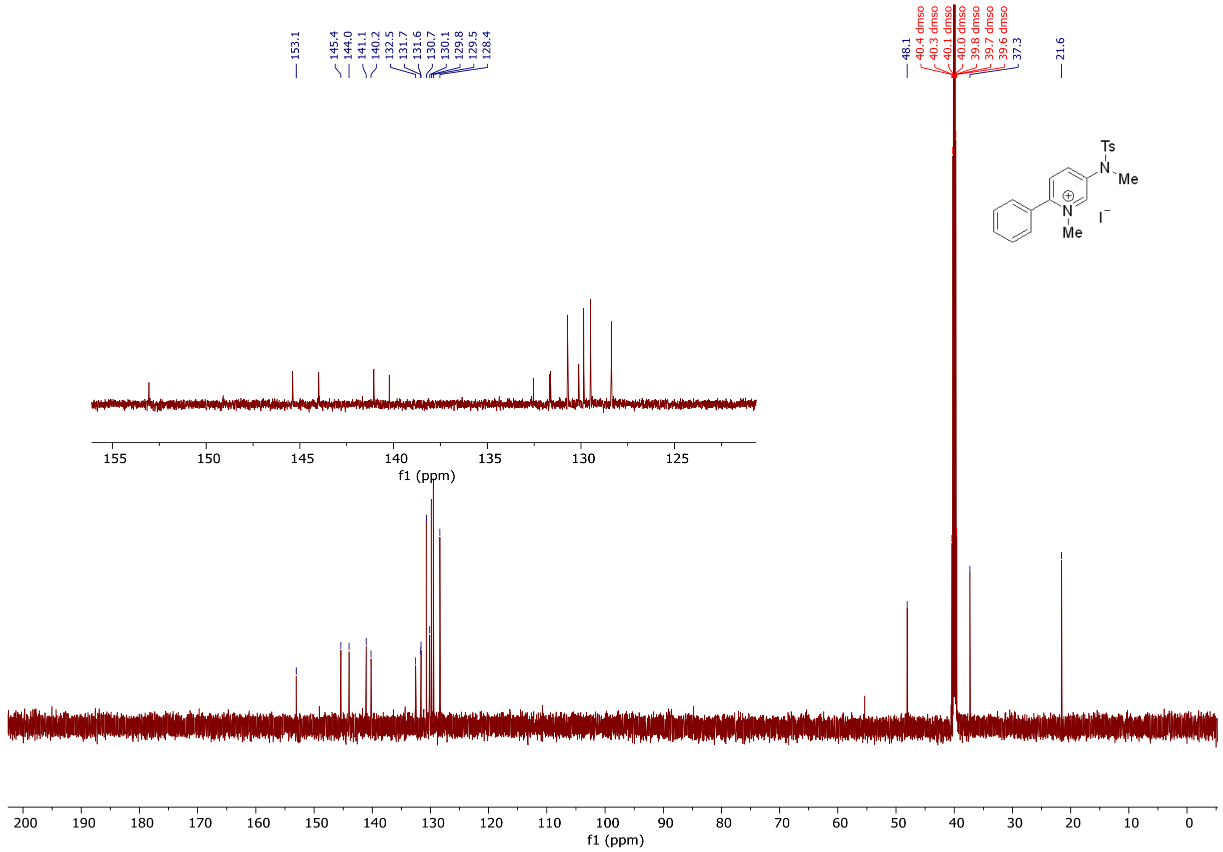

^{1}H NMR spectrum of compound 41a ($CDCl_3$, 298 K)

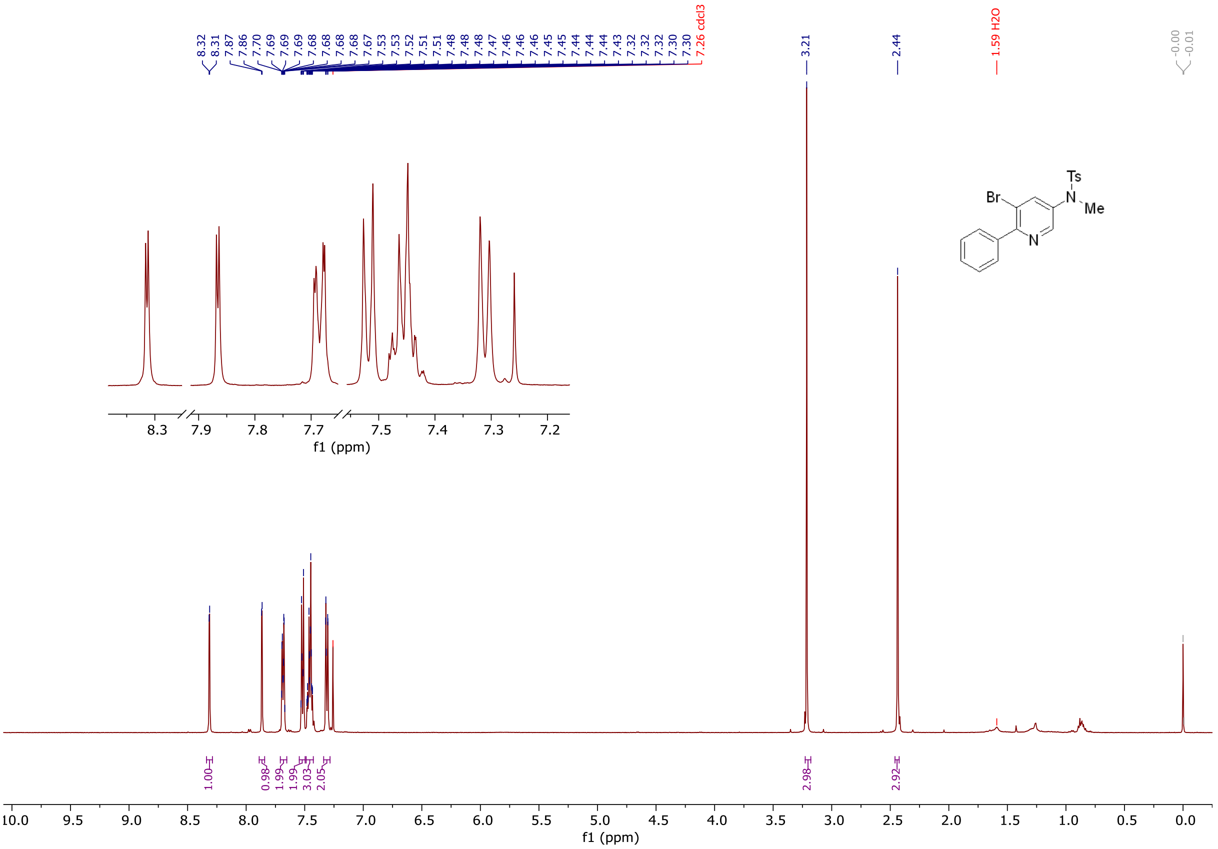

^{13}C NMR spectrum of compound 41a ($CDCl_3$, 298 K)

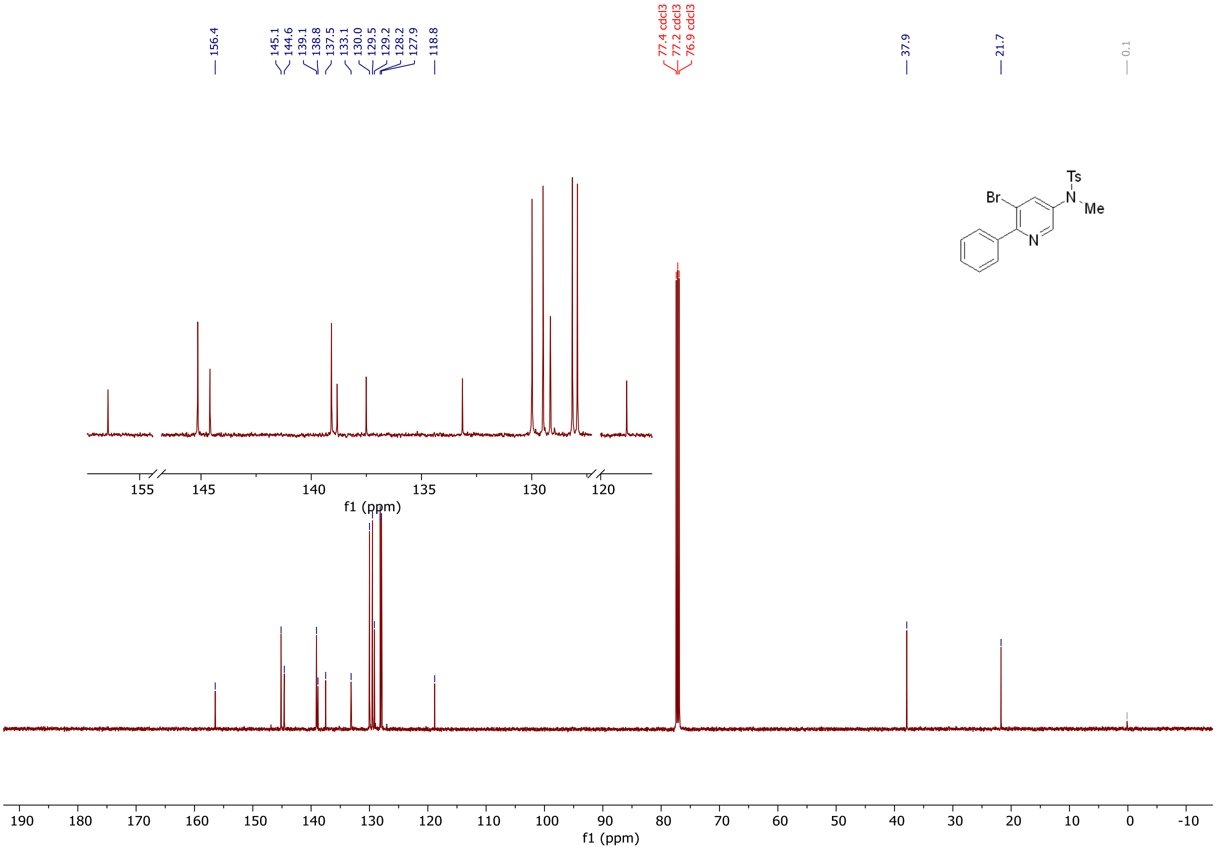

^{1}H NMR spectrum of compound 41b ($CDCl_3$, 298 K)

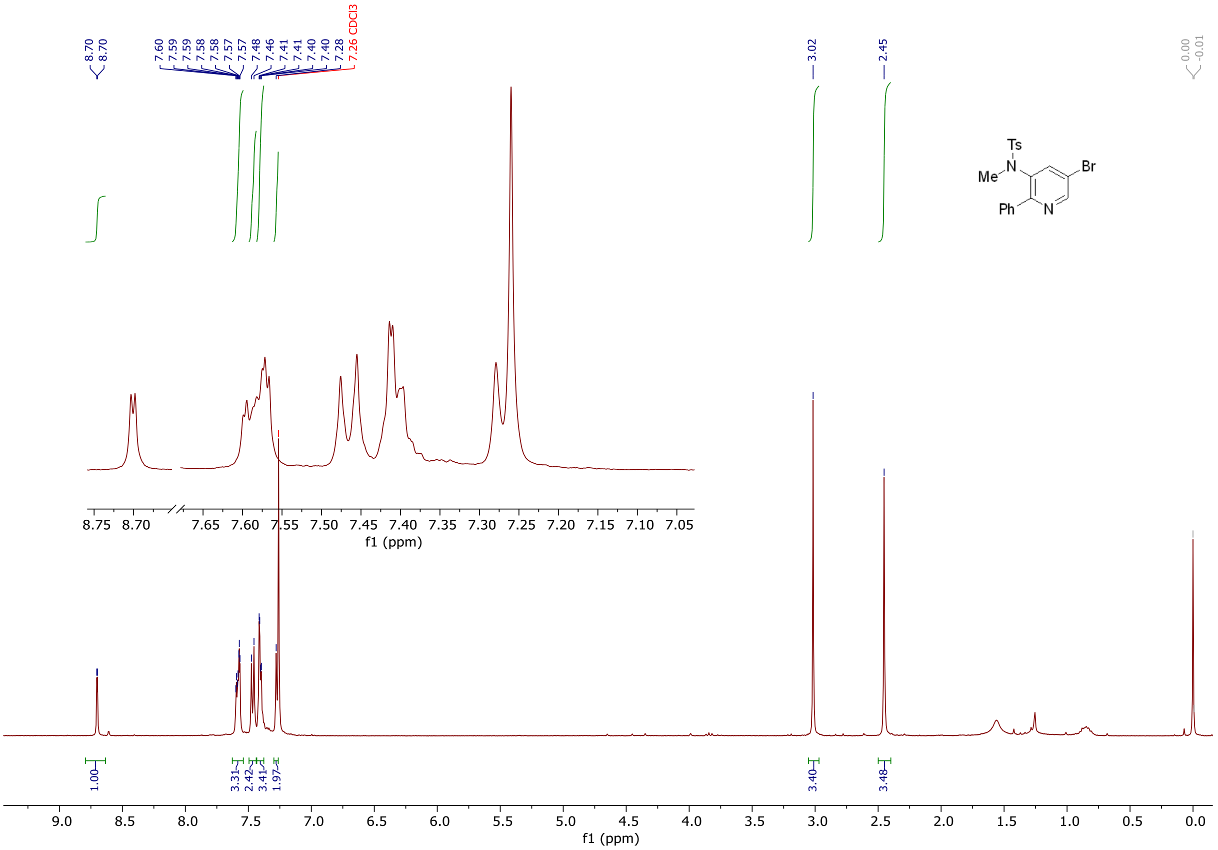

^{13}C NMR spectrum of compound 41b ($CDCl_3$, 298 K)

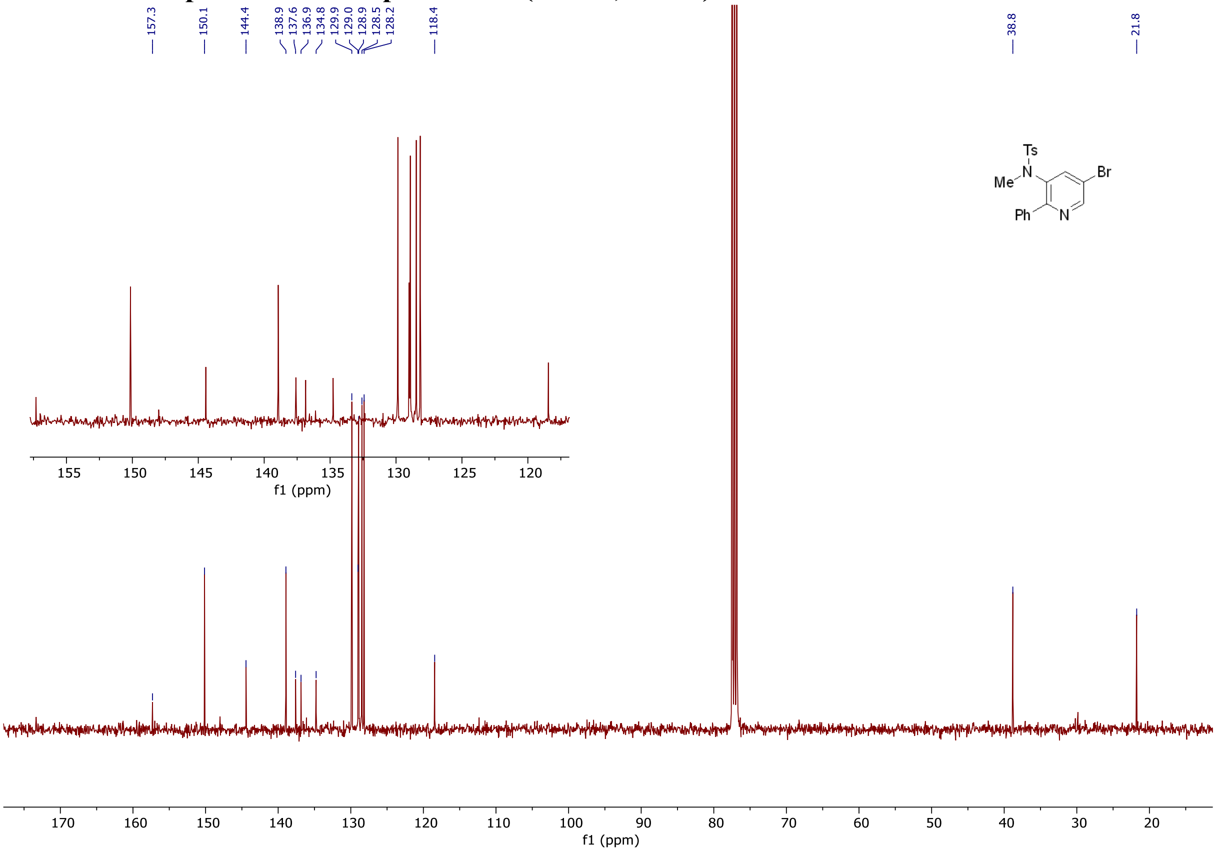

7. Crystallographic data for intermediate 2a

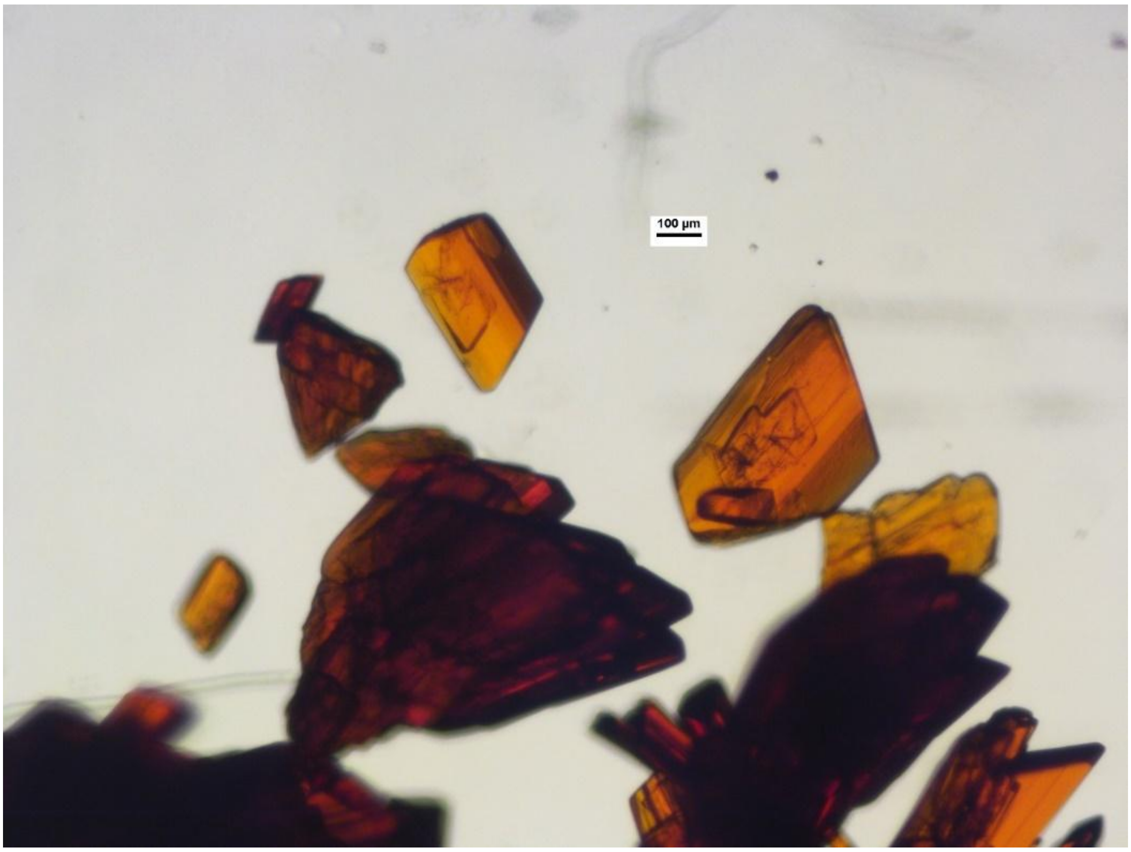

Supplementary Figure 18. Photography of a **2a** monocrystal.

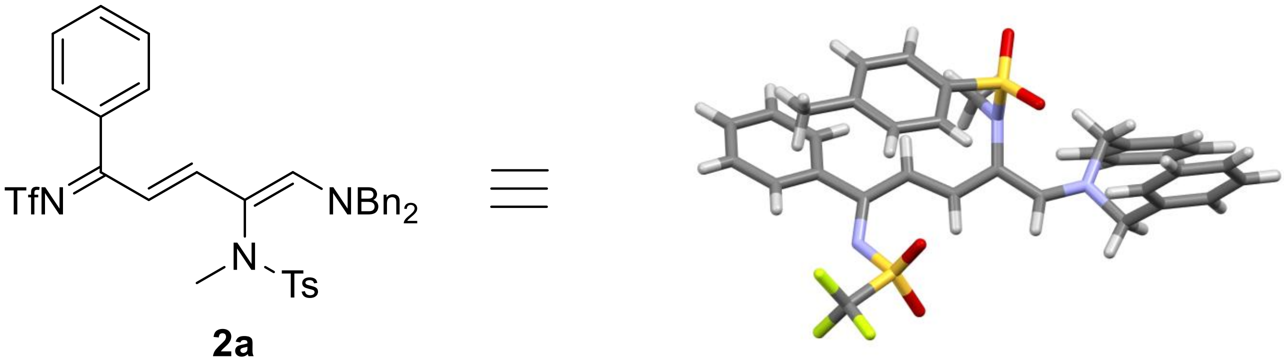

Supplementary Figure 19. Mercury drawing of **2a**.

A single crystal of $C_{34}H_{32}F_3N_3O_4S_2$ was prepared by slow evaporation of a DCM/ n-hexane solution. The suitable crystals of compounds 1-5 were mounted in paratone oil onto a nylon loop. All data were collected at 100.0(1) K, using a SuperNova Agilent, HyPix fitted with CuKα radiation (λ = 1.54184 Å). Data collection and unit cell refinement were performed using *CrysAlisPro* software.[5] The total number of data was measured in the 7.02° < 2θ < 140.112°, for compound 1, using ω scans. Data processing and absorption correction, giving minimum and maximum transmission factors (0.586, 0.754 for the compound, it was accomplished with

CrysAlisPro. All non-H atoms were refined with anisotropic displacement parameters. All hydrogen atom positions were determined by geometry and refined by a riding model.

Supplementary Table 12. Crystallographic data for the measured crystal.

Identification code	Compound ***2c-int.***
Empirical formula	$C_{34}H_{32}F_3N_3O_4S_2$
Formula weight	710.21
Crystal system	Triclinic
Space group	*P-1*
a (Å)	10.3347(3)
b (Å)	12.7875(5)
c (Å)	13.8621(5)
α (°)	113.322(4)
β (°)	95.246(3)
γ (°)	96.184(3)
Volume (Å^3)	1654.34(11)
Z	2
ρ (calc.)	1.426
λ	1.54184
Temp. (K)	100.0(1)
Crystal Size(mm)	0.220x 0.170x 0.110
Crystal Color	red
Crystal Morphology	prism
F(000)	738
μ (mm^{-1})	2.724
T_{min}, T_{max}	0.586, 0.754
$2\theta_{range}$ (°)	7.02 to 140.112
Reflections collected	15182
Independent reflections	6216<br>[R(int) = 0.0260]
Completeness	99.8%
Data / restraints / parameters	6216 / 0 / 444

Observed data [I > 2σ(I)]	5589
wR(F^2 all data)	0.0949
R(F obsd data)	0.0356
Goodness-of-fit on F^2	1.008
largest diff. peak and hole (e Å^{-3})	0.299 / -0.536

$$wR_2 = \{ \Sigma [w(F_o^2 - F_c^2)^2] / \Sigma [w(F_o^2)^2] \}^{1/2}$$

$$R_1 = \Sigma ||F_o| - |F_c|| / \Sigma |F_o|$$

8. Supplementary References

[1] B. T. Boyle, J. N. Levy, L. de Lescure, R. S. Paton, A. McNally, *Science* **2022**, *378*, 773–779.

[2] K. Goliszewska, K. Rybicka-Jasińska, J. Szurmak, D. Gryko, *J. Org. Chem.* **2019**, *84*, 15834–15844.

[3] K. F. Szabó, K. Goliszewska, J. Szurmak, K. Rybicka-Jasińska, D. Gryko, *Org. Lett.* **2022**, *24*, 8120–8124.

[4] J. Park, S. Chang, *Angew. Chemie Int. Ed.* **2015**, *54*, 14103–14107.

[5] S. Goggins, E. Rosevere, C. Bellini, J. C. Allen, B. J. Marsh, M. F. Mahon, C. G. Frost, *Org. Biomol. Chem.* **2014**, *12*, 47–52.

[6] S. Mo, Y. Zhu, Z. Shen, *Org. Biomol. Chem.* **2013**, *11*, 2756–2760.

[7] K. T. Hylland, I. L. Schmidtke, D. S. Wragg, A. Nova, M. Tilset, *Dalt. Trans.* **2022**, *51*, 5082–5097.

[8] L. Pignataro, M. Benaglia, R. Annunziata, M. Cinquini, F. Cozzi, *J. Org. Chem.* **2006**, *71*, 1458–1463.

[9] P. Sharma, S. Rohilla, N. Jain, *J. Org. Chem.* **2015**, *80*, 4116–4122.

[10] B. Qu, H. P. R. Mangunuru, S. Tcyrulnikov, D. Rivalti, O. V Zatolochnaya, D. Kurouski, S. Radomkit, S. Biswas, S. Karyakarte, K. R. Fandrick, J. D. Sieber, S. Rodriguez, J.-N. Desrosiers, N. Haddad, K. McKellop, S. Pennino, H. Lee, N. K. Yee, J. J. Song, M. C. Kozlowski, C. H. Senanayake, *Org. Lett.* **2018**, *20*, 1333–1337.

[11] M. S. M. Holmsen, A. Nova, K. Hylland, D. S. Wragg, S. Øien-ØDegaard, R. H. Heyn, M. Tilset,

Chem. Commun. **2018**, *54*, 11104–11107.

[12] X. Rao, C. Liu, J. Qiu, Z. Jin, *Org. Biomol. Chem.* **2012**, *10*, 7875–7883.

[13] J. L. Bolliger, C. M. Frech, *Adv. Synth. Catal.* **2010**, *352*, 1075–1080.

[14] J. Martín, E. Gómez-Bengoa, A. Genoux, C. Nevado, *Angew. Chemie - Int. Ed.* **2022**, *61*, 1–7.

[15] P. Chatelain, C. Muller, A. Sau, D. Brykczyńska, M. Bahadori, C. N. Rowley, J. Moran, *Angew. Chemie - Int. Ed.* **2021**, *60*, 25307–25312.

[16] T. Nguyen, N. German, A. M. Decker, T. L. Langston, T. F. Gamage, C. E. Farquhar, J. X. Li, J. L. Wiley, B. F. Thomas, Y. Zhang, *J. Med. Chem.* **2017**, *60*, 7410–7424.

[17] Y. Zou, G. Yue, J. Xu, J. (Steve) Zhou, *European J. Org. Chem.* **2014**, *2014*, 5901–5905.

[18] X. Chen, W. J. Li, S. Li, J. Tang, X. Du, X. L. Zheng, M. L. Yuan, H. Y. Fu, R. X. Li, H. Chen, *J. Org. Chem.* **2020**, *85*, 622–632.

[19] Y. Nishigaya, K. Umei, D. Watanabe, Y. Kohno, S. Seto, *Tetrahedron* **2016**, *72*, 1566–1572.

[20] K. K. Y. Kung, V. K. Y. Lo, H. M. Ko, G. L. Li, P. Y. Chan, K. C. Leung, Z. Zhou, M. Z. Wang, C. M. Che, M. K. Wong, *Adv. Synth. Catal.* **2013**, *355*, 2055–2070.

[21] C. V. Reddy, J. V. Kingston, J. G. Verkade, *J. Org. Chem.* **2008**, *73*, 3047–3062.

[22] S. T. Hobson, J. D. Boecker, J. H. Gifford, T. L. Nohe, C. H. Wierks, *J. Heterocycl. Chem.* **2003**, *40*, 277–282.

[23] J. D. Selingo, J. W. Greenwood, M. K. Andrews, C. Patel, A. J. Neel, B. Pio, M. Shevlin, E. M. Phillips, M. L. Maddess, A. McNally, *J. Am. Chem. Soc.* **2024**, *146*, 936–945.

[24] H. Wang, M. F. Greaney, *Angew. Chemie - Int. Ed.* **2024**, *63*, DOI 10.1002/anie.202315418.

[25] H. M. H. Nguyen, D. C. Thomas, M. A. Hart, K. R. Steenback, J. N. Levy, A. McNally, *J. Am. Chem. Soc.* **2024**, *146*, 2944–2949.

[26] M. J. Frisch, G. W. Trucks, H. B. Schlegel, G. E. Scuseria, M. A. Robb, J. R. Cheeseman, G. Scalmani, V. Barone, G. A. Petersson, H. Nakatsuji, X. Li, M. Caricato, A. V. Marenich, J. Bloino, B. G. Janesko, R. Gomperts, B. Mennucci, H. P. Hratchian, J. V. Ortiz, A. F. Izmaylov, J. L. Sonnenberg, D. Williams-Young, F. Ding, F. Lipparini, F. Egidi, J. Goings, B. Peng, A. Petrone, T. Henderson, D. Ranasinghe, V. G. Zakrzewski, J. Gao, N. Rega, G. Zheng, W. Liang, M. Hada, M. Ehara, K. Toyota, R. Fukuda, J. Hasegawa, M. Ishida, T. Nakajima, Y. Honda, O. Kitao, H. Nakai, T. Vreven, K. Throssell, J. A. Montgomery, Jr., J. E. Peralta, F. Ogliaro, M. J. Bearpark, J. J. Heyd, E. N. Brothers, K. N. Kudin, V. N. Staroverov, T. A. Keith, R. Kobayashi, J. Normand, K. Raghavachari, A.

P. Rendell, J. C. Burant, S. S. Iyengar, J. Tomasi, M. Cossi, J. M. Millam, M. Klene, C. Adamo, R. Cammi, J. W. Ochterski, R. L. Martin, K. Morokuma, O. Farkas, J. B. Foresman, D. J. Fox, *Gaussian 16, Revision C.01*, Gaussian, Inc., Wallingford CT **2016**.

[27] J.-D. Chai, M. Head-Gordon, *Phys. Chem. Chem. Phys.* **2008**, *10*, 6615–6620.

[28] F. Weigend, *Phys. Chem. Chem. Phys.* **2006**, *8*, 1057–1065.

[29] F. Weigend, R. Ahlrichs, *Phys. Chem. Chem. Phys.* **2005**, *7*, 3297–3305.

[30] A. V Marenich, C. J. Cramer, D. G. Truhlar, *J. Phys. Chem. B* **2009**, *113*, 6378–6396.

[31] C. Riplinger, F. Neese, *J. Chem. Phys.* **2013**, *138*, 34106.

[32] C. Riplinger, B. Sandhoefer, A. Hansen, F. Neese, *J. Chem. Phys.* **2013**, *139*, 134101.

[33] F. Neese, *WIREs Comput. Mol. Sci.* **2012**, *2*, 73–78.

[34] F. Neese, *WIREs Comput. Mol. Sci.* **2018**, *8*, e1327.

[35] F. Neese, F. Wennmohs, U. Becker, C. Riplinger, *J. Chem. Phys.* **2020**, *152*, 224108.

[36] F. Neese, F. Wennmohs, A. Hansen, U. Becker, *Chem. Phys.* **2009**, *356*, 98–109.

[37] C. Legault, *CYLview User Manual*, **2012**.

[38] R.I., Rodríguez, V., Corti, L. Rizzo, *et al. Nat Catal.,* **2004**, *7*, 1223–1231.
