# Supplementary material for: Photochemical C3-amination of pyridines via Zincke imine intermediates
Source: Nat Commun. 2025 May 31;16:5072. doi: 10.1038/s41467-025-59809-9 (PMC12126555; doi:10.1038/s41467-025-59809-9)
Supplement: Supplementary file 2 — Description of Additional Supplementary Files [file 41467_2025_59809_MOESM2_ESM.docx]

File name: Supplementary Data 1

Description: Cartesian coordinates of DFT calculated geometries (PDF).
