# Supplementary material for: Photochemical C3-amination of pyridines via Zincke imine intermediates
Source: Nat Commun. 2025 May 31;16:5072. doi: 10.1038/s41467-025-59809-9 (PMC12126555; doi:10.1038/s41467-025-59809-9)
Supplement: Supplementary file 3 — Supplementary Data 1 [file 41467_2025_59809_MOESM3_ESM.pdf]

Photochemical C3-Amination of Pyridines via Zincke Imine Intermediates

XYZ Cartesian coordinates and energies for all the calculated structures

Kitti Franciska Szabó,+[a] Piotr Banachowicz,+[a] Antoni Powała,[a,b] Danijela Lunic,[c] Ignacio Funes Ardoiz,*[c] and Dorota Gryko*[a]

[a]*Institute of Organic Chemistry Polish Academy of Sciences; Kasprzaka 44/52, 01-224 Warsaw, Poland; correspondence: dorota.gryko@icho.edu.pl*

[b]*Department of Chemistry Warsaw University of Technology, Noakowskiego 3, 00-664 Warsaw, Poland*

[c]*Complejo Científico-Tecnológico Universidad de La Rioja, Madre de Dios, 53, 26004 Logroño, La Rioja, Spain; correspondence :ignacio.funesa@unirioja.es*

correspondence:

dorota.gryko@icho.edu.pl

ignacio.funesa@unirioja.es

XYZ Cartesian coordinates and energies for all the calculated structures

The final G energies are calculated by the sum of E(DLPNO-CCSD(T)) + E(SMD/wB97xD/Def2TZVPP) – E(gas/wB97xD/Def2TZVPP) + G_{Corr} (SMD/wB97xD/Def2SVP)

[Ir^{III}]*

E (DLPNO-CCSD(T)) =-1537.64664
E (SMD/wB97xD/Def2TZVPP) = -1540.72049
E (gas/wB97xD/Def2TZVPP) = -1540.673991
G_{Corr}= 0.421248

Ir	-0.33209600	0.23854900	-0.11266000
N	-0.21646900	2.29409100	0.10712100
C	-1.26505000	3.17329700	0.13632700
C	1.10363400	2.80189700	0.01454200
C	-1.10647900	4.52688300	0.09622400
H	-2.25902600	2.72412500	0.20461600
C	1.29245600	4.19486800	-0.03328900
C	0.22574200	5.07113500	0.01106300
H	-1.98354400	5.17525100	0.13102400
H	2.31123500	4.58176800	-0.11238800
H	0.38660900	6.15023000	-0.02585900
N	-2.51468300	0.14951600	-0.19227600
C	-3.34174600	0.11761000	0.85624600
C	-2.99817100	0.08623600	-1.45071800
C	-4.71886200	0.02800200	0.70575000
H	-2.87824300	0.16987200	1.84414500
C	-4.37463700	-0.00678900	-1.67311200
C	-5.23967000	-0.03468000	-0.58537800
H	-5.36318200	0.00702600	1.58516200
H	-4.76652100	-0.05946000	-2.68855400
H	-6.31719200	-0.10697700	-0.74615800
N	-0.17285000	0.13845600	2.13263700
C	-0.07410500	1.19681700	2.94482700
C	-0.15691500	-1.11107300	2.64218000
C	0.04218300	1.06841000	4.32106000
H	-0.09240700	2.17582400	2.46389100
C	-0.04355600	-1.31180600	4.02208400
C	0.05708000	-0.21407600	4.86656000
H	0.11872500	1.95888900	4.94574700
H	-0.03388600	-2.32164400	4.43131400
H	0.14717300	-0.35915800	5.94509600
C	-0.60630900	0.18194000	-2.07927400
C	-1.96526900	0.11822200	-2.50725000
C	0.39675300	0.20854900	-3.06869900
C	-2.27964100	0.08606300	-3.86871700
C	0.07385300	0.17039000	-4.42211700
H	1.44571200	0.25296000	-2.76938700
C	-1.26372400	0.11029100	-4.82274900
H	-3.31904200	0.04274400	-4.20003100
H	0.86768700	0.18857500	-5.17283100
H	-1.51802000	0.08231000	-5.88461200
C	1.68970400	0.43639700	-0.14229600
C	2.64732600	-0.57750400	-0.22574900
C	2.13491200	1.79325100	-0.05663700
C	4.01924800	-0.29213600	-0.23641100
H	2.32432400	-1.62062200	-0.29592100
C	3.52177800	2.06912200	-0.06280000
C	4.44930900	1.03695900	-0.15394700
H	4.74793600	-1.10407000	-0.30827500
H	3.87682900	3.10080400	0.00744500
H	5.51783000	1.26927200	-0.15810300
C	-0.36206100	-1.77730200	0.29006100
C	-0.48338700	-2.77899100	-0.68908600
C	-0.27017800	-2.18558400	1.63828400
C	-0.49963600	-4.12966100	-0.34154200
H	-0.55694200	-2.50621600	-1.74421900
C	-0.28841100	-3.54500600	1.98321100
C	-0.40159000	-4.51783300	0.99635800
H	-0.58993200	-4.88680800	-1.12509400
H	-0.21361700	-3.85249000	3.02833700
H	-0.41445300	-5.57537800	1.26892900

[Ir^{III}]

E (DLPNO-CCSD(T)) =-1537.772353
E (SMD/wB97xD/Def2TZVPP) =-1540.820225
E (gas/wB97xD/Def2TZVPP) = -1540.774413
G_{Corr}= 0.427103

Ir	-0.33929200	0.21533300	0.01050800
N	-0.17999100	2.37880600	-0.07791200
C	-1.21041900	3.23306000	-0.09266800
C	1.08990300	2.84025900	-0.13982600
C	-1.03748900	4.60699700	-0.16727700
H	-2.20792100	2.79060600	-0.04184700
C	1.33203600	4.21754700	-0.22127600
C	0.26402500	5.10440000	-0.23417200
H	-1.90559300	5.26701200	-0.17520000
H	2.35435300	4.59123200	-0.27653400
H	0.44560600	6.17943100	-0.29727400
N	-2.50782800	0.14872400	-0.09826000
C	-3.33860500	0.12588900	0.95087400
C	-2.99847000	0.11642600	-1.35843700
C	-4.71708000	0.07310200	0.80805800
H	-2.87303300	0.15191700	1.93873300
C	-4.38186300	0.05895500	-1.57037000
C	-5.24450800	0.03803800	-0.48281200
H	-5.35743200	0.05770900	1.69065600
H	-4.77947200	0.02937900	-2.58461700
H	-6.32413000	-0.00636500	-0.64111400
N	-0.22283700	0.06592400	2.17183800
C	-0.17589300	1.10088400	3.01949500
C	-0.18584900	-1.20234000	2.64067700
C	-0.09197700	0.93457300	4.39372000
H	-0.20814100	2.09654300	2.57116600
C	-0.09719700	-1.43790300	4.01865700
C	-0.05082600	-0.36513100	4.89862100
H	-0.05789500	1.80619300	5.04823700
H	-0.06392400	-2.45898600	4.39829200
H	0.01779700	-0.54166000	5.97415400
C	-0.62028600	0.18926000	-1.99226900
C	-1.97571600	0.14911200	-2.42248200
C	0.35817000	0.21889100	-3.00549000
C	-2.31283700	0.13450700	-3.78477300
C	0.02227300	0.20321900	-4.35859600
H	1.41561900	0.24938700	-2.72814100
C	-1.31759300	0.16090500	-4.75595000
H	-3.35922800	0.10328600	-4.09807400
H	0.81289900	0.22444500	-5.11434600
H	-1.58325500	0.14908200	-5.81555400
C	1.66882700	0.44909900	-0.05130400
C	2.65964200	-0.55221900	-0.02611300

C	2.13014100	1.79351700	-0.11216000
C	4.01979200	-0.24820100	-0.06532700
H	2.35855100	-1.60258400	0.01980300
C	3.49943300	2.09866400	-0.14961000
C	4.44762900	1.08145100	-0.12711600
H	4.75707500	-1.05613800	-0.04687700
H	3.83591000	3.13728200	-0.19490600
H	5.51283600	1.32217000	-0.15658000
C	-0.31995400	-1.79257400	0.25482200
C	-0.38143500	-2.78782500	-0.74054300
C	-0.24935000	-2.24784600	1.60074700
C	-0.36680000	-4.14686600	-0.42942900
H	-0.43654900	-2.49139900	-1.79178500
C	-0.23562400	-3.61597100	1.91300500
C	-0.29354600	-4.56882700	0.90153700
H	-0.41328400	-4.88793300	-1.23276400
H	-0.18051800	-3.94798000	2.95257800
H	-0.28233600	-5.63313400	1.14770900

[Ir^{IV}]

E (DLPNO-CCSD(T)) =-1537.541746
E (SMD/wB97xD/Def2TZVPP) = -1540.634042
E (gas/wB97xD/Def2TZVPP) = -1540.544763
G_{Corr}= 0.427392

Ir	-0.28057400	0.15951800	-0.03163200
N	-0.17271500	2.35263400	-0.17366000
C	-1.22488500	3.17429400	-0.27251800
C	1.08732100	2.84080900	-0.19672900
C	-1.07600800	4.54617900	-0.39815500
H	-2.21350300	2.71173900	-0.24834300
C	1.30418700	4.21665300	-0.32088600
C	0.21564000	5.07234000	-0.42316300
H	-1.95673100	5.18420600	-0.47471100
H	2.31916500	4.61260400	-0.33664200
H	0.37373400	6.14807700	-0.52235700
N	-2.47851200	0.09862700	-0.09011700
C	-3.28188700	0.03267200	0.97867700
C	-2.98782800	0.09926200	-1.34309600
C	-4.66008500	-0.03329900	0.85545500
H	-2.79615800	0.03351600	1.95658200
C	-4.37186800	0.03271600	-1.53207400
C	-5.20969700	-0.03438600	-0.42709600
H	-5.28511500	-0.08502400	1.74718000
H	-4.78789200	0.03364400	-2.53910600
H	-6.29137300	-0.08831700	-0.56537700
N	-0.13510900	0.02589500	2.14892200
C	-0.02160600	1.07047900	2.97867800
C	-0.15037000	-1.23810900	2.62691200
C	0.07817300	0.90883600	4.35109700
H	-0.01236300	2.06121100	2.51906800
C	-0.05391900	-1.46732000	4.00309300
C	0.06103800	-0.38722500	4.86773900
H	0.16787500	1.78222300	4.99742100
H	-0.07000200	-2.48508900	4.39187200
H	0.13772400	-0.55516000	5.94382800
C	-0.62792300	0.23696900	-2.02543700
C	-1.98565300	0.17792200	-2.42201400
C	0.35414600	0.34998400	-3.01969000
C	-2.33091500	0.21303800	-3.77961600
C	0.00312700	0.37453000	-4.36911200
H	1.40900100	0.40593000	-2.74045800
C	-1.33884500	0.30561400	-4.75074000
H	-3.37743700	0.16868000	-4.08784000
H	0.78352100	0.44993100	-5.13062600
H	-1.61268700	0.32803500	-5.80769500
C	1.71495100	0.47607700	0.03768300
C	2.68477600	-0.52514200	0.19981100
C	2.14388600	1.82046400	-0.07994600
C	4.04175900	-0.20594100	0.20696000
H	2.37983700	-1.56879800	0.30428300
C	3.50881500	2.13321000	-0.06013800
C	4.45504300	1.12210400	0.07677200
H	4.78435800	-1.00002700	0.31771500
H	3.84258600	3.16880900	-0.15025800
H	5.51831600	1.37099200	0.08748500
C	-0.37554200	-1.83675000	0.25422300
C	-0.53174900	-2.79671600	-0.75820500
C	-0.27500700	-2.28286800	1.59611700
C	-0.55321800	-4.15666300	-0.45353600
H	-0.62066600	-2.48099900	-1.80007700
C	-0.30923300	-3.65018500	1.89497900
C	-0.44204900	-4.58441100	0.87184800
H	-0.65901100	-4.89073600	-1.25616800
H	-0.23165700	-3.99570100	2.92777200
H	-0.46264600	-5.65022100	1.10914600

[1]Py-salt

E (DLPNO-CCSD(T)) = -1278.258639
E (SMD/wB97xD/Def2TZVPP) =-1280.389402
E (gas/wB97xD/Def2TZVPP) = -1280.29965
G_{Corr}= 0.298925

N	-1.01113100	0.82738100	-0.33849200
C	-0.85442000	-0.61463400	-0.50184300
H	-0.06966300	-0.95019400	0.18783800
H	-1.78972000	-1.11511100	-0.21509600
H	-0.58509200	-0.89597900	-1.53007400
S	-2.31743300	1.64101400	-1.05642200
O	-2.57124800	0.97445000	-2.32204900
O	-1.96422400	3.05041600	-0.99742600
N	0.06012700	1.58228100	0.09693900
C	0.96092700	2.06760400	-0.81432800
C	0.17925000	1.79244400	1.44578100
C	2.01452900	2.83134800	-0.34866300
C	1.23632600	2.55663900	1.90010200
C	2.17344700	3.10012700	1.01455800
H	2.72656700	3.22356900	-1.07549200
H	1.32557400	2.72698100	2.97366000
C	-3.69110800	1.33836600	0.01223900
C	-4.44090300	0.17329400	-0.14694600
C	-4.00974500	2.28169300	0.99120000
C	-5.51240900	-0.05652300	0.71102300
H	-4.20083600	-0.54121700	-0.93605900
C	-5.08734200	2.03490300	1.83317500
H	-3.42835400	3.20040000	1.08657500
C	-5.84974300	0.86265000	1.71351000
H	-6.10462000	-0.96682300	0.59176100
H	-5.34713400	2.77145100	2.59729500
C	-6.99204500	0.59883200	2.65271500
H	-7.68034300	-0.15601800	2.24836100
H	-6.61187200	0.22488700	3.61745600
H	-7.55699000	1.51922500	2.85989000
C	0.77587000	1.76666400	-2.26278500
H	0.63428000	0.69157900	-2.43654600
H	-0.09712500	2.30028500	-2.66405900
H	1.66253100	2.09882400	-2.81453300
C	-0.81585200	1.17125900	2.36443900
H	-1.83114700	1.53522400	2.15912500
H	-0.82401600	0.07775400	2.25011100
H	-0.55619500	1.41797600	3.39988500
C	3.32782900	3.90473600	1.51397000
H	4.15738000	3.22277200	1.76440700
H	3.68910300	4.60483200	0.74935700

H	3.06368800	4.45495100	2.42698800

col

E (DLPNO-CCSD(T)) = -365.5708833
E (SMD/wB97xD/Def2TZVPP) =-366.2657277
E (gas/wB97xD/Def2TZVPP) = -366.2538219
G_{Corr}= 0.135736

C	0.40281800	0.22975900	0.05410500
C	1.80056200	0.24216300	0.02882100
C	2.48372500	1.46070200	-0.01873100
C	1.71178100	2.62582900	-0.03484800
C	0.31693300	2.53560800	-0.00805200
N	-0.31667200	1.35782400	0.03522500
H	2.35381200	-0.70016800	0.04884300
H	2.19342000	3.60638200	-0.06634800
C	-0.54890200	3.76528700	-0.02549700
H	-1.20107800	3.78501400	0.86149900
H	0.04870700	4.68661500	-0.04293200
H	-1.20547400	3.75615200	-0.90966100
C	-0.36949300	-1.05973600	0.10836600
H	-0.99377900	-1.09186000	1.01504800
H	-1.04909800	-1.13321900	-0.75491000
H	0.29400400	-1.93499200	0.10866200
C	3.98389000	1.51544800	-0.07911600
H	4.43814200	0.65035900	0.42464400
H	4.32155600	1.50131300	-1.12845900
H	4.37092300	2.43684000	0.37887500

col-H^+

E (DLPNO-CCSD(T)) = -365.9563344
E (SMD/wB97xD/Def2TZVPP) =-366.7372187
E (gas/wB97xD/Def2TZVPP) = -366.6472544
G_{Corr}= 0.150951

C	0.43994700	0.18521200	-0.01226100
C	1.82327100	0.22953600	0.03392300
C	2.49715100	1.45815500	0.01590000
C	1.73710800	2.63457800	-0.04227500
C	0.35521700	2.57728800	-0.08748000
H	2.37825500	-0.70803700	0.08509400
H	2.22400200	3.61055800	-0.05085900
C	-0.53924800	3.76805400	-0.15041100
H	-1.22801200	3.77282200	0.70800900
H	0.05100400	4.69116100	-0.14048700
H	-1.14563900	3.74007500	-1.06855300
C	-0.36844300	-1.06759400	0.00722600
H	-1.00897500	-1.09043300	0.90220600
H	-1.02242900	-1.11393900	-0.87663300
H	0.28664400	-1.94602600	0.01545000
C	3.99141900	1.51784900	0.02912400
H	4.42637800	0.61868000	0.48486500
H	4.35865900	1.58164200	-1.00845900
H	4.34811100	2.41038300	0.56096700
N	-0.22762500	1.35811300	-0.07260100
H	-1.24843600	1.32112000	-0.10764500

^{1}1b

E (DLPNO-CCSD(T)) = -1959.220897
E (SMD/wB97xD/Def2TZVPP) = -1962.384818
E (gas/wB97xD/Def2TZVPP) = -1962.334707
G_{Corr}= 0.377928

C	1.63022400	1.20952500	-1.18897600
H	2.20727700	0.73962100	-1.98896400
C	2.12405800	2.29737700	-0.48620900
H	1.51972800	2.69023100	0.33851400
C	0.37340000	0.68124600	-0.84733100
H	-0.16117600	1.17679900	-0.02859300
N	3.26788000	2.94697600	-0.68901400
C	4.17623400	2.60693900	-1.77495600
H	3.58635200	2.18569500	-2.60244900
H	4.60821900	3.54579700	-2.14956500
C	3.69511700	4.00036400	0.23576900
H	4.08621700	4.83767600	-0.35976700
H	2.80699700	4.35457900	0.77604700
C	5.29909400	1.65710100	-1.40010900
C	6.54591900	1.79860900	-2.01774700
C	5.12728700	0.64363700	-0.45140000
C	7.60382200	0.95041400	-1.69024900
H	6.69565900	2.59440300	-2.75288700
C	6.18576400	-0.19971600	-0.11581500
H	4.16510900	0.52165400	0.05118900
C	7.42844900	-0.04892000	-0.73300500
H	8.57349200	1.08002500	-2.17708900
H	6.03840800	-0.97755700	0.63729800
H	8.25875800	-0.70678200	-0.46558500
C	4.74586700	3.51515600	1.21200100
C	6.10003600	3.78972700	1.00384700
C	4.37366900	2.71130800	2.29538700
C	7.07093800	3.25219300	1.84964400
H	6.39867000	4.41534100	0.15822500
C	5.34003200	2.17606700	3.14491300
H	3.31571200	2.49168400	2.46603200
C	6.69271200	2.44029300	2.91902600
H	8.12735200	3.46487700	1.66917800
H	5.03804300	1.54722700	3.98590000
H	7.45176800	2.01532400	3.58017500
C	-0.22015500	-0.41351800	-1.44834800
H	0.31688500	-0.95407200	-2.23319900
C	-1.48340600	-0.94068500	-1.08116800
C	-2.34616900	-0.22238500	-0.09523900
C	-2.78879700	1.08059500	-0.35427300
C	-2.72722400	-0.85893600	1.09309100
C	-3.61053100	1.73484700	0.56146200
H	-2.50029600	1.57592600	-1.28399800
C	-3.53551000	-0.19461700	2.01290600
H	-2.38573700	-1.87508900	1.30051300
C	-3.98201700	1.10038100	1.74730900
H	-3.96212100	2.74650800	0.34667300
H	-3.81898100	-0.69319900	2.94254100
H	-4.62170300	1.61675600	2.46685200
N	-1.84479000	-2.09187200	-1.62601800
S	-3.31371600	-2.74743200	-1.47181100
O	-4.43520800	-1.83381800	-1.65517800
C	-3.27069600	-3.75135200	-3.03120300
O	-3.37712500	-3.73791700	-0.40336000
F	-2.27808100	-4.62640000	-3.01133200
F	-4.41780400	-4.41101000	-3.13164700
F	-3.13563300	-2.96889600	-4.09088700

^{2}A

E (DLPNO-CCSD(T)) = -1278.425985
E (SMD/wB97xD/Def2TZVPP) = -1280.492721
E (gas/wB97xD/Def2TZVPP) = -1280.467586
G_{Corr}= 0.29446

N	-0.89747700	0.47601500	-0.93070900
C	-0.89428700	-0.97966300	-1.02341100
H	0.09823400	-1.31954400	-0.70374300

H	-1.65404800	-1.44979500	-0.37742200
H	-1.05569300	-1.29304000	-2.06244500
S	-2.21957300	1.34428200	-1.50783400
O	-2.77591700	0.53630200	-2.58474200
O	-1.75363100	2.70075200	-1.75777500
N	0.00736500	1.12489600	-0.12517700
C	0.99653500	1.91027000	-0.75839000
C	-0.24859900	1.27759200	1.26233200
C	1.57403000	2.93183900	-0.06134300
C	0.33679500	2.33070100	1.92064700
C	1.22872600	3.23001800	1.29110800
H	2.34562600	3.52003600	-0.56595600
H	0.12931100	2.43361800	2.99005800
C	-3.40576200	1.41549900	-0.19196800
C	-4.30770600	0.36533100	-0.02778200
C	-3.35978400	2.47846900	0.71154600
C	-5.16731000	0.37976000	1.06815400
H	-4.33908700	-0.45399400	-0.74829400
C	-4.22442400	2.47468200	1.79938300
H	-2.64860700	3.29320100	0.56601800
C	-5.13669700	1.42653200	1.99876100
H	-5.87614400	-0.44091300	1.20286400
H	-4.19168900	3.30266700	2.51220100
C	-6.07127100	1.44795200	3.17567600
H	-6.56749700	0.47764800	3.31448400
H	-5.53444300	1.70359200	4.10142500
H	-6.85290000	2.21147200	3.03235500
C	1.35056500	1.54168700	-2.16300900
H	1.61024300	0.47327300	-2.24162500
H	0.51073600	1.72554300	-2.85024100
H	2.20963800	2.13668000	-2.49993400
C	-1.03527000	0.20550500	1.94723000
H	-2.02181900	0.03418500	1.49144600
H	-0.49645800	-0.75730400	1.92171300
H	-1.19352800	0.47960900	2.99863000
C	1.81154000	4.41571500	2.00199100
H	2.89193200	4.51660500	1.80478300
H	1.34323600	5.36374300	1.67956600
H	1.67183000	4.33889200	3.09097900

TS-1

E (DLPNO-CCSD(T)) = -1278.201573
E (SMD/wB97xD/Def2TZVPP) =-1280.479996
E (gas/wB97xD/Def2TZVPP) =-1280.454336
G_{Corr}= 0.292835

N	-0.94308400	0.40065000	-1.14941400
C	-1.18171700	-1.02430800	-1.14270500
H	-0.23625100	-1.51177800	-0.86684700
H	-1.95708400	-1.34909600	-0.42603800
H	-1.46253200	-1.36683600	-2.14973000
S	-2.14652600	1.44468600	-1.53658900
O	-2.85742200	0.88465100	-2.68602600
O	-1.54195900	2.76840100	-1.64421200
N	0.26458900	0.89302200	-0.05003700
C	1.13272700	1.76161200	-0.66308700
C	-0.16007900	1.13539500	1.23363500
C	1.51549000	2.92694300	-0.02387200
C	0.19157400	2.31649400	1.86864700
C	1.02566700	3.25862900	1.25012400
H	2.22676000	3.58696800	-0.52703300
H	-0.16125400	2.48190300	2.89003300
C	-3.29913700	1.49553700	-0.18036100
C	-4.26001900	0.49221200	-0.05499800
C	-3.18016000	2.49625400	0.78461800
C	-5.09114900	0.48308800	1.06315100
H	-4.36109100	-0.27417700	-0.82570300
C	-4.01915600	2.47413400	1.89331200
H	-2.43068500	3.28009400	0.66634200
C	-4.98188300	1.46629100	2.05524600
H	-5.84152500	-0.30492300	1.16573800
H	-3.92554300	3.25603000	2.65161300
C	-5.89186800	1.46685900	3.25220900
H	-6.38892300	0.49541900	3.38138600
H	-5.33742200	1.70283300	4.17256700
H	-6.67582000	2.23343100	3.13917200
C	1.61823300	1.39201100	-2.02909100
H	1.86855100	0.32182200	-2.08309100
H	0.83111700	1.58522300	-2.77363200
H	2.50477700	1.98245700	-2.29669600
C	-0.95473500	0.06310400	1.91196200
H	-1.94643500	-0.06566100	1.45173900
H	-0.43514300	-0.90541400	1.84448400
H	-1.10537300	0.31057300	2.97084400
C	1.37991500	4.55724500	1.91373200
H	2.40292500	4.87115700	1.65805300
H	0.70111900	5.36212800	1.58417100
H	1.29725400	4.48524800	3.00793700

^{2}B

E (DLPNO-CCSD(T)) =-912.8687315
E (SMD/wB97xD/Def2TZVPP) = -914.24234
E (gas/wB97xD/Def2TZVPP) =-914.2250597
G_{Corr}= 0.128493

N	-1.32040000	0.44809000	0.06530400
S	-0.55708600	1.13953200	-1.26474000
O	0.21798200	0.08028500	-1.90456200
O	0.11300100	2.36329200	-0.83731200
C	-1.93216100	1.55755700	-2.29299300
C	-2.44126400	0.60121600	-3.17313800
C	-2.49178900	2.82967000	-2.19873200
C	-3.53326000	0.93378200	-3.96527100
H	-1.98498100	-0.38819000	-3.23920700
C	-3.58560500	3.14379400	-3.00167700
H	-2.07619000	3.56726400	-1.50984800
C	-4.12249800	2.20615800	-3.89304500
H	-3.93809900	0.19188900	-4.65826100
H	-4.02923800	4.14006600	-2.93433800
C	-5.29472700	2.54729700	-4.76938800
H	-4.99011900	2.57326600	-5.82778600
H	-6.08629500	1.78799500	-4.67902800
H	-5.71942700	3.52708300	-4.51192300
C	-0.42365800	0.14197700	1.14026100
H	-0.90596000	-0.57398600	1.81888900
H	0.55127700	-0.24973100	0.80106200
H	-0.22070900	1.06943000	1.71173500

C5-selectivity

TS-2

E (DLPNO-CCSD(T)) =-2872.10894
E (SMD/wB97xD/Def2TZVPP) =-2876.634886
E (gas/wB97xD/Def2TZVPP) =-2876.5724300
G_{Corr}= 0.535675

C	1.99162000	2.15551100	-1.55800800
H	1.35406700	1.91228600	-2.41078500

C	0.36181900	1.02258300	-0.19738700
H	-0.22006000	0.92095200	-1.11775200
N	3.15444600	2.69982200	-1.84736900
C	4.07879700	3.16776700	-0.81953500
H	4.55890600	4.07748500	-1.20627600
H	3.48010200	3.47829800	0.04931500
C	3.56249200	2.86732900	-3.24694100
H	2.67171400	2.72756600	-3.87246700
H	3.91175000	3.90175900	-3.37196400
C	5.14787700	2.16833300	-0.42028000
C	4.93820600	0.78681400	-0.46905000
C	6.38730400	2.64759700	0.01784000
C	5.95143900	-0.09773100	-0.09920700
H	3.98441000	0.38710900	-0.82138300
C	7.39823100	1.76526000	0.39647600
H	6.56759100	3.72572200	0.04974600
C	7.18513300	0.38768500	0.33446700
H	5.77408800	-1.17438900	-0.15556900
H	8.36195900	2.15682900	0.73088100
H	7.97987300	-0.30554600	0.61975900
C	4.64683500	1.88615900	-3.63149300
C	5.98997100	2.27086100	-3.64342100
C	4.31613500	0.55262400	-3.89620300
C	6.99206300	1.33282500	-3.89404000
H	6.25448300	3.31118000	-3.43532100
C	5.31417100	-0.38576700	-4.15065000
H	3.26654500	0.24479900	-3.88731300
C	6.65586100	0.00212300	-4.14279800
H	8.03978500	1.64239500	-3.89003600
H	5.04587700	-1.42551400	-4.35233600
H	7.43987200	-0.73439100	-4.33423500
C	-0.05747500	0.38184600	0.93628900
H	0.47339900	0.54026000	1.87861200
C	-1.21398200	-0.46708800	1.01220900
C	-1.86589400	-0.95974400	-0.23209000
C	-1.09923700	-1.56116100	-1.24041500
C	-3.24994400	-0.82897000	-0.40595000
C	-1.71165200	-2.02681700	-2.40156300
H	-0.02247000	-1.68169400	-1.10594000
C	-3.85573300	-1.28249900	-1.57403300
H	-3.85521500	-0.35634300	0.36875800
C	-3.08886800	-1.88296300	-2.57303500
H	-1.10886400	-2.50563500	-3.17626800
H	-4.93361000	-1.16420300	-1.70449400
H	-3.56656300	-2.24254900	-3.48729800
N	-1.63822300	-0.75214000	2.22280900
S	-2.80038500	-1.83022000	2.58151400
O	-2.73144400	-3.09024300	1.85549000
C	-2.19514700	-2.23379500	4.28888600
O	-4.09072700	-1.18593400	2.78703600
F	-2.19387300	-1.15907200	5.05825900
F	-3.01290500	-3.13642400	4.81163000
F	-0.97283400	-2.73756200	4.24168200
C	1.50786000	1.88639400	-0.24516600
H	2.24337600	1.75316400	0.55206400
N	1.09837200	3.66267300	0.53900100
S	0.07933700	4.59478000	-0.31440300
C	0.84175300	3.51531400	1.95441400
O	0.54118100	4.57449800	-1.70434700
O	-0.06179200	5.89355800	0.35111900
C	-1.54092400	3.83854900	-0.28671400
H	1.69599800	2.99053700	2.40913100
H	0.75941900	4.50098100	2.44064200
H	-0.07382200	2.93837000	2.18783500
C	-1.98461600	3.10573400	-1.38561000
C	-2.34091800	3.96303300	0.85151500
C	-3.23332500	2.48944400	-1.33993400
H	-1.35625900	3.02519500	-2.27429800
C	-3.58000500	3.33089000	0.88798600
H	-2.00371200	4.56015000	1.70076900
C	-4.04907100	2.58910900	-0.20561100
H	-3.58027900	1.91771200	-2.20442800
H	-4.20278600	3.42373100	1.78165200
C	-5.40668800	1.94432800	-0.15955300
H	-5.54398800	1.23226900	-0.98488600
H	-5.56215200	1.41379300	0.79208900
H	-6.19826100	2.70733200	-0.23760100

^{2}C

E (DLPNO-CCSD(T)) =-2872.109611

E (SMD/wB97xD/Def2TZVPP) =-2876.653958

E (gas/wB97xD/Def2TZVPP) =-2876.579182

G_{Corr}= 0.537752

C	2.03965900	0.22040600	0.20753600
H	1.99716500	-0.46516900	1.05546900
C	0.41124500	-0.19441300	-0.79334000
H	-0.03388200	0.75960400	-1.08205900
N	2.36425000	1.48827200	0.47442200
C	2.59442900	2.47172700	-0.57405500
H	1.73422000	2.47599000	-1.26340700
H	2.59541700	3.46048200	-0.09430200
C	2.47536000	1.92584400	1.87002300
H	1.94391300	2.88243800	1.97641100
H	1.96430100	1.18468400	2.49709000
C	3.87707100	2.30520600	-1.36796200
C	4.03638000	3.06634900	-2.53311300
C	4.90525400	1.44433100	-0.97905800
C	5.20342700	2.97422600	-3.28766700
H	3.23409800	3.73789200	-2.85173100
C	6.07059500	1.34153200	-1.74202000
H	4.80162300	0.84130800	-0.07536300
C	6.22558000	2.10704800	-2.89597000
H	5.31174200	3.57582300	-4.19338300
H	6.86142900	0.65704700	-1.42598900
H	7.13635000	2.02556600	-3.49377000
C	3.92329700	2.06520400	2.28195300
C	4.58593700	3.29134700	2.17374100
C	4.64349200	0.93523500	2.68866500
C	5.95058900	3.38516500	2.45069400
H	4.03001100	4.17962600	1.86159700
C	6.00580800	1.02673200	2.96749600
H	4.13124700	-0.02688200	2.77873400
C	6.66351200	2.25248800	2.84304100
H	6.45900000	4.34773300	2.35760700
H	6.55803000	0.13824000	3.28281100
H	7.73240900	2.32471700	3.05765000
C	-0.35500100	-1.20176900	-0.20880500
H	0.13632100	-2.14954000	0.02923500
C	-1.71223900	-1.10737700	0.13552000
C	-2.50091400	0.11094200	-0.21194900
C	-2.61369100	0.53738900	-1.54195100
C	-3.14385600	0.84044300	0.79748300
C	-3.35858900	1.67241700	-1.85635600
H	-2.12582700	-0.03475200	-2.33452500
C	-3.87938200	1.98083600	0.48115400
H	-3.06645100	0.51296900	1.83637100
C	-3.99042700	2.39841600	-0.84565600
H	-3.44891600	1.98942800	-2.89794600
H	-4.36953800	2.54647300	1.27698800
H	-4.57191300	3.28976000	-1.09283100
N	-2.24733200	-2.13089600	0.82567100
S	-3.81697300	-2.33428100	1.05137300
O	-4.67284300	-2.05726400	-0.10023500
C	-3.80501100	-4.18450600	1.17857400
O	-4.27434800	-1.89697100	2.36866700
F	-3.04935000	-4.59578000	2.18680100

F	-5.04927700	-4.60190100	1.38631300
F	-3.35827100	-4.73435900	0.05807800
C	1.85923900	-0.34279800	-1.14214700
H	2.16764500	0.35239400	-1.93673800
N	2.38206800	-1.68759700	-1.31876900
S	2.09751100	-2.33972400	-2.84212900
C	3.70945700	-1.96625400	-0.77146500
O	0.72720100	-2.00675700	-3.19974500
O	2.52509400	-3.72875100	-2.78454800
C	3.18793500	-1.45238600	-3.92834100
H	3.70395700	-1.75984700	0.30593700
H	3.93153900	-3.03103300	-0.90566600
H	4.50106400	-1.36167800	-1.24638200
C	2.73856700	-0.28198900	-4.54466300
C	4.50077400	-1.89358400	-4.09465000
C	3.62404200	0.45371300	-5.32435700
H	1.70483300	0.04499200	-4.41905400
C	5.37263500	-1.14272400	-4.87847100
H	4.83797700	-2.81642000	-3.61953300
C	4.95224000	0.03941500	-5.50139800
H	3.27766100	1.37223800	-5.80464700
H	6.40306000	-1.48285700	-5.00721400
C	5.89237500	0.84440000	-6.35316000
H	5.68643000	0.67058600	-7.42198500
H	5.76838400	1.92161400	-6.16886700
H	6.94023600	0.57277500	-6.16483600

[1]D

E (DLPNO-CCSD(T)) = -2871.939588

E (SMD/wB97xD/Def2TZVPP) =-2876.524789

E (gas/wB97xD/Def2TZVPP) =-2876.400013

G_{Corr}= 0.546006

C	2.72840300	1.92313000	0.05069400
H	2.09118900	1.91875200	0.94203100
C	0.75242700	0.97509400	-1.11992100
H	0.18526500	1.90671500	-1.02552900
N	3.84333000	2.54619400	0.14782300
C	4.78937100	2.73756800	-0.97619500
H	4.42529300	2.18535000	-1.84763300
H	4.75276200	3.81240600	-1.20287000
C	4.22774100	3.15189600	1.44404700
H	4.76493800	4.08159600	1.21947500
H	3.29918500	3.40983200	1.96960900
C	6.19576800	2.31231900	-0.63754200
C	7.19614400	3.26757500	-0.44576200
C	6.50637700	0.95392300	-0.50604900
C	8.48987300	2.87296400	-0.10389600
H	6.95763400	4.32875400	-0.55384300
C	7.79336500	0.55999100	-0.15054800
H	5.73691400	0.20084100	-0.68794600
C	8.78663000	1.52042400	0.05625900
H	9.26583100	3.62683900	0.04694800
H	8.02459500	-0.50177900	-0.03971100
H	9.79639000	1.21071800	0.33568000
C	5.08447800	2.22083300	2.27773700
C	6.31937400	2.65807700	2.76286300
C	4.65303000	0.92536200	2.58026700
C	7.12109600	1.80749900	3.52377200
H	6.66400100	3.66820600	2.52979000
C	5.45599700	0.07177600	3.33413800
H	3.68725800	0.56430900	2.21937600
C	6.69496000	0.50970700	3.80440700
H	8.08845600	2.15946500	3.88930400
H	5.11082600	-0.94074800	3.55539300
H	7.32689000	-0.16075100	4.39140000
C	0.12849400	-0.20268000	-1.23369200
H	0.68430300	-1.14011500	-1.28645900
C	-1.32800700	-0.36471700	-1.27612600
C	-2.19419800	0.80095700	-1.58165200
C	-1.94591300	1.56030200	-2.73461200
C	-3.25718400	1.14495300	-0.73499300
C	-2.77302700	2.63458000	-3.04996700
H	-1.11769000	1.29307200	-3.39365400
C	-4.06456700	2.23595900	-1.04537700
H	-3.44360400	0.57238400	0.17478900
C	-3.82989300	2.97533800	-2.20488200
H	-2.58783100	3.21039400	-3.95909100
H	-4.88301800	2.50899400	-0.37615400
H	-4.47171200	3.82467500	-2.44959800
N	-1.74427800	-1.57197500	-1.05149500
S	-3.31549200	-2.04988000	-1.18462100
O	-3.92929100	-1.70058500	-2.45524800
C	-2.96702300	-3.87404100	-1.26592400
O	-4.04149200	-1.86592000	0.06006500
F	-2.39323700	-4.27796500	-0.14861800
F	-4.12453700	-4.49617200	-1.41417600
F	-2.18440900	-4.16156500	-2.28992100
C	2.25026400	1.15592100	-1.16674900
H	2.44510500	1.78713500	-2.05132800
N	3.04459400	-0.05085500	-1.29705700
S	3.58235600	-0.50247600	-2.81474300
C	3.08123600	-1.04502400	-0.22140900
O	4.57609000	-1.53824800	-2.58586400
O	3.93309100	0.72950100	-3.51461500
C	2.17440300	-1.20425700	-3.62281300
H	2.44549000	-0.69412900	0.60195000
H	2.68720600	-2.01843300	-0.54960500
H	4.10190000	-1.18030700	0.16163200
C	1.83346800	-2.53457900	-3.37532200
C	1.34677200	-0.37305500	-4.37826700
C	0.62521200	-3.02102200	-3.86597400
H	2.49881100	-3.18105200	-2.80001900
C	0.14918700	-0.88087300	-4.86743000
H	1.63122100	0.66254400	-4.57171700
C	-0.24133400	-2.20184700	-4.60390000
H	0.34946800	-4.05926800	-3.66895300
H	-0.50279700	-0.23535900	-5.46087100
C	-1.57562000	-2.70454300	-5.07517700
H	-1.59446300	-3.80064900	-5.14529900
H	-2.35968300	-2.39920500	-4.36379700
H	-1.83661200	-2.28114200	-6.05574700

TS-3

E (DLPNO-CCSD(T)) =-3237.518256

E (SMD/wB97xD/Def2TZVPP) = -3242.791875

E (gas/wB97xD/Def2TZVPP) = -3242.669086

G_{Corr}= 0.704826

C	6.87578500	-0.76473300	-4.76761100
H	7.60117300	-1.33109500	-4.17384000
C	5.30849600	-2.03028600	-3.44098100
H	6.19008900	-2.44971600	-2.94961200
N	7.40216700	-0.16601500	-5.78947600
C	6.71288700	0.75295600	-6.71420700
H	5.66046200	0.80195700	-6.42671700
H	7.15349200	1.74506100	-6.54105300
C	8.85453300	-0.33310300	-6.02748200
H	9.23607300	0.63035700	-6.38851300
H	9.32379800	-0.53635200	-5.05610700
C	6.88047700	0.36346100	-8.16380600
C	7.48920700	1.24908900	-9.05562000
C	6.44747900	-0.88313700	-8.63072000
C	7.66802800	0.89576100	-10.39442000

H	7.83753800	2.22135100	-8.69721300
C	6.63346500	-1.24198900	-9.96255800
H	5.98721500	-1.59030100	-7.93869600
C	7.24585700	-0.35217800	-10.84870000
H	8.14790900	1.59677300	-11.08123500
H	6.30297600	-2.22310300	-10.31122700
H	7.39412400	-0.63475900	-11.89359300
C	9.17403500	-1.43577000	-7.01395700
C	9.91519700	-1.15528500	-8.16448500
C	8.73242200	-2.74421200	-6.78966800
C	10.19261800	-2.16095000	-9.09034600
H	10.26046000	-0.13469200	-8.34781500
C	9.00389000	-3.74916300	-7.71598200
H	8.16003600	-2.98757500	-5.89025200
C	9.73073000	-3.45862300	-8.87202100
H	10.76312200	-1.92523300	-9.99171400
H	8.64615500	-4.76533100	-7.53439100
H	9.93815100	-4.24535800	-9.60109200
C	4.11793000	-2.63713400	-3.27647500
H	3.20610000	-2.24063400	-3.72087900
C	3.91445500	-3.79334700	-2.42161600
C	5.07564900	-4.47995400	-1.79709800
C	5.16065000	-4.55736000	-0.40105200
C	6.09108600	-5.02655700	-2.59164000
C	6.25788900	-5.17501200	0.19337100
H	4.37718900	-4.11845800	0.22038000
C	7.17565800	-5.66056800	-1.99032500
H	6.02164300	-4.96795700	-3.68002600
C	7.26260400	-5.73083300	-0.59925800
H	6.32746600	-5.22225100	1.28210500
H	7.95828100	-6.10035700	-2.61217200
H	8.11880200	-6.22090600	-0.13029500
N	2.67222200	-4.12295900	-2.21710200
S	2.19624800	-5.45511700	-1.38606700
O	1.83572300	-5.11252800	-0.01837600
C	0.58998900	-5.69735700	-2.29002900
O	2.95961600	-6.66141700	-1.65686200
F	0.81782300	-5.88067200	-3.57853200
F	0.00608700	-6.77529700	-1.79233300
C	5.54094300	-0.76092700	-4.17575000
H	5.89115600	0.01937500	-3.14227600
N	4.43652500	-0.29632600	-4.97953600
S	3.43467400	0.94189000	-4.47927200
C	3.93170900	-1.14672800	-6.06494200
O	2.66159400	1.31092600	-5.65567100
O	4.26072800	1.93267200	-3.80437600
C	2.34325700	0.23900300	-3.27543600
H	2.93135900	-1.55377900	-5.85159900
H	3.89330700	-0.59188400	-7.01147200
H	4.62026000	-1.99296900	-6.17955400
C	1.21722000	-0.47435500	-3.69023500
C	2.69201000	0.31746700	-1.92841300
C	0.46289500	-1.15279700	-2.73759800
H	0.93726400	-0.50593600	-4.74473400
C	1.92400900	-0.36382100	-0.99080500
H	3.56102400	0.90153600	-1.62159500
C	0.81195300	-1.12371700	-1.37948100
H	-0.41256100	-1.72318100	-3.05645200
H	2.19918700	-0.31055700	0.06547100
C	0.04404300	-1.92059800	-0.36460600
H	0.54051600	-2.89113800	-0.20443800
H	0.00697300	-1.40350200	0.60498000
H	-0.98303900	-2.12126500	-0.69924300
C	7.04055800	1.94622100	-2.21245300
C	7.42381000	2.77913200	-1.16426800
C	7.20248600	2.39863800	0.16071200
C	6.60408500	1.15574300	0.37121900
C	6.23755800	0.35310700	-0.70802300
N	6.43647300	0.76444100	-1.97588700
H	7.90015500	3.73501300	-1.39229900
H	6.41913400	0.79687700	1.38568300
C	5.64884000	-1.00409600	-0.46234800
H	6.36311500	-1.78752200	-0.75871500
H	5.42414100	-1.13835900	0.60328100
H	4.72746800	-1.15736000	-1.03545600
C	7.32948100	2.33382600	-3.63093100
H	8.16275700	1.73085900	-4.02386200
H	6.44400900	2.17776200	-4.25851600
H	7.62272000	3.38938800	-3.69145700
C	7.62419500	3.27311400	1.30318000
H	7.45827900	4.33498600	1.07220800
H	7.08622600	3.01748000	2.22615600
H	8.70196800	3.14353200	1.49419900
F	-0.21078700	-4.65906600	-2.13122900

[1]E

E (DLPNO-CCSD(T)) = -2871.575463

E (SMD/wB97xD/Def2TZVPP) =-2876.094609

E (gas/wB97xD/Def2TZVPP) = -2876.038797

G_{Corr}= 0.533212

C	3.20023800	1.98672600	-0.89493200
H	2.44694400	2.71248500	-0.57783400
C	1.29653200	0.54455000	-0.99666000
H	0.75735700	1.42956200	-0.64530300
N	4.43260800	2.47622800	-0.88212700
C	5.63623600	1.71926600	-1.20862000
H	5.51998500	1.28674800	-2.20956900
H	6.45540300	2.44868500	-1.28209800
C	4.63500300	3.86824500	-0.44705700
H	5.33170500	4.34426200	-1.15125800
H	3.67333400	4.39016000	-0.52238400
C	6.03119300	0.64032900	-0.22382900
C	6.98706300	-0.29904000	-0.63024100
C	5.50036400	0.54655700	1.06409600
C	7.40428800	-1.30926000	0.23324400
H	7.39447200	-0.24653100	-1.64375000
C	5.91632200	-0.46632000	1.93079800
H	4.74814300	1.26222400	1.40057000
C	6.86897900	-1.39698300	1.52047000
H	8.14699900	-2.03701800	-0.10263700
H	5.48744700	-0.52583400	2.93407500
H	7.19176700	-2.19112600	2.19783100
C	5.16828600	3.94599300	0.96510200
C	6.54277000	4.01368100	1.21158300
C	4.28560500	3.86626400	2.04934600
C	7.03035800	3.98265700	2.51883900
H	7.23928600	4.08299100	0.37149600
C	4.76970300	3.83797400	3.35583700
H	3.20881100	3.81512000	1.86478900
C	6.14511600	3.88983400	3.59254200
H	8.10696800	4.03007200	2.69873800
H	4.07171300	3.77419700	4.19402300
H	6.52590700	3.86319500	4.61629900
C	0.58268300	-0.62376000	-1.14705700
H	1.08473600	-1.55158300	-1.41824800
C	-0.82060500	-0.73205400	-0.96458300
C	-1.65416900	0.48889900	-0.75862700
C	-1.68649900	1.48749800	-1.74051500
C	-2.41086300	0.64134200	0.41020000
C	-2.47915000	2.61892200	-1.56055700
H	-1.09918000	1.36514700	-2.65285700
C	-3.18759300	1.78348400	0.59278000
H	-2.38566900	-0.13074100	1.18120000
C	-3.22792900	2.77008400	-0.39293000
H	-2.51012400	3.38701400	-2.33653600
H	-3.76558300	1.90232700	1.51193600

H	-3.84407500	3.66082100	-0.24980100
N	-1.33449100	-1.95033300	-0.99727800
S	-2.91911400	-2.26786600	-0.96459900
O	-3.71619000	-1.55862700	-1.96088200
C	-2.80522400	-4.01480600	-1.58174700
O	-3.45213500	-2.40499400	0.38537300
F	-2.10643100	-4.76241800	-0.74388500
F	-4.03626900	-4.50029800	-1.66618600
F	-2.24331600	-4.07013200	-2.77932100
N	3.49269600	-0.33945700	-1.71697400
S	3.69309400	-0.44295600	-3.37284800
C	3.68072700	-1.56683200	-0.94010600
O	4.61395900	-1.54889400	-3.60058300
O	4.02293600	0.89440300	-3.85690500
C	2.10398200	-0.86328300	-4.03465100
H	3.38046900	-1.35319500	0.09260000
H	3.07156300	-2.40667500	-1.31142800
H	4.73667200	-1.86600800	-0.94148900
C	1.68781300	-2.19528300	-4.02869400
C	1.24301600	0.15878100	-4.43226600
C	0.37681600	-2.49405200	-4.38563200
H	2.37810900	-2.98892800	-3.73673700
C	-0.06087500	-0.16015900	-4.79598700
H	1.58826300	1.19356900	-4.44462500
C	-0.52242100	-1.48325800	-4.75652400
H	0.04258800	-3.53377000	-4.37077100
H	-0.73977200	0.63735700	-5.10823600
C	-1.95813000	-1.79874500	-5.06476200
H	-2.07399300	-2.82089600	-5.45133100
H	-2.56055200	-1.71901500	-4.14531000
H	-2.37428900	-1.09288100	-5.79777400
C	2.68690800	0.72700500	-1.21793200

^{1}F

E (DLPNO-CCSD(T)) =-2871.559285
E (SMD/wB97xD/Def2TZVPP) =-2876.060136
E (gas/wB97xD/Def2TZVPP) = -2876.020308
G_{Corr}= 0.532004

C	1.09225400	0.67891900	-0.82663800
C	0.06172900	1.56851300	-0.48467400
H	-0.91850800	1.09823300	-0.38673500
C	0.77943700	-0.65833200	-1.15069500
H	1.47966500	-1.10644800	-1.86817400
N	0.02469200	2.88404500	-0.36120900
C	1.18537100	3.77774200	-0.45856200
H	2.07749600	3.15725100	-0.56913200
H	1.07062200	4.37743200	-1.37417300
C	-1.28798300	3.54822400	-0.26516500
H	-1.23065100	4.46574400	-0.86711000
H	-2.02658200	2.89068600	-0.74277000
C	1.32559300	4.68259700	0.74237300
C	1.26771500	6.07053300	0.60508300
C	1.50521300	4.12775500	2.01477500
C	1.36934200	6.89748100	1.72586500
H	1.12681400	6.50968200	-0.38647200
C	1.58839300	4.94916600	3.13559200
H	1.58013700	3.04336600	2.11704300
C	1.51695600	6.33793700	2.99406100
H	1.31885900	7.98254300	1.60727200
H	1.71257800	4.50438500	4.12598700
H	1.58114400	6.98260800	3.87401100
C	-1.73365200	3.87939200	1.14324500
C	-1.89387100	5.20583400	1.55438000
C	-2.00289900	2.85205500	2.05096100
C	-2.28481700	5.50092400	2.86039200
H	-1.69566400	6.01654000	0.84902700
C	-2.38873800	3.14172600	3.35822800
H	-1.89614000	1.80963700	1.74341200
C	-2.52439000	4.46867800	3.76871700
H	-2.39496300	6.54267200	3.17130200
H	-2.57896400	2.31881800	4.05076500
H	-2.82187100	4.69873800	4.79472500
C	-0.23691700	-1.52459800	-0.75801100
C	-0.94682100	-1.57014500	0.46265000
C	-1.83650300	-2.75737800	0.65485800
C	-1.37000000	-4.05591400	0.41219000
C	-3.16453100	-2.56845900	1.06134900
C	-2.21562400	-5.14885800	0.58868300
H	-0.33297600	-4.21325000	0.10689800
C	-4.01209900	-3.66270200	1.21809900
H	-3.53986900	-1.56003000	1.24738500
C	-3.53873200	-4.95470200	0.98790500
H	-1.83775400	-6.15882800	0.41433800
H	-5.04903100	-3.50396900	1.52267200
H	-4.20257100	-5.81237100	1.11920600
N	-0.80986300	-0.63643800	1.40196600
S	-1.22537000	-0.87660800	2.94280600
O	-1.00578800	-2.22015900	3.46703700
C	0.11018500	0.12844000	3.76794900
O	-2.46490300	-0.18807800	3.29349500
F	0.31414300	1.29205600	3.18027000
F	-0.26768100	0.34303400	5.02153500
F	1.24968600	-0.54962400	3.78387200
H	-0.36279100	-2.40695500	-1.38820200
N	2.42376600	1.11861800	-1.11275100
C	2.84183900	1.23656800	-2.50544100
S	3.56679600	0.72726000	0.04806700
H	3.82637800	1.71546000	-2.55341900
H	2.88741400	0.26192900	-3.02187000
H	2.11544800	1.87931200	-3.02220900
O	3.04482600	1.20729000	1.32078800
C	3.59910100	-1.04585300	0.11386000
O	4.84835000	1.21314100	-0.44531000
C	2.80816100	-1.70280100	1.05353500
C	4.32257200	-1.75813600	-0.84534100
C	2.73259500	-3.09230300	1.02559100
H	2.23933200	-1.13106100	1.78546100
C	4.23321000	-3.14563000	-0.86015000
H	4.94476500	-1.23434600	-1.57393100
C	3.43477700	-3.83439400	0.06785000
H	2.10232900	-3.60442000	1.75710200
H	4.79292700	-3.70984100	-1.61051000
C	3.35814300	-5.33518000	0.04149100
H	4.26473800	-5.77369600	0.48954000
H	2.49204800	-5.70331000	0.60889700
H	3.29011800	-5.71169700	-0.98972800

^{1}TS^{-F}

E (DLPNO-CCSD(T)) = -2871.53695
E (SMD/wB97xD/Def2TZVPP) =-2876.031239
E (gas/wB97xD/Def2TZVPP) = -2875.990992
G_{Corr}= 0.532643

C	1.42970600	-0.10755300	-0.24460000
C	0.19324200	0.73276100	-0.12258800
H	-0.58602100	0.32859600	-0.77613200
C	1.27436700	-1.44896100	-0.22379800
H	2.11073300	-2.08547100	-0.52036300
N	0.15577700	2.07050900	-0.08758900
C	1.24930800	2.97510600	0.31810400
H	2.19582000	2.54023300	-0.01263300
H	1.09935100	3.89673600	-0.25942300

C	-0.99748000	2.71439200	-0.74312500
H	-0.64255000	3.14119900	-1.69567700
H	-1.72182800	1.92593200	-0.98399100
C	1.38340600	3.32244900	1.78584300
C	0.77431000	4.45760200	2.32971900
C	2.18298700	2.52777200	2.61199200
C	0.93416200	4.77350100	3.67836100
H	0.16418300	5.10226400	1.69336300
C	2.34146100	2.83658900	3.96177800
H	2.68392900	1.64955100	2.20061700
C	1.71296600	3.95900900	4.50144200
H	0.44852900	5.66309600	4.08773800
H	2.96075400	2.19598900	4.59481600
H	1.83726800	4.20474100	5.55924200
C	-1.68754100	3.79523800	0.05242000
C	-1.55520200	5.13757600	-0.31677500
C	-2.47935900	3.47215600	1.15949800
C	-2.18130400	6.14462800	0.41975200
H	-0.94621000	5.39812300	-1.18700600
C	-3.09931000	4.47432800	1.90191400
H	-2.58790200	2.42867300	1.45590000
C	-2.94803000	5.81424400	1.53665600
H	-2.06514800	7.18947600	0.12223700
H	-3.70523300	4.20932000	2.77174500
H	-3.43468200	6.59959100	2.12013900
C	0.04338000	-2.09912300	0.15405600
C	-0.91886600	-1.50874000	0.91365900
C	-2.14483700	-2.26173900	1.29109100
C	-2.06707100	-3.57373200	1.77167000
C	-3.40453500	-1.67663600	1.09894600
C	-3.22976000	-4.29154300	2.05309700
H	-1.08855100	-4.02788400	1.94413400
C	-4.56340500	-2.39709200	1.36999700
H	-3.46893600	-0.65054100	0.73012400
C	-4.47927800	-3.70662500	1.85040500
H	-3.15706000	-5.31179300	2.43708700
H	-5.54010100	-1.93501700	1.20773000
H	-5.39003100	-4.26902900	2.06982200
N	-0.77329700	-0.16088500	1.27929800
S	-0.98426400	0.26276200	2.82426400
O	-2.18109000	-0.28826500	3.44450500
C	0.38893500	-0.59893500	3.75326900
O	-0.72126600	1.67978100	2.96381300
F	0.48300500	-0.08169200	4.97142900
F	0.11423000	-1.89483200	3.86048800
F	1.54852900	-0.46584900	3.13807400
H	-0.10777500	-3.13067400	-0.16801700
N	2.63553600	0.50903100	-0.67300100
C	2.67833200	0.97839200	-2.05769000
S	4.10115900	0.20177300	0.06982000
H	3.53572100	1.64793600	-2.19828100
H	2.73737200	0.13601700	-2.76775800
H	1.76474700	1.55201900	-2.26732700
O	3.81663300	-0.20889800	1.43690500
C	4.84147900	-1.17195600	-0.77918700
O	4.93048900	1.37258500	-0.18457600
C	4.75565400	-2.44932200	-0.22151300
C	5.49127700	-0.96014900	-1.99468200
C	5.31391800	-3.52335200	-0.90472200
H	4.26074300	-2.59722400	0.73992200
C	6.03976300	-2.04981800	-2.66699100
H	5.57854100	0.04434800	-2.41217500
C	5.95875100	-3.34420000	-2.13831900
H	5.25118400	-4.52434600	-0.47031500
H	6.54689300	-1.88745500	-3.62115700
C	6.55700900	-4.52019800	-2.85824600
H	7.37546600	-4.96068500	-2.26720900
H	5.80548300	-5.31040400	-3.00859900
H	6.95894900	-4.23165100	-3.83905200

$^{1}F^{+}$

E (DLPNO-CCSD(T)) =-2871.929067
E (SMD/wB97xD/Def2TZVPP) =-2876.460808
E (gas/wB97xD/Def2TZVPP) = -2876.387973
G_{Corr}= 0.547401

C	1.13341000	0.66838300	1.20693400
C	0.57145800	1.86445900	0.98801200
H	0.78515400	2.69543200	1.65639500
C	1.06244600	-0.52607400	0.34062600
H	2.05159400	-0.84589200	-0.00896400
N	-0.31250700	2.16635600	-0.12552100
C	0.28561400	2.07094400	-1.50816700
H	0.69077700	1.05326100	-1.58739700
H	-0.57137100	2.13357900	-2.18994000
C	-1.16366700	3.39754800	0.12596600
H	-1.90148200	3.39744600	-0.68552500
H	-1.68871700	3.18524200	1.06502900
C	1.32839600	3.09129800	-1.89137600
C	1.20342800	3.71594100	-3.13700800
C	2.43084500	3.40599300	-1.08806400
C	2.14295600	4.65299700	-3.56401600
H	0.34769400	3.47865800	-3.77453500
C	3.35873500	4.35899200	-1.50375800
H	2.57270700	2.91992600	-0.12269700
C	3.21773500	4.98754800	-2.74171700
H	2.02445300	5.13385800	-4.53768400
H	4.20247200	4.60666700	-0.85564500
H	3.94727600	5.73336300	-3.06551400
C	-0.43429600	4.71324100	0.18944200
C	-0.37107900	5.53499100	-0.94196000
C	0.16123700	5.15430300	1.37798200
C	0.32473700	6.74074000	-0.90704700
H	-0.86510000	5.21859700	-1.86295200
C	0.86349200	6.35860000	1.41477900
H	0.06651800	4.56130000	2.29110500
C	0.95687400	7.14796600	0.26892600
H	0.37386500	7.36483700	-1.80221500
H	1.33070600	6.68525800	2.34652700
H	1.50889700	8.09035400	0.29614900
C	0.03114500	-1.32607600	0.02040400
C	-1.35547300	-1.22027300	0.48627800
C	-2.02032100	-2.51440600	0.79712100
C	-1.51942200	-3.28072400	1.85831000
C	-3.09103500	-2.98223100	0.02690000
C	-2.11887500	-4.49776600	2.16915800
H	-0.68864500	-2.89798500	2.45567200
C	-3.66752300	-4.21293100	0.33317100
H	-3.46584300	-2.39161600	-0.80931300
C	-3.19022600	-4.96500100	1.40633700
H	-1.74470200	-5.08501500	3.01027500
H	-4.49869300	-4.58324000	-0.27050300
H	-3.65435400	-5.92385400	1.64814000
N	-1.88300100	-0.04221900	0.60259000
S	-3.43965200	0.27523000	1.14437700
O	-4.44736900	-0.28806800	0.27053700
C	-3.62004800	-0.55524000	2.83561300
O	-3.44147700	1.69109000	1.45132700
F	-4.21264100	-1.72331200	2.72007300
F	-2.43858900	-0.69662800	3.40124500
F	-4.36865000	0.24322300	3.56889300
H	-1.01368900	1.38158000	-0.08557400
N	2.07927600	0.59079500	2.26404700
C	3.29912400	1.39559700	2.22593700
S	1.97166000	-0.65956500	3.37066300
H	3.83818600	1.27308900	3.17250900
H	3.95089200	1.09328700	1.39140100

H	3.04548400	2.45956900	2.12846600
O	0.59961400	-1.14254400	3.28374100
C	3.07145600	-1.93508300	2.80679500
O	2.49553400	-0.15123500	4.62754200
C	2.57185600	-3.01253700	2.07785700
C	4.43563200	-1.82936400	3.09199700
C	3.45493600	-3.98591100	1.61664600
H	1.50473700	-3.09710900	1.87073700
C	5.30161700	-2.80710700	2.61789400
H	4.81725700	-0.99713100	3.68600000
C	4.82888300	-3.89800300	1.87173000
H	3.06414300	-4.83344200	1.04850700
H	6.36932400	-2.72720700	2.83732700
C	5.78493700	-4.94495200	1.37373200
H	6.29637500	-5.43595400	2.21639600
H	5.26915900	-5.71685100	0.78663100
H	6.56481000	-4.49152500	0.74249700
H	0.25040900	-2.22982100	-0.55391300

$^{1}TS^{F+}$

E (DLPNO-CCSD(T)) =-2871.894013
E (SMD/wB97xD/Def2TZVPP) =-2876.426061
E (gas/wB97xD/Def2TZVPP) =-2876.351288
G_{Corr}= 0.54984

C	0.43469200	0.66861700	1.43925300
C	-0.57343000	1.53885000	1.06669600
H	-0.84601300	2.29769500	1.79928200
C	0.54935900	-0.65685300	0.96350200
H	1.50841200	-1.16797100	1.09560300
N	-0.92730300	1.99955400	-0.28586700
C	-0.39778400	1.26266700	-1.50705300
H	-0.38828100	0.19884500	-1.25595600
H	-1.18200600	1.41785100	-2.25702100
C	-0.82011100	3.53077500	-0.36549400
H	-0.86382400	3.77149900	-1.43326500
H	-1.74134400	3.88990300	0.10914400
C	0.92305300	1.71556100	-2.06793000
C	0.92670700	2.49557800	-3.23151300
C	2.14834400	1.34751200	-1.49897100
C	2.12618500	2.92671200	-3.79715100
H	-0.02304100	2.77307600	-3.69638700
C	3.34734900	1.78619900	-2.05577100
H	2.17206600	0.71700600	-0.61068600
C	3.33963500	2.58149500	-3.20261900
H	2.11108400	3.53719800	-4.70261400
H	4.29423300	1.50040900	-1.59230600
H	4.28102300	2.92378300	-3.63851200
C	0.37721800	4.15680300	0.31025800
C	1.54599700	4.45992400	-0.39819700
C	0.30477800	4.51165700	1.66586200
C	2.63156800	5.05551600	0.24211400
H	1.60856300	4.24105300	-1.46386500
C	1.39141300	5.09924300	2.30964600
H	-0.61791200	4.34173200	2.22739100
C	2.56277300	5.36411400	1.60029200
H	3.53539100	5.28084500	-0.32840800
H	1.31763300	5.35781700	3.36816100
H	3.41634000	5.82483500	2.10287500
C	-0.52425900	-1.41967600	0.54293700
C	-1.88757500	-1.08643700	0.78355900
C	-2.84291700	-2.22361500	0.87729500
C	-2.57972800	-3.30546600	1.72991500
C	-3.98338000	-2.23973500	0.06299000
C	-3.47091800	-4.37113900	1.79618200
H	-1.68596300	-3.29858100	2.35619500
C	-4.85948400	-3.32112500	0.11798300
H	-4.17839100	-1.41382000	-0.62136700
C	-4.61151500	-4.37960900	0.99076800
H	-3.27227200	-5.20136000	2.47702800
H	-5.74196500	-3.33377200	-0.52501200
H	-5.30720300	-5.22041100	1.03993700
N	-2.25643000	0.17946600	0.97097000
S	-3.66998200	0.69878700	1.69700800
O	-4.80756000	0.54767700	0.81222900
C	-4.03102400	-0.36371000	3.22368100
O	-3.34500600	2.00530300	2.23690400
F	-4.91018300	-1.29847800	2.93157400
F	-2.92748900	-0.91268900	3.68747600
F	-4.53808800	0.44193800	4.13512300
H	-1.93946900	1.82154200	-0.34857300
H	-0.33141000	-2.45714400	0.26809700
N	1.25884300	1.11548200	2.51240900
C	2.57690100	1.64755500	2.18053100
S	0.95256000	0.51725000	4.04123600
H	3.00264200	2.14079100	3.06176100
H	3.26627600	0.86013500	1.83066400
H	2.45575800	2.39573500	1.38552800
O	-0.49517300	0.53704100	4.19455600
C	1.47708000	-1.17791500	4.03337200
O	1.80504200	1.26580100	4.95273900
C	0.52396500	-2.18856200	3.94642700
C	2.84305700	-1.47170800	4.04938400
C	0.94582600	-3.51330800	3.86196900
H	-0.53786500	-1.93911900	3.93741200
C	3.24594200	-2.79867500	3.95939500
H	3.58367400	-0.67362100	4.13376200
C	2.30751100	-3.83940200	3.86179800
H	0.19990800	-4.30920400	3.79296800
H	4.31276300	-3.03606500	3.96853500
C	2.76547900	-5.26858700	3.78219100
H	3.17684200	-5.59480500	4.75100900
H	1.93834600	-5.94285400	3.52110200
H	3.56441300	-5.38651100	3.03489900

^{1}G

E (DLPNO-CCSD(T)) =-1986.932958
E (SMD/wB97xD/Def2TZVPP) =-1990.3227
E (gas/wB97xD/Def2TZVPP) = -1990.284398
G_{Corr}= 0.52291

C	2.03771400	1.19177400	1.52435900
C	1.03917000	2.11926200	1.65314200
H	1.11504600	2.76403900	2.53239500
C	2.19033400	0.11203300	0.54994100
H	3.19836300	0.03110900	0.12327500
N	-0.03211400	2.37364800	0.86464700
C	-0.10540600	1.98856000	-0.53268800
H	-0.23032200	0.89986300	-0.63918900
H	-1.02559000	2.43265800	-0.93898800
C	-1.03696800	3.32501400	1.35446900
H	-2.02442700	2.97647000	1.02009100
H	-1.03263800	3.28542200	2.45127800
C	1.06573300	2.42371500	-1.39433200
C	1.39905600	1.65631100	-2.51625200
C	1.80284400	3.58325900	-1.13214300
C	2.43572900	2.04224600	-3.36520500
H	0.84178200	0.73785200	-2.72241700
C	2.84650500	3.96773700	-1.97484100
H	1.56641300	4.19340000	-0.25805400
C	3.16616100	3.20039500	-3.09524500
H	2.68001000	1.42997000	-4.23677100
H	3.41277000	4.87527300	-1.75137700
H	3.98467400	3.50128200	-3.75357300

C	-0.78663800	4.73919400	0.87895900
C	-1.38364200	5.22156600	-0.29057300
C	0.12062100	5.55918700	1.56251100
C	-1.06463300	6.48842200	-0.78154000
H	-2.10308500	4.59604700	-0.82631300
C	0.43995100	6.82580200	1.07585500
H	0.58801600	5.19590300	2.48185000
C	-0.14807600	7.29088000	-0.10224900
H	-1.53509300	6.85008300	-1.69902100
H	1.15062500	7.45395800	1.61838000
H	0.10368900	8.28230100	-0.48642700
C	1.32170900	-0.84287400	0.14188400
C	-0.01695000	-1.17054100	0.67350900
C	-0.71810900	-2.33755700	0.04108400
C	-0.02123900	-3.46425800	-0.41755000
C	-2.11714100	-2.32783200	-0.04944700
C	-0.70859500	-4.55567000	-0.94941300
H	1.06701200	-3.50994100	-0.33916300
C	-2.80249100	-3.41058100	-0.59530500
H	-2.66300900	-1.45431500	0.31315000
C	-2.09967100	-4.53015100	-1.04534300
H	-0.15056900	-5.43161000	-1.28882700
H	-3.89228600	-3.38176800	-0.66952400
H	-2.63656000	-5.38187900	-1.46982200
N	-0.62278100	-0.58696900	1.64393400
H	-0.05662200	0.17656600	2.01536000
N	3.14091400	1.36185400	2.43495700
C	4.42922700	1.78363000	1.89676100
S	3.10591800	0.42384300	3.81453400
H	5.10392900	2.05030700	2.71791000
H	4.91375900	1.01151000	1.27366200
H	4.25823300	2.67671700	1.27953600
O	1.84382200	0.69012500	4.49402600
C	3.05892000	-1.26555500	3.25885700
O	4.36386200	0.65729300	4.51585400
C	1.87994100	-1.99356200	3.37452500
C	4.18162000	-1.80758600	2.62612000
C	1.81371900	-3.27161000	2.82278200
H	1.01132000	-1.55337300	3.86517200
C	4.09719900	-3.08077600	2.07736900
H	5.10896100	-1.23535200	2.55168900
C	2.91010800	-3.82877900	2.15622300
H	0.88249700	-3.83855800	2.89786900
H	4.96742500	-3.50544500	1.57022900
C	2.83238200	-5.19769900	1.53990500
H	3.47022400	-5.90814700	2.09012700
H	1.80453200	-5.58586900	1.54931400
H	3.19045600	-5.18166200	0.49924700
H	1.69166300	-1.51520900	-0.63487400

^{1}TS^{-G}

E (DLPNO-CCSD(T)) = -2871.575463
E (SMD/wB97xD/Def2TZVPP) =-2876.094609
E (gas/wB97xD/Def2TZVPP) = -2876.038797
G_{Corr}= 0.533212

C	1.97837400	0.87226000	1.09246900
C	0.76593200	1.60400000	1.35965400
H	0.59701200	1.89754500	2.40011300
C	2.08879800	-0.17504700	0.18896000
H	3.10490900	-0.46911500	-0.10248800
N	0.09821200	2.34151500	0.47412200
C	0.32121400	2.23186200	-0.96110500
H	0.18766200	1.18500400	-1.28186400
H	-0.46684800	2.81888600	-1.45332400
C	-1.12224700	3.02434800	0.90604900
H	-1.98169800	2.58764400	0.37382500
H	-1.26010900	2.81664100	1.97475400
C	1.66785100	2.74911000	-1.42178100
C	2.43724800	2.01023600	-2.32522100
C	2.14857000	3.98835900	-0.98278600
C	3.66780700	2.49165300	-2.77389900
H	2.07466300	1.03766600	-2.66865800
C	3.37699200	4.47255200	-1.42996100
H	1.55911000	4.57634200	-0.27542800
C	4.14348000	3.72394200	-2.32535000
H	4.26051000	1.89662500	-3.47310400
H	3.73871800	5.44051900	-1.07455000
H	5.10875400	4.10054600	-2.67217200
C	-1.04962300	4.51488200	0.67153200
C	-1.71518200	5.11545400	-0.40037900
C	-0.25653400	5.31000000	1.50950100
C	-1.58424500	6.48529500	-0.63994800
H	-2.34058400	4.50410600	-1.05702400
C	-0.12527700	6.67665400	1.27455600
H	0.27052000	4.84708000	2.34866600
C	-0.78753100	7.26716500	0.19501700
H	-2.10707300	6.94182100	-1.48378900
H	0.49638200	7.28580400	1.93513000
H	-0.68335900	8.33866200	0.00803800
C	1.04345300	-0.97242000	-0.28595200
C	-0.19112100	-1.06395300	0.42014300
C	-1.01876800	-2.30332800	0.24307800
C	-0.43064000	-3.56295700	0.05345200
C	-2.41840600	-2.21836800	0.29115500
C	-1.21990300	-4.70534800	-0.07162900
H	0.65696400	-3.65505700	0.02719000
C	-3.20833900	-3.35945900	0.15996400
H	-2.89516800	-1.24293600	0.41812900
C	-2.61108600	-4.60763000	-0.01928700
H	-0.74297700	-5.67928400	-0.20609100
H	-4.29696700	-3.27178800	0.19285200
H	-3.22899600	-5.50318200	-0.11941000
N	-0.57510300	-0.13699100	1.26474800
H	-1.32976900	-0.45345800	1.87652100
N	3.10969100	1.26401700	1.89320900
C	4.31660100	1.70581600	1.20394800
S	3.27555300	0.44953100	3.34222400
H	5.02317100	2.13474300	1.92369400
H	4.82037500	0.89350700	0.65025600
H	4.02544000	2.48701100	0.48872300
O	2.09413400	0.75271300	4.14539200
C	3.20034200	-1.28194700	2.94108600
O	4.59717600	0.76399200	3.87657700
C	2.00801100	-1.97472500	3.12960900
C	4.30470900	-1.89641600	2.34713300
C	1.91506100	-3.29619400	2.70013500
H	1.15439600	-1.47457400	3.58862200
C	4.19243500	-3.21354500	1.91636500
H	5.23959200	-1.34772900	2.21438100
C	2.99495600	-3.93008700	2.07469000
H	0.97788400	-3.84014100	2.84049100
H	5.04994600	-3.69804600	1.44211400
C	2.88010000	-5.34089400	1.56818400
H	3.64858100	-5.98651000	2.02117900
H	1.89340600	-5.76965200	1.79129400
H	3.03191300	-5.37494400	0.47773100
H	1.27745200	-1.74699300	-1.01747300

^{1}H

E (DLPNO-CCSD(T)) =-1986.961148
E (SMD/wB97xD/Def2TZVPP) =-1990.3536
E (gas/wB97xD/Def2TZVPP) = -1990.31688
G_{Corr}= 0.527143

C	1.71791500	0.71533400	1.40033600
C	0.30061700	0.71853800	1.94367600
H	0.34382700	0.56555300	3.02838400
C	2.04224800	0.05092300	0.26814900
H	3.05428500	0.13381400	-0.13595200
N	-0.36594200	1.98781400	1.76532100
C	-0.69535500	2.42311900	0.42226200
H	-0.96630300	1.55691800	-0.20815300
H	-1.60318000	3.04469000	0.48194600
C	-1.17324700	2.50287800	2.85478900
H	-2.24197100	2.55382200	2.57554800
H	-1.10299200	1.79687200	3.69663100
C	0.37586100	3.22406100	-0.29788700
C	0.88894600	2.78497600	-1.52250000
C	0.86185300	4.42471700	0.23690100
C	1.87288000	3.51498400	-2.19287200
H	0.52112000	1.84788000	-1.95041500
C	1.84018400	5.15968700	-0.43007500
H	0.47245800	4.78530200	1.19120000
C	2.35350300	4.70507600	-1.64714200
H	2.26713600	3.14918400	-3.14428700
H	2.20717700	6.09267600	0.00505100
H	3.12494000	5.27775600	-2.16770600
C	-0.72030500	3.86960600	3.32947100
C	-1.53630600	4.99516500	3.19999100
C	0.56969100	4.02841300	3.85476700
C	-1.07413700	6.25974000	3.57759700
H	-2.54265500	4.88473900	2.78530300
C	1.03459100	5.28484100	4.23173700
H	1.22197200	3.15711800	3.94527600
C	0.21307400	6.40802700	4.09075000
H	-1.72247600	7.13216000	3.46337300
H	2.04526500	5.39088700	4.63418200
H	0.57860000	7.39586300	4.38185000
C	1.08534100	-0.76329100	-0.43389200
C	-0.13491600	-0.97187400	0.14388400
C	-1.17586900	-1.84173300	-0.46017500
C	-1.35339000	-1.87487100	-1.85149600
C	-1.99483700	-2.64993900	0.34339600
C	-2.32104600	-2.69821300	-2.42352800
H	-0.73897100	-1.23608500	-2.48981600
C	-2.96255600	-3.47235000	-0.23121500
H	-1.86373000	-2.65481200	1.42830500
C	-3.12924200	-3.49978000	-1.61595700
H	-2.45029800	-2.70664800	-3.50836100
H	-3.58615800	-4.10067300	0.40905400
H	-3.88913200	-4.14335400	-2.06538700
N	-0.41632700	-0.41788100	1.36219600
H	-1.36909700	-0.51369100	1.69696100
N	2.64110800	1.58861100	2.05428300
C	3.58174600	2.34843800	1.23178300
S	3.27273700	1.06138900	3.51627000
H	3.95170500	3.21185500	1.79716800
H	4.44132000	1.74477900	0.88959400
H	3.04008100	2.70896000	0.35045500
O	2.17726000	0.49361500	4.29406700
C	4.39227200	-0.25172900	3.10302300
O	4.03757100	2.17213300	4.06763100
C	3.89292000	-1.54337200	2.91606000
C	5.74096100	0.03477100	2.89593600
C	4.76219700	-2.55106900	2.51396300
H	2.83647400	-1.75695000	3.08707200
C	6.59689100	-0.98867600	2.49553500
H	6.11872800	1.04666600	3.05279700
C	6.12435500	-2.29227800	2.29678800
H	4.37591400	-3.56271100	2.36592500
H	7.65506400	-0.76805600	2.33469100
C	7.05392000	-3.40009800	1.88650900
H	7.30775300	-4.02887200	2.75554300
H	6.58564000	-4.05489100	1.13698400
H	7.99239700	-3.00610900	1.47248300
H	1.35126500	-1.25123300	-1.37016300

[1]I

E (DLPNO-CCSD(T)) = -1390.800294

E (SMD/wB97xD/Def2TZVPP) =-1393.061767

E (gas/wB97xD/Def2TZVPP) = -1393.036528

G_{Corr}= 0.283171

C	0.27424300	1.07848100	-0.90345500
C	-1.01002800	1.18992300	-0.35966800
H	-1.49995100	2.16773600	-0.34189900
C	0.85924800	-0.18812900	-0.93305800
H	1.85384400	-0.33589600	-1.35804100
C	0.15448300	-1.26749100	-0.41431100
C	-1.11478800	-1.05764900	0.14115300
C	-1.91810800	-2.16965800	0.72781600
C	-1.33164500	-3.38760900	1.10311600
C	-3.29770000	-2.00314800	0.92126900
C	-2.10493300	-4.41104600	1.64883700
H	-0.25716000	-3.54318300	0.98777500
C	-4.07048700	-3.02732700	1.46312300
H	-3.75835000	-1.05549200	0.63792500
C	-3.47727400	-4.23672200	1.82851400
H	-1.62840300	-5.35008400	1.94000700
H	-5.14443200	-2.88005700	1.60046600
H	-4.08241600	-5.04014400	2.25518300
N	-1.67149500	0.16168100	0.15107400
H	0.59572500	-2.26366800	-0.45066000
N	0.93118000	2.23532300	-1.40774000
C	1.71819200	2.09323400	-2.63193100
S	1.56481300	3.28228800	-0.24904100
H	1.92578200	3.08765100	-3.04314800
H	2.67191000	1.55962300	-2.47528400
H	1.11443600	1.53405900	-3.35842700
O	0.55208200	3.44414900	0.78279100
C	2.93944200	2.40529200	0.45055000
O	2.06471000	4.43934300	-0.97556700
C	2.71979900	1.53087400	1.51546000
C	4.20699300	2.54642800	-0.11744100
C	3.78721000	0.78541400	2.00829800
H	1.72707600	1.43951100	1.95985900
C	5.26106400	1.79407700	0.38998900
H	4.36732500	3.24541000	-0.94025700
C	5.06918900	0.89992400	1.45389500
H	3.62029200	0.10297700	2.84523800
H	6.25682900	1.90620900	-0.04638600
C	6.21418700	0.07419600	1.97012800
H	7.13438200	0.67235300	2.04272000
H	5.99167900	-0.35099200	2.95852500
H	6.42289300	-0.76207300	1.28304600

[1]NH_4^+

E (DLPNO-CCSD(T)) = -56.81655671

E (SMD/wB97xD/Def2TZVPP) =-56.9862016

E (gas/wB97xD/Def2TZVPP) = -56.91072833

G_{Corr}= 0.029582

N	1.04156500	3.60719200	0.00000400
H	1.38392700	2.63960500	-0.00000900
H	1.38417000	4.09132400	0.83776400
H	1.38417600	4.09131200	-0.83776100
H	0.01558500	3.60721600	0.00000200

[1]OAc^-

E (DLPNO-CCSD(T)) = -228.1827909
E (SMD/wB97xD/Def2TZVPP) =-228.5955088
E (gas/wB97xD/Def2TZVPP) = -228.540793
G_{Corr}= 0.021068

C	-0.00277500	1.80257900	0.04534400
O	-1.13634300	2.01213100	0.52589300
O	0.90698400	1.08407000	0.51157700
C	0.33930600	2.55562200	-1.26901200
H	0.92864400	3.45739700	-1.02659900
H	0.96122100	1.93411000	-1.93287900
H	-0.56645800	2.87972200	-1.80453800

[1]HOAc

E (DLPNO-CCSD(T)) = -228.7574464
E (SMD/wB97xD/Def2TZVPP) =-229.1209448
E (gas/wB97xD/Def2TZVPP) = -229.1144942
G_{Corr}= 0.035021

C	-0.05391900	1.87450800	0.00769400
O	-1.14351400	1.92105400	0.52366800
O	0.95828200	1.18026900	0.53945900
C	0.33924200	2.55688700	-1.26762800
H	1.18468200	3.23531700	-1.08107500
H	0.67279700	1.80747800	-2.00058000
H	-0.51119700	3.11936700	-1.66857100
H	0.63498100	0.76144200	1.35512300

[1]Tf-NH_2

E (DLPNO-CCSD(T)) =-941.1022256
E (SMD/wB97xD/Def2TZVPP) =-942.3080714
E (gas/wB97xD/Def2TZVPP) = -942.3017246
G_{Corr}=0.017983

S	-4.01032900	-0.37641800	-0.24680600
O	-5.06286400	-1.14069600	-0.88394500
C	-3.86466200	-1.09674500	1.45799800
O	-4.09644600	1.05411400	-0.03618200
F	-2.83556400	-0.55751100	2.09029600
F	-4.97276500	-0.84201300	2.13462200
F	-3.69228200	-2.40589800	1.38287400
N	-2.57954400	-0.75901300	-0.89146600
H	-2.57095300	-1.49997900	-1.58994500
H	-1.90249300	-0.00513400	-0.99585400

[1]$HNBn_2$

E (DLPNO-CCSD(T)) =-596.1655118
E (SMD/wB97xD/Def2TZVPP) =-597.2987944
E (gas/wB97xD/Def2TZVPP) = -597.2824033
G_{Corr}= 0.217974

N	-1.80966400	-0.01736300	0.13848800
C	-1.37893800	-0.09684000	-1.24690400
H	-1.52156900	-1.13914700	-1.57762700
H	-1.99999600	0.53427800	-1.91619000
C	-1.97673700	1.33444500	0.65585900
H	-2.41502000	2.01831800	-0.09970000
H	-2.69345800	1.28195700	1.49021400
C	0.07416000	0.28268200	-1.45257000
C	0.44486100	1.16822700	-2.46814100
C	1.07293900	-0.23711500	-0.61952400
C	1.78040100	1.53793700	-2.64498400
H	-0.32506700	1.59000500	-3.12091800
C	2.40505800	0.13318100	-0.78682700
H	0.79012100	-0.91863100	0.18638800
C	2.76409700	1.02653100	-1.80022200
H	2.04963300	2.23790600	-3.44001300
H	3.16978200	-0.27268500	-0.11959400
H	3.80829500	1.32222000	-1.92811400
C	-0.68333300	1.93458100	1.17079200
C	-0.09275600	3.03903300	0.55181100
C	-0.03285800	1.35317400	2.26668500
C	1.13021700	3.54273400	1.00116800
H	-0.58635600	3.49964600	-0.30879900
C	1.18450200	1.85343600	2.72276600
H	-0.48258500	0.48374400	2.75465600
C	1.77427400	2.94864800	2.08584700
H	1.58403300	4.39894500	0.49577000
H	1.68051900	1.38553900	3.57701700
H	2.73333800	3.33760600	2.43667800
H	-2.68418100	-0.52444600	0.23901400

C2-selectivity

TS-2

E (DLPNO-CCSD(T)) = -2872.095396
E (SMD/wB97xD/Def2TZVPP) = -2876.629857
E (gas/wB97xD/Def2TZVPP) = -2876.562893
G_{Corr}= 0.536432

C	1.80990800	0.48849800	-1.04026300
H	2.36861400	-0.38530300	-1.38094400
C	0.47689200	0.34553600	-0.66138900
H	-0.07047000	1.24703300	-0.36038800
N	3.55128300	2.10302300	-1.59346600
C	4.43137800	1.14887200	-2.28639300
H	3.85804000	0.25276800	-2.54331700
H	4.74711000	1.62340400	-3.22452700
C	4.01889500	3.48913200	-1.52829400
H	4.56266700	3.69643900	-2.45863500
H	3.12839600	4.13233900	-1.52432000
C	5.62470600	0.81593100	-1.42055700
C	6.85542600	1.44166200	-1.63471500
C	5.47753500	-0.05571000	-0.33557300
C	7.92287000	1.21425900	-0.76567000
H	6.97249100	2.12751700	-2.47799400
C	6.54330700	-0.28587200	0.53169000
H	4.51537300	-0.54537100	-0.16102300
C	7.76633300	0.35492600	0.32159500
H	8.87838500	1.71575700	-0.93584500
H	6.41903400	-0.96499200	1.37834500
H	8.59963800	0.18098100	1.00645800
C	4.88904200	3.82047800	-0.33468300
C	5.84318100	4.83431100	-0.46975900
C	4.73013800	3.19882000	0.90630000
C	6.62348100	5.22385000	0.61751600
H	5.97692100	5.32302100	-1.43861700
C	5.51899600	3.57938900	1.99228300
H	3.99458500	2.40150900	1.03751700
C	6.46554100	4.59444500	1.85298400
H	7.36396100	6.01798500	0.49599900
H	5.38972800	3.07798100	2.95426700
H	7.08160500	4.89187700	2.70471000
C	-0.15467100	-0.88560900	-0.75412500
H	0.42892900	-1.77082300	-1.02323700
C	-1.56778600	-1.12462400	-0.53658700

C	-2.51355300	0.00522800	-0.35372900
C	-2.50749400	1.08524200	-1.24588300
C	-3.41739000	-0.00221800	0.72070800
C	-3.40358200	2.13689500	-1.07486100
H	-1.79334800	1.12007300	-2.07078300
C	-4.30634500	1.05379300	0.88881800
H	-3.41503700	-0.82788000	1.43487000
C	-4.30459800	2.12110300	-0.01212200
H	-3.35766300	2.98823500	-1.75617100
H	-4.99970700	1.04716800	1.73255700
H	-5.00112500	2.95143400	0.12602600
N	-1.89942000	-2.38749100	-0.51859100
S	-3.44130900	-2.95653100	-0.47253500
O	-4.36237000	-2.28561700	-1.37322800
C	-3.07492700	-4.58960700	-1.27890800
O	-3.83593000	-3.28031200	0.88818800
F	-2.20811000	-5.28149000	-0.56344100
F	-4.21129100	-5.26347900	-1.35683500
F	-2.59412900	-4.40511300	-2.49544500
C	2.40272500	1.79323400	-1.05277300
H	1.89400100	2.58196300	-0.50132100
N	0.52728400	2.97162000	-2.46318200
S	-0.22674900	4.02722100	-1.57191100
C	0.89930400	3.35287700	-3.80493500
O	-0.50942800	3.39045800	-0.26700900
O	-1.36267800	4.69374900	-2.24160200
C	0.92532700	5.35759400	-1.17425700
H	1.21175500	2.44990600	-4.35492600
H	0.07054400	3.81025000	-4.37975000
H	1.75453800	4.06062500	-3.85016800
C	1.54106000	5.38646000	0.07708500
C	1.29447700	6.28737700	-2.15116100
C	2.54817500	6.31486400	0.33722700
H	1.23578700	4.66834800	0.84031600
C	2.30960200	7.20255700	-1.88403200
H	0.79075900	6.29549900	-3.11998400
C	2.96207300	7.22513200	-0.64161300
H	3.03312500	6.32034900	1.31729900
H	2.60422400	7.91731100	-2.65767100
C	4.07497400	8.20330500	-0.38003400
H	4.88519000	8.08310100	-1.11628200
H	3.71577200	9.24146600	-0.46277500
H	4.50158900	8.06814900	0.62373100

^{2}C

E (DLPNO-CCSD(T)) = -2872.140812
E (SMD/wB97xD/Def2TZVPP) =-2876.661797
E (gas/wB97xD/Def2TZVPP) = -2876.605948
G_{Corr}= 0.535955

C	1.35888500	0.89536900	0.07883300
H	2.06215500	0.15744700	0.47540300
C	0.09823200	0.51054200	-0.29621200
H	-0.58106500	1.28299300	-0.66659000
N	3.23184400	2.43044700	-0.22735500
C	3.75936200	1.71075200	-1.38035800
H	3.00710500	0.97390900	-1.70445200
H	3.91518100	2.38371500	-2.24333300
C	3.74176600	3.78384000	-0.05182200
H	3.36372000	4.46063100	-0.84460900
H	3.34540000	4.16216700	0.90336400
C	5.04855100	0.96323200	-1.09678600
C	6.13262200	1.05137200	-1.97414000
C	5.17142900	0.15998000	0.04288400
C	7.31954400	0.36223100	-1.71598700
H	6.05474600	1.68465000	-2.86259600
C	6.35125200	-0.53382600	0.30267100
H	4.33864300	0.10123900	0.74781500
C	7.43319400	-0.43191900	-0.57535800
H	8.16159300	0.45402700	-2.40661300
H	6.43119200	-1.15297900	1.19979600
H	8.36260000	-0.96793100	-0.36799800
C	5.24878200	3.87218900	-0.02498700
C	5.94140900	4.53379600	-1.04175200
C	5.97844300	3.27329800	1.00968100
C	7.33756700	4.58255700	-1.03952700
H	5.38095900	5.00681300	-1.85322700
C	7.36977800	3.31549700	1.01483800
H	5.44441200	2.75243600	1.80873100
C	8.05469400	3.96741500	-0.01502700
H	7.86508500	5.09823200	-1.84569300
H	7.92619100	2.83502200	1.82338400
H	9.14703000	3.99698000	-0.01410300
C	-0.35158400	-0.81586100	-0.22485600
H	0.30269900	-1.59013000	0.18551500
C	-1.65466600	-1.24306500	-0.63526800
C	-2.58533000	-0.27650600	-1.28607000
C	-2.22109000	0.35388900	-2.48304100
C	-3.81692200	0.02044700	-0.68984500
C	-3.08698400	1.26543700	-3.08122500
H	-1.26111500	0.12196800	-2.94983200
C	-4.67364600	0.94291600	-1.28730500
H	-4.10104600	-0.46026800	0.24815000
C	-4.31220500	1.56364700	-2.48264500
H	-2.80185800	1.74770800	-4.01877000
H	-5.62810900	1.17947600	-0.81208400
H	-4.98670200	2.28522900	-2.94919600
N	-1.95555500	-2.49535300	-0.37745400
S	-3.34901700	-3.20495200	-0.87155700
O	-3.66783900	-2.99595500	-2.27432900
C	-2.73800500	-4.95542100	-0.76721400
O	-4.38784600	-3.10210500	0.14095000
F	-2.38011600	-5.25498300	0.46818200
F	-3.72835000	-5.75297500	-1.13725200
F	-1.71027200	-5.13705800	-1.57803900
C	1.82399300	2.32716800	0.05992400
H	1.67442000	2.70369100	1.08683000
N	0.93938900	3.15251400	-0.78488300
S	0.00761500	4.34762400	-0.12080600
C	1.06188500	3.08909200	-2.23297100
O	-0.16939700	4.01371700	1.28816700
O	-1.15914500	4.51313100	-0.97854900
C	0.97146600	5.83678500	-0.22142800
H	1.16246900	2.03952000	-2.54189000
H	0.15003200	3.48399500	-2.69647300
H	1.93388000	3.65327800	-2.60325800
C	1.62347500	6.31120700	0.91529800
C	1.13318900	6.46501400	-1.45712500
C	2.46797100	7.41147000	0.80296600
H	1.47684800	5.81755500	1.87730300
C	1.98652700	7.56029300	-1.55231300
H	0.59994800	6.10552800	-2.33922300
C	2.67538200	8.04389500	-0.43069600
H	2.98040200	7.78500100	1.69311500
H	2.11857700	8.05227500	-2.51918700
C	3.63226800	9.19705700	-0.54983000
H	3.62647600	9.81786700	0.35760800
H	4.66092000	8.82423700	-0.68595000
H	3.39222600	9.83216600	-1.41406200

^{1}D

E (DLPNO-CCSD(T)) = -2871.856724
E (SMD/wB97xD/Def2TZVPP) =-2876.449212
E (gas/wB97xD/Def2TZVPP) = -2876.32488

G_{Corr}= 0.537936

C	1.28341700	1.06486800	0.04593300
H	1.93602000	0.32140700	0.51743100
C	-0.00344100	0.69567200	-0.35207300
H	-0.66782500	1.44103200	-0.79472700
N	3.19470200	2.50280200	-0.30157900
C	3.68652700	1.78894100	-1.47856400
H	2.88741300	1.11015200	-1.82332800
H	3.87797200	2.48471400	-2.31446500
C	3.77557900	3.81987800	-0.04676200
H	3.40013400	4.56386600	-0.77577900
H	3.43390900	4.13953800	0.94911800
C	4.93059700	0.96094400	-1.22449700
C	6.01307400	1.02353300	-2.10569800
C	5.01619900	0.11547600	-0.11277100
C	7.16345600	0.26561700	-1.87866000
H	5.96347700	1.69010000	-2.97135100
C	6.16031500	-0.64543200	0.11638500
H	4.18729400	0.07303700	0.59748600
C	7.24113000	-0.57048900	-0.76551300
H	8.00553500	0.33680400	-2.57146400
H	6.21296200	-1.29666000	0.99240100
H	8.14265200	-1.15988300	-0.58175600
C	5.28351200	3.82894000	-0.08188800
C	5.96378100	4.47596600	-1.11619200
C	6.02288000	3.16375800	0.90369400
C	7.35861500	4.44497000	-1.17971200
H	5.39423100	5.00024200	-1.88869300
C	7.41293600	3.12437400	0.84092700
H	5.49775700	2.65505400	1.71638300
C	8.08500800	3.76218800	-0.20583200
H	7.87735500	4.95079700	-1.99757800
H	7.97793900	2.59216100	1.61002300
H	9.17594700	3.72841500	-0.25733000
C	-0.37281700	-0.61197400	-0.20323000
H	0.29591100	-1.32802800	0.28506000
C	-1.68426400	-1.15661600	-0.63607400
C	-2.54401700	-0.37295600	-1.54781400
C	-2.00516900	0.14684500	-2.73551300
C	-3.88736400	-0.13340100	-1.22288200
C	-2.81390300	0.87892900	-3.59844800
H	-0.96099000	-0.04100500	-2.99582600
C	-4.68170800	0.61906100	-2.08212000
H	-4.30636800	-0.51138100	-0.28868200
C	-4.14905500	1.11974700	-3.27019100
H	-2.39799400	1.26684400	-4.53026900
H	-5.72268100	0.81673000	-1.81944600
H	-4.77778800	1.70604300	-3.94388600
N	-1.93708000	-2.29827000	-0.09675500
S	-3.30186600	-3.20616600	-0.42353500
O	-3.60201800	-3.30274700	-1.83694700
C	-2.55608800	-4.84336200	0.05466300
O	-4.32338200	-2.91657800	0.56199300
F	-2.20810400	-4.83250600	1.32441100
F	-3.47495000	-5.76911800	-0.14665500
F	-1.50228400	-5.09306300	-0.69711200
C	1.79906100	2.44380900	-0.02782700
H	1.63556900	2.69104100	1.05762200
N	0.92203800	3.33952600	-0.76785800
S	0.06107400	4.52313300	0.03279600
C	1.04689400	3.40750700	-2.21771300
O	-0.06282000	4.05377700	1.40888300
O	-1.12553600	4.78599500	-0.76573400
C	1.08016200	5.97262900	0.02252600
H	1.08691000	2.38604200	-2.62215600
H	0.15773400	3.89628200	-2.63313100
H	1.94956800	3.95511100	-2.53348500
C	1.77721900	6.32443200	1.17769900
C	1.22602800	6.70019700	-1.15939100
C	2.65189400	7.40480400	1.13539500
H	1.64051700	5.75344500	2.09746900
C	2.11102300	7.77376200	-1.18369100
H	0.65834400	6.43676400	-2.05370600
C	2.84439200	8.13664600	-0.04500000
H	3.20001900	7.68429100	2.03854400
H	2.23224500	8.34457700	-2.10750400
C	3.83107300	9.26912000	-0.08887000
H	3.82618100	9.83950500	0.85118600
H	4.85193200	8.87703400	-0.22853400
H	3.62045000	9.95577100	-0.92043100

C3-selectivity

TS-2

E (DLPNO-CCSD(T)) = -2872.100425

E (SMD/wB97xD/Def2TZVPP) = -2876.635608

E (gas/wB97xD/Def2TZVPP) =-2876.570092

G_{Corr}= 0.537928

C	2.14658800	2.26620300	0.80626700
H	1.95932900	1.80458300	1.77657800
C	0.27367400	0.98032000	-0.15831400
H	-0.39713800	0.92329800	-1.02011600
N	3.05583500	3.21346000	0.80116800
C	3.50617900	3.86906300	-0.42397700
H	4.58476100	4.04811600	-0.31364900
H	3.39376600	3.14753100	-1.24628300
C	3.69761900	3.62814200	2.05598600
H	3.43596800	2.88854600	2.82276800
H	4.78499700	3.59398500	1.89965700
C	2.81183000	5.17983600	-0.74054600
C	1.49933200	5.45194400	-0.34331500
C	3.51394300	6.15169000	-1.46213800
C	0.90574500	6.67681000	-0.64904200
H	0.93460900	4.71504900	0.23272400
C	2.91914700	7.37262800	-1.77668800
H	4.54506800	5.95457400	-1.76830600
C	1.61300500	7.64180200	-1.36587100
H	-0.11625900	6.87762600	-0.31895200
H	3.48426000	8.12257400	-2.33541200
H	1.14891100	8.60260300	-1.60058800
C	3.25906800	5.01236500	2.47557600
C	4.04116200	6.13185900	2.17949100
C	2.01633500	5.19074900	3.09294700
C	3.57911400	7.41474200	2.47462800
H	5.01262700	5.99828400	1.69593300
C	1.55421300	6.47052100	3.39313300
H	1.39978000	4.31818600	3.32716600
C	2.33261900	7.58607100	3.07699200
H	4.19365400	8.28394100	2.22881200
H	0.58089100	6.59985000	3.87233000
H	1.96792400	8.59049500	3.30487900
C	-0.00962800	0.20370000	0.93445500
H	0.59283700	0.26465700	1.84350200
C	-1.07362800	-0.74836700	0.99561900
C	-1.84960900	-1.09905600	-0.22595100
C	-1.18002200	-1.52197600	-1.38241500
C	-3.24943200	-1.03045300	-0.22564700
C	-1.90146500	-1.89191900	-2.51452600
H	-0.09084400	-1.58006500	-1.38774100
C	-3.96542600	-1.38150100	-1.36760000
H	-3.78050600	-0.69441800	0.66677600
C	-3.29476400	-1.81937400	-2.51031400

H	-1.36936700	-2.23715200	-3.40384200
H	-5.05550800	-1.31383100	-1.36333800
H	-3.86034700	-2.10315900	-3.40085500
N	-1.29214900	-1.29980100	2.17439000
S	-2.28782000	-2.55105400	2.43703500
O	-2.14218900	-3.67002800	1.51422300
C	-1.50296300	-3.12869900	4.01768000
O	-3.62483600	-2.12823200	2.83171500
F	-1.56206700	-2.18823500	4.94439400
F	-2.16995000	-4.19306700	4.43971200
F	-0.23626100	-3.46721100	3.82716800
C	1.42040000	1.82679200	-0.34561600
H	1.31550900	2.55569800	-1.15195600
N	2.71497200	0.86790800	-1.45335000
S	3.62147700	-0.29334500	-0.77955400
C	2.18321000	0.63899600	-2.77892600
O	3.90341500	0.13232800	0.59481200
O	4.75632900	-0.59260100	-1.65847500
C	2.66660300	-1.80289500	-0.68242500
H	1.74827600	1.58167200	-3.14584400
H	2.98307700	0.35140800	-3.48071100
H	1.39363900	-0.13580300	-2.81763900
C	2.05774600	-2.16406000	0.51969500
C	2.51003700	-2.59657300	-1.81999300
C	1.26411700	-3.30543900	0.56942700
H	2.20712100	-1.55079300	1.40881000
C	1.71265700	-3.73784000	-1.75511400
H	3.01196400	-2.32934000	-2.75181500
C	1.06905800	-4.10424900	-0.56621700
H	0.76332700	-3.56962600	1.50250300
H	1.58198900	-4.35123500	-2.65033900
C	0.18318700	-5.31601700	-0.49290300
H	-0.75954100	-5.06723000	0.01644600
H	-0.04431000	-5.71117700	-1.49277800
H	0.66734200	-6.11855600	0.08704600

^{2}C

E (DLPNO-CCSD(T)) = -2872.140399
E (SMD/wB97xD/Def2TZVPP) =-2876.66157
E (gas/wB97xD/Def2TZVPP) =-2876.607654
G_{Corr}= 0.539553

C	1.41241800	0.47320700	-0.91722100
C	2.13504600	1.64081800	-0.70395500
H	1.69924500	2.43774300	-0.09428800
C	0.14872200	0.21598000	-0.40057600
H	-0.37623900	0.96678800	0.19589500
N	3.38579200	1.91390500	-1.18265100
C	4.10299100	0.98809200	-2.03615900
H	3.37437300	0.47540800	-2.68460200
H	4.74600200	1.57865500	-2.70643400
C	4.13739900	3.02518100	-0.61596600
H	4.75194600	3.47140300	-1.41238800
H	3.41708400	3.78732300	-0.28458000
C	4.96322400	-0.04856000	-1.33024700
C	6.21037100	-0.38352100	-1.86657800
C	4.54870800	-0.68702600	-0.15469500
C	7.02479000	-1.33502800	-1.25205500
H	6.55553500	0.11693600	-2.77565500
C	5.36180000	-1.63448800	0.46559500
H	3.58920800	-0.42969200	0.29689000
C	6.60296700	-1.96471600	-0.08167700
H	7.99738400	-1.57861700	-1.68677600
H	5.02281000	-2.11584400	1.38631500
H	7.24039500	-2.70589400	0.40628600
C	5.02295100	2.60899900	0.54297900
C	6.39660900	2.42218900	0.36453500
C	4.45749800	2.31587500	1.78983900
C	7.18904700	1.92808100	1.40155300
H	6.84794200	2.64630300	-0.60611600
C	5.24545800	1.82449300	2.82937800
H	3.38448000	2.46213100	1.94341600
C	6.61370100	1.62269200	2.63520700
H	8.25943200	1.77562000	1.24288900
H	4.79023600	1.59566100	3.79615400
H	7.23125700	1.23107400	3.44713000
C	-1.78527400	-1.27470400	0.19493600
C	-2.82491300	-0.24978800	-0.05010900
C	-3.13285800	0.14547600	-1.36033100
C	-3.47052200	0.36628600	1.03165100
C	-4.09999200	1.12134400	-1.58188700
H	-2.63706400	-0.32743400	-2.21036700
C	-4.41966300	1.35843900	0.80294400
H	-3.21630400	0.08570300	2.05581600
C	-4.74030000	1.73177300	-0.50224700
H	-4.35173500	1.41009100	-2.60439900
H	-4.91020200	1.84232000	1.64996400
H	-5.49061900	2.50558800	-0.67948600
N	-1.86683800	-2.26334500	1.00983800
N	0.44180500	-2.23095900	-0.30652500
C	1.19407700	-2.29934300	0.93725500
H	0.76168400	-3.02446800	1.64072100
H	2.24683800	-2.55889000	0.74489500
H	1.17896700	-1.30365800	1.39877200
S	0.55779100	-3.50997100	-1.34242600
O	-0.56715400	-3.42174900	-2.26813100
C	2.04774800	-3.21778300	-2.27096200
C	2.00498600	-2.35197000	-3.36649300
C	3.23923900	-3.82968100	-1.88829000
C	3.17170200	-2.10514700	-4.08000800
H	1.06632900	-1.88068300	-3.66418100
C	4.39885200	-3.57430700	-2.61885000
H	3.25801600	-4.50921900	-1.03463700
C	4.38434900	-2.71662000	-3.72465900
H	3.14097900	-1.42978800	-4.93885600
H	5.33386100	-4.05547600	-2.32177000
C	5.62331200	-2.47883400	-4.54125300
H	5.70020700	-1.42705700	-4.85286200
H	6.53111700	-2.74899500	-3.98477800
H	5.59686200	-3.08977600	-5.45872900
H	1.88937400	-0.33714300	-1.47896400
O	0.76304700	-4.71630000	-0.55180900
S	-3.22215900	-2.74340300	1.81348200
O	-4.44217200	-2.71176200	1.02731500
C	-2.74746400	-4.53771600	1.90997600
O	-3.20128600	-2.25309100	3.18047400
F	-1.62819300	-4.68784400	2.59424400
F	-3.72410000	-5.18262100	2.52732100
F	-2.59397700	-5.03505300	0.69693700
C	-0.48230800	-1.14334300	-0.58096000
H	-0.79565700	-1.26819100	-1.63461500

^{1}D

E (DLPNO-CCSD(T)) = -2871.94498
E (SMD/wB97xD/Def2TZVPP) = -2876.536166
E (gas/wB97xD/Def2TZVPP) = -2876.407642
G_{Corr}=0.543454

C	1.34940500	0.20412000	-0.92253500
C	2.02620300	1.45581600	-0.72361700
H	1.47927700	2.27602100	-0.24824800
C	0.05965800	0.06933800	-0.57196600
H	-0.50093800	0.92765700	-0.18894100
N	3.26003900	1.68679200	-1.03943800

C	4.10866800	0.70470000	-1.73383000
H	3.47108300	0.11928500	-2.40961600
H	4.79631500	1.27945000	-2.36638900
C	3.88177700	2.99416300	-0.74942900
H	4.10201700	3.46818200	-1.71637500
H	3.13889900	3.60336400	-0.22002700
C	4.89579400	-0.21691900	-0.82457200
C	6.22547300	-0.49921700	-1.15071500
C	4.32777300	-0.83991800	0.29240100
C	6.96864500	-1.40219700	-0.39102600
H	6.68645400	-0.00594800	-2.00983100
C	5.06996800	-1.74079300	1.05420400
H	3.30129300	-0.62516100	0.59488100
C	6.39136300	-2.02956600	0.71223800
H	8.00606100	-1.61109100	-0.66209500
H	4.60985400	-2.21791900	1.92255800
H	6.97200800	-2.73596200	1.30984100
C	5.14322300	2.82689500	0.06169500
C	6.39410300	3.05971900	-0.51424000
C	5.06883200	2.37286500	1.38328000
C	7.56038600	2.82730300	0.21612200
H	6.45593100	3.41202600	-1.54721700
C	6.23147800	2.13314800	2.11094300
H	4.09141200	2.18681900	1.83721600
C	7.48038300	2.35572100	1.52563100
H	8.53468700	3.00675300	-0.24418000
H	6.16521200	1.76780200	3.13827600
H	8.39280800	2.16238200	2.09461700
C	-1.92273400	-1.35206700	0.14218200
C	-2.89733700	-0.24324700	0.05092700
C	-3.24015500	0.30147800	-1.19614300
C	-3.45443000	0.29235900	1.22176200
C	-4.15336200	1.34930100	-1.26704000
H	-2.81673100	-0.10675600	-2.11614400
C	-4.35000800	1.35410800	1.14324800
H	-3.17379200	-0.10762200	2.19815600
C	-4.70493200	1.87884500	-0.09971600
H	-4.43396200	1.75584700	-2.24069500
H	-4.77243700	1.77323300	2.05859300
H	-5.41361300	2.70791200	-0.15921000
N	-2.03157400	-2.41258900	0.85229200
N	0.23799100	-2.37099800	-0.52427500
C	0.93510200	-2.59324400	0.73816200
H	0.50396000	-3.43451900	1.29621800
H	2.00741600	-2.77348300	0.56659300
H	0.84737500	-1.68670900	1.34989200
S	0.38157100	-3.53876000	-1.69571900
O	-0.62205700	-3.23661100	-2.71045100
C	1.99170800	-3.26021100	-2.39335200
C	2.12393900	-2.37369500	-3.46420400
C	3.10606600	-3.88604200	-1.83574200
C	3.39152900	-2.11343900	-3.97155900
H	1.24394800	-1.89502900	-3.89767300
C	4.36928500	-3.60572800	-2.35230400
H	2.98895900	-4.58960900	-1.00976000
C	4.53300300	-2.72112000	-3.42533400
H	3.49938600	-1.42289200	-4.81179900
H	5.24513600	-4.08844500	-1.91211300
C	5.89079700	-2.44478300	-4.00634600
H	6.01638900	-1.37559200	-4.23128700
H	6.69300900	-2.75814100	-3.32454800
H	6.01811500	-2.99378900	-4.95378900
H	1.89917400	-0.65551000	-1.30922800
O	0.39983800	-4.83580300	-1.03743400
S	-3.37573500	-2.88534100	1.68663400
O	-4.62638500	-2.69079200	0.97785600
C	-3.02314500	-4.70786600	1.58101500
O	-3.24165400	-2.53107800	3.08774800
F	-1.88248500	-4.99488600	2.17956700
F	-4.00859500	-5.34396700	2.19131100
F	-2.96969300	-5.08805400	0.31887300
C	-0.65154700	-1.25638000	-0.70274800
H	-1.03190300	-1.30183800	-1.74263600

C4-selectivity

TS-2

E (DLPNO-CCSD(T)) = -2872.097562
E (SMD/wB97xD/Def2TZVPP) =-2876.615817E
(gas/wB97xD/Def2TZVPP) = -2876.563313
G_{Corr}= 0.538517

C	2.61949500	-0.45235300	-0.07551000
H	2.88780700	-0.59394800	0.97211200
C	3.56783900	-0.01637400	-0.98532900
H	3.28047700	0.13898300	-2.02662100
N	4.84287900	0.25995600	-0.73140600
C	5.40999300	0.13893300	0.60236800
H	5.02069200	-0.77977400	1.06862200
H	6.49272400	-0.01322100	0.48954800
C	5.73183500	0.73860600	-1.79130500
H	6.59688500	0.06065000	-1.85218400
H	5.18511300	0.67262900	-2.74105200
C	5.16397500	1.31979900	1.52614800
C	5.94299600	1.43454300	2.68432500
C	4.17818900	2.27924200	1.27933200
C	5.73808800	2.48347700	3.57933900
H	6.72284200	0.69414400	2.88470400
C	3.97262400	3.33015400	2.17525500
H	3.56010700	2.22373900	0.38107800
C	4.74918500	3.43702500	3.32792600
H	6.35773300	2.55902000	4.47630200
H	3.19278300	4.06546300	1.96178200
H	4.58834100	4.26057000	4.02776500
C	6.20298200	2.15611800	-1.55017500
C	7.51082300	2.41146400	-1.13035100
C	5.31137500	3.22635900	-1.69552700
C	7.92510900	3.71604800	-0.85297000
H	8.21138100	1.57994000	-1.01229600
C	5.72322800	4.52822600	-1.42108800
H	4.27584400	3.03216300	-1.98929400
C	7.03116800	4.77609900	-0.99642600
H	8.94928200	3.90250700	-0.52096100
H	5.01766700	5.35539200	-1.53129300
H	7.35166500	5.79740300	-0.77668500
C	0.71068100	-0.62350800	-1.72563700
H	1.30573000	-0.31090000	-2.58609500
C	-0.61291800	-1.06363000	-2.02820900
C	-1.52215300	-1.55745000	-0.95597600
C	-2.70923300	-0.86787500	-0.68404000
C	-1.20135500	-2.71077900	-0.22973500
C	-3.56468500	-1.32759200	0.31519600
H	-2.95581400	0.03267900	-1.24849800
C	-2.06830000	-3.17370600	0.75670100
H	-0.27783700	-3.25188200	-0.44893500
C	-3.24809200	-2.48070100	1.03302800
H	-4.48454300	-0.78039900	0.53236100
H	-1.82055600	-4.08016600	1.31324500
H	-3.92340000	-2.84185900	1.81206400
N	-0.95960100	-0.99248400	-3.29422100
S	-2.35330700	-1.64757600	-3.86776000
O	-3.46653800	-0.71285800	-3.84631900
C	-1.81447000	-1.74464200	-5.64295600
O	-2.55745000	-3.02804700	-3.46098500
F	-0.76703400	-2.54156400	-5.75904200

F	-2.82164800	-2.24399300	-6.34231500
F	-1.50907400	-0.55063900	-6.11783400
C	1.25046600	-0.57079100	-0.40012400
H	0.62589700	-1.01306600	0.37661600
N	0.22451900	1.25395400	0.07660100
S	0.71286400	2.51421500	-0.83801600
C	0.50833300	1.36912000	1.49466800
O	2.07914100	2.37272200	-1.37660700
O	0.44010800	3.77046000	-0.12808000
C	-0.39043500	2.40916200	-2.22909400
H	1.58440600	1.32657800	1.74543800
H	0.10959800	2.31633000	1.89209100
H	-0.00115200	0.54399600	2.01424100
C	0.12249900	2.36199000	-3.51999900
C	-1.76909500	2.43935500	-2.00583300
C	-0.75755800	2.32025300	-4.60177500
H	1.20191300	2.34502700	-3.67753100
C	-2.63131800	2.39210600	-3.09204600
H	-2.16076300	2.48222700	-0.98752200
C	-2.14179200	2.32648300	-4.40718700
H	-0.35562000	2.27013200	-5.61666300
H	-3.71065800	2.39822700	-2.92129900
C	-3.09858700	2.23442000	-5.56144200
H	-3.62859200	1.27018400	-5.52588600
H	-3.85828000	3.02936900	-5.50982400
H	-2.57994600	2.30990300	-6.52700100

^{2}C

E (DLPNO-CCSD(T)) = -2872.120701
E (SMD/wB97xD/Def2TZVPP) =-2876.626834
E (gas/wB97xD/Def2TZVPP) =-2876.588279
G_{Corr}= 0.538756

C	2.20661800	0.81367600	-1.00789700
H	2.72455500	-0.13956100	-0.86890400
C	2.88463500	1.95030000	-1.27312000
H	2.33206600	2.89127000	-1.34865800
N	4.22771200	2.10512200	-1.48115300
C	5.14065300	0.98471000	-1.44772400
H	4.61169700	0.09689100	-1.83137700
H	5.95554300	1.18520300	-2.16096100
C	4.79267800	3.44672500	-1.47742100
H	5.65197400	3.46292600	-2.16500800
H	4.03478700	4.13529900	-1.87951400
C	5.75466500	0.66669200	-0.09270100
C	7.07929100	0.22341600	-0.02238800
C	5.03709700	0.82275500	1.09988600
C	7.67905600	-0.05236100	1.20749400
H	7.65763600	0.10931100	-0.94394500
C	5.63476600	0.55498200	2.33037800
H	4.00642500	1.18144700	1.06934600
C	6.95948300	0.11740000	2.38988600
H	8.71807000	-0.38956600	1.24112200
H	5.06263500	0.69560500	3.25083200
H	7.42938800	-0.08666800	3.35502500
C	5.22949700	3.91268300	-0.10165100
C	6.57419000	3.87404900	0.27799500
C	4.27214600	4.30669900	0.84106800
C	6.95605700	4.19461600	1.58141400
H	7.32971600	3.56796400	-0.45112500
C	4.64873300	4.62860100	2.14416900
H	3.21773500	4.35019800	0.55223500
C	5.99257900	4.56631300	2.51965800
H	8.00992000	4.14814600	1.86673200
H	3.89058600	4.93005500	2.87123000
H	6.28828900	4.81276800	3.54231300
C	0.08565900	-0.15258200	-1.87826200
H	0.59795400	-0.28400800	-2.83440600
C	-1.05689500	-0.96344200	-1.67523800
C	-1.82680100	-0.90519400	-0.39676300
C	-2.52750000	0.25248100	-0.03654700
C	-1.84211300	-2.02057700	0.45021400
C	-3.24454000	0.28608300	1.15713000
H	-2.50810600	1.13012500	-0.68291900
C	-2.55173100	-1.97525900	1.64889900
H	-1.29252400	-2.92244400	0.17475500
C	-3.25616400	-0.82472900	2.00199700
H	-3.79592100	1.18895800	1.42864100
H	-2.55237400	-2.84505000	2.30942300
H	-3.81545900	-0.79243700	2.93985300
N	-1.35963000	-1.76365200	-2.68228200
S	-2.70970800	-2.69801900	-2.69095000
O	-3.93669700	-1.94209500	-2.50572700
C	-2.66221200	-3.14223600	-4.49600000
O	-2.50478900	-3.95932600	-1.99776700
F	-1.48815000	-3.64822000	-4.82646900
F	-3.60220300	-4.04952100	-4.70867400
F	-2.90915500	-2.08342900	-5.24802100
C	0.71050700	0.73351700	-0.83284700
H	0.53011200	0.19378300	0.11615500
N	-0.01139100	1.99309200	-0.60624300
S	-0.42249300	3.14613600	-1.74676200
C	0.07861700	2.53409300	0.74593500
O	0.61711200	4.17261400	-1.86102200
O	-1.75977800	3.61737300	-1.39085600
C	-0.50917300	2.24817700	-3.26496400
H	1.11725100	2.77157200	1.02979100
H	-0.52994900	3.44395900	0.82512800
H	-0.32955000	1.79667200	1.45343800
C	0.61511600	2.16803900	-4.08732700
C	-1.71846600	1.65242400	-3.62635100
C	0.53385600	1.42273900	-5.25886200
H	1.54280800	2.67185200	-3.81144300
C	-1.78101400	0.91803600	-4.80270500
H	-2.59588000	1.74721400	-2.98439800
C	-0.65436800	0.77352900	-5.62579200
H	1.41224500	1.34359100	-5.90378300
H	-2.72078300	0.43753400	-5.07986400
C	-0.71276300	-0.08367900	-6.85753900
H	0.01856400	0.24277200	-7.61008700
H	-0.47931300	-1.12890400	-6.59687200
H	-1.71561300	-0.07345900	-7.30695600

^{1}D

E (DLPNO-CCSD(T)) = -2871.940957
E (SMD/wB97xD/Def2TZVPP) = -2876.527337
E (gas/wB97xD/Def2TZVPP) = -2876.406614
G_{Corr}= 0.546244

C	0.58012000	1.65405800	-1.32783200
H	1.08188300	1.47386300	-2.27723500
C	1.38905200	2.11409900	-0.21259800
H	0.91179000	2.26386400	0.76185900
N	2.65411700	2.34419500	-0.26619900
C	3.42780600	2.26497400	-1.51200300
H	2.83453900	2.74333200	-2.30334300
H	4.31627400	2.89238500	-1.36901100
C	3.40332800	2.70804500	0.95511700
H	3.90249200	3.66538000	0.75472900
H	2.67146200	2.84855900	1.75916200
C	3.84184100	0.87305700	-1.94124400
C	4.81111200	0.78273400	-2.94829100
C	3.28565000	-0.29986300	-1.42619500
C	5.21028600	-0.45801200	-3.43784900

H	5.25590900	1.69662900	-3.35086100
C	3.68900500	-1.54402400	-1.91602400
H	2.53768300	-0.27077900	-0.63133200
C	4.64757100	-1.62785700	-2.92334700
H	5.96787400	-0.51155600	-4.22307500
H	3.24578800	-2.45145100	-1.50098600
H	4.96087700	-2.60247200	-3.30437100
C	4.40179800	1.63036100	1.30093000
C	5.75718800	1.79081900	1.00063500
C	3.96107400	0.43545100	1.88091300
C	6.66379300	0.76520300	1.27069200
H	6.10411100	2.72314000	0.54738400
C	4.86717800	-0.58577400	2.15650500
H	2.90048500	0.29464800	2.10734900
C	6.21929200	-0.42398500	1.84745400
H	7.72100500	0.89704700	1.02945600
H	4.51593000	-1.51416300	2.61121200
H	6.92821400	-1.22783300	2.05937900
C	-0.57745000	0.70724600	-1.03857900
H	-0.71373600	-0.03866300	-1.82902900
C	-0.76142500	0.14142900	0.34114300
C	-1.93385100	0.55642700	1.13314600
C	-3.18131000	0.68906000	0.50418700
C	-1.80361800	0.86541300	2.49554200
C	-4.29182600	1.08419800	1.24136000
H	-3.28515200	0.45919000	-0.55726100
C	-2.91389200	1.28705000	3.22032200
H	-0.83099200	0.80691900	2.98732200
C	-4.15813300	1.38778600	2.59777200
H	-5.26519800	1.16405700	0.75346200
H	-2.80482700	1.54044600	4.27657700
H	-5.02921000	1.71246400	3.17127700
N	0.17845400	-0.66046300	0.70641200
S	0.17757200	-1.58899800	2.07360400
O	-1.09393500	-2.21508800	2.37991800
C	1.24282400	-2.95155300	1.38889800
O	0.96424400	-0.94939200	3.11556500
F	2.44822700	-2.49889700	1.09923100
F	1.33781500	-3.88614800	2.31712400
F	0.69107300	-3.46013900	0.30372400
C	-0.87332900	2.11836300	-1.38657400
H	-1.19052300	2.77143900	-0.56356800
N	-1.41796900	2.38911600	-2.67747300
S	-0.78001700	3.69393900	-3.50419600
C	-2.79670800	1.98291400	-2.92453800
O	-0.26945500	4.66813700	-2.54580000
O	-1.77578200	4.10193800	-4.48550800
C	0.61842800	3.00126600	-4.35861500
H	-2.87655400	0.89806500	-2.76715700
H	-3.50661300	2.50086000	-2.25924400
H	-3.05919500	2.20104400	-3.96506000
C	1.79711700	3.73917700	-4.42873100
C	0.51236800	1.75141000	-4.97093100
C	2.88910600	3.20693700	-5.11197300
H	1.86961800	4.70975100	-3.93512200
C	1.61419000	1.23247100	-5.64040500
H	-0.41191500	1.17378000	-4.90016200
C	2.81846700	1.94936100	-5.72345500
H	3.81988800	3.77764000	-5.15678000
H	1.54220300	0.24572500	-6.10440500
C	3.99395900	1.38178100	-6.46752000
H	4.93761600	1.84191300	-6.14306700
H	4.06458700	0.29326600	-6.32987900
H	3.88581000	1.56906300	-7.54863500

Me-substrate

^{1}1b^{Me}

E (DLPNO-CCSD(T)) = -1767.846512
E (SMD/wB97xD/Def2TZVPP) = -1770.635573
E (gas/wB97xD/Def2TZVPP) = -1770.604665
G_{Corr}= 0.327382

C	-7.12712200	-1.11364100	6.16793100
C	-6.29422000	-0.26117600	6.87670500
H	-6.64386500	0.10706600	7.84689500
C	-8.38769300	-1.43995800	6.69299100
H	-8.66444900	-0.99021500	7.65510500
N	-5.09061800	0.18072800	6.52078000
C	-4.48618700	-0.15282400	5.23878700
H	-5.29132000	-0.30365200	4.50439100
H	-3.92179300	0.72805900	4.90081600
C	-4.29261900	0.98743600	7.44829800
H	-3.87418900	1.83649500	6.88845600
H	-4.97058400	1.38732700	8.21397200
C	-3.56292200	-1.35752700	5.25325600
C	-2.46373800	-1.38068600	4.38822000
C	-3.77349700	-2.44625800	6.10542000
C	-1.58795100	-2.46604600	4.37742000
H	-2.28239500	-0.53002900	3.72515800
C	-2.89345500	-3.52822000	6.10343700
H	-4.61935300	-2.44569400	6.79645900
C	-1.79686600	-3.54194400	5.24062400
H	-0.73039400	-2.46422600	3.70016700
H	-3.06506300	-4.36440800	6.78553800
H	-1.10553300	-4.38797400	5.24330100
C	-3.18235100	0.18619400	8.09372400
C	-1.87083700	0.26583900	7.61830500
C	-3.47883600	-0.71124700	9.12576200
C	-0.87389900	-0.55473300	8.14770000
H	-1.63202900	0.96328500	6.81067000
C	-2.48527000	-1.52932700	9.65969000
H	-4.50322900	-0.77973700	9.50319400
C	-1.18074000	-1.45746900	9.16581300
H	0.14548100	-0.49151400	7.75954400
H	-2.72891300	-2.22914600	10.46265900
H	-0.40246800	-2.10398100	9.57836000
C	-10.57272700	-2.57218500	6.69657900
N	-10.89657200	-2.01100800	7.85308500
H	-6.81252200	-1.55337600	5.21828900
S	-12.30237200	-2.28571000	8.58656400
O	-12.38117500	-3.58773000	9.24157900
C	-12.07738200	-1.07148900	9.96900800
O	-13.47113200	-1.80686500	7.85496300
F	-11.94512100	0.15984800	9.50084400
F	-13.15414000	-1.12044100	10.74263200
F	-11.01475300	-1.37098900	10.69960200
C	-9.31377500	-2.28437200	6.10785700
H	-9.08382500	-2.76063100	5.15159200
C	-11.50689200	-3.49945000	5.97050200
H	-11.85255200	-4.29727500	6.64505700
H	-12.39758300	-2.94049400	5.64385300
H	-11.02876600	-3.95182900	5.09389300

C5-selectivity

TS-2^{Me}

E (DLPNO-CCSD(T)) =-2680.727133

E (SMD/wB97xD/Def2TZVPP) =-2684.871669
E (gas/wB97xD/Def2TZVPP) =-2684.831151
G_{Corr}= 0.482984

C	1.93200100	2.18244300	-1.07996700
H	1.27473600	2.08791800	-1.94901100
C	0.27727700	0.92522400	0.16608300
H	-0.32078000	0.94480400	-0.74967100
N	3.09765300	2.76799000	-1.30550000
C	4.05217200	3.05444200	-0.23809000
H	4.48371000	4.04379300	-0.44577000
H	3.49902600	3.13974100	0.70719300
C	3.49333300	3.11341100	-2.67553300
H	2.58199600	3.14210500	-3.28686900
H	3.92563300	4.12336600	-2.65607600
C	5.16752700	2.03707100	-0.09646000
C	4.94408400	0.66643500	-0.26476800
C	6.45501100	2.47660300	0.22641000
C	5.99191100	-0.24354800	-0.13131200
H	3.94830700	0.30075800	-0.52720000
C	7.50266400	1.56707200	0.37099100
H	6.64313900	3.54652200	0.35187400
C	7.27547900	0.20345100	0.18605300
H	5.80346400	-1.30951000	-0.27982800
H	8.50406900	1.92812500	0.61758600
H	8.09668800	-0.51011500	0.28662800
C	4.48437900	2.12425000	-3.25008600
C	5.84534200	2.43226000	-3.31972200
C	4.05089300	0.85589000	-3.65165600
C	6.76469400	1.48070400	-3.76268900
H	6.19093200	3.42054600	-3.00465200
C	4.96591000	-0.09562100	-4.09770900
H	2.98706200	0.60673200	-3.60031500
C	6.32691600	0.21347800	-4.14765100
H	7.82791000	1.72912500	-3.80192000
H	4.61716500	-1.08434200	-4.40527800
H	7.04607100	-0.53411500	-4.49071000
C	-0.13204000	0.19682400	1.23960700
H	0.45370500	0.22215200	2.16237200
C	-1.32027100	-0.62045700	1.28431000
N	-1.52588100	-1.24072200	2.41905700
S	-2.80611300	-2.23192300	2.64061700
O	-2.77508300	-3.41303700	1.79037800
C	-2.33315000	-2.82939500	4.33227900
O	-4.05700300	-1.50963000	2.82509300
F	-2.29195300	-1.82524000	5.18983900
F	-3.25229300	-3.69807700	4.72716400
F	-1.15626600	-3.42998300	4.30406200
C	1.46939100	1.73830200	0.18413300
H	2.20985700	1.50401800	0.95119200
N	0.99967500	3.47172100	1.09068200
S	1.27697700	3.37092800	2.69220500
C	-0.33628900	3.88114500	0.71200400
O	2.57987900	2.71185700	2.86343500
O	0.14658100	2.82744700	3.45616600
C	1.47192500	5.07595800	3.18878000
H	-0.40124000	3.86359400	-0.38685700
H	-1.13412400	3.23323600	1.11746200
H	-0.54453500	4.91764300	1.03418200
C	2.60983700	5.77883400	2.78299000
C	0.48377600	5.69653200	3.94626000
C	2.74955100	7.11188600	3.14721200
H	3.38110000	5.28530100	2.18739700
C	0.63821800	7.03668900	4.30393200
H	-0.39760100	5.13250500	4.25636100
C	1.76787500	7.76338800	3.91327900
H	3.63968500	7.66398800	2.83384200
H	-0.13764900	7.52453800	4.89960100
C	1.94725600	9.20353500	4.30727400
H	2.18916600	9.82510200	3.43166000
H	1.04253200	9.60808100	4.78164300
H	2.78145800	9.30945500	5.01932400
C	-2.21501400	-0.77351400	0.09012900
H	-2.08693800	0.03542700	-0.63645500
H	-1.97921100	-1.72735000	-0.40776000
H	-3.26943800	-0.80303100	0.39555000

C3-selectivity

TS-2^{Me} (estimation via adduct formation)

E (DLPNO-CCSD(T)) = -2680.728856
E (SMD/wB97xD/Def2TZVPP) = -2684.882689
E (gas/wB97xD/Def2TZVPP) =-2684.841634
G_{Corr}= 0.484794

C	1.91281100	0.36886900	-0.86692100
C	2.93883100	1.00493600	-0.15884700
H	2.74707900	1.27997800	0.88306200
C	0.71566300	0.08373300	-0.21416800
H	0.63974600	0.32528100	0.85139900
N	4.13773700	1.33420200	-0.61021000
C	4.59278900	0.98481500	-1.95127300
H	5.66161000	0.74029900	-1.88101200
H	4.08067100	0.06096400	-2.25260400
C	5.05351200	2.12043900	0.22552600
H	4.69465300	2.06096500	1.26107200
H	6.04462300	1.64814600	0.17753600
C	4.38850200	2.05871800	-3.00144300
C	3.26978100	2.89779400	-2.99967900
C	5.34480700	2.20906700	-4.01141800
C	3.11643300	3.87434400	-3.98354700
H	2.51698100	2.80923300	-2.21294500
C	5.18730000	3.17721700	-5.00260800
H	6.23176500	1.56965400	-4.01232000
C	4.07364700	4.01718000	-4.98862800
H	2.24295700	4.53027900	-3.96095600
H	5.94628500	3.28447500	-5.78123300
H	3.95350100	4.78466000	-5.75681900
C	5.12884000	3.56071400	-0.23166500
C	4.07845100	4.43880200	0.05778500
C	6.19720400	4.00955300	-1.01202700
C	4.08763800	5.73997700	-0.44021400
H	3.23871100	4.09393000	0.66796000
C	6.20748800	5.31096700	-1.51546600
H	7.01946400	3.32737100	-1.24423200
C	5.15029800	6.17634200	-1.23465600
H	3.26087500	6.41657500	-0.21135800
H	7.04262100	5.64780200	-2.13415000
H	5.15496100	7.19410000	-1.63202300
C	-1.63098100	-0.72625900	-0.07159700
N	-1.60641500	-0.49019200	1.21201900
N	-0.30321800	-2.75469100	-0.40936200
C	0.63466500	-2.88884500	0.67570000
H	0.74604600	-3.94747100	0.96696600
H	1.64312600	-2.48470000	0.46191300
H	0.22337500	-2.34775000	1.54260500
S	-0.00033600	-3.53931300	-1.79672100
O	-1.08040900	-3.19985700	-2.72705700
C	1.51166000	-2.92056600	-2.52866700
C	1.44470800	-2.04451400	-3.61160000
C	2.74806500	-3.34350400	-2.03689800
C	2.62137600	-1.59194300	-4.20317100
H	0.47251200	-1.73095400	-3.99584000
C	3.91689700	-2.87705400	-2.63319800
H	2.80006300	-4.04920900	-1.20559100

C	3.87418500	-2.00619800	-3.73096000
H	2.56576700	-0.90759700	-5.05380300
H	4.88408800	-3.20785400	-2.24606400
C	5.13939300	-1.54749000	-4.40137600
H	5.00908100	-0.56189500	-4.86989500
H	5.97575800	-1.49315100	-3.68985100
H	5.42900000	-2.25441700	-5.19640100
H	2.02491600	0.12333600	-1.92583000
O	0.23901300	-4.95727100	-1.50513200
S	-2.88809100	-0.77586600	2.17190900
O	-3.17743400	-2.19404400	2.33467200
C	-2.07963200	-0.26211400	3.76045500
O	-3.98275200	0.16136600	1.96954900
F	-1.03278300	-1.02806500	4.02289500
F	-1.69025400	1.00016900	3.70219600
F	-2.96604100	-0.39637800	4.73558600
C	-0.39220900	-0.51885500	-0.80700000
H	-0.43325200	-0.64272200	-1.88995800
C	-2.84804100	-1.13121300	-0.84092700
H	-3.50837700	-1.77494600	-0.24578000
H	-2.55763300	-1.66153700	-1.75519400
H	-3.40392300	-0.21783600	-1.10943700

*i*Pr-substrate

^{1}1b^{iPr}

E (DLPNO-CCSD(T)) =-1846.327895
E (SMD/wB97xD/Def2TZVPP) = -1849.269638
E (gas/wB97xD/Def2TZVPP) =-1849.23819
G_{Corr}= 0.382073

C	-7.12853700	-1.11586800	6.12270700
C	-6.30312700	-0.24922600	6.82057100
H	-6.66479500	0.14418800	7.77635300
C	-8.39886600	-1.42516000	6.63901000
H	-8.68389500	-0.95414600	7.58825200
N	-5.09282400	0.18223400	6.47052700
C	-4.47054100	-0.18640200	5.20724300
H	-5.26524800	-0.35819600	4.46603600
H	-3.90127600	0.68466400	4.85229800
C	-4.30642800	1.00886400	7.38975900
H	-3.86975300	1.83801700	6.81424800
H	-4.99599400	1.43659400	8.12974600
C	-3.54782300	-1.39007600	5.26891100
C	-2.43086000	-1.43397000	4.42791500
C	-3.77684100	-2.45840500	6.14206000
C	-1.55595200	-2.51963800	4.46072000
H	-2.23488600	-0.59927900	3.74888900
C	-2.89779800	-3.54034200	6.18389400
H	-4.63710600	-2.44090800	6.81492700
C	-1.78362300	-3.57486100	5.34435600
H	-0.68459900	-2.53398500	3.80146500
H	-3.08412800	-4.36027700	6.88169400
H	-1.09327500	-4.42090000	5.38111800
C	-3.21536500	0.21934900	8.08103800
C	-1.89314600	0.28058700	7.63352900
C	-3.53904500	-0.64860700	9.13002600
C	-0.91213600	-0.52947100	8.20705800
H	-1.63294300	0.95507700	6.81310400
C	-2.56151800	-1.45612200	9.70799900
H	-4.57171600	-0.70249400	9.48672500
C	-1.24588400	-1.40311100	9.24194400
H	0.11593500	-0.48093800	7.84030200
H	-2.82647300	-2.13281900	10.52394100
H	-0.48010400	-2.04140200	9.68921900
C	-10.58676800	-2.55184100	6.64199400
N	-10.89002500	-1.99901000	7.80508000
H	-6.80375900	-1.57940800	5.18788600
S	-12.28613200	-2.22679300	8.57188300
O	-12.47011400	-3.57388300	9.10388000
C	-11.89397500	-1.18446400	10.05372000
O	-13.42745300	-1.56658000	7.94485000
F	-11.66027900	0.07070100	9.70416900
F	-12.93998200	-1.21287100	10.86917900
F	-10.83851300	-1.65602500	10.69877700
C	-9.32261900	-2.27113000	6.05630100
H	-9.07890800	-2.75442500	5.10826500
C	-11.51414600	-3.53429300	5.93880400
H	-12.52904200	-3.36791300	6.32866900
C	-11.09746100	-4.96467700	6.30826900
H	-10.08544500	-5.18854900	5.93639200
H	-11.11341600	-5.10869900	7.39798200
H	-11.79639100	-5.68458500	5.85580100
C	-11.57931200	-3.33703200	4.42416900
H	-11.80842100	-2.29322800	4.16155000
H	-10.63915000	-3.62124300	3.92810900
H	-12.37516500	-3.97394100	4.00933100

C5-selectivity

TS-2^{iPr}

E (DLPNO-CCSD(T)) =-2759.2039
E (SMD/wB97xD/Def2TZVPP) =-2763.501842
E (gas/wB97xD/Def2TZVPP) = -2763.460726
G_{Corr}= 0.537154

C	1.92035600	2.23777500	-1.08588300
H	1.26421300	2.18598300	-1.95931400
C	0.22298600	1.00715400	0.12733200
H	-0.36859600	1.06472000	-0.79056000
N	3.10991100	2.77844900	-1.29423000
C	4.07421700	2.98683400	-0.21825200
H	4.58087500	3.94183000	-0.41632700
H	3.52365500	3.10798300	0.72544500
C	3.52275100	3.15161600	-2.65214100
H	2.61763000	3.20757700	-3.27048300
H	3.96841600	4.15505800	-2.60355100
C	5.10971600	1.88821000	-0.07236500
C	4.80702900	0.54425200	-0.31353300
C	6.40498100	2.22624600	0.33302600
C	5.78543900	-0.43902800	-0.16986300
H	3.80482000	0.25655700	-0.64060600
C	7.38219100	1.24332000	0.48715800
H	6.65507400	3.27488700	0.51636900
C	7.07682400	-0.09332500	0.22996600
H	5.53587000	-1.48270000	-0.37549800
H	8.39081500	1.52587000	0.79838000
H	7.84367900	-0.86391400	0.33848500
C	4.50590600	2.16285900	-3.23910200
C	5.87570900	2.43763100	-3.25397800
C	4.05350800	0.92346700	-3.70477600
C	6.78447600	1.48006500	-3.70568000
H	6.23582900	3.40340700	-2.88925000
C	4.95806400	-0.03356700	-4.16019300
H	2.98252200	0.70130200	-3.69625700
C	6.32736600	0.24109200	-4.15482500
H	7.85433200	1.70153000	-3.70198900
H	4.59466400	-0.99981100	-4.51801600
H	7.03814700	-0.51120000	-4.50500200

C	-0.20164800	0.26038500	1.18293100
H	0.39336600	0.25119800	2.09997500
C	-1.39960300	-0.55054100	1.22590100
N	-1.55813200	-1.18512600	2.35863800
S	-2.69615300	-2.24469800	2.81240200
O	-2.87794900	-3.38322100	1.91946100
C	-1.76096400	-2.93854700	4.25703300
O	-3.85827500	-1.59570200	3.39911500
F	-1.52692300	-2.00324300	5.16068700
F	-2.50454600	-3.88949700	4.80483600
F	-0.61252400	-3.46396600	3.86489600
C	1.43271500	1.79215200	0.16904200
H	2.15993300	1.52814600	0.93918200
N	0.98921900	3.52806700	1.07456800
S	1.28832900	3.43201900	2.67204200
C	-0.35237400	3.93865700	0.71610100
O	2.60937900	2.80527700	2.82620400
O	0.18348400	2.85696100	3.45023700
C	1.44315300	5.14157800	3.16794400
H	-0.43474500	3.91925000	-0.38149500
H	-1.14460300	3.29231000	1.13493100
H	-0.55394300	4.97586600	1.03980800
C	2.50408300	5.90336900	2.66983400
C	0.50202100	5.70429100	4.02336800
C	2.61433300	7.23697400	3.04138000
H	3.23697100	5.45666700	1.99419000
C	0.62648700	7.04584100	4.38809700
H	-0.32014100	5.09451600	4.40185800
C	1.67866400	7.83102600	3.90586600
H	3.44362300	7.83563000	2.65505200
H	-0.11211100	7.48797200	5.06170500
C	1.81975600	9.27675500	4.29483400
H	2.76977800	9.44735300	4.82538300
H	1.82735700	9.92341000	3.40352400
H	0.99851200	9.60076800	4.94880200
C	-2.29643500	-0.63950600	0.00523300
H	-2.09893400	0.26770400	-0.57962700
C	-3.79751400	-0.64164300	0.29887400
H	-4.15046800	-1.60305600	0.69382400
H	-4.07542500	0.15423400	1.00487100
H	-4.33822400	-0.46351500	-0.64241300
C	-1.86810200	-1.83944800	-0.85442000
H	-2.02681500	-2.78297500	-0.31309900
H	-2.46839600	-1.86122600	-1.77626800
H	-0.80717800	-1.76982700	-1.13670400

C3-selectivity

TS-2^{iPr}

E (DLPNO-CCSD(T)) =-2759.20611
E (SMD/wB97xD/Def2TZVPP) = -2763.512611
E (gas/wB97xD/Def2TZVPP) =-2763.471699
G_{Corr}= 0.541637

C	1.92533300	0.25013900	-0.87159900
C	2.91166600	0.92493000	-0.14462400
H	2.68326600	1.20367100	0.88895000
C	0.71454400	-0.03256800	-0.24374200
H	0.62066300	0.23187100	0.81473900
N	4.11084900	1.29506700	-0.56605900
C	4.61012700	0.95819600	-1.89276400
H	5.69213700	0.78804600	-1.80405900
H	4.16705500	-0.00349900	-2.18530800
C	4.97422600	2.11985900	0.28678100
H	4.57602300	2.07482800	1.30852300
H	5.97782500	1.67150500	0.28804400
C	4.34939100	1.99443000	-2.96852100
C	3.20570000	2.79896800	-2.97117000
C	5.28012000	2.14070000	-4.00292900
C	3.00184800	3.73626000	-3.98405900
H	2.47175900	2.71511000	-2.16657300
C	5.07285100	3.06949800	-5.02188400
H	6.18588100	1.52861700	-4.00246300
C	3.93317700	3.87428100	-5.01345200
H	2.10863700	4.36520500	-3.96464800
H	5.81282500	3.17375600	-5.81903400
H	3.77362000	4.61082100	-5.80441800
C	5.03302600	3.55073900	-0.20054900
C	3.96323600	4.41688600	0.05138900
C	6.10466500	3.99923400	-0.97675000
C	3.95708200	5.70536600	-0.47865800
H	3.12054800	4.07194900	0.65744200
C	6.09946300	5.28756900	-1.51289400
H	6.94222000	3.32629600	-1.17974600
C	5.02331700	6.14064500	-1.26892800
H	3.11551400	6.37276700	-0.27850600
H	6.93752900	5.62383100	-2.12792300
H	5.01599100	7.14815900	-1.69158700
C	-1.67315200	-0.61347400	-0.10994300
N	-1.58862600	-0.56397100	1.18807600
N	-0.25720600	-2.79169400	-0.50980100
C	0.59458100	-2.91993300	0.65140300
H	0.67165200	-3.97514900	0.96039400
H	1.62141700	-2.52999900	0.50997800
H	0.12638700	-2.36241700	1.47574500
S	0.18562100	-3.64588500	-1.82234000
O	-0.85568500	-3.48805200	-2.83796000
C	1.67371500	-2.95032500	-2.53519500
C	1.57443900	-2.09972000	-3.63492000
C	2.92470800	-3.30642200	-2.02664100
C	2.73446200	-1.61235300	-4.23418200
H	0.59218500	-1.83764400	-4.03190800
C	4.07486400	-2.80980000	-2.63264300
H	2.99933200	-3.99080400	-1.17951100
C	4.00055700	-1.97001000	-3.75399900
H	2.65422800	-0.95139300	-5.10107100
H	5.05399200	-3.09369000	-2.23787100
C	5.25467300	-1.48760600	-4.42861200
H	5.04407300	-0.67632600	-5.13878300
H	5.98917400	-1.12760000	-3.69278900
H	5.73138700	-2.31013600	-4.98617300
H	2.07152400	-0.00626000	-1.92361600
O	0.53684600	-5.00968300	-1.40568300
S	-2.87290700	-0.57395900	2.19033600
O	-3.56075200	-1.85596000	2.21987600
C	-1.90215500	-0.49986900	3.77025400
O	-3.64475400	0.66010800	2.16268800
F	-1.15109300	-1.57961800	3.91110300
F	-1.13070000	0.57362300	3.79581700
F	-2.76191300	-0.43916500	4.77544100
C	-0.40506200	-0.62672200	-0.83938900
H	-0.45174200	-0.71213900	-1.92514200
C	-2.98254100	-0.52313700	-0.86521300
H	-3.77533800	-0.87932000	-0.19053600
C	-3.04907800	-1.35758900	-2.13914800
H	-4.08152000	-1.34065200	-2.51936900
H	-2.39941200	-0.94940500	-2.92776500
H	-2.74552700	-2.39676300	-1.96716500
C	-3.23617300	0.96577900	-1.15927900
H	-2.45472100	1.37200200	-1.82034400
H	-4.20576300	1.07502300	-1.66765300
H	-3.26161900	1.55844700	-0.23434500
